# Denoising inferred functional association networks obtained by gene fusion analysis

**DOI:** 10.1186/1471-2164-8-460

**Published:** 2007-12-14

**Authors:** Atanas Kamburov, Leon Goldovsky, Shiri Freilich, Aliki Kapazoglou, Victor Kunin, Anton J Enright, Athanasios Tsaftaris, Christos A Ouzounis

**Affiliations:** 1 Computational Genomics Group, The European Bioinformatics Institute, EMBL Cambridge Outstation, Cambridge CB10 1SD, UK; 2 Computational Genomics Unit at the Center for Research & Technology Hellas, GR-57001 Thessalonica, Greece; 3 Institute of Agrobiotechnology, Center for Research & Technology Hellas, GR-57001 Thessalonica, Greece; 4 The Sanger Institute, Wellcome Trust Genome Campus, Cambridge CB10 1SA, UK; 5 Max-Planck Institute for Molecular Genetics, Ihnestrasse 63-73, D-14195 Berlin, Germany; 6 DOE Joint Genome Institute, 2800 Mitchel Drive, Walnut Creek CA 94598, USA; 7 Centre for Bioinformatics, School of Physical Sciences and Engineering, King's College London, Strand, London WC2R 2LS, UK

## Abstract

**Background:**

Gene fusion detection – also known as the 'Rosetta Stone' method – involves the identification of fused composite genes in a set of reference genomes, which indicates potential interactions between its un-fused counterpart genes in query genomes. The precision of this method typically improves with an ever-increasing number of reference genomes.

**Results:**

In order to explore the usefulness and scope of this approach for protein interaction prediction and generate a high-quality, non-redundant set of interacting pairs of proteins across a wide taxonomic range, we have exhaustively performed gene fusion analysis for 184 genomes using an efficient variant of a previously developed protocol. By analyzing interaction graphs and applying a threshold that limits the maximum number of possible interactions within the largest graph components, we show that we can reduce the number of implausible interactions due to the detection of promiscuous domains. With this generally applicable approach, we generate a robust set of over 2 million distinct and testable interactions encompassing 696,894 proteins in 184 species or strains, most of which have never been the subject of high-throughput experimental proteomics. We investigate the cumulative effect of increasing numbers of genomes on the fidelity and quantity of predictions, and show that, for large numbers of genomes, predictions do not become saturated but continue to grow linearly, for the majority of the species. We also examine the percentage of component (and composite) proteins with relation to the number of genes and further validate the functional categories that are highly represented in this robust set of detected genome-wide interactions.

**Conclusion:**

We illustrate the phylogenetic and functional diversity of gene fusion events across genomes, and their usefulness for accurate prediction of protein interaction and function.

## Background

Recently a number of genome analysis methods have been developed that predict protein-protein interactions and functional associations between proteins based primarily on genome context and not directly relying on sequence homology [1-6]. The context of a gene in a complete genome relates to its location on the genome and also its phylogenetic distribution across multiple genomes [7]. Such methods include the detection of gene clusters that are conserved across multiple genomes [4,6], the detection of gene sets which exhibit similar presence or absence patterns called phylogenetic profiles [2] and finally the detection of gene fusion events [1,5], all indicative of protein interaction or general functional association of the corresponding gene products.

The gene-fusion approach relies on the observation that pairs of genes encoding proteins of known function (usually interacting or forming a complex) tend to be found in other species as a fused composite gene encoding a single multifunctional protein. This event had been previously noted in protein evolution but not explicitly used for the prediction of protein function [8]. The fusion of two genes, which interact or are closely functionally linked, appears to decrease the regulational load in the cell, for a particular process [1,2,8] and may hence be advantageous under specific circumstances. Therefore, the detection of a gene fusion in one (query) genome allows the prediction of functional association between corresponding homologous genes that remain separate in another (reference) genome. Typically, we refer to the fused gene as a 'composite' protein, while the un-fused counterpart genes in the reference organism are referred to as 'component' proteins [1,3].

The accuracy of this approach has previously been demonstrated [3,9], however the presence of so-called 'promiscuous domains' in eukaryotic species has made it problematic to apply this method across a broad phylogenetic spectrum. These widespread domains (e.g. WD40 or TPR) are present in a multitude of combinations in many eukaryotic proteins [10] and can confound gene-fusion detection by appearing to be multiple conserved examples of gene-fusion components [3]. For this reason, we have improved our detection method to filter highly promiscuous domains from predictions.

Previously, we used sequence homology and clustering to attempt resolving this issue, however this approach is computationally intensive. For this analysis, we take a simpler approach based on the PFAM domain database [11]. By delineating the domain architecture of each protein involved in a fusion event, we are able to locate and filter those domains that appear to link an inordinate number of proteins together based on domain interaction graph connectivity analysis (see **Methods**). This approach has only recently been made feasible due to the increasing coverage of the PFAM domain database (currently 75% of proteins [11]) and the availability of large-scale computational resources.

In order to further explore the evolution and dynamics of gene fusion events in complete genomes, we have applied the approach to a set of 184 genomes. This represents the largest analysis of its type undertaken thus far. As we have previously stated, the fidelity of functional predictions based on genome context methods greatly improves with larger numbers of available genomes. Hence, we use the COGENT database [12] as our source for genome sequences, as it is constantly updated with new genomes and uses a consistent naming scheme for protein identifiers. The availability of a large number of genomes allows us to explore various aspects of fusion and function.

In this work, we explore the distribution of both fusion events and of composite and component proteins across each of the 184 genomes and their taxa. We also quantify the cumulative effect of how access to multiple genomes allows more predictions of functional association, and assess whether we are approaching the limits of prediction with larger numbers of reference genomes. Finally, we automatically assign proteins involved in fusion events with Gene Ontology (GO) [13] classifiers and show that certain classes of genes tend to be more frequently involved in fusion events than those of other classes, previously shown for individual classes [14,15], and that genes involved in fusions tend to be of the same functional class, an observation that has been previously reported (for 30 microbial genomes) [9]. We also compare these functional categories with interactions obtained from an experimental protein-protein interaction database. These results are further validated using a robust randomized trial.

## Results and Discussion

### Gene fusions

**Figure 1:**
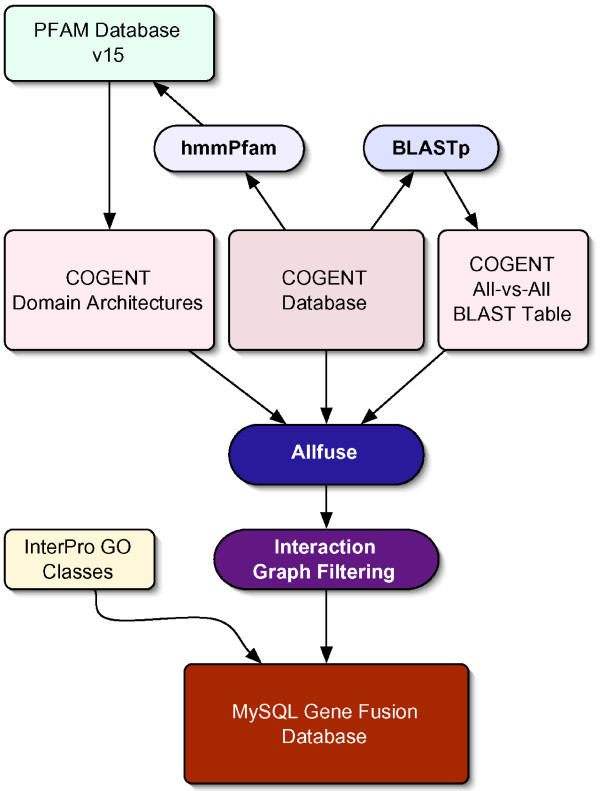
Analysis flowchart of prediction system (see **Methods **for details).

**Figure 2:**
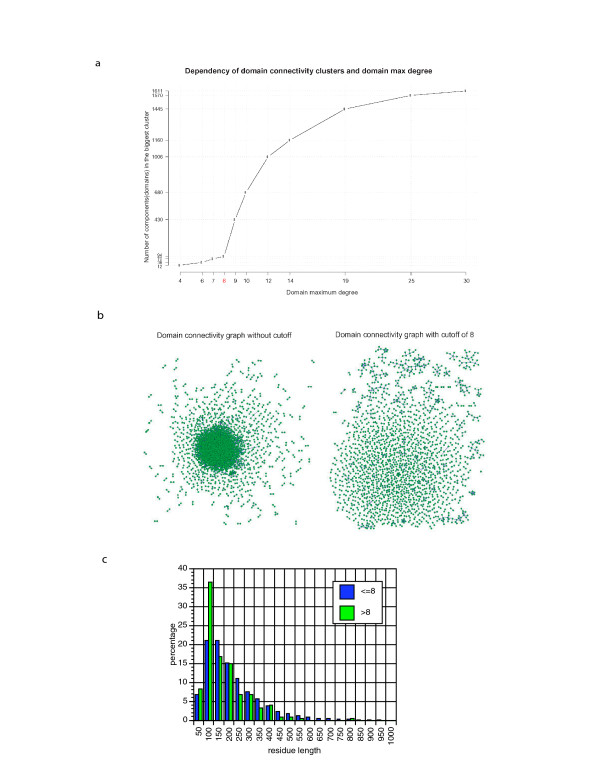
Domain Connectivity Filtering. **a) **Dependency of domain maximum degree with the number of components (domains) in the largest cluster. The threshold used for the analysis was C = 8 (shown in red). **b) **Domain connectivity graphs. Dots (nodes) indicate domains connected to other domains by virtue of a detected fusion event, lines (edges) represent the inferred functional associations obtained by fusion analysis. The leftmost graph shows connectivity of domains without cutoff, the rightmost graph shows domain connectivity with a cutoff threshold of C = 8. **c) **Length distribution of domains with connectivities of C = 8 (blue bars) and C>8 (green bars). The x-axis shows domain length bins in amino acid residues, the y-axis represents the percentage of domains of this length.

A total of 184 complete genomes from the COGENT database [12] were analyzed for fusion events (see **Methods**, Figure 1). This set comprises 148 Bacterial, 17 Archaeal and 19 Eukaryotic genomes, representing a total of 696,894 proteins. From these genomes, a total of 8,134,139 possible distinct fusion events were detected involving 275,003 proteins. In total, 130,229 composite and 235,124 component proteins were detected. These fusion events were filtered to remove those containing promiscuous domains using a threshold measure based on graph connectivity of protein domains (see **Methods**, Figure 2). Filtering the most promiscuous domains (those that interact with more than eight other distinct domains), in this fashion, removes a total of 5,942,120 interactions, leaving 2,192,019 potential interactions for further analysis. Since interaction data for all these genomes are not available, in order to validate this approach, we aim at high specificity (low number of false positives), and low sensitivity (high number of false negatives). The two million unique pairwise interactions that can be detected form a high-quality dataset upon which further analyses can be performed.

### Phylogenetic distribution of gene fusion events

**Figure 3:**
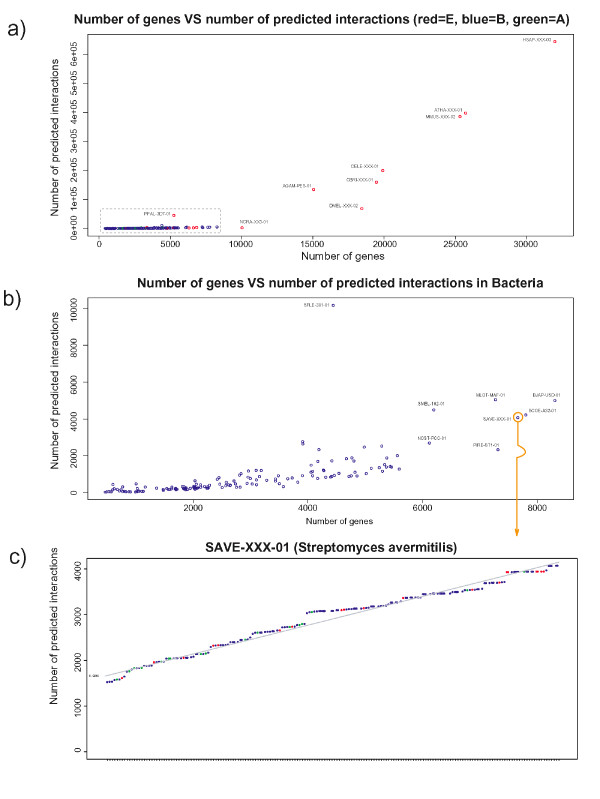
Dependency of predicted interactions and genome size. **a) **The x-axis represents the total number of genes for a given query species, the y-axis represents the total number of predicted interactions for that species. **b) **Enlargement of the dashed box from **Figure 3a **for Bacteria only. **c) **An example of a single species plot for *Streptomyces avermitilis *showing the growth of unique predicted interactions based on the number of genomes analysed. Similar plots for all 184 species are provided as Additional File 5. The distribution of slopes is provided in Additional File 3. Color coding of corresponding genomes/species as follows: Archaea (green), Bacteria (blue), Eukarya (red).

Across different genomes the pattern of detection of gene fusion events is very different. As has been observed previously [3], Eukaryota exhibit the largest numbers of predicted interactions (detected components) because they represent the largest genomes and total number of detected interactions relates strongly to genome size (Figure 3). However, a number of exceptions to this observation exist. For example; *Plasmodium falciparum *exhibits a larger number of detected interactions, than one might expect (Figure 3a), for its genome size; similarly, *Shigella flexneri *(serotype 2a T301) has the largest number of detected interactions (10,160) of any bacterium (more than twice that of the next largest), yet its genome is only about half the size of the largest bacterial genome (Figure 3b). Interestingly, the other strain of *S. flexneri *(2457T) does not exhibit such a large number of detected interactions (only 1495). Both of these serotypes are pathogenic strains isolated from infected patients, and their genomes possess large numbers of rearrangements [16], however the exact reason for such a striking difference is, as yet, undetermined.

When one examines the distribution of the fraction of detected composite (fused) proteins in each genome, compared with its genome size, a similar pattern across taxa emerges (as in the case for detected components, see above) (see Additional File 1). The larger Eukaryotic genomes tend to have a larger proportion of fused composite proteins, than smaller genomes, with human and mouse the largest, at 29% apiece. Once again, the *P. falciparum *genome is an exception to this rule with a far greater proportion of composite genes (27%) than one might expect for its genome size, perhaps due to its role as an obligate intracellular parasite. The *Bordetella pertussis *genome possesses the largest proportion of composites of any other bacterial genome studied (22%). In general, the Archaea appear to contribute the fewest composite proteins in the analysis, even in relation to their small genome sizes, although this may be due to the fact that there were only 17 Archaeal genomes available (see Additional File 1).

As expected, a similar pattern emerges between the number of unique components versus composites (see Additional File 2a), irrespective of genome size. The distribution of the fraction of proteins involved in predicted interactions for the three major taxonomic domains illustrates the major differences between each domain (see Additional File 2b). For Archaea and Bacteria, the distribution is unimodal with a median between 12–14% of proteins involved in interactions for Archaea and 16–18% for Bacteria. The distribution for Eukaryota is manifestly bimodal with many genomes at 14–16% and a separate set of outliers at 30–42%. Inspection of the latter set indicates that it represents multicellular species, while the set with the smaller median value represents unicellular species. All of the above percentages are markedly higher than those obtained in a previous analysis involving 24 genomes [3], where on average 9% of proteins of the total encoded in a genome were found to correspond to unique components involved in gene fusion.

### Cumulative effect of multiple genomes

In order to assess the effect of an increasing number of genomes for fusion analysis, we analyzed the number of detected individual interactions for each genome based on fused genes detected in each of the reference genomes. The reference genomes are displayed in terms of the number of interactions they may contribute for each query genome. For those genomes with good correlation coefficients between the relative rank order of reference species and number of contributed interactions (> 0.9), these data are virtually linear (for an example see Figure 3c), with 115 such genomes having slopes greater than 1.0 (average slope 4.75, see Additional File 3). These data indicate that there does not appear to be a saturation effect and increasing numbers of genomes might still give rise to novel protein interaction predictions (see Additional Files 4 &5; for example, Figure 3c corresponds to page 114 of Additional File 5). In particular, certain reference genomes generate a large number of novel interactions, e.g. eukaryotic genomes, typically in a non-monotonic fashion. This indicates that detection of protein interactions based on gene fusion will continue to be a powerful tool for genome analysis.

A small number of genomes did appear to exhibit saturation effects, where new reference genomes did not significantly boost the number of initially obtained interactions (see Additional Files 4 &5). One striking case being that of the *Wolbachia pipientis *genome (slope 0.63; see page 159 of Additional File 5). This result is not surprising as *W. pipientis *is an endosymbiont of insect species and possesses a minimal genome necessary for transmission and survival [17]. In fact, many other species with similar slopes also possess small genomes and/or are endosymbionts and obligate intracellular parasites e.g. *Nanoarchaeum equitans *(a hyperthermophilic obligate symbiont [18]; slope 0.05), *Mycoplasma genitalium *(slope 0.16), and a number of *Chlamydia *strains such as *Chlamydia trachomatis *(MoPn) (slope 0.16) – (for all these cases, see Additional Files 4 &5).

Another such example is the extremophile *Methanopyrus kandleri *(slope 1.05) which is a rod-shaped Gram-positive methanogen that grows chemolithoautotrophically at 80–110°C in the H_2_CO_2 _atmosphere of a deep water geothermal chimney [19]. These results appear to indicate that it might be more difficult to detect protein interactions for genomes from poorly represented taxonomic classes, i.e. most gene fusion events are detected between large groups of related species. This result is borne out when one examines the genomes with the largest slopes. The top ten such genomes are all Proteobacteria, Actinobacteria or Firmicutes, classes of bacteria highly over-represented in COGENT (119 genomes or 65%).

### Biological diversity of gene fusion events

**Figure 4:**
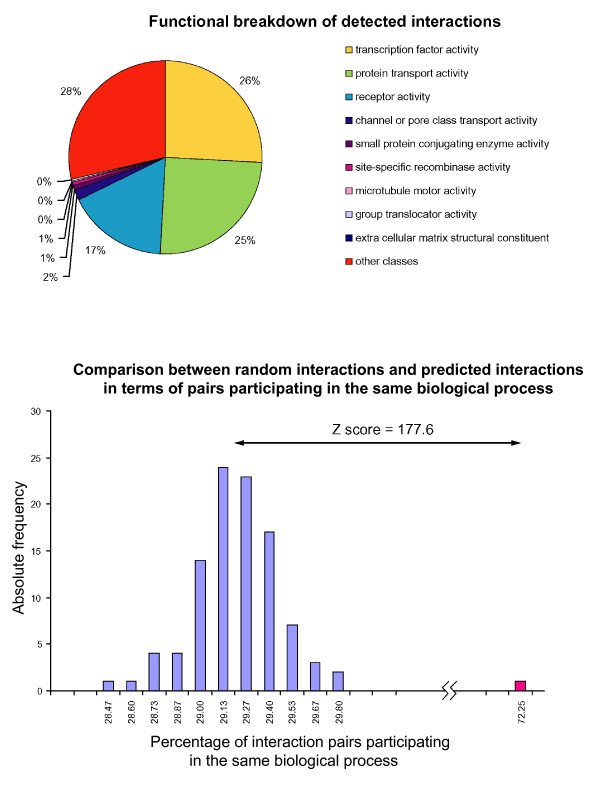
Functional classification of predicted interactions. **a) **Functional classifications of predicted interactions based on Gene Ontology (GO) assignments for Molecular Function section of GO. **b) **Distribution of percentages of interacting pairs belonging to the same GO class for both randomly picked pairs (blue) and for real pairs (red).

In order to delineate the functional diversity of proteins predicted to interact by virtue of gene fusion events, we infer functional classes for each component of a fusion event (where possible), based on GO [13] annotations derived from their domains (see **Methods**). A number of functional classes relating to both the 'biological process' and 'molecular function' ontologies appear to be highly over-represented in components of gene fusion events (Table 1). The most highly over-represented molecular function classes, overall, are for receptors and protein transporters, both occurring more than ten times than expected (Table 1 & Figure 4). Small protein conjugating enzymes are the third most over-represented class, occurring eight times more than expected. These three classes represent excellent targets for gene fusion, as they involve proteins that either form structural complexes or facilitate the channeling of various small-molecule and macromolecular substrates [1,20]. Transcription factors, and pore/channel transporters are also highly over-represented (by two fold) (Table 1).

**Table 1:** Over-represented GO classes from the *'Molecular Function' *and *'Biological Process' *hierarchies observed among genes involved in fusion events

**GO Molecular Function Class**	**Number of domains found**	**Domains with GO Annotation**	**Percentage in fusion events**	**Percentage in all domains**	**Log Odds Expectation**
receptor activity	122299	720242	0.170	0.017	**3.349**
protein transporter activity	180926	720242	0.251	0.029	**3.120**
small protein conjugating activity	5566	720242	0.008	0.001	**2.798**
microtubule motor activity	2780	720242	0.004	0.001	**1.797**
channel or pore class transporter activity	11394	720242	0.016	0.009	**0.832**
transcription factor activity	185303	720242	0.260	0.161	**0.675**
site-specific recombinase activity	3732	720242	0.005	0.003	**0.636**
extracellular matrix structural constituent	1242	720242	0.002	0.001	**0.634**
group translocator activity	1742	720242	0.002	0.002	**0.122**
other classes	205258	720242	0.285		

**GO Biological Process Class**					

cell communication	25613	416772	0.061	0.010	**2.674**
regulation of cellular process	48082	416772	0.115	0.055	**1.068**
regulation of development	4082	416772	0.010	0.007	**0.510**
cellular physiological process	34681	416772	0.083	0.059	**0.493**
regulation of physiological process	205318	416772	0.493	0.356	**0.468**
other classes	98996	416772	0.238		

We compared this breakdown of functional classes for gene-fusion based predictions of interaction against a set of experimentally determined interacting proteins from the DIP database [21], to determine whether the detected classes are representative of interacting proteins in general, or only those involved in gene fusion events (Figure 4a). The breakdown of GO classes for DIP interactions is significantly different than those obtained by gene fusion analysis (not shown), consistent with previous findings that certain classes of genes are more likely to be involved in fusion events, previously shown in certain cases [14,15], Thus, these sets of interactions appear to contain functionally diverse sets of proteins.

Finally, we evaluate the performance of gene fusion based prediction of protein-protein interaction and functional association, using these derived functional classes. We expect that if the method performs accurately, then predicted interactions should, in most cases, link proteins of the same or similar functional class [9]. In order to test this hypothesis, we calculated the number of interactions for which both sets of proteins had matching functional classes (see **Methods**). We then generate 100 randomized trials and performed the same evaluation (see **Methods**). When the distribution of matches is compared for the real and randomized data, we find that the observed pairs are significantly more likely to link genes of the same functional class, than randomized pairs (Z-score 177.6) with 72.25% of real interactions having matching classes, compared to an average of 29.2% for the randomized trials (Figure 4b).

### Examples of protein interactions for validated annotations in Chlamydia

To further exemplify the validity of our predictions, we validated all detected pairs of interacting proteins against a high-quality, manually curated set of functional annotations from the genome of *Chlamydia trachomatis *serovar D [22]. This set consists of 40 pairs (10 additional pairs of adjacent open reading frames are detected but excluded from this analysis, due to possible artefacts in gene prediction). Of the 40 instances, 4 (10%) are well-known interactions (5, 6, 13, 37), evident from the associated annotations (see Additional File 6, known). Another 4 (10%) instances (12, 29, 30, 31) are probably false, due to paralogy between one of the proteins and the genuine interacting partner (see Additional File 6, paralog). There are 10 (25%) instances (2, 4, 17, 19, 21, 22, 25, 34, 35, 40) where at least one of the partners is not functionally characterized (see Additional File 6, unknown). In cases where one protein is characterized, this analysis provides an indication for the association of the other partner in a similar process (e.g. CT379 associates with cytidylate kinase, CT452). The remaining 22 instances (55%) represent interactions, ranging from the highly likely, supported by annotation (e.g. 7–9, 14: AroA/B or 15: AroA/E), to reasonable predictions (e.g. 11: a tRNA modification enzyme with riboflavin kinase, 16: GPI with transaldolase [23]), to highly surprising (e.g. 18: DnaK with Ser/Thr protein kinase) (see Additional File 6, predicted).

### A network of protein interactions in the model plant species Arabidopsis

**Figure 5:**
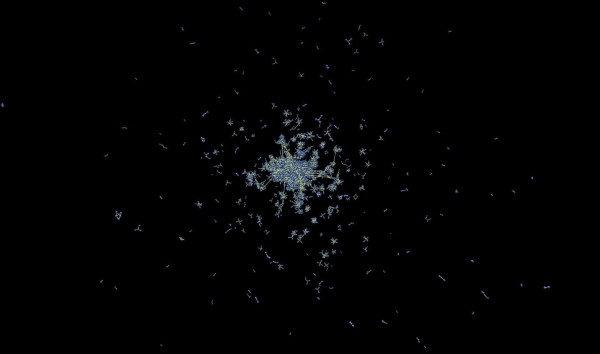
A representation of predicted protein interactions in *Arabidopsis thaliana*, using BioLayout [34]. Nodes represent proteins, and links represent interactions.

Finally, a question that typically arises in this type of analysis relates to the efficacy of this method for the delineation of protein interactions in eukaryotic species. In fact, a large part of the predicted interactions occurs in a relatively small group of eukaryotes (Figure 3a). For instance, about a fifth of the total number of detected interactions occur in *Arabidopsis thaliana*. The analysis of this network is of particular interest, since there have not been yet any high-throughput protein interaction detection experiments for this important model plant species. In total, we are able to predict 409,217 interactions in *A. thaliana*, encompassing 9,834 unique proteins and forming 312 distinct network components. The structure of the *A. thaliana *interaction network is representative of those found in other eukaryotes, with a giant component dominating the picture, and smaller peripheral interaction clusters (Figure 5). An analysis of such network presents a challenge, for two main reasons: first, complex eukaryotic species undergo various developmental stages, thus any protein complement analysis has to take into account sub-cellular, cell type, tissue, organ and developmental stage co-ordinates, something that is certainly not always possible; second, the paralogous nature of eukaryotic genomes obscures potential protein interactions, usually by over-predicting possible, not necessarily false, functional associations (as one-to-many and many-to-many relationships) (Figure 5).

In order to perform an analysis of the functional network in this model plant species, we focus on the functional significance of one-to-one interactions only, i.e. these interactions where the two members are found to interact with each other, on the basis of a composite protein elsewhere. Our survey reveals a few examples of well-annotated interactions, as well as predictions for novel cases of interacting proteins. Known cases include a multitude of self-evident protein pairs, as assessed by the corresponding gene descriptions. This fact provides further reassurance for the relevance of this approach. Such examples include the detection of interactions between components of the NADH-ubiquinone oxidoreductase complex, components of the L-arginine biosynthesis pathway, and components of the glycerophospholipid metabolism pathway (see Additional File 7). Less evident examples include interactions between proteins that are less well characterized, with partial annotations that can be used to shed a light on the biological process in which these proteins participate. One such case is the interaction between At4g10100 (accession: Q9S7A3) (molybdopterin synthase small subunit) and At2g43760 (O22827) (putative molybdopterin synthase large subunit), which provides further reassurance that At2g43760 is indeed a component of the molybdopterin synthase complex (see Additional File 7). Another less clear case is the interaction between At2g32600 (O80897) (putative spliceosome associated protein) and At5g46840 (Q9LUK6) (similarity to RNA-binding protein), which suggests that the latter protein might also be involved in spliceosome activity (see Additional File 7). A particularly striking example is the interaction between At1g79150 (Q1KS85) and At1g79140 (Q6NME0), both hypothetical proteins: the former containing a NOC3p domain [24] and the latter containing a CBF domain, a domain found in the yeast NOC1 protein [25] (Q12176) (see Additional File 7). In yeast, Noc1p and Noc3p are required for ribosome maturation and transport, where mutation of each Noc protein impairs intranuclear transport of 60S subunits at different stages and inhibits pre-rRNA processing [26]. Since homologs for each of the Noc1-3p proteins exist in higher eukaryotes, it is likely that their functions in ribosome synthesis are conserved in evolution [26]. The inferred interaction between the two *A. thaliana *proteins provides some preliminary evidence for the existence of an homologous complex and molecular process in *A. thaliana*.

## Conclusion

We present the largest analysis, as yet undertaken, toward the computational detection of protein-protein interactions and functional associations based on gene fusion events. We illustrate that gene fusion events are widespread across the different domains of life and are present in intricate patterns across various genomes, both in terms of their phylogenetic distribution and in terms of the biological diversity of proteins involved in these events. The fact that we have by no means saturated the predictive power of this approach with 184 genomes is encouraging, since previous research has already shown that the fidelity of these predictions increases as more genomes are considered. Unlike other genome context methods, such as phylogenetic profiles which perform best with non-eukaryotic genomes [27], the gene fusion approach benefits from the multi-domain nature of eukaryotic proteins for the prediction of protein interactions, if properly implemented. As more genomes become available, particularly for taxonomic groups that are currently not well represented, we expect that gene fusion analysis will continue to be a valuable tool for the rigorous exploration of protein function in entire genome sequences [28].

## Methods

### Genome Analysis

Sequence data was obtained from the COGENT database [12] (v190) for 696,894 protein sequences from 184 complete genomes. Sequence similarities for all proteins against each other was generated using NCBI BLASTp v2.0 [29] with an E-value threshold of E ≤ 1e^-10^. Two proteins will be considered as interacting partners of a gene fusion event if both were similar to a non-overlapping part of a third sequence and were not similar to each other. To check similarity between the interacting proteins, we used a Smith-Waterman dynamic programming alignment tool [30,31] (prss33) with a threshold p-value of 0.04.

### Promiscuous Domain Filtering

We calculated the domain architecture of all protein sequences using HMMER [32] against the PFAM (v15) database [11] as a reference, using an e-value threshold of E ≤ 1e^-10^. A frequency analysis was then performed on the connectivity of PFAM domains. Recently, an analysis similar in spirit but different in detail has also been published [33]. The distribution obtained is logarithmic, with a long tail. We assume that this tail indicates the presence of promiscuous domains. When these data are visualized as a network, using the BioLayout tool [34], most of the nodes (i.e. proteins) are connected (i.e. interacting) in a super-cluster, due to the presence of promiscuous domains. We use this domain connectivity graph in order to determine a threshold for the removal of promiscuous domains (Figure 2). The ideal threshold should remove promiscuous domains but leave intact all the other domains. The size of the super-cluster (largest connected component) was plotted against the threshold and we look for a threshold where this super-cluster is drastically reduced while still retaining most of the data (Figure 2). From this graph, we choose a threshold C of 8 connections (removing 10% of the data) as a reasonable compromise. At this point the super-cluster is reduced to 94 proteins from the initial 1611 whereas 90% of all nodes are retained. Interestingly, the median length of domains with connectivities of C = 8 is 153 residues, while the median length of domains with connectivities of C>8 is 117 residues (Figure 2c).

### Functional class annotation of interactions

GO annotations [13], for each PFAM domain, are obtained from the InterPro database [35]. To build functional classes from GO terms, we flatten the GO hierarchies for both molecular function and biological process to three levels below the tree root, and map all GO terms up to this level. For each predicted interaction, we then create a list of PFAM domains involved. Then for each domain we find the corresponding GO terms. These terms are applied to the corresponding COGENT entries. Thus, for each predicted interaction we generate a list of GO annotations for both interacting partners.

### Randomized Trial

We also examine every interaction to assess whether both proteins have at least one common GO annotation (in terms of biological process) and if this is the case, we count the interaction as a match, and if not, we count it as a mismatch. The total number of matches was 13,841 and the number of mismatches was 5,316. To find whether this result is significant or random, we randomized the interactions in terms of GO annotations and compared the results from the real and shuffled data.

### Data availability

The interaction results from this study are available as Additional File 8.

## Authors' contributions

A. Kamburov, LG, VK and AJE participated in the study design and performed the data analysis. SF, A. Kapazoglou, AT and CAO performed the data validation and annotation case studies. AJE and CAO coordinated the study and drafted the manuscript. All authors read and approved the final manuscript.

## Supplementary Material

Additional File 1 Dependence of fraction of composite (fused) genes on genome size. The x-axis represents genome size (number of genes) for a given query species, the y-axis represents the fraction of composite genes in that species. Color coding as in Figure 3. Click here for file

Additional File 2 a) Dependence of fraction of composite (fused) genes on fraction of component (un-fused) genes, as a ratio of unique instances over total number of genes, per genome. b) Distribution of fraction of distinct interacting proteins in the three domains of life (color coding as in Figure 3) and all domains (grey). Click here for file

Additional File 3 Distribution of slopes (relative rank order of reference species and number of contributed interactions) across genomes (see Figure 3c for an example). a) Distribution of slope values for all species; 25 species are excluded with slopes>12. b) Plot of slopes versus genome size. The x-axis represents the slope for a given species, the y-axis represents genome size (total number of genes) for that species. Both axes are shown on a logarithmic scale. Grey boxes represent the 25 species with slopes>12. Click here for file

Additional File 4 Distribution of composites across 184 genomes. Click here for file

Additional File 5 Distribution of interactions across 184 genomes. Click here for file

Additional File 6 Analysis of predicted interactions from *Chlamydia trachomatis*. Examples of previously characterised interactions in *C. trachomatis *that are detected by gene fusion analysis. Cases in bold are presumed as known; cases in purple are artifacts from genomic paralogy. Click here for file

Additional File 7 Examples of predicted interactions from *Arabidopsis thaliana*. The species of origin is also given for the composite proteins. Click here for file

Additional File 8 Inferred interactions in this study. The data forms part of the COGENT++ data environment.[36]. Click here for file
