# Supplementary figures and images for: Denoising inferred functional association networks obtained by gene fusion analysis
Source: BMC Genomics. 2007 Dec 14;8:460. doi: 10.1186/1471-2164-8-460 (PMC2248599; doi:10.1186/1471-2164-8-460)

Number of genes VS fraction of composite genes

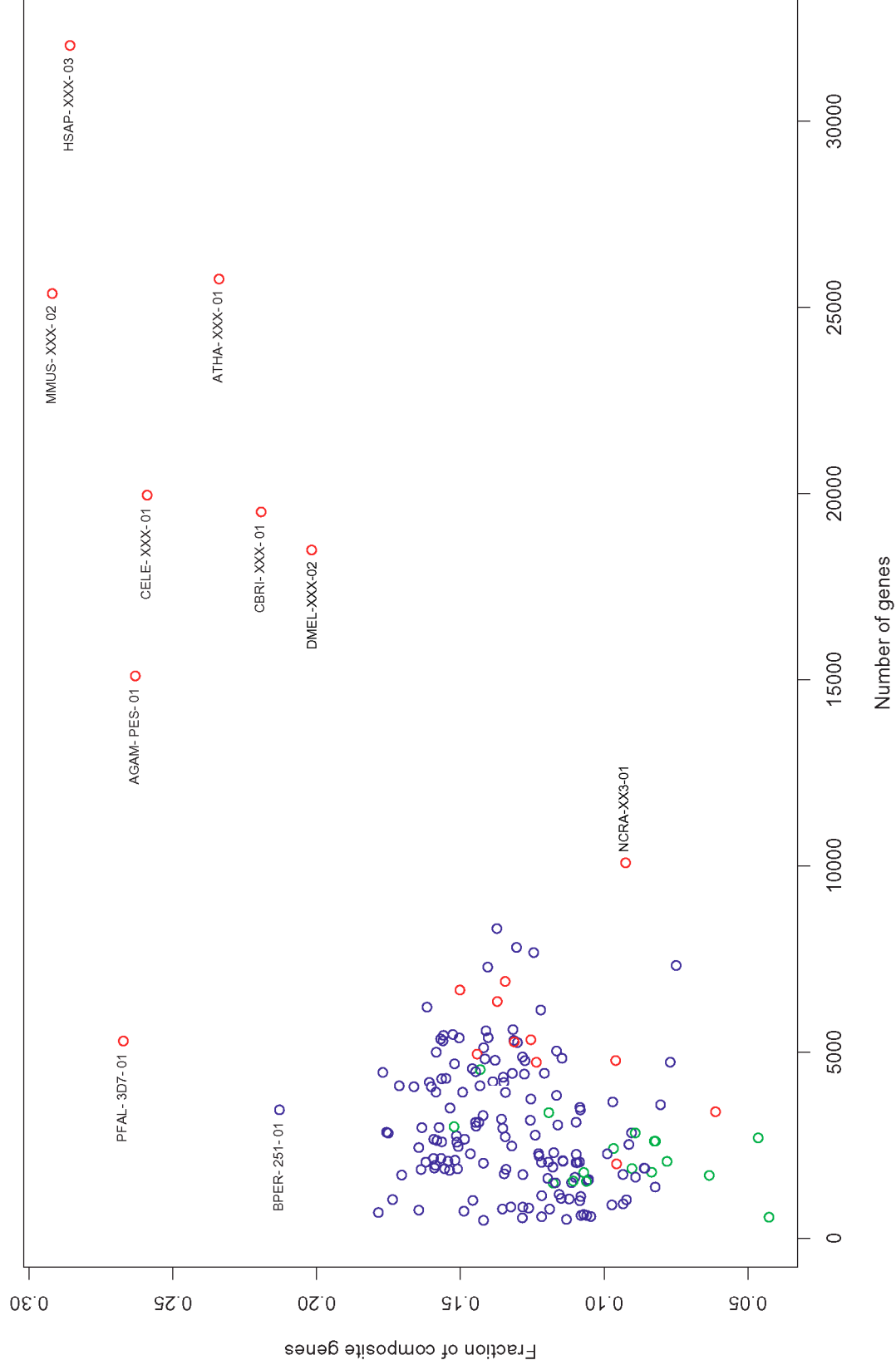

Supplement: Additional File 1 — Dependence of fraction of composite (fused) genes on genome size. The x-axis represents genome size (number of genes) for a given query species, the y-axis represents the fraction of composite genes in that species. Color coding as in Figure 3. [file 1471-2164-8-460-S1.pdf]

a)

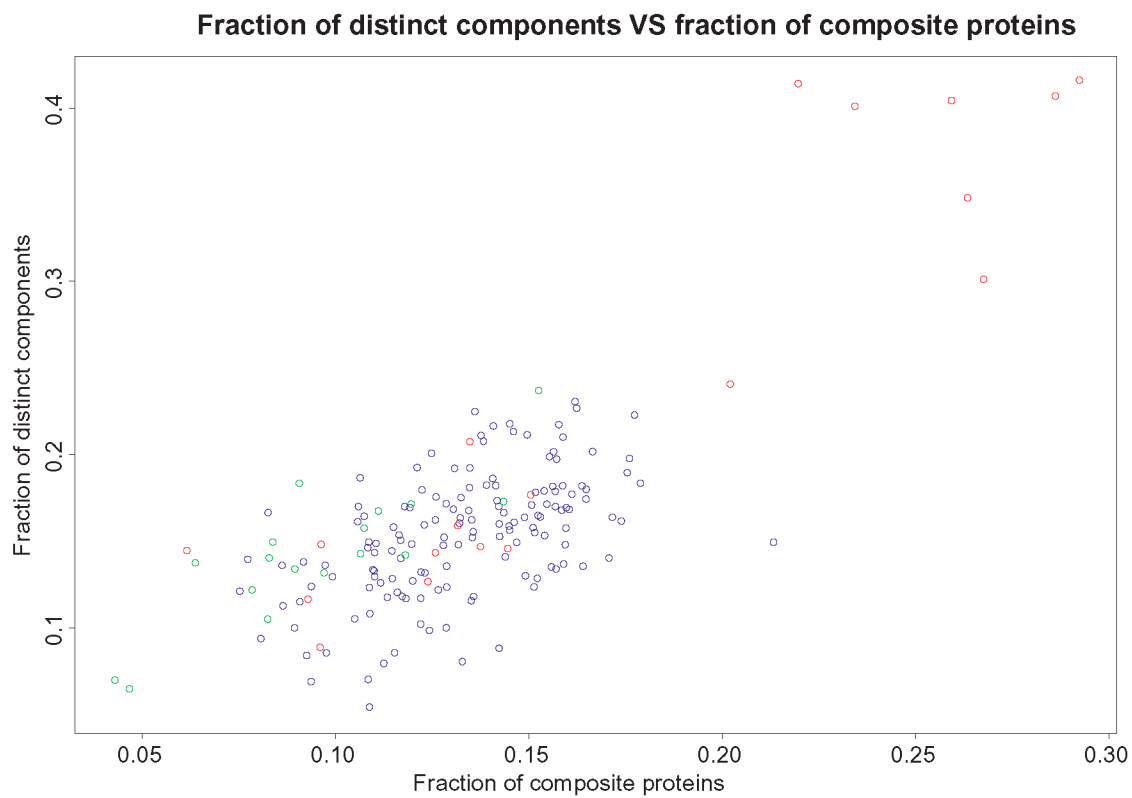

b)

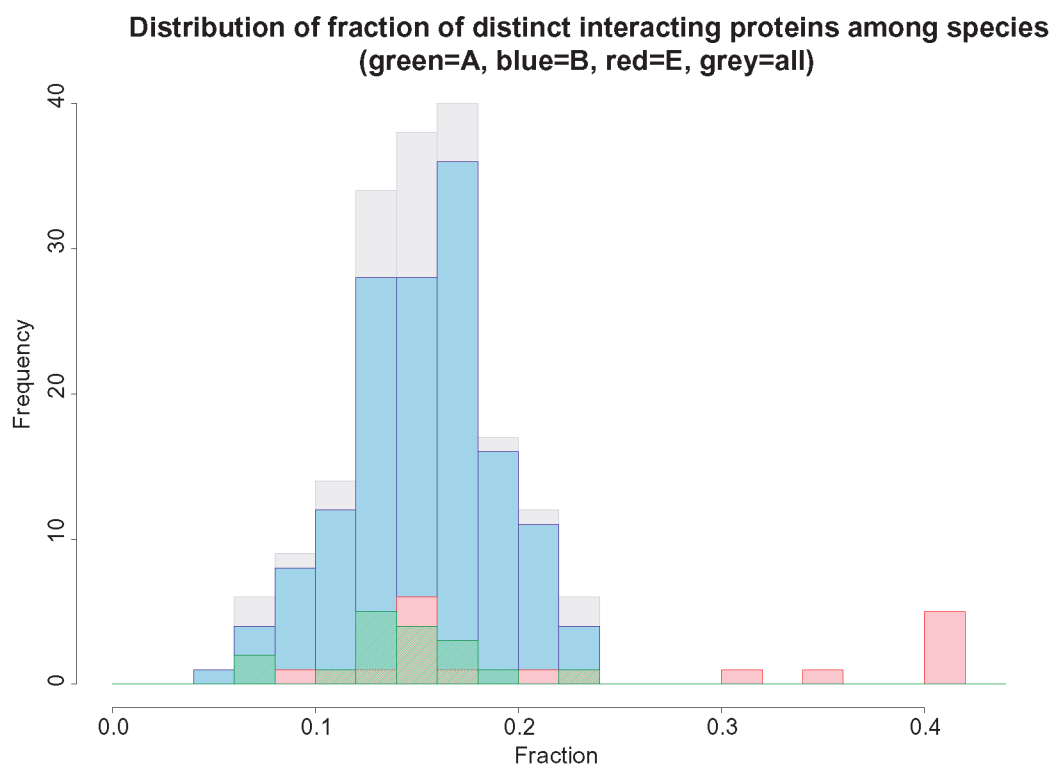

Supplement: Additional File 2 — a) Dependence of fraction of composite (fused) genes on fraction of component (un-fused) genes, as a ratio of unique instances over total number of genes, per genome. b) Distribution of fraction of distinct interacting proteins in the three domains of life (color coding as in Figure 3) and all domains (grey). [file 1471-2164-8-460-S2.pdf]

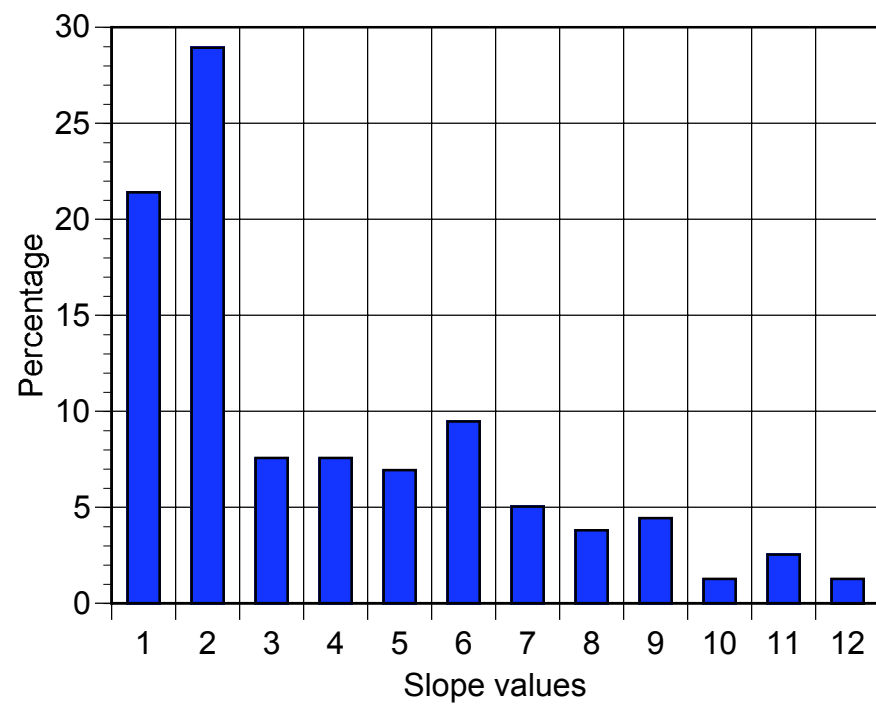

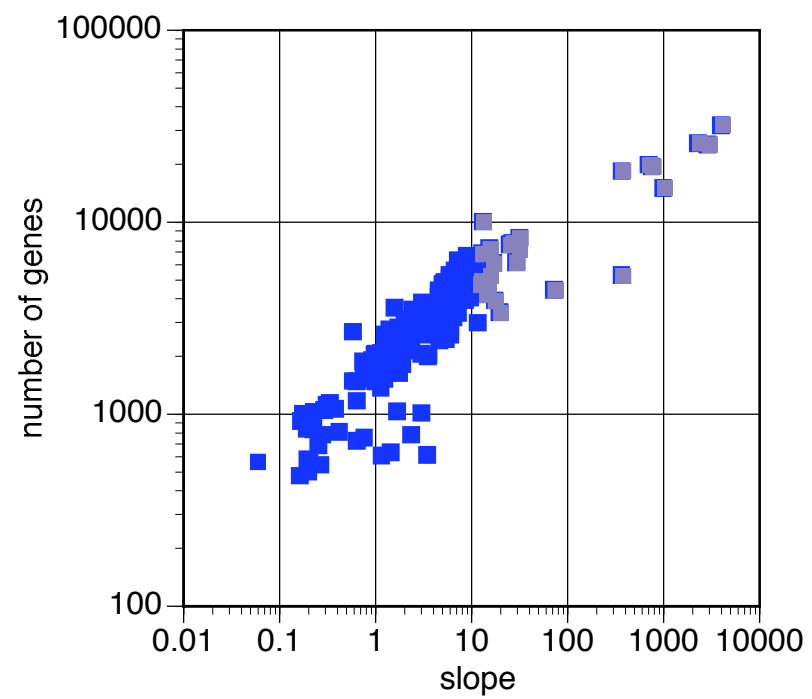

Supplement: Additional File 3 — Distribution of slopes (relative rank order of reference species and number of contributed interactions) across genomes (see Figure 3c for an example). a) Distribution of slope values for all species; 25 species are excluded with slopes>12. b) Plot of slopes versus genome size. The x-axis represents the slope for a given species, the y-axis represents genome size (total number of genes) for that species. Both axes are shown on a logarithmic scale. Grey boxes represent the 25 species with slopes>12. [file 1471-2164-8-460-S3.pdf]
