# Supplementary material for: Denoising inferred functional association networks obtained by gene fusion analysis
Source: BMC Genomics. 2007 Dec 14;8:460. doi: 10.1186/1471-2164-8-460 (PMC2248599; doi:10.1186/1471-2164-8-460)
Supplement: Additional File 4 — Distribution of composites across 184 genomes. [file 1471-2164-8-460-S4.pdf]

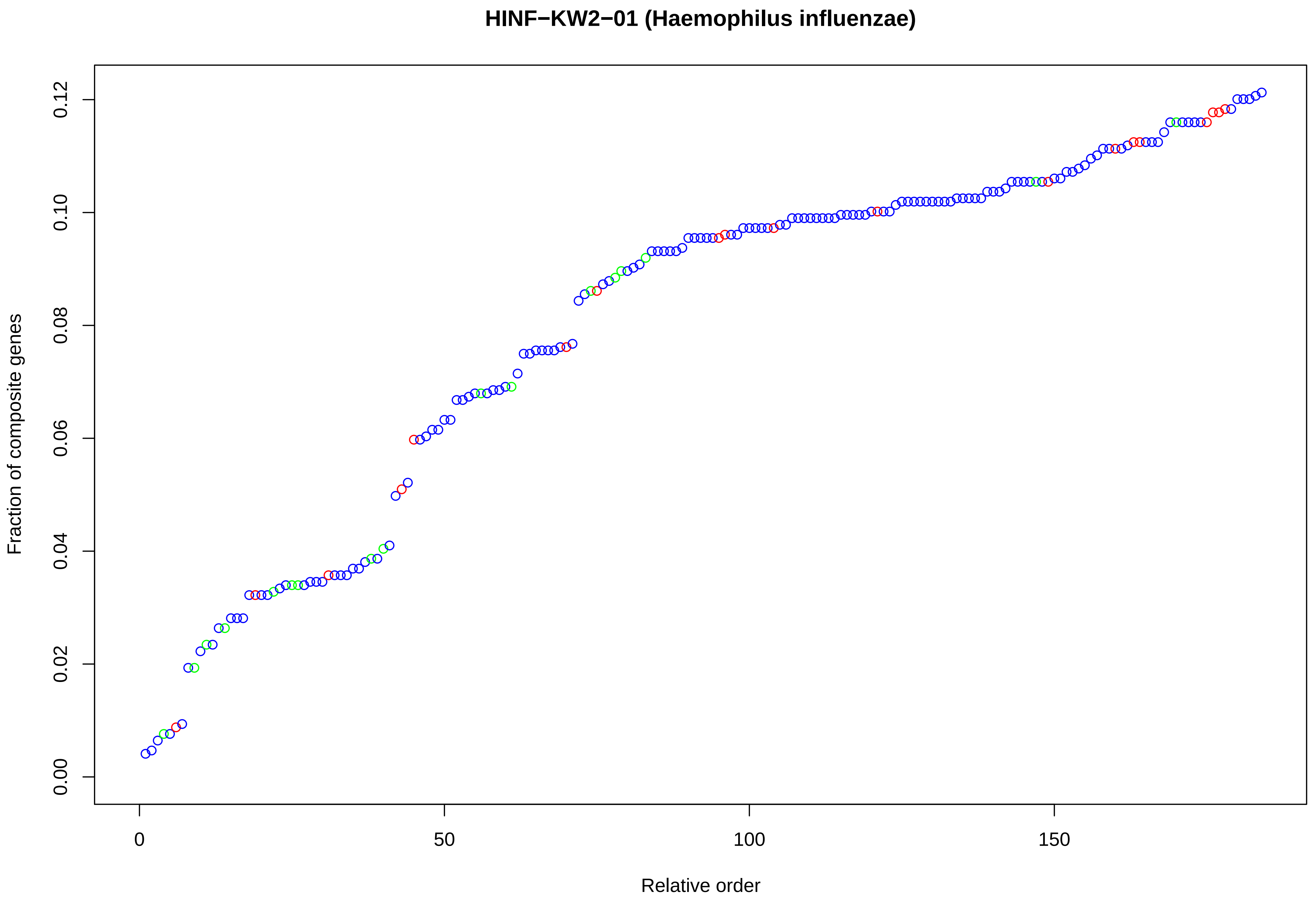
HINF−KW2−01 (Haemophilus influenzae)
Fraction of composite genes
Relative order
0.00
0.02
0.04
0.06
0.08
0.10
0.12
0
50
100
150

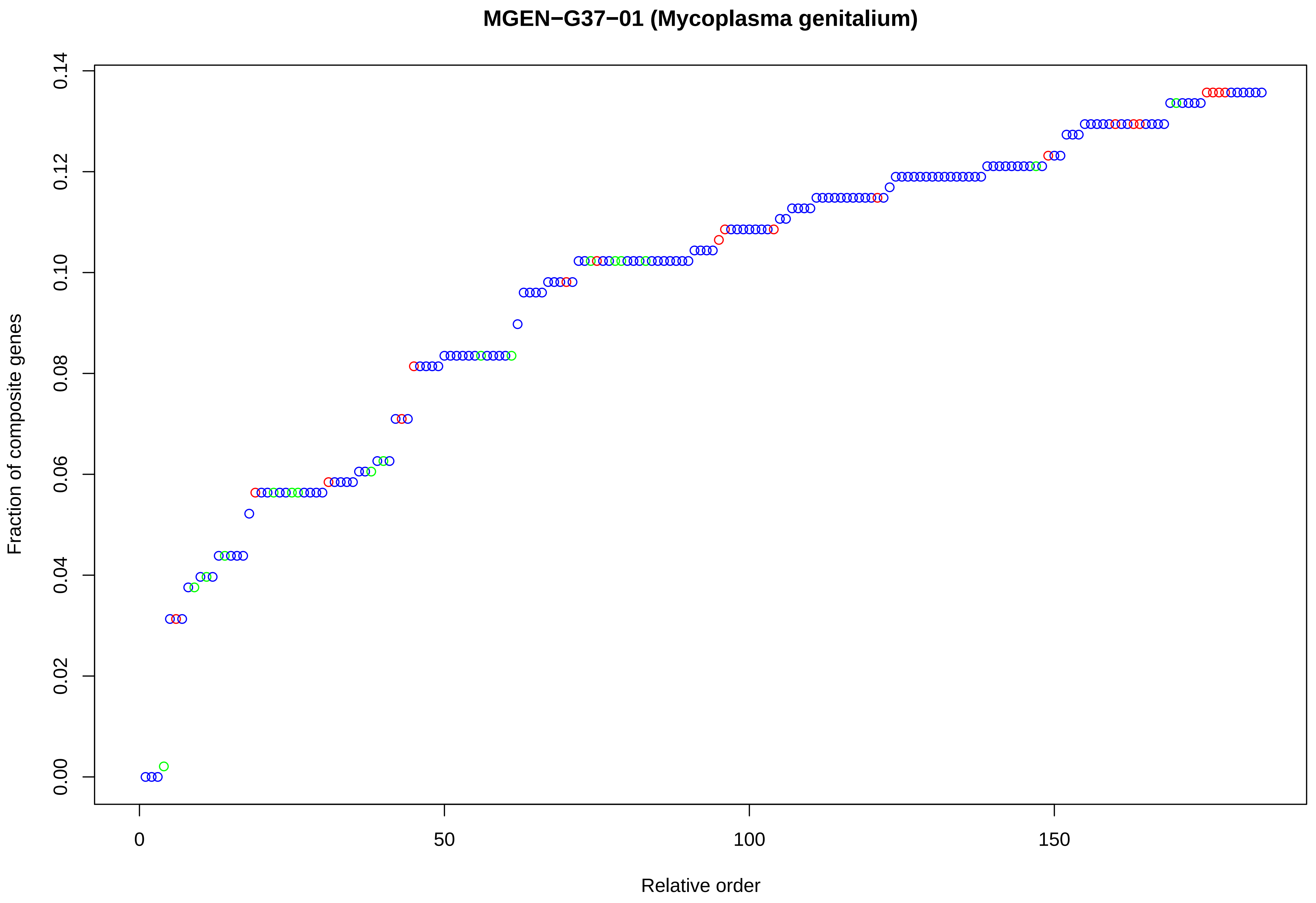
MGEN−G37−01 (Mycoplasma genitalium)
Fraction of composite genes
0.00
0.02
0.04
0.06
0.08
0.10
0.12
0.14
Relative order
0
50
100
150

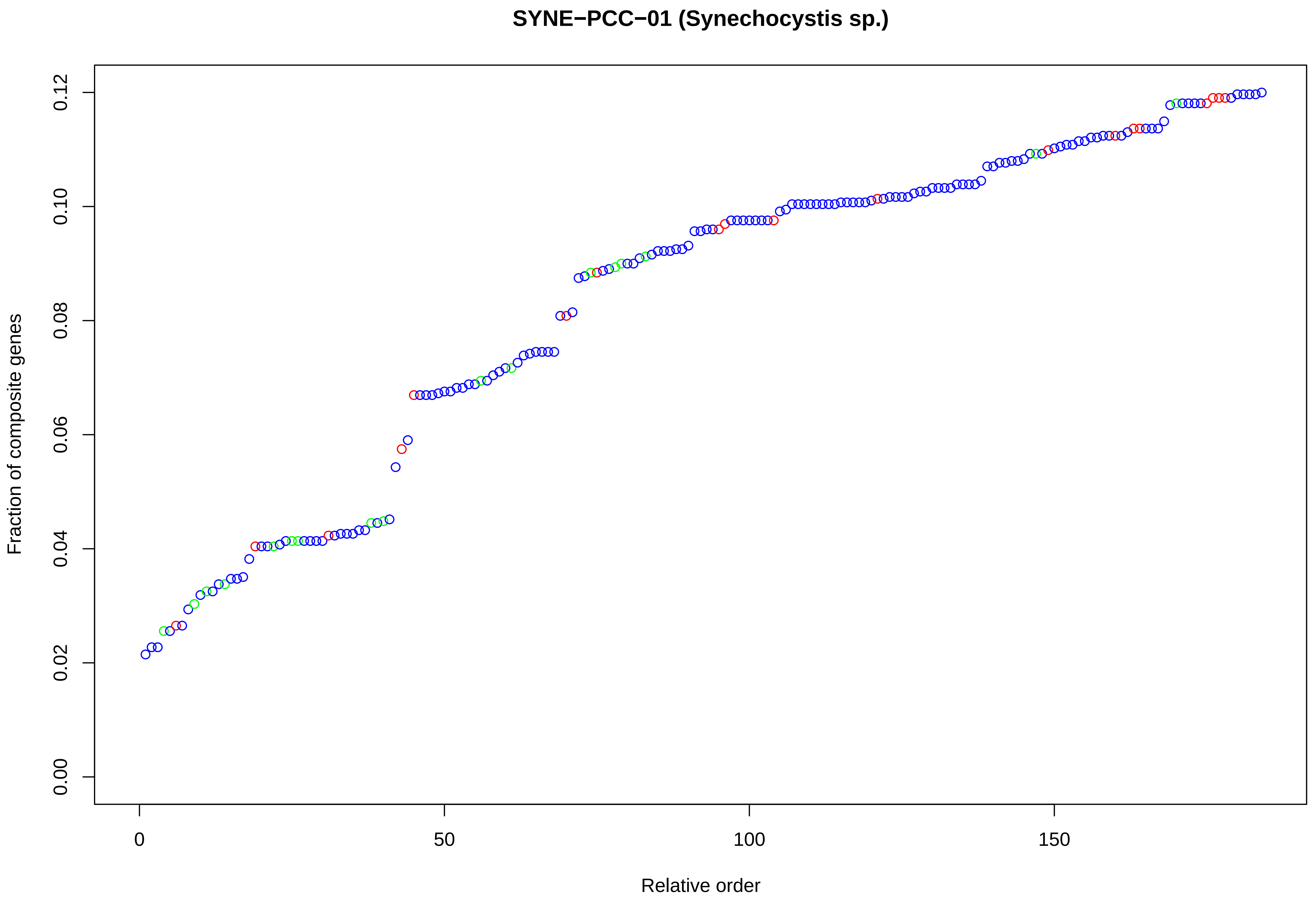

SYNE−PCC−01 (Synechocystis sp.)
Fraction of composite genes
0.00
0.02
0.04
0.06
0.08
0.10
0.12
0
50
100
150
Relative order

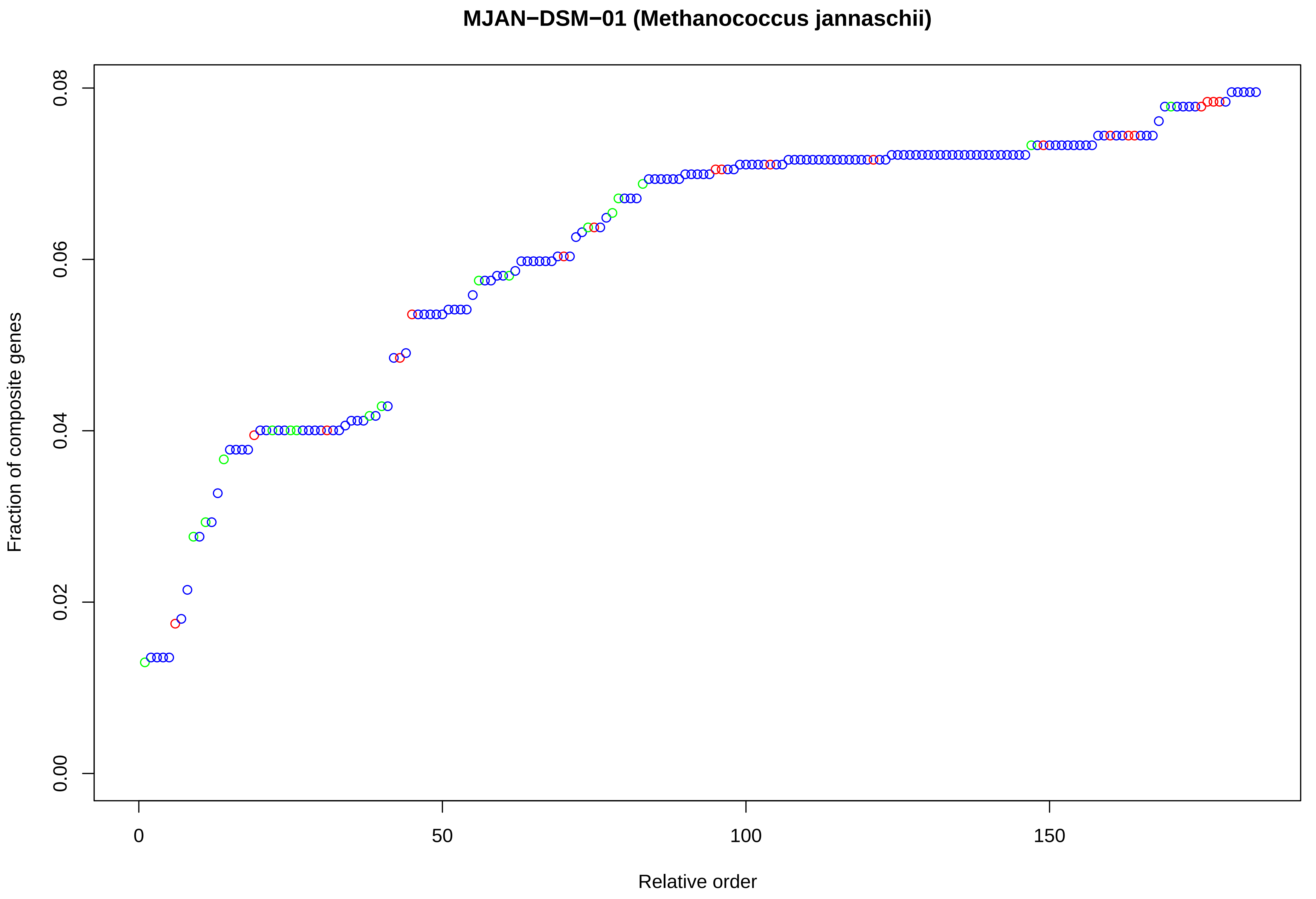
MJAN−DSM−01 (Methanococcus jannaschii)
Fraction of composite genes
0.00
0.02
0.04
0.06
0.08
Relative order
0
50
100
150

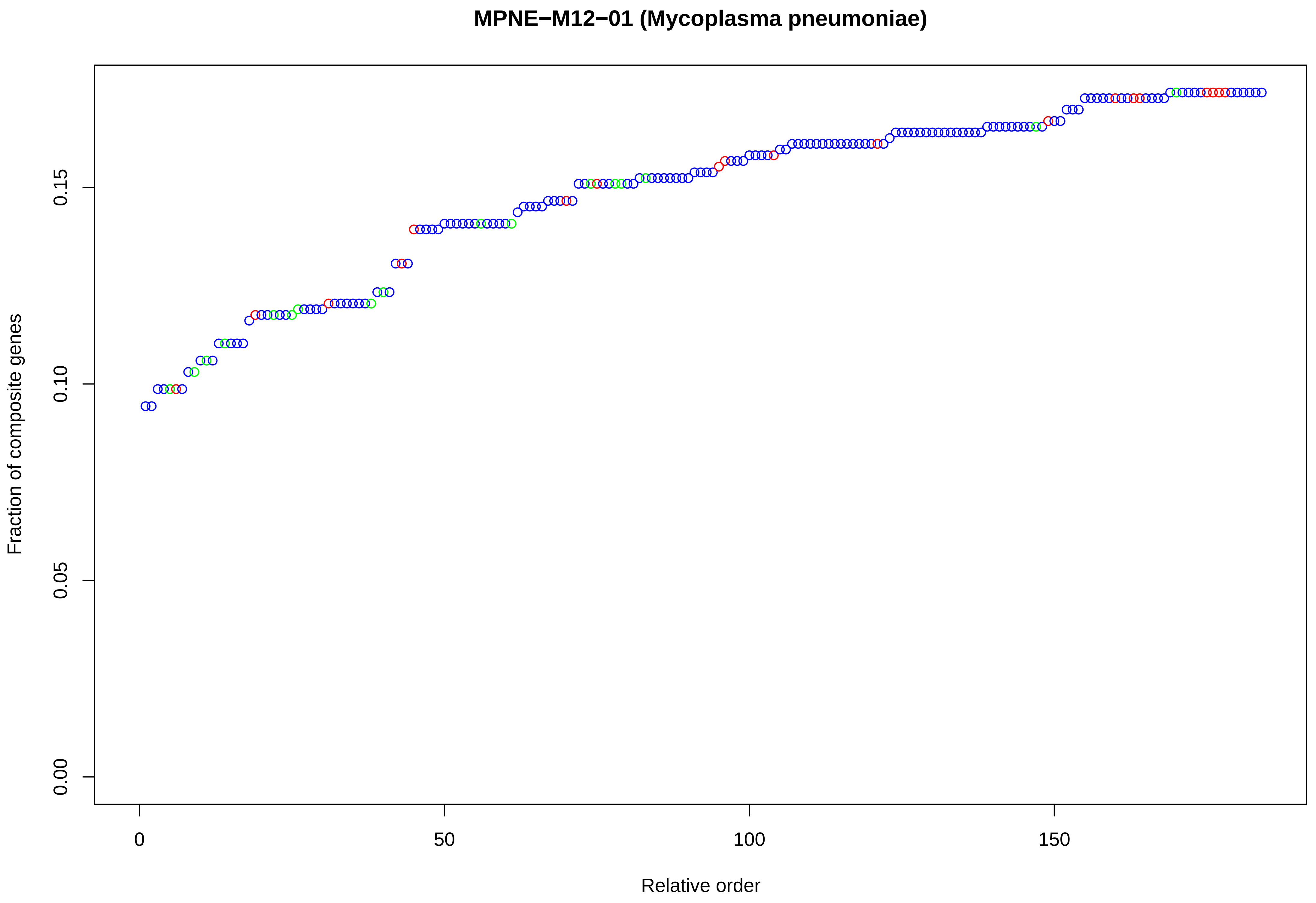
MPNE−M12−01 (Mycoplasma pneumoniae)
Fraction of composite genes
0.00
0.05
0.10
0.15
Relative order
0
50
100
150

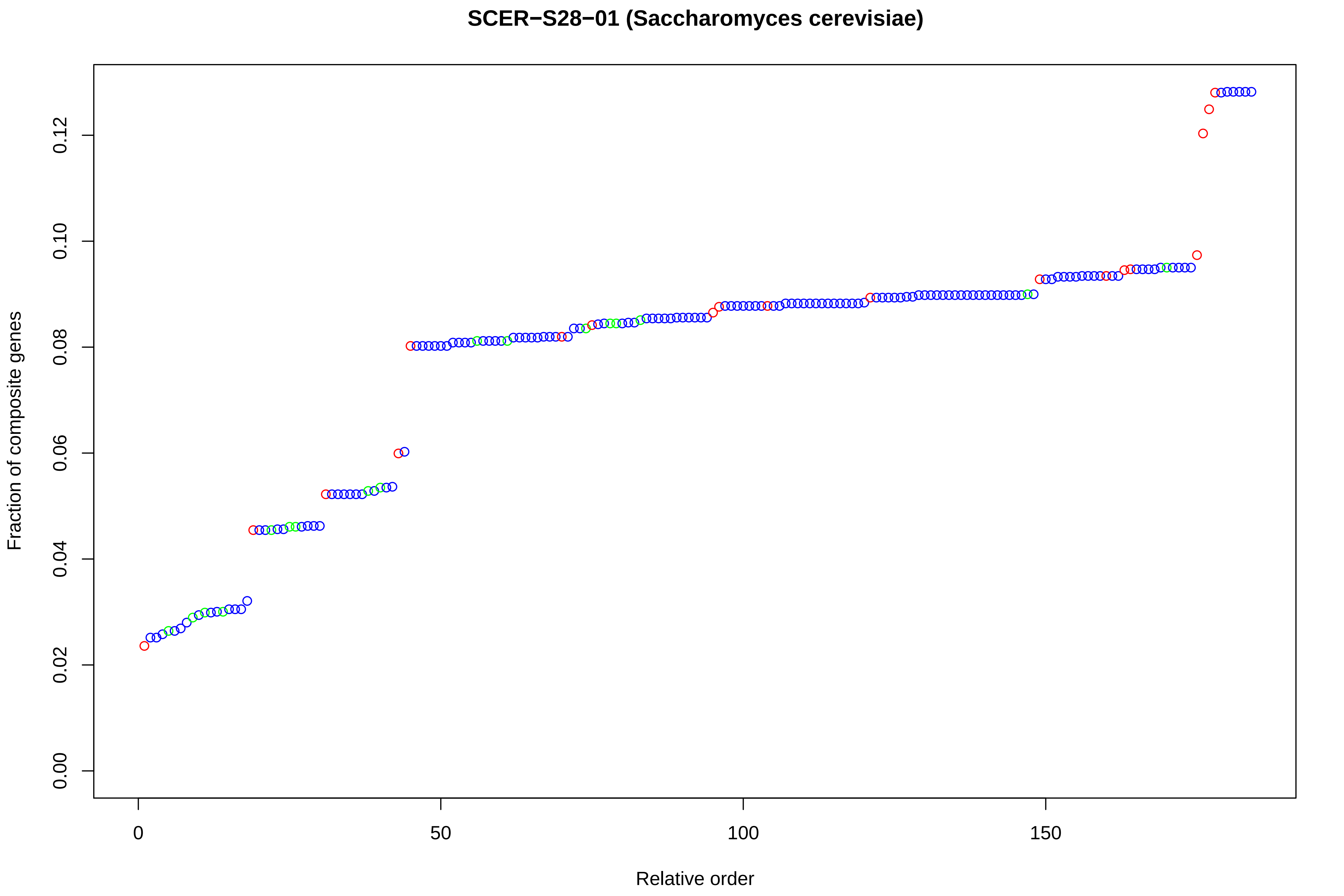

SCER−S28−01 (Saccharomyces cerevisiae)
Fraction of composite genes
0.00
0.02
0.04
0.06
0.08
0.10
0.12
0
50
100
150
Relative order

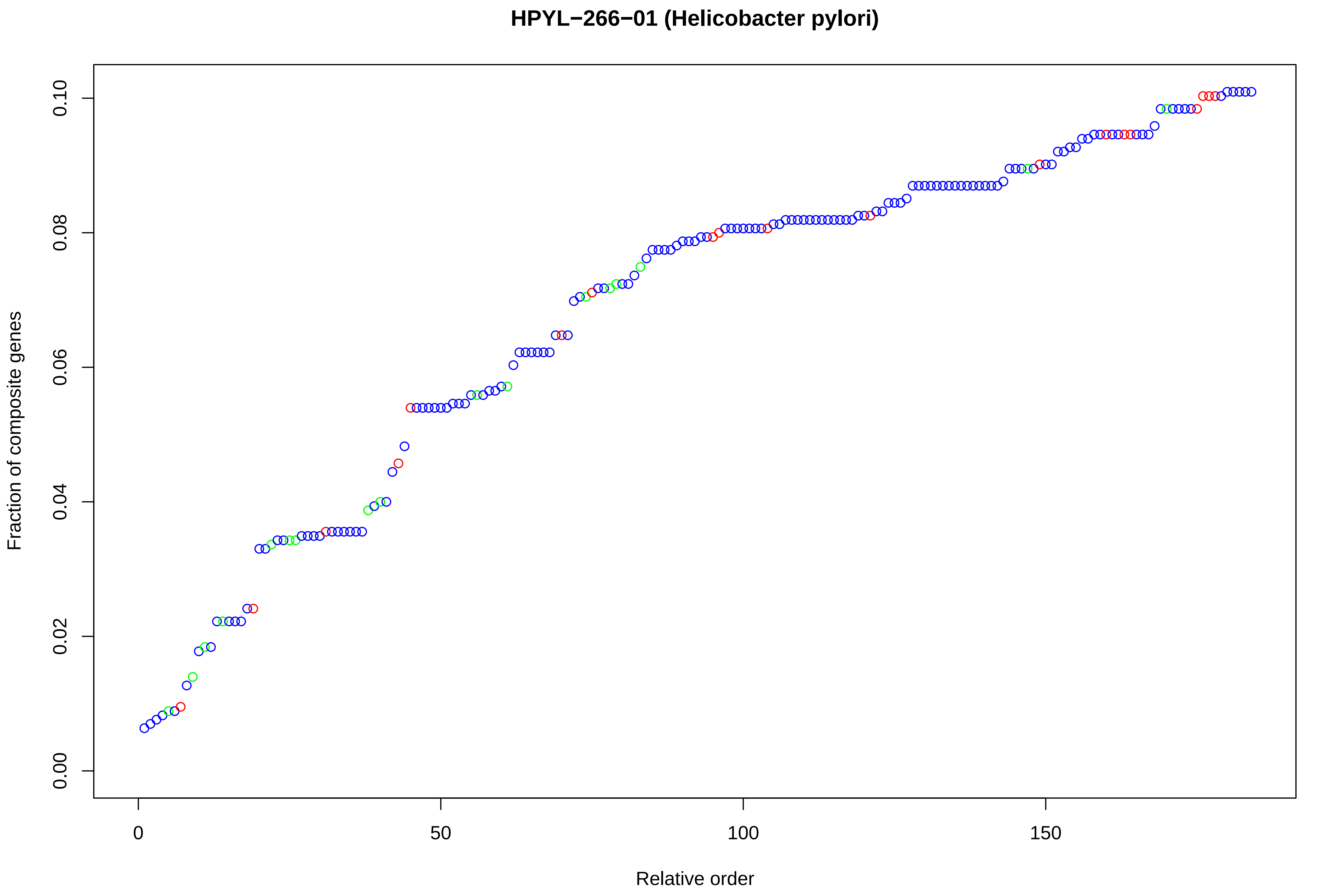
HPYL−266−01 (Helicobacter pylori)
Fraction of composite genes
0.00
0.02
0.04
0.06
0.08
0.10
0
50
100
150
Relative order

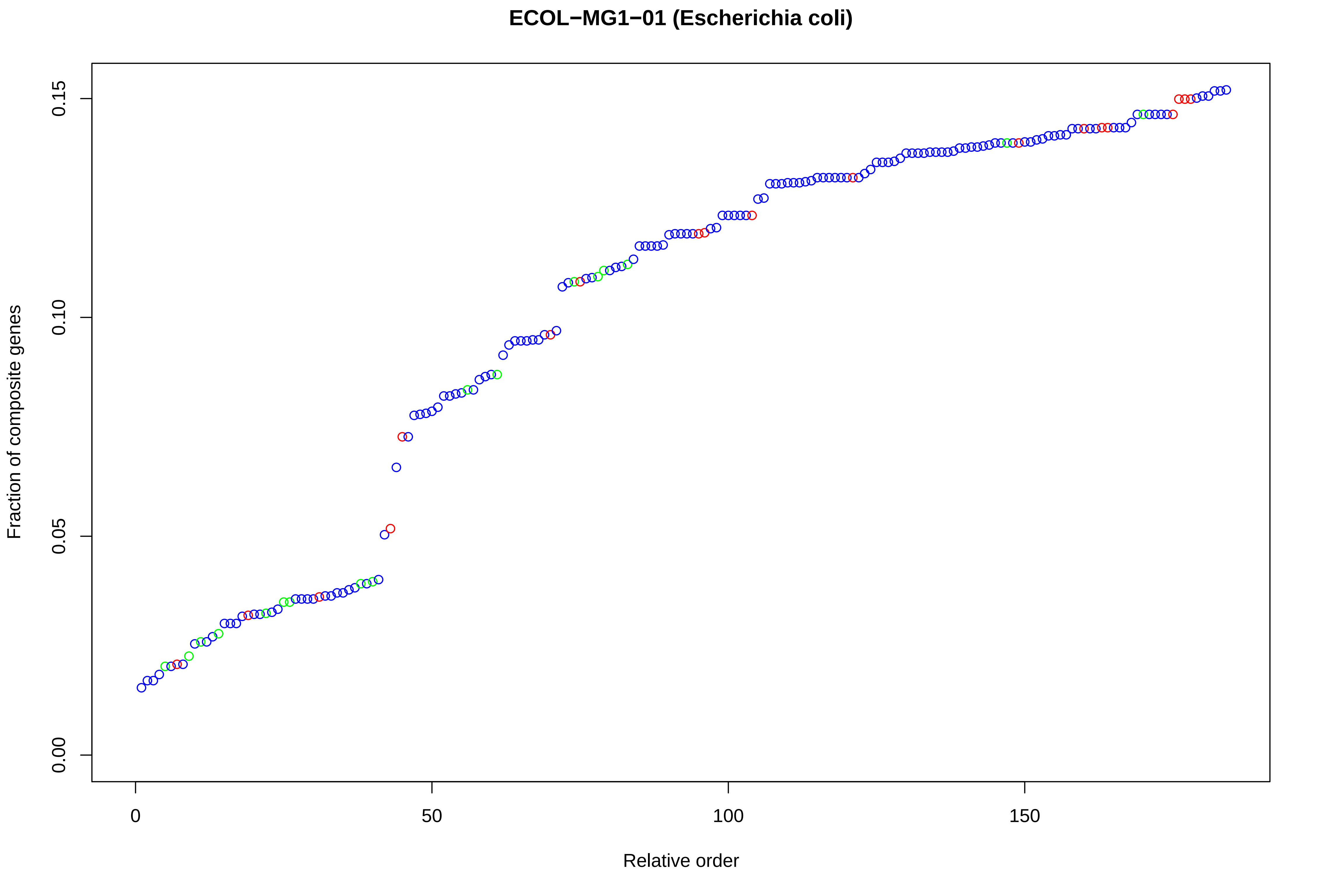

ECOL−MG1−01 (Escherichia coli)
Fraction of composite genes
0.00
0.05
0.10
0.15
Relative order
0
50
100
150

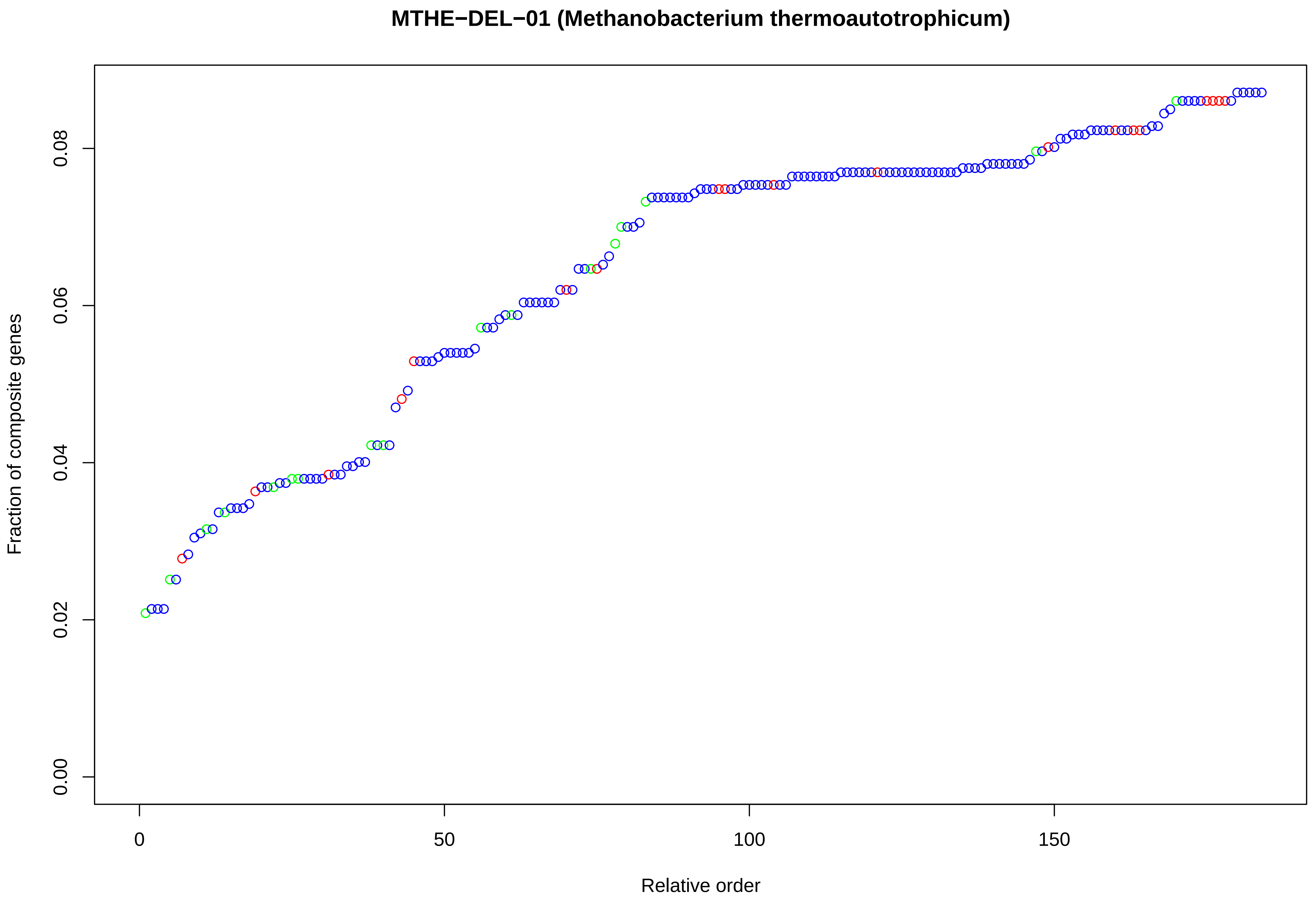
MTHE−DEL−01 (Methanobacterium thermoautotrophicum)
Fraction of composite genes
Relative order
0.00
0.02
0.04
0.06
0.08
0
50
100
150

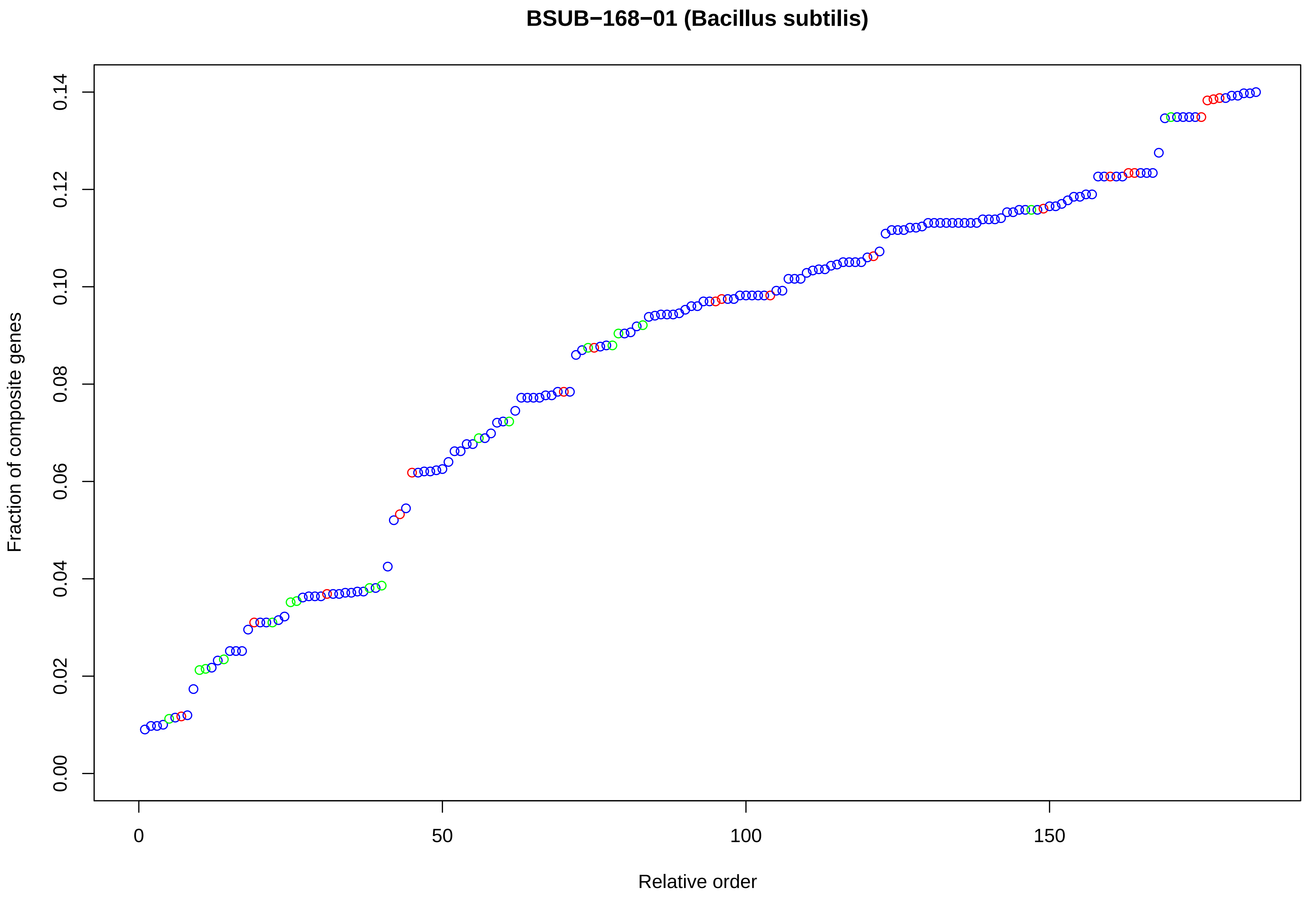

BSUB−168−01 (Bacillus subtilis)
Fraction of composite genes
0.00
0.02
0.04
0.06
0.08
0.10
0.12
0.14
Relative order
0
50
100
150

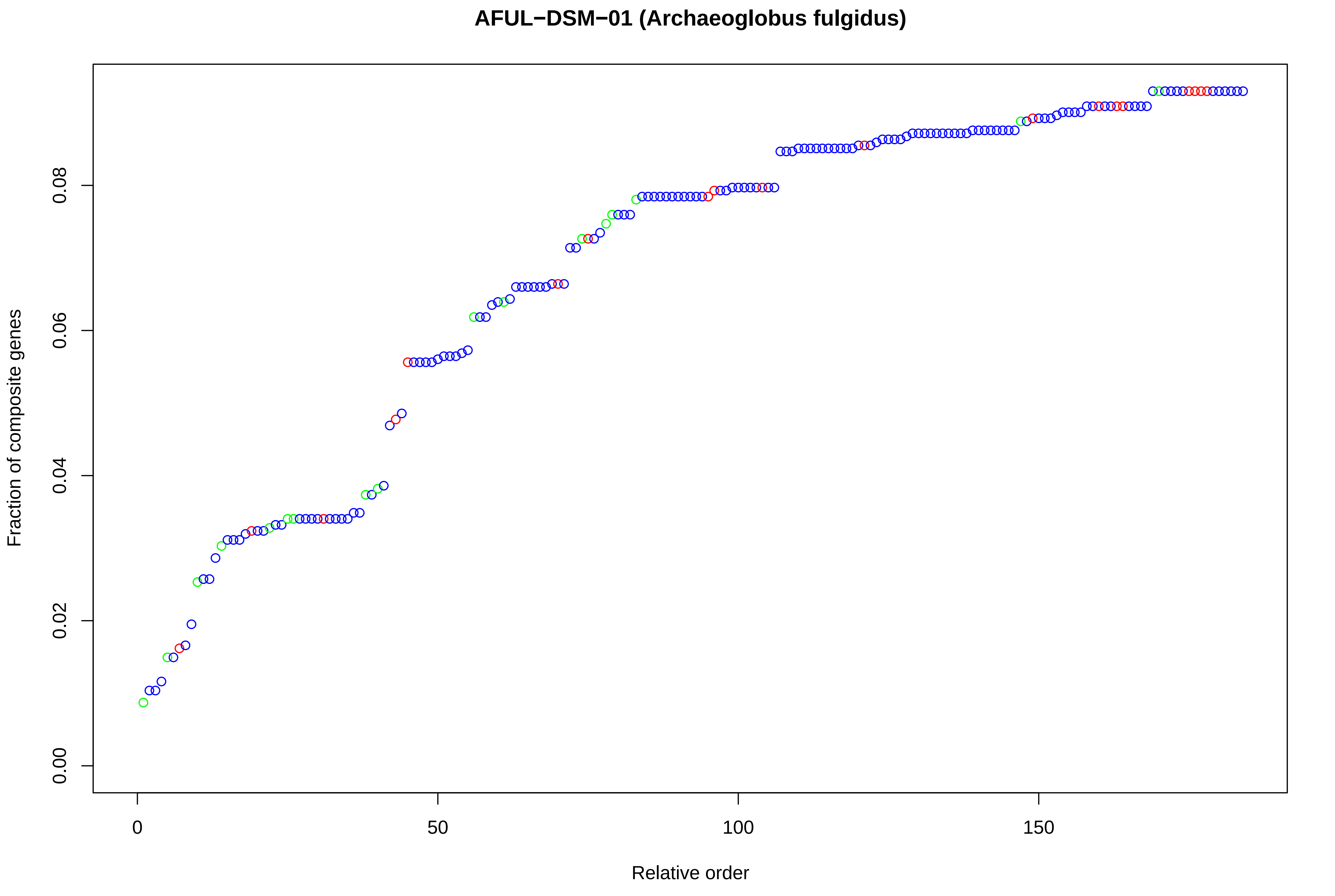

AFUL−DSM−01 (Archaeoglobus fulgidus)
Fraction of composite genes
0.00
0.02
0.04
0.06
0.08
0
50
100
150
Relative order

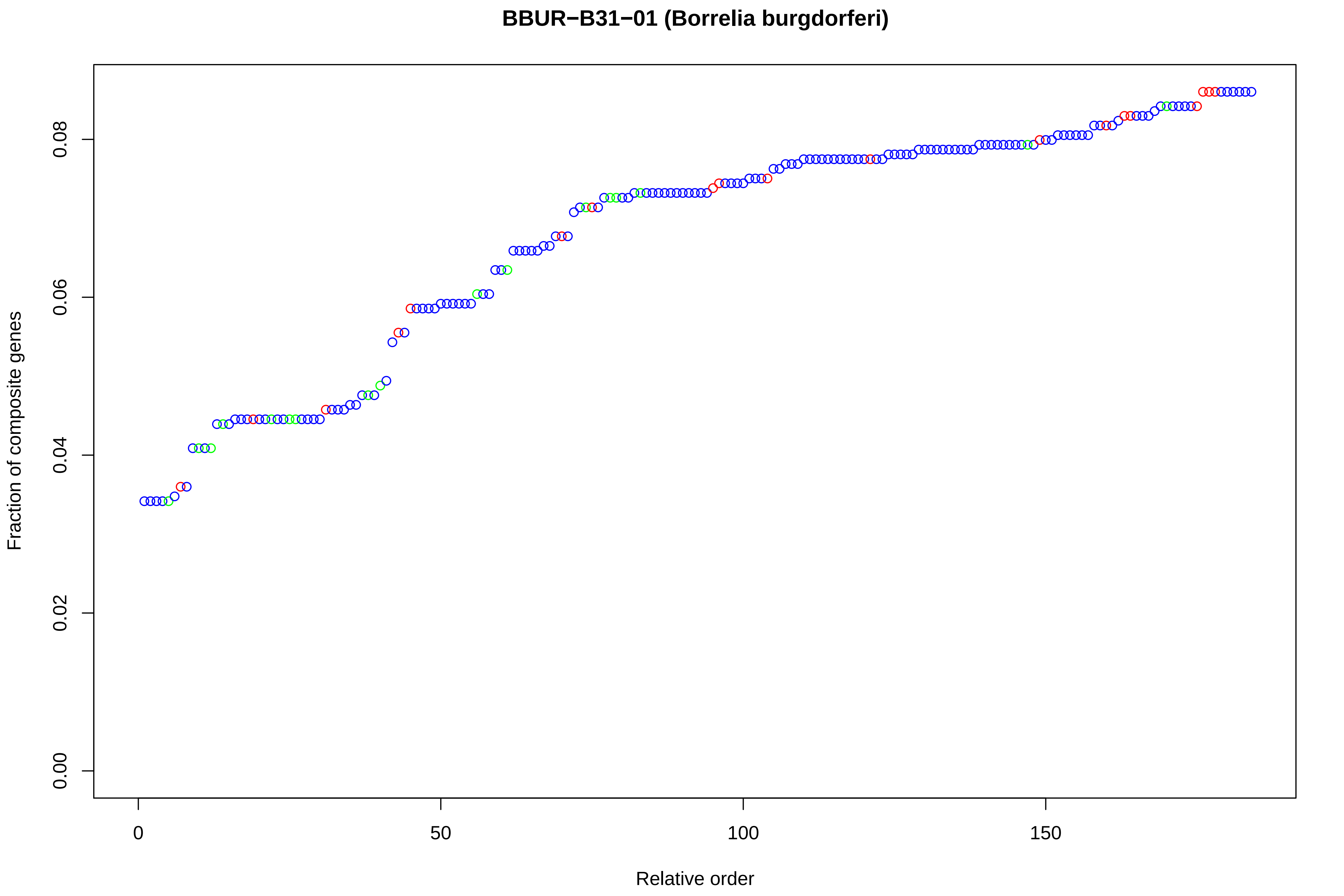

BBUR−B31−01 (Borrelia burgdorferi)
Fraction of composite genes
0.00
0.02
0.04
0.06
0.08
Relative order
0
50
100
150

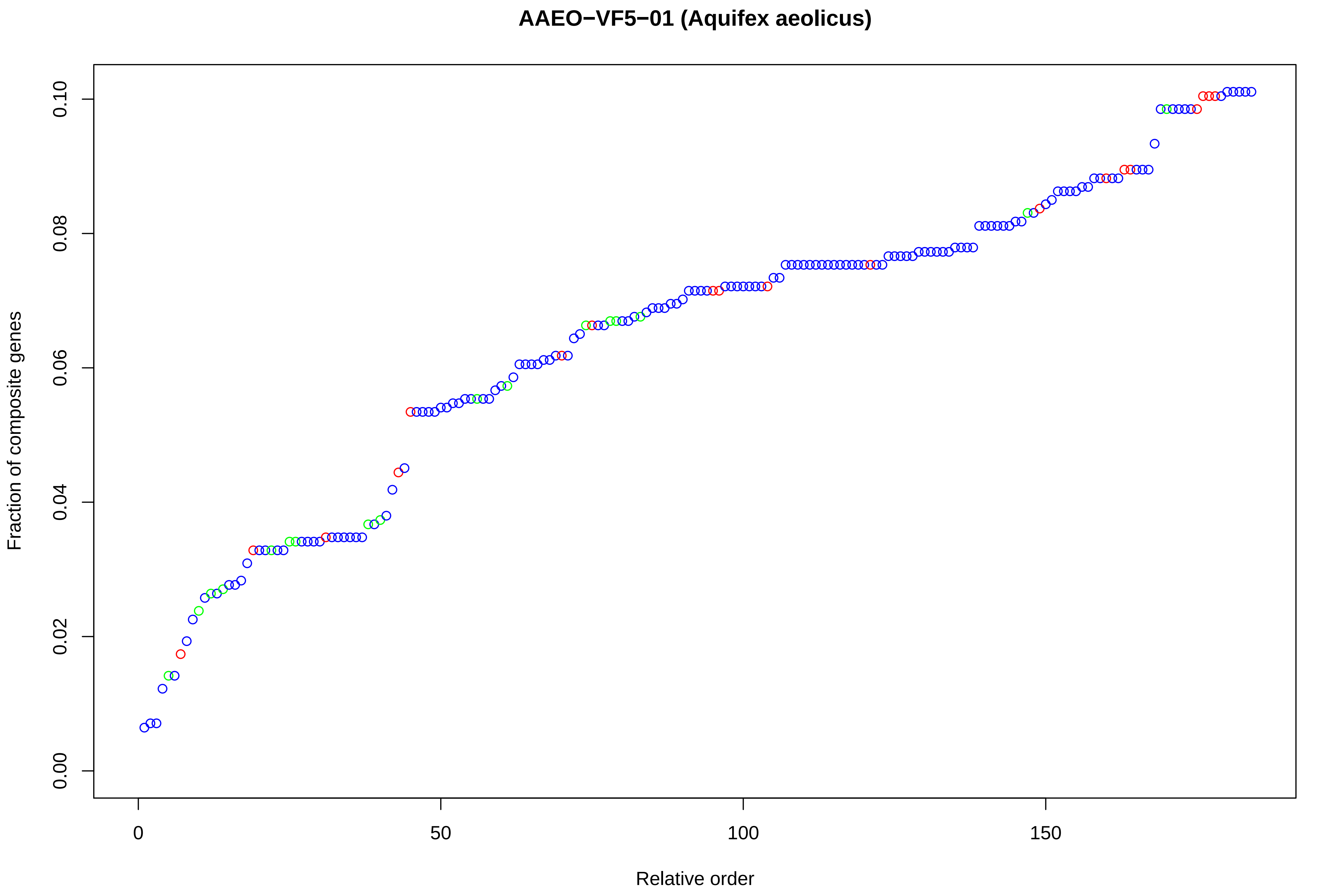
AAEO−VF5−01 (Aquifex aeolicus)
Fraction of composite genes
0.00
0.02
0.04
0.06
0.08
0.10
0
50
100
150
Relative order

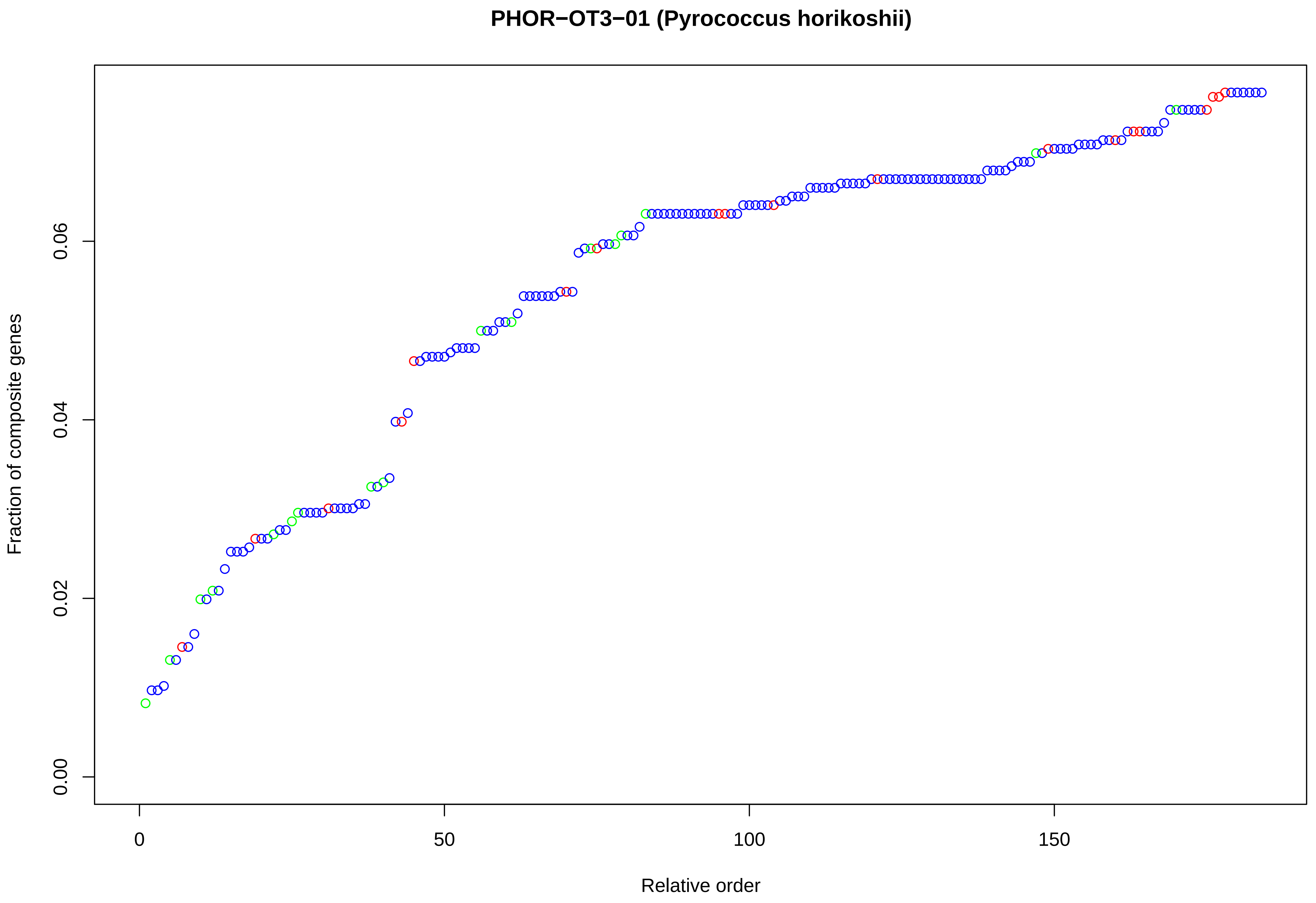
PHOR−OT3−01 (Pyrococcus horikoshii)
Fraction of composite genes
0.00
0.02
0.04
0.06
Relative order
0
50
100
150

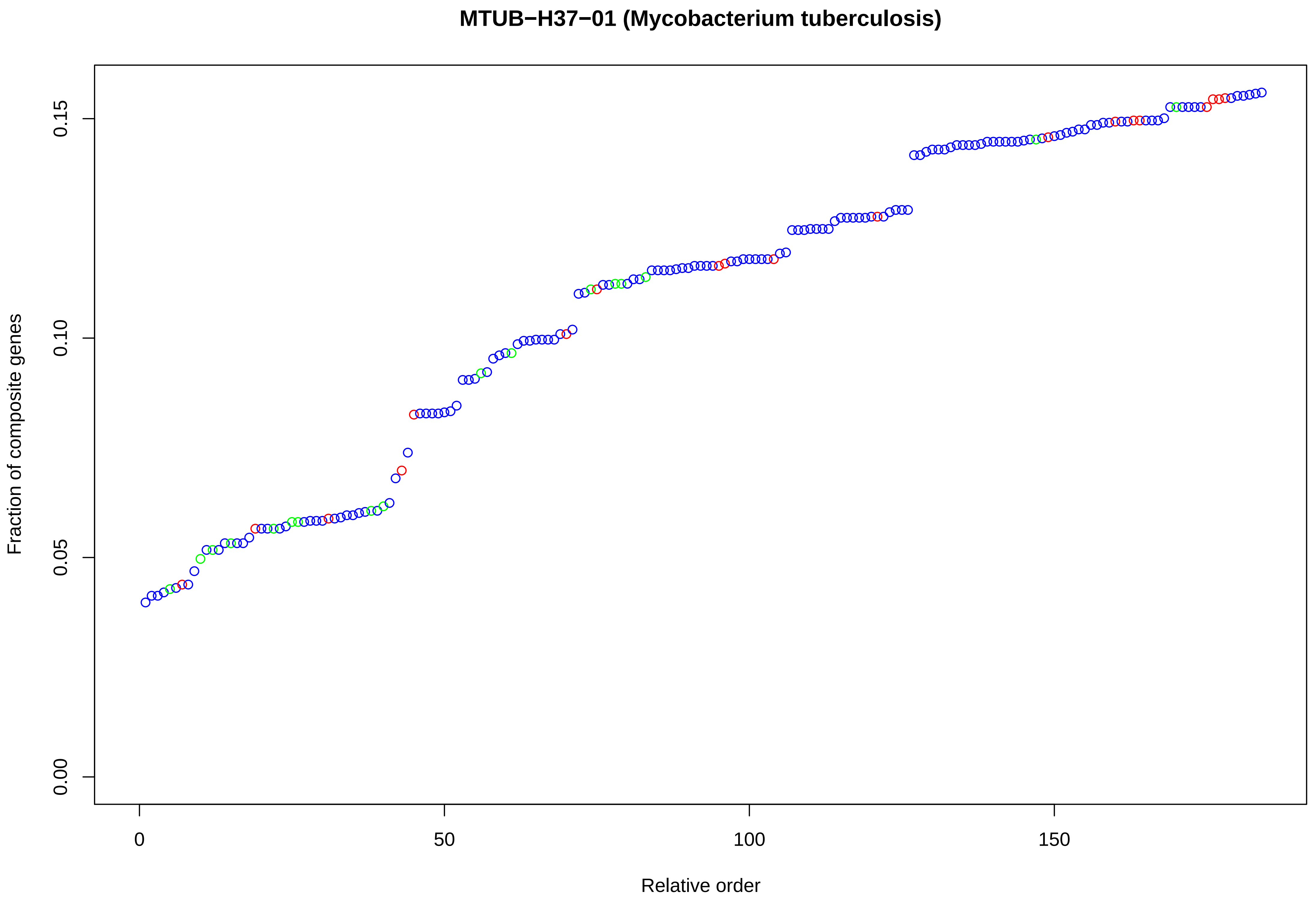
MTUB−H37−01 (Mycobacterium tuberculosis)
Fraction of composite genes
0.00
0.05
0.10
0.15
0
50
100
150
Relative order

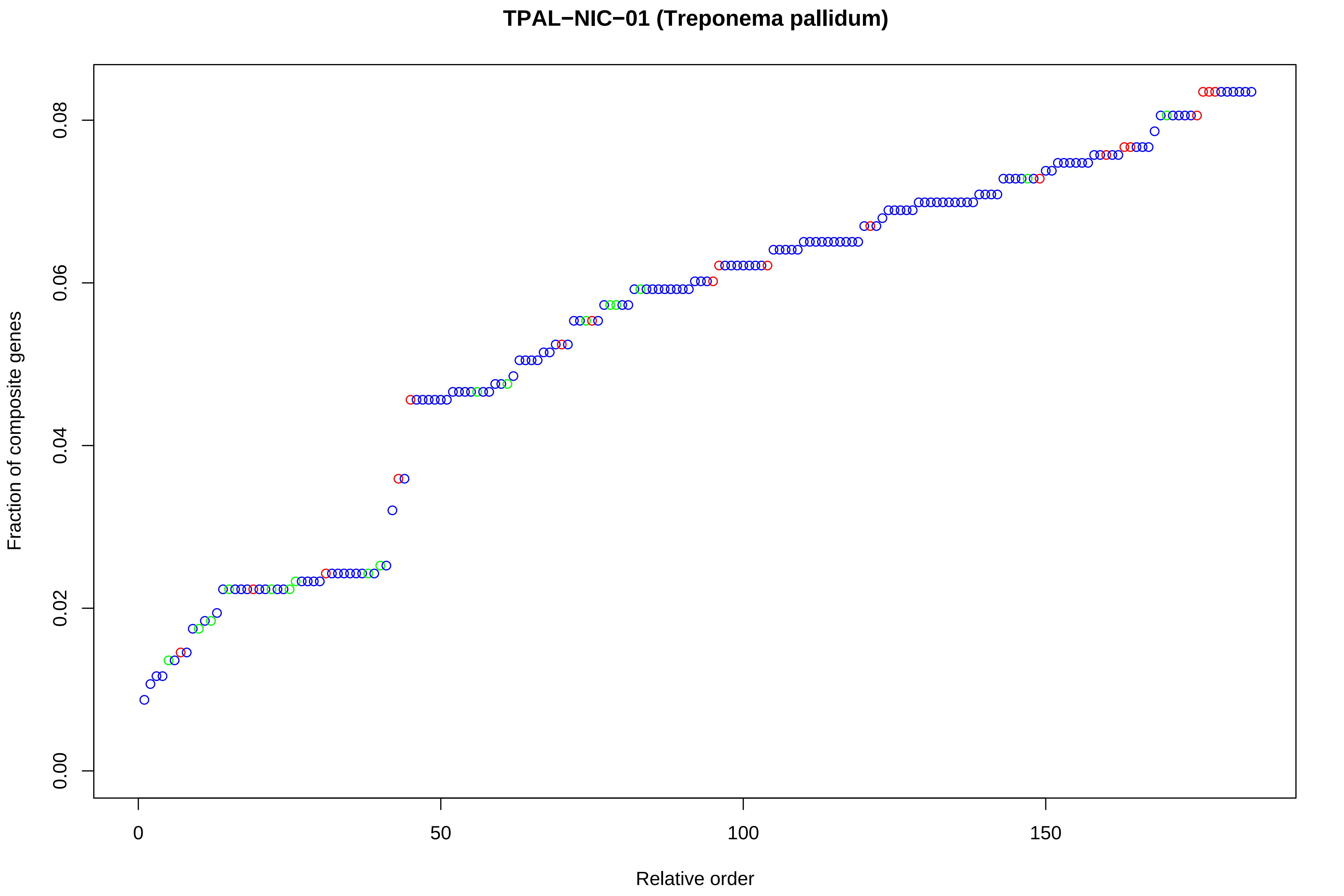
TPAL−NIC−01 (Treponema pallidum)
Fraction of composite genes
0.00
0.02
0.04
0.06
0.08
Relative order
0
50
100
150

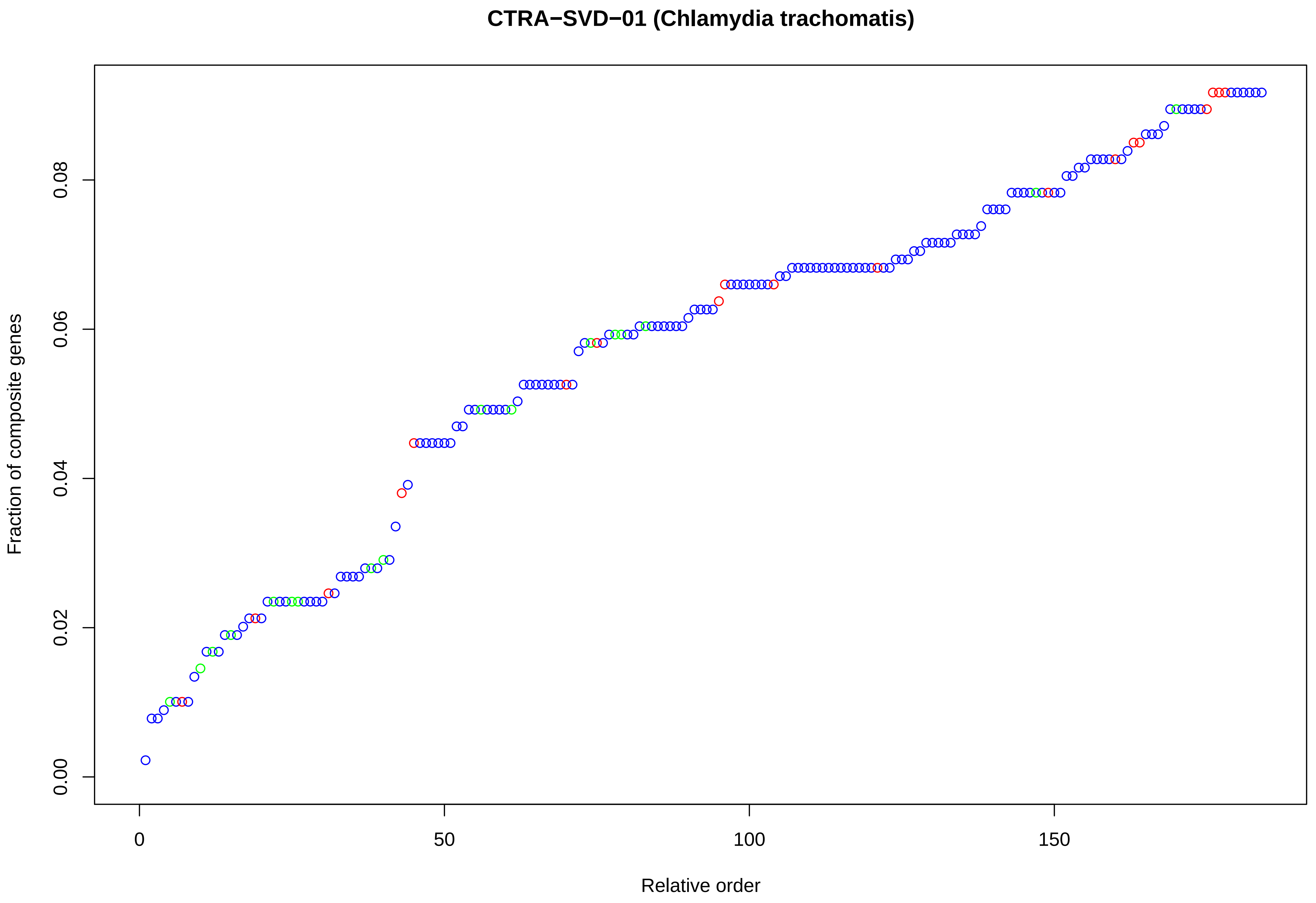
CTRA−SVD−01 (Chlamydia trachomatis)
Fraction of composite genes
0.00
0.02
0.04
0.06
0.08
Relative order
0
50
100
150

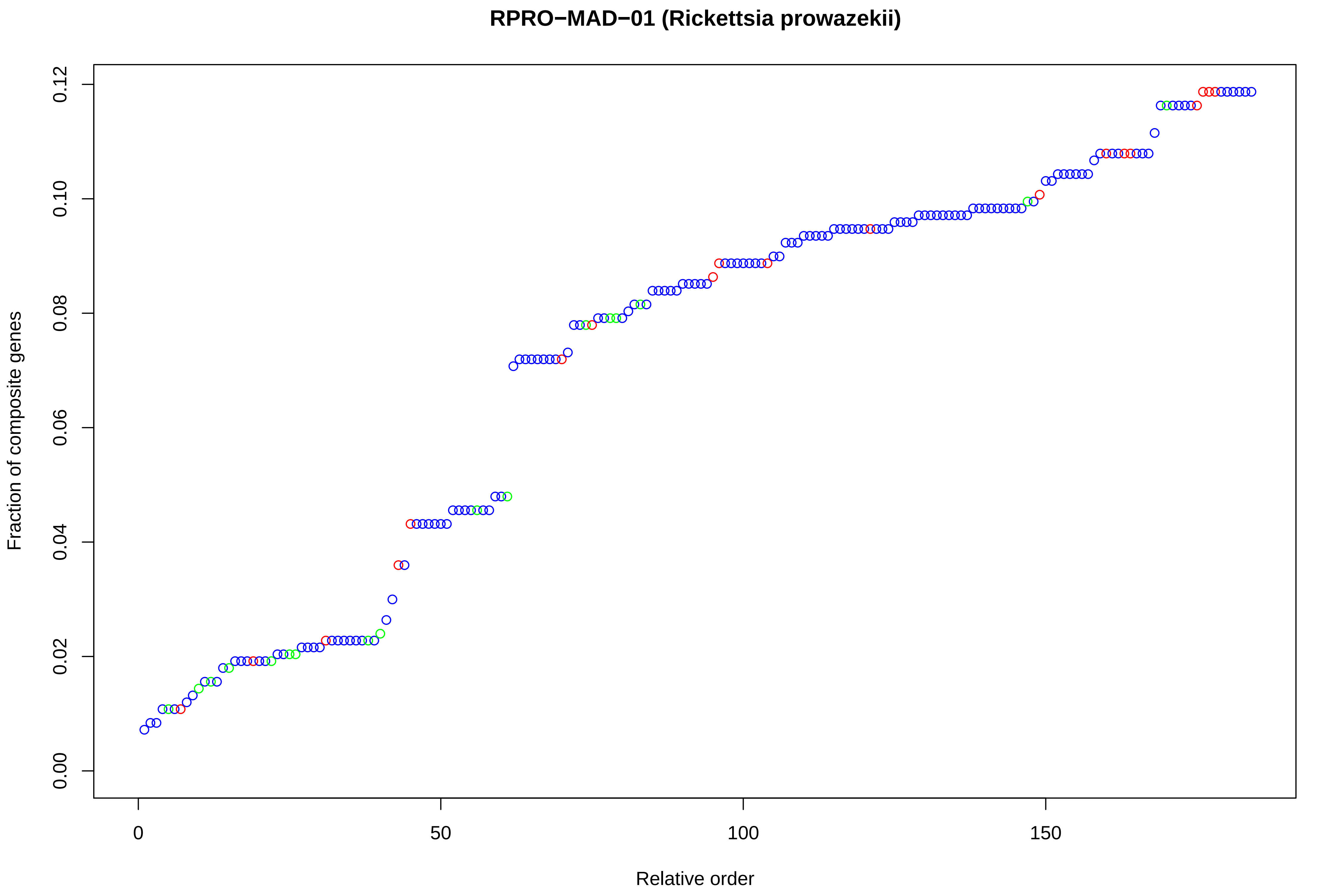
RPRO−MAD−01 (Rickettsia prowazekii)
Fraction of composite genes
0.00
0.02
0.04
0.06
0.08
0.10
0.12
Relative order
0
50
100
150

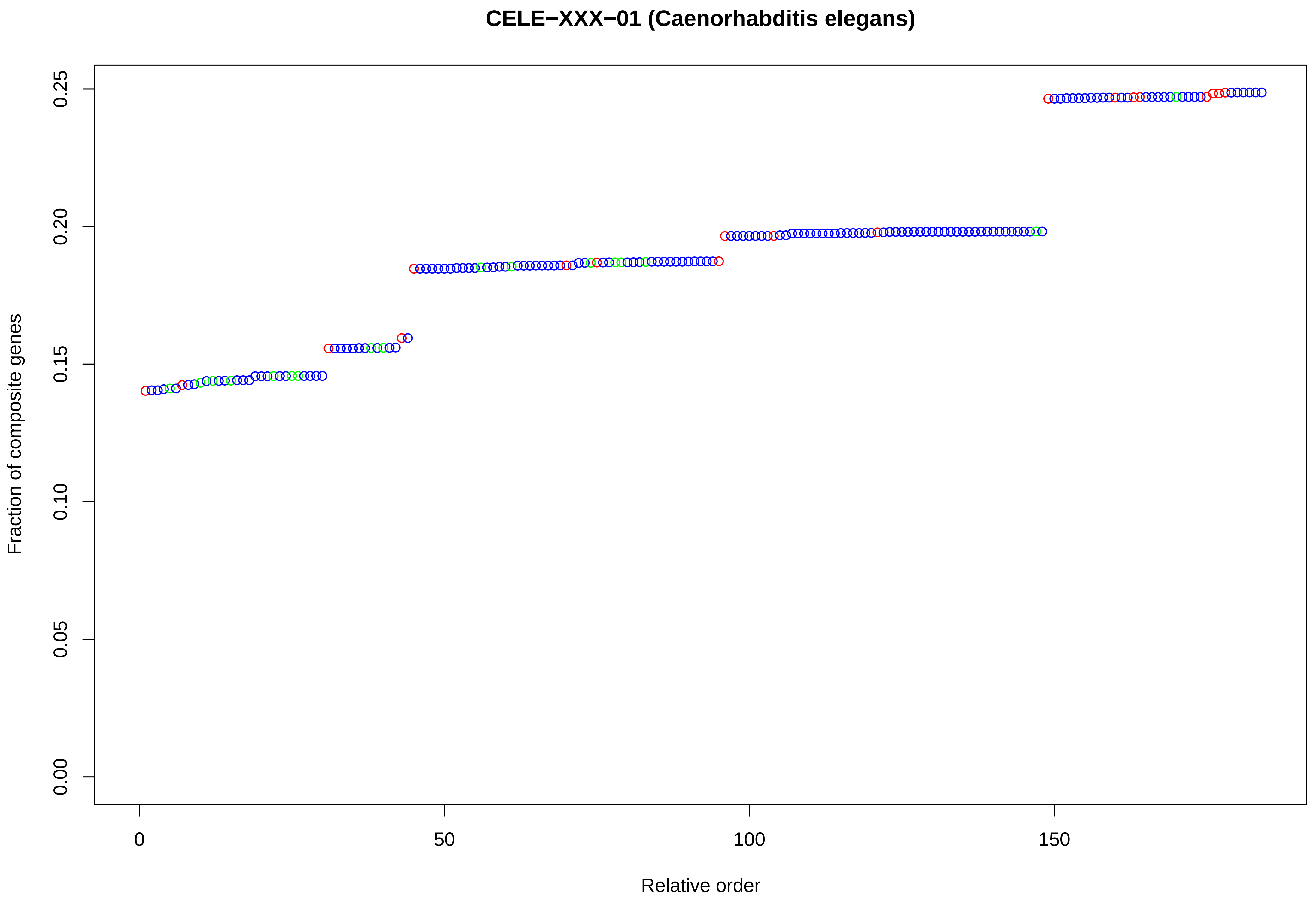

CELE−XXX−01 (Caenorhabditis elegans)
Fraction of composite genes
0.00
0.05
0.10
0.15
0.20
0.25
0
50
100
150
Relative order

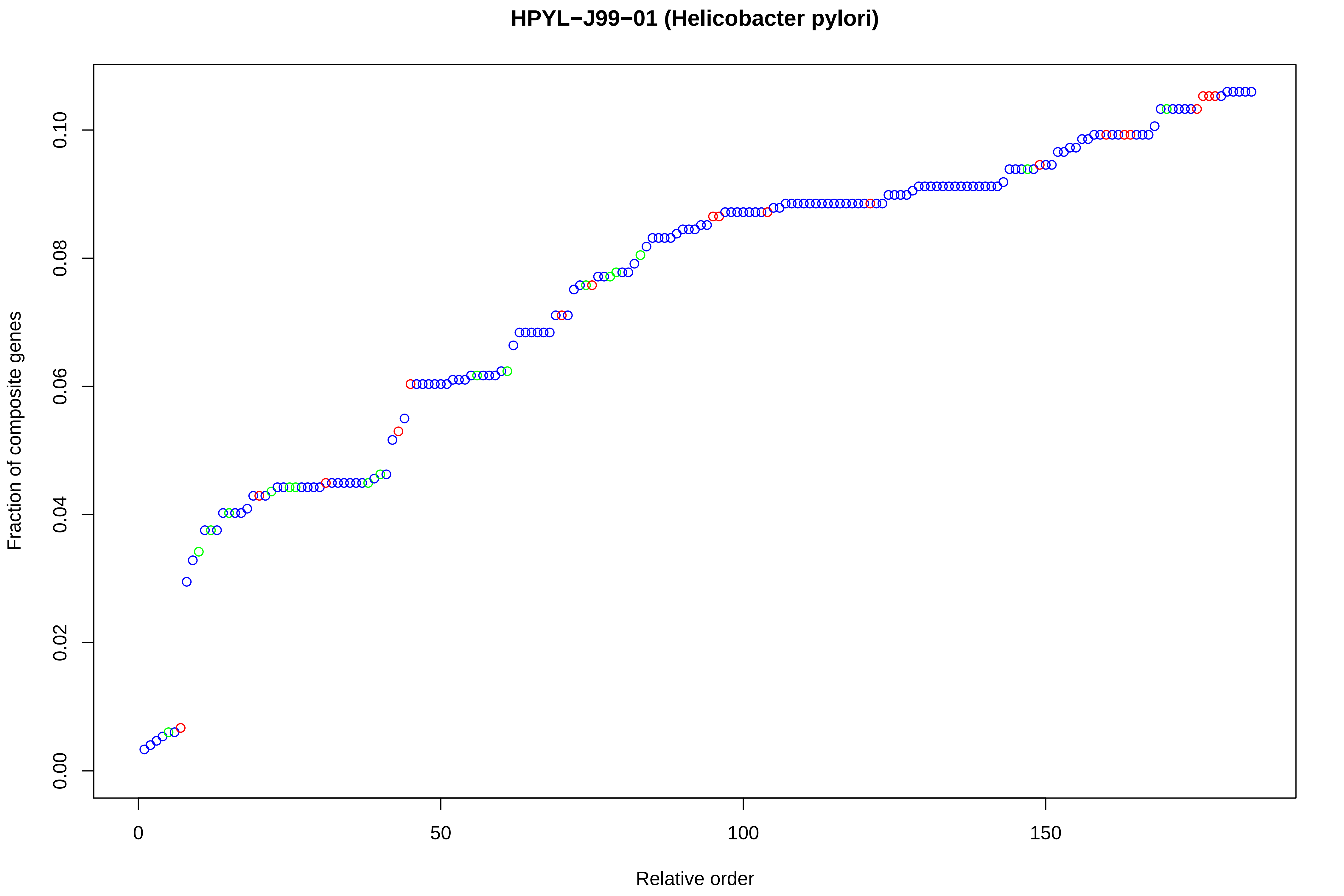
HPYL−J99−01 (Helicobacter pylori)
Fraction of composite genes
0.00
0.02
0.04
0.06
0.08
0.10
0
50
100
150
Relative order

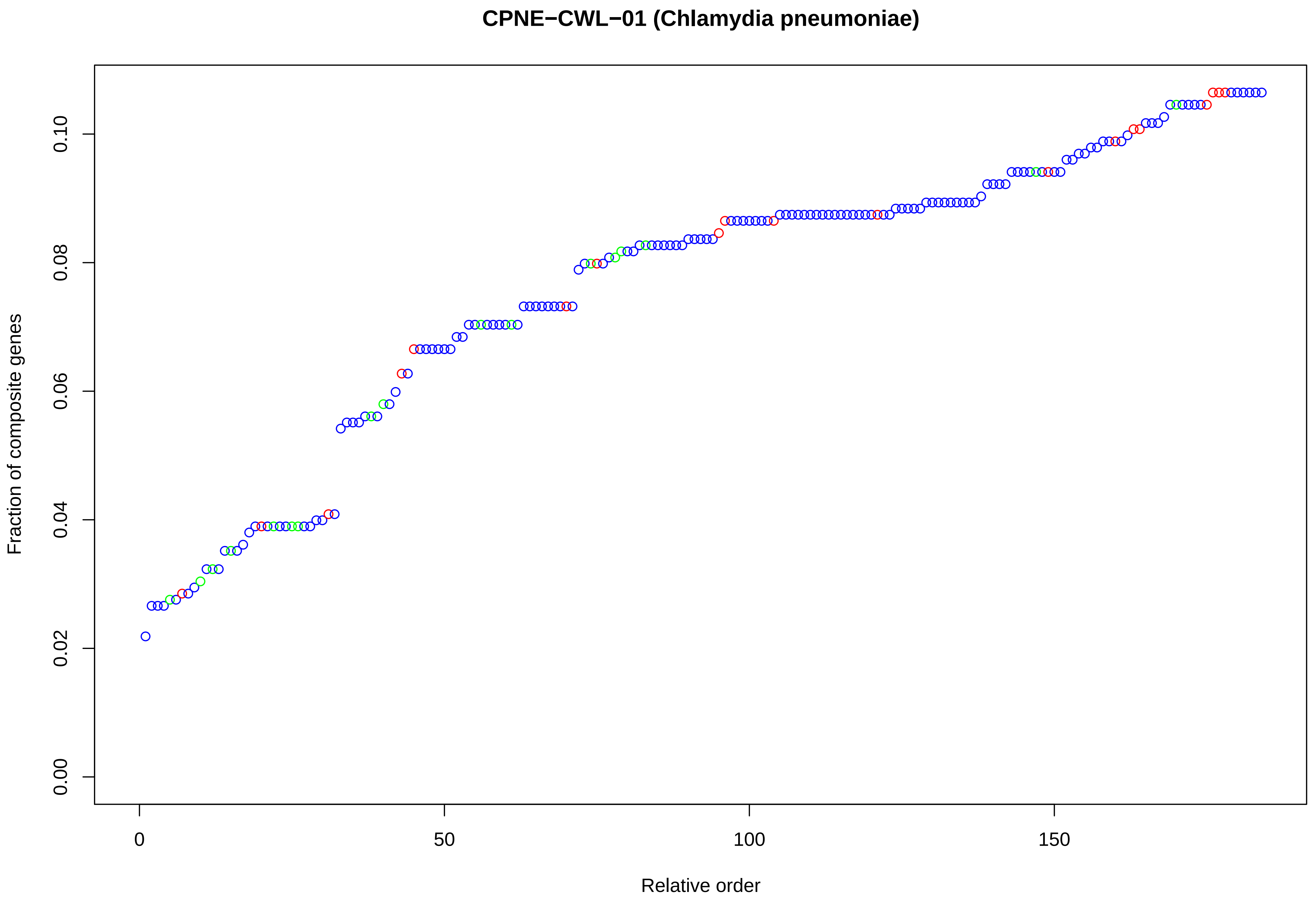
CPNE−CWL−01 (Chlamydia pneumoniae)
Fraction of composite genes
0.00
0.02
0.04
0.06
0.08
0.10
0
50
100
150
Relative order

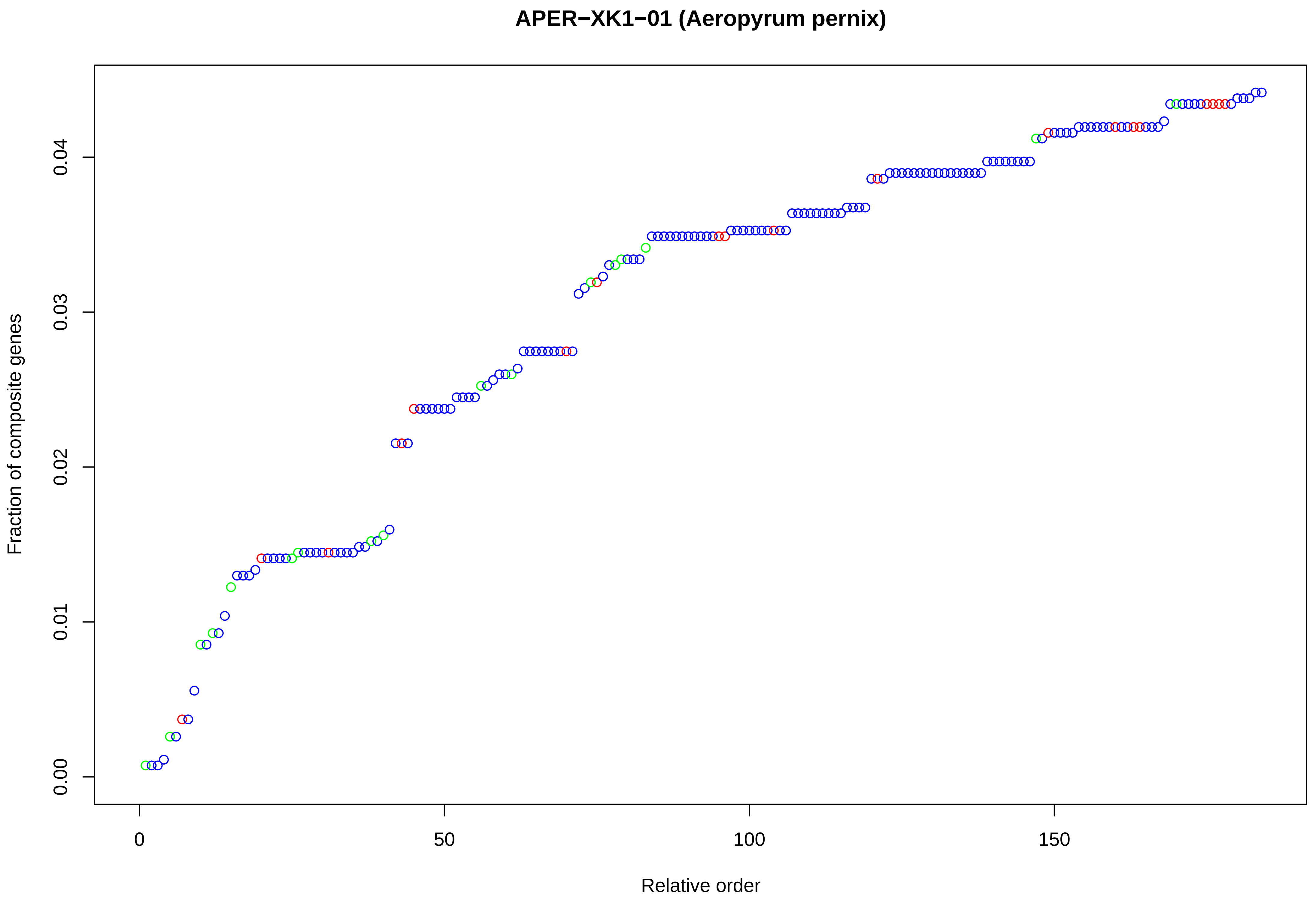
APER−XK1−01 (Aeropyrum pernix)
Fraction of composite genes
0.00
0.01
0.02
0.03
0.04
0
50
100
150
Relative order

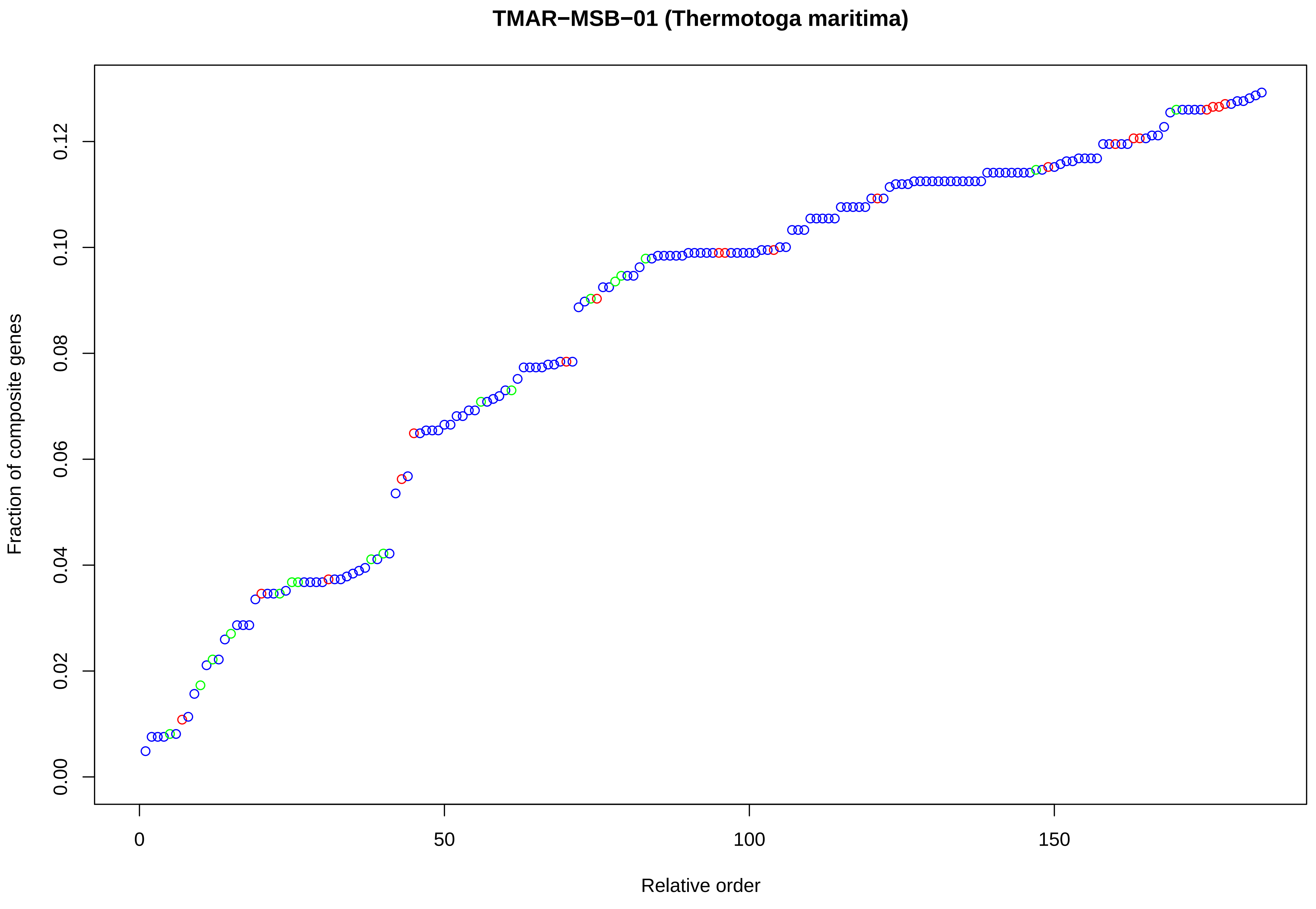

TMAR−MSB−01 (Thermotoga maritima)
Fraction of composite genes
0.00
0.02
0.04
0.06
0.08
0.10
0.12
Relative order
0
50
100
150

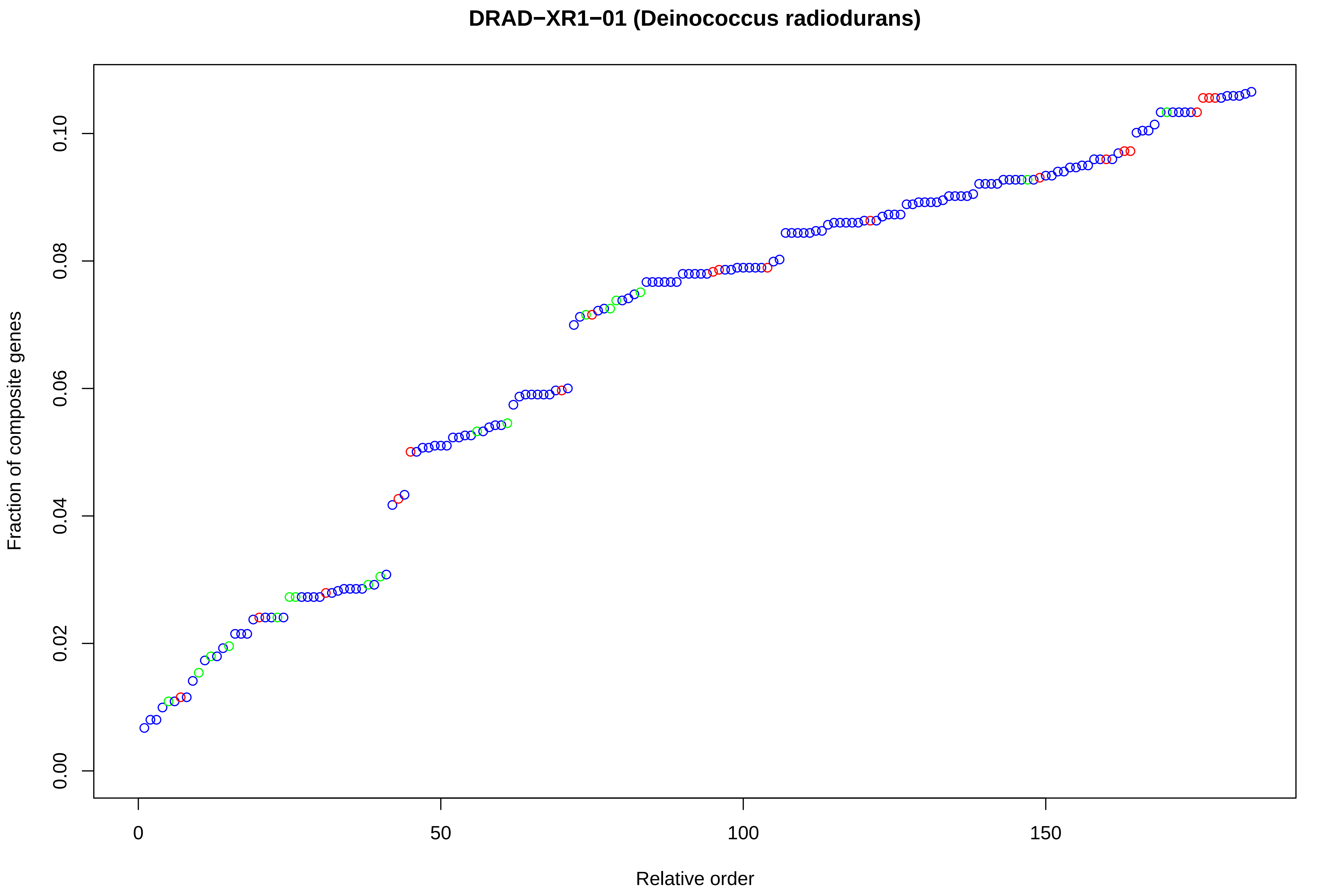

DRAD−XR1−01 (Deinococcus radiodurans)
Fraction of composite genes
0.00
0.02
0.04
0.06
0.08
0.10
0
50
100
150
Relative order

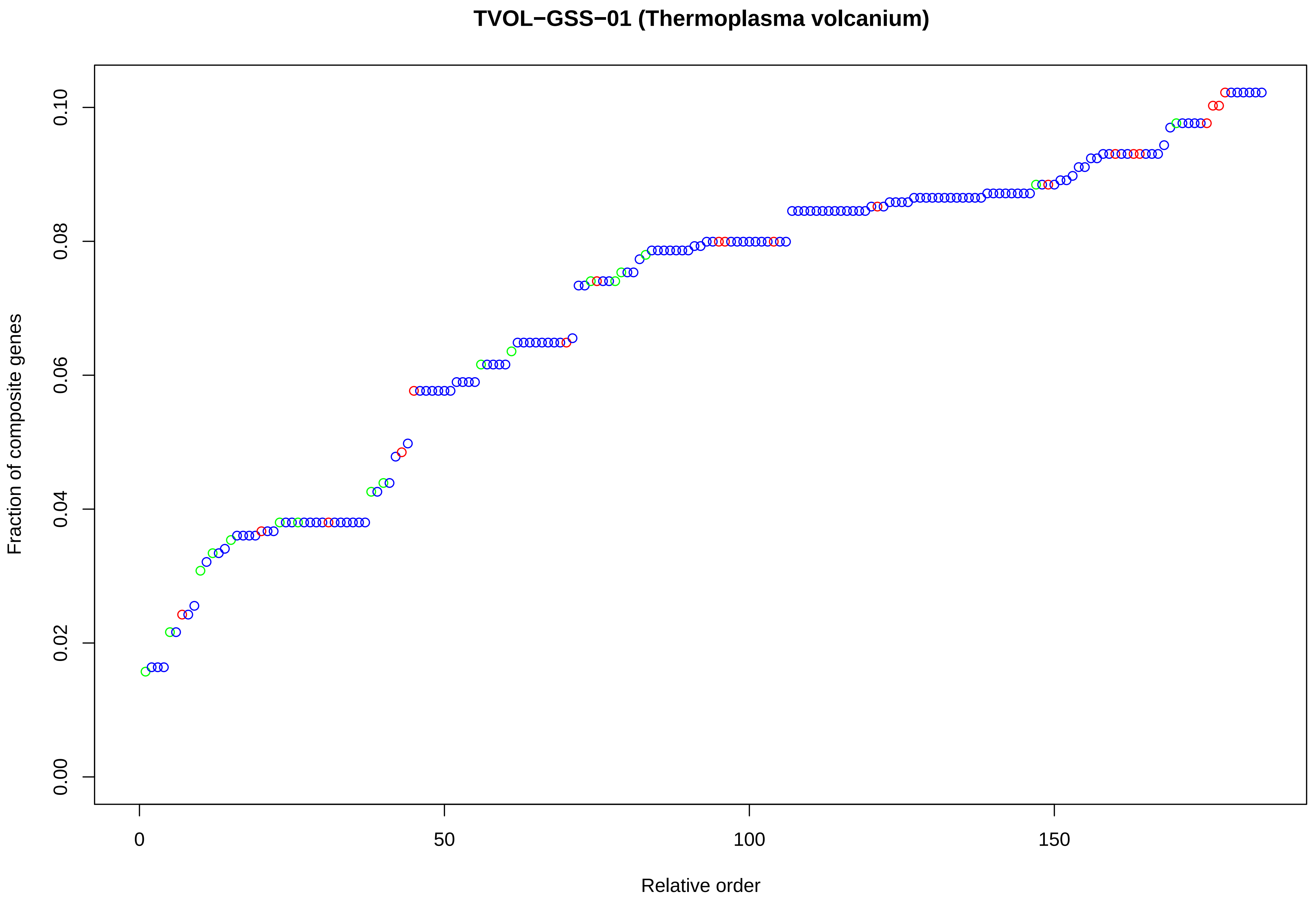
TVOL−GSS−01 (Thermoplasma volcanium)
Fraction of composite genes
0.00
0.02
0.04
0.06
0.08
0.10
Relative order
0
50
100
150

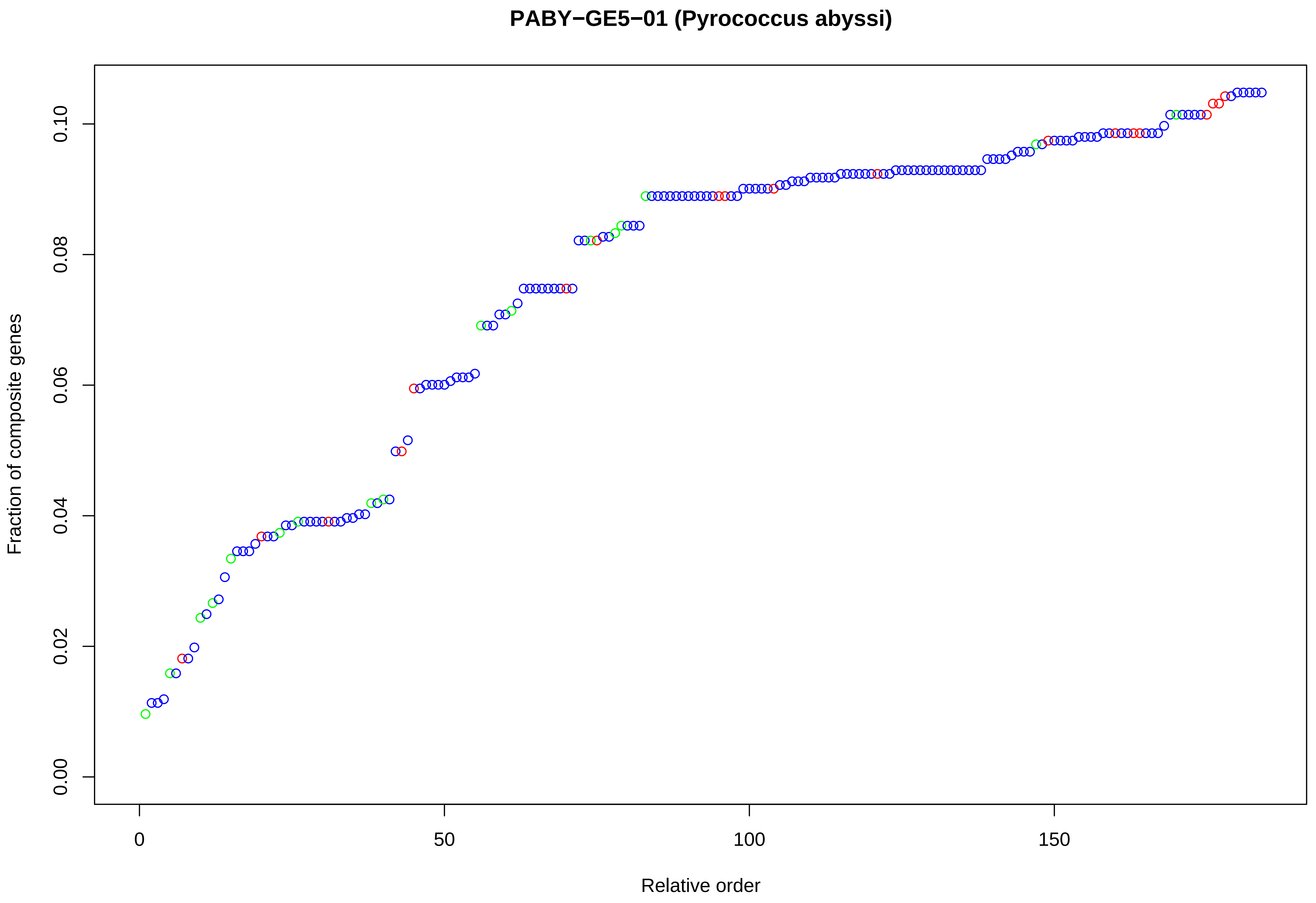
PABY−GE5−01 (Pyrococcus abyssi)
Fraction of composite genes
Relative order
0.00
0.02
0.04
0.06
0.08
0.10
0
50
100
150

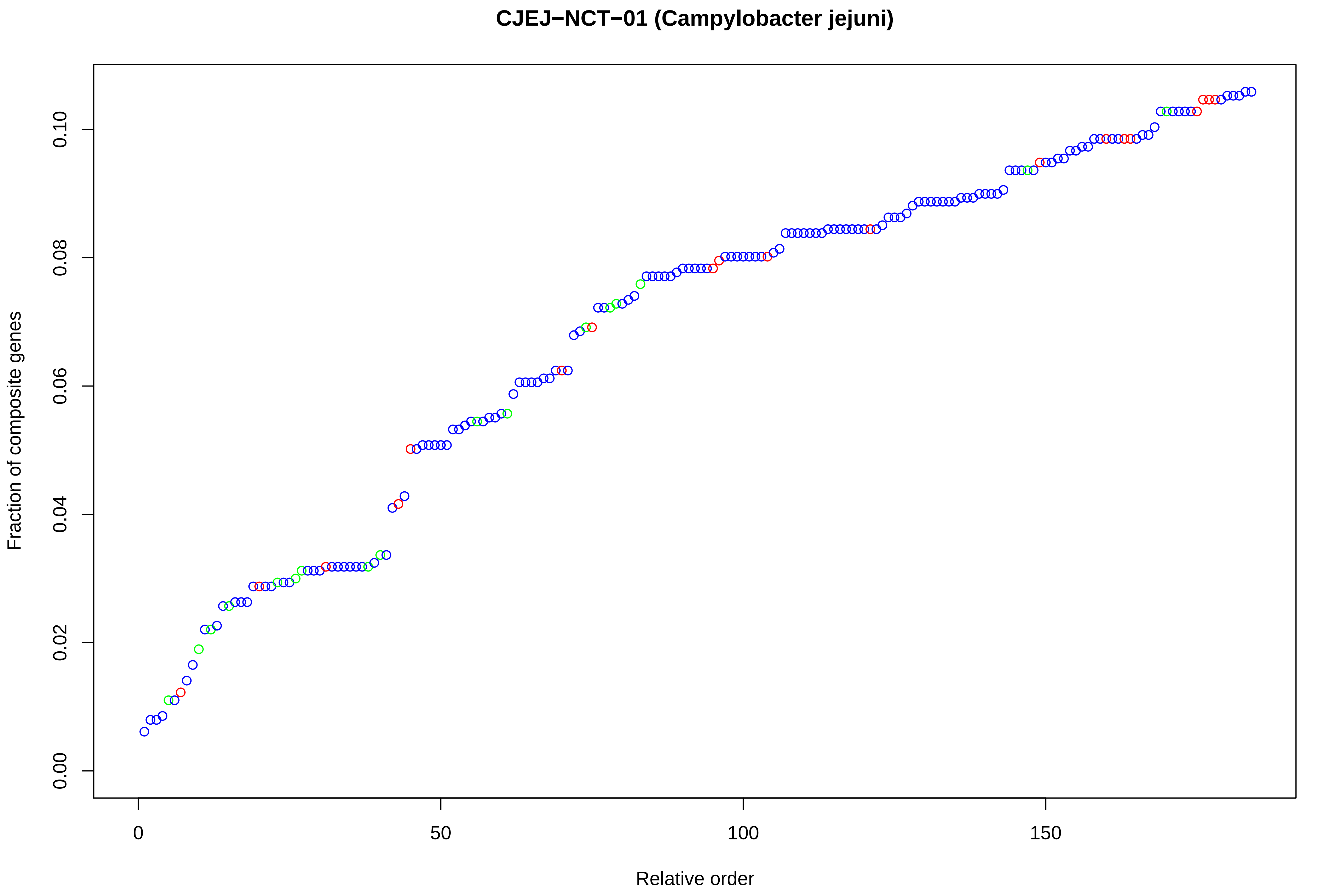
CJEJ−NCT−01 (Campylobacter jejuni)
Fraction of composite genes
0.00
0.02
0.04
0.06
0.08
0.10
0
50
100
150
Relative order

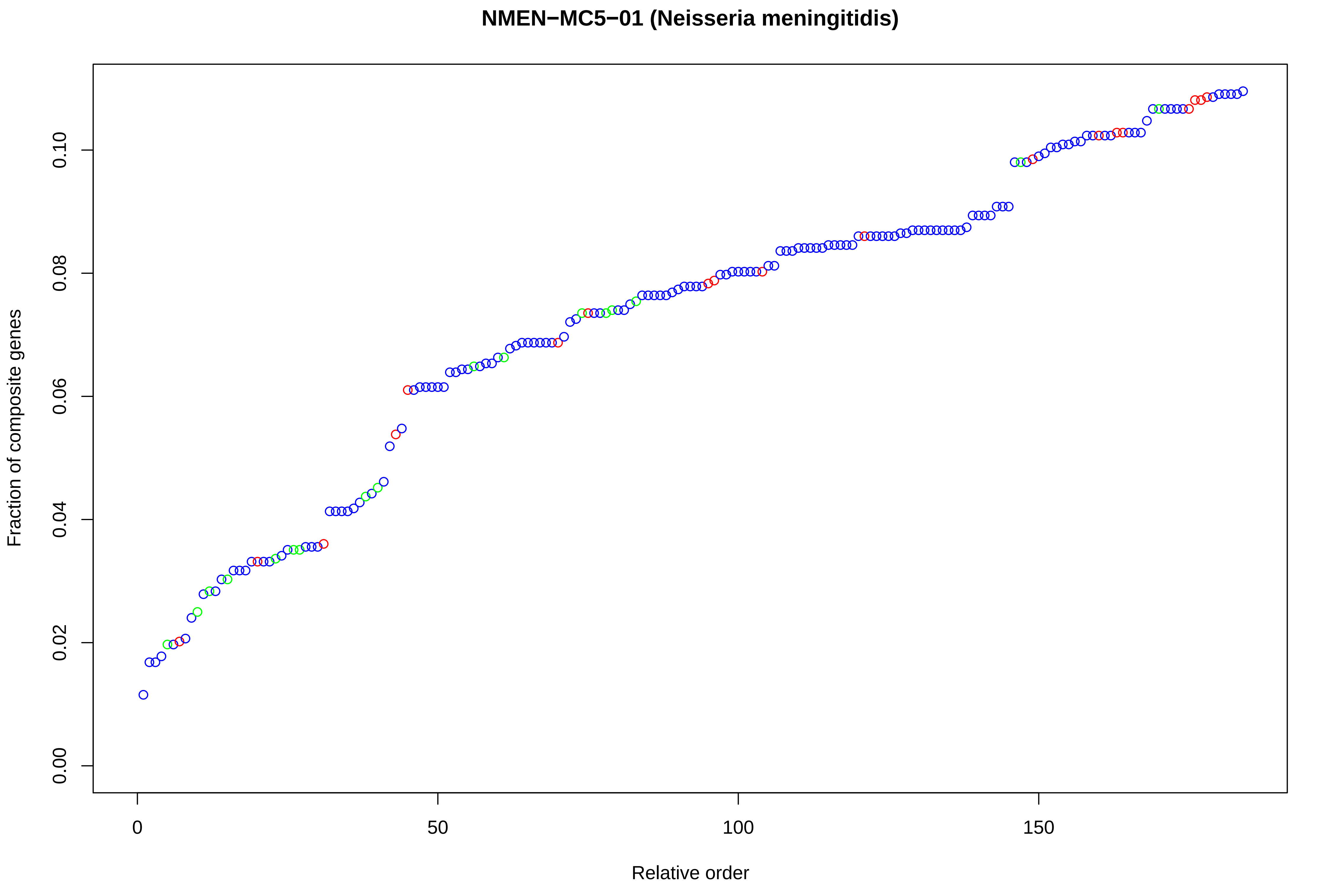
NMEN−MC5−01 (Neisseria meningitidis)
Fraction of composite genes
Relative order
0.00
0.02
0.04
0.06
0.08
0.10
0
50
100
150

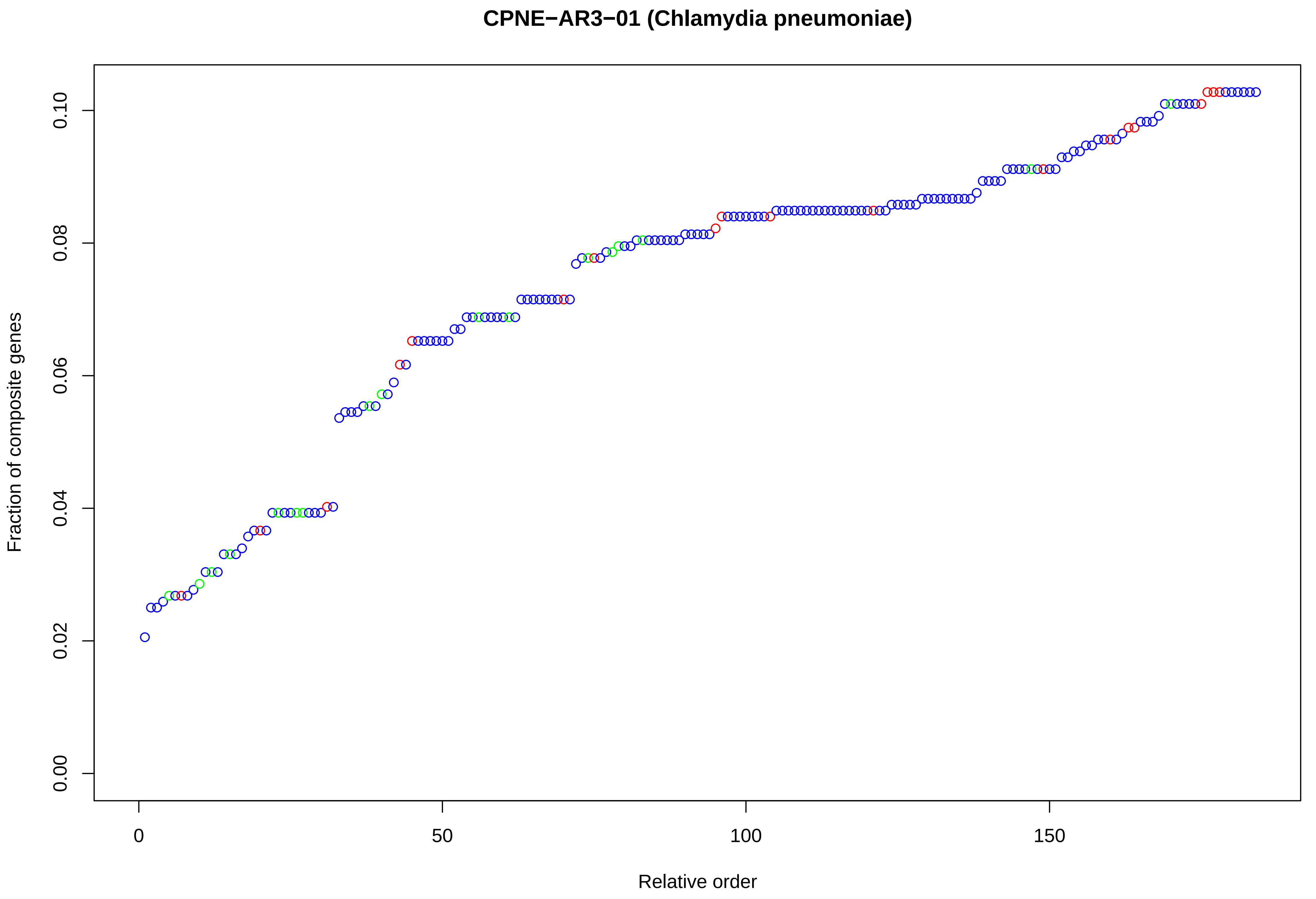
CPNE−AR3−01 (Chlamydia pneumoniae)
Fraction of composite genes
Relative order
0.00
0.02
0.04
0.06
0.08
0.10
0
50
100
150

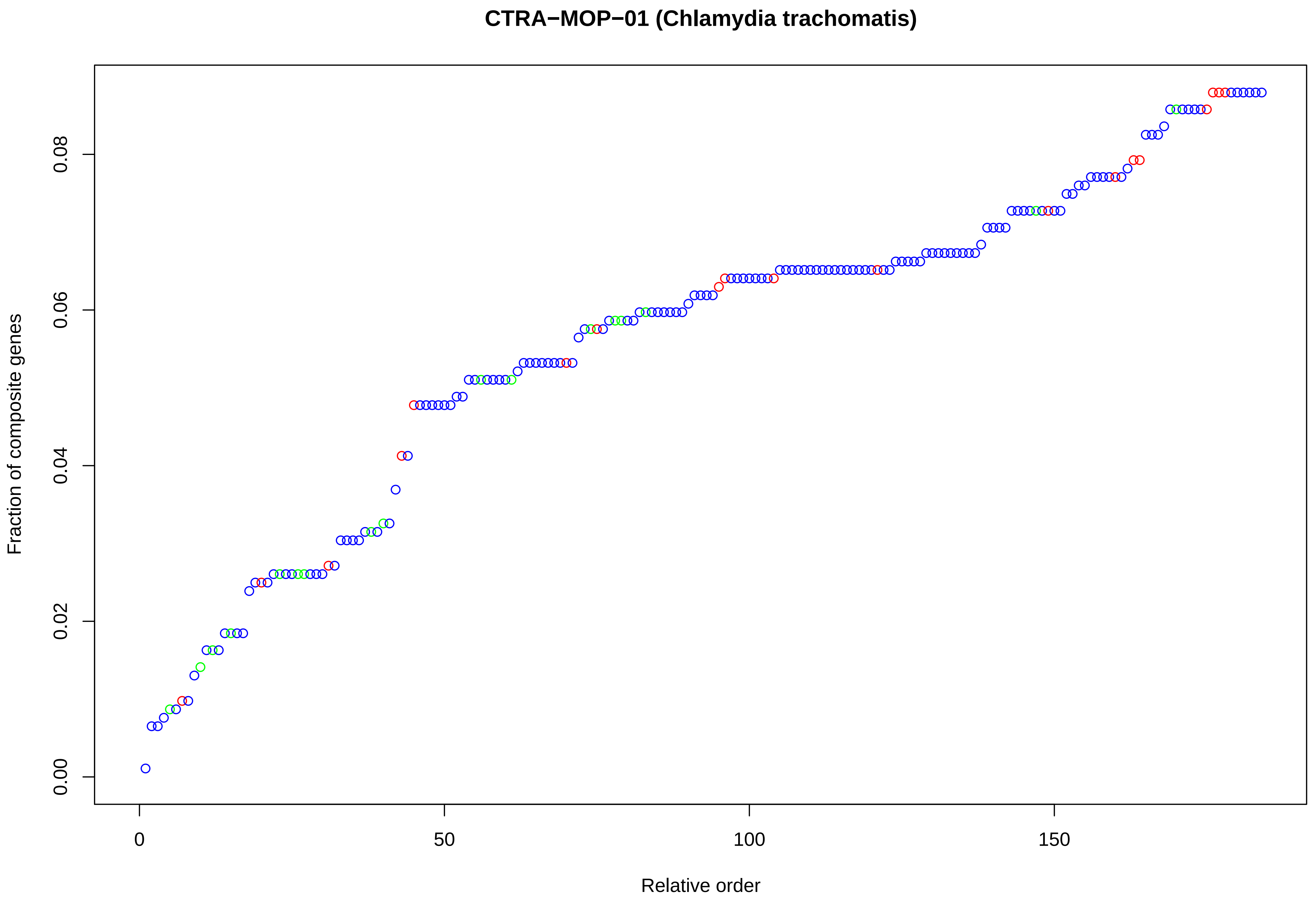
CTRA−MOP−01 (Chlamydia trachomatis)
Fraction of composite genes
0.00
0.02
0.04
0.06
0.08
Relative order
0
50
100
150

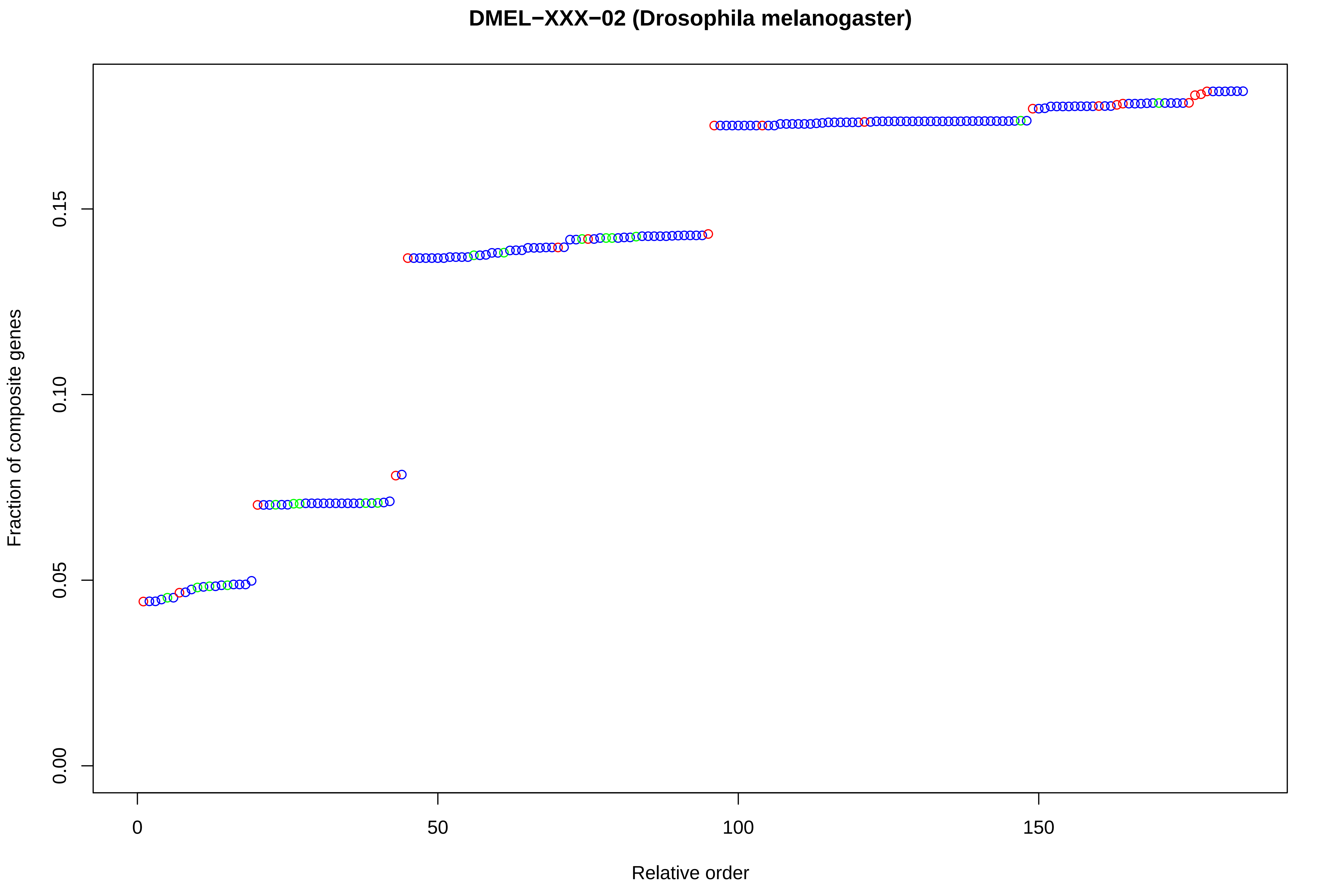
DMEL−XXX−02 (Drosophila melanogaster)
Fraction of composite genes
0.00
0.05
0.10
0.15
0
50
100
150
Relative order

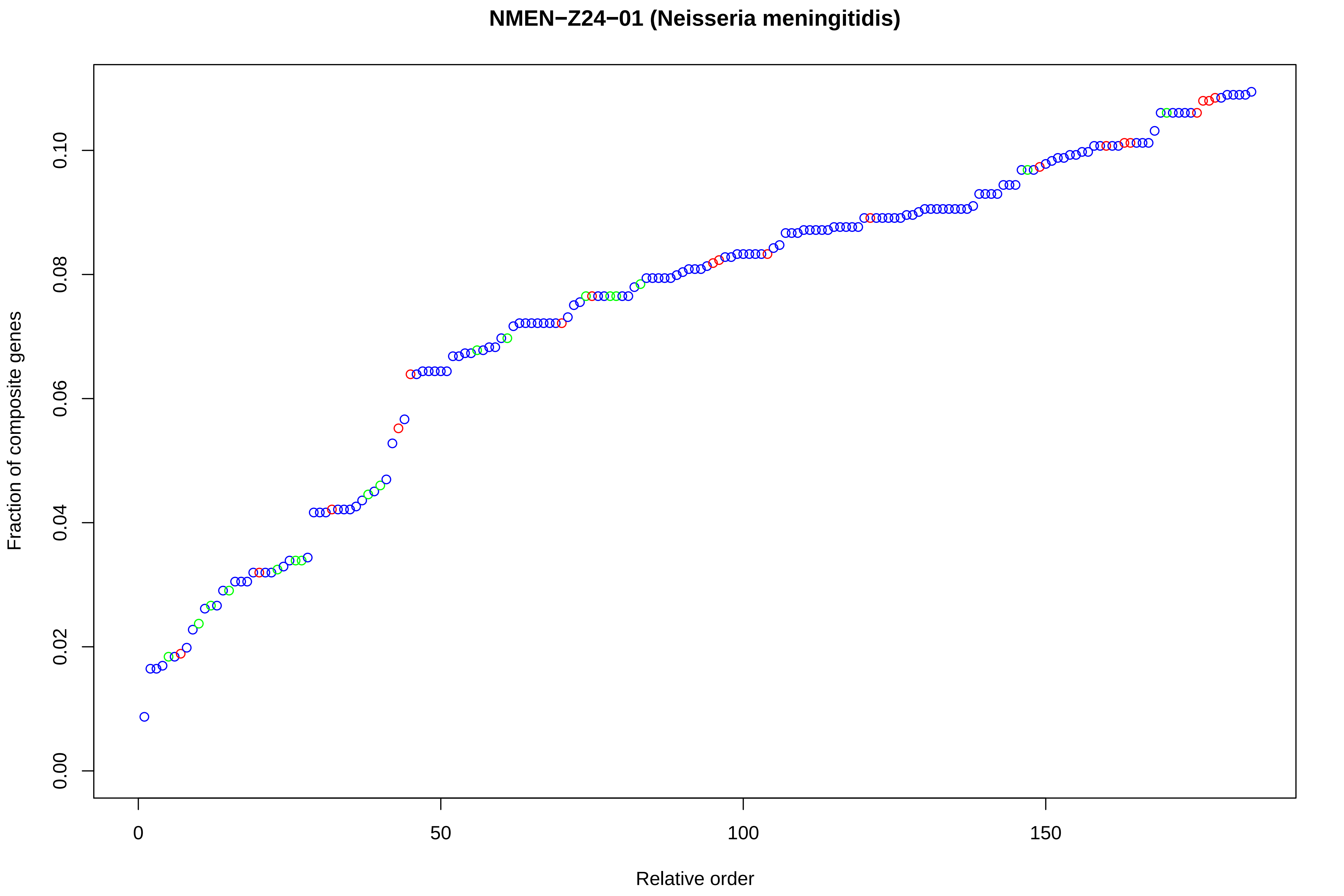
NMEN−Z24−01 (Neisseria meningitidis)
Fraction of composite genes
0.00
0.02
0.04
0.06
0.08
0.10
0
50
100
150
Relative order

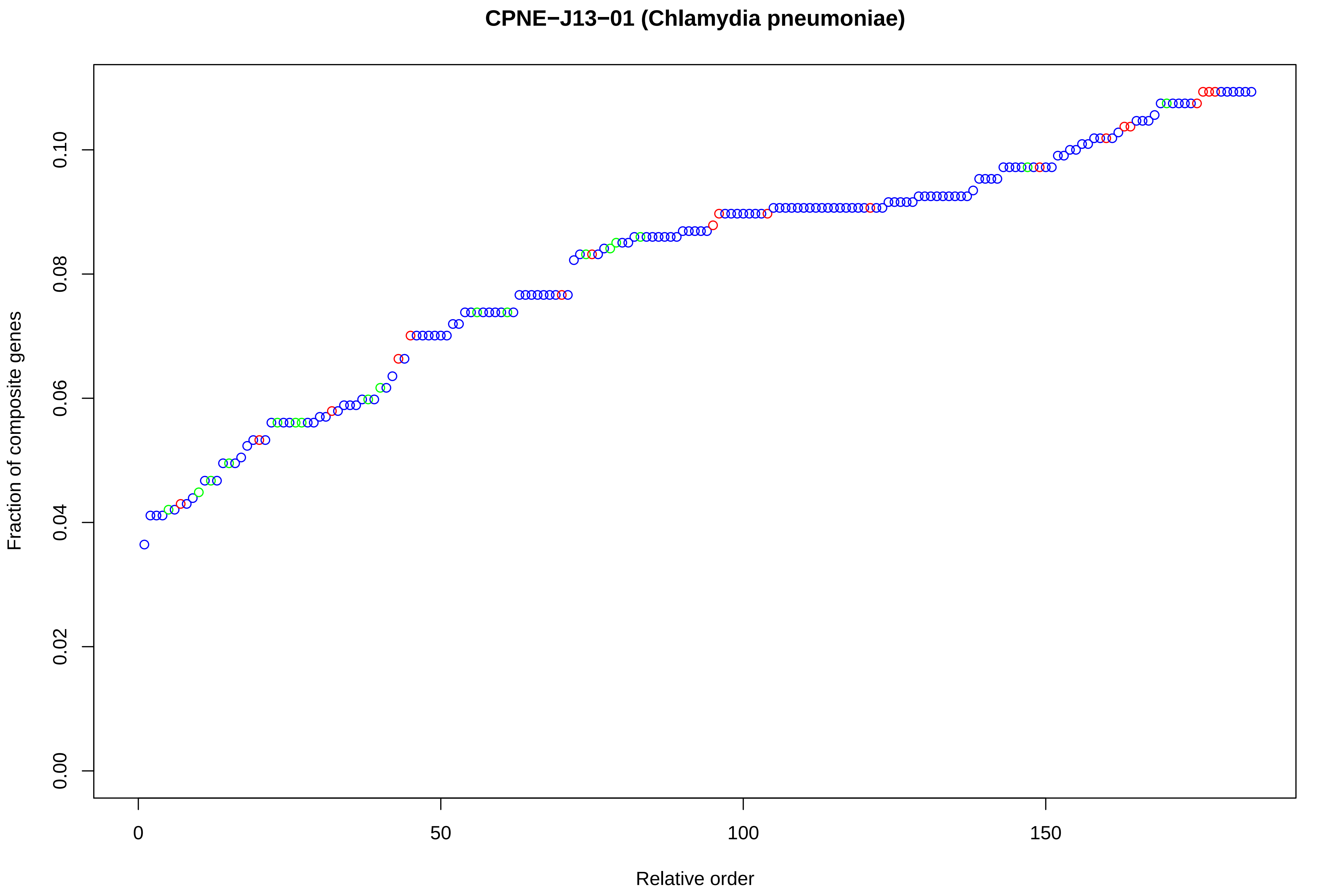

CPNE−J13−01 (Chlamydia pneumoniae)
Fraction of composite genes
0.00
0.02
0.04
0.06
0.08
0.10
0
50
100
150
Relative order

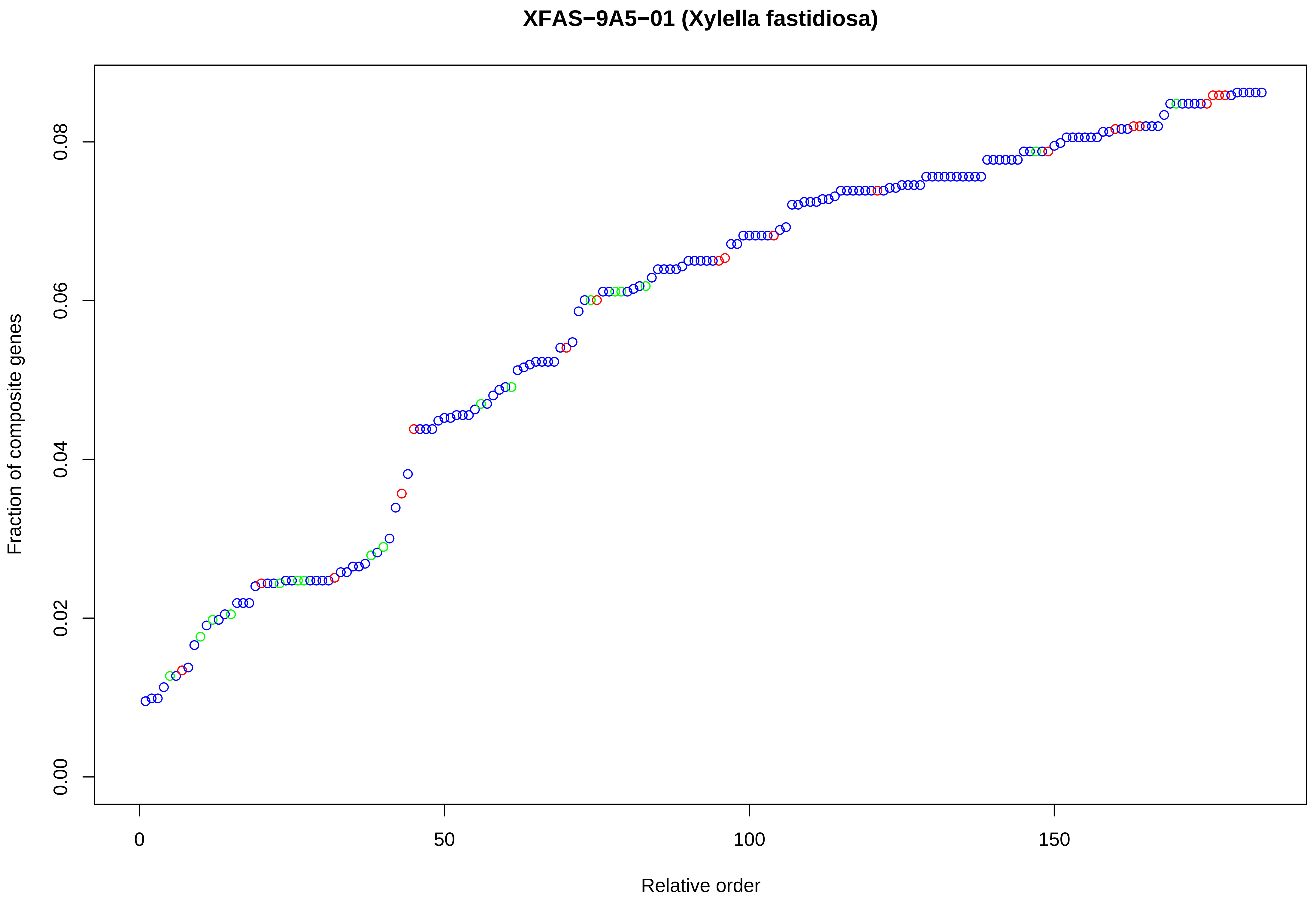

XFAS−9A5−01 (Xylella fastidiosa)
Fraction of composite genes
0.00
0.02
0.04
0.06
0.08
0
50
100
150
Relative order

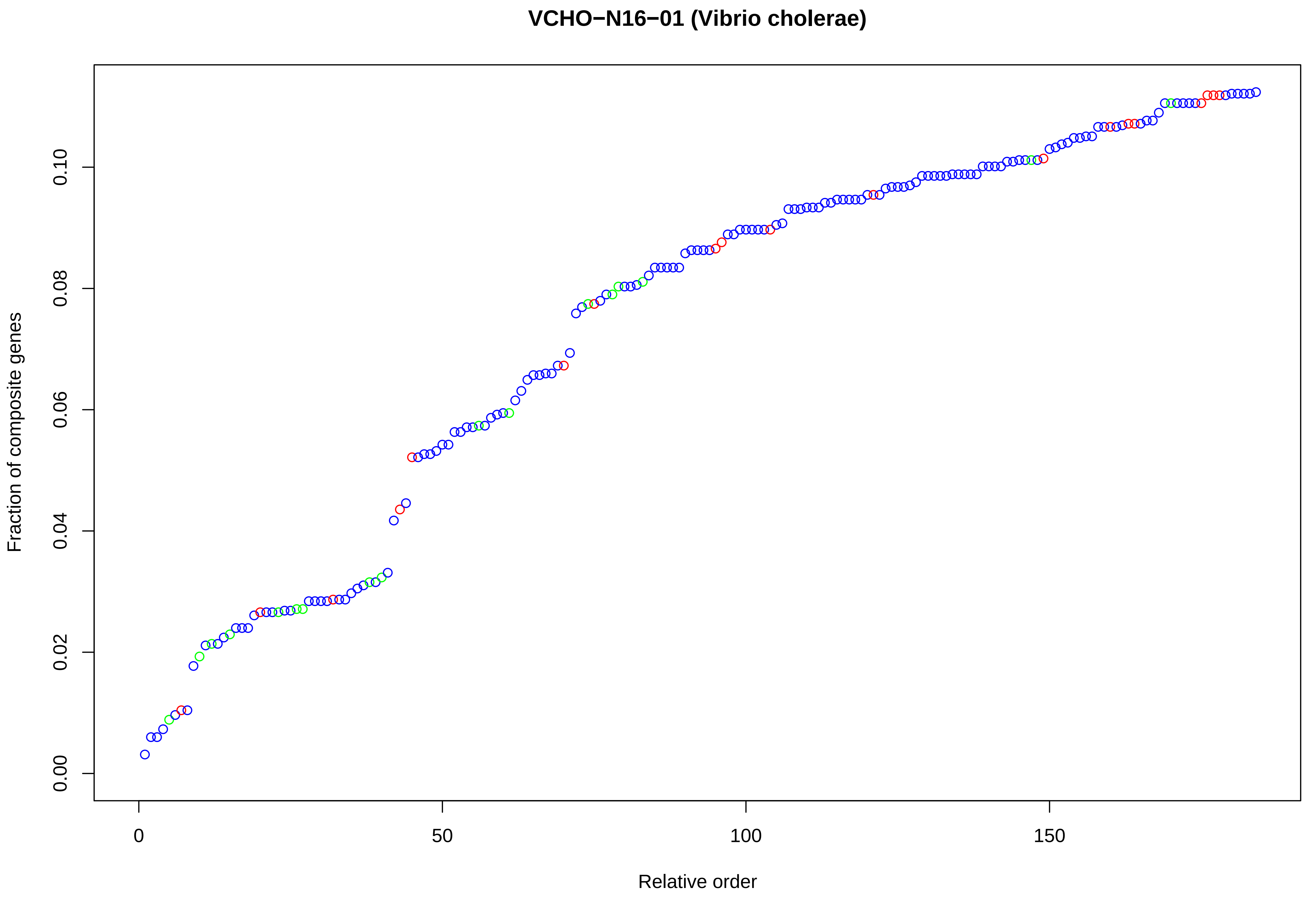

VCHO−N16−01 (Vibrio cholerae)
Fraction of composite genes
0.00
0.02
0.04
0.06
0.08
0.10
Relative order
0
50
100
150

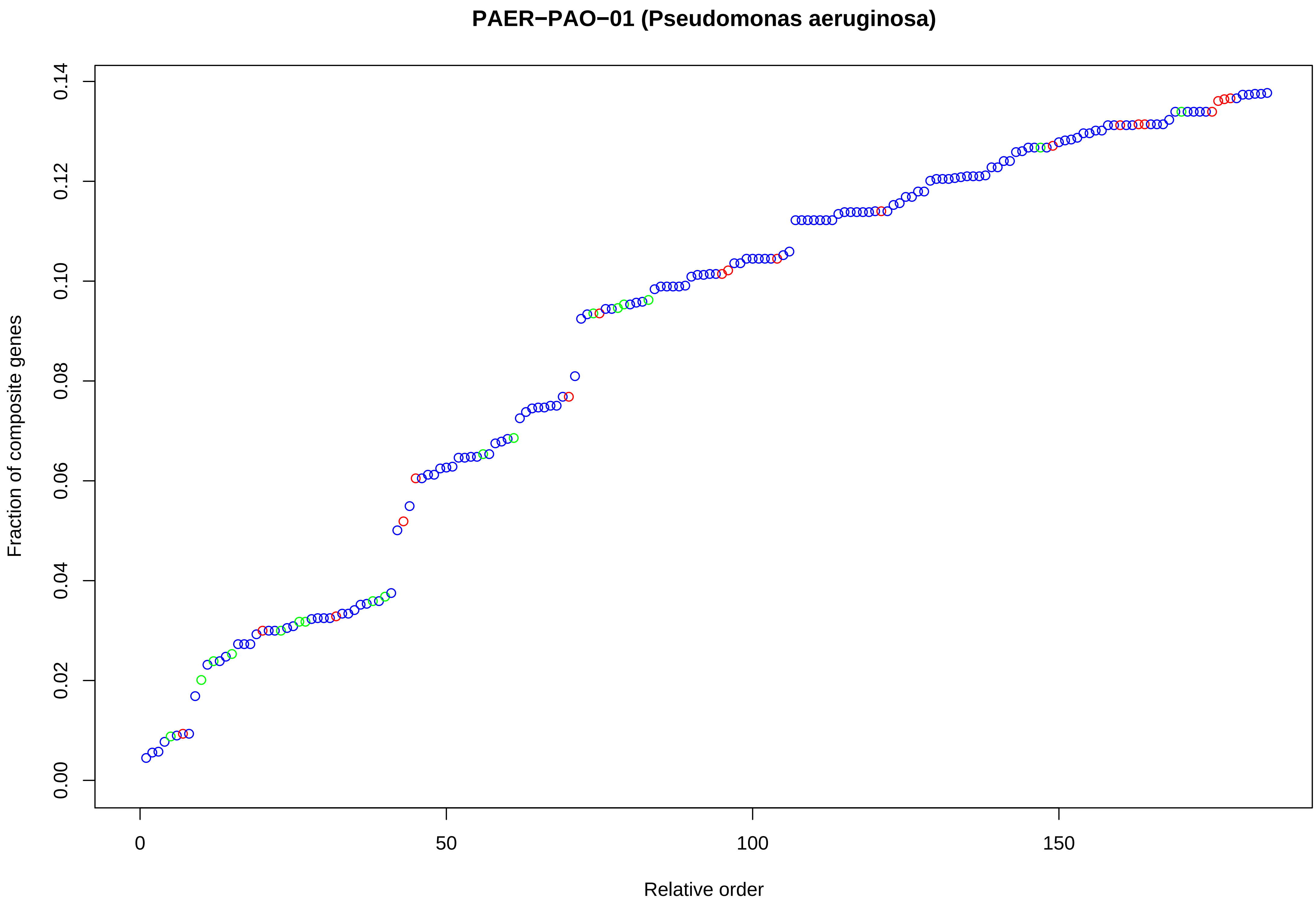
PAER−PAO−01 (Pseudomonas aeruginosa)
Fraction of composite genes
0.00
0.02
0.04
0.06
0.08
0.10
0.12
0.14
0
50
100
150
Relative order

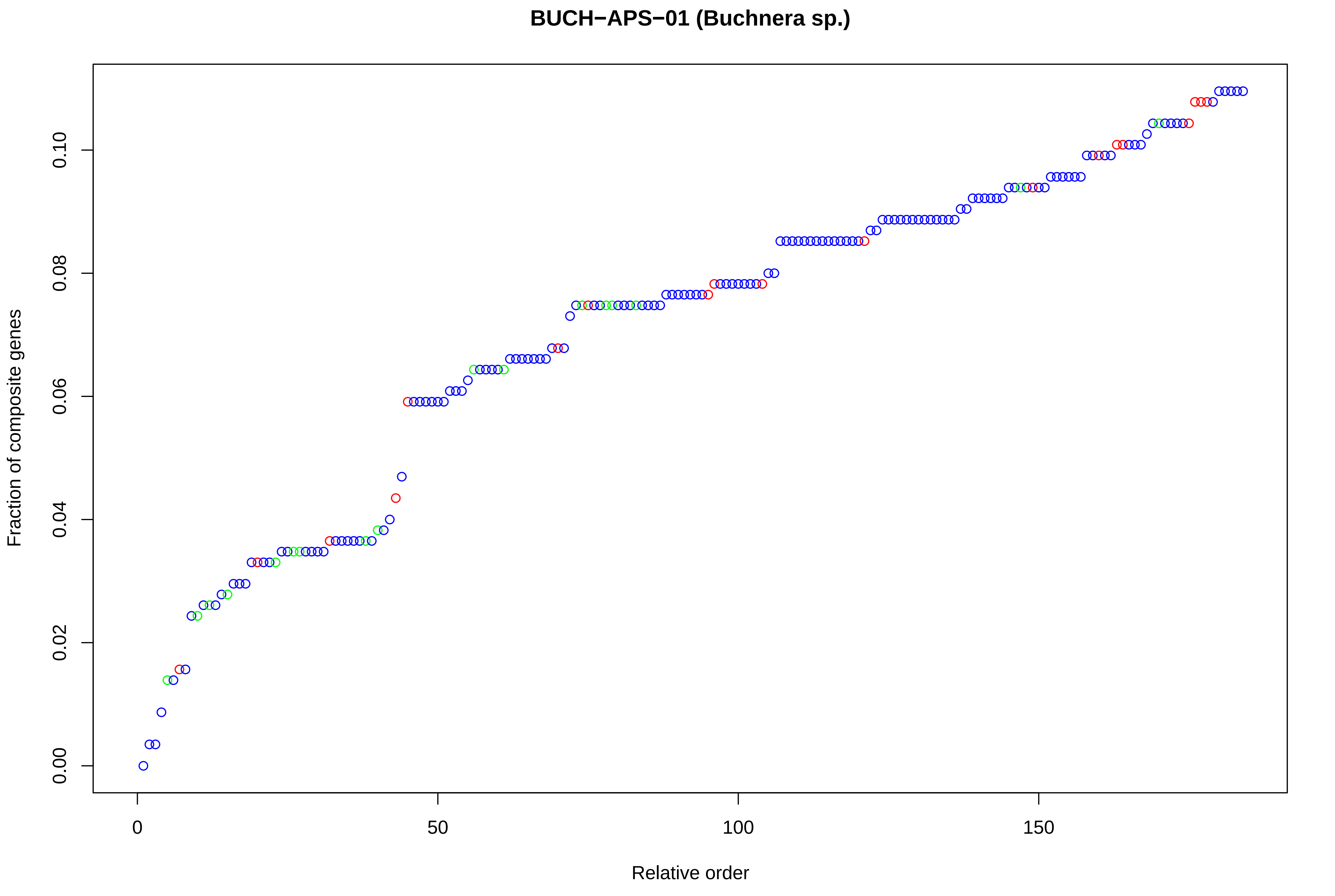

BUCH−APS−01 (Buchnera sp.)
Fraction of composite genes
0.00
0.02
0.04
0.06
0.08
0.10
0
50
100
150
Relative order

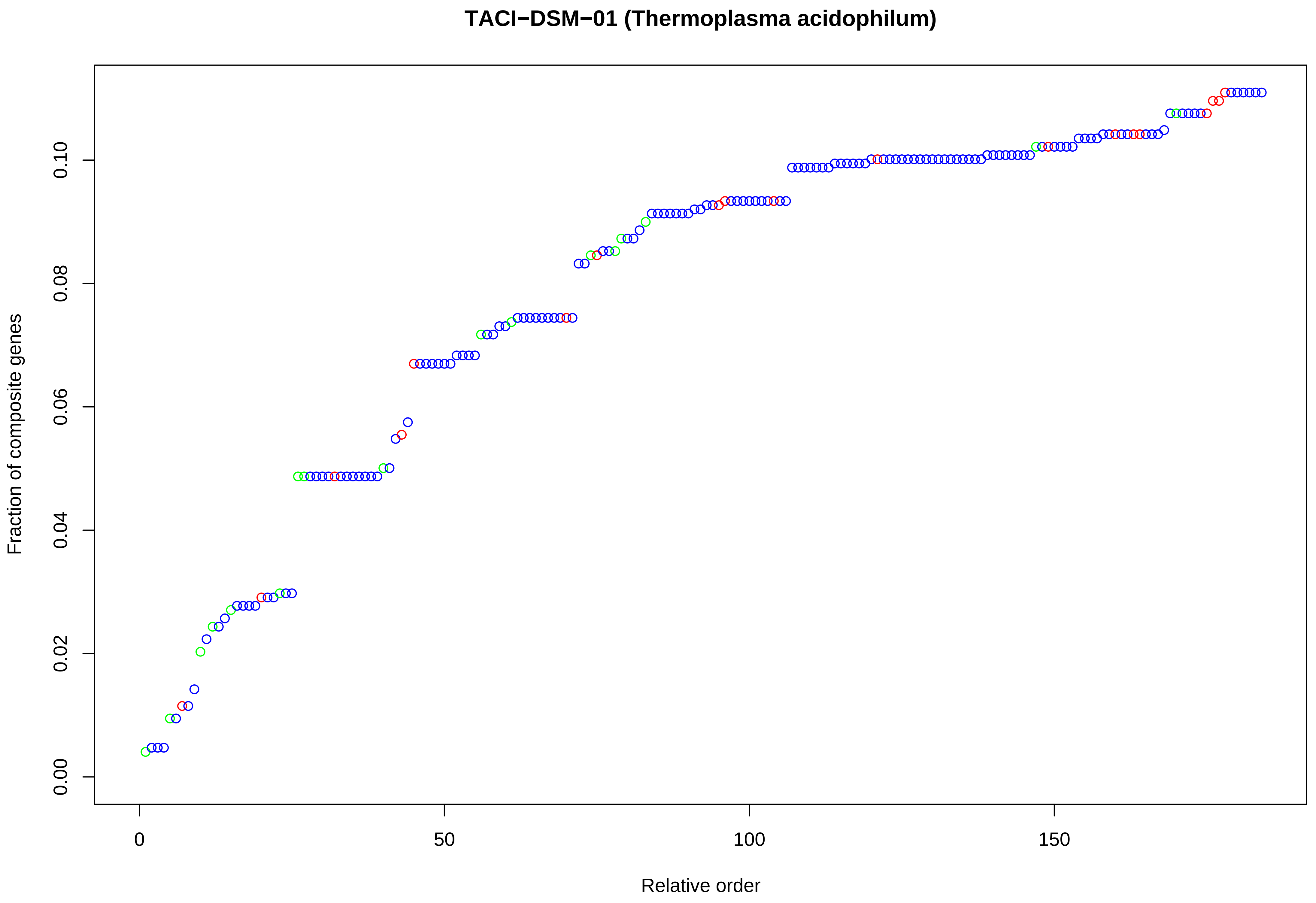
TACI−DSM−01 (Thermoplasma acidophilum)
Fraction of composite genes
Relative order
0.00
0.02
0.04
0.06
0.08
0.10
0
50
100
150

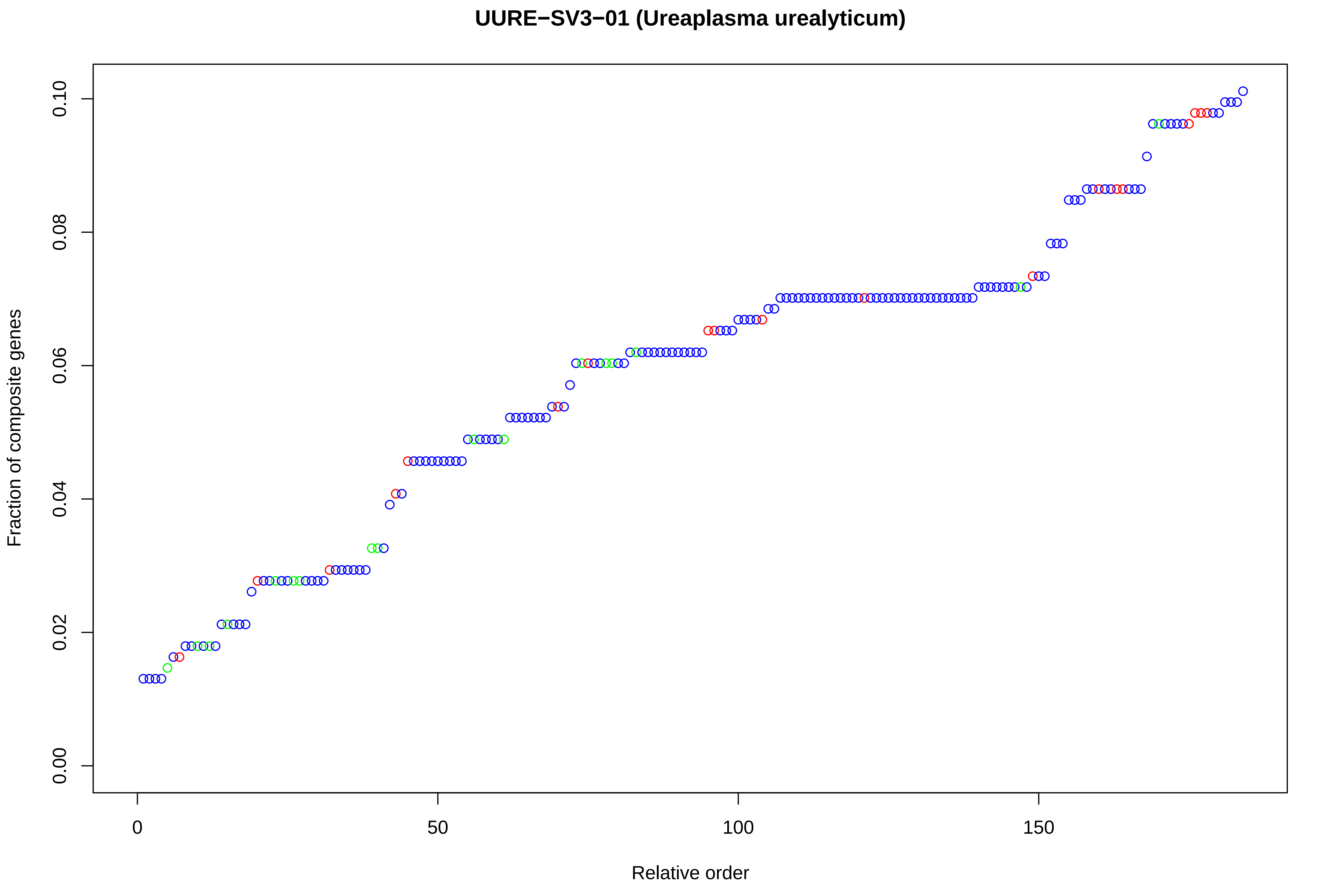
UURE−SV3−01 (Ureaplasma urealyticum)
Fraction of composite genes
Relative order
0.00
0.02
0.04
0.06
0.08
0.10
0
50
100
150

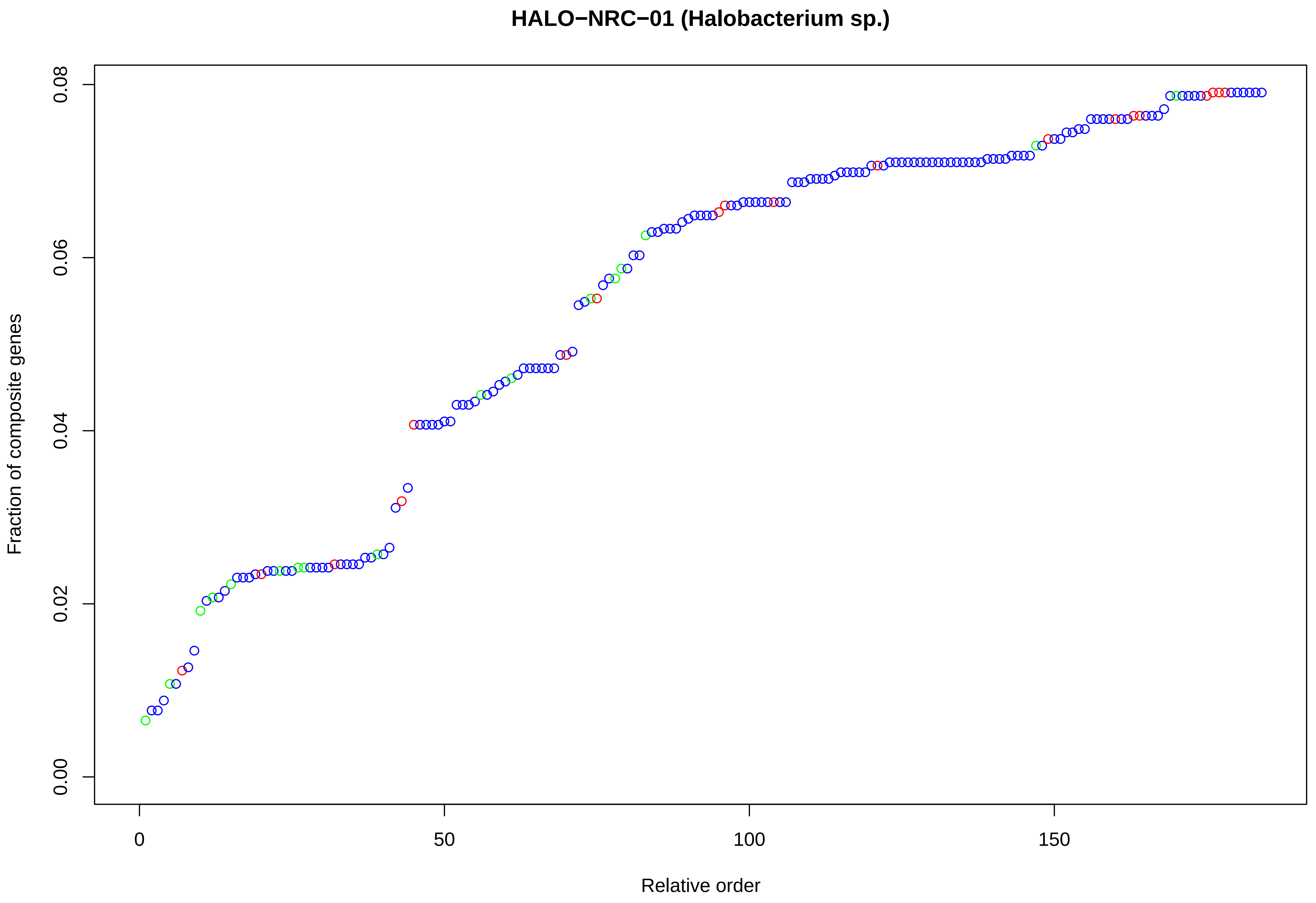

HALO−NRC−01 (Halobacterium sp.)
Fraction of composite genes
0.00
0.02
0.04
0.06
0.08
0
50
100
150
Relative order

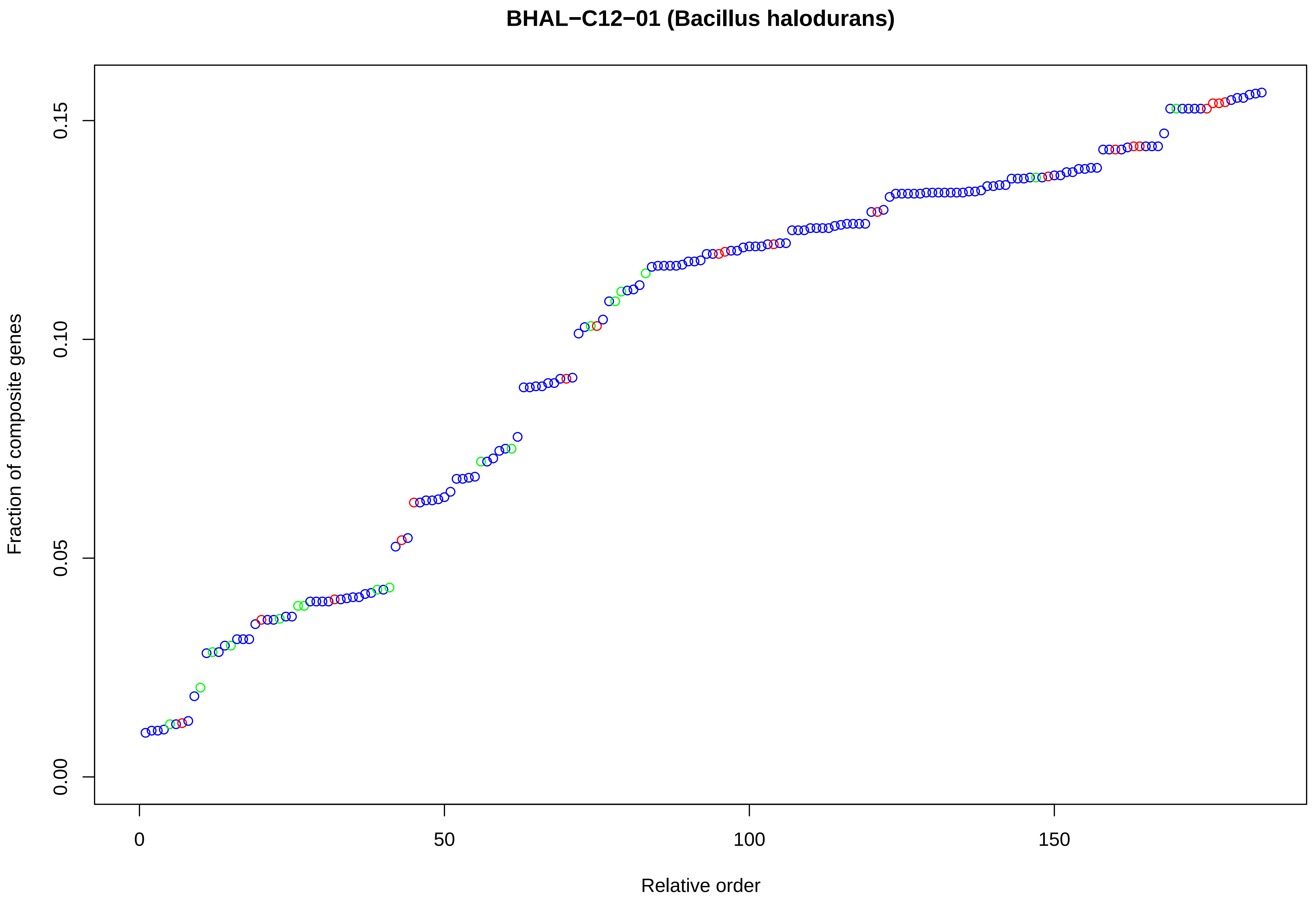

BHAL−C12−01 (Bacillus halodurans)
Fraction of composite genes
0.00
0.05
0.10
0.15
0
50
100
150
Relative order

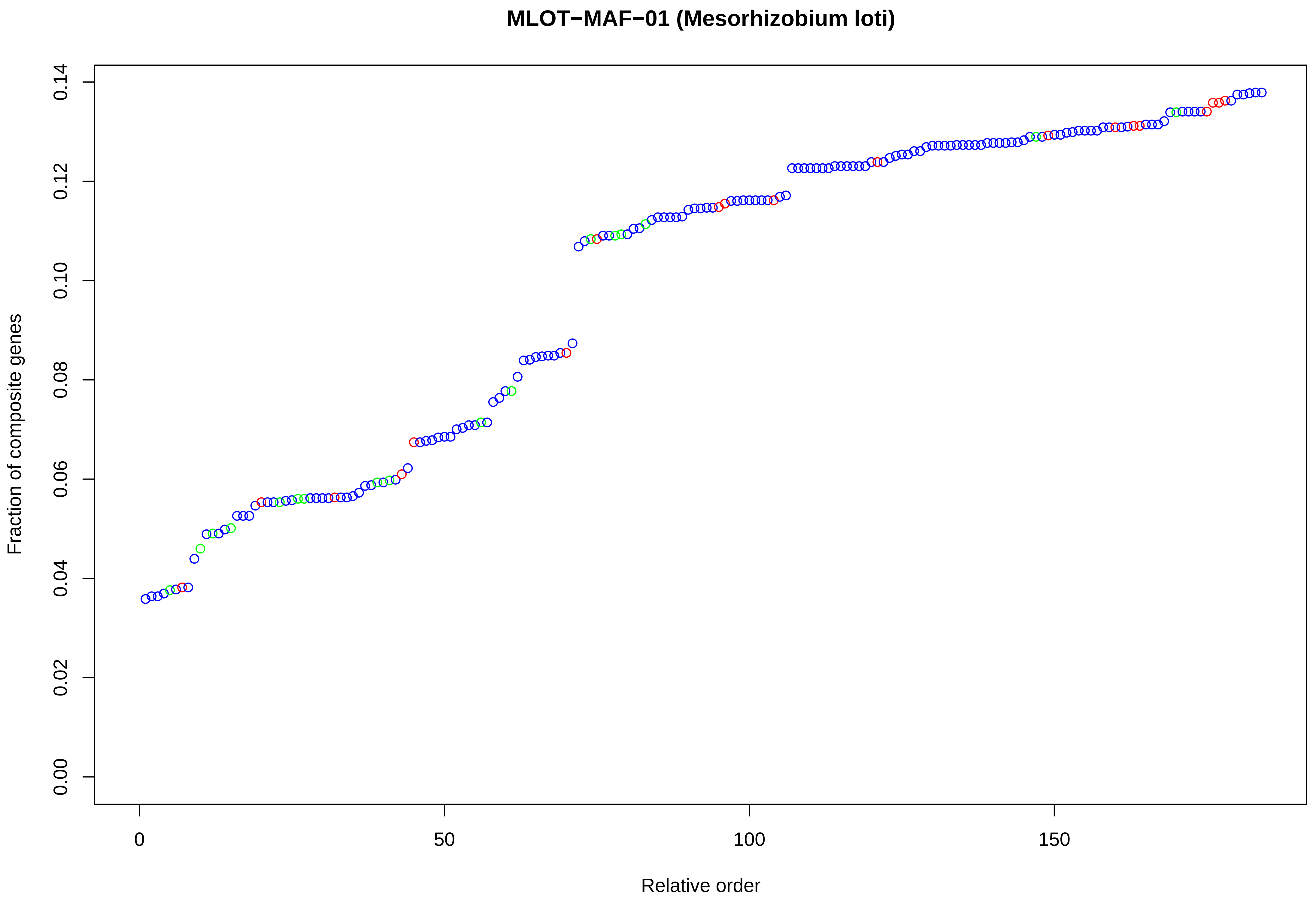
MLOT−MAF−01 (Mesorhizobium loti)
Fraction of composite genes
Relative order
0.00
0.02
0.04
0.06
0.08
0.10
0.12
0.14
0
50
100
150

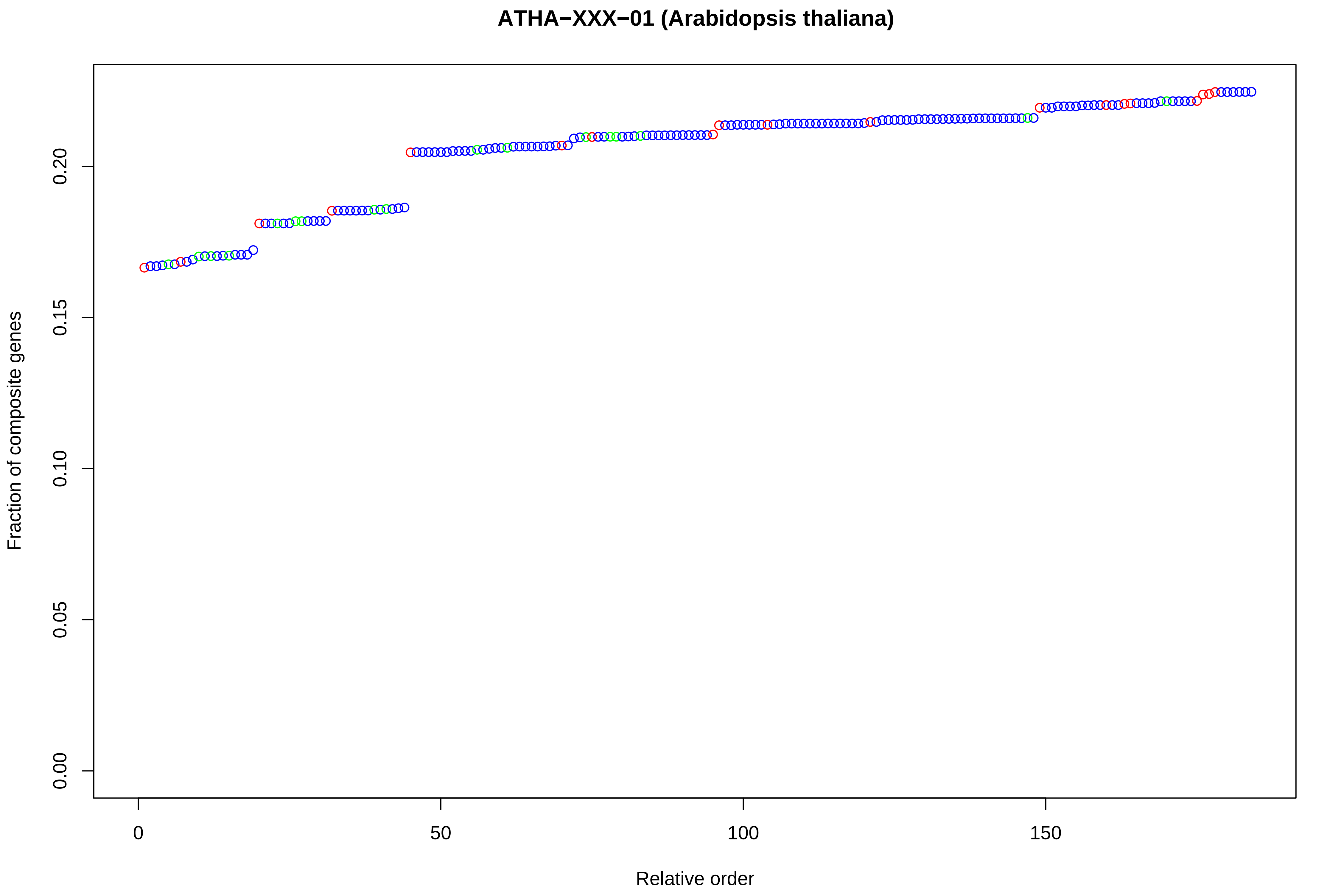
ATHA−XXX−01 (Arabidopsis thaliana)
Fraction of composite genes
0.00
0.05
0.10
0.15
0.20
0
50
100
150
Relative order

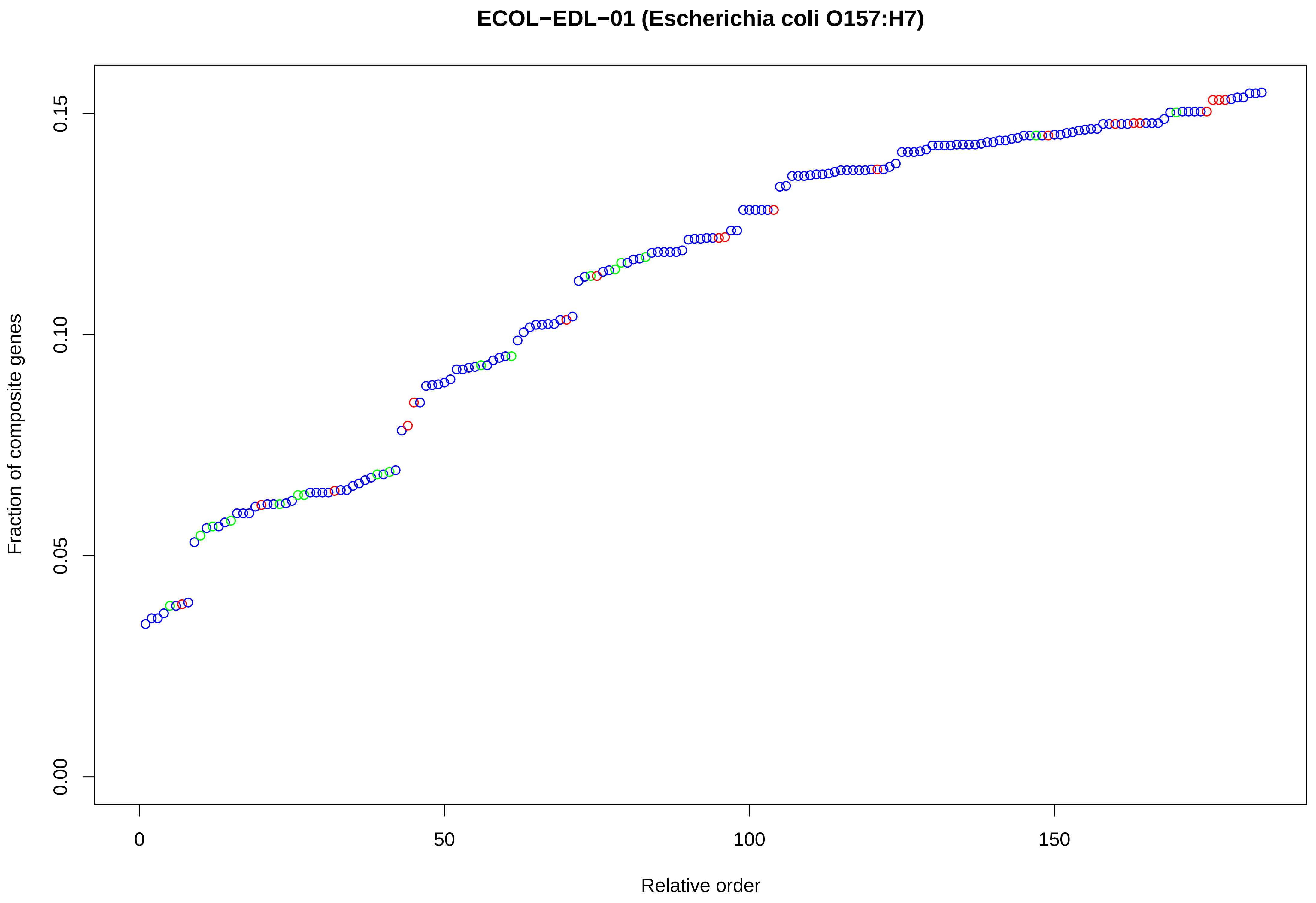
ECOL−EDL−01 (Escherichia coli O157:H7)
Fraction of composite genes
0.00
0.05
0.10
0.15
0
50
100
150
Relative order

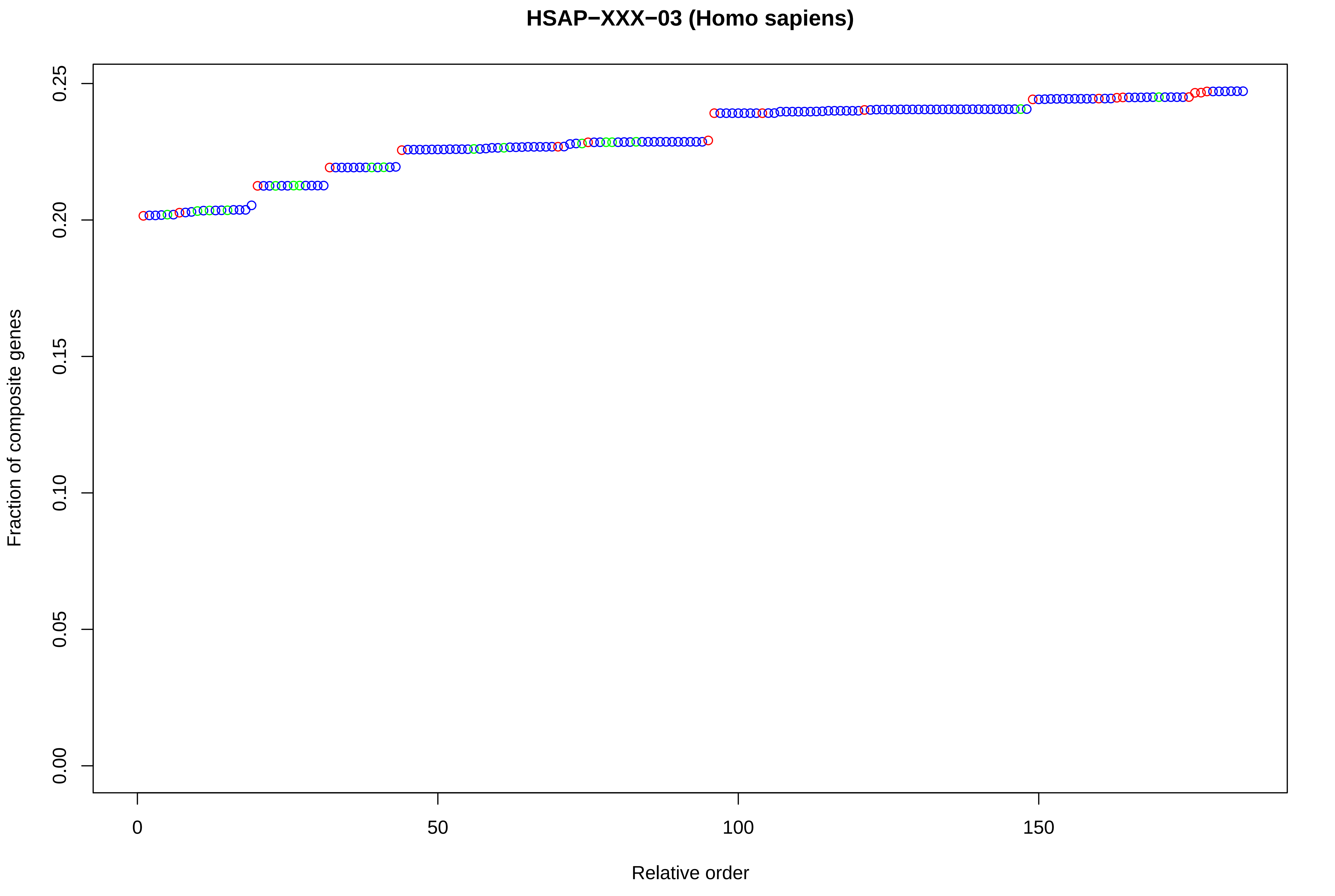
HSAP−XXX−03 (Homo sapiens)
Fraction of composite genes
0.00
0.05
0.10
0.15
0.20
0.25
Relative order
0
50
100
150

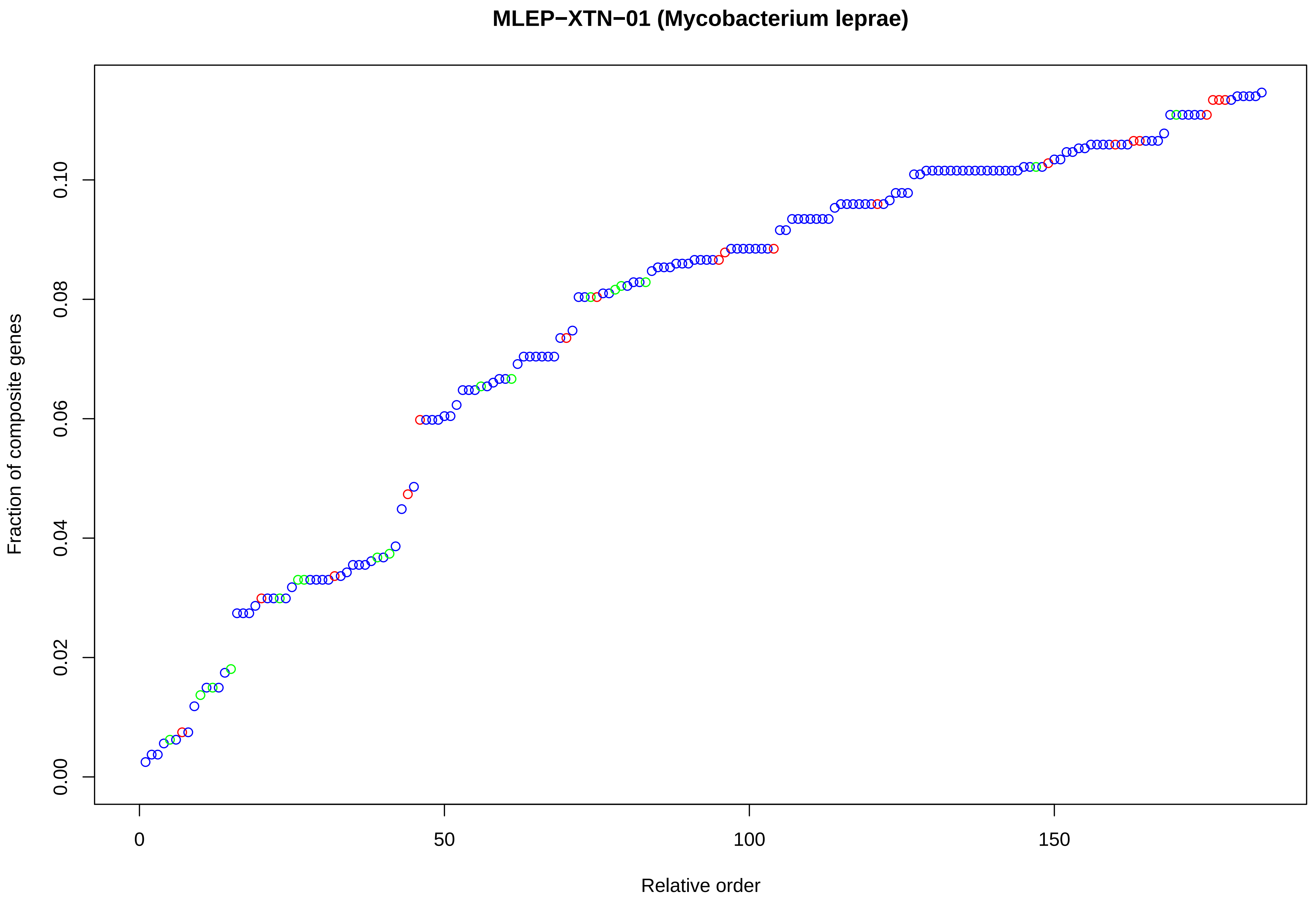

MLEP−XTN−01 (Mycobacterium leprae)
Fraction of composite genes
0.00
0.02
0.04
0.06
0.08
0.10
Relative order
0
50
100
150

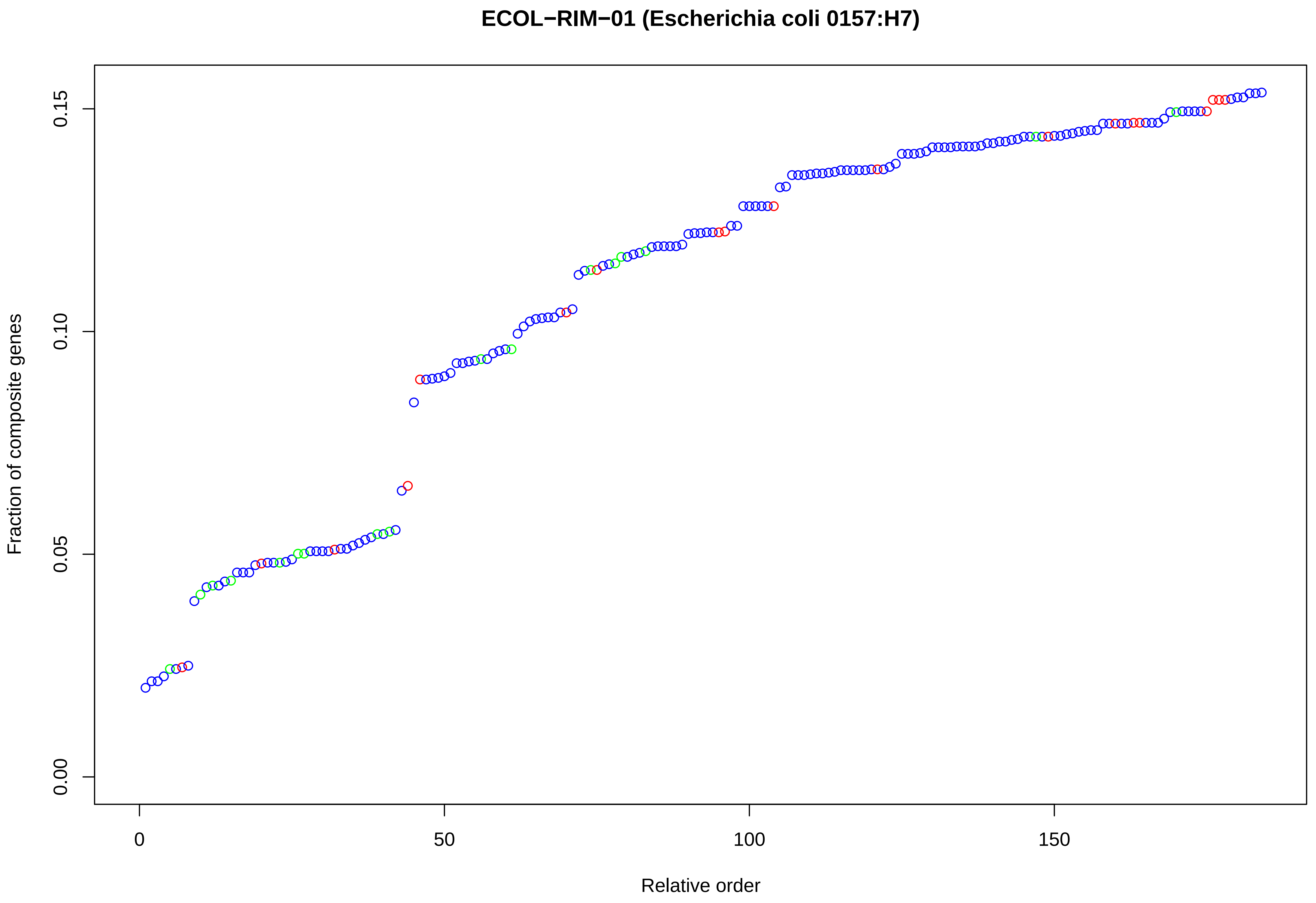
ECOL−RIM−01 (Escherichia coli 0157:H7)
Fraction of composite genes
0.00
0.05
0.10
0.15
Relative order
0
50
100
150

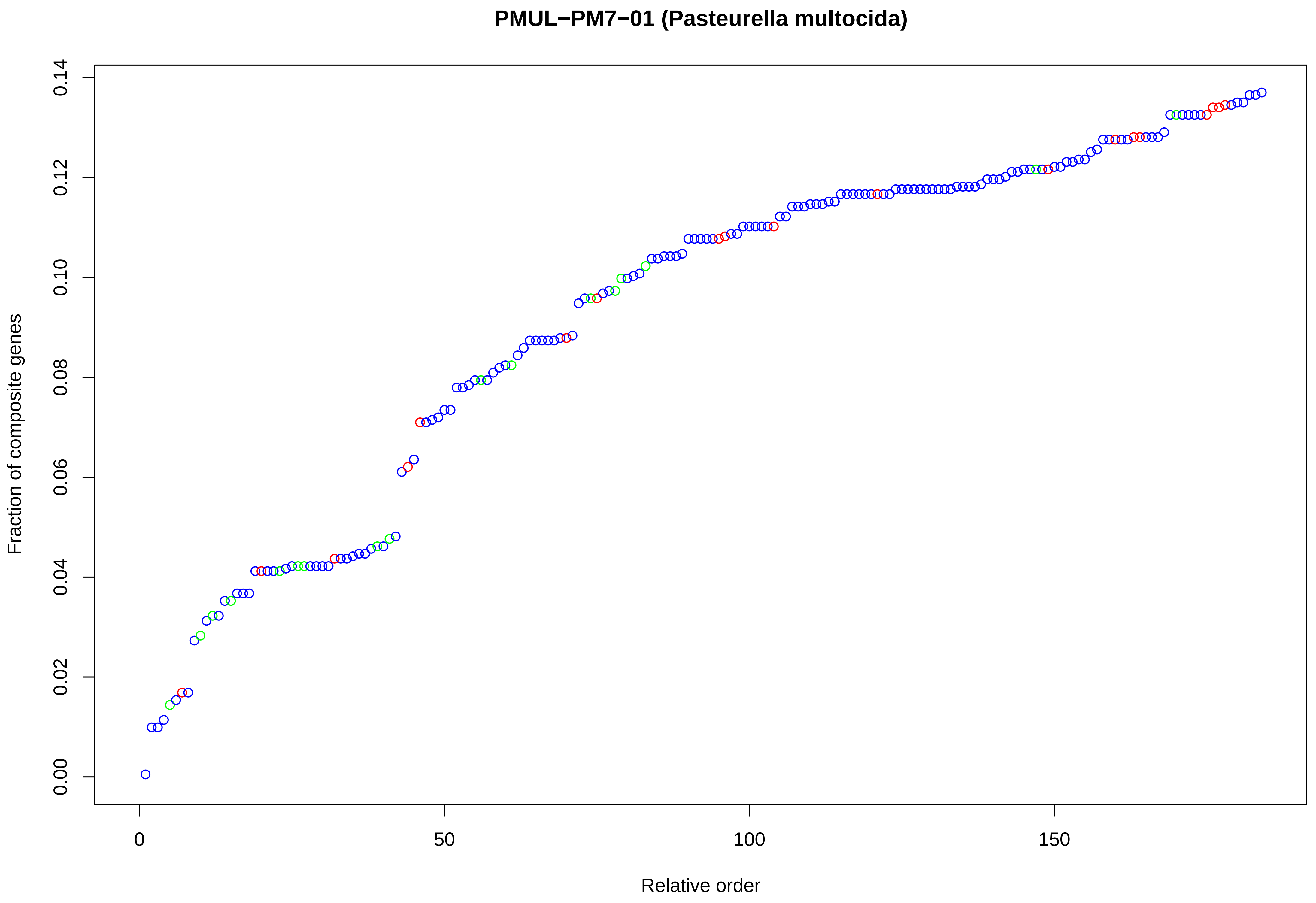
PMUL−PM7−01 (Pasteurella multocida)
Fraction of composite genes
0.00
0.02
0.04
0.06
0.08
0.10
0.12
0.14
Relative order
0
50
100
150

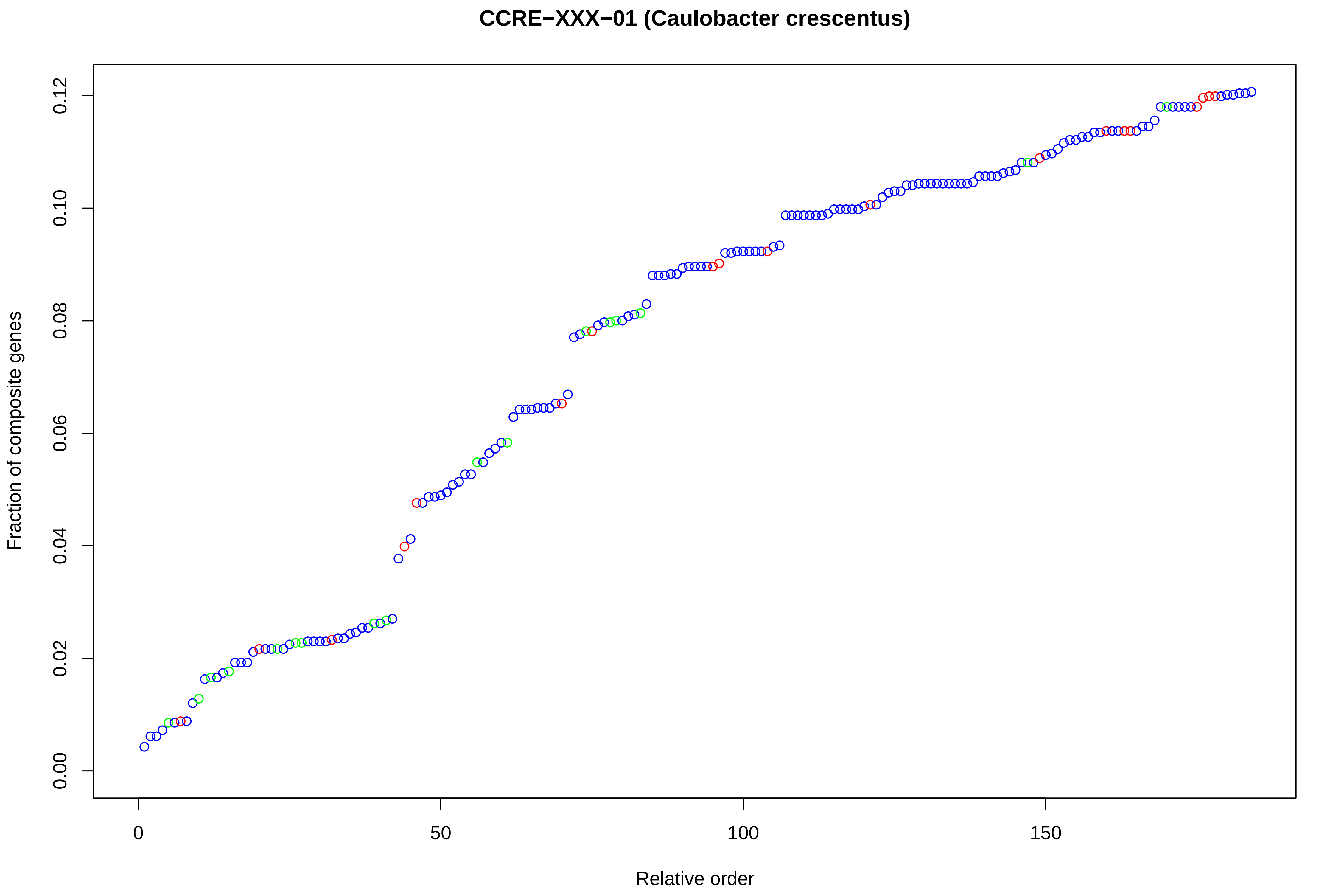

CCRE−XXX−01 (Caulobacter crescentus)
Fraction of composite genes
Relative order
0.00
0.02
0.04
0.06
0.08
0.10
0.12
0
50
100
150

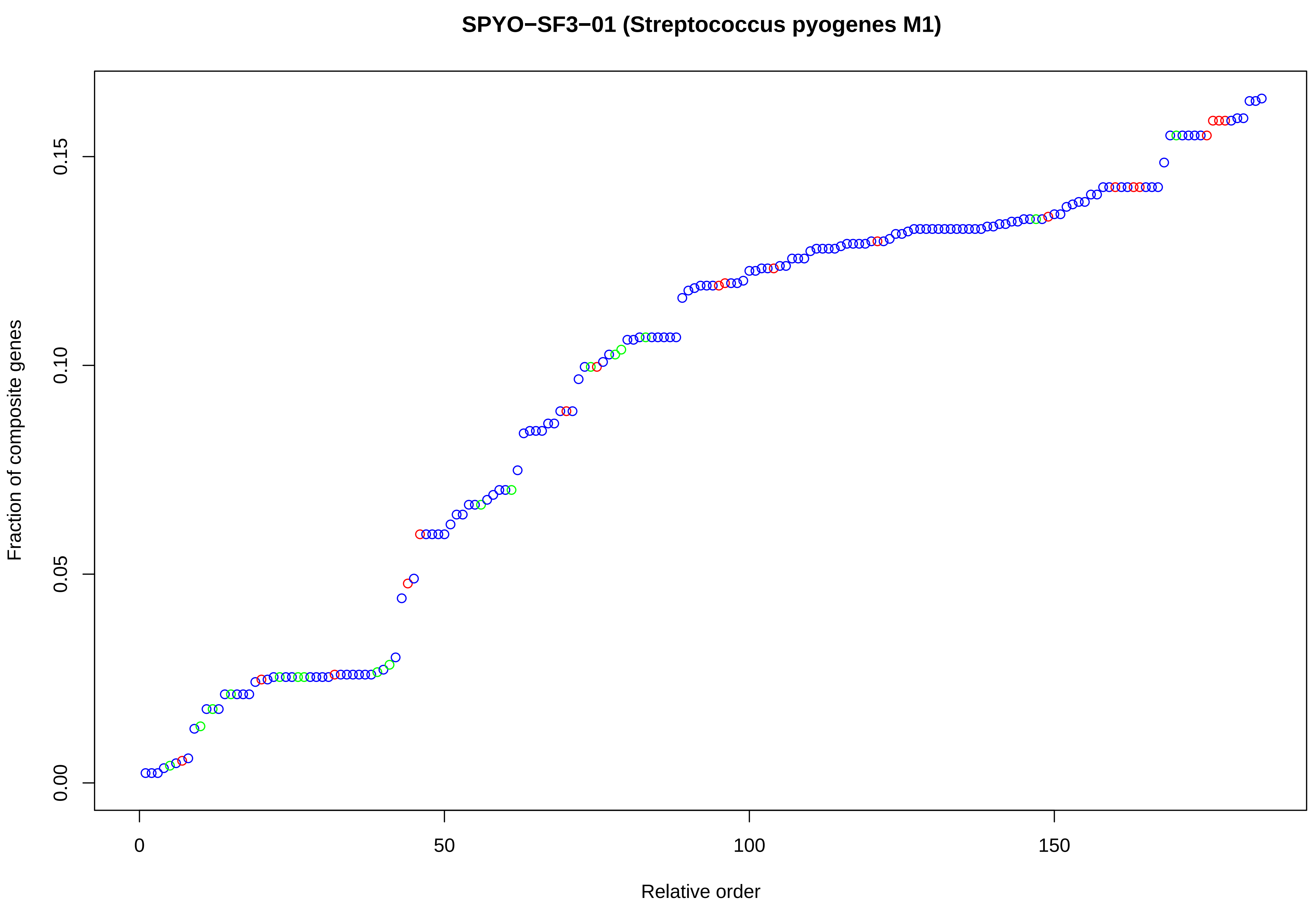
SPYO−SF3−01 (Streptococcus pyogenes M1)
Fraction of composite genes
0.00
0.05
0.10
0.15
Relative order
0
50
100
150

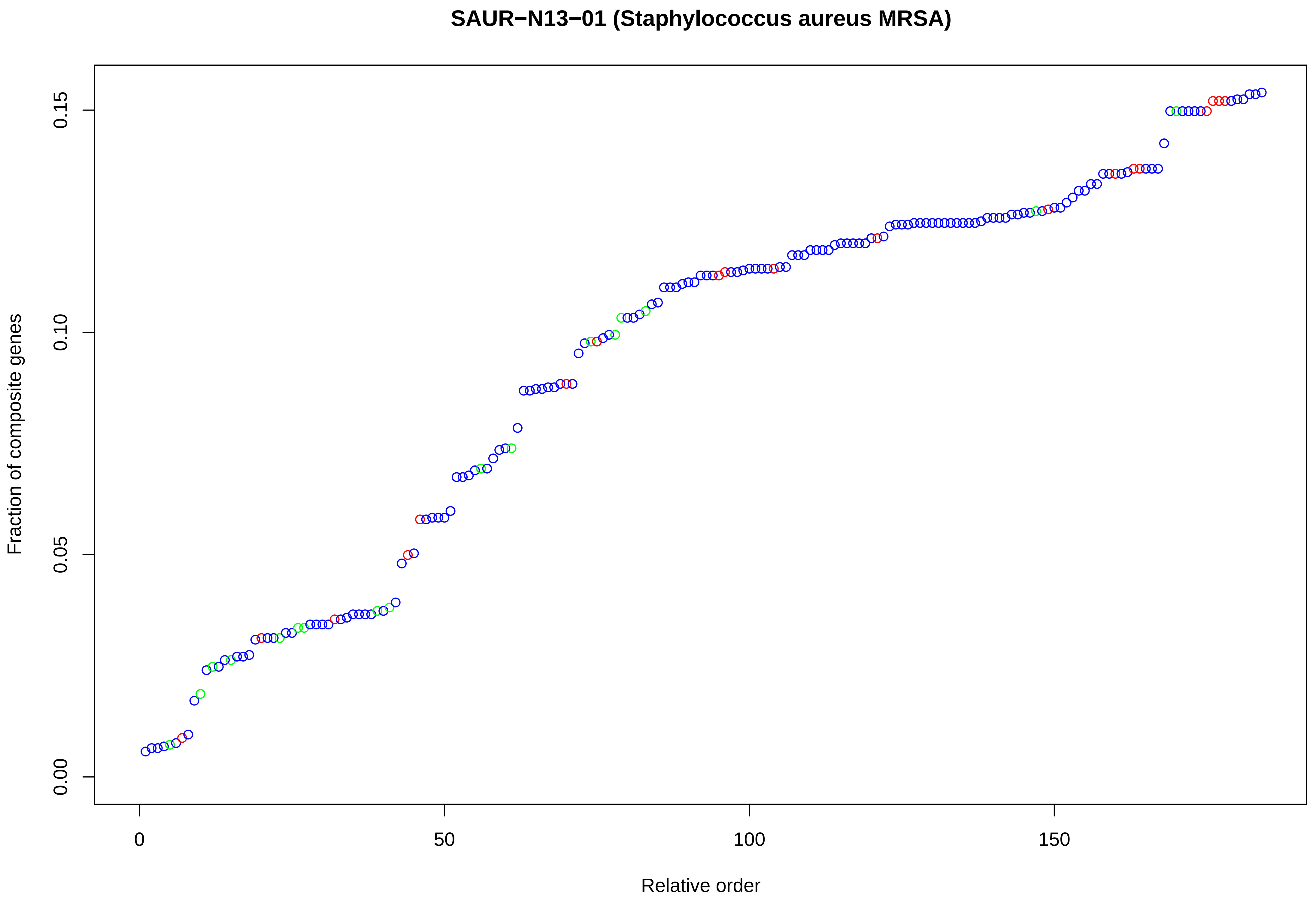

SAUR−N13−01 (Staphylococcus aureus MRSA)
Fraction of composite genes
0.00
0.05
0.10
0.15
0
50
100
150
Relative order

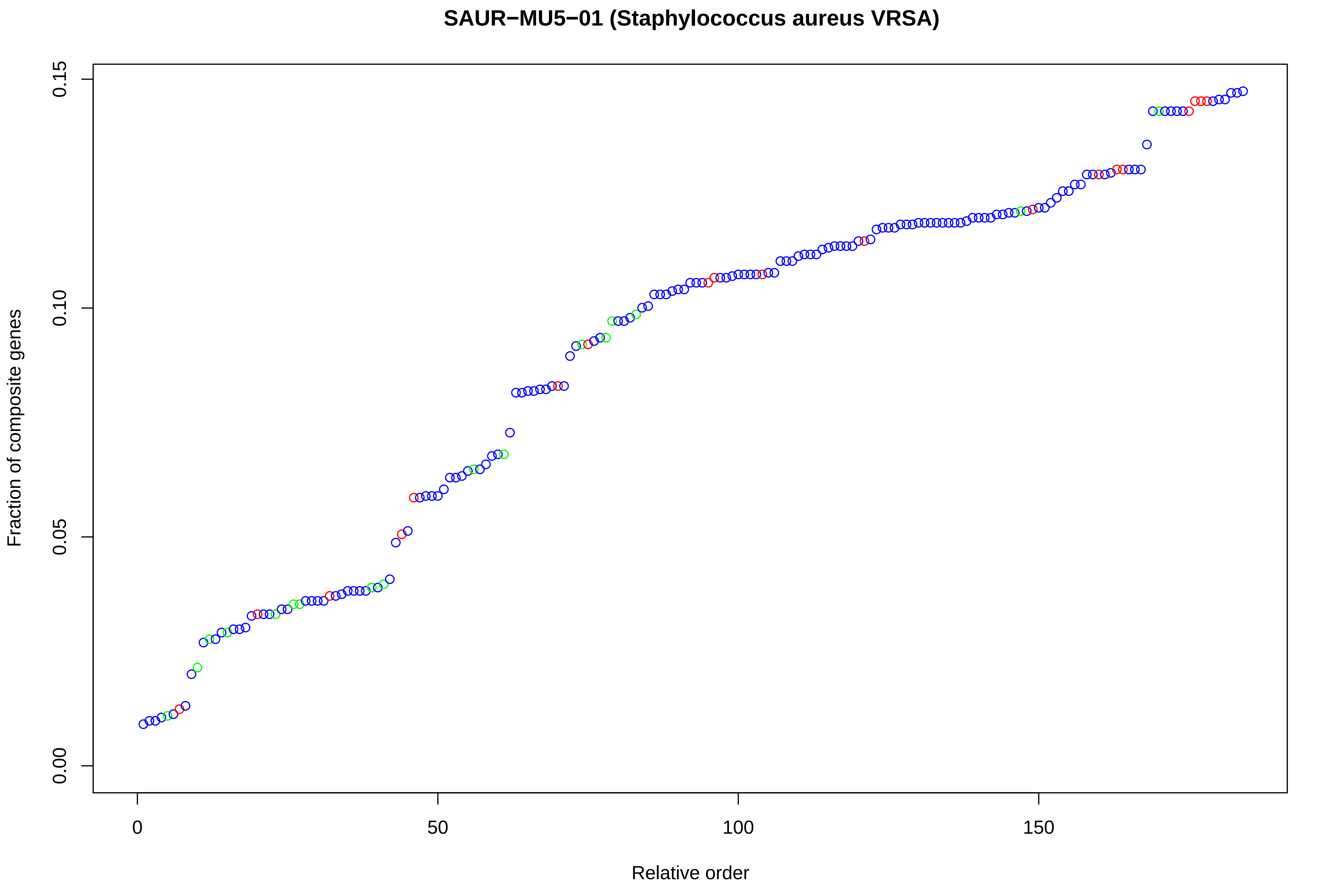
SAUR−MU5−01 (Staphylococcus aureus VRSA)
Fraction of composite genes
Relative order
0.00
0.05
0.10
0.15
0
50
100
150

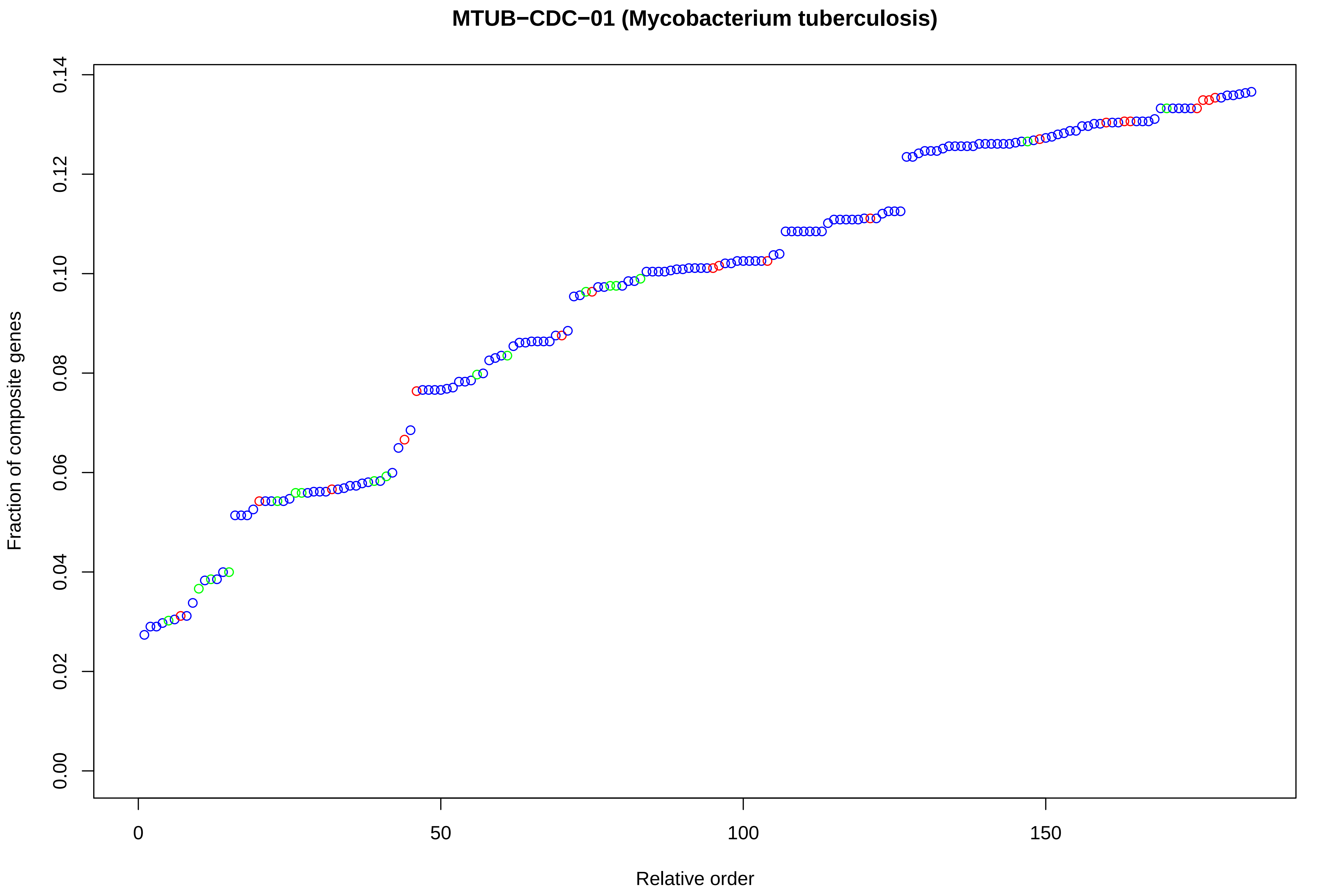

MTUB−CDC−01 (Mycobacterium tuberculosis)
Fraction of composite genes
Relative order
0.00
0.02
0.04
0.06
0.08
0.10
0.12
0.14
0
50
100
150

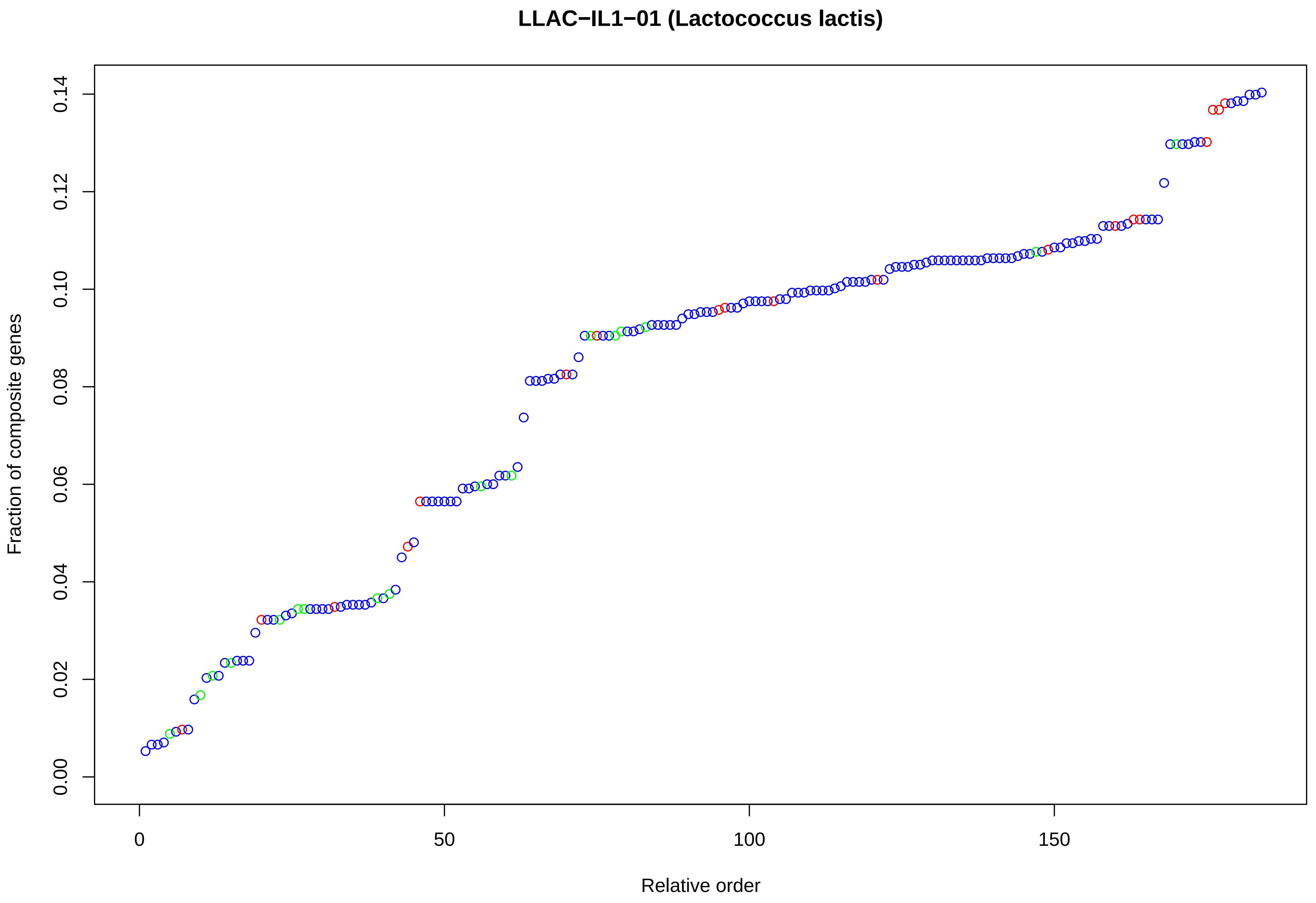
LLAC−IL1−01 (Lactococcus lactis)
Fraction of composite genes
0.00
0.02
0.04
0.06
0.08
0.10
0.12
0.14
Relative order
0
50
100
150

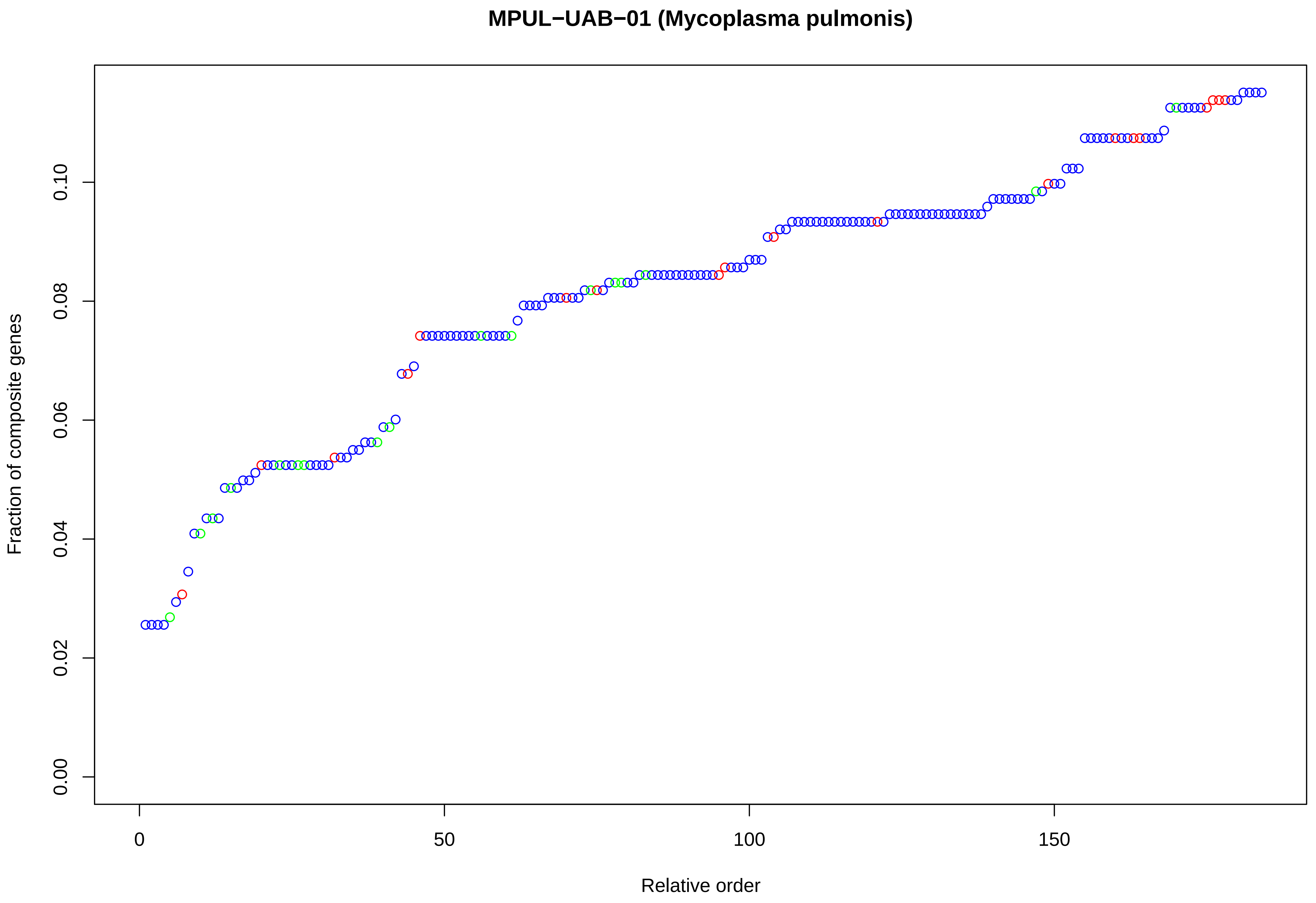

MPUL−UAB−01 (Mycoplasma pulmonis)
Fraction of composite genes
0.00
0.02
0.04
0.06
0.08
0.10
Relative order
0
50
100
150

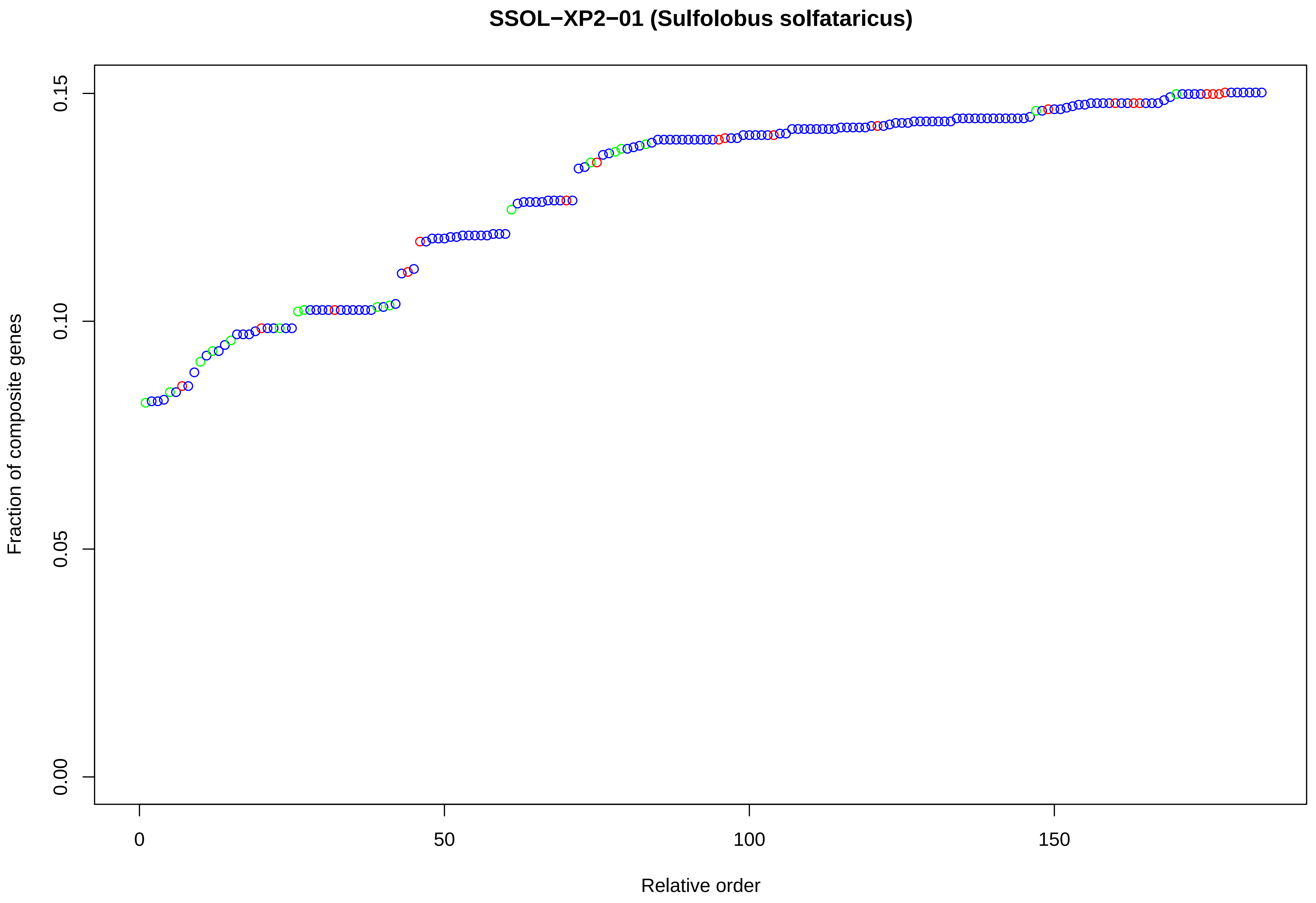

SSOL−XP2−01 (Sulfolobus solfataricus)
Fraction of composite genes
0.00
0.05
0.10
0.15
0
50
100
150
Relative order

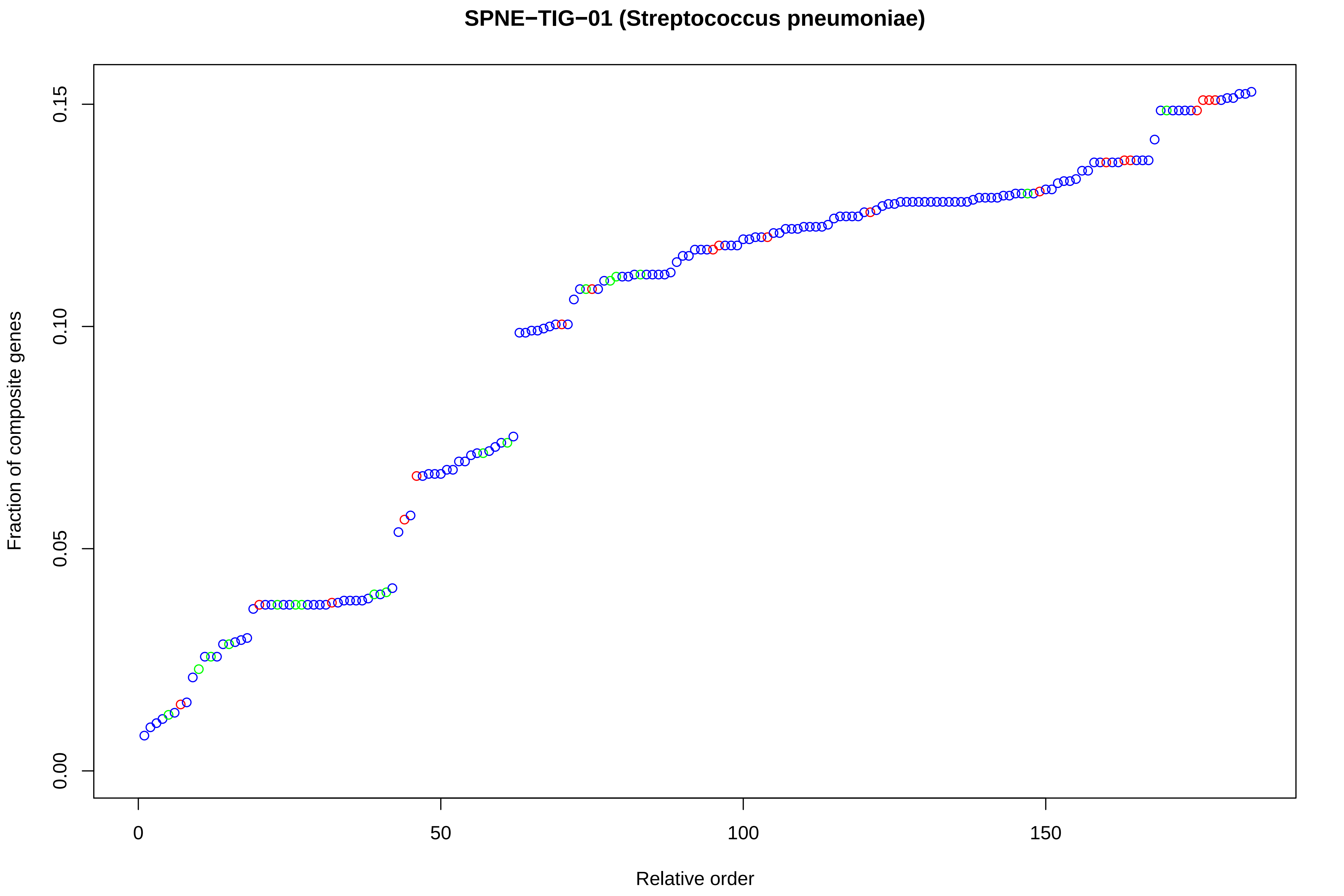
SPNE−TIG−01 (Streptococcus pneumoniae)
Fraction of composite genes
0.00
0.05
0.10
0.15
0
50
100
150
Relative order

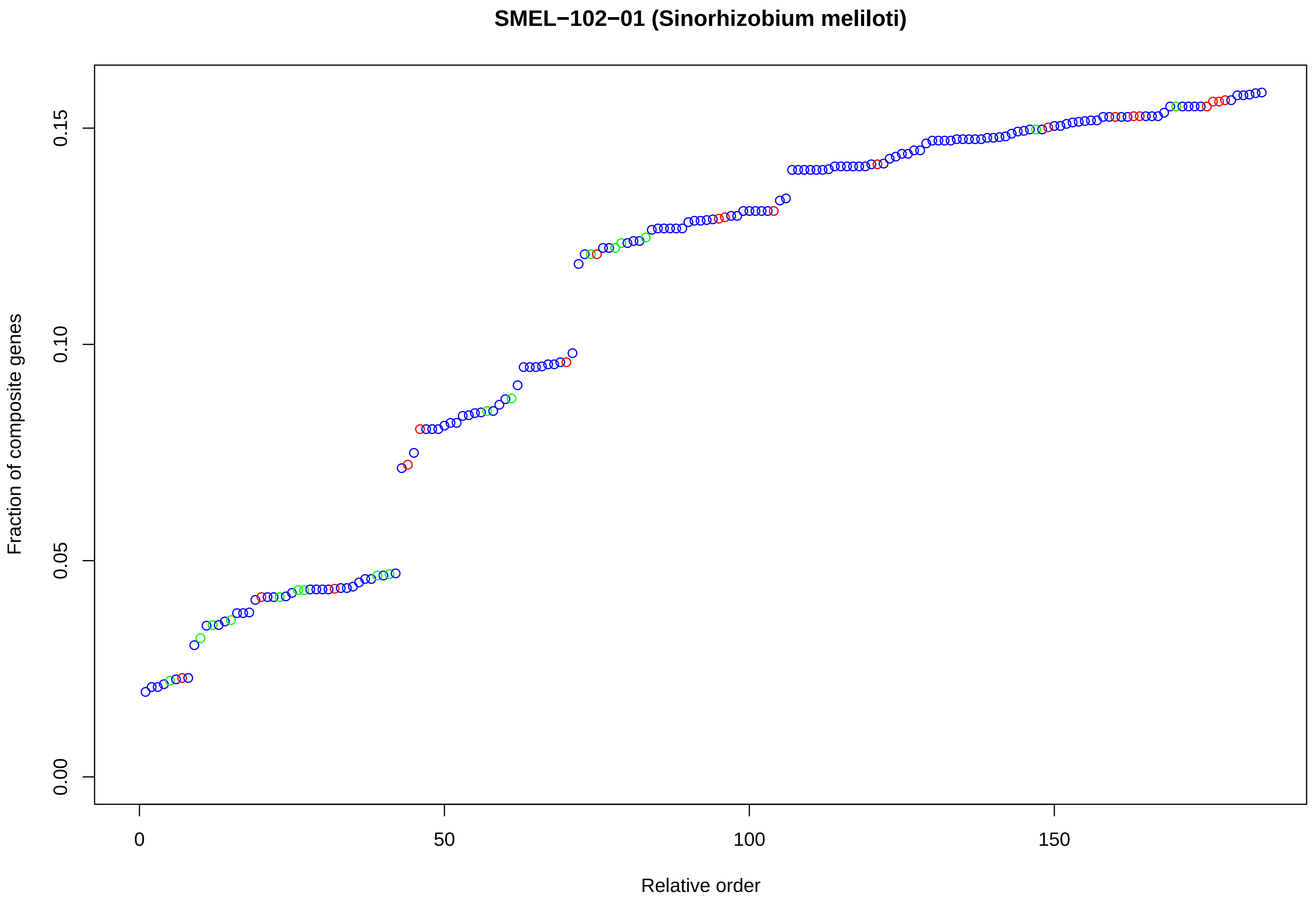
SMEL−102−01 (Sinorhizobium meliloti)
Fraction of composite genes
Relative order
0.00
0.05
0.10
0.15
0
50
100
150

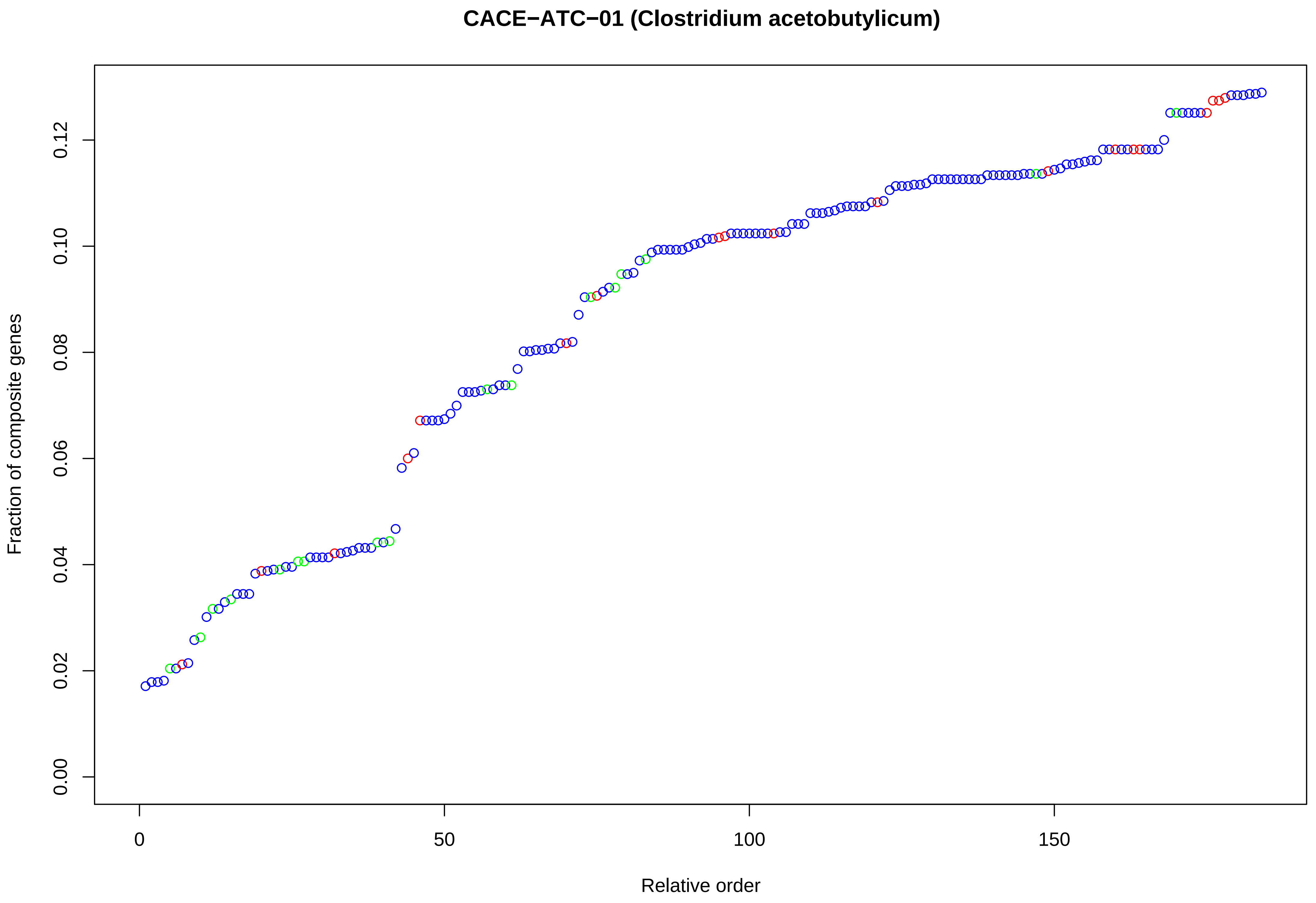
CACE−ATC−01 (Clostridium acetobutylicum)
Fraction of composite genes
0.00
0.02
0.04
0.06
0.08
0.10
0.12
Relative order
0
50
100
150

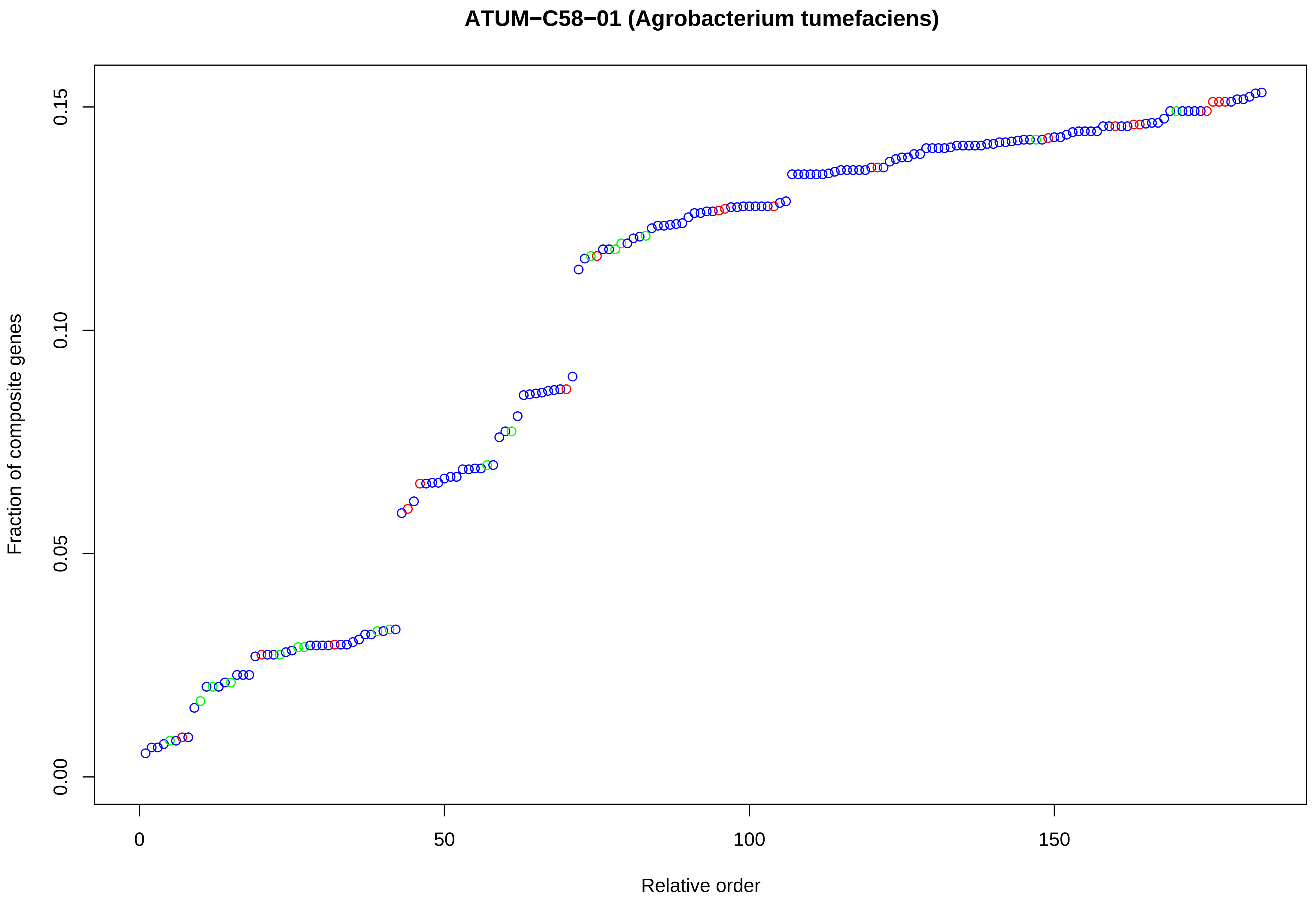

ATUM−C58−01 (Agrobacterium tumefaciens)
Fraction of composite genes
0.00
0.05
0.10
0.15
0
50
100
150
Relative order

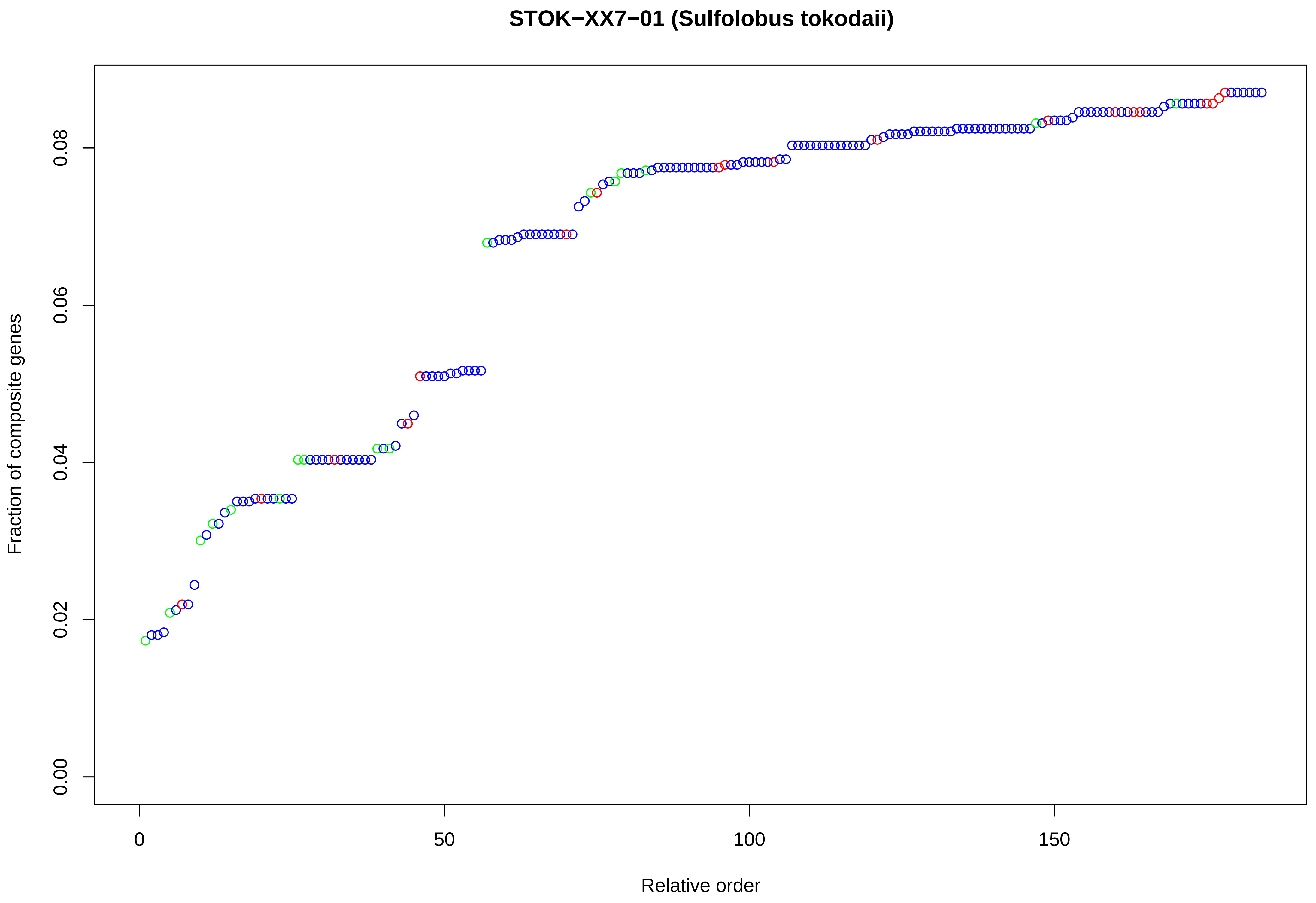

STOK−XX7−01 (Sulfolobus tokodaii)
Fraction of composite genes
Relative order
0.00
0.02
0.04
0.06
0.08
0
50
100
150

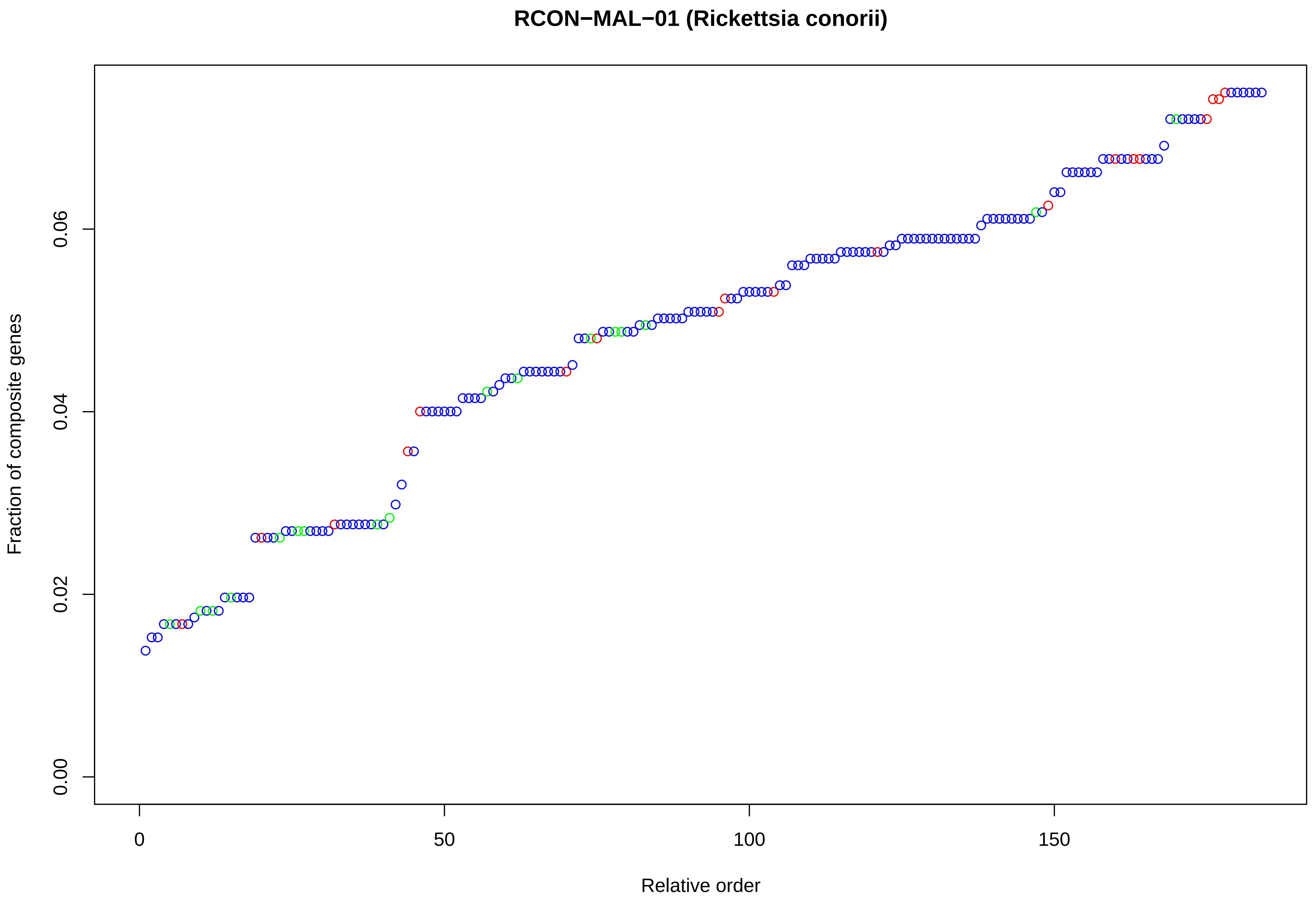

RCON−MAL−01 (Rickettsia conorii)
Fraction of composite genes
0.00
0.02
0.04
0.06
Relative order
0
50
100
150

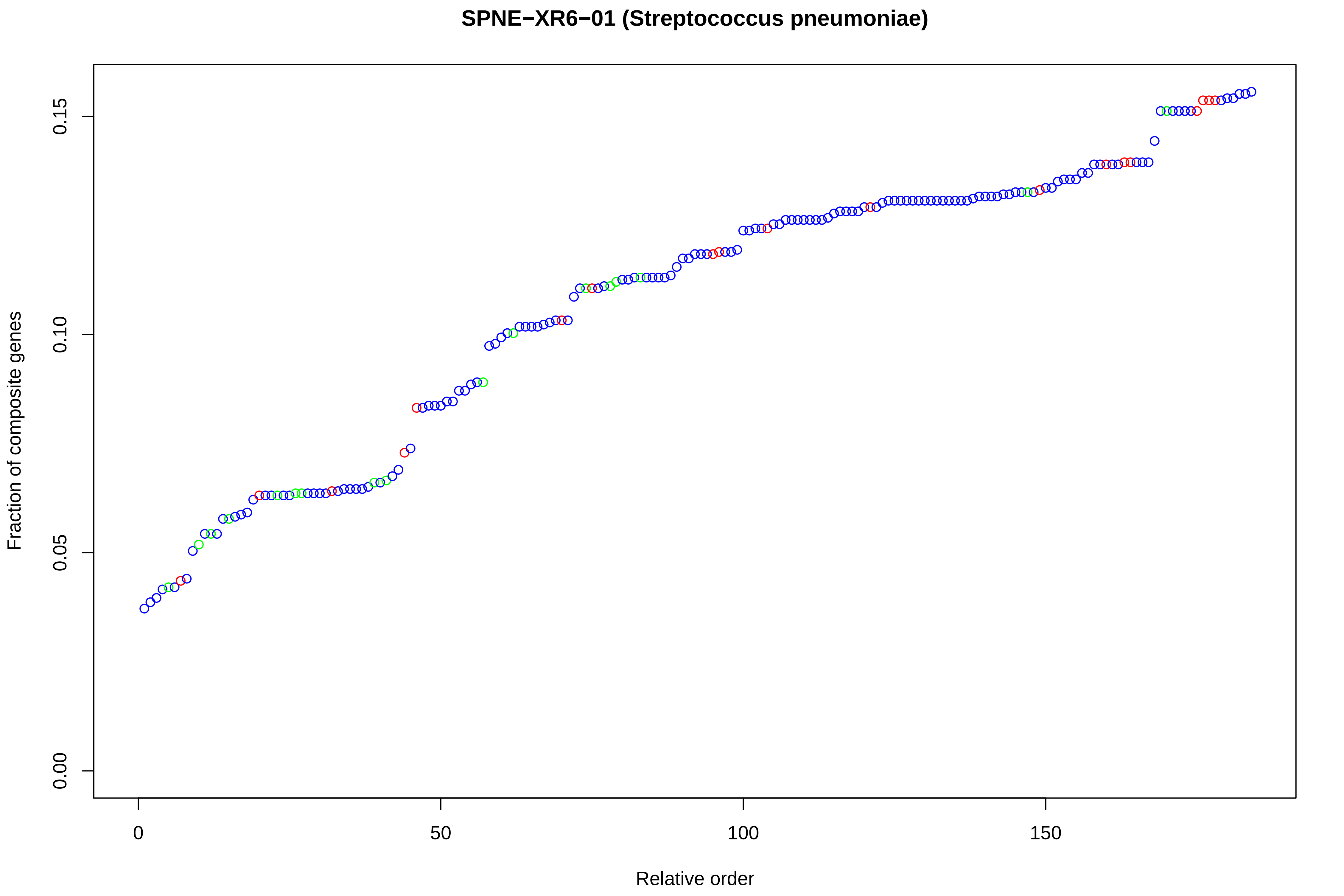

SPNE−XR6−01 (Streptococcus pneumoniae)
Fraction of composite genes
0.00
0.05
0.10
0.15
0
50
100
150
Relative order

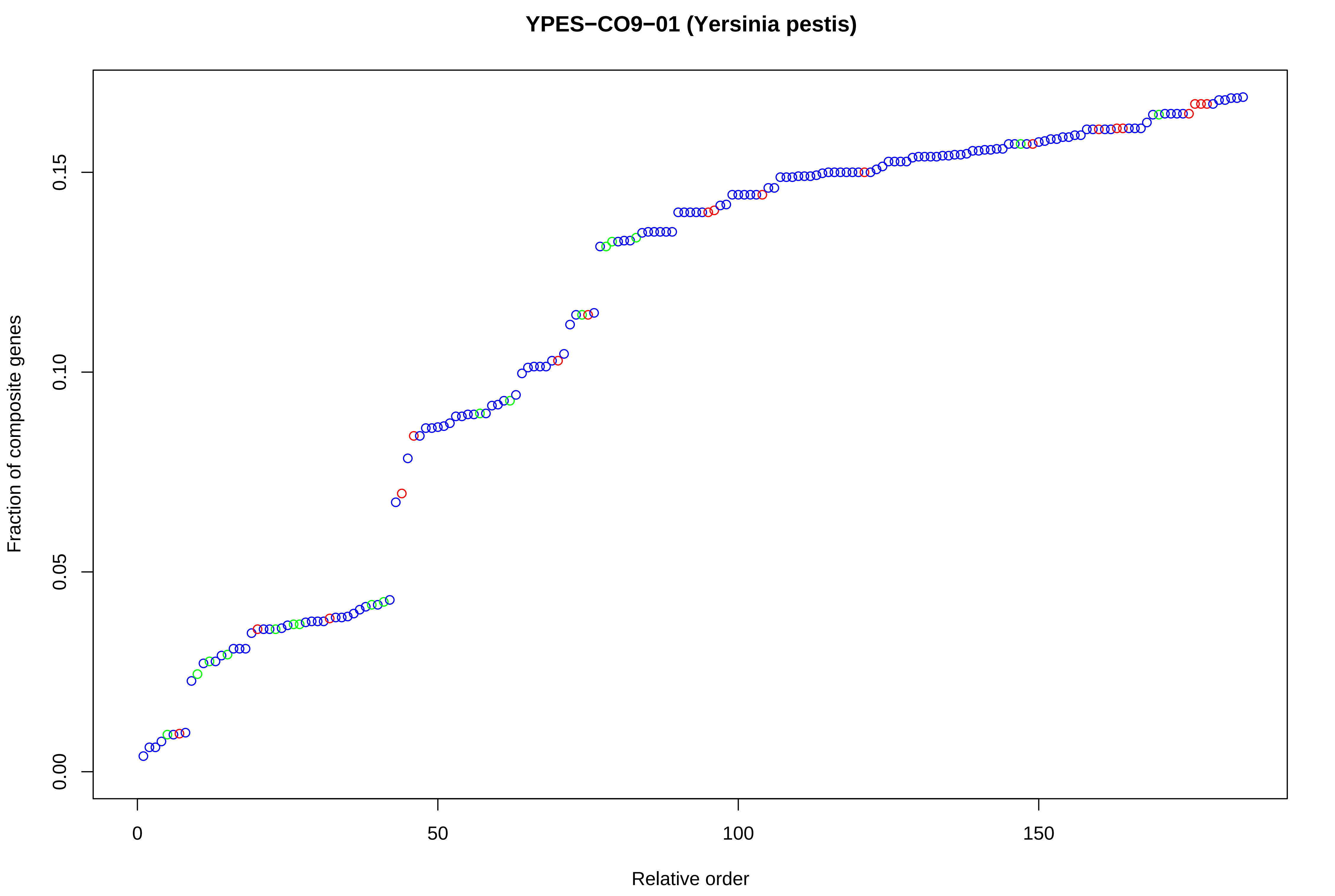

YPES−CO9−01 (Yersinia pestis)
Fraction of composite genes
0.00
0.05
0.10
0.15
0
50
100
150
Relative order

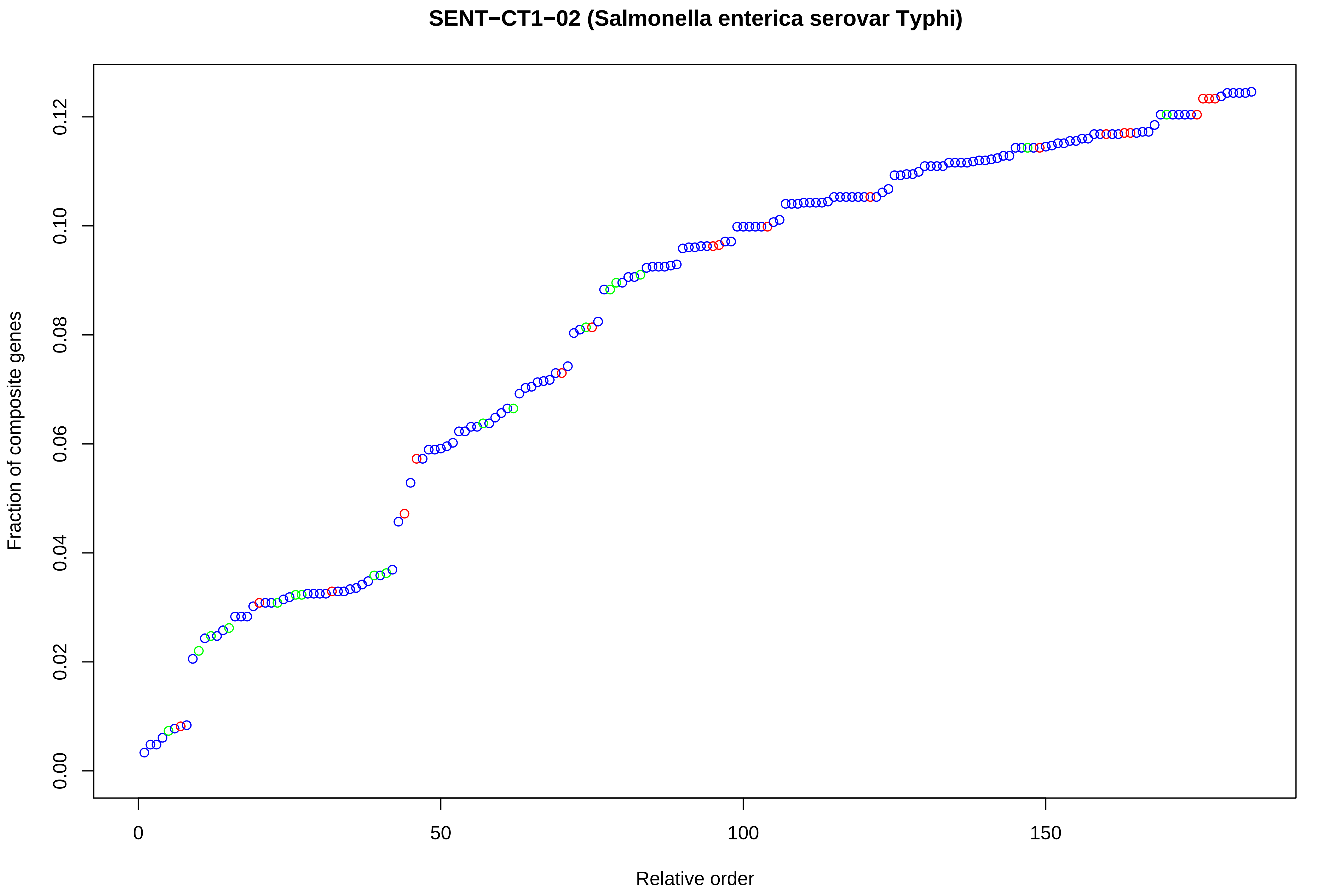

SENT−CT1−02 (Salmonella enterica serovar Typhi)
Fraction of composite genes
0.00
0.02
0.04
0.06
0.08
0.10
0.12
Relative order
0
50
100
150

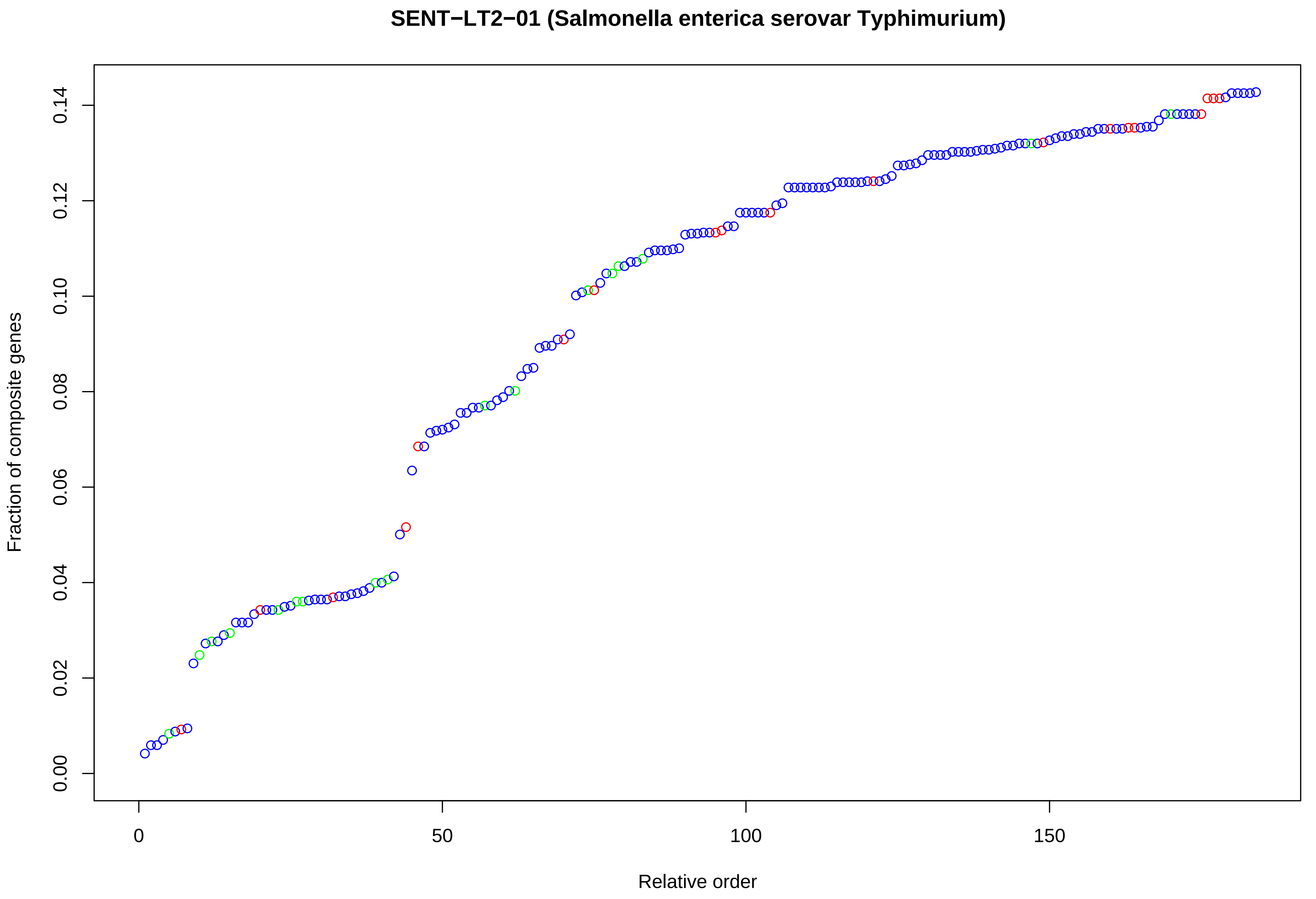
SENT−LT2−01 (Salmonella enterica serovar Typhimurium)
Fraction of composite genes
0.00
0.02
0.04
0.06
0.08
0.10
0.12
0.14
0
50
100
150
Relative order

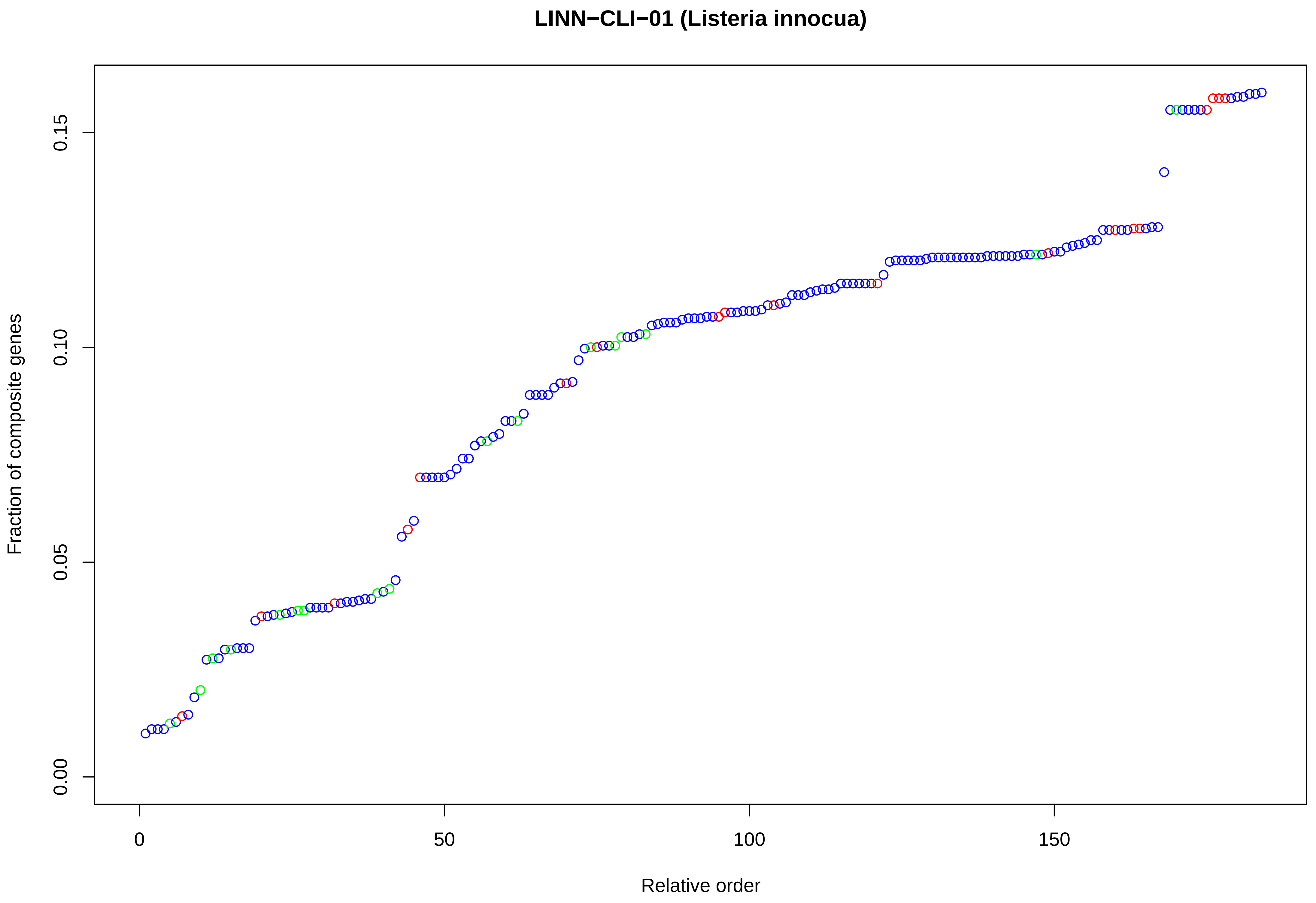

LINN−CLI−01 (Listeria innocua)
Fraction of composite genes
0.00
0.05
0.10
0.15
Relative order
0
50
100
150

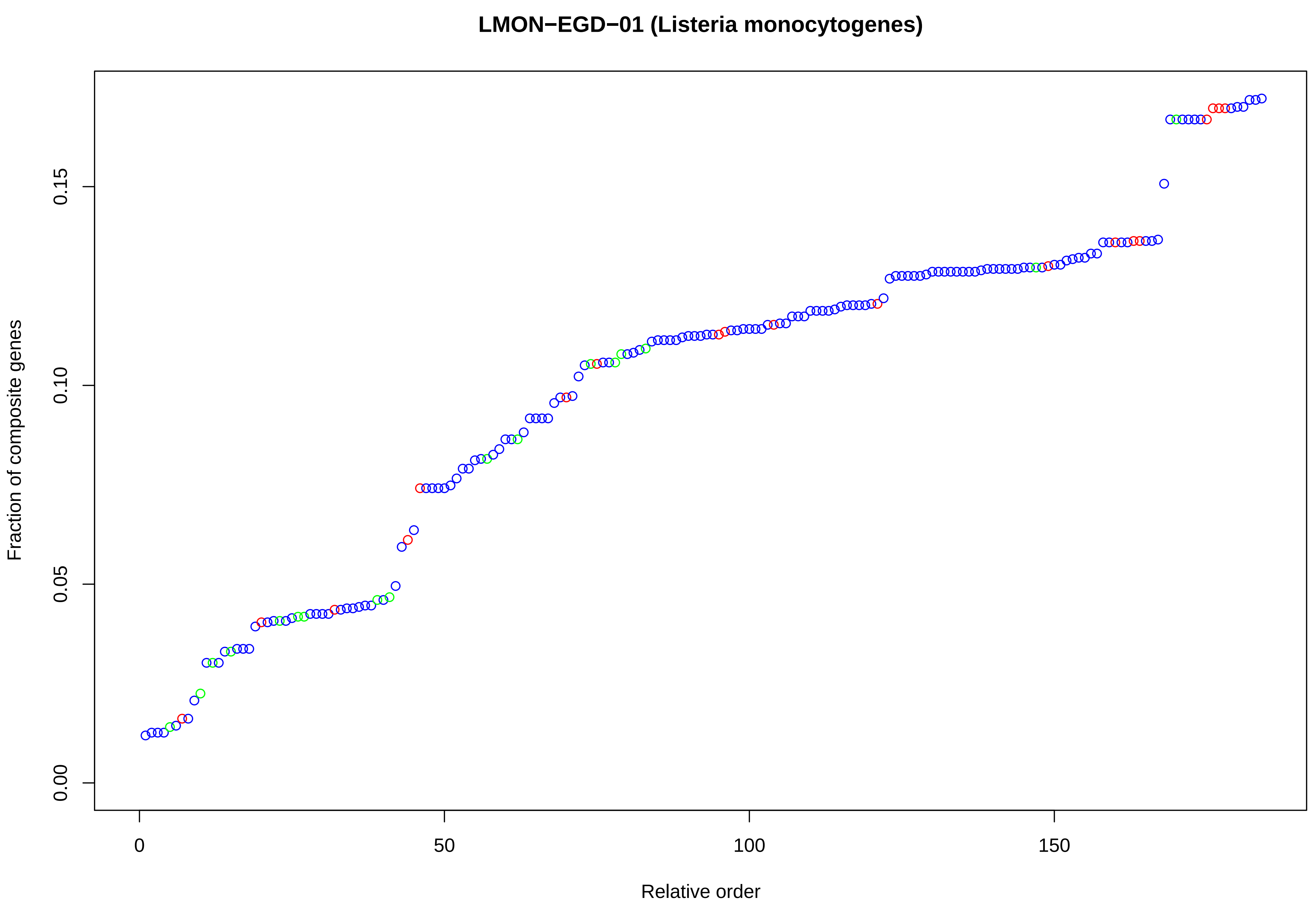

LMON−EGD−01 (Listeria monocytogenes)
Fraction of composite genes
Relative order
0.00
0.05
0.10
0.15
0
50
100
150

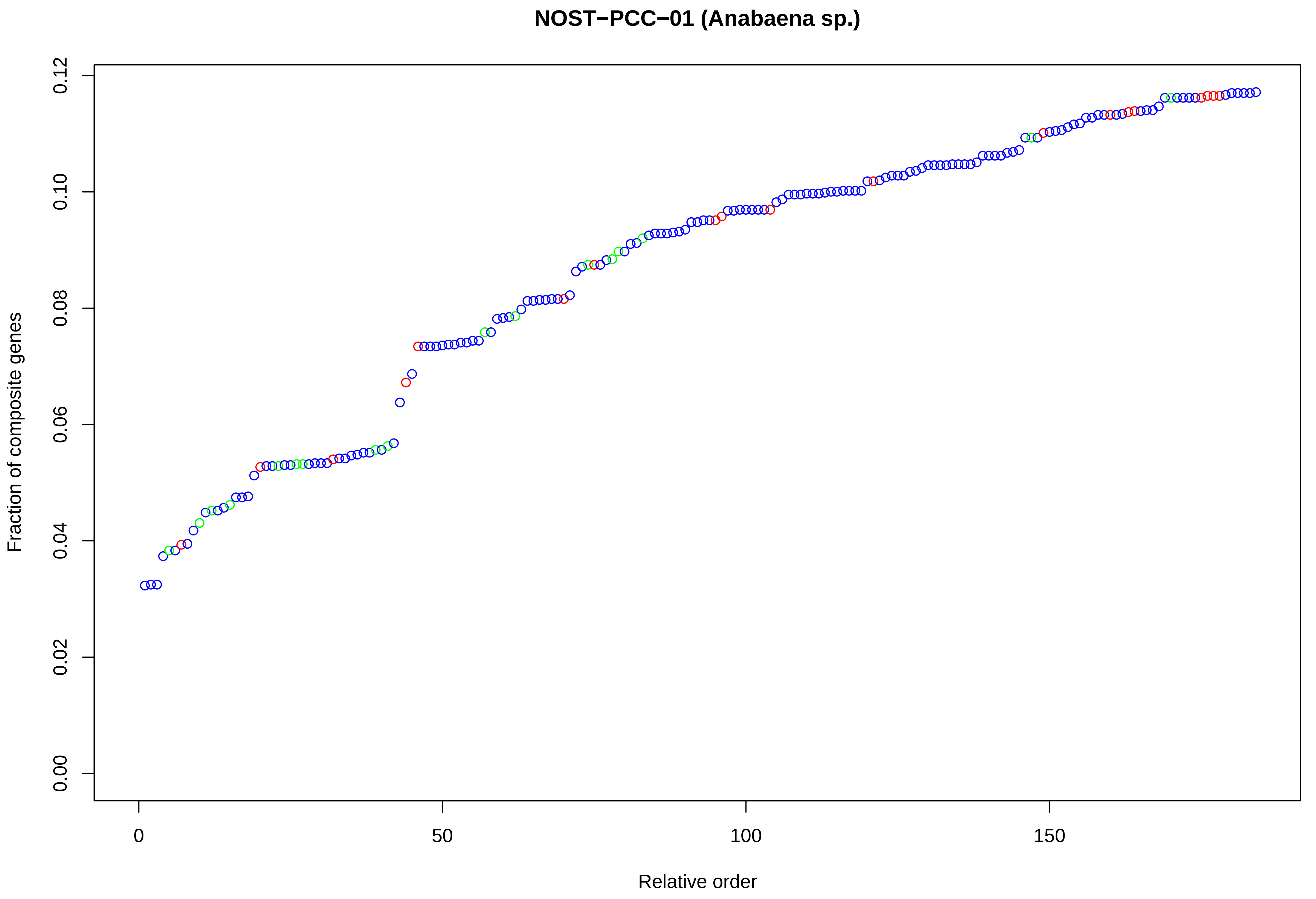
NOST−PCC−01 (Anabaena sp.)
Fraction of composite genes
Relative order
0.00
0.02
0.04
0.06
0.08
0.10
0.12
0
50
100
150

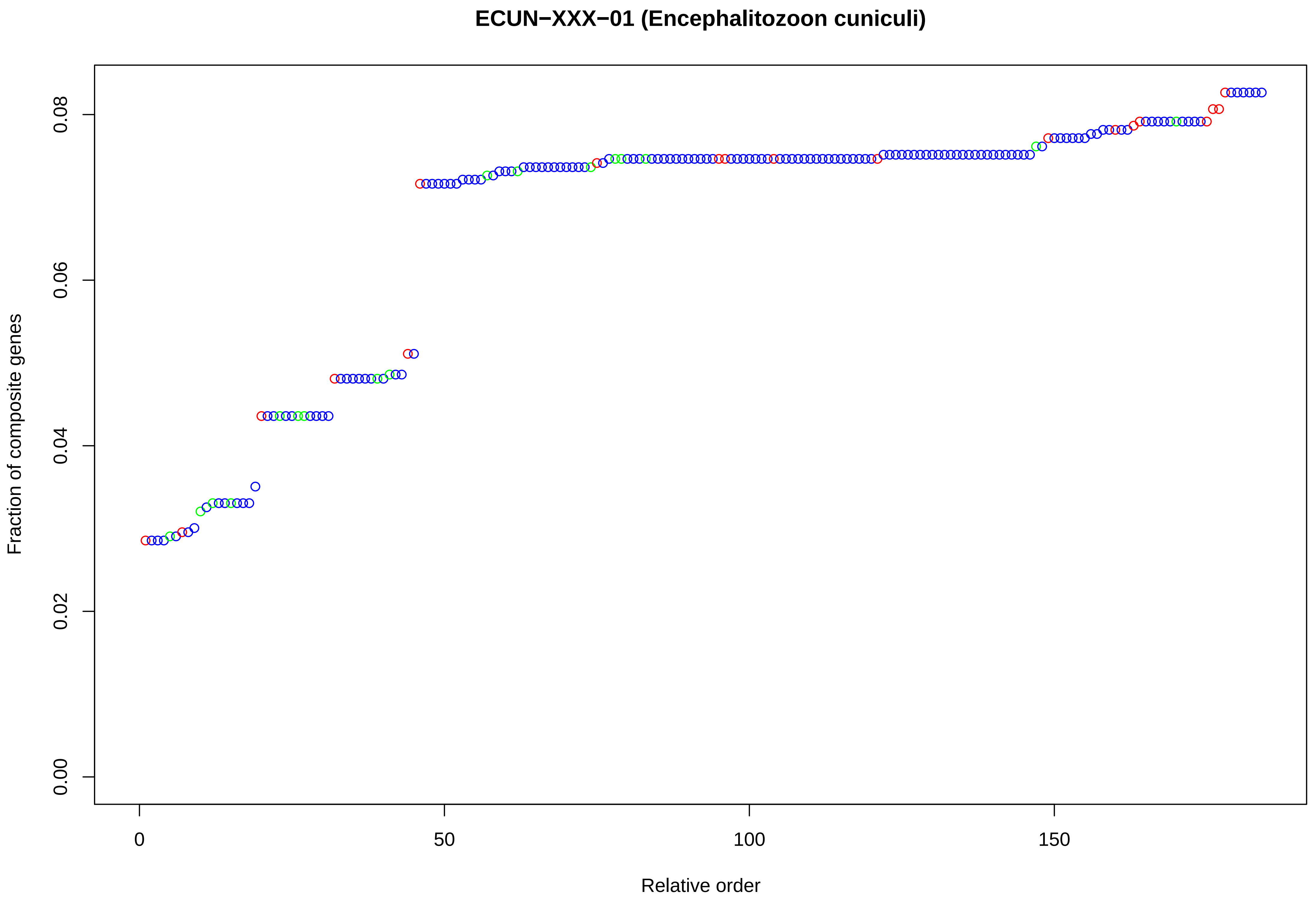
ECUN−XXX−01 (Encephalitozoon cuniculi)
Fraction of composite genes
0.00
0.02
0.04
0.06
0.08
0
50
100
150
Relative order

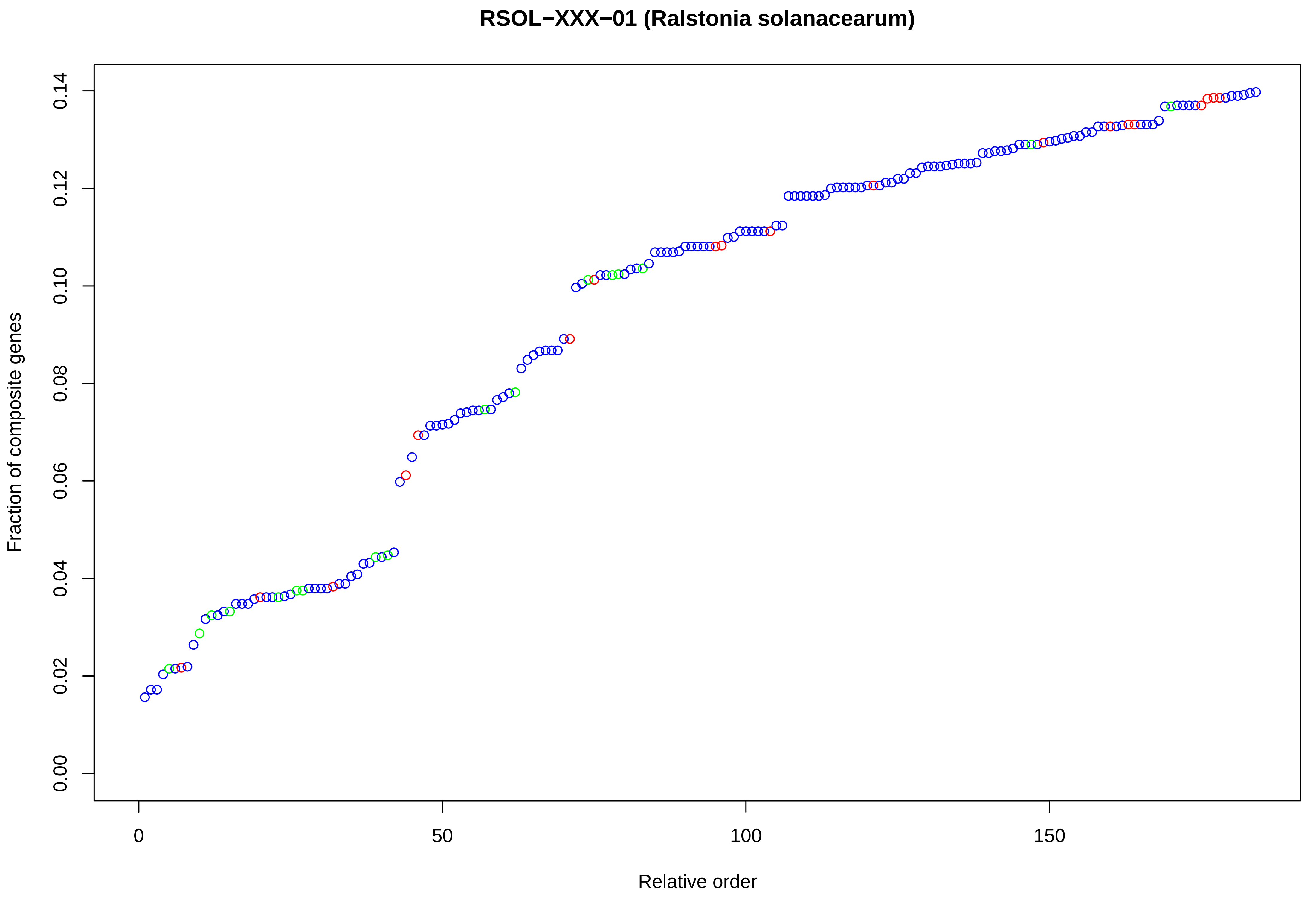

RSOL−XXX−01 (Ralstonia solanacearum)
Fraction of composite genes
0.00
0.02
0.04
0.06
0.08
0.10
0.12
0.14
Relative order
0
50
100
150

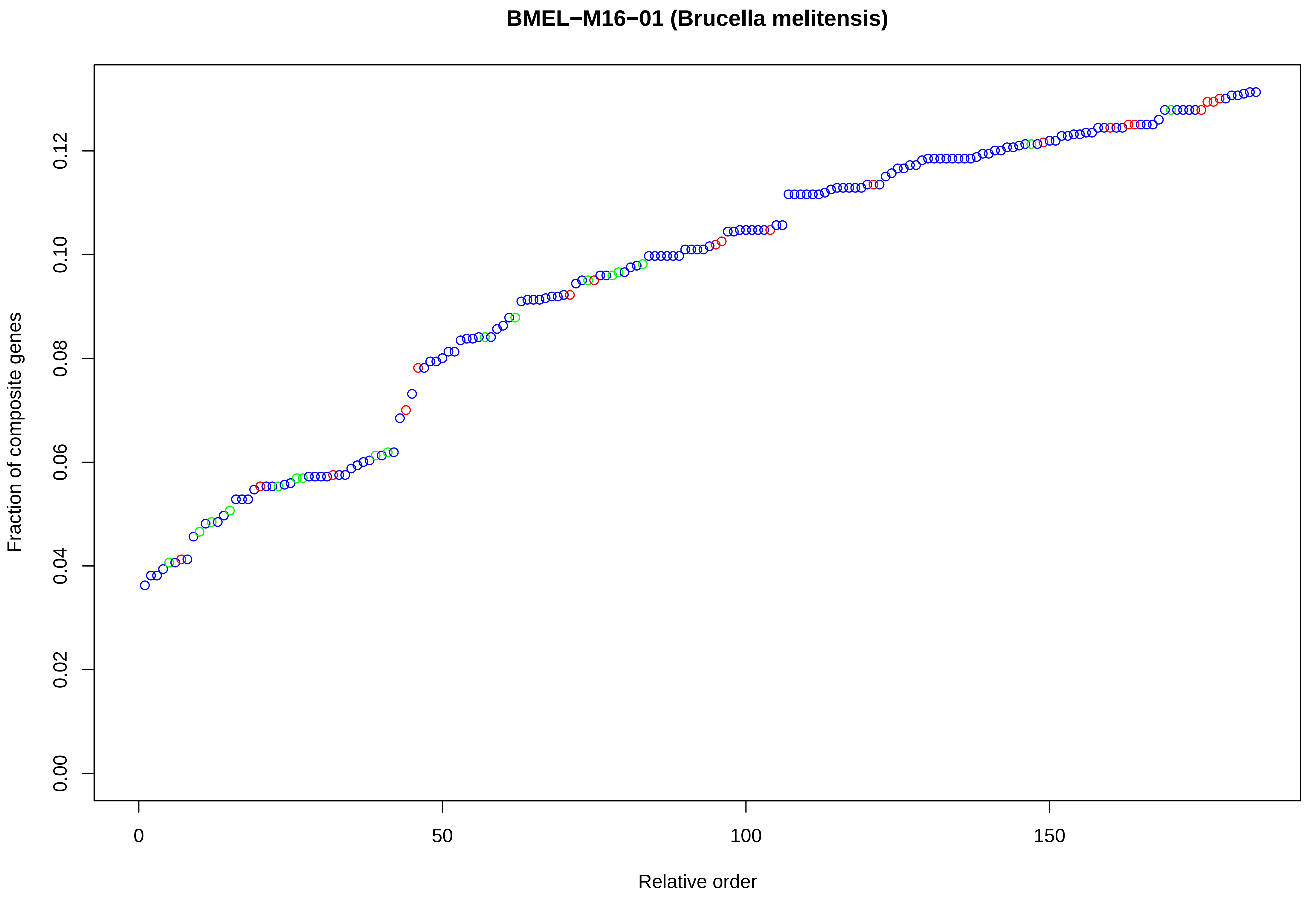

BMEL−M16−01 (Brucella melitensis)
Fraction of composite genes
0.00
0.02
0.04
0.06
0.08
0.10
0.12
0
50
100
150
Relative order

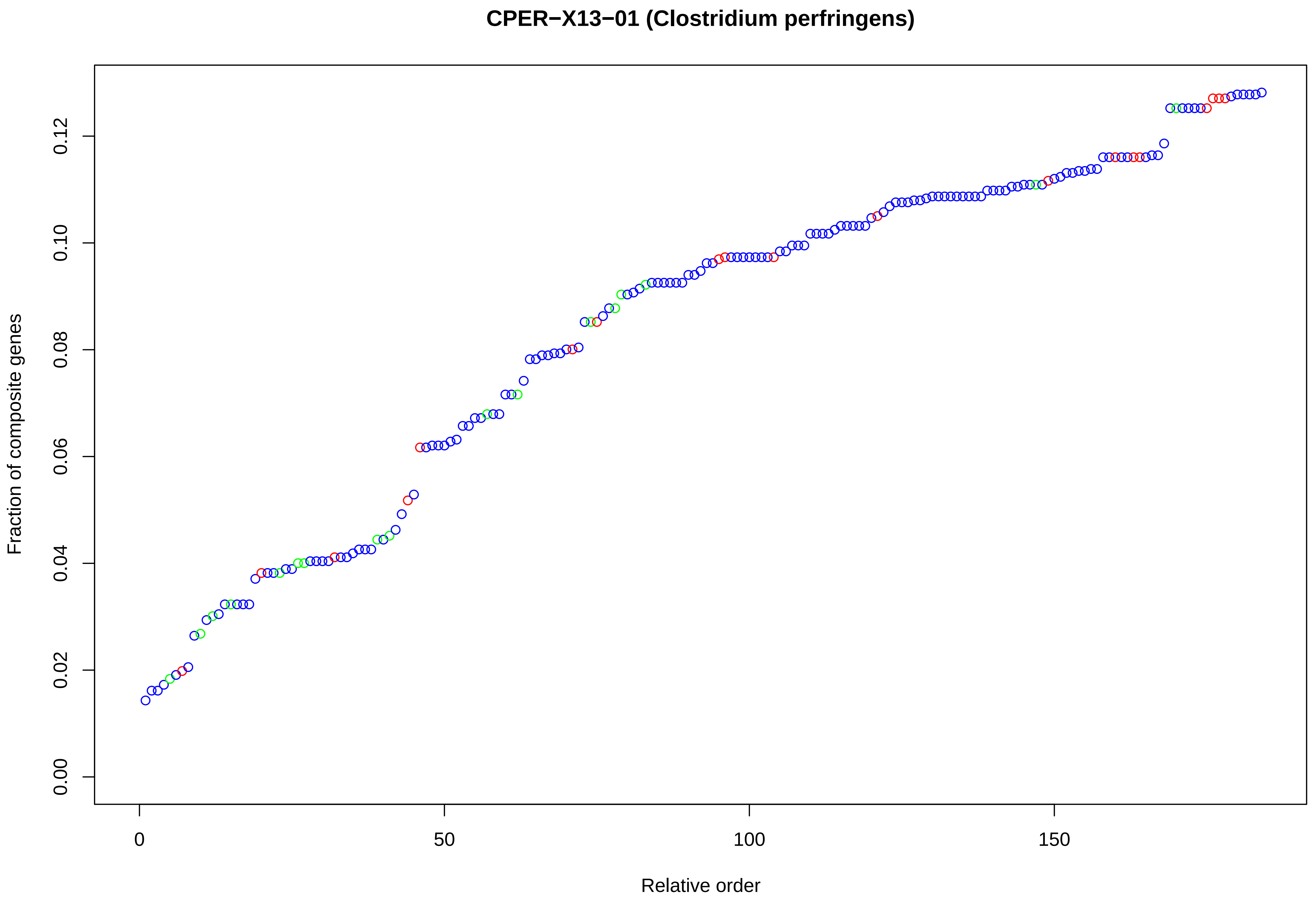
CPER−X13−01 (Clostridium perfringens)
Fraction of composite genes
0.00
0.02
0.04
0.06
0.08
0.10
0.12
Relative order
0
50
100
150

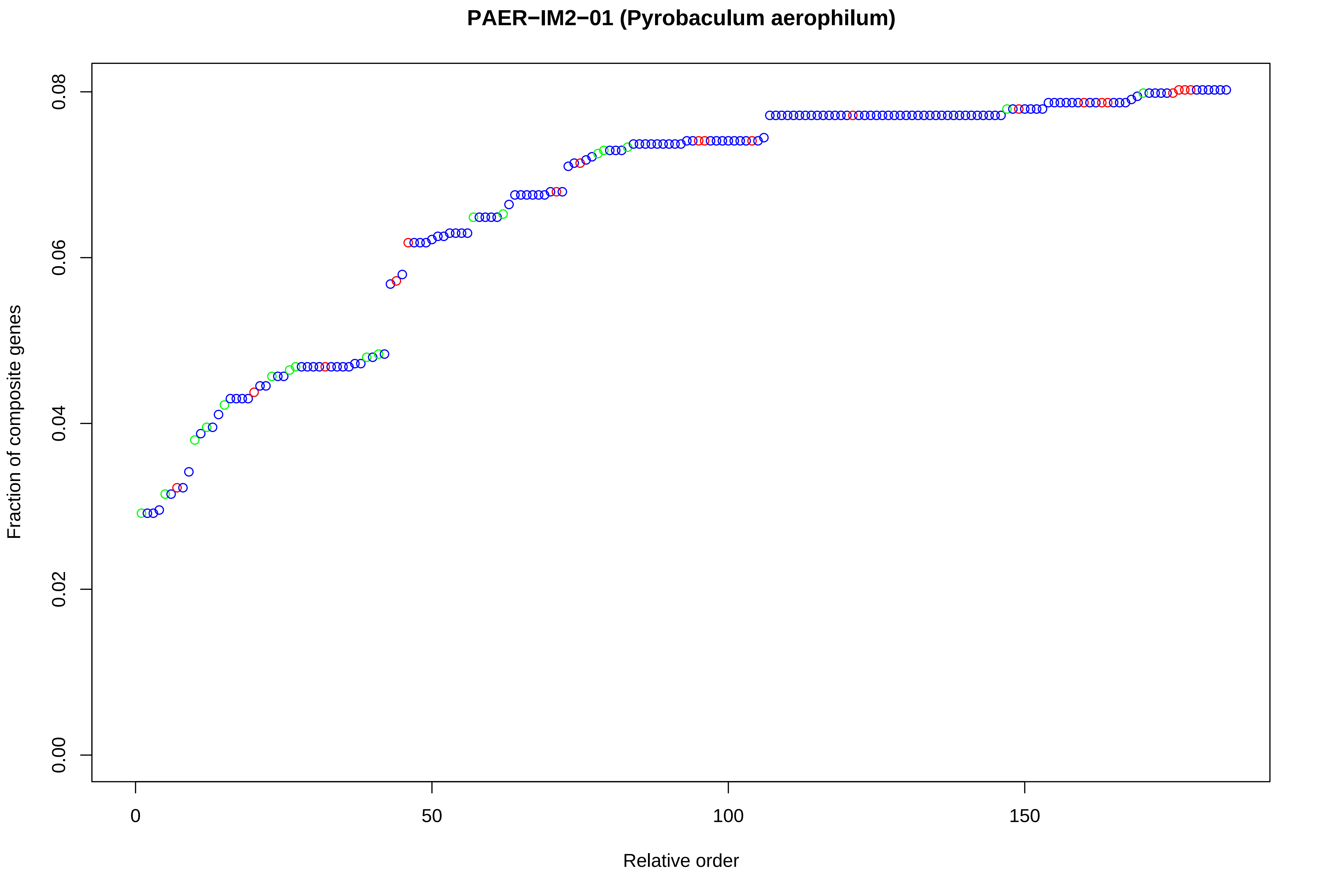
PAER−IM2−01 (Pyrobaculum aerophilum)
Fraction of composite genes
Relative order
0.00
0.02
0.04
0.06
0.08
0
50
100
150

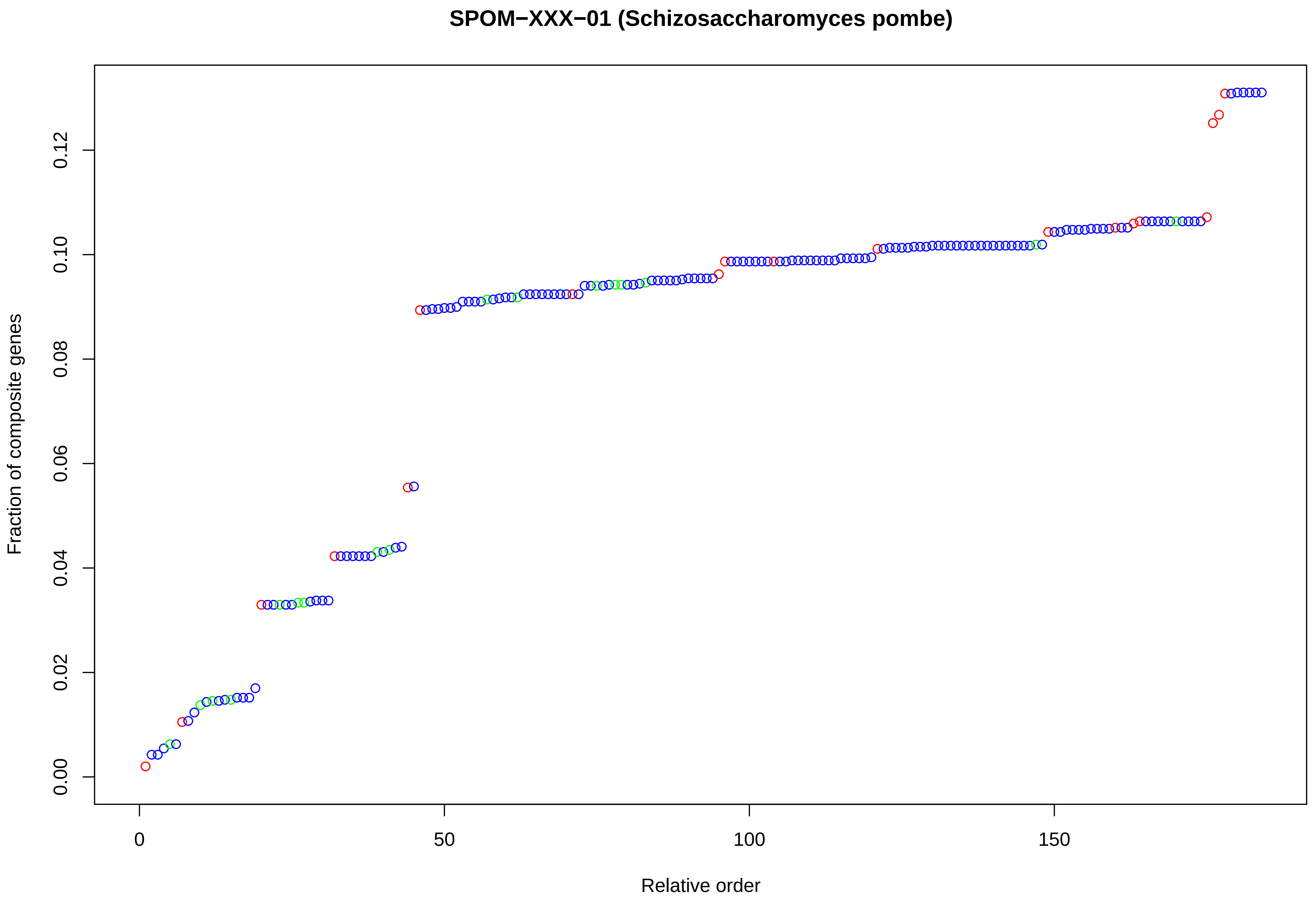
SPOM−XXX−01 (Schizosaccharomyces pombe)
Fraction of composite genes
0.00
0.02
0.04
0.06
0.08
0.10
0.12
Relative order
0
50
100
150

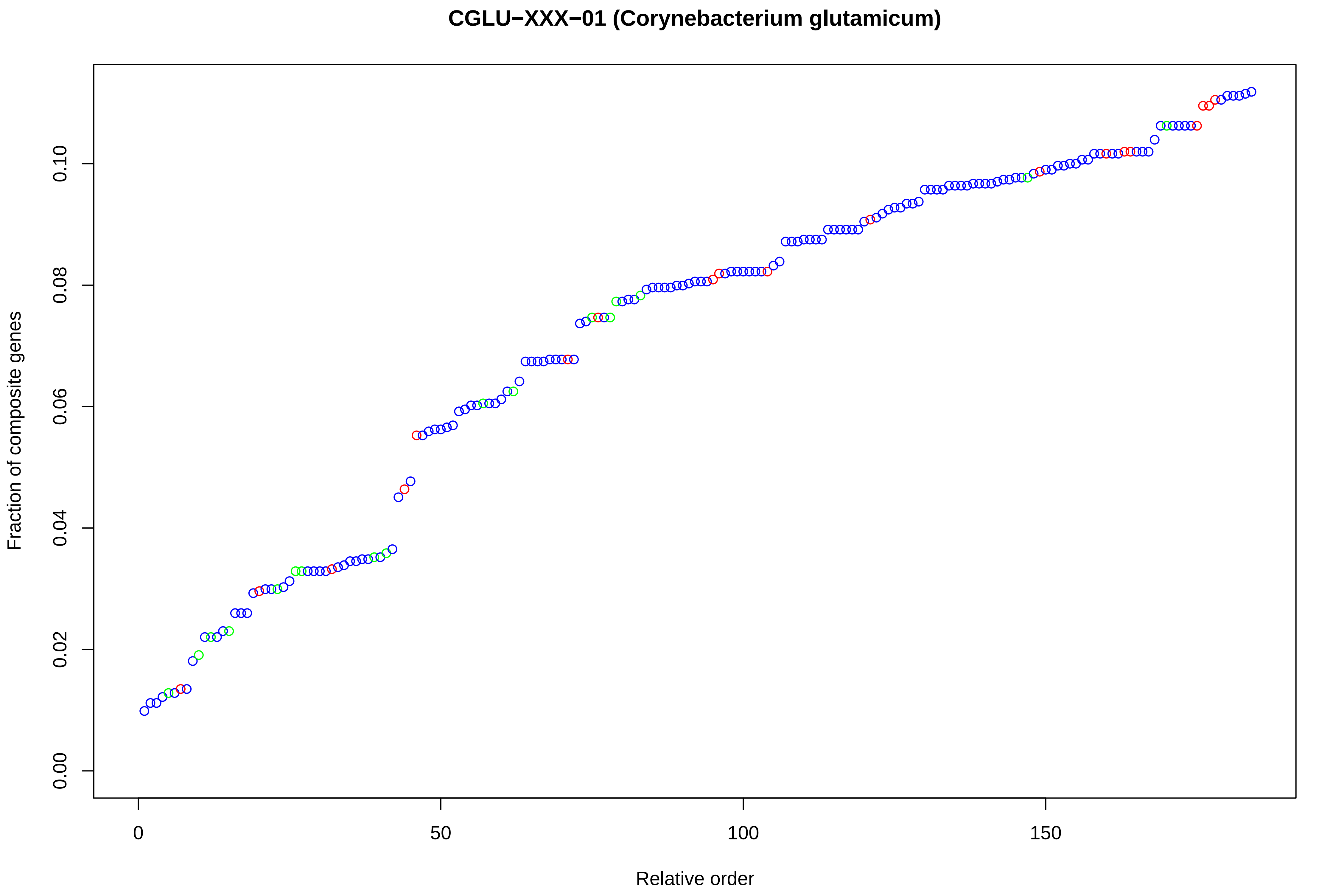

CGLU−XXX−01 (Corynebacterium glutamicum)
Fraction of composite genes
0.00
0.02
0.04
0.06
0.08
0.10
Relative order
0
50
100
150

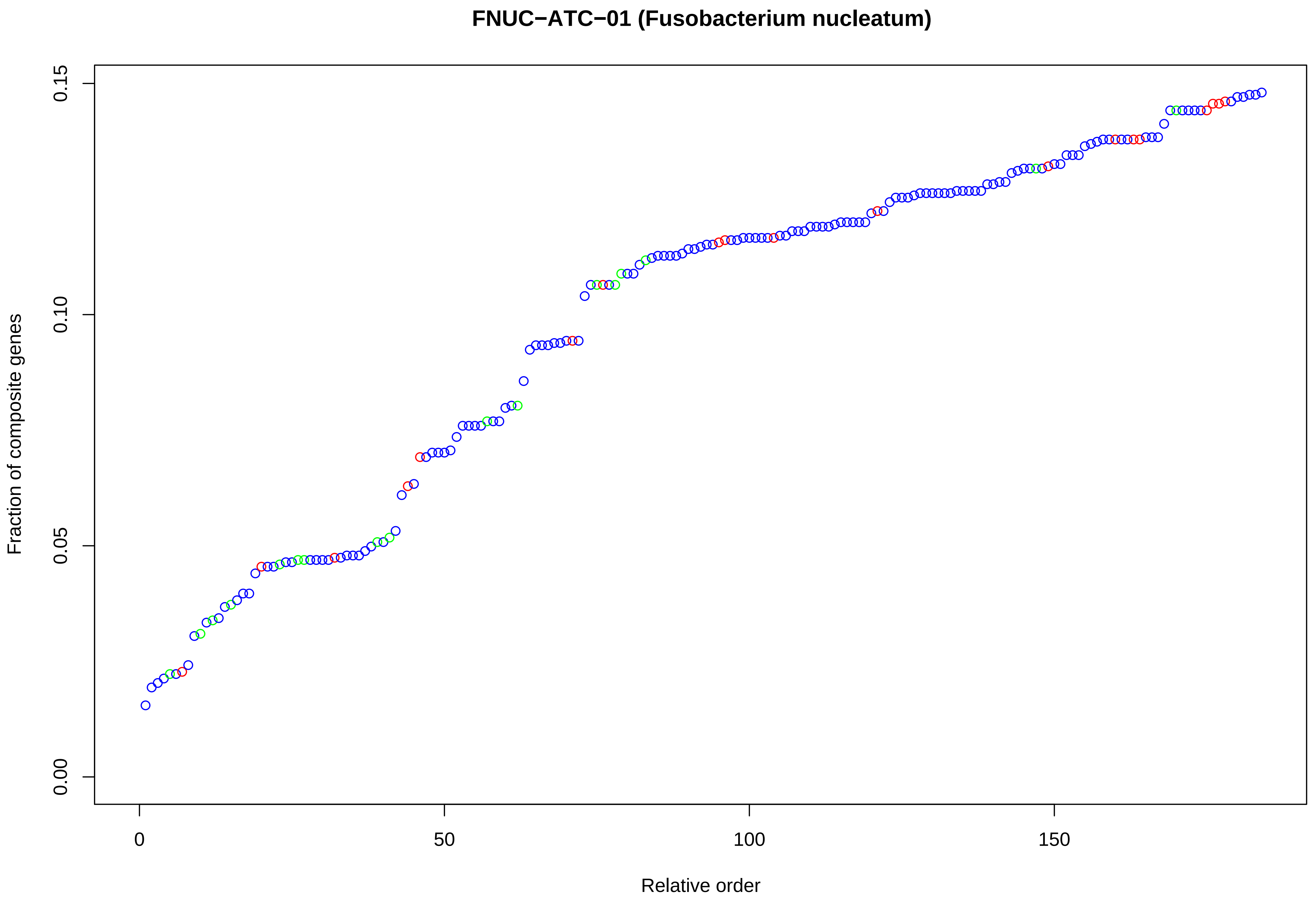

FNUC−ATC−01 (Fusobacterium nucleatum)
Fraction of composite genes
0.00
0.05
0.10
0.15
0
50
100
150
Relative order

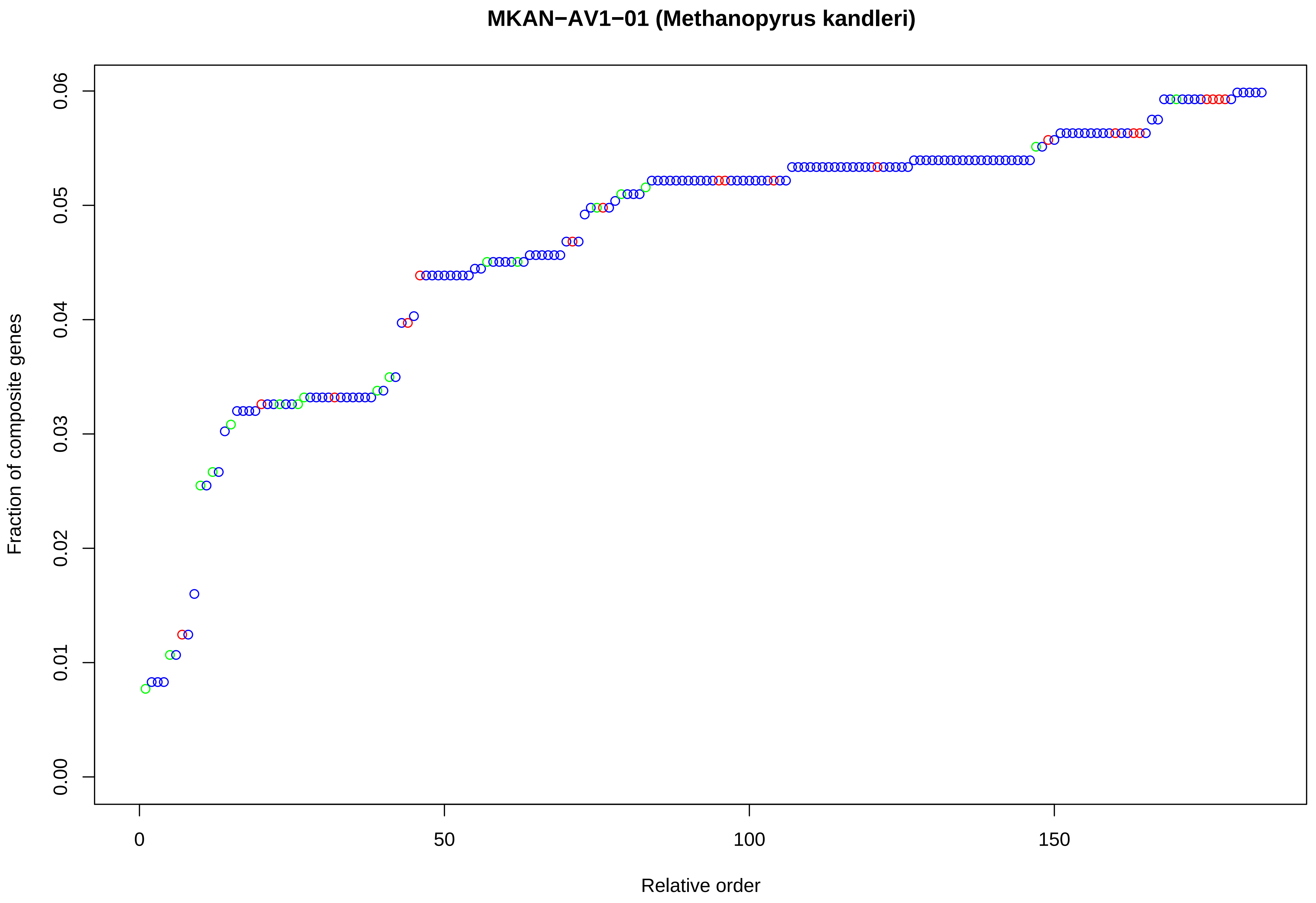
MKAN−AV1−01 (Methanopyrus kandleri)
Fraction of composite genes
0.00
0.01
0.02
0.03
0.04
0.05
0.06
Relative order
0
50
100
150

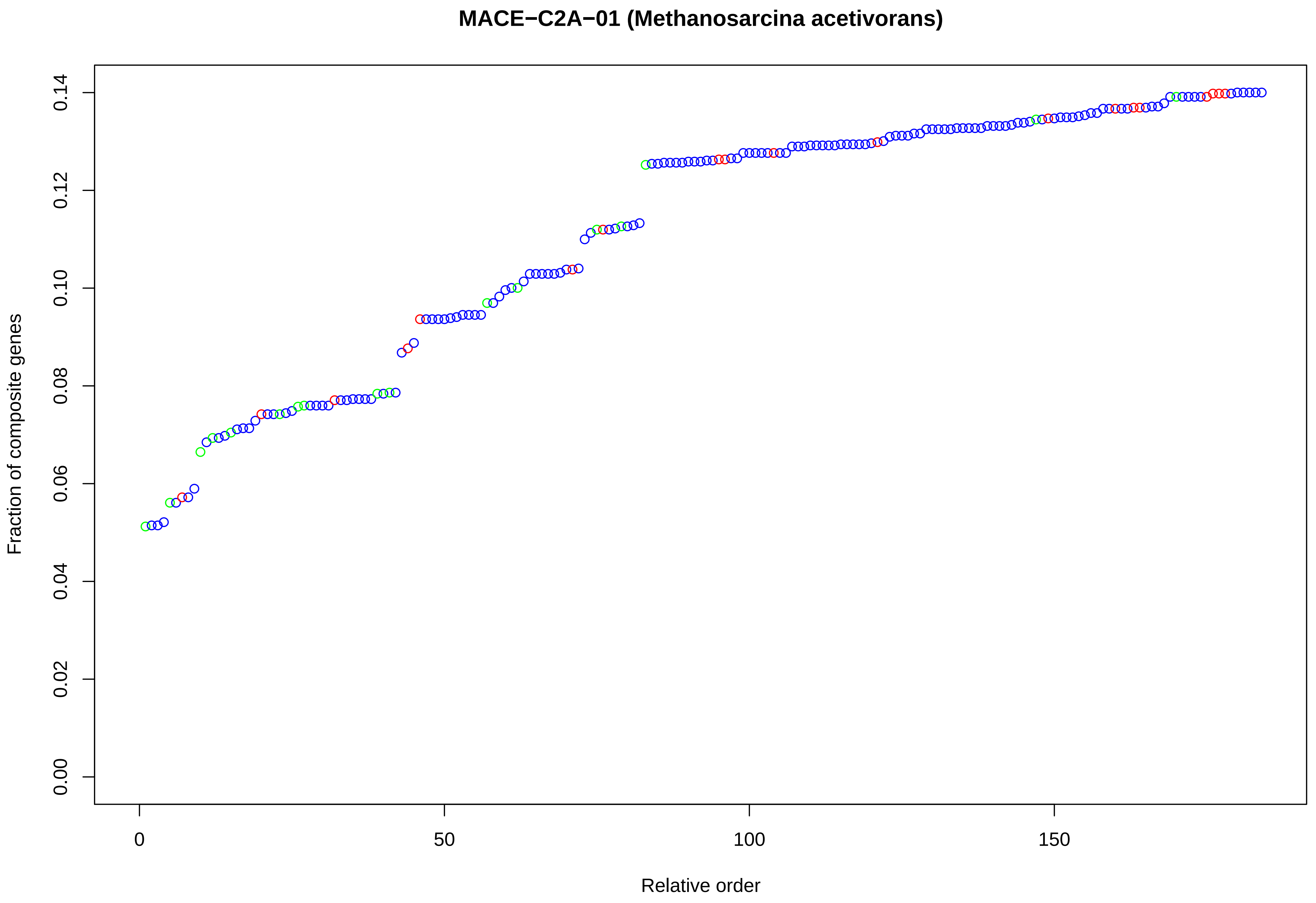

MACE−C2A−01 (Methanosarcina acetivorans)
Fraction of composite genes
0.00
0.02
0.04
0.06
0.08
0.10
0.12
0.14
0
50
100
150
Relative order

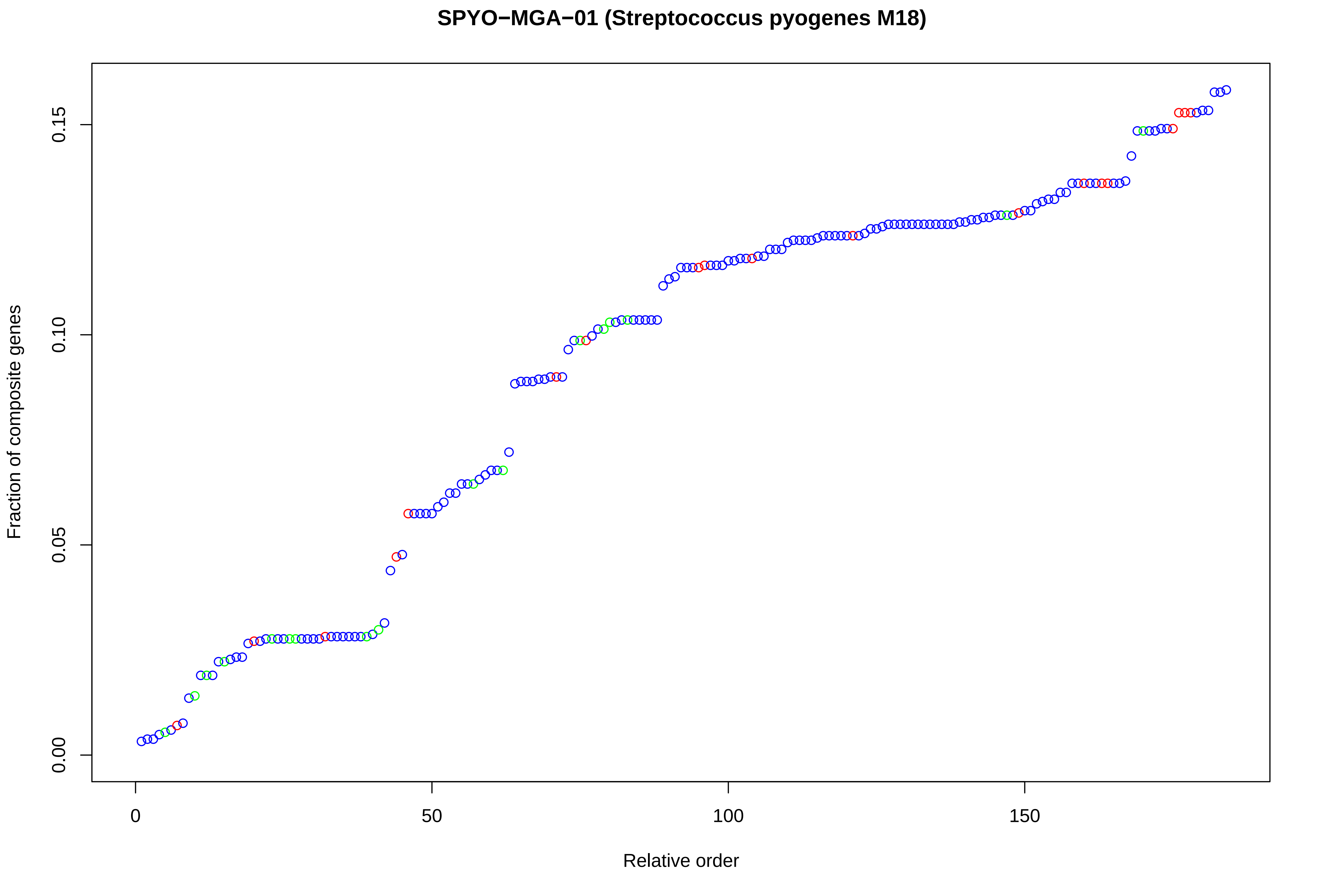
SPYO−MGA−01 (Streptococcus pyogenes M18)
Fraction of composite genes
0.00
0.05
0.10
0.15
0
50
100
150
Relative order

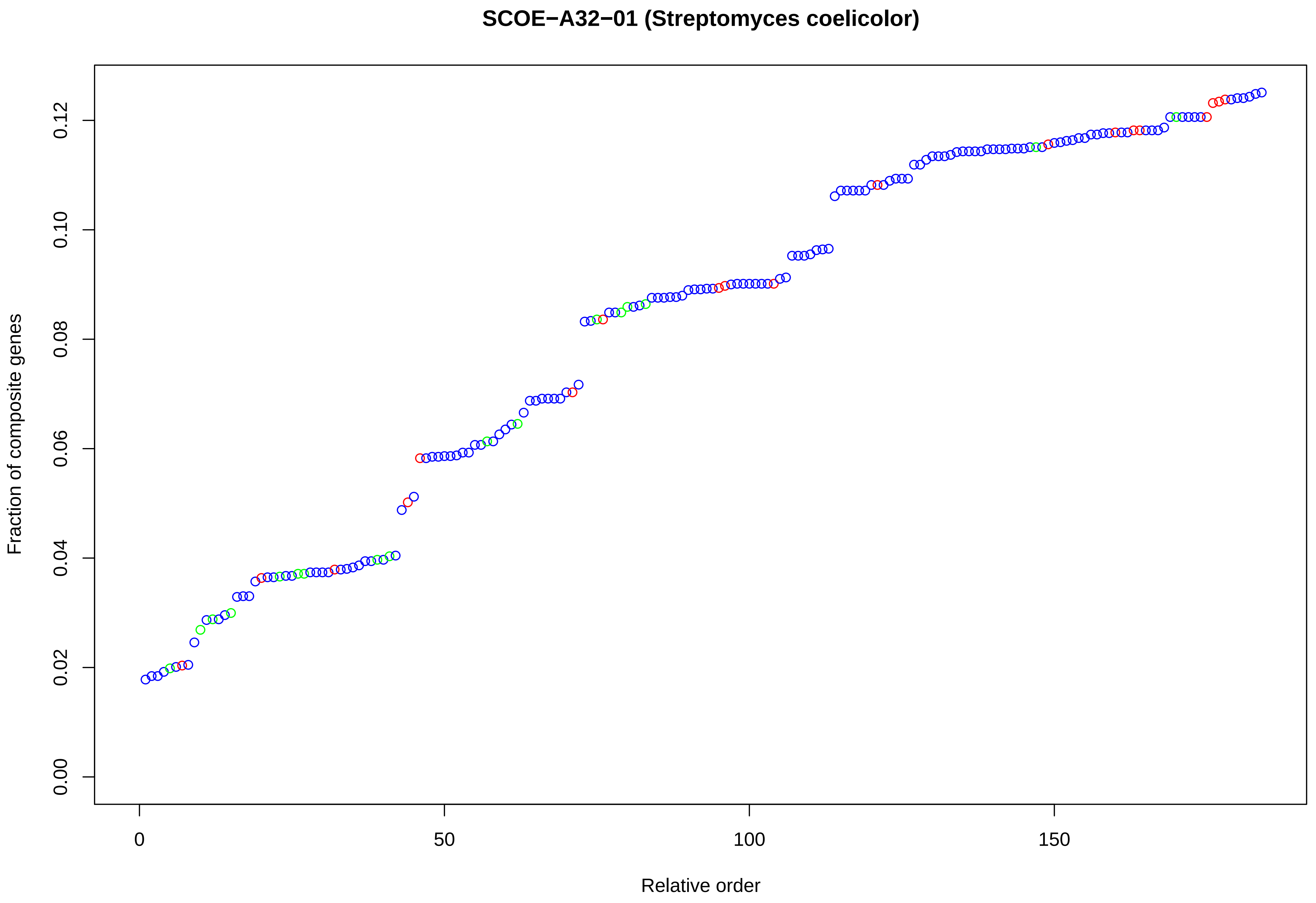
SCOE−A32−01 (Streptomyces coelicolor)
Fraction of composite genes
0.00
0.02
0.04
0.06
0.08
0.10
0.12
0
50
100
150
Relative order

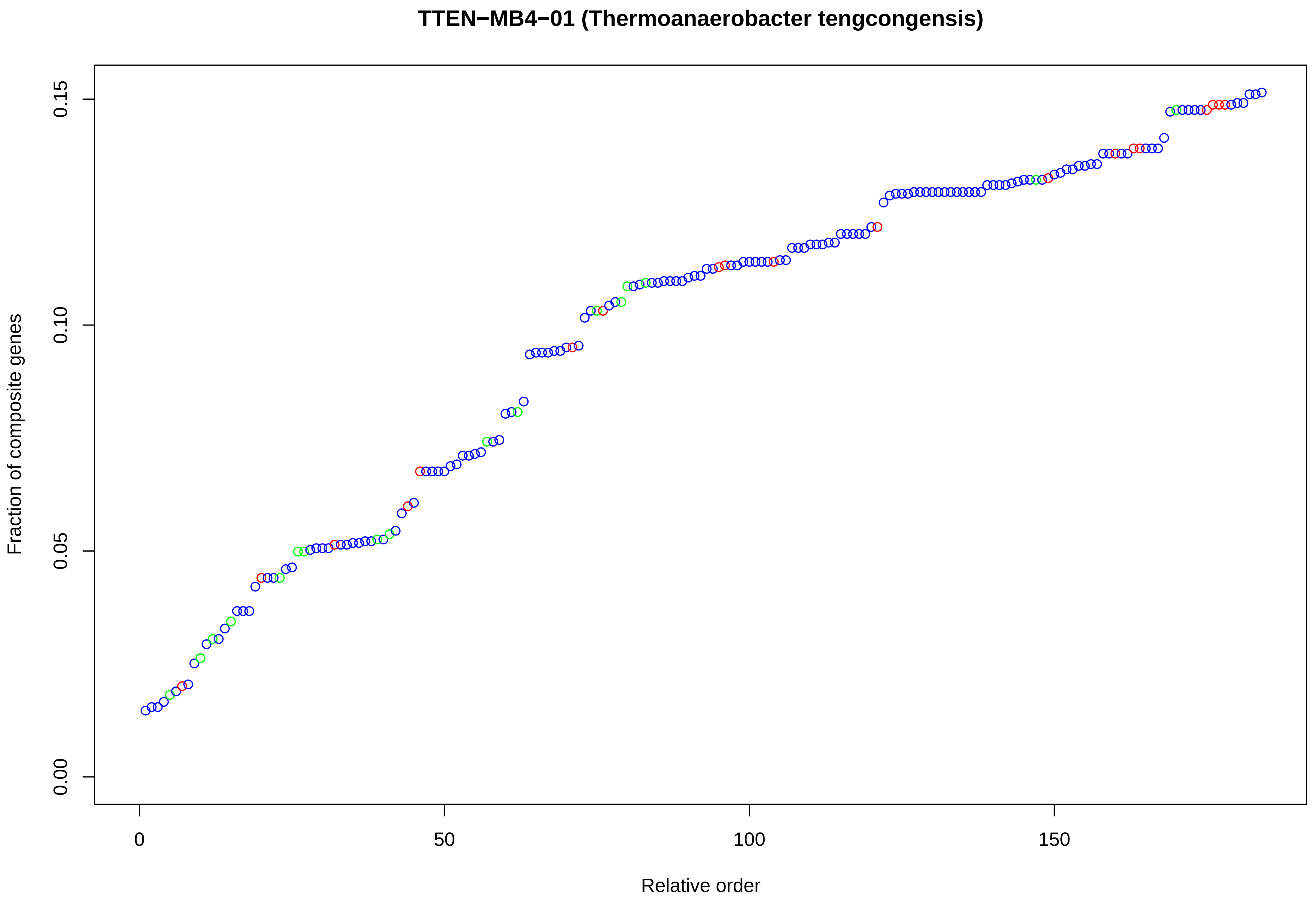

TTEN−MB4−01 (Thermoanaerobacter tengcongensis)
Fraction of composite genes
0.00
0.05
0.10
0.15
0
50
100
150
Relative order

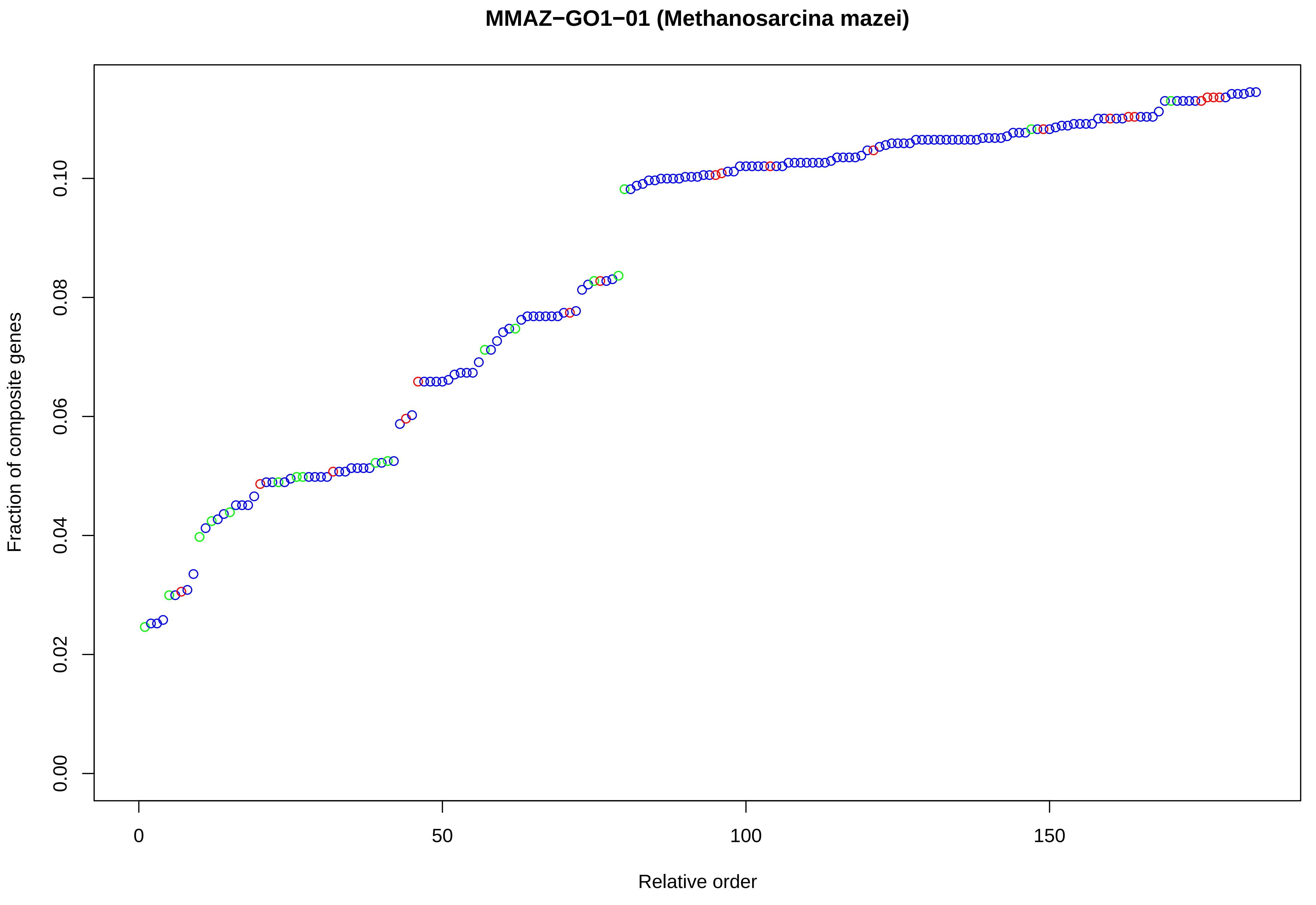
MMAZ−GO1−01 (Methanosarcina mazei)
Fraction of composite genes
Relative order
0.00
0.02
0.04
0.06
0.08
0.10
0
50
100
150

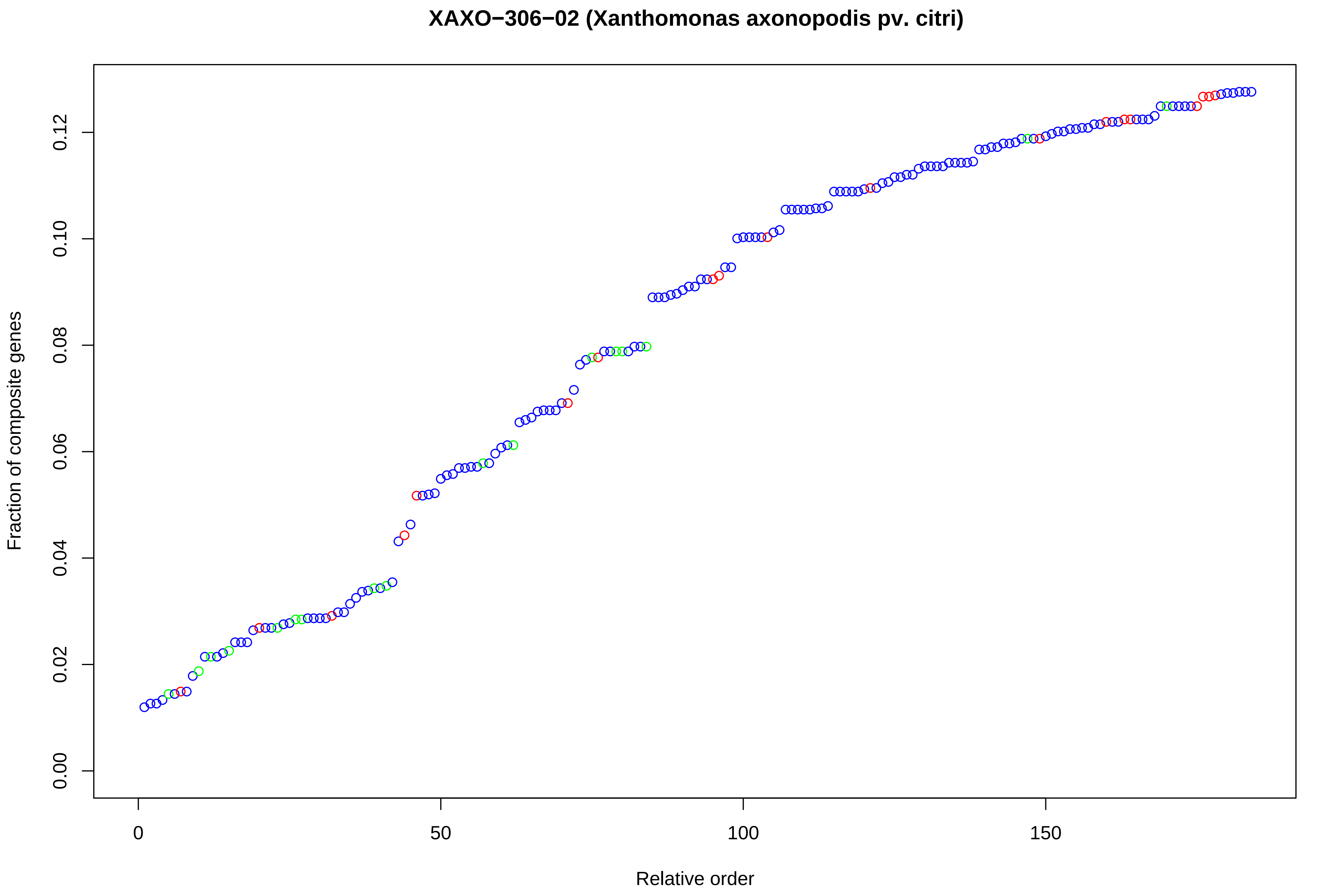

XAXO−306−02 (Xanthomonas axonopodis pv. citri)
Fraction of composite genes
0.00
0.02
0.04
0.06
0.08
0.10
0.12
Relative order
0
50
100
150

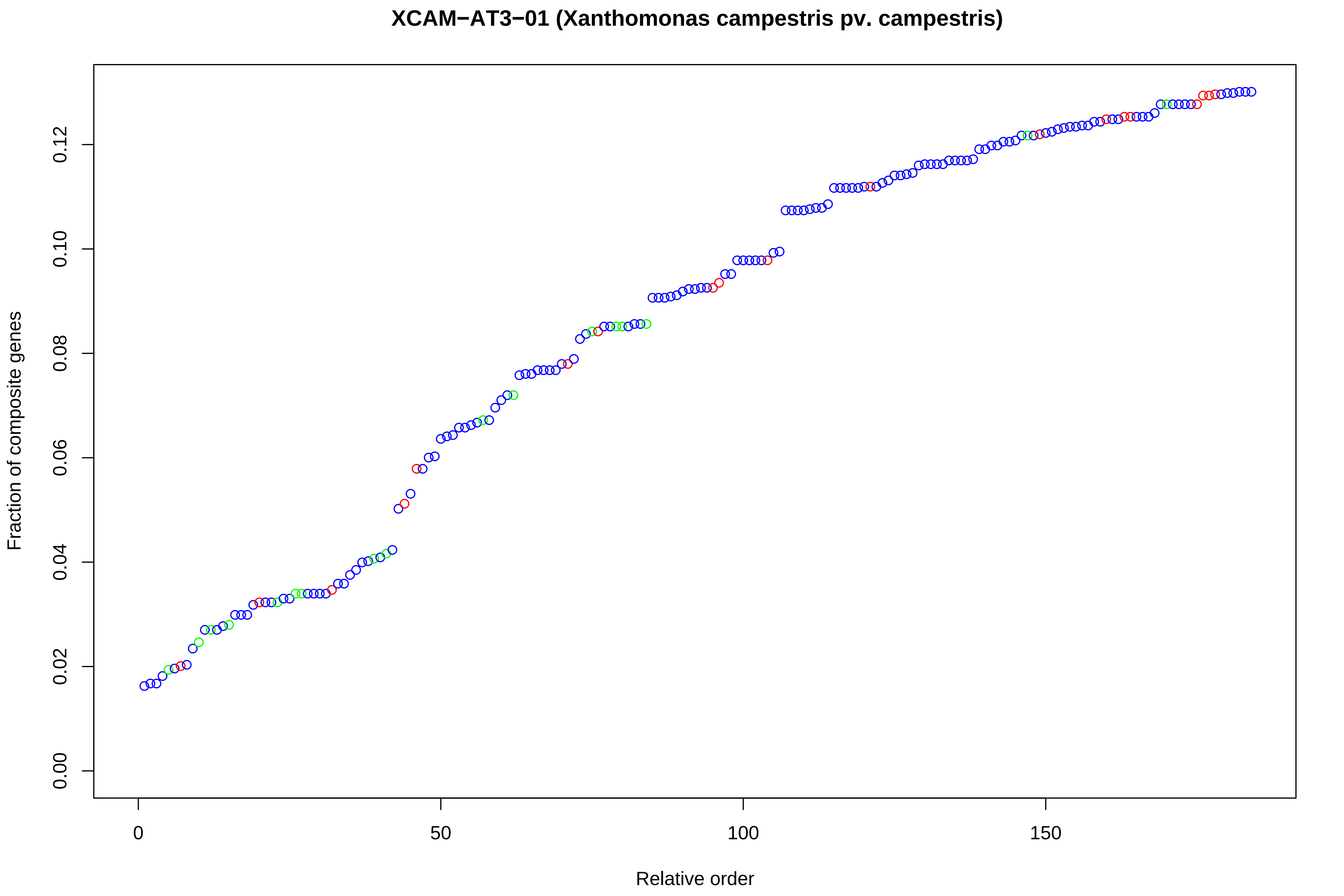
XCAM−AT3−01 (Xanthomonas campestris pv. campestris)
Fraction of composite genes
0.00
0.02
0.04
0.06
0.08
0.10
0.12
0
50
100
150
Relative order

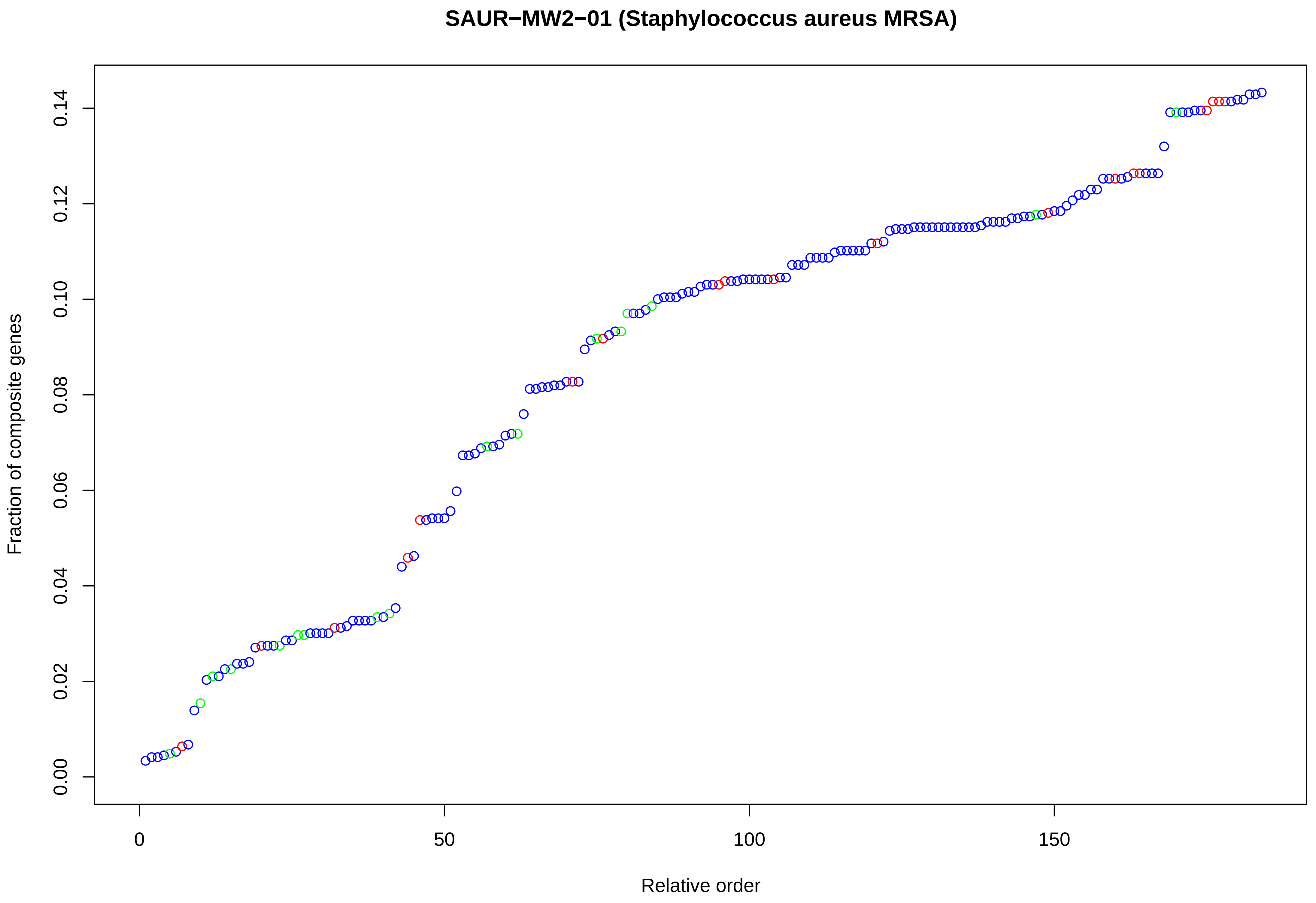

SAUR−MW2−01 (Staphylococcus aureus MRSA)
Fraction of composite genes
0.00
0.02
0.04
0.06
0.08
0.10
0.12
0.14
0
50
100
150
Relative order

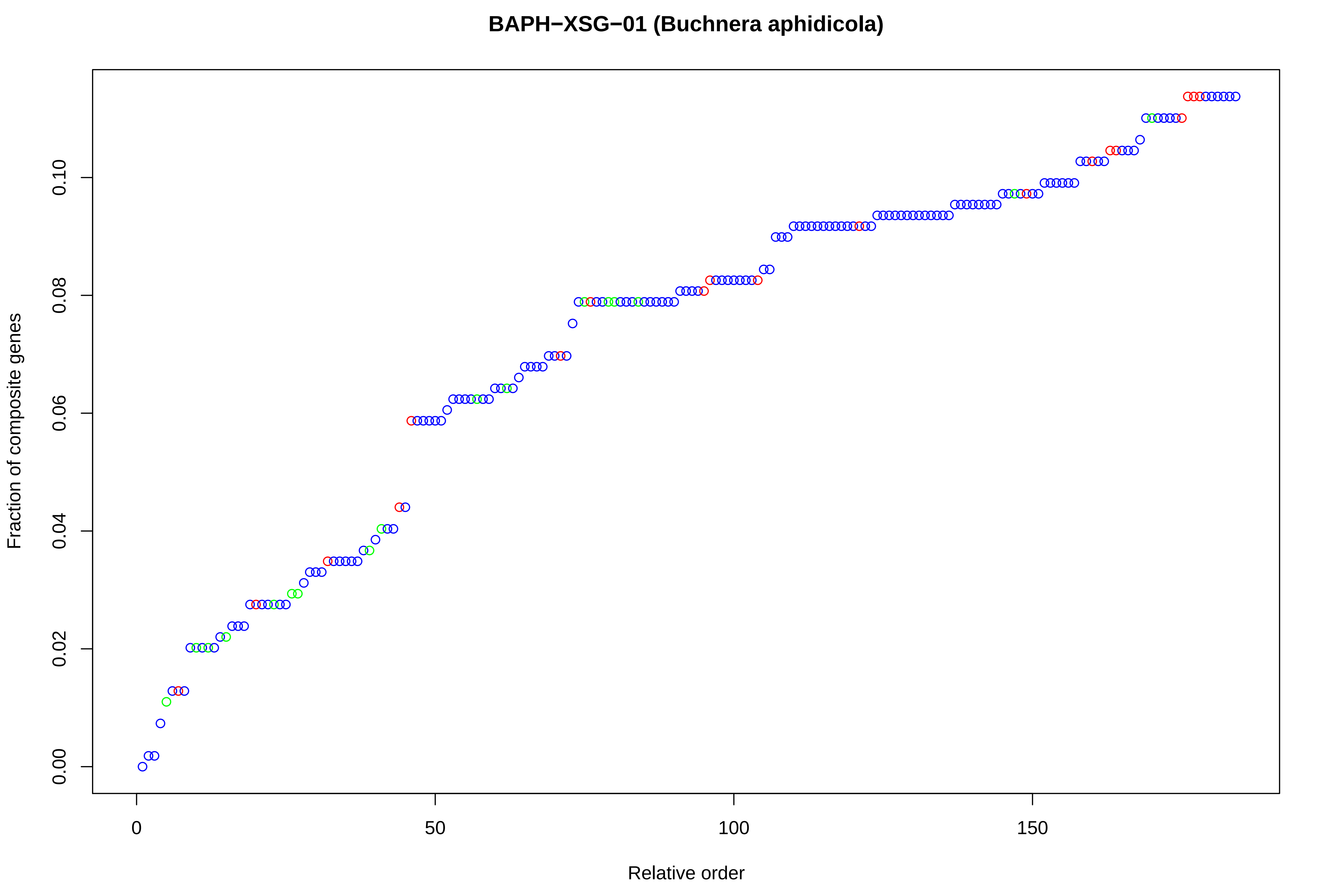

BAPH−XSG−01 (Buchnera aphidicola)
Fraction of composite genes
0.00
0.02
0.04
0.06
0.08
0.10
Relative order
0
50
100
150

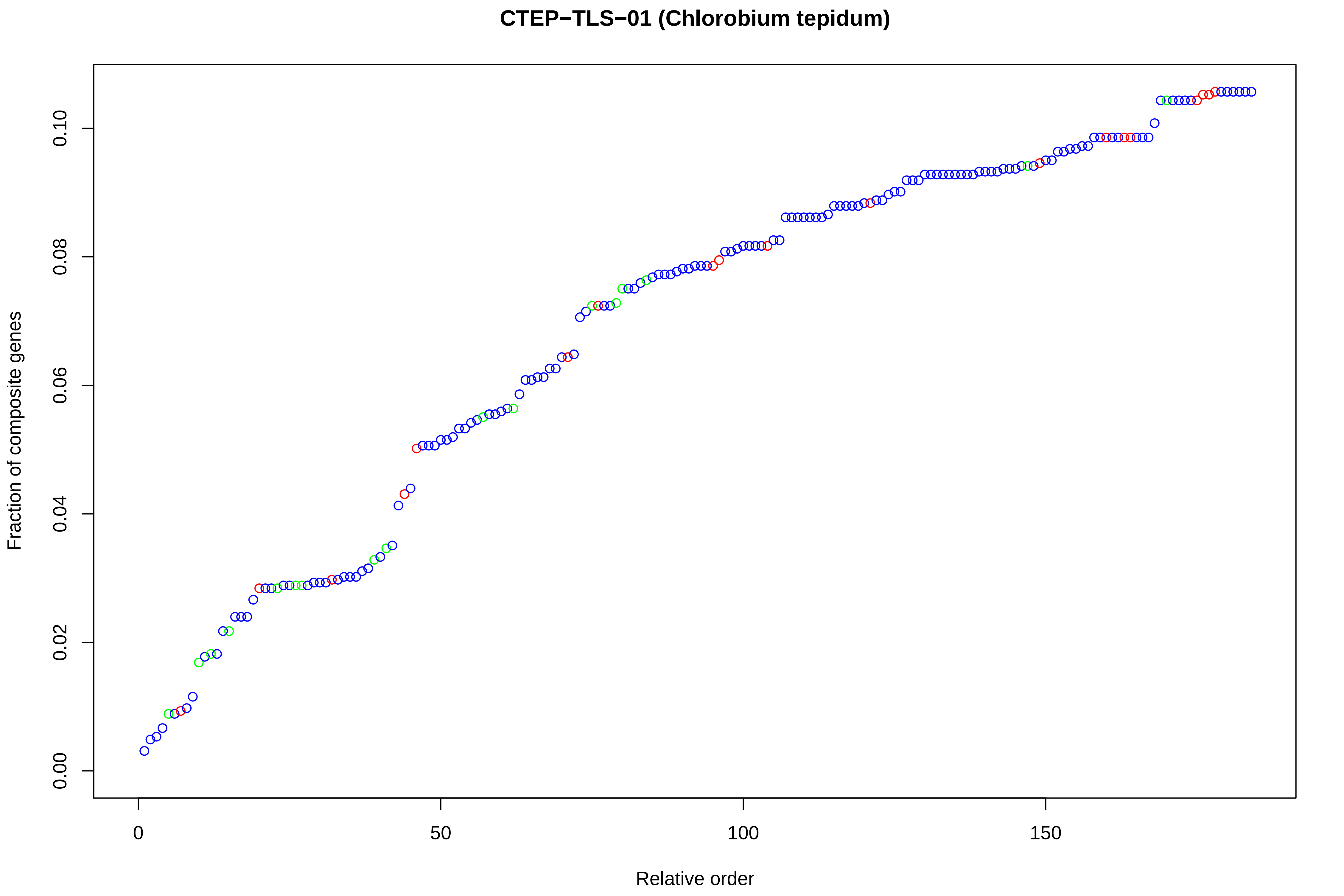

CTEP−TLS−01 (Chlorobium tepidum)
Fraction of composite genes
Relative order
0.00
0.02
0.04
0.06
0.08
0.10
0
50
100
150

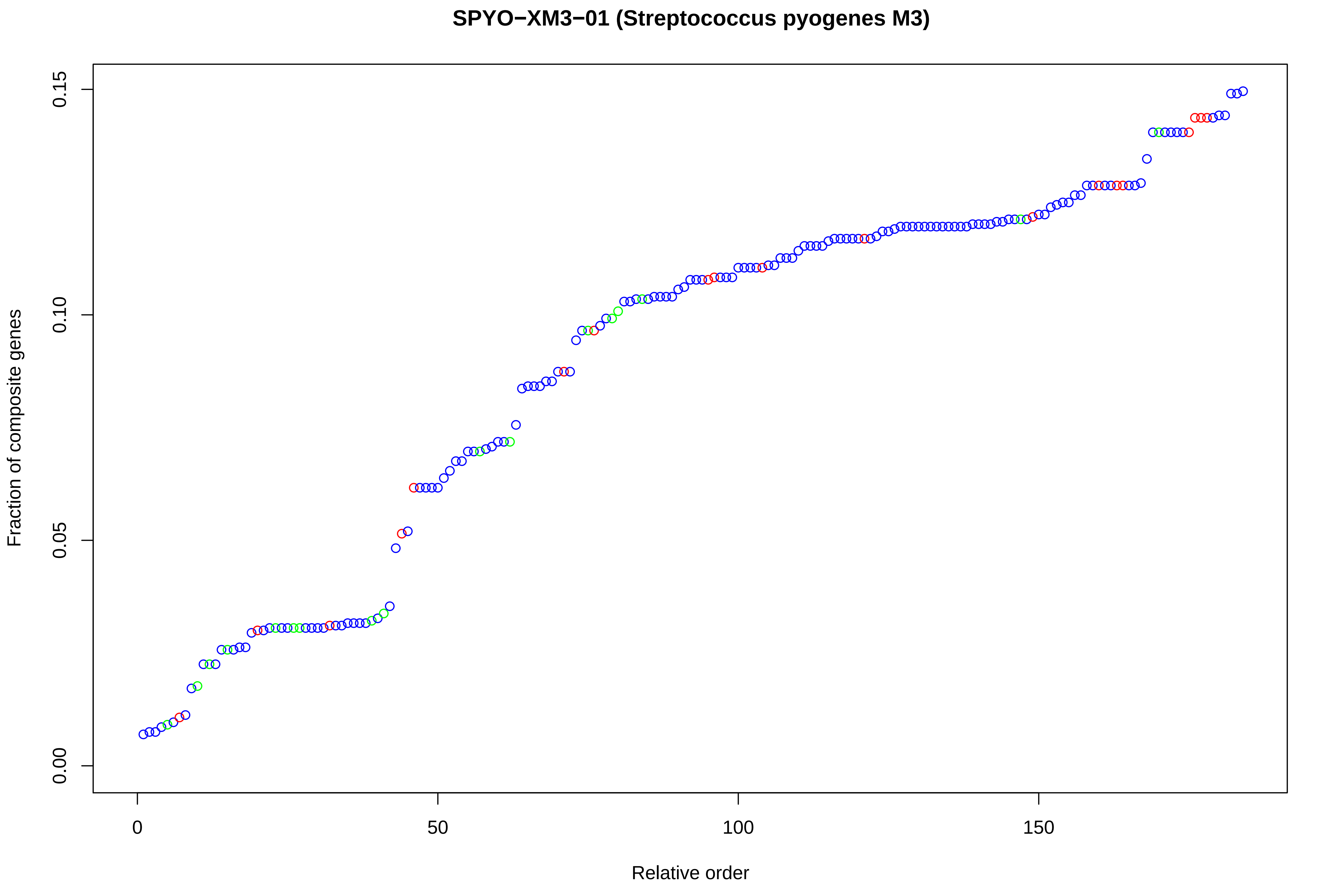
SPYO−XM3−01 (Streptococcus pyogenes M3)
Fraction of composite genes
0.00
0.05
0.10
0.15
0
50
100
150
Relative order

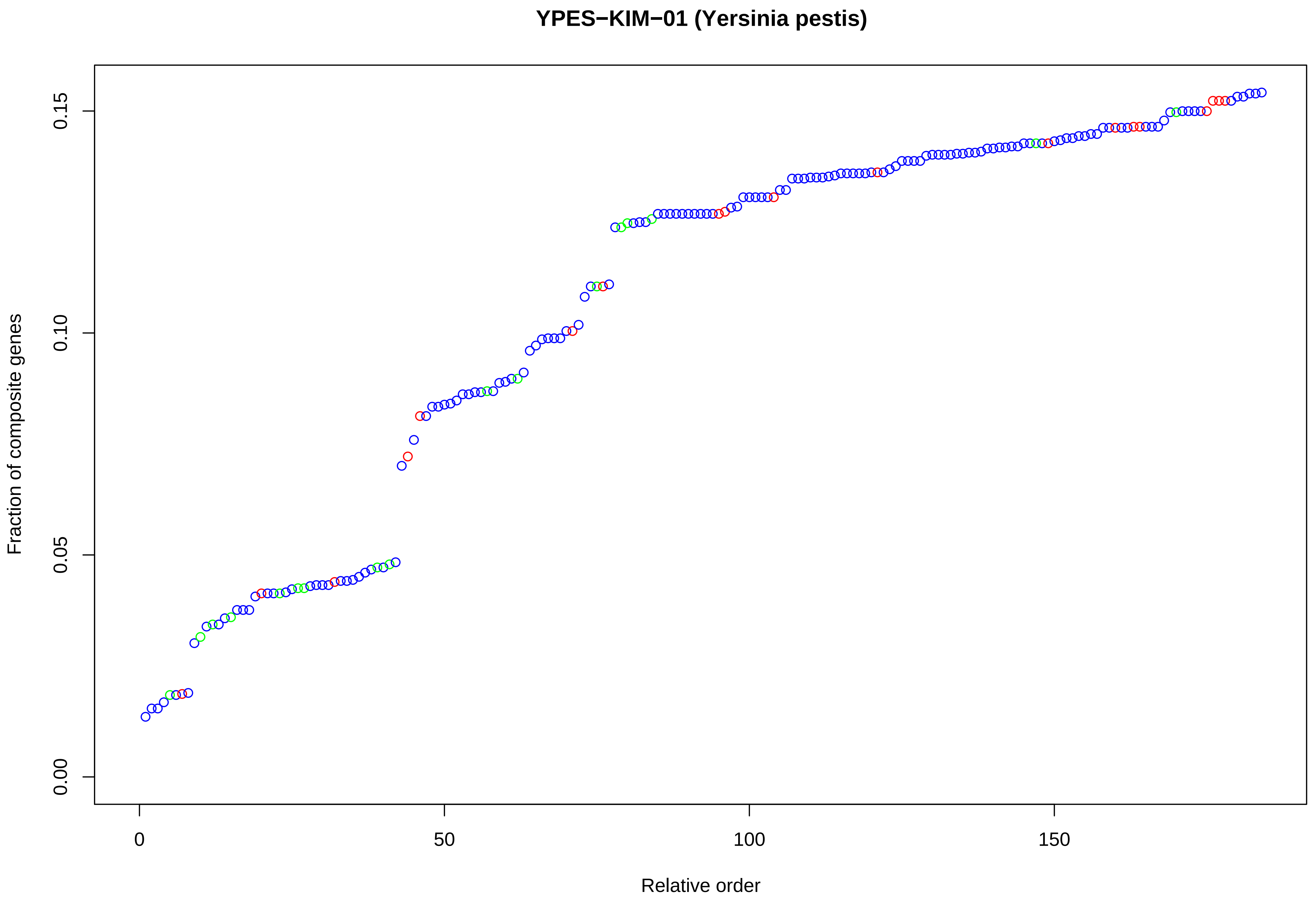
YPES−KIM−01 (Yersinia pestis)
Fraction of composite genes
0.00
0.05
0.10
0.15
0
50
100
150
Relative order

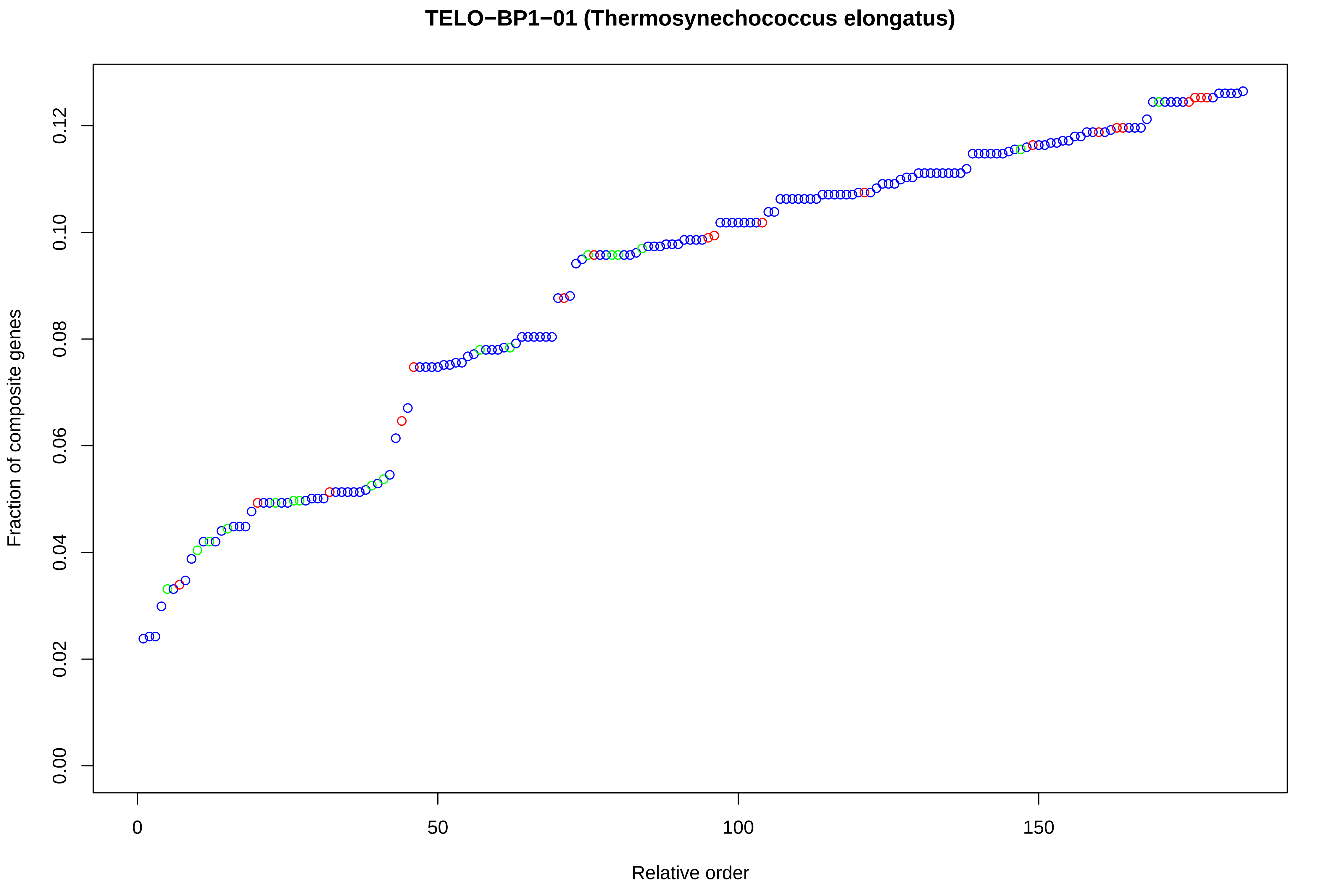

TELO−BP1−01 (Thermosynechococcus elongatus)
Fraction of composite genes
0.00
0.02
0.04
0.06
0.08
0.10
0.12
Relative order
0
50
100
150

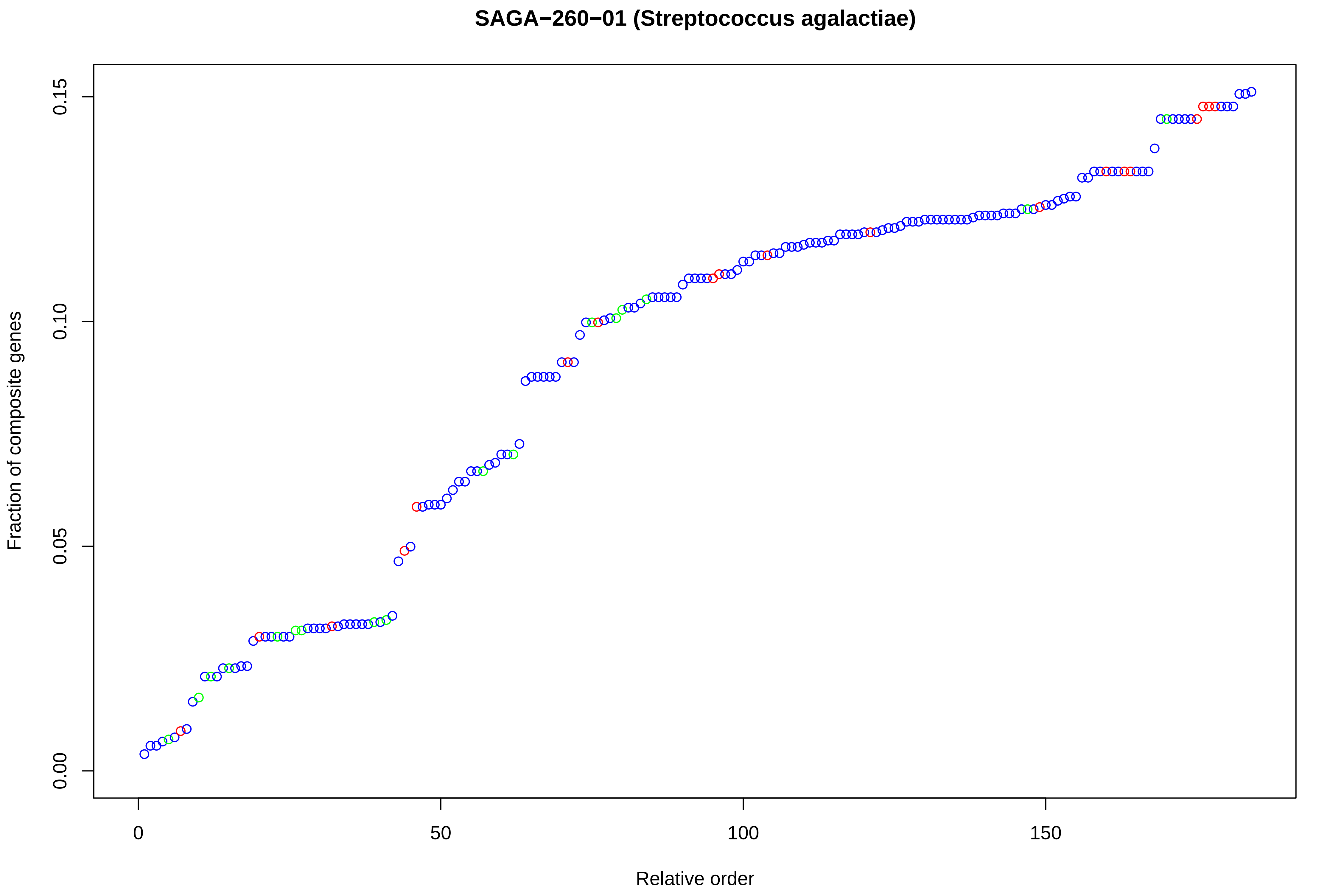
SAGA−260−01 (Streptococcus agalactiae)
Fraction of composite genes
0.00
0.05
0.10
0.15
Relative order
0
50
100
150

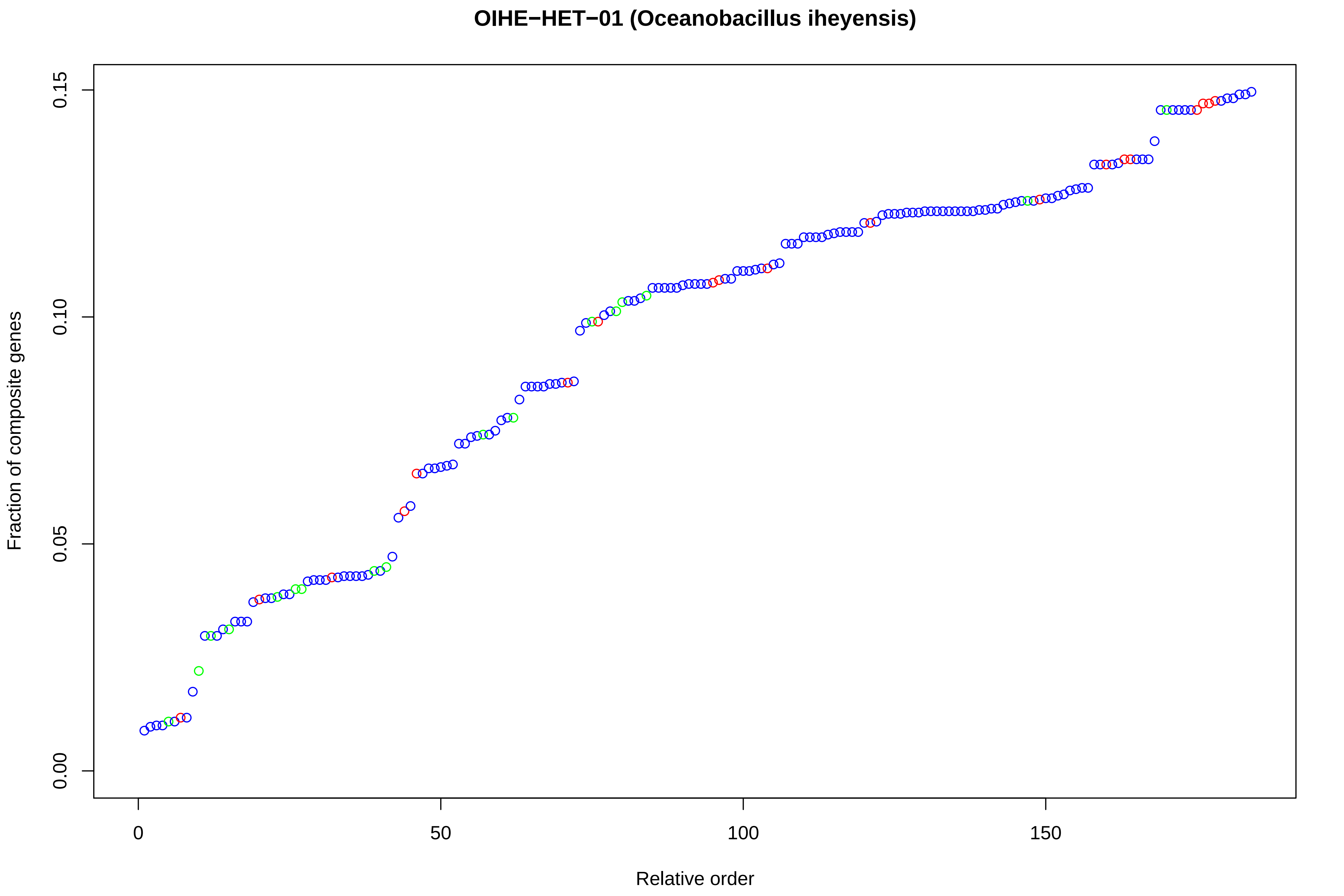

OIHE−HET−01 (Oceanobacillus iheyensis)
Fraction of composite genes
0.00
0.05
0.10
0.15
0
50
100
150
Relative order

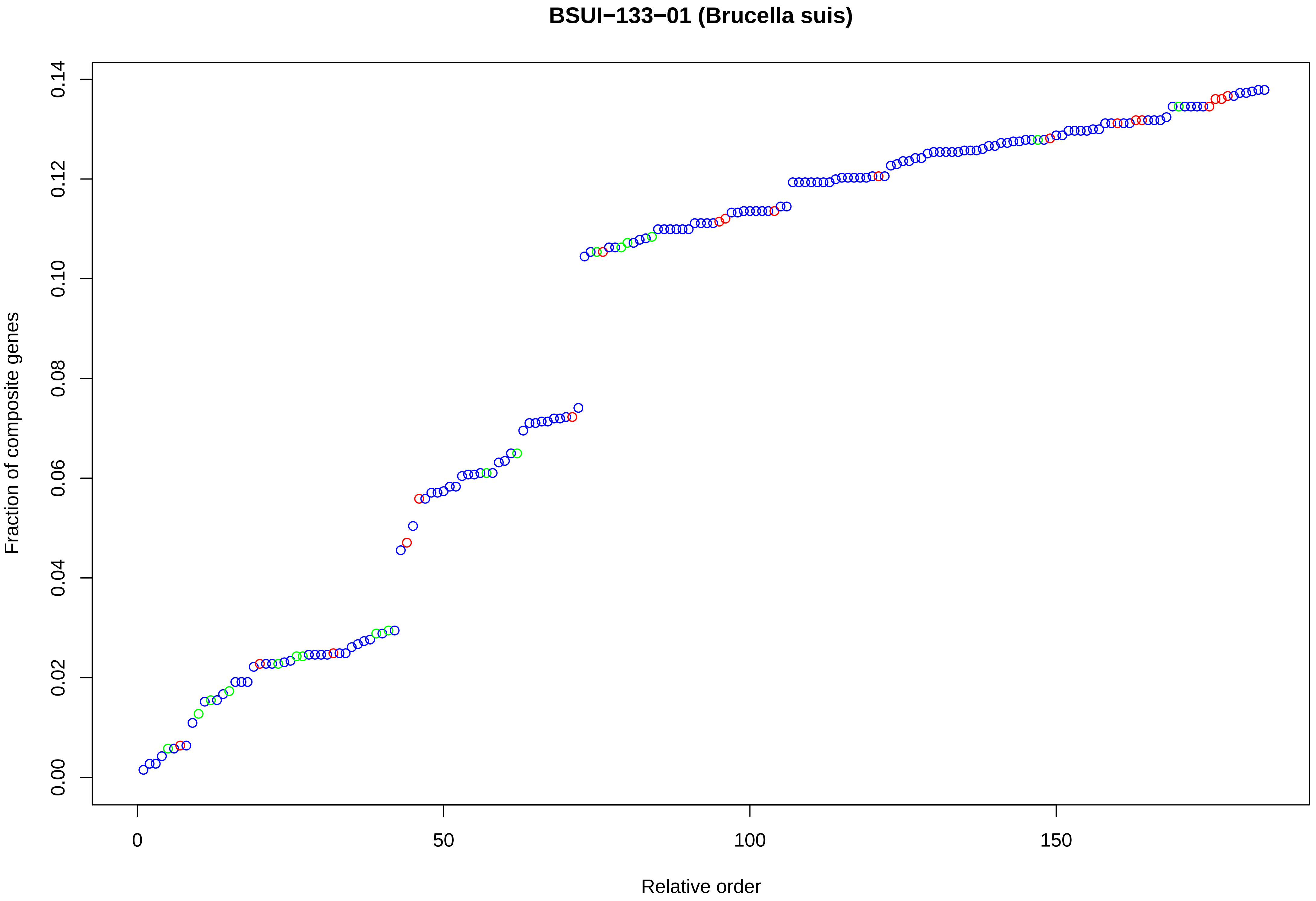

BSUI−133−01 (Brucella suis)
Fraction of composite genes
0.00
0.02
0.04
0.06
0.08
0.10
0.12
0.14
0
50
100
150
Relative order

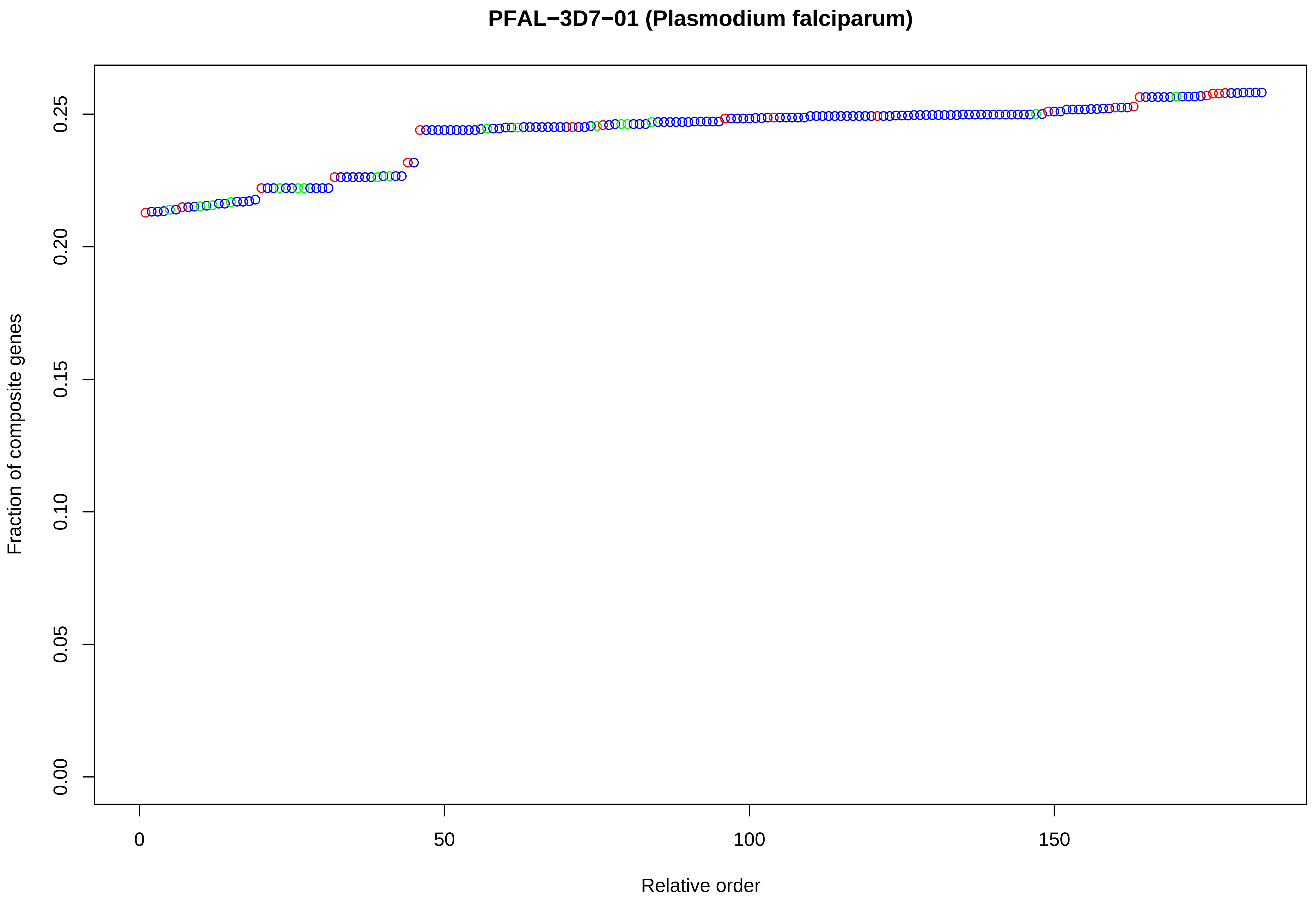
PFAL−3D7−01 (Plasmodium falciparum)
Fraction of composite genes
0.00
0.05
0.10
0.15
0.20
0.25
0
50
100
150
Relative order

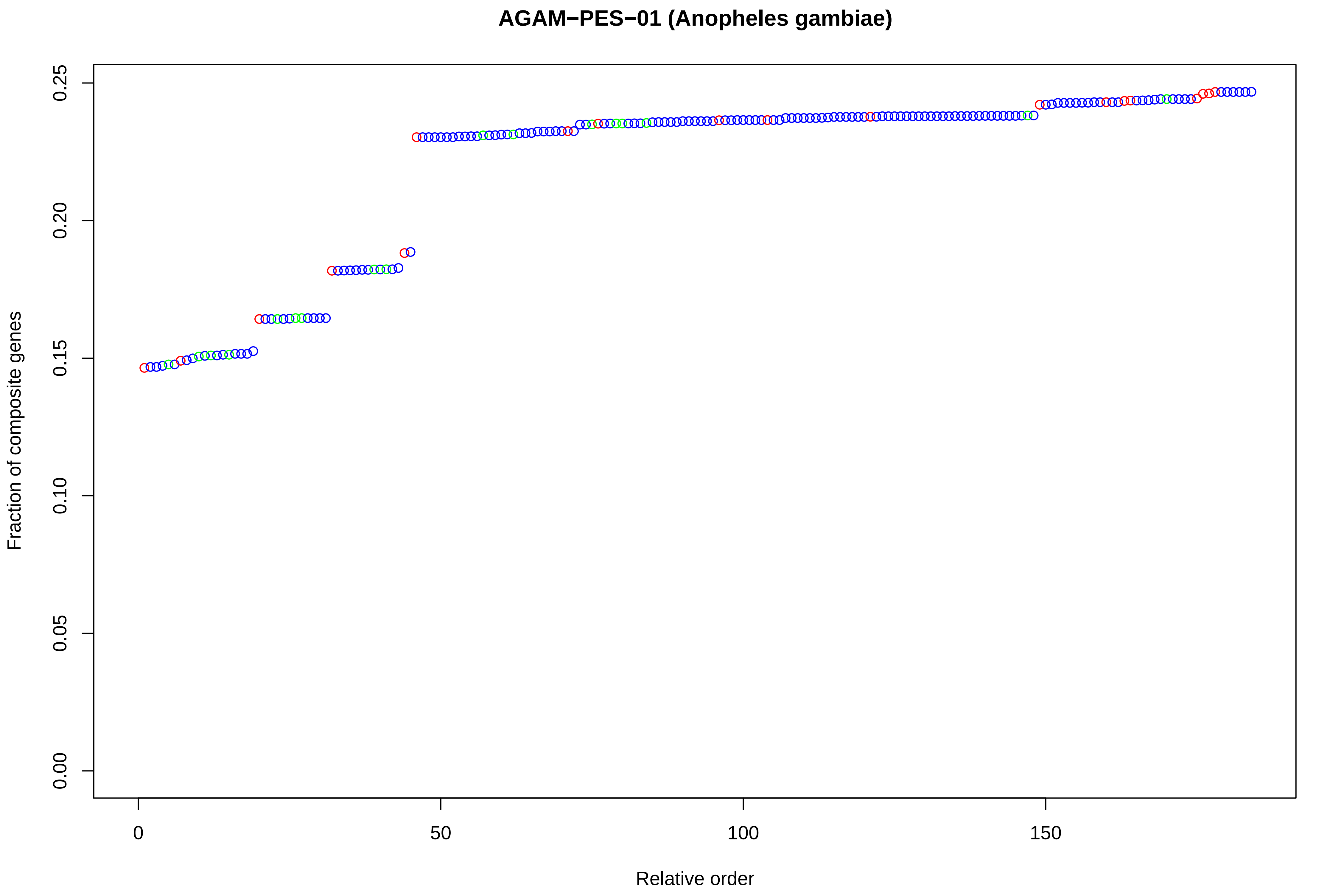

AGAM−PES−01 (Anopheles gambiae)
Fraction of composite genes
0.00
0.05
0.10
0.15
0.20
0.25
Relative order
0
50
100
150

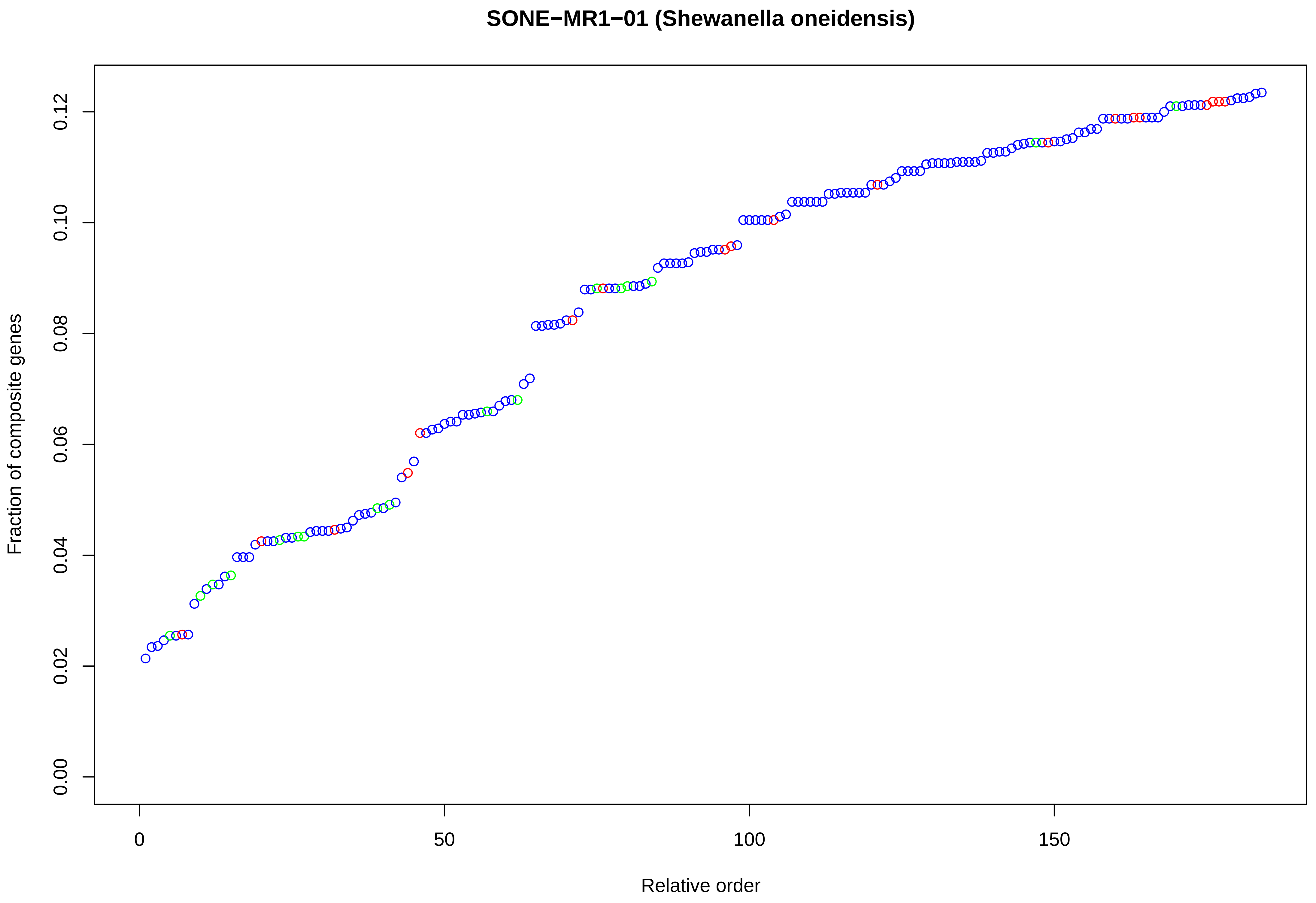

SONE−MR1−01 (Shewanella oneidensis)
Fraction of composite genes
Relative order
0.00
0.02
0.04
0.06
0.08
0.10
0.12
0
50
100
150

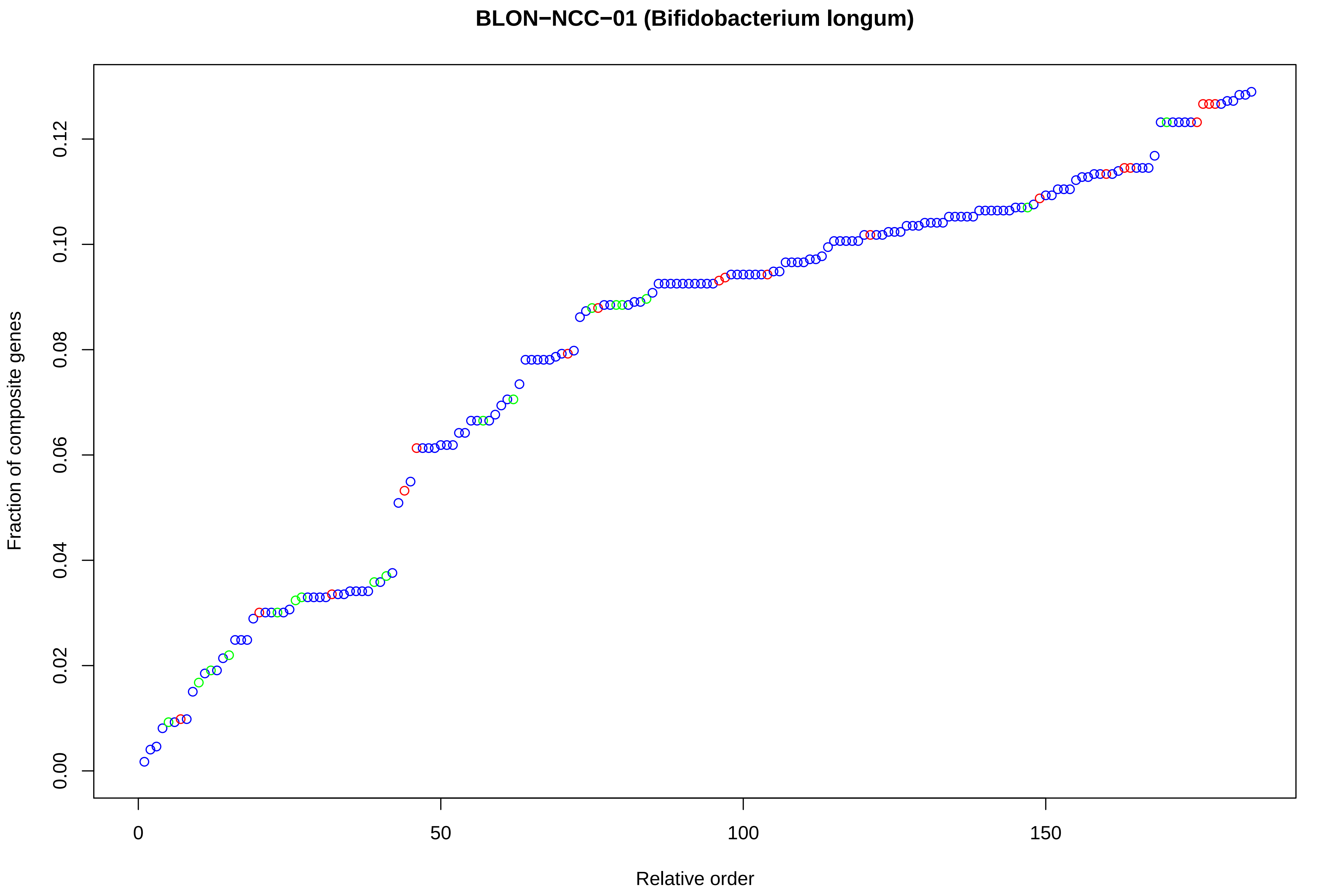
BLON−NCC−01 (Bifidobacterium longum)
Fraction of composite genes
0.00
0.02
0.04
0.06
0.08
0.10
0.12
0
50
100
150
Relative order

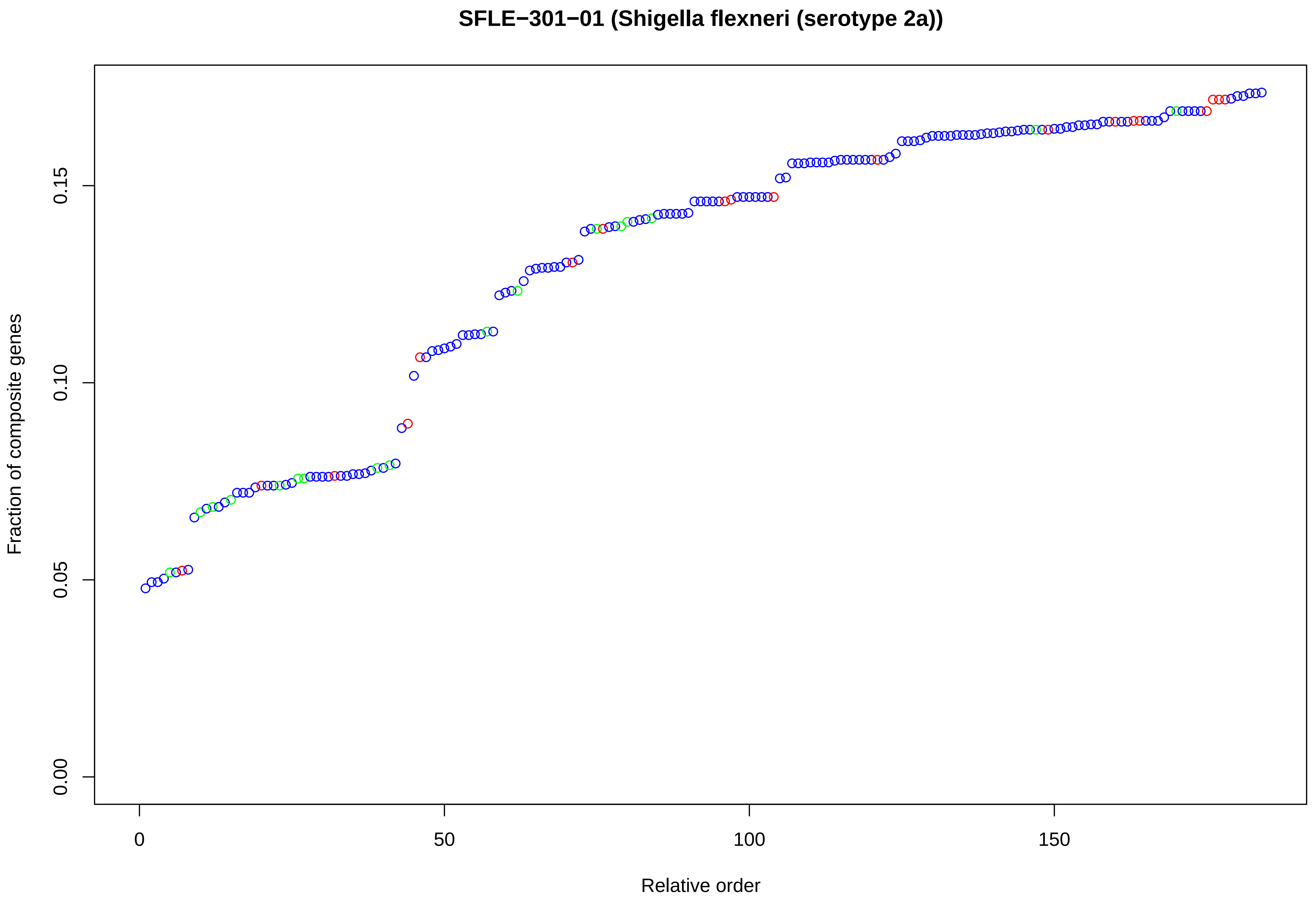
SFLE−301−01 (Shigella flexneri (serotype 2a))
Fraction of composite genes
0.00
0.05
0.10
0.15
0
50
100
150
Relative order

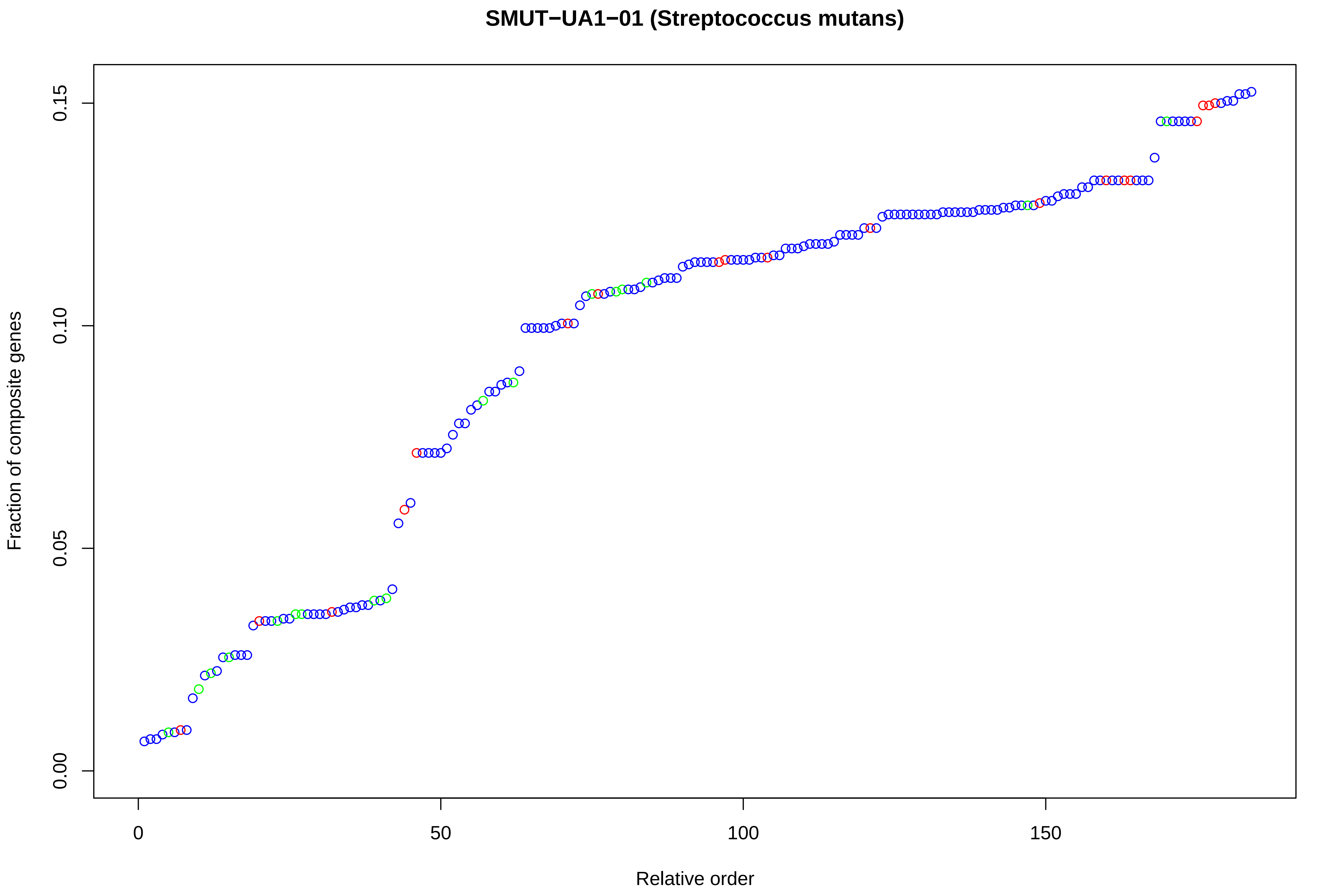
SMUT−UA1−01 (Streptococcus mutans)
Fraction of composite genes
0.00
0.05
0.10
0.15
0
50
100
150
Relative order

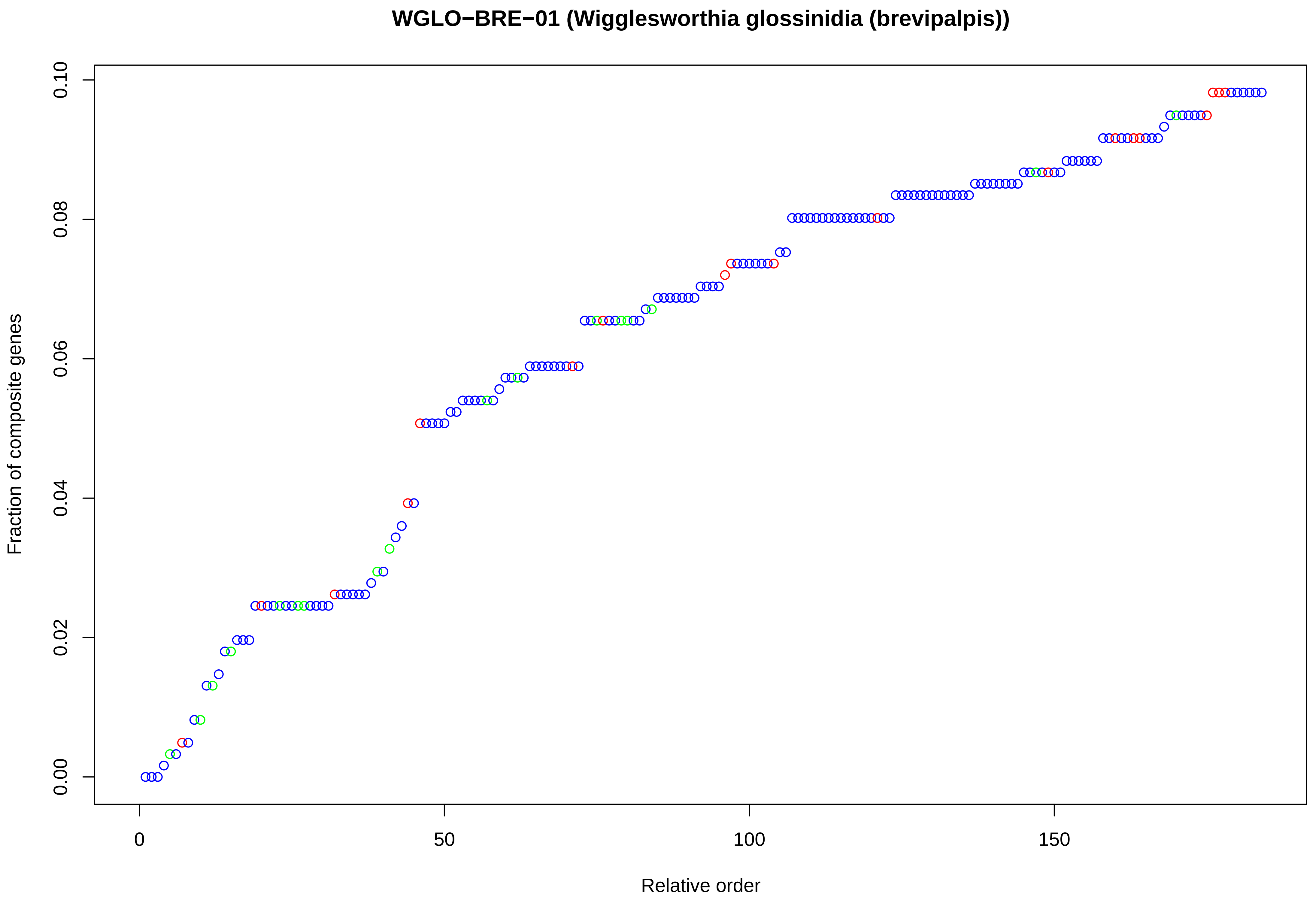

WGLO−BRE−01 (Wigglesworthia glossinidia (brevipalpis))
Fraction of composite genes
0.00
0.02
0.04
0.06
0.08
0.10
0
50
100
150
Relative order

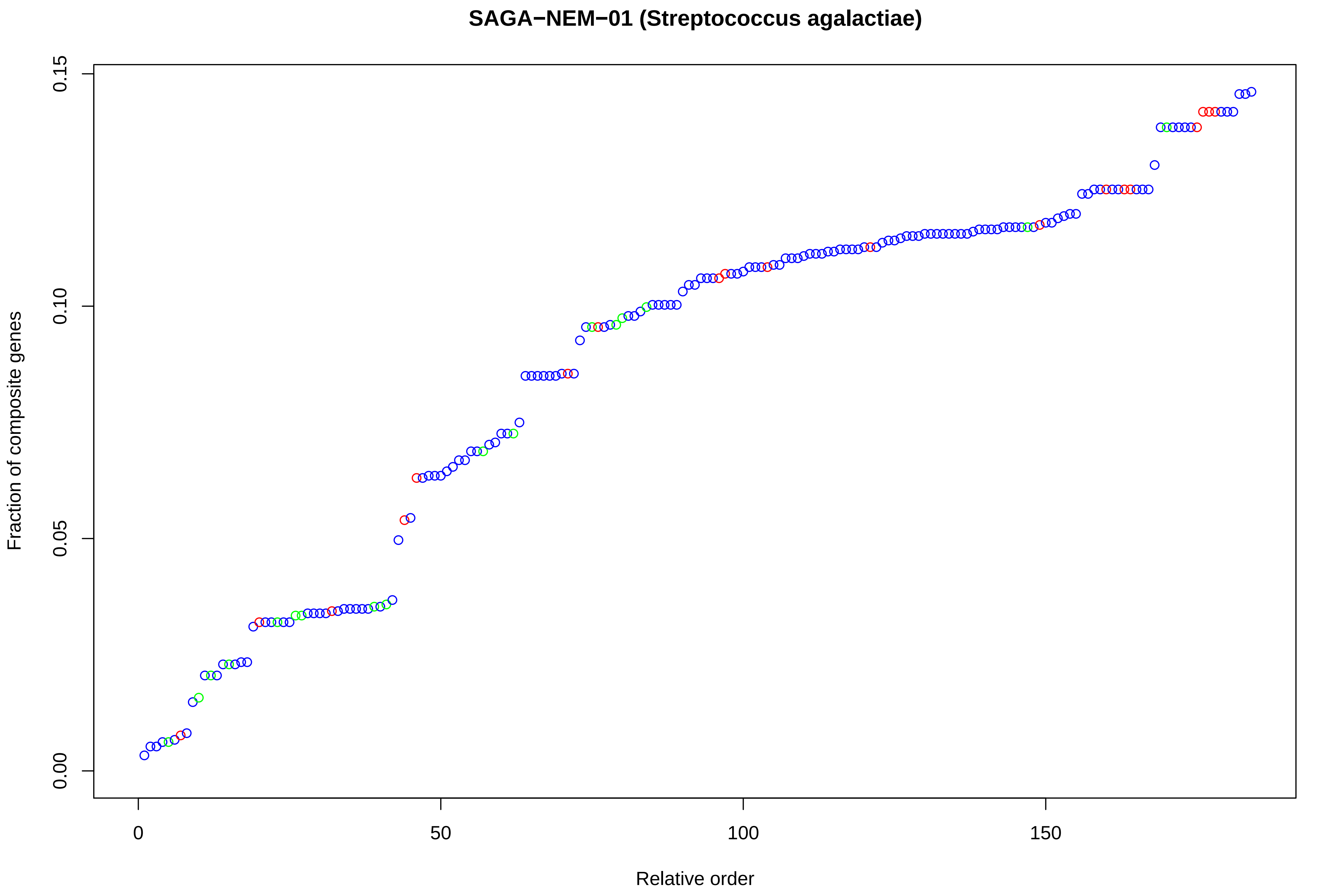
SAGA−NEM−01 (Streptococcus agalactiae)
Fraction of composite genes
0.00
0.05
0.10
0.15
0
50
100
150
Relative order

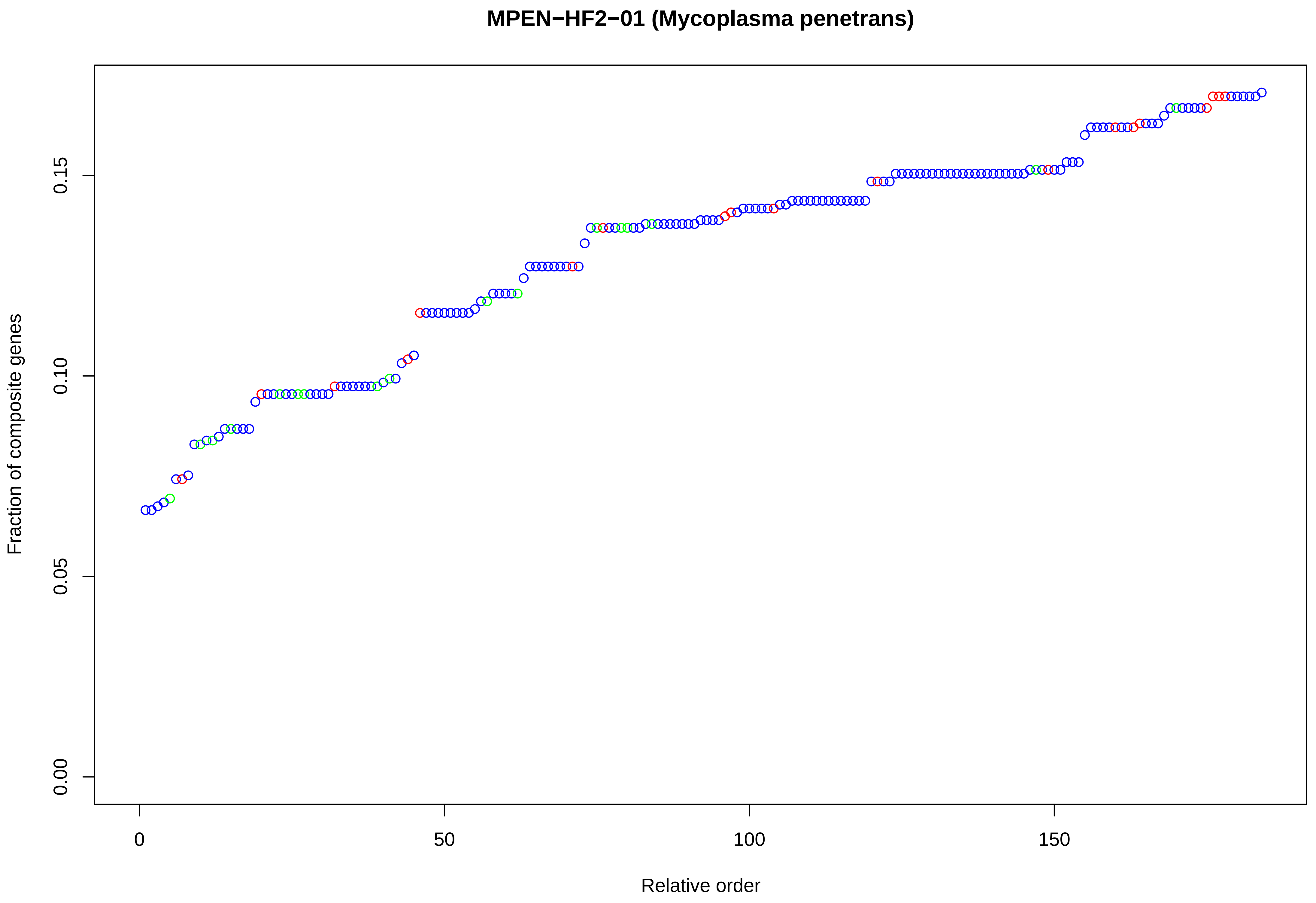
MPEN−HF2−01 (Mycoplasma penetrans)
Fraction of composite genes
0.00
0.05
0.10
0.15
0
50
100
150
Relative order

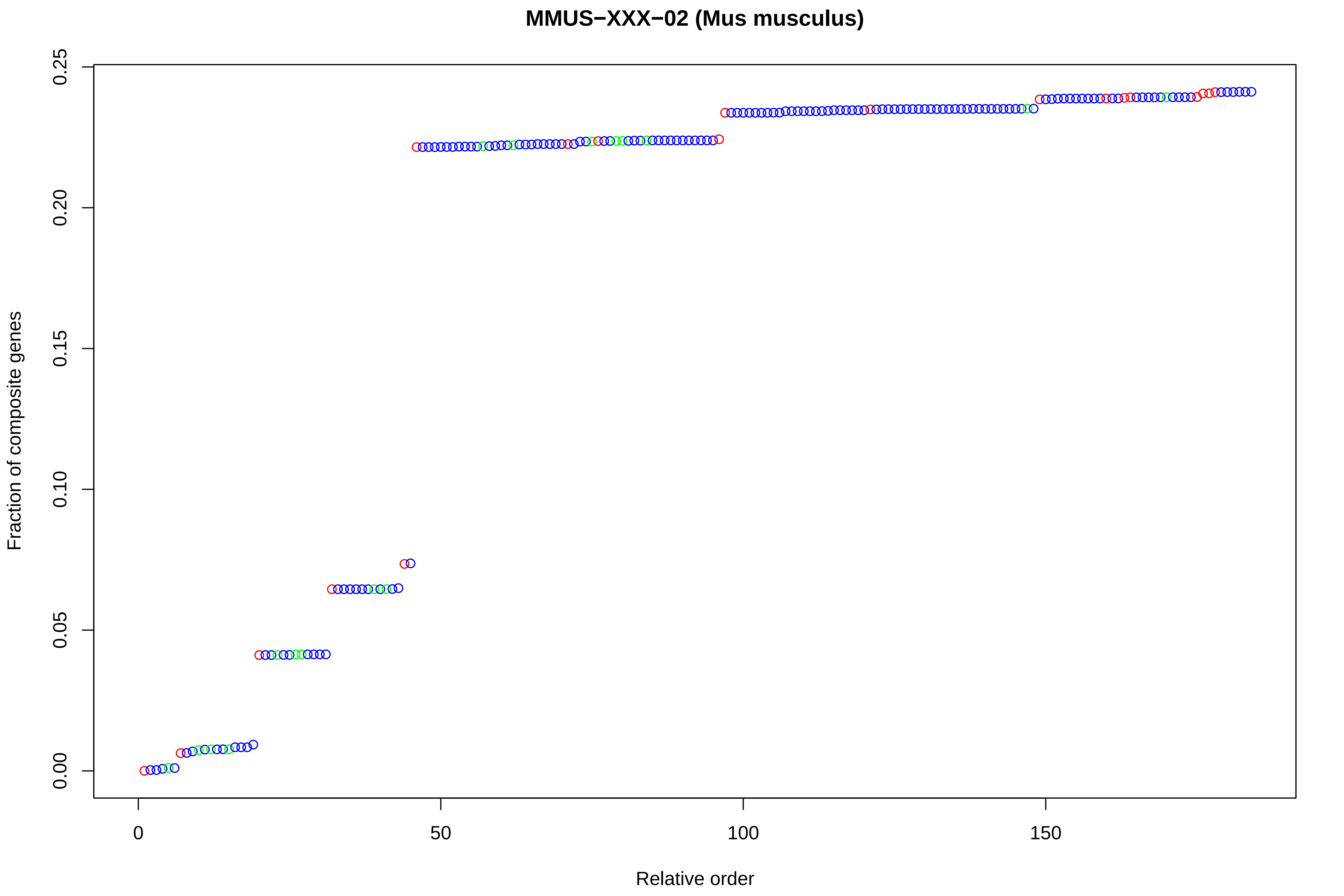
MMUS−XXX−02 (Mus musculus)
Fraction of composite genes
0.00
0.05
0.10
0.15
0.20
0.25
0
50
100
150
Relative order

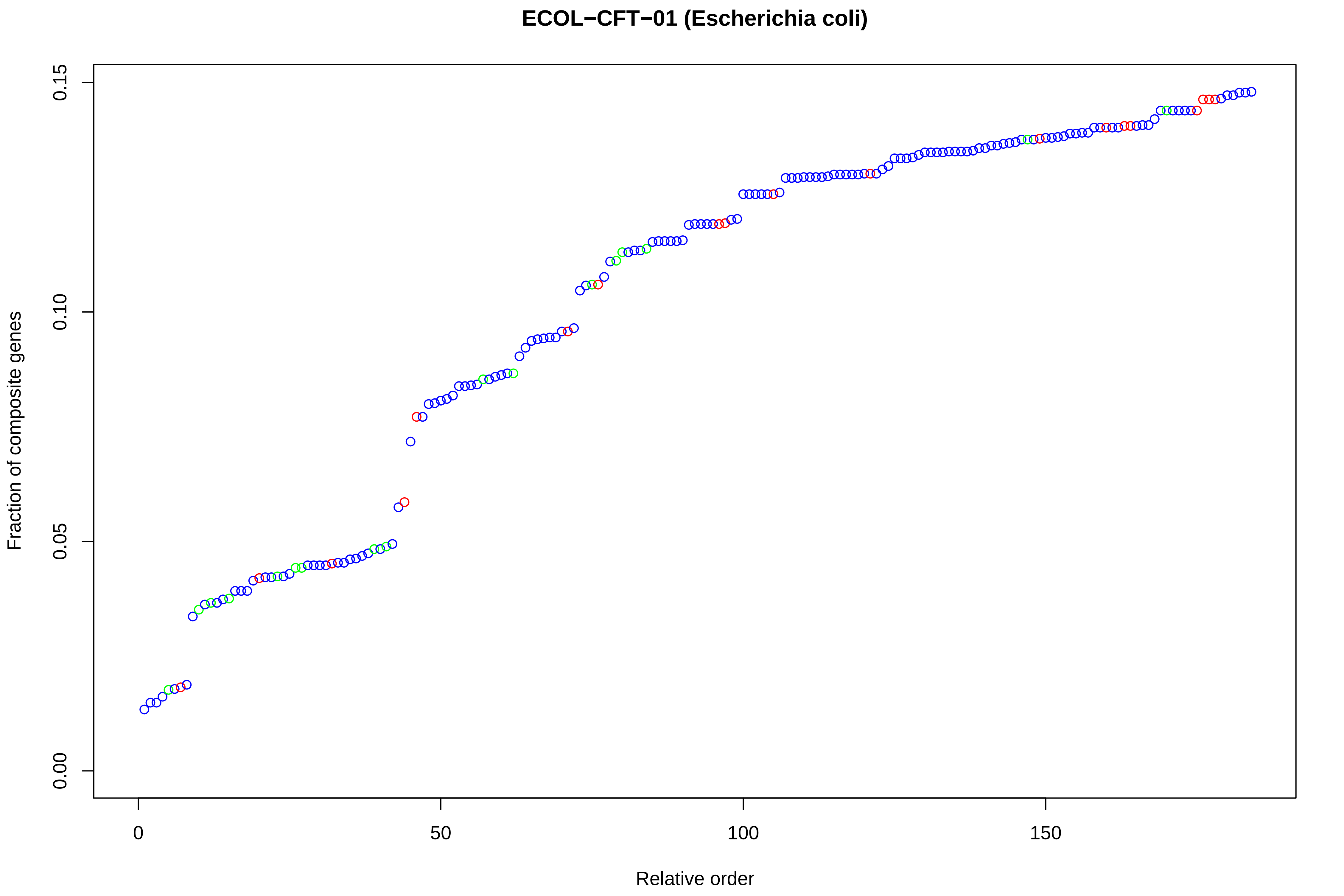

ECOL−CFT−01 (Escherichia coli)
Fraction of composite genes
0.00
0.05
0.10
0.15
0
50
100
150
Relative order

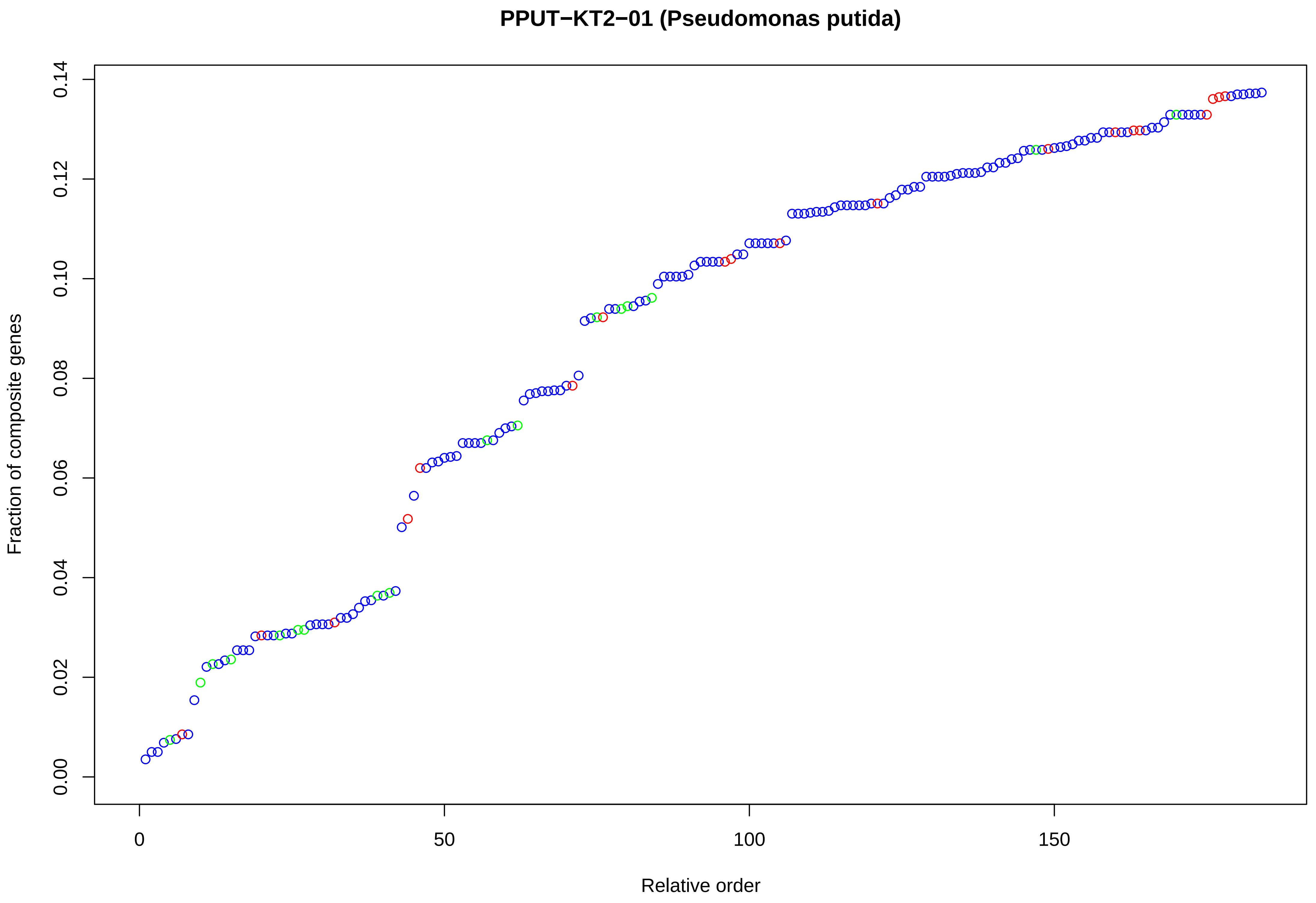

PPUT−KT2−01 (Pseudomonas putida)
Fraction of composite genes
0.00
0.02
0.04
0.06
0.08
0.10
0.12
0.14
Relative order
0
50
100
150

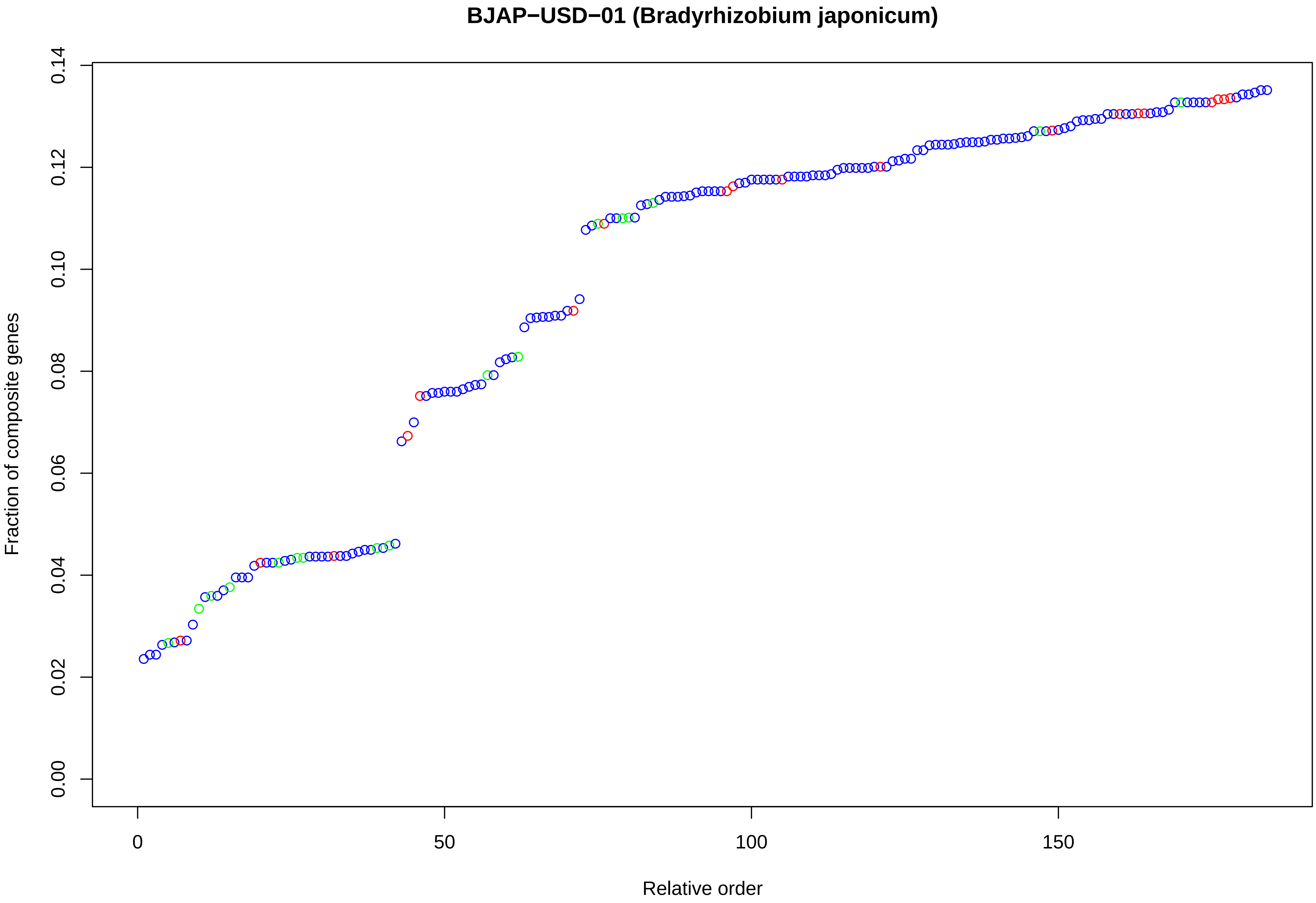
BJAP−USD−01 (Bradyrhizobium japonicum)
Fraction of composite genes
0.00
0.02
0.04
0.06
0.08
0.10
0.12
0.14
Relative order
0
50
100
150

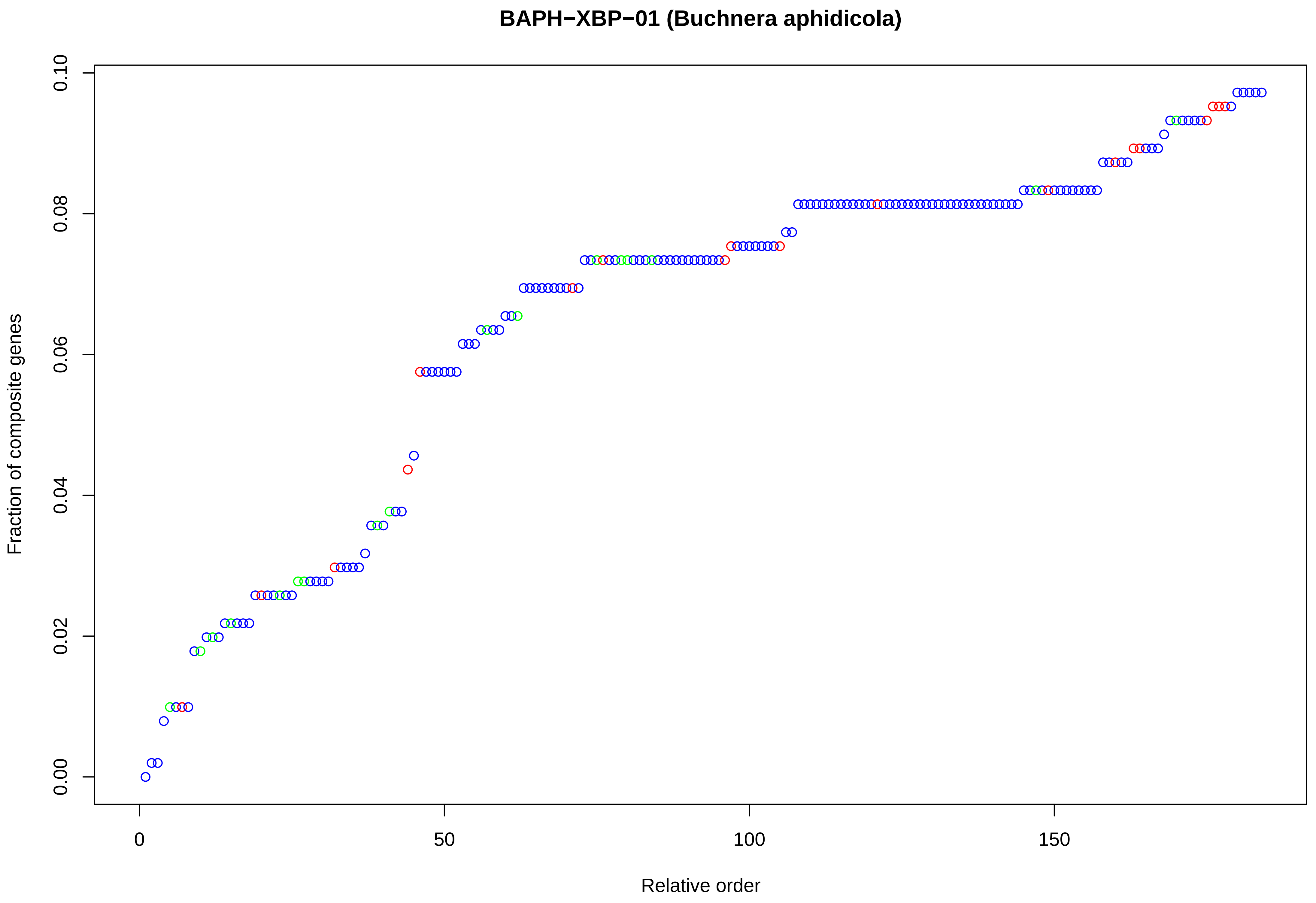
BAPH−XBP−01 (Buchnera aphidicola)
Fraction of composite genes
0.00
0.02
0.04
0.06
0.08
0.10
0
50
100
150
Relative order

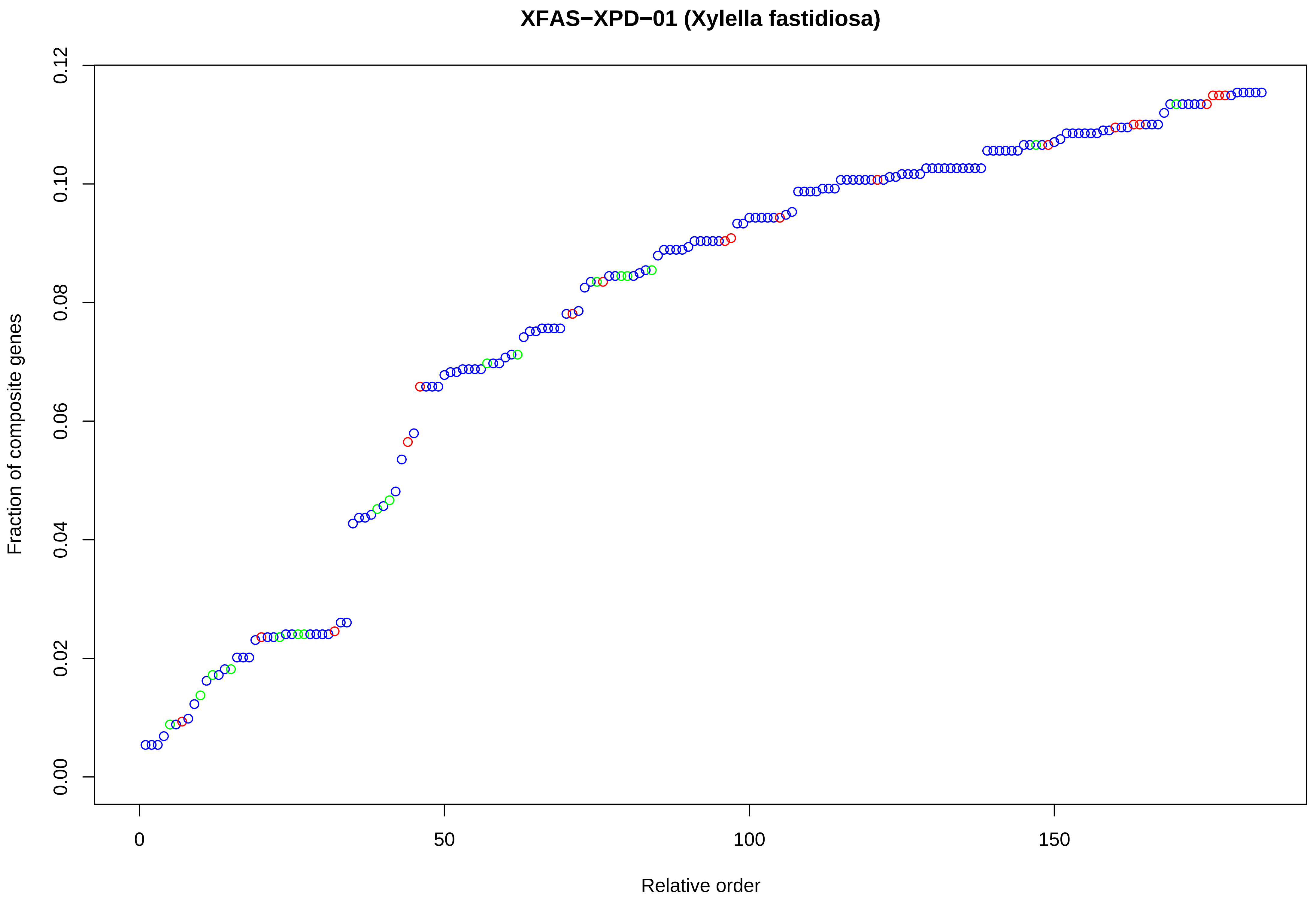
XFAS−XPD−01 (Xylella fastidiosa)
Fraction of composite genes
0.00
0.02
0.04
0.06
0.08
0.10
0.12
0
50
100
150
Relative order

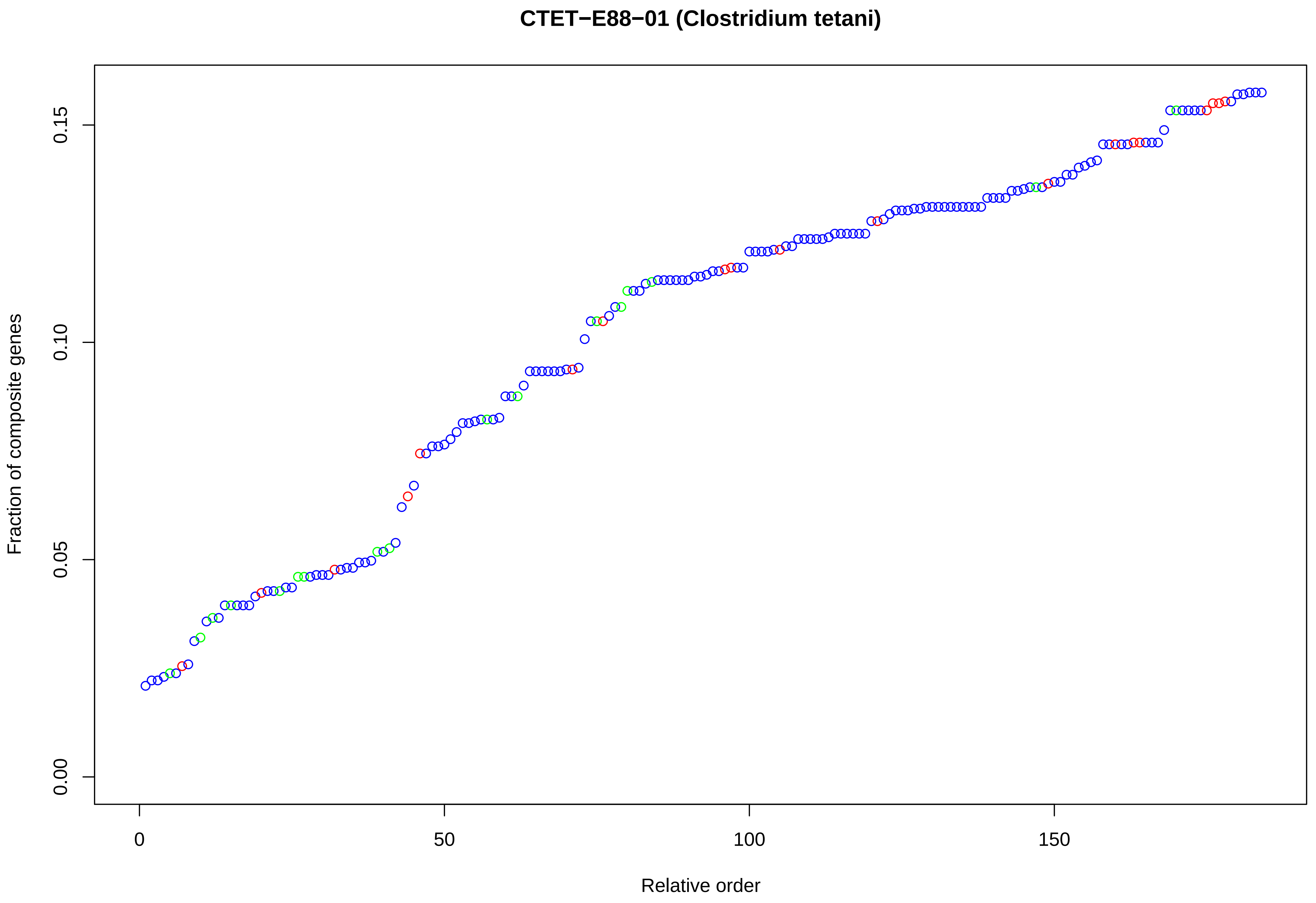

CTET−E88−01 (Clostridium tetani)
Fraction of composite genes
0.00
0.05
0.10
0.15
0
50
100
150
Relative order

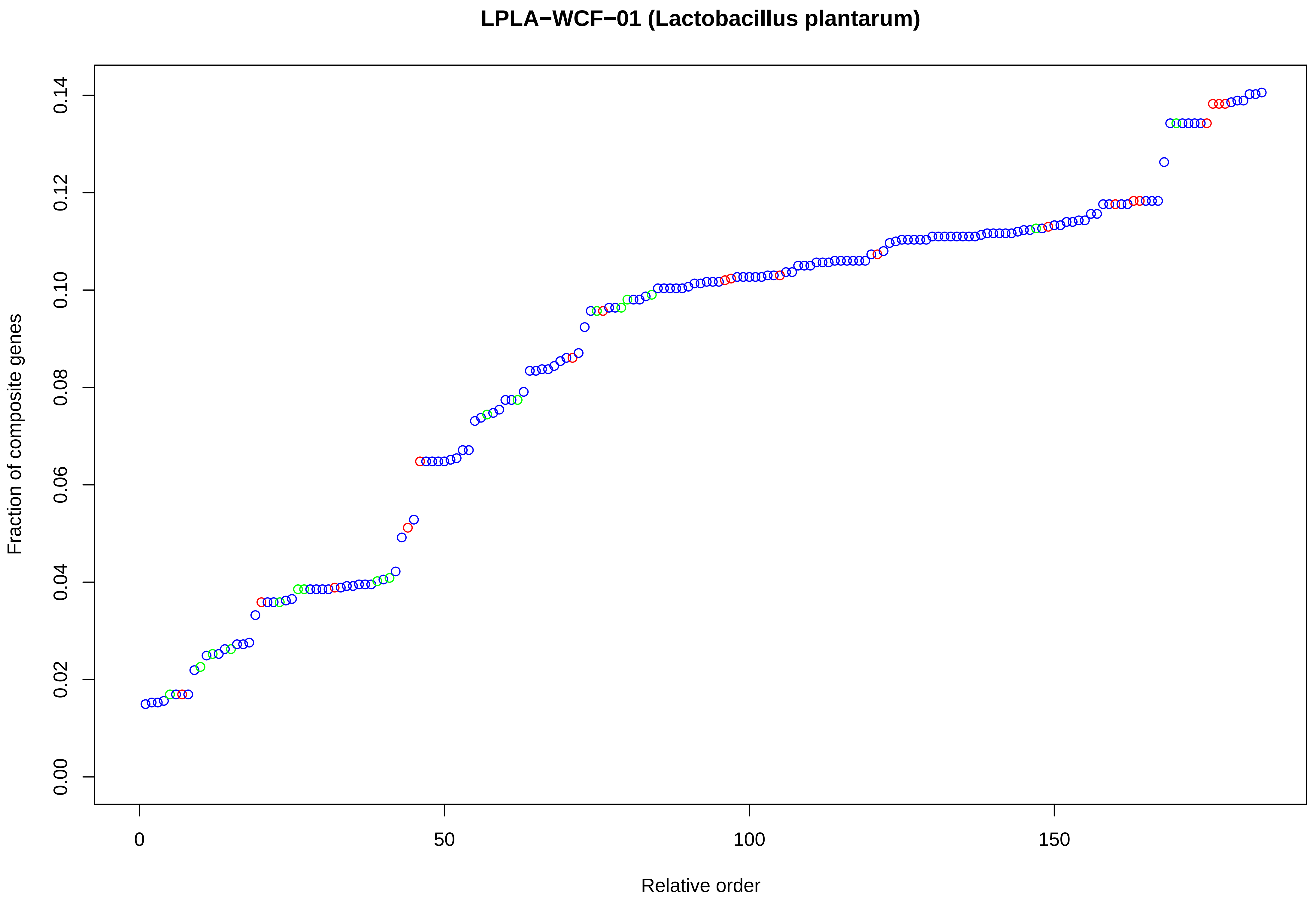

LPLA−WCF−01 (Lactobacillus plantarum)
Fraction of composite genes
Relative order
0.00
0.02
0.04
0.06
0.08
0.10
0.12
0.14
0
50
100
150

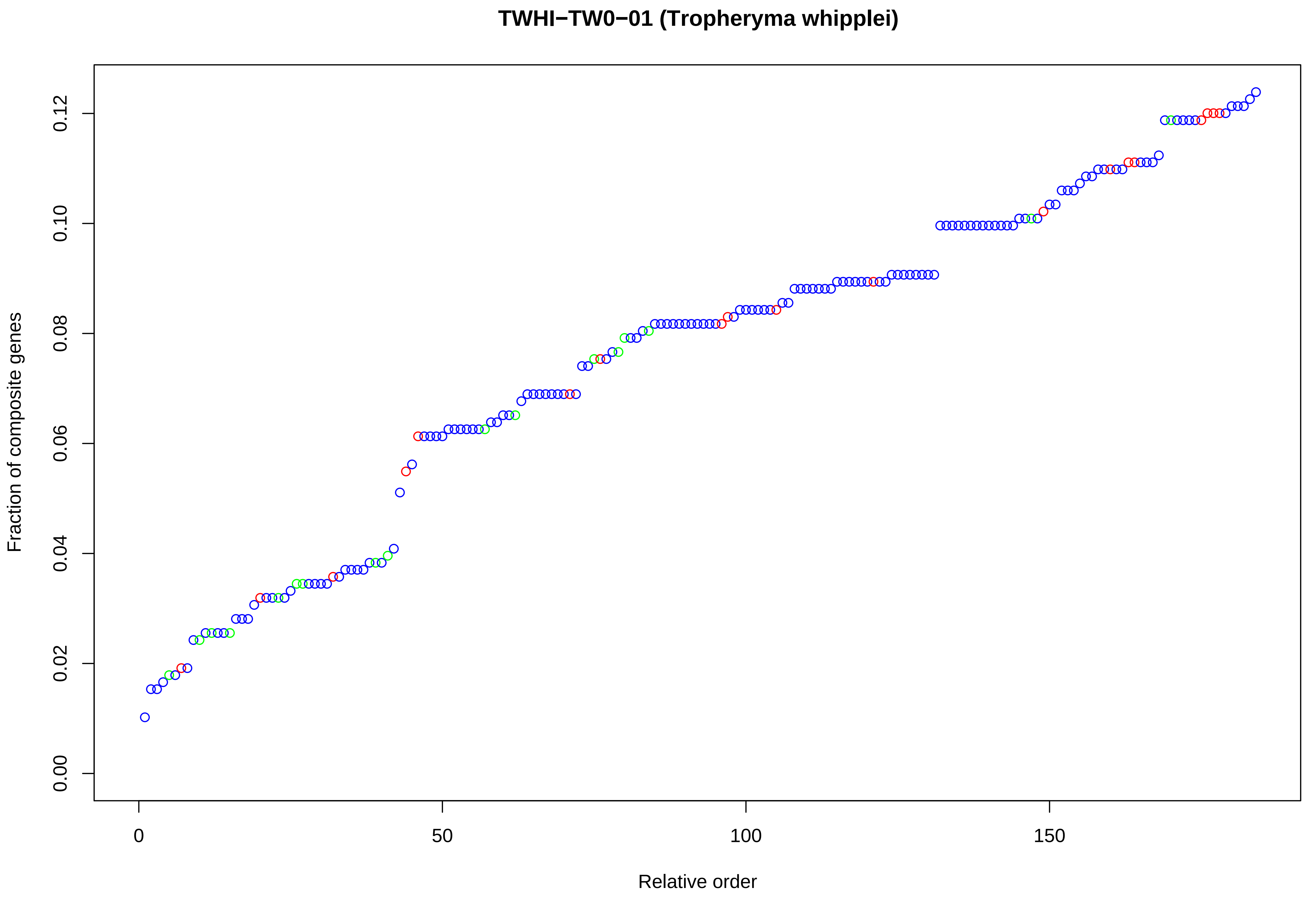

TWHI−TW0−01 (Tropheryma whipplei)
Fraction of composite genes
0.00
0.02
0.04
0.06
0.08
0.10
0.12
Relative order
0
50
100
150

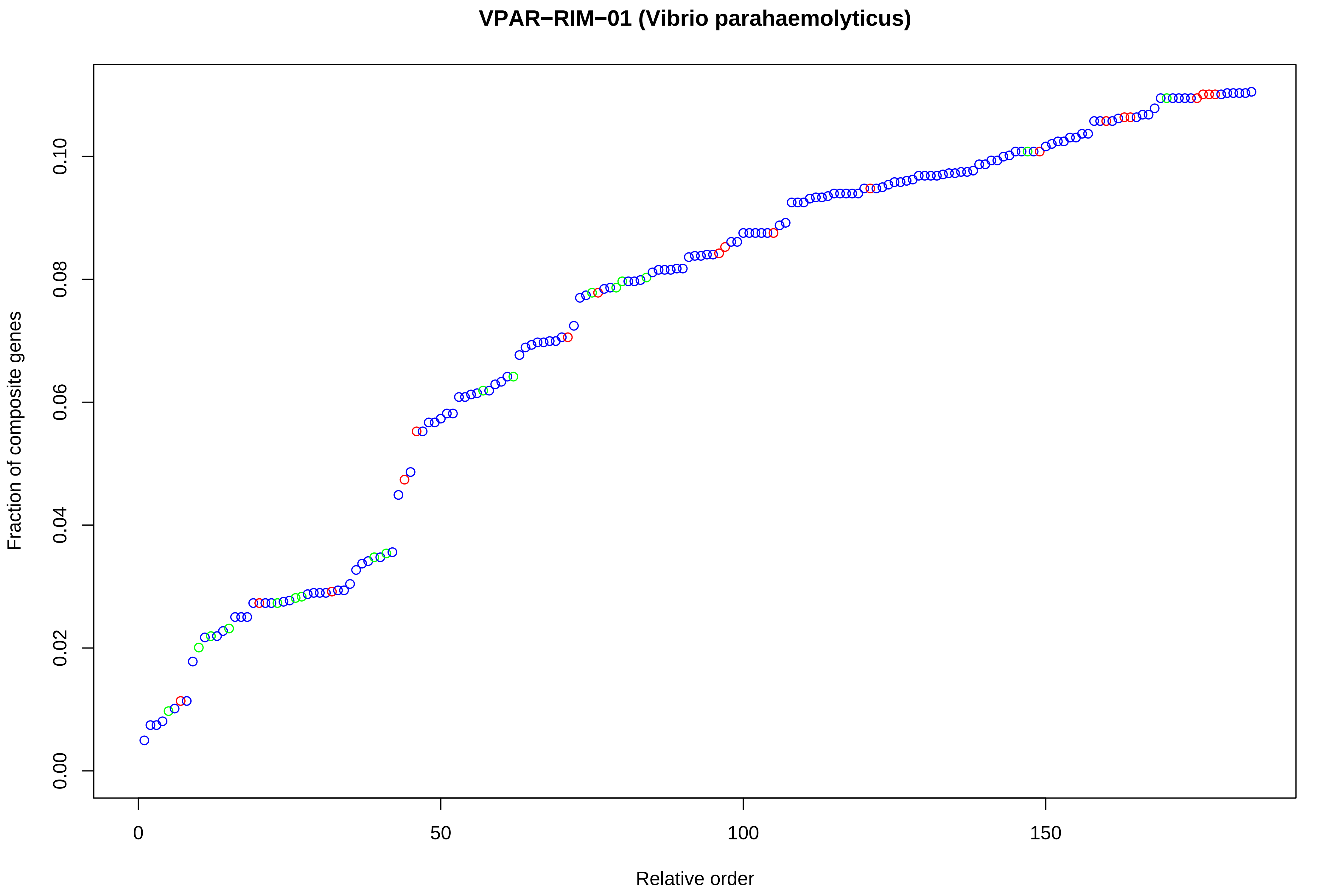
VPAR−RIM−01 (Vibrio parahaemolyticus)
Fraction of composite genes
0.00
0.02
0.04
0.06
0.08
0.10
Relative order
0
50
100
150

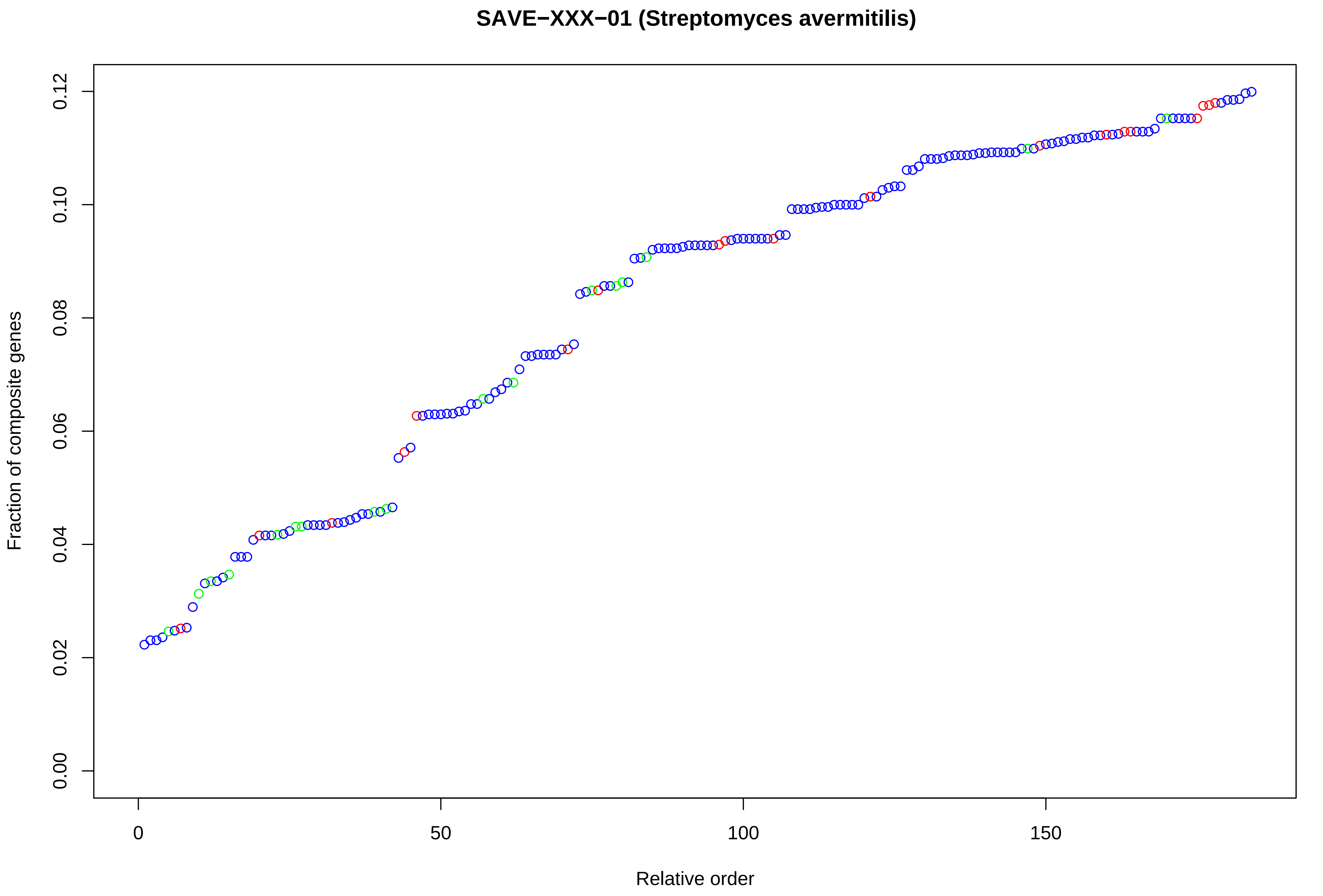

SAVE−XXX−01 (Streptomyces avermitilis)
Fraction of composite genes
Relative order
0.00
0.02
0.04
0.06
0.08
0.10
0.12
0
50
100
150

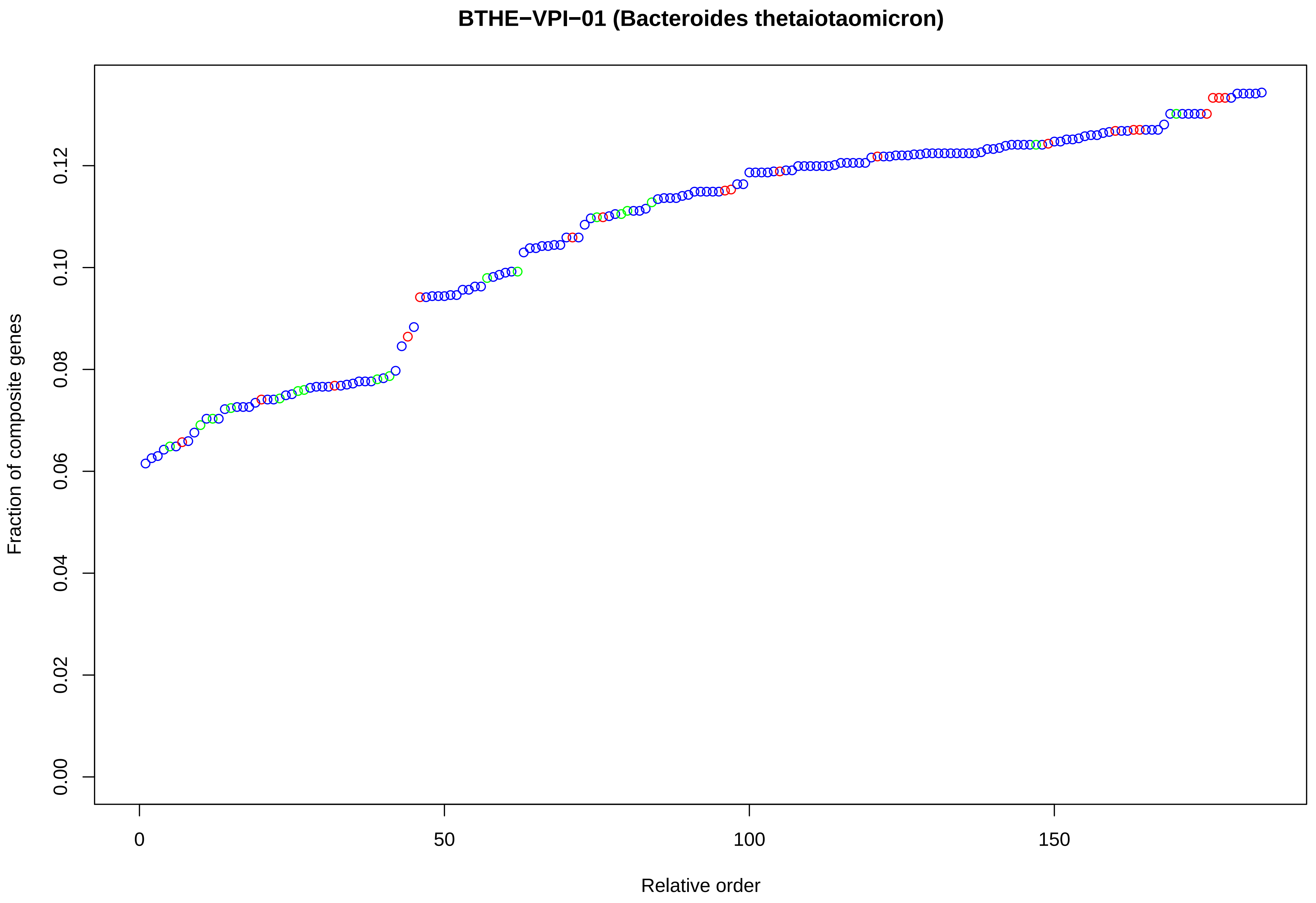
BTHE−VPI−01 (Bacteroides thetaiotaomicron)
Fraction of composite genes
Relative order
0.00
0.02
0.04
0.06
0.08
0.10
0.12
0
50
100
150

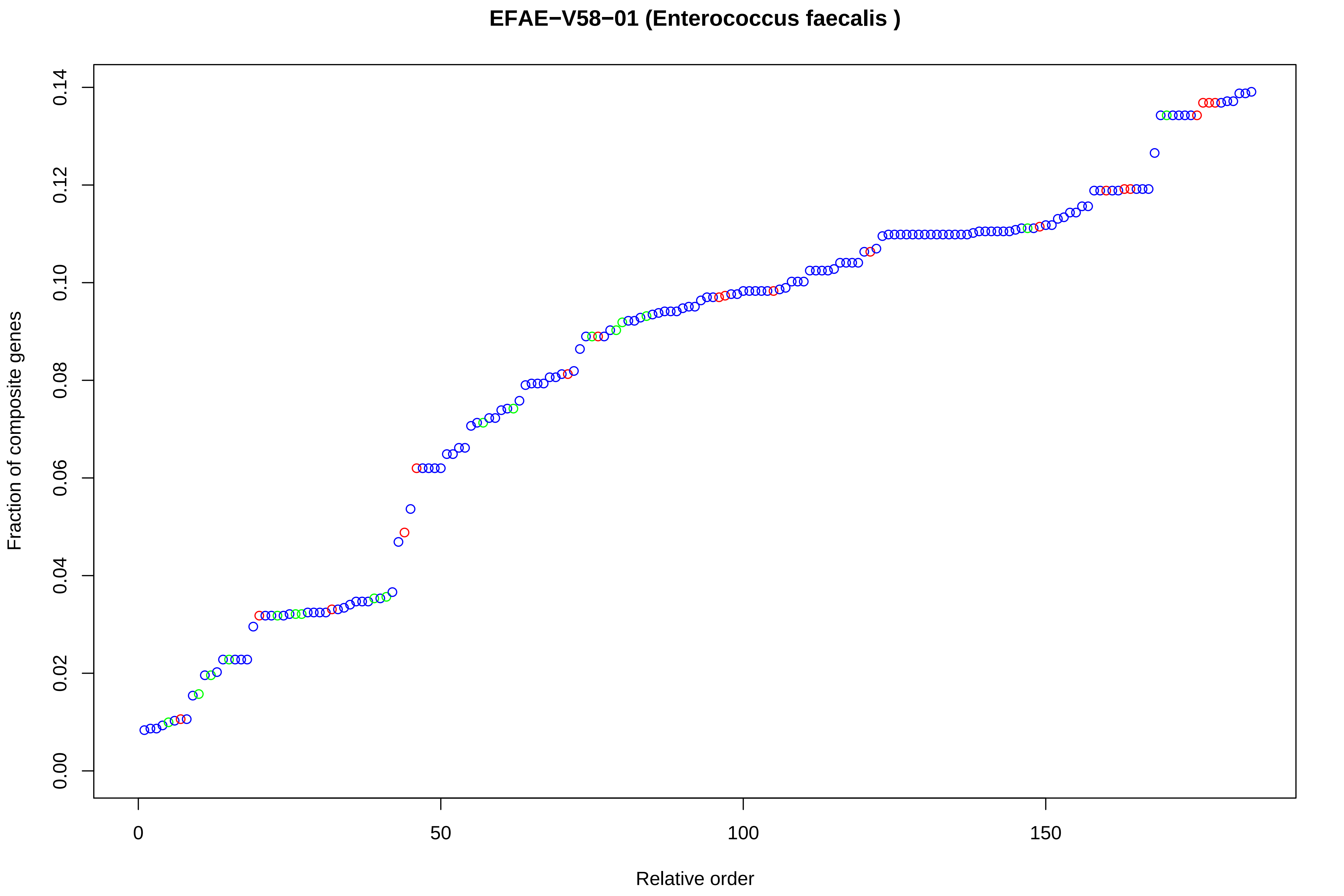

EFAE−V58−01 (Enterococcus faecalis)
Fraction of composite genes
Relative order
0.00
0.02
0.04
0.06
0.08
0.10
0.12
0.14
0
50
100
150

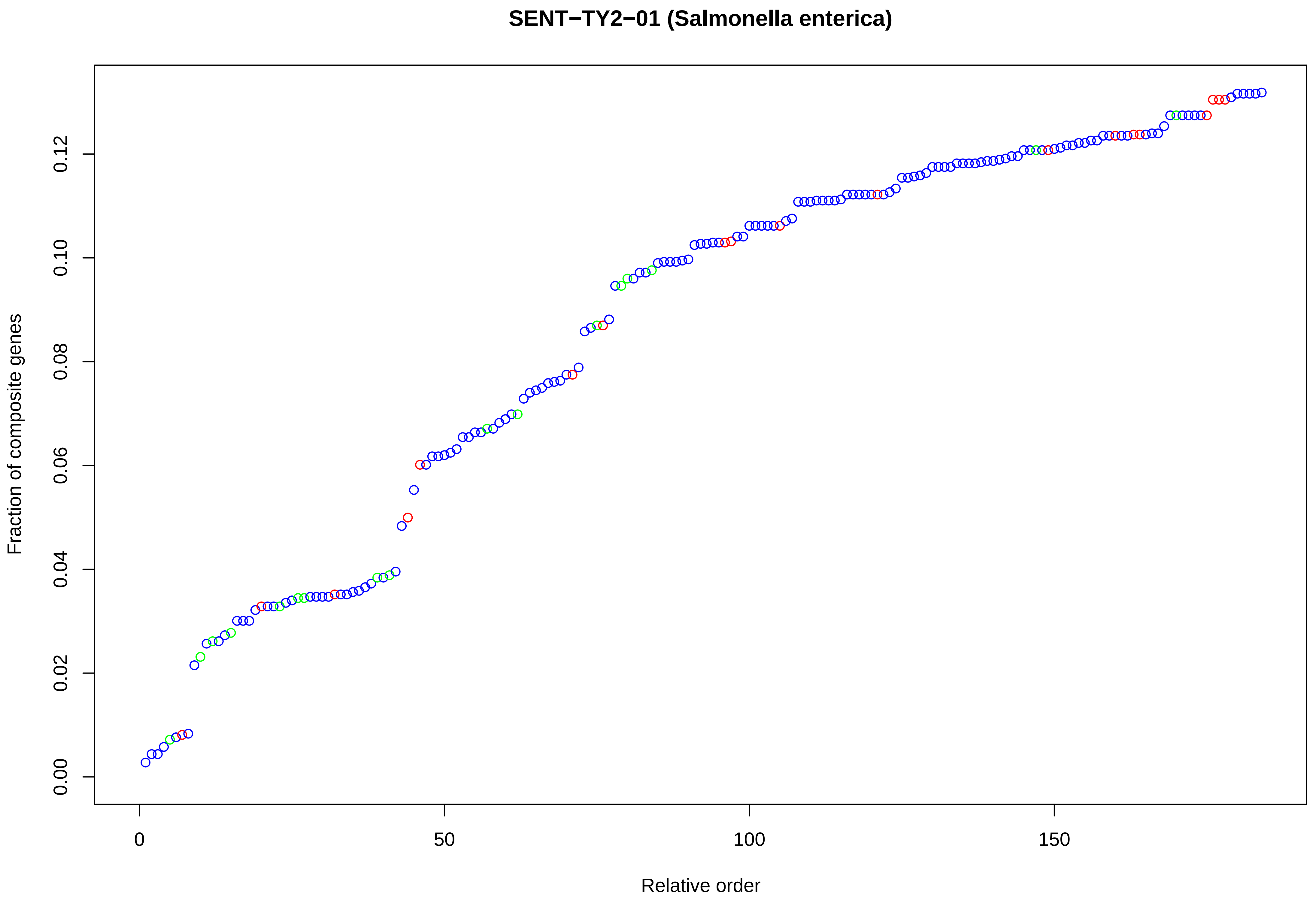

SENT−TY2−01 (Salmonella enterica)
Fraction of composite genes
Relative order
0.00
0.02
0.04
0.06
0.08
0.10
0.12
0
50
100
150

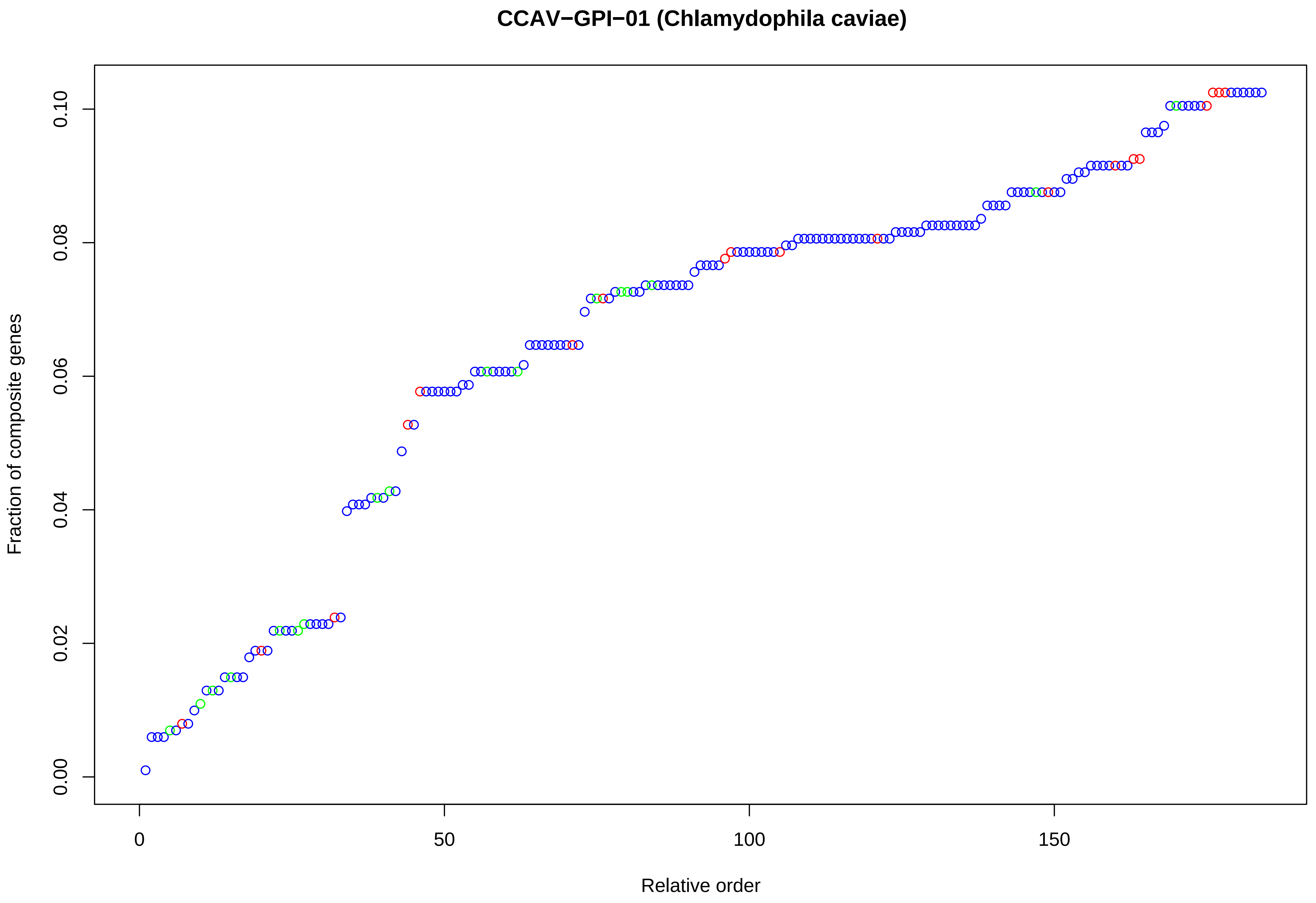

CCAV−GPI−01 (Chlamydophila caviae)
Fraction of composite genes
0.00
0.02
0.04
0.06
0.08
0.10
Relative order
0
50
100
150

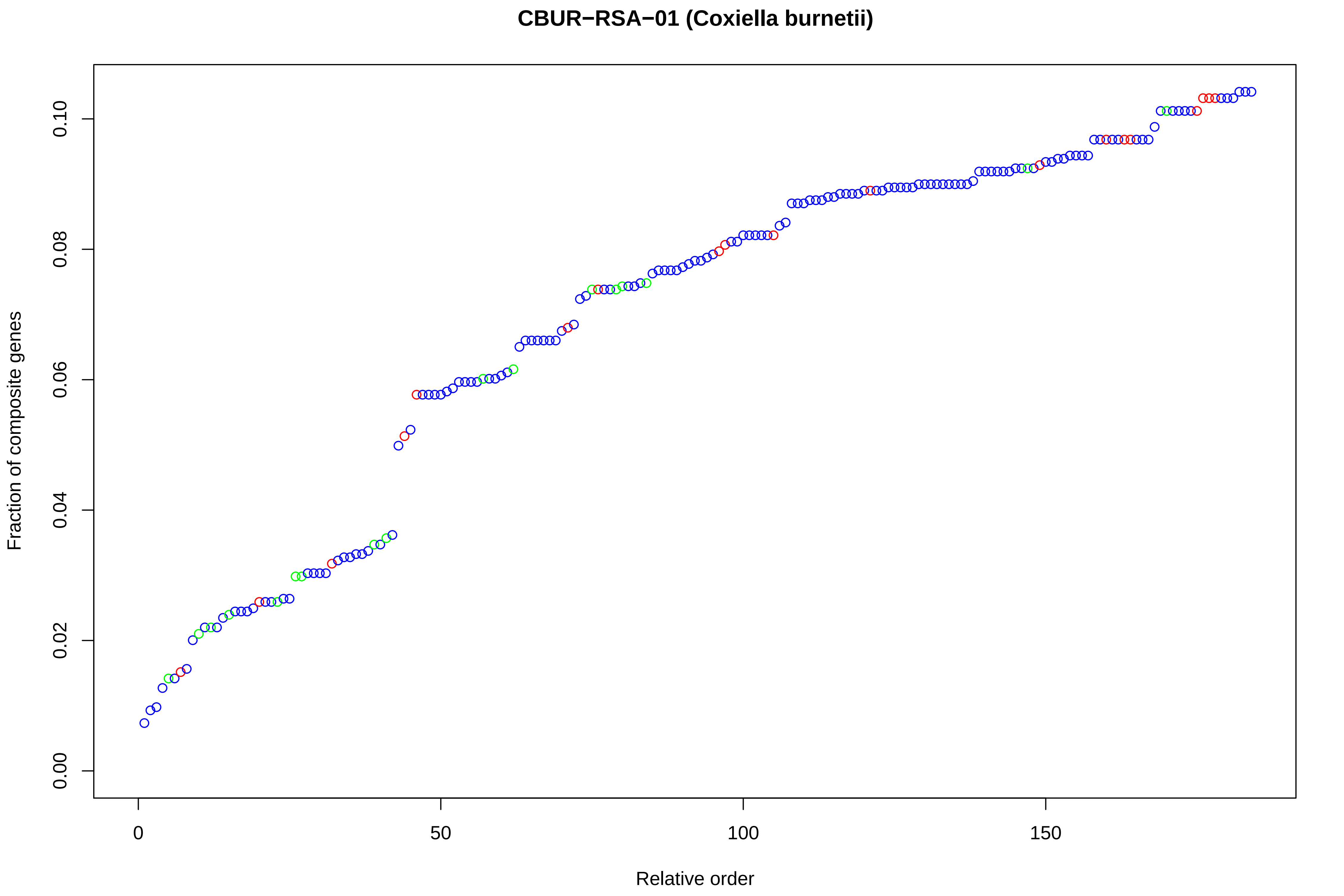

CBUR−RSA−01 (Coxiella burnetii)
Fraction of composite genes
0.00
0.02
0.04
0.06
0.08
0.10
0
50
100
150
Relative order

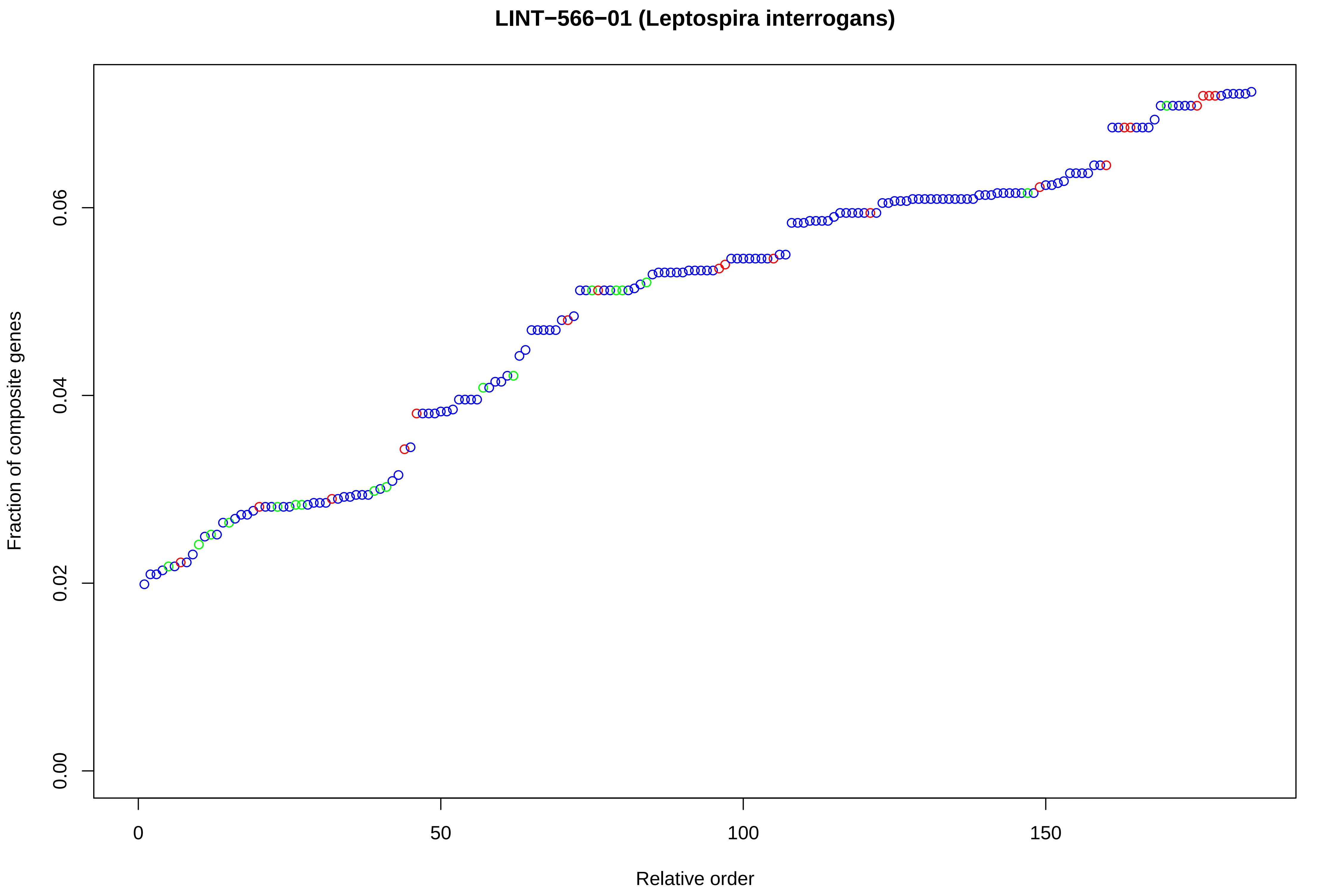

LINT−566−01 (Leptospira interrogans)
Fraction of composite genes
Relative order
0.00
0.02
0.04
0.06
0
50
100
150

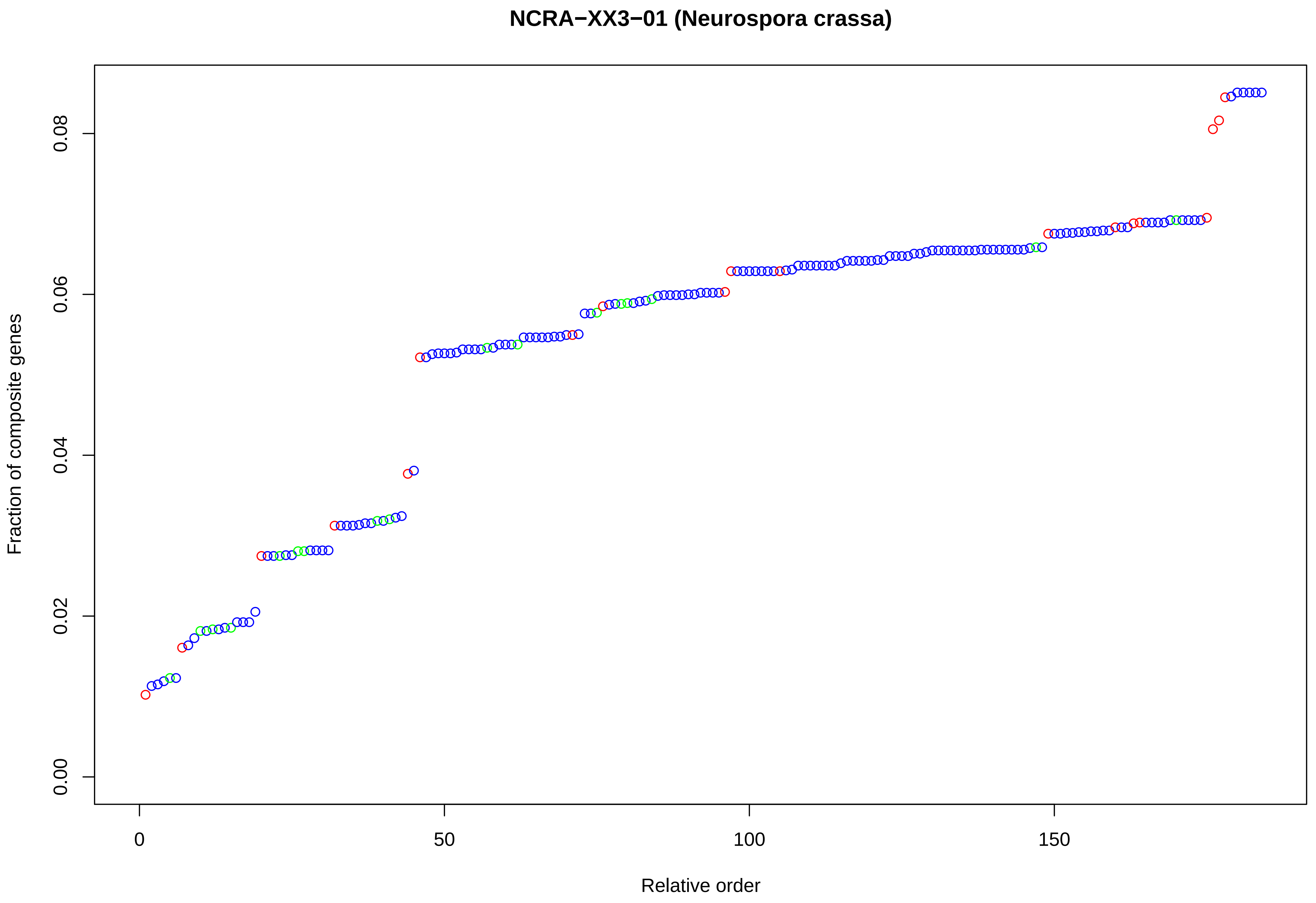
NCRA−XX3−01 (Neurospora crassa)
Fraction of composite genes
0.00
0.02
0.04
0.06
0.08
Relative order
0
50
100
150

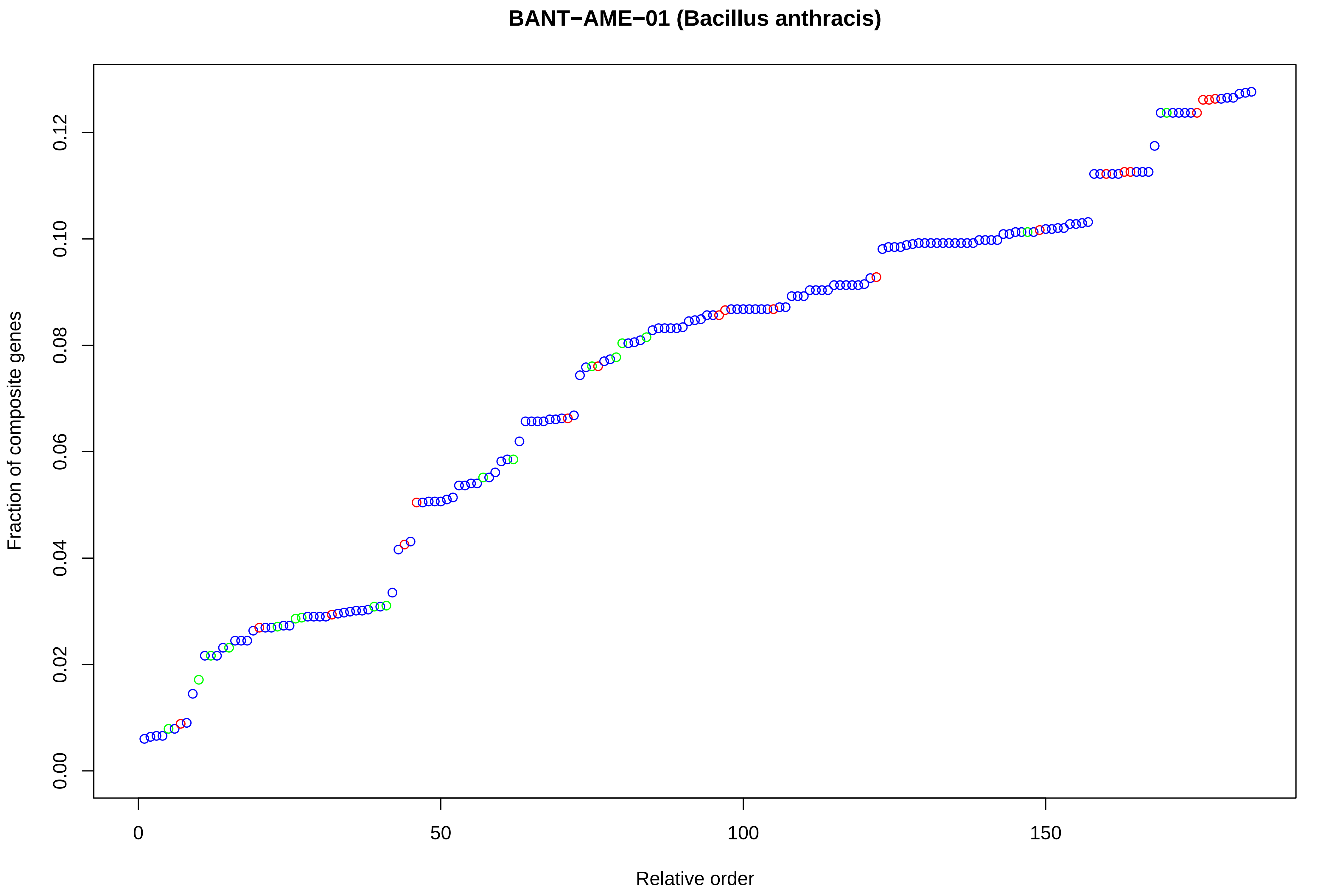

BANT−AME−01 (Bacillus anthracis)
Fraction of composite genes
0.00
0.02
0.04
0.06
0.08
0.10
0.12
Relative order
0
50
100
150

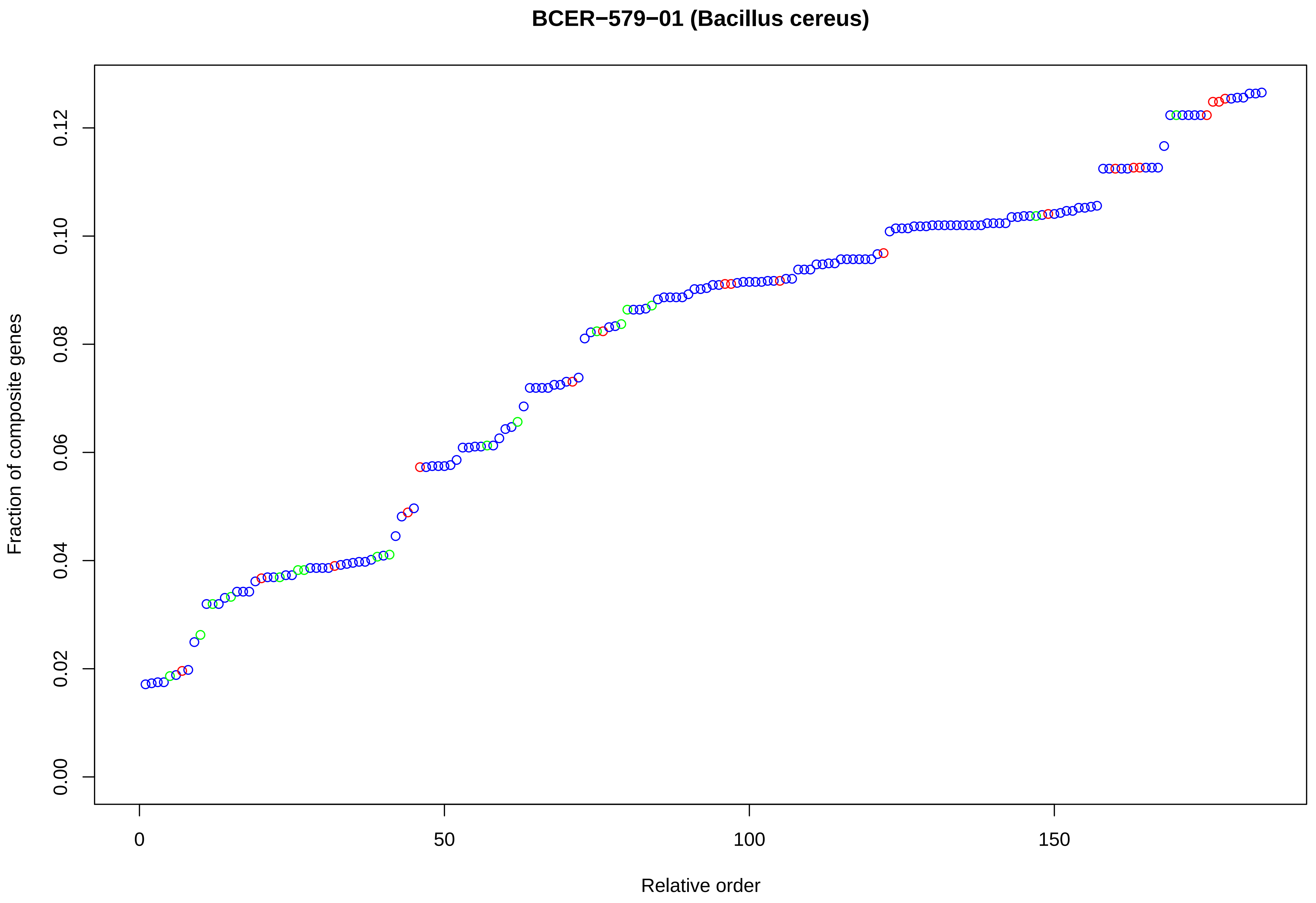
BCER−579−01 (Bacillus cereus)
Fraction of composite genes
0.00
0.02
0.04
0.06
0.08
0.10
0.12
0
50
100
150
Relative order

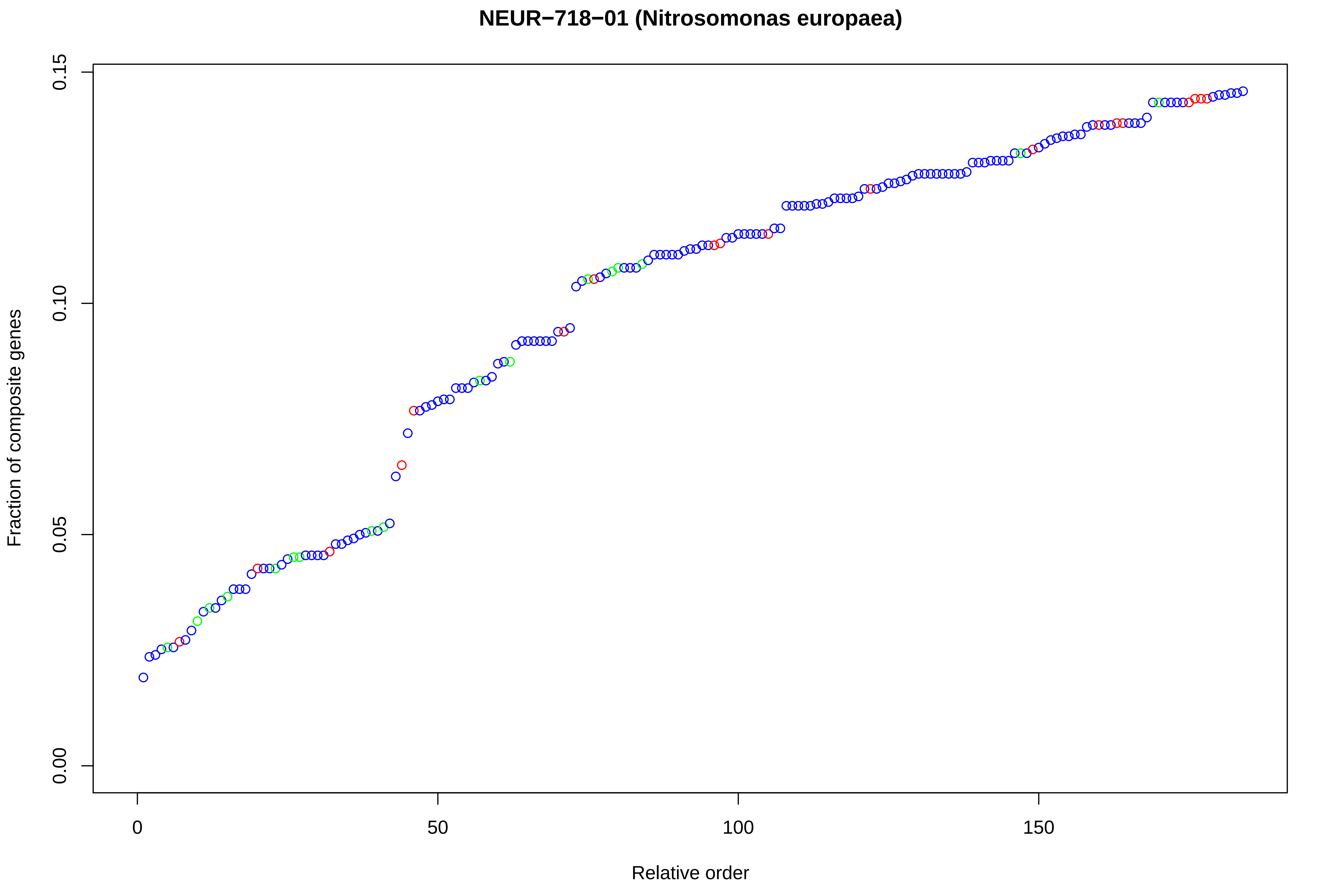

NEUR−718−01 (Nitrosomonas europaea)
Fraction of composite genes
0.00
0.05
0.10
0.15
Relative order
0
50
100
150

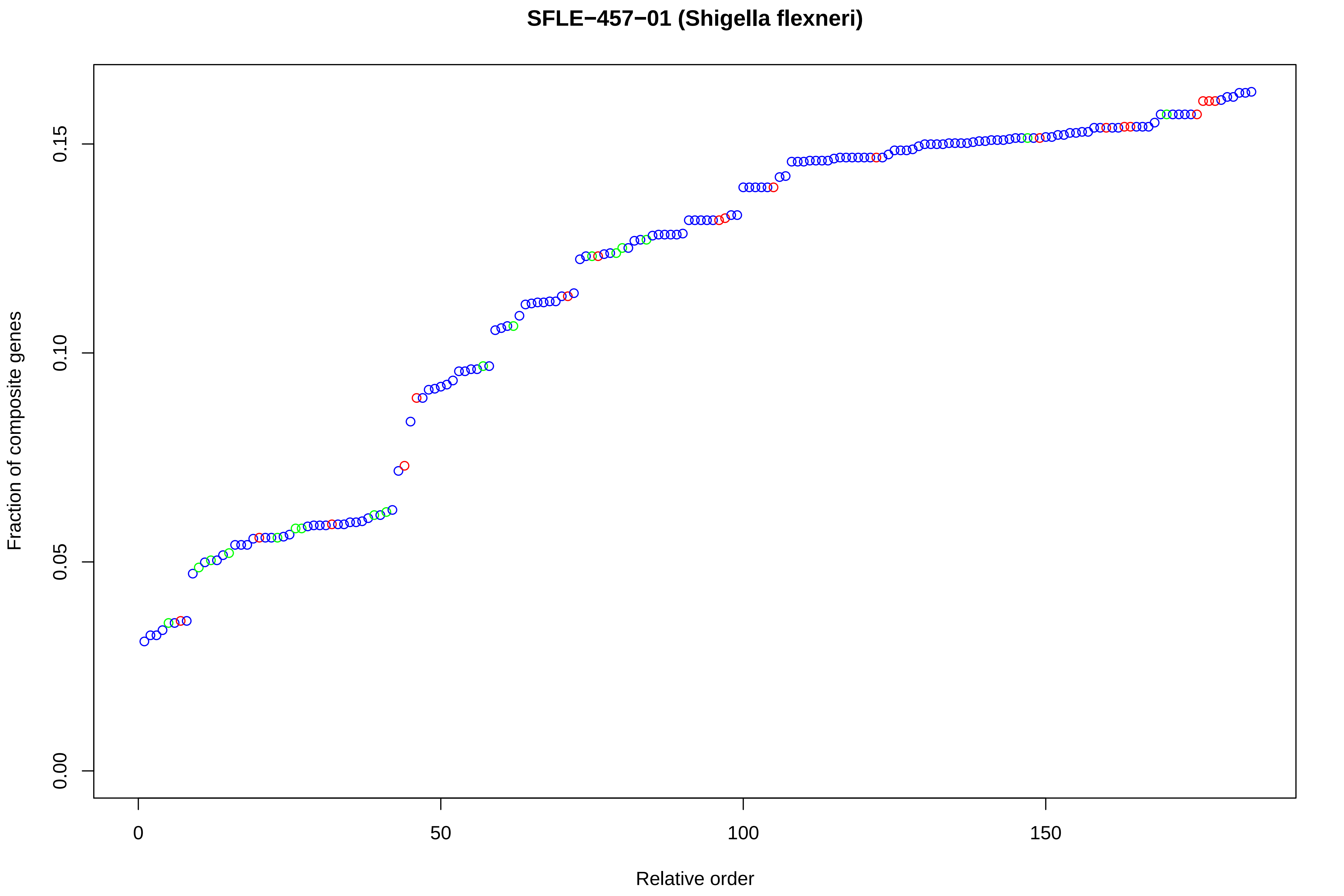

SFLE−457−01 (Shigella flexneri)
Fraction of composite genes
0.00
0.05
0.10
0.15
Relative order
0
50
100
150

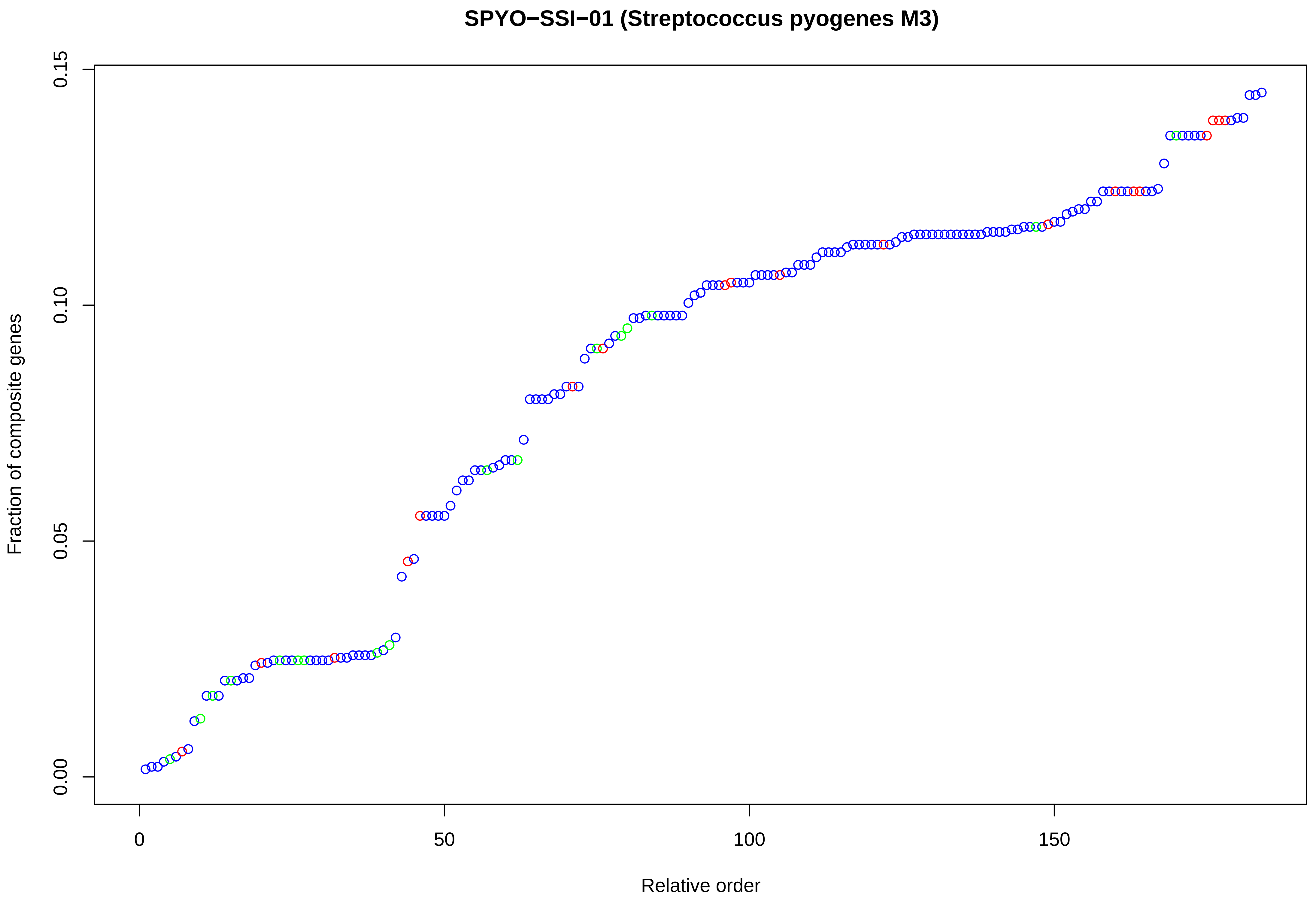
SPYO−SSI−01 (Streptococcus pyogenes M3)
Fraction of composite genes
0.00
0.05
0.10
0.15
0
50
100
150
Relative order

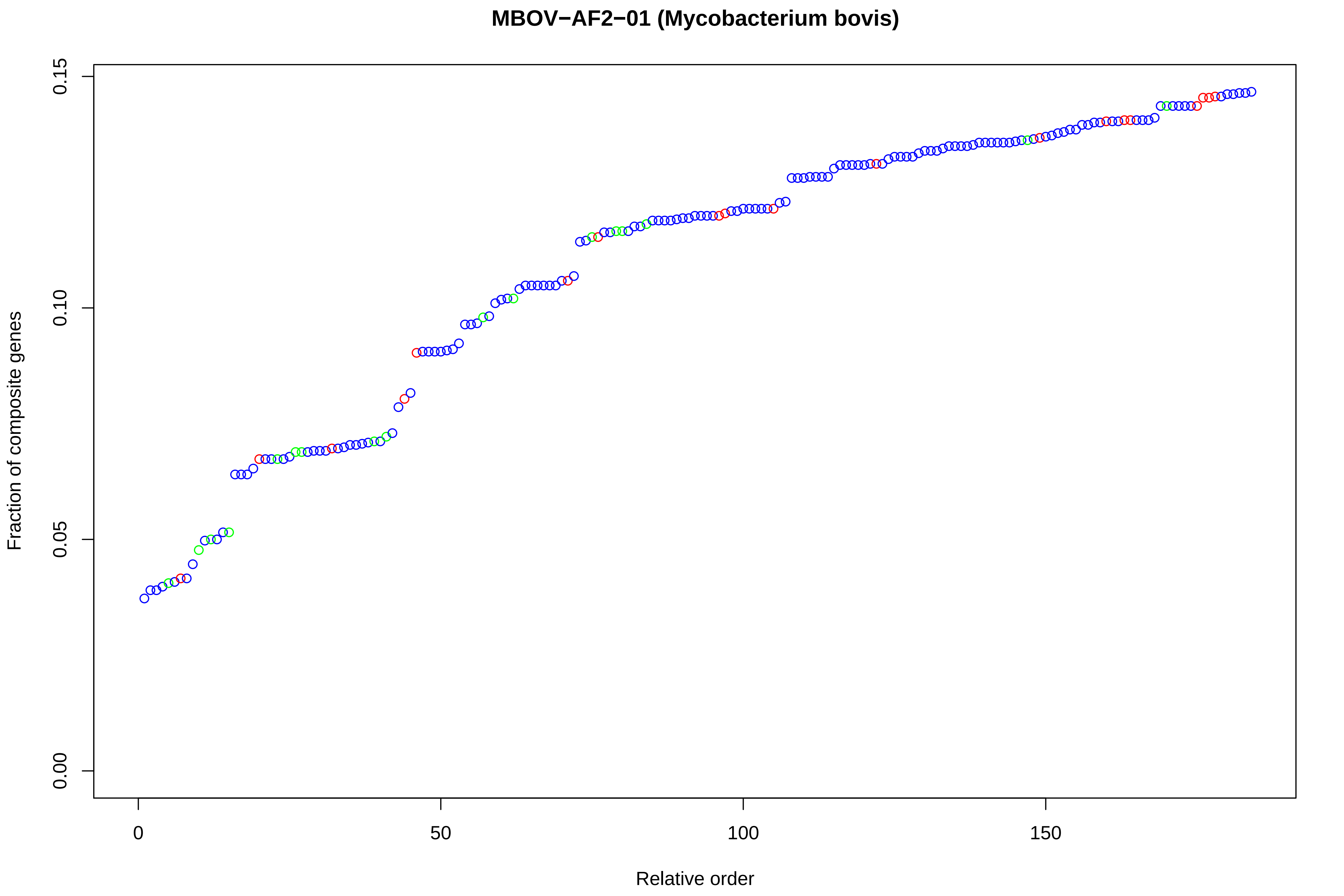
MBOV−AF2−01 (Mycobacterium bovis)
Fraction of composite genes
Relative order
0.00
0.05
0.10
0.15
0
50
100
150

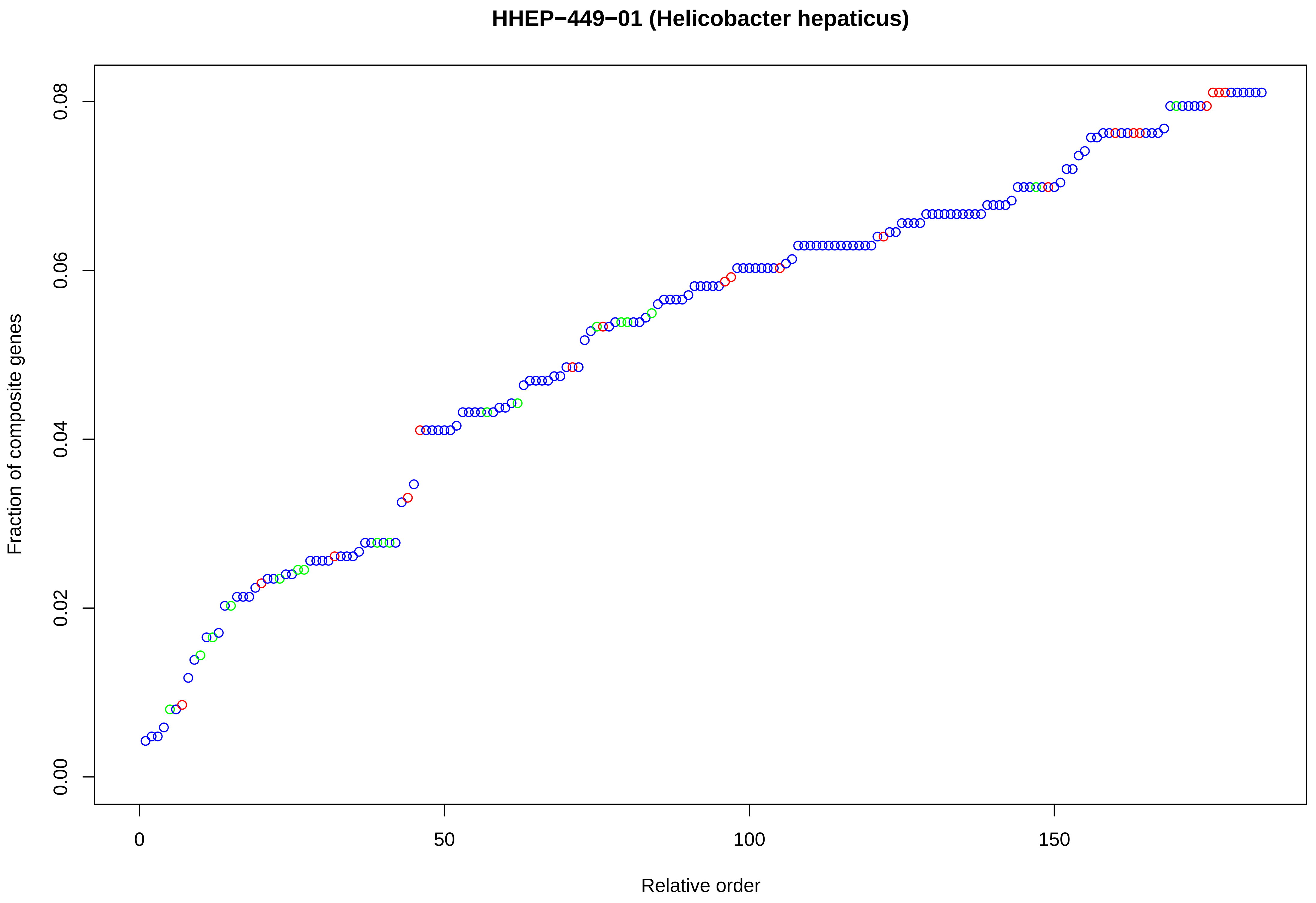

HHEP−449−01 (Helicobacter hepaticus)
Fraction of composite genes
0.00
0.02
0.04
0.06
0.08
0
50
100
150
Relative order

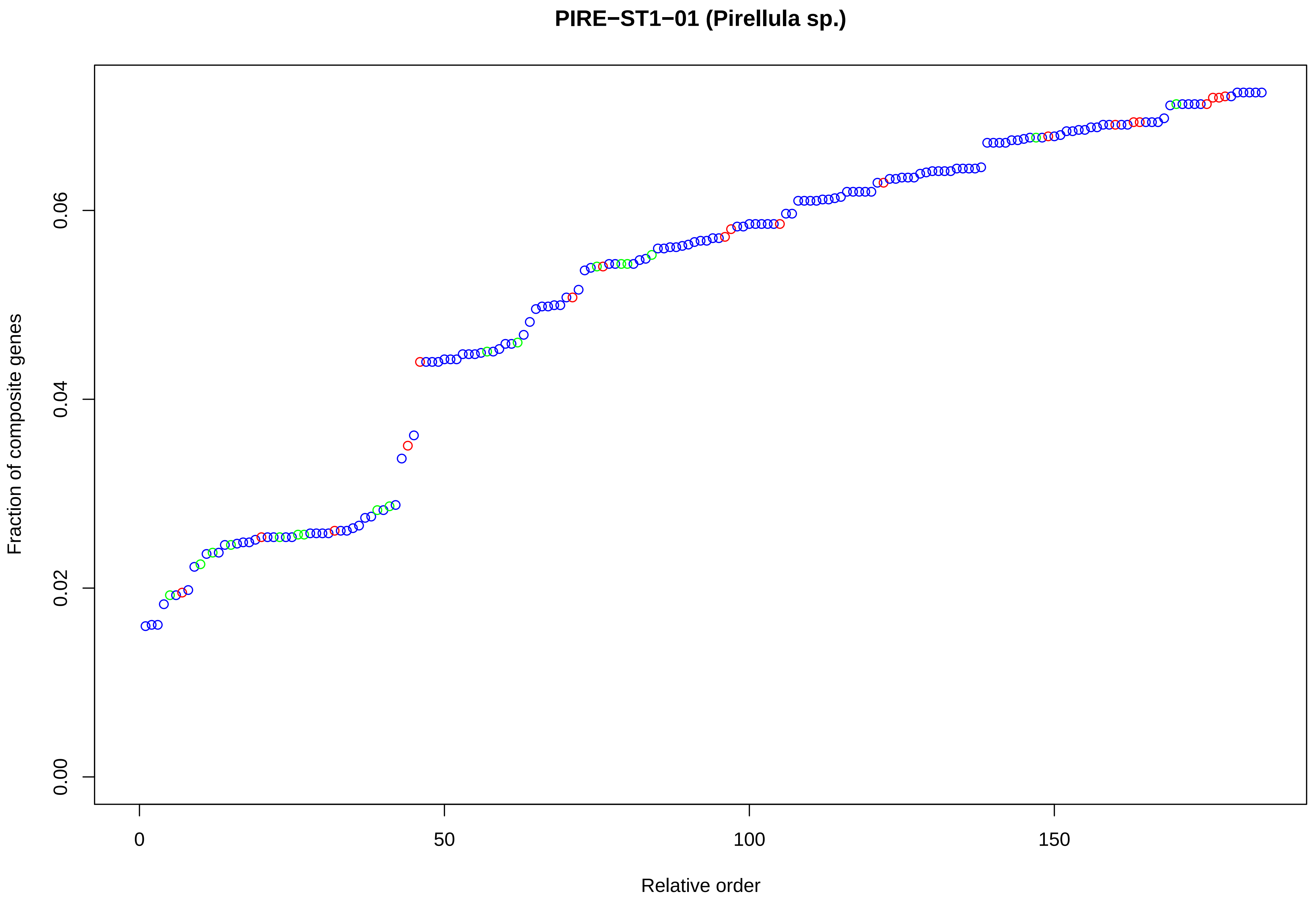

PIRE−ST1−01 (Pirellula sp.)
Fraction of composite genes
0.00
0.02
0.04
0.06
0
50
100
150
Relative order

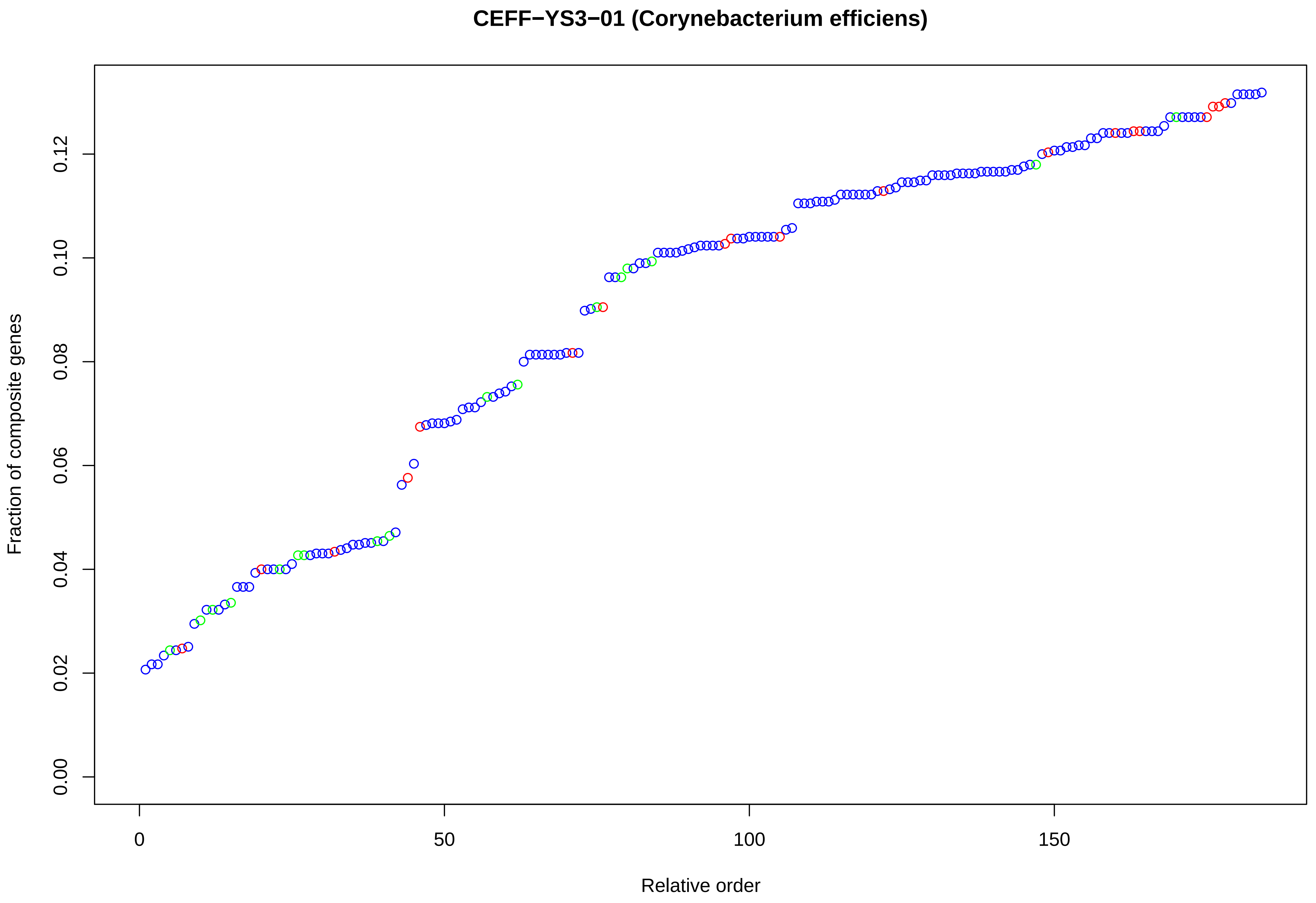

CEFF−YS3−01 (Corynebacterium efficiens)
Fraction of composite genes
Relative order
0.00
0.02
0.04
0.06
0.08
0.10
0.12
0
50
100
150

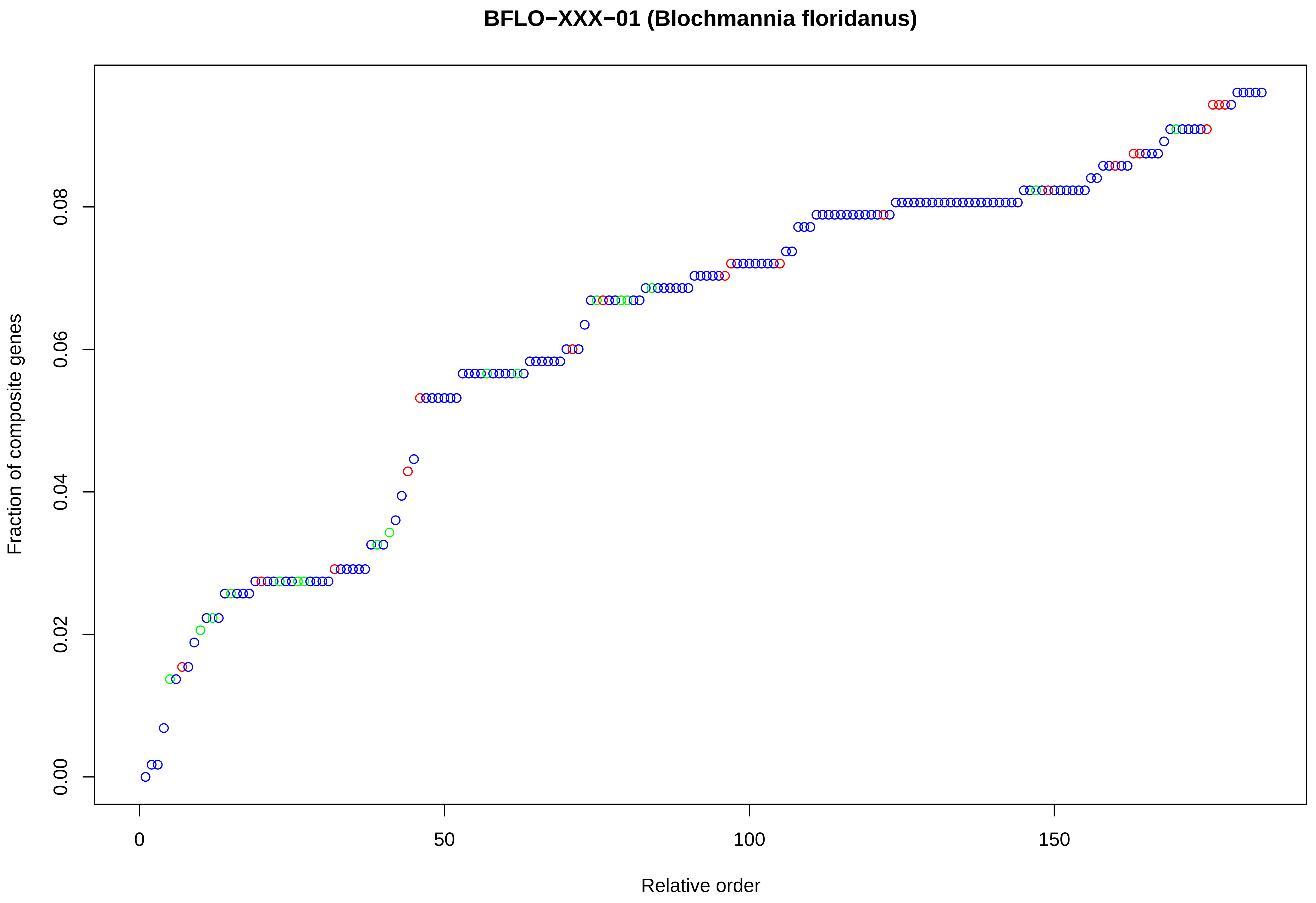

BFLO−XXX−01 (Blochmannia floridanus)
Fraction of composite genes
0.00
0.02
0.04
0.06
0.08
0
50
100
150
Relative order

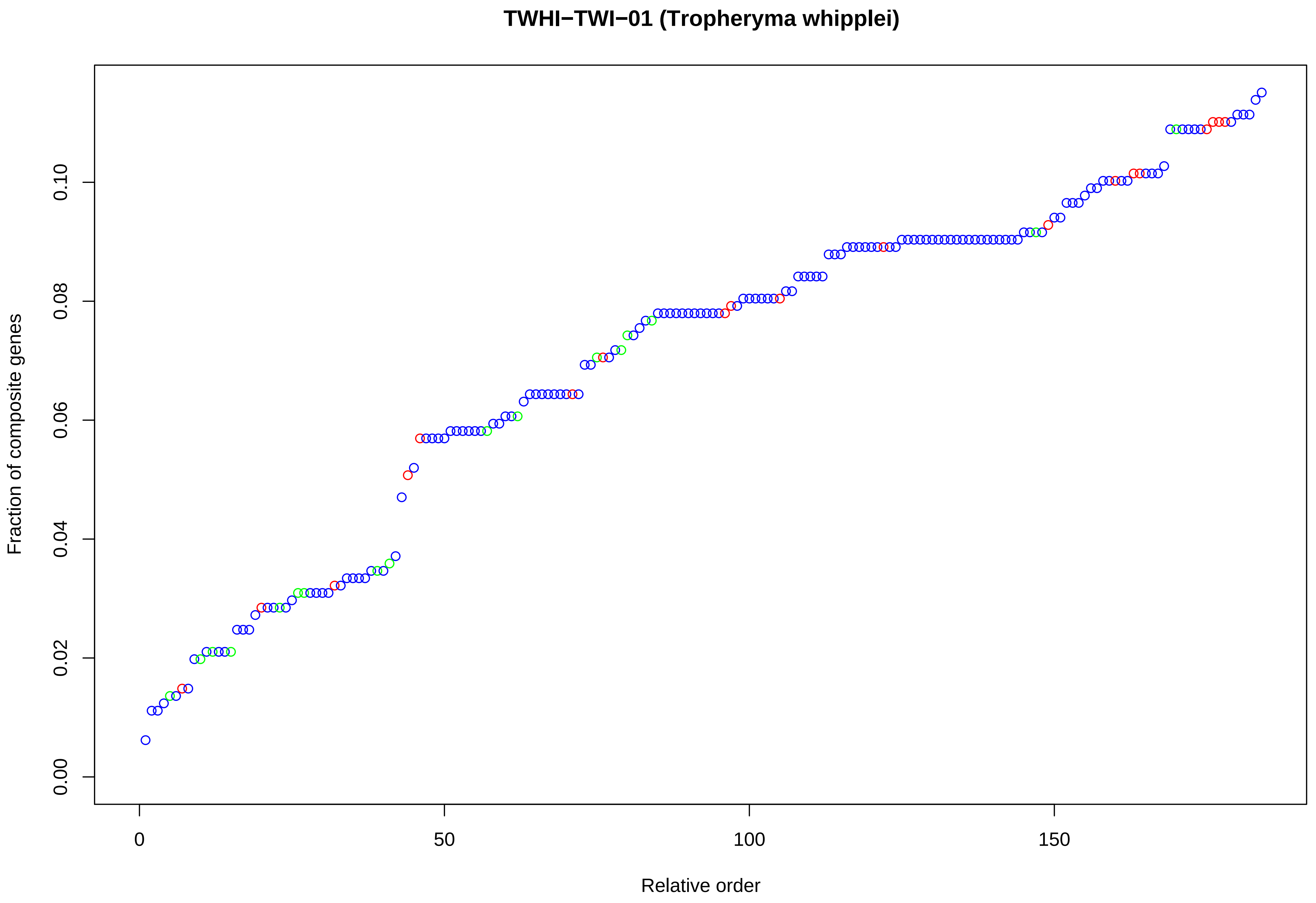

TWHI−TWI−01 (Tropheryma whipplei)
Fraction of composite genes
0.00
0.02
0.04
0.06
0.08
0.10
0
50
100
150
Relative order

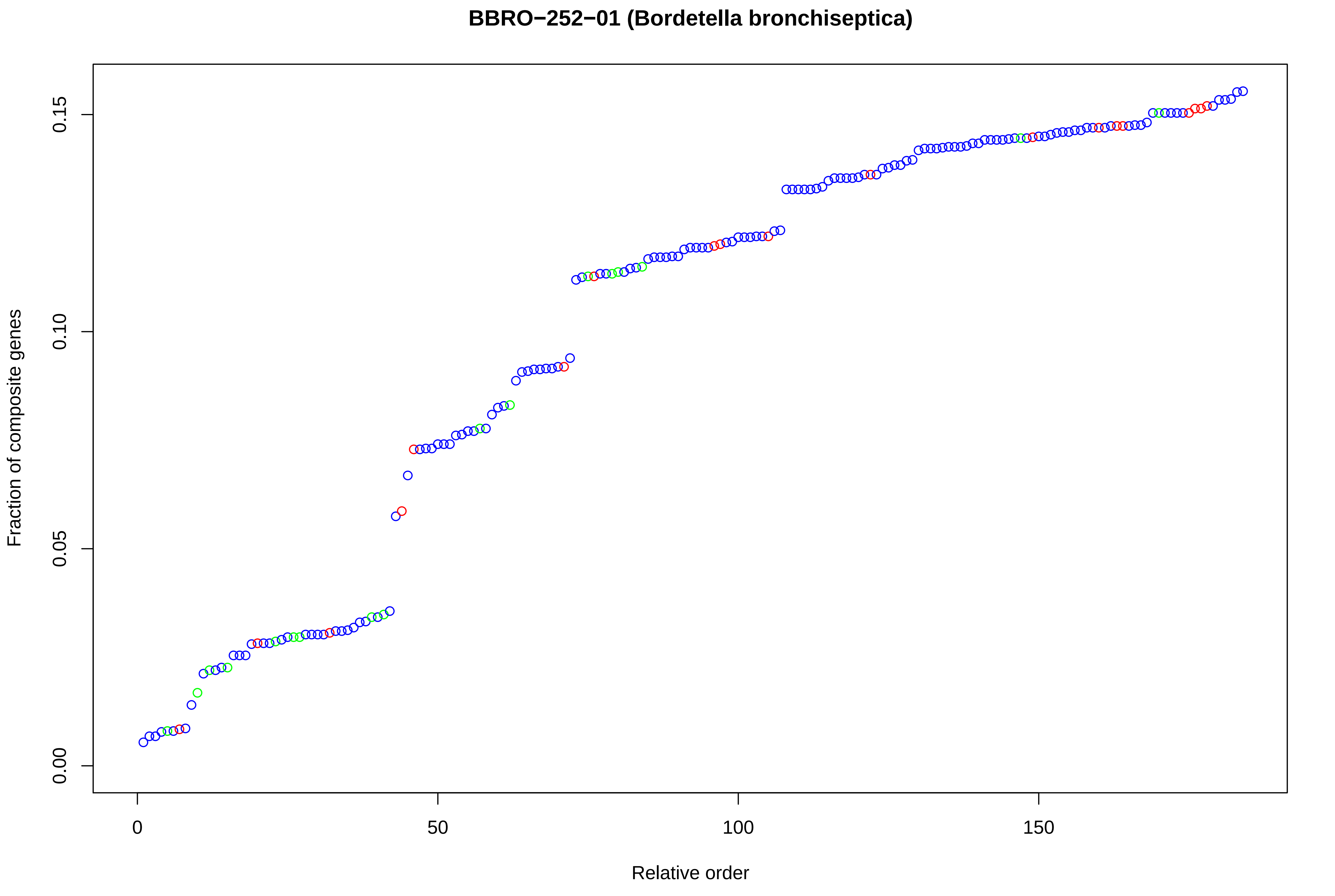
BBRO−252−01 (Bordetella bronchiseptica)
Fraction of composite genes
0.00
0.05
0.10
0.15
Relative order
0
50
100
150

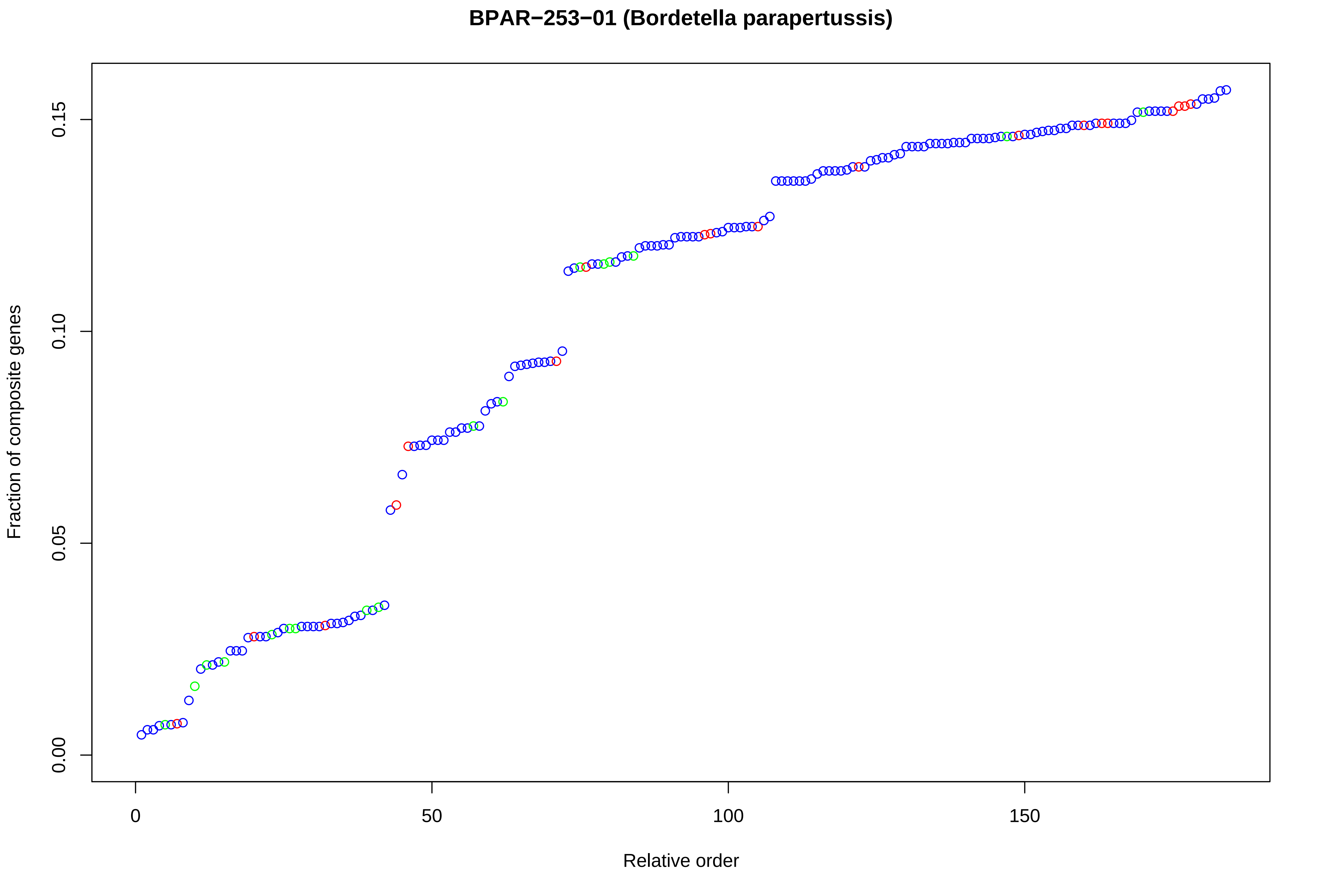
BPAR−253−01 (Bordetella parapertussis)
Fraction of composite genes
0.00
0.05
0.10
0.15
0
50
100
150
Relative order

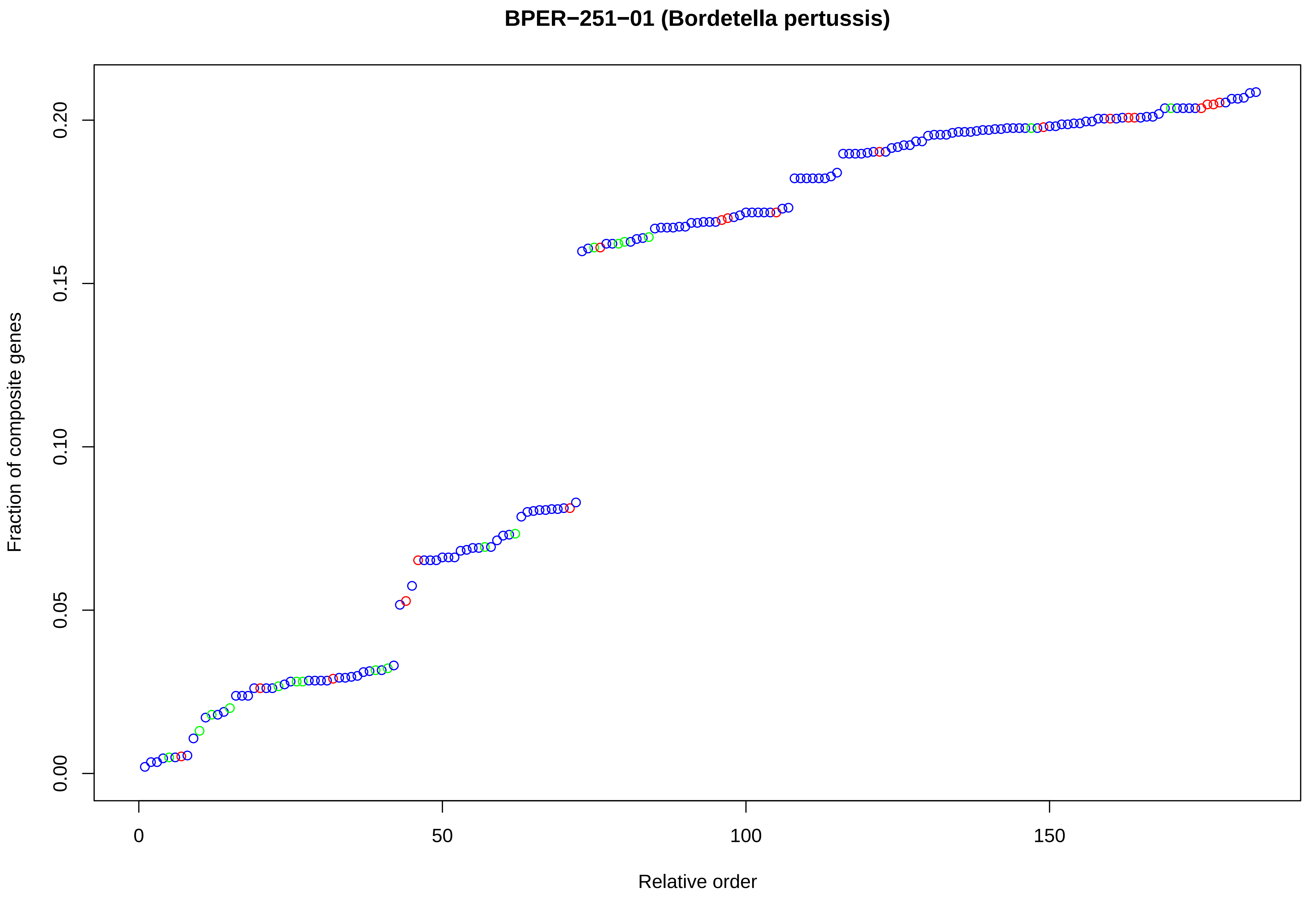
BPER−251−01 (Bordetella pertussis)
Fraction of composite genes
0.00
0.05
0.10
0.15
0.20
Relative order
0
50
100
150

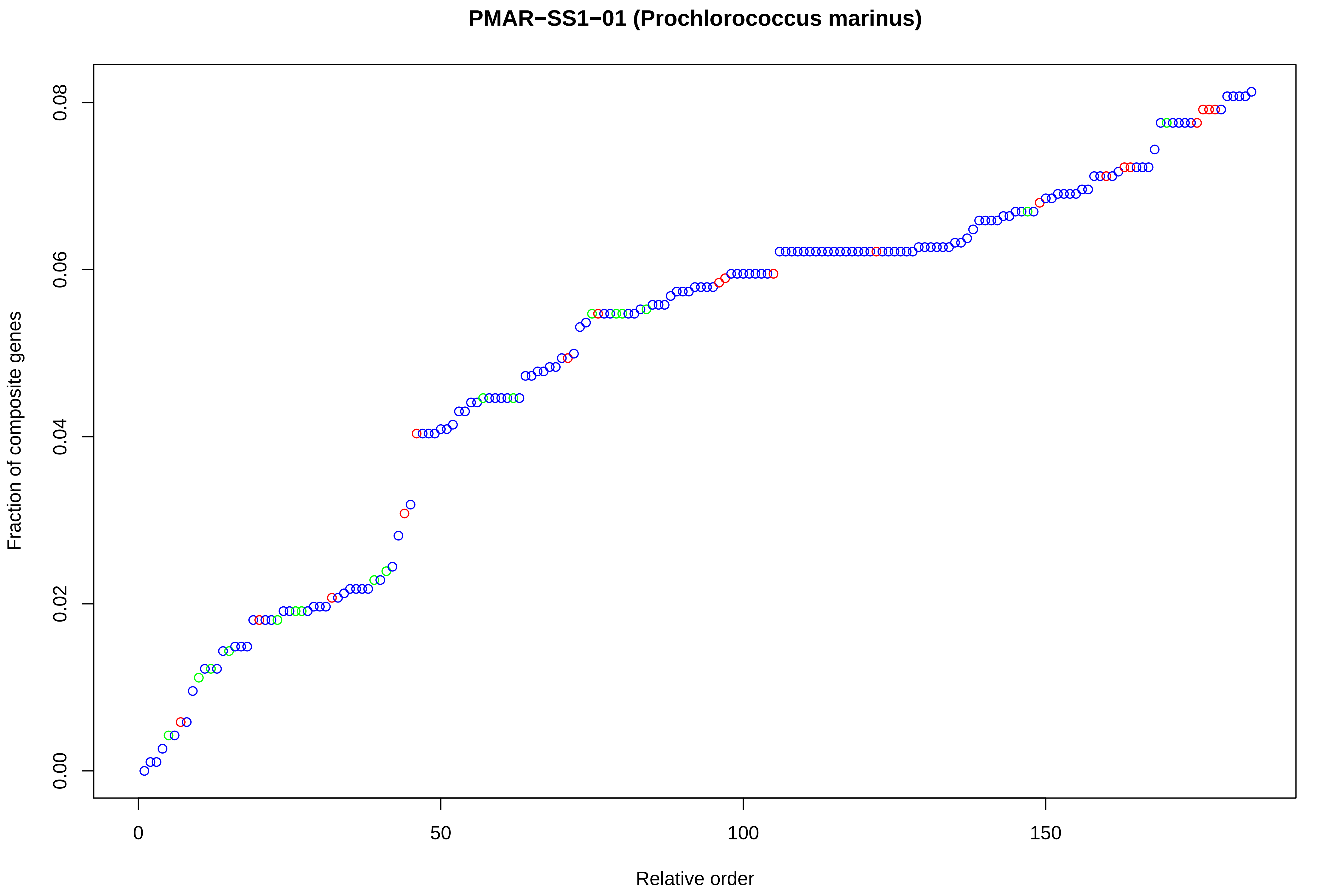
PMAR−SS1−01 (Prochlorococcus marinus)
Fraction of composite genes
Relative order
0.00
0.02
0.04
0.06
0.08
0
50
100
150

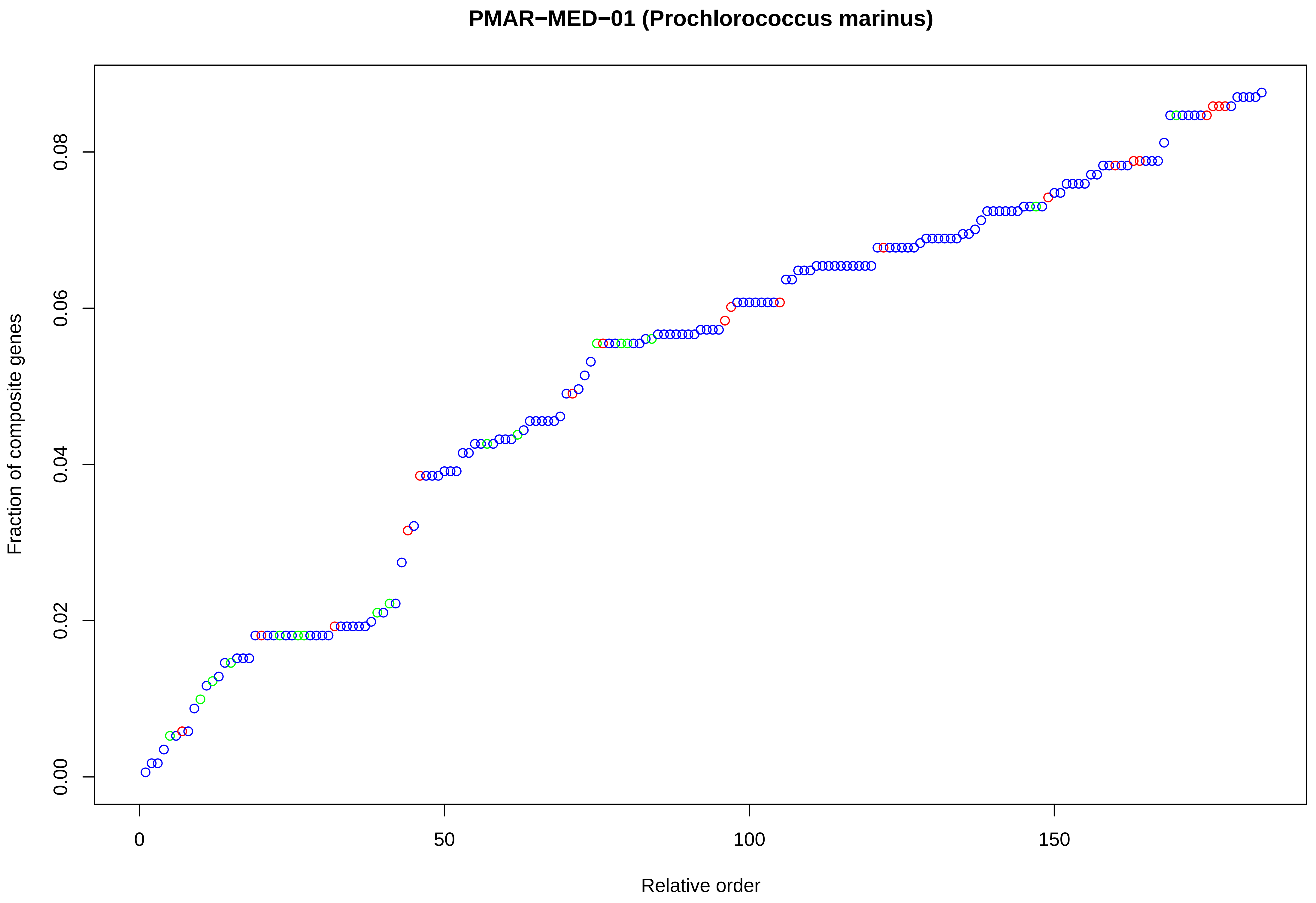

PMAR−MED−01 (Prochlorococcus marinus)
Fraction of composite genes
0.00
0.02
0.04
0.06
0.08
Relative order
0
50
100
150

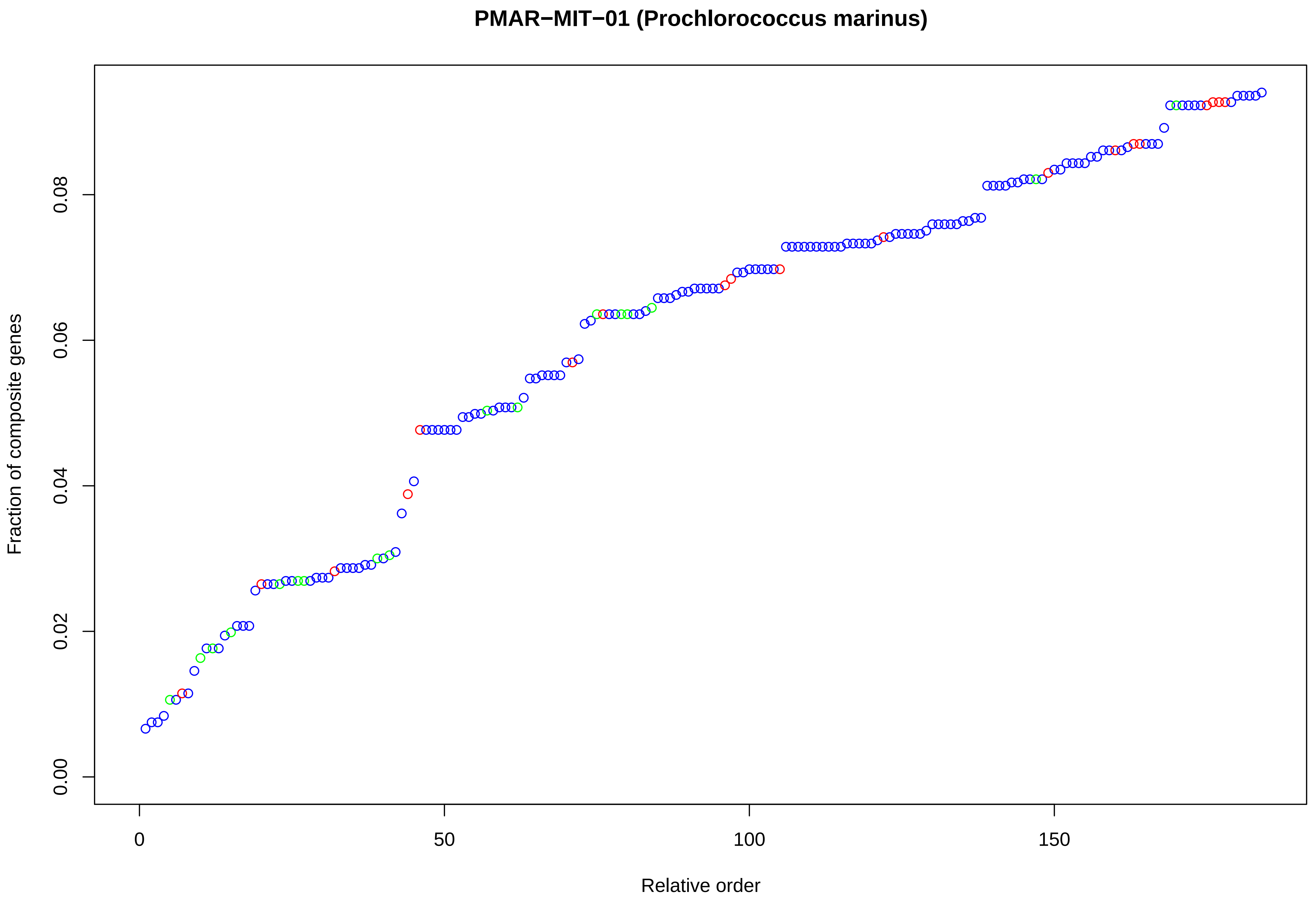
PMAR−MIT−01 (Prochlorococcus marinus)
Fraction of composite genes
0.00
0.02
0.04
0.06
0.08
0
50
100
150
Relative order

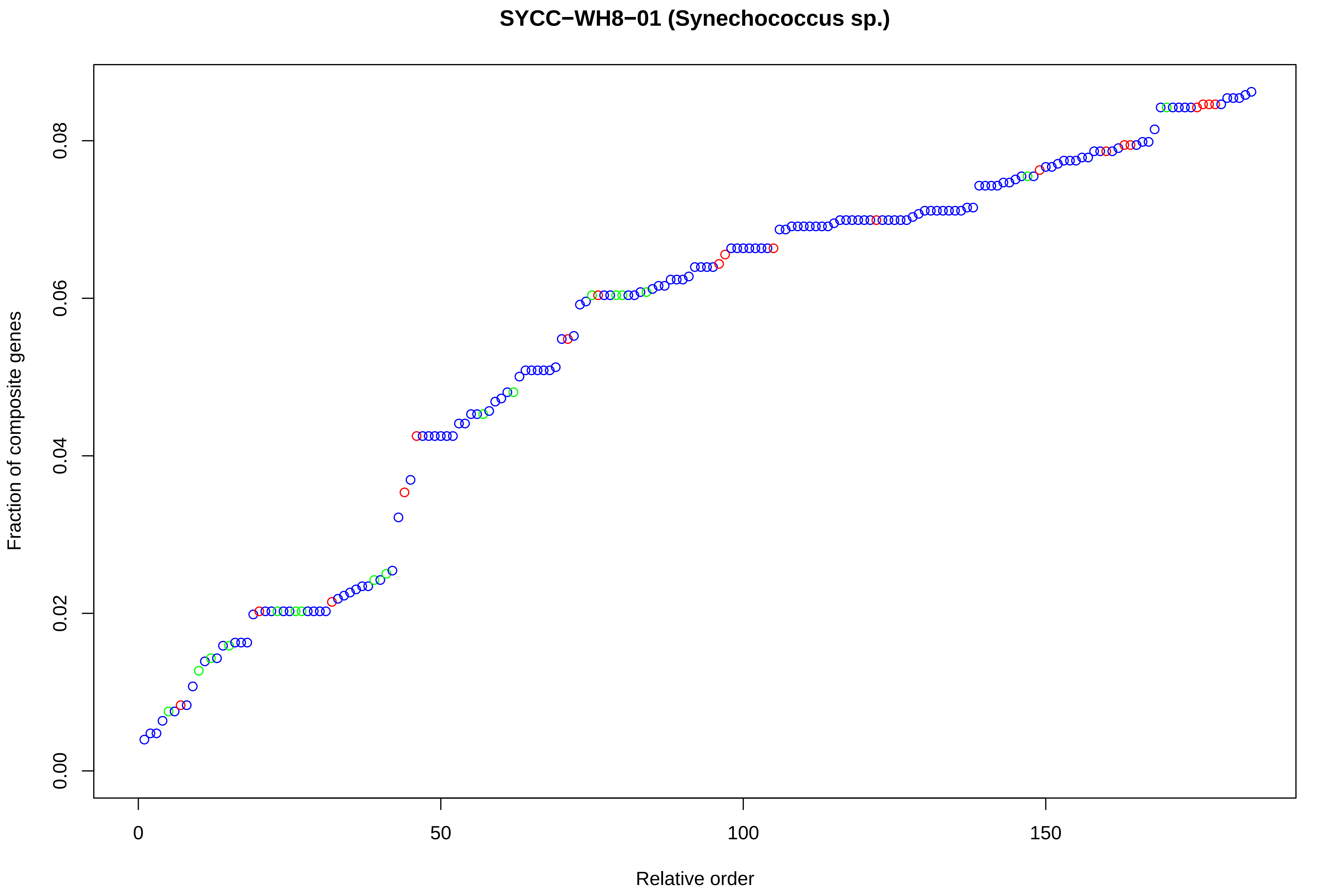

SYCC−WH8−01 (Synechococcus sp.)
Fraction of composite genes
0.00
0.02
0.04
0.06
0.08
Relative order
0
50
100
150

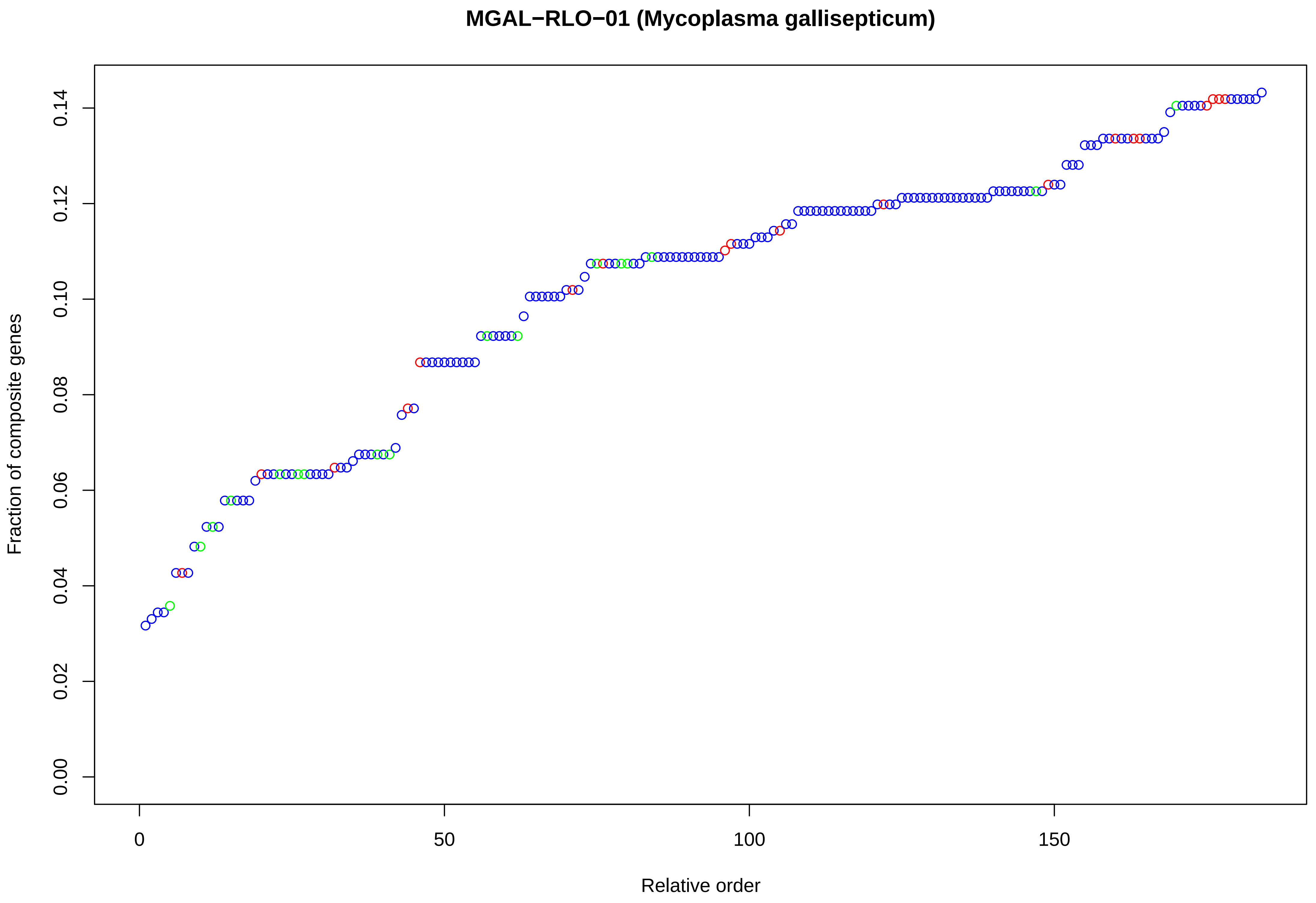
MGAL−RLO−01 (Mycoplasma gallisepticum)
Fraction of composite genes
0.00
0.02
0.04
0.06
0.08
0.10
0.12
0.14
0
50
100
150
Relative order

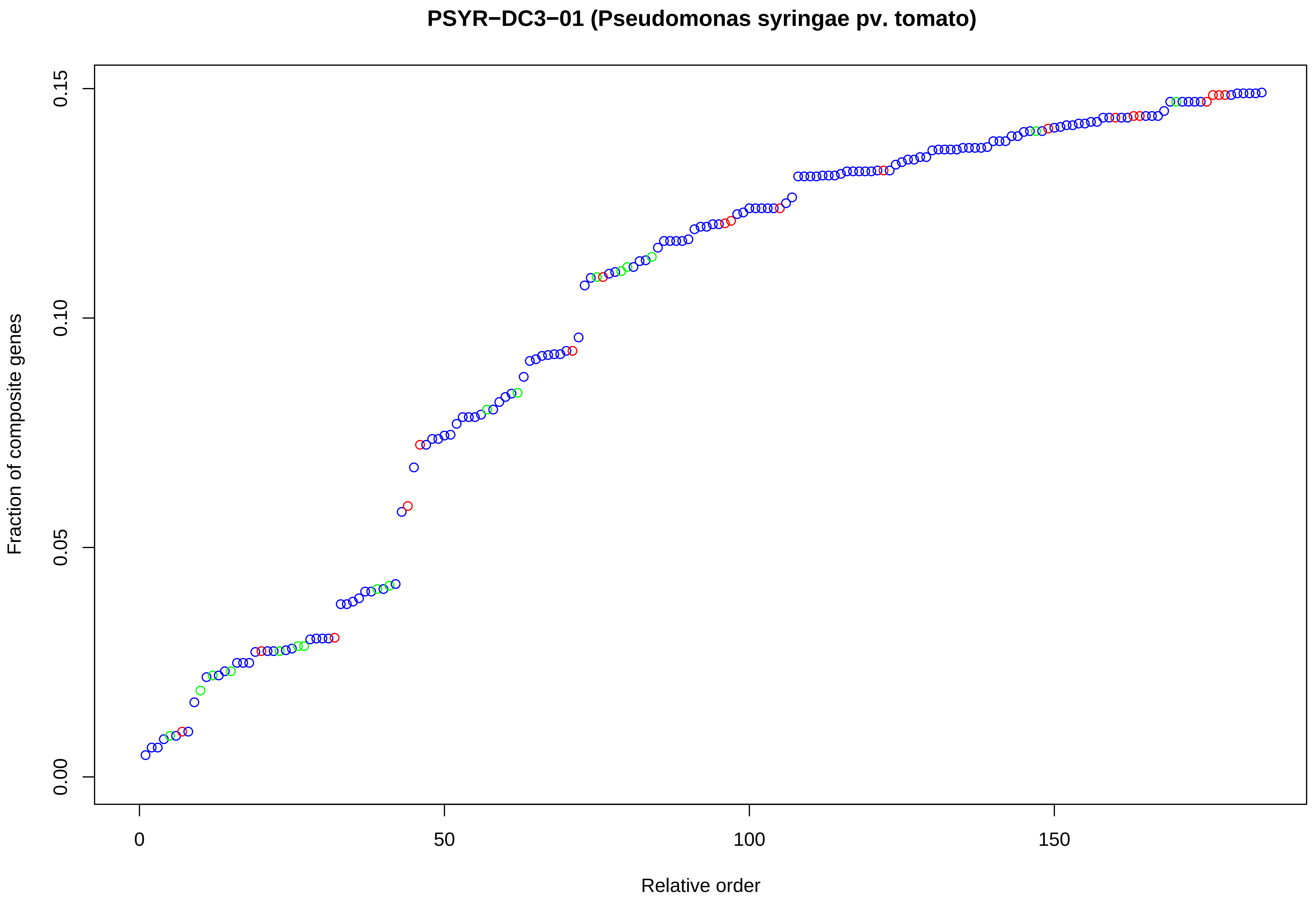

PSYR−DC3−01 (Pseudomonas syringae pv. tomato)
Fraction of composite genes
0.00
0.05
0.10
0.15
0
50
100
150
Relative order

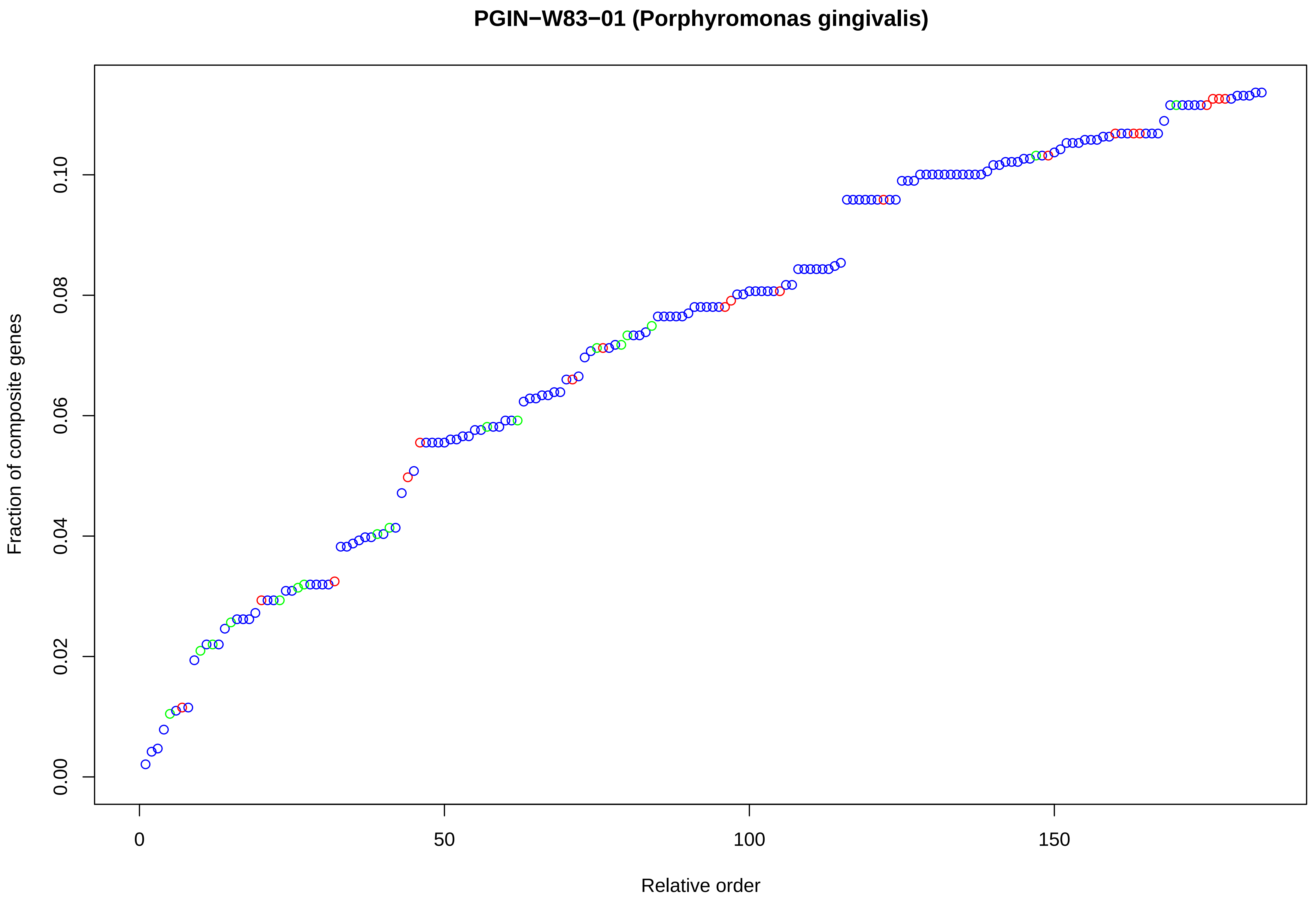

PGIN−W83−01 (Porphyromonas gingivalis)
Fraction of composite genes
0.00
0.02
0.04
0.06
0.08
0.10
Relative order
0
50
100
150

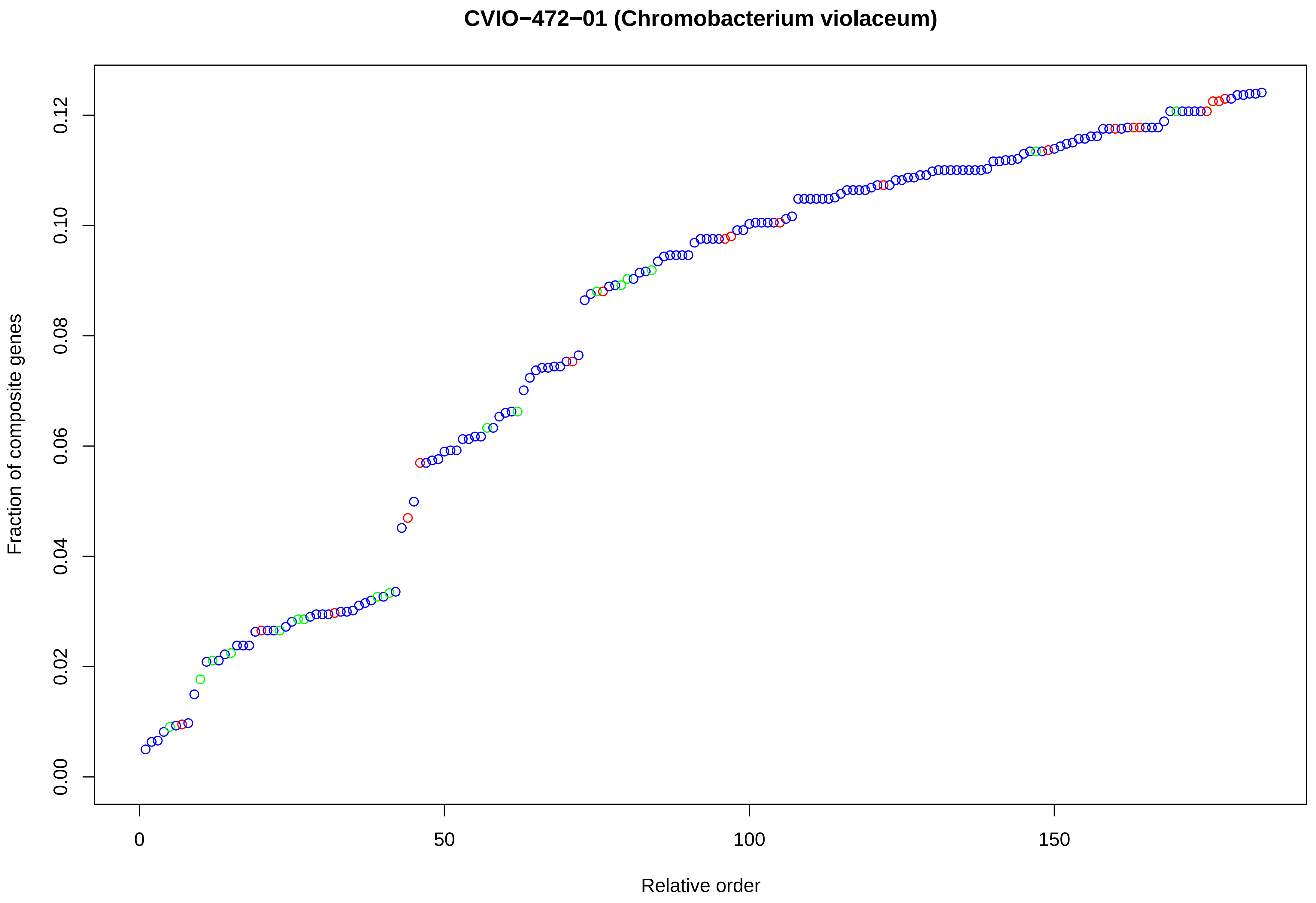

CVIO−472−01 (Chromobacterium violaceum)
Fraction of composite genes
0.00
0.02
0.04
0.06
0.08
0.10
0.12
Relative order
0
50
100
150

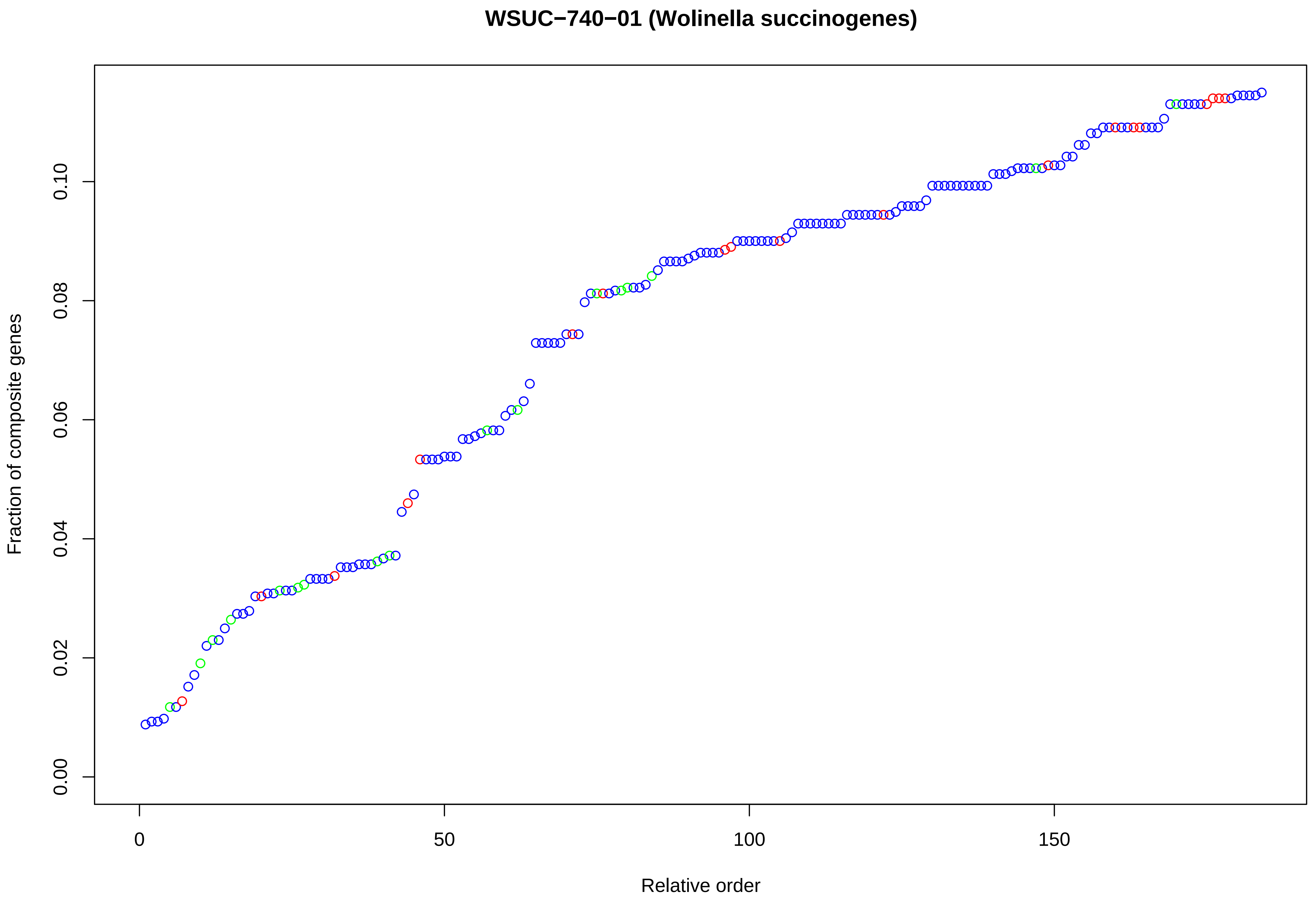
WSUC−740−01 (Wolinella succinogenes)
Fraction of composite genes
Relative order
0.00
0.02
0.04
0.06
0.08
0.10
0
50
100
150

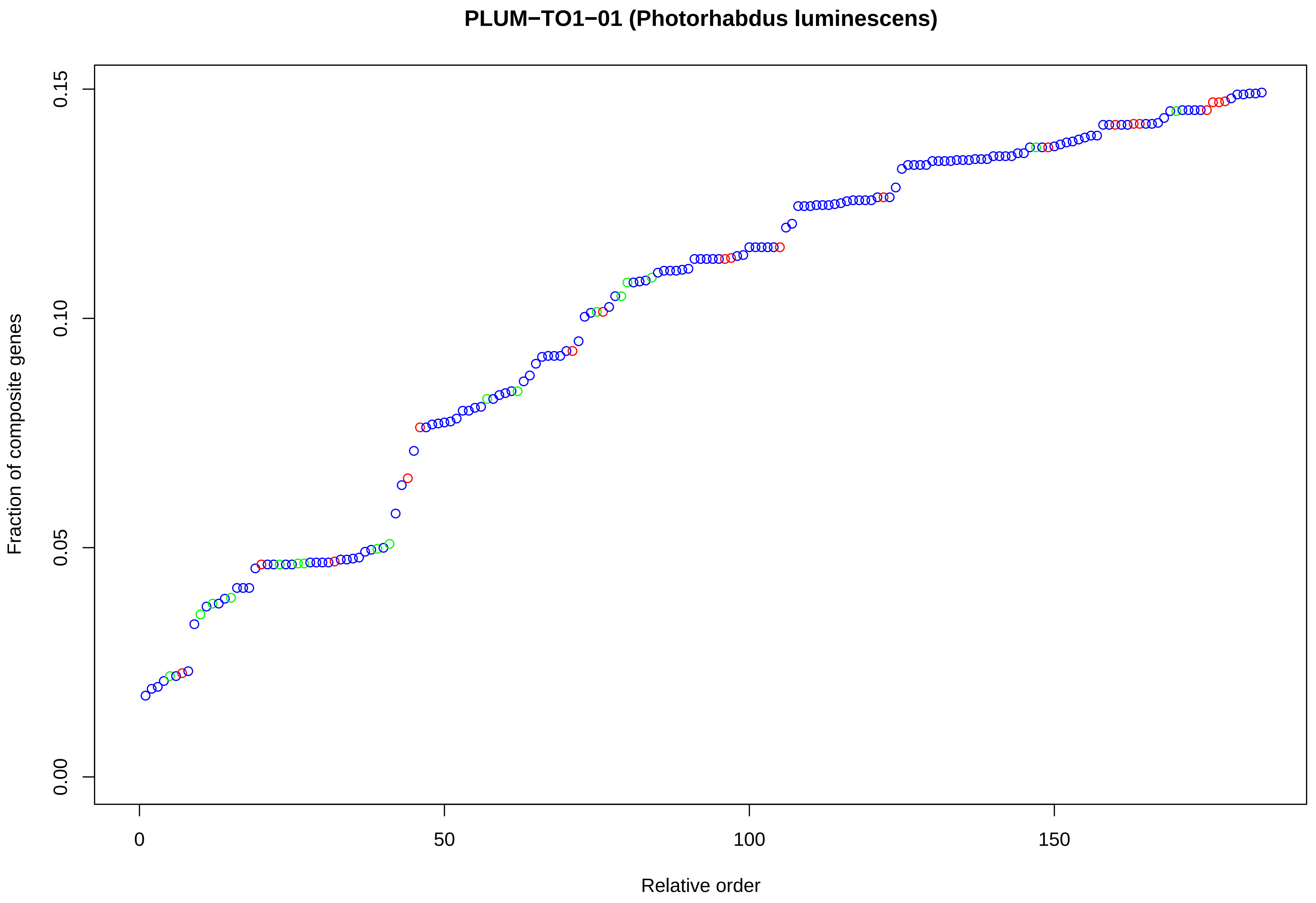

PLUM−TO1−01 (Photorhabdus luminescens)
Fraction of composite genes
Relative order
0.00
0.05
0.10
0.15
0
50
100
150

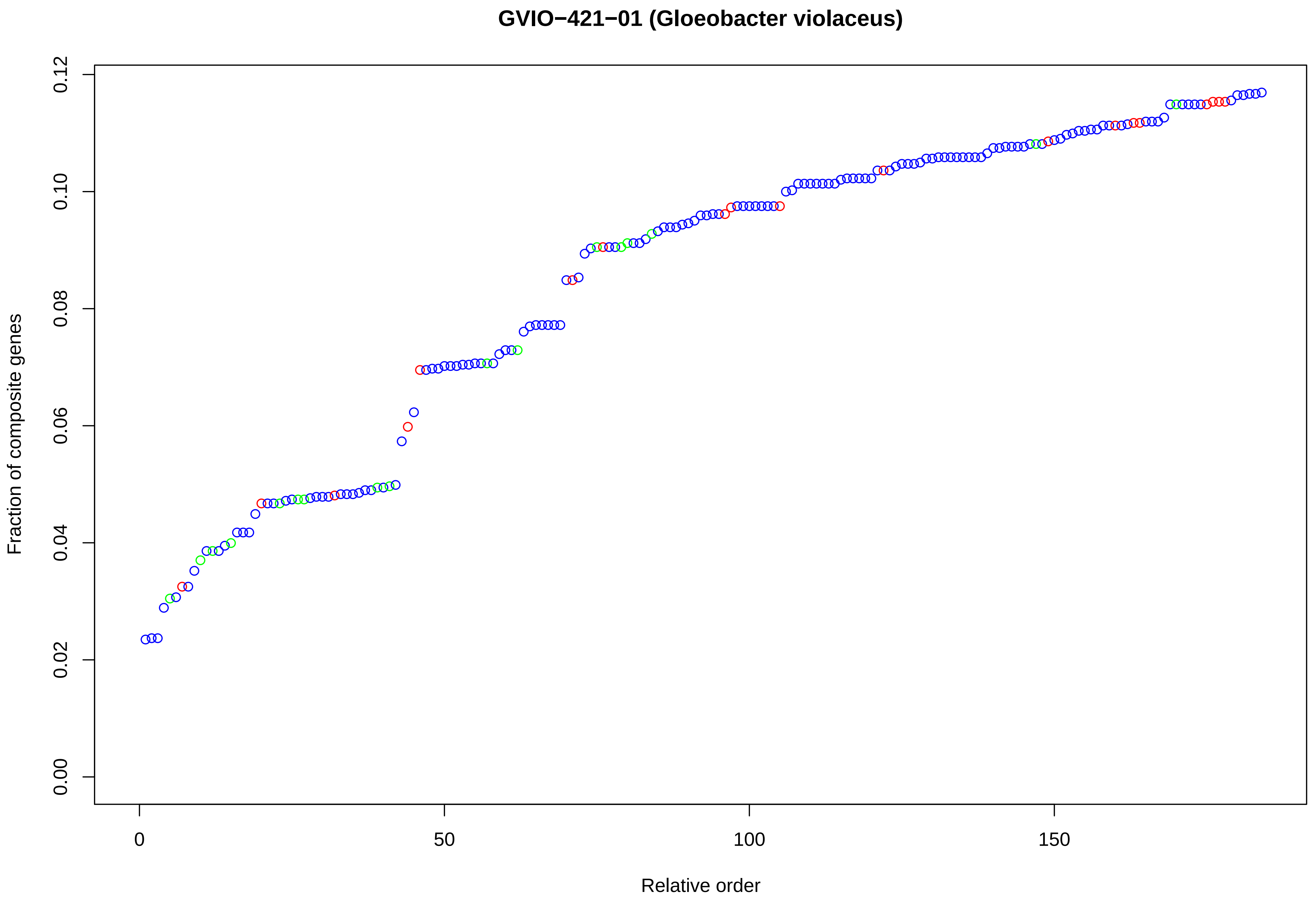

GVIO−421−01 (Gloeobacter violaceus)
Fraction of composite genes
0.00
0.02
0.04
0.06
0.08
0.10
0.12
Relative order
0
50
100
150

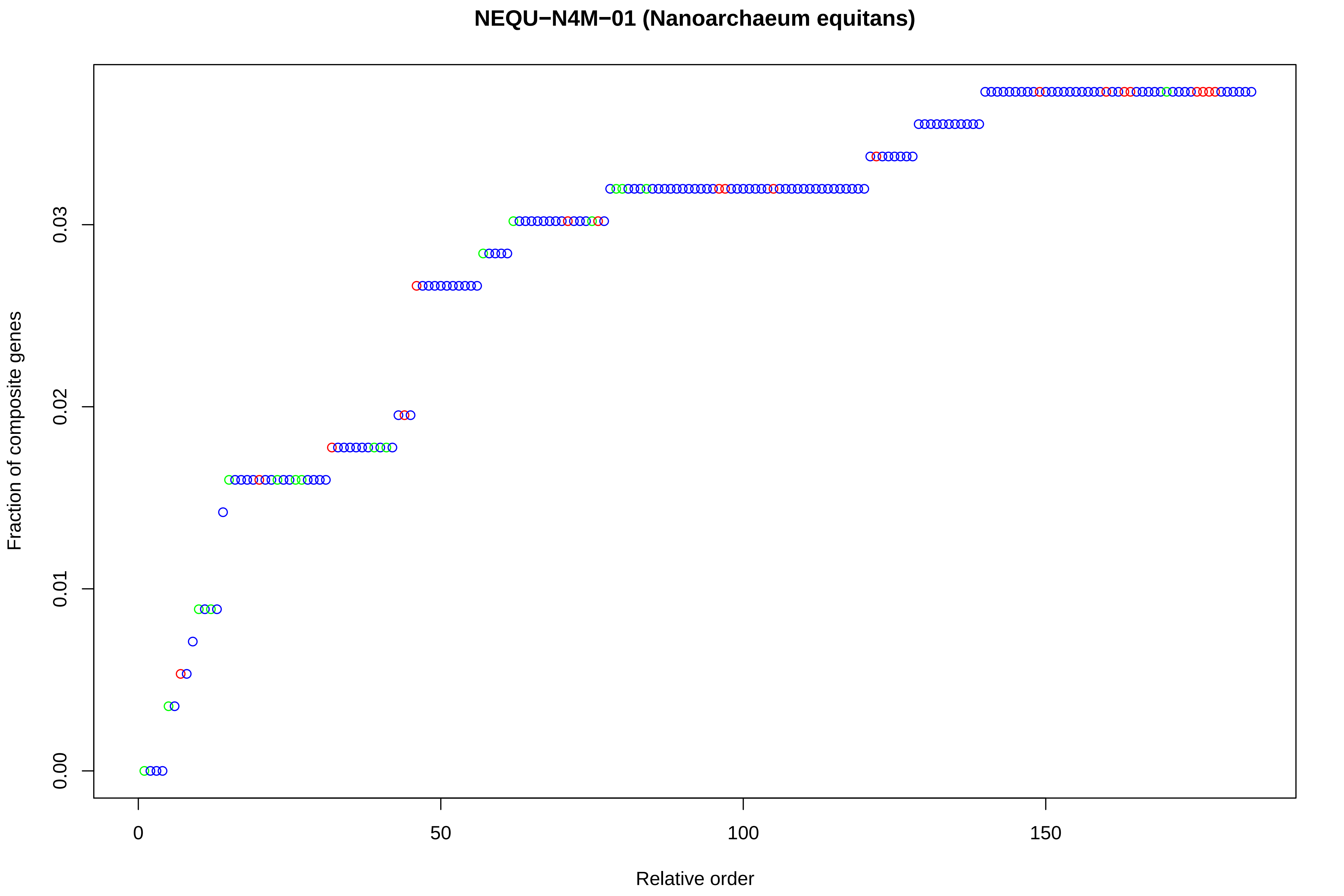

NEQU−N4M−01 (Nanoarchaeum equitans)
Fraction of composite genes
0.00
0.01
0.02
0.03
0
50
100
150
Relative order

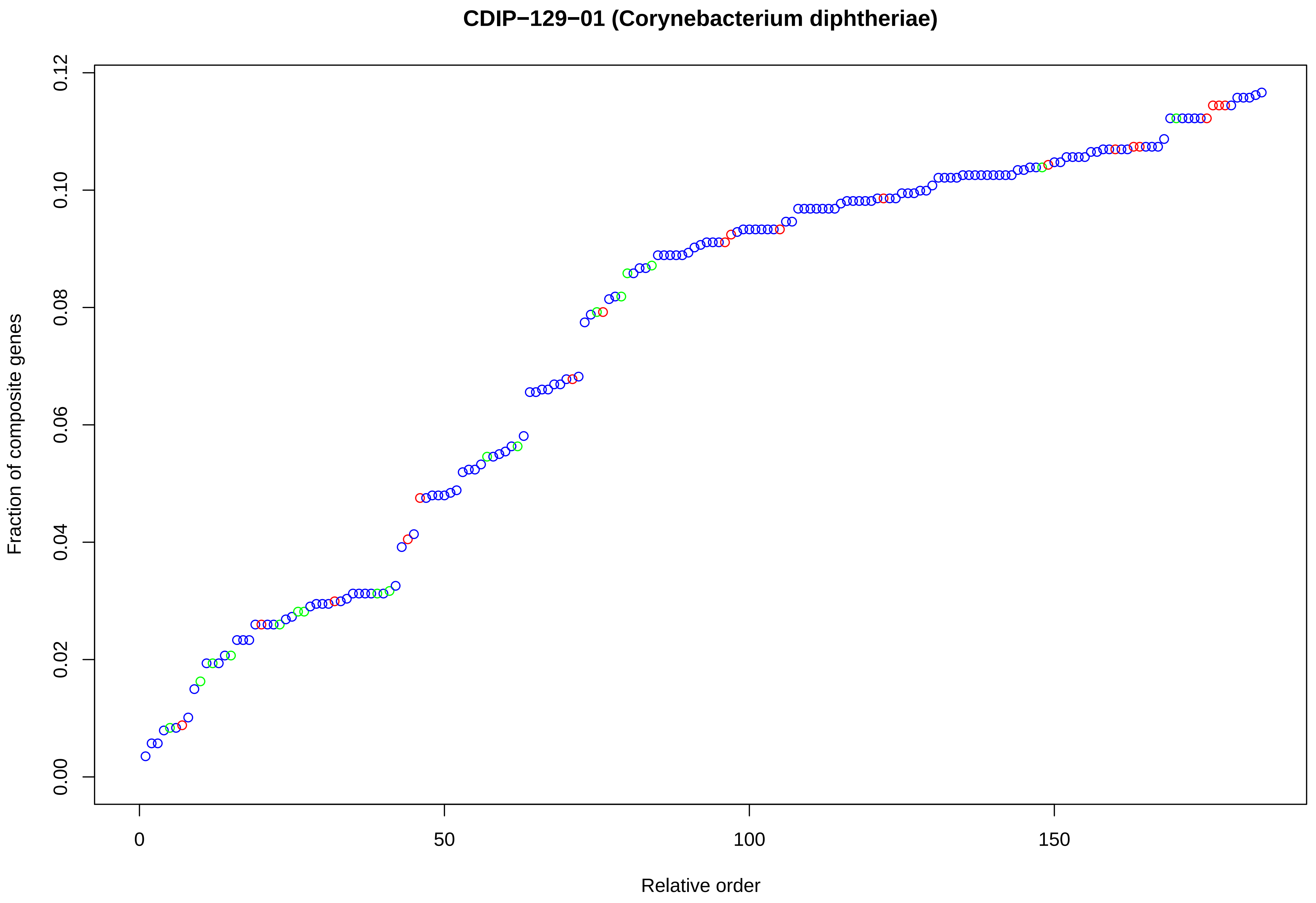
CDIP−129−01 (Corynebacterium diphtheriae)
Fraction of composite genes
0.00
0.02
0.04
0.06
0.08
0.10
0.12
Relative order
0
50
100
150

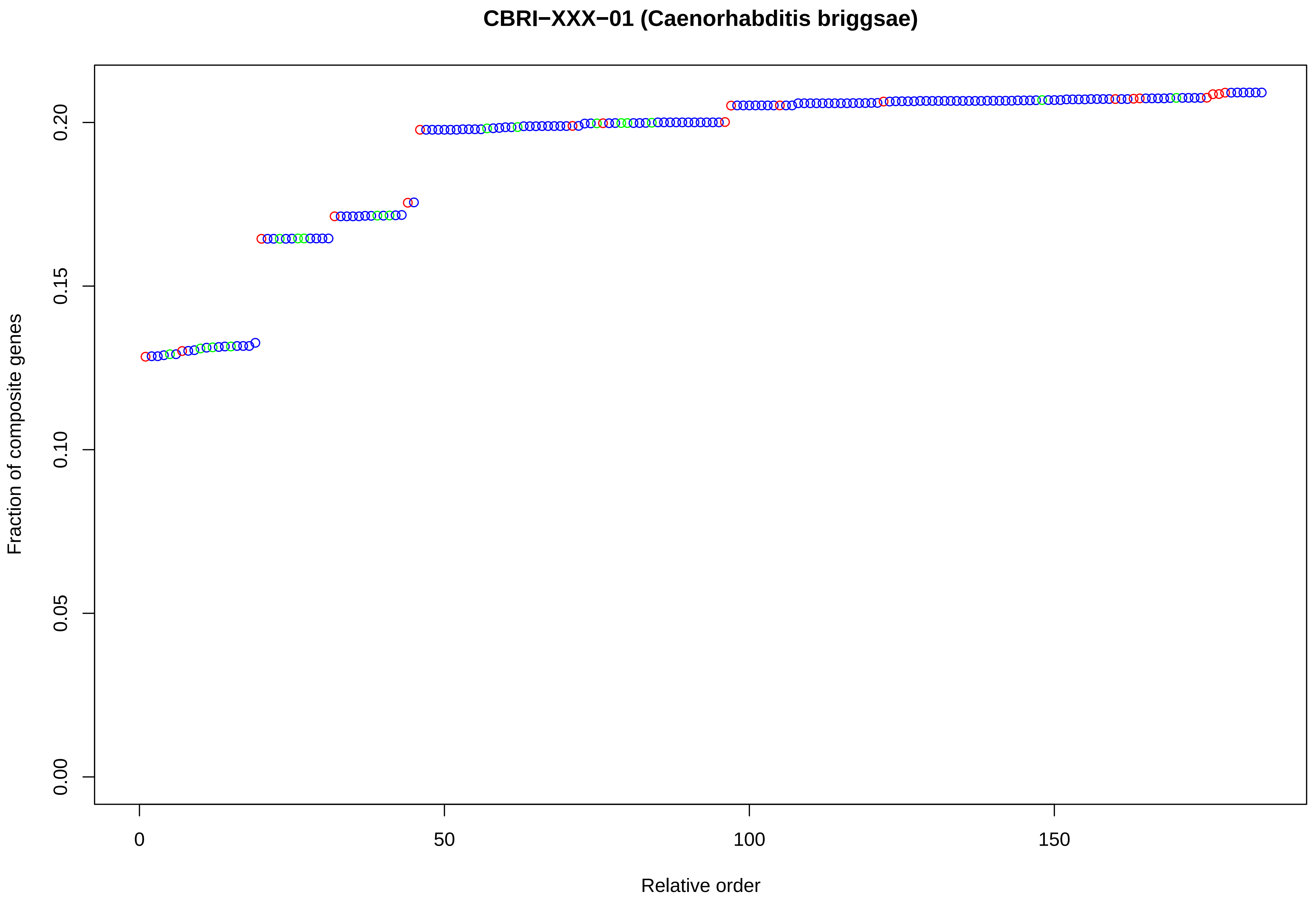

CBRI−XXX−01 (Caenorhabditis briggsae)
Fraction of composite genes
0.00
0.05
0.10
0.15
0.20
0
50
100
150
Relative order

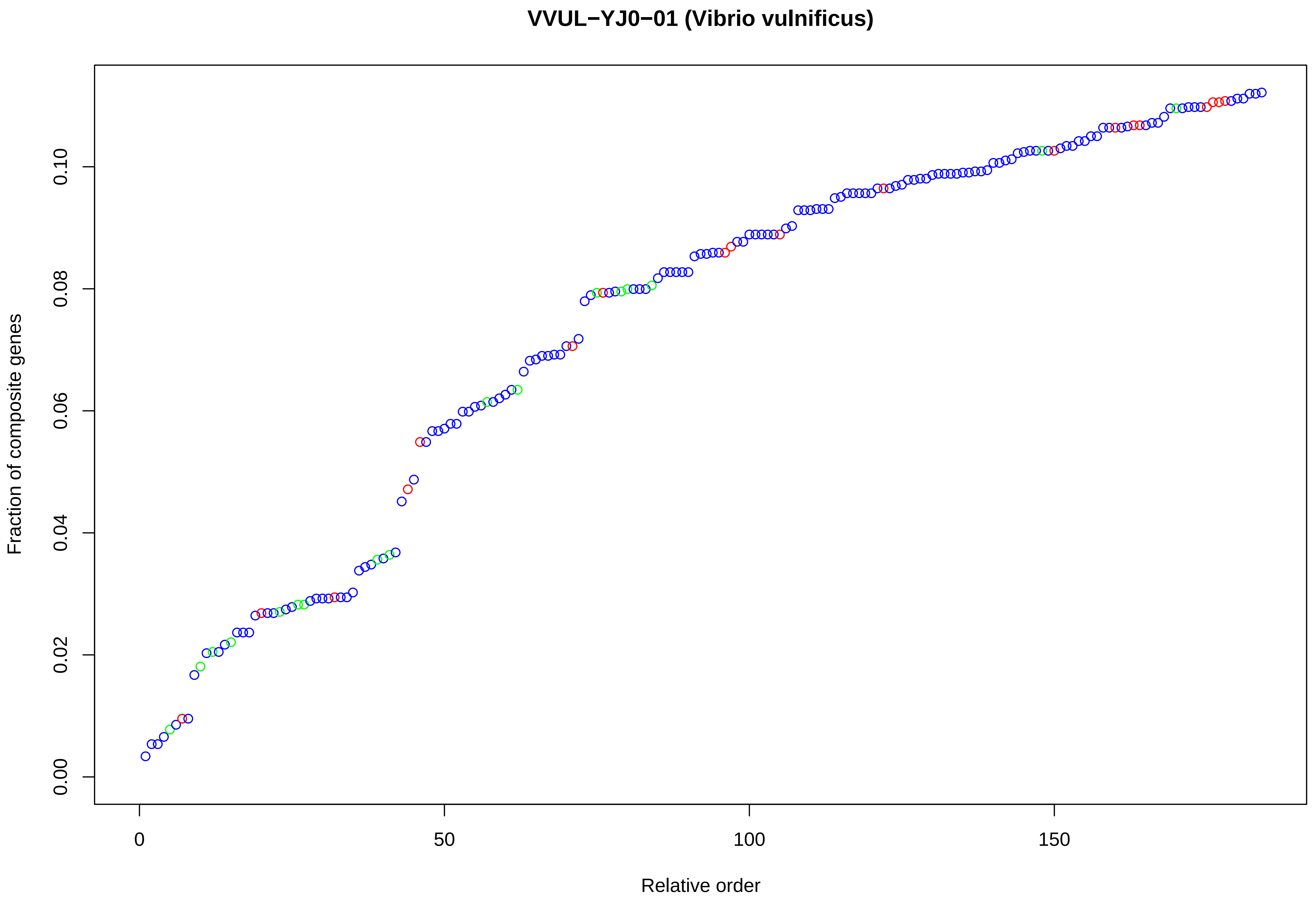
VVUL−YJ0−01 (Vibrio vulnificus)
Fraction of composite genes
0.00
0.02
0.04
0.06
0.08
0.10
Relative order
0
50
100
150

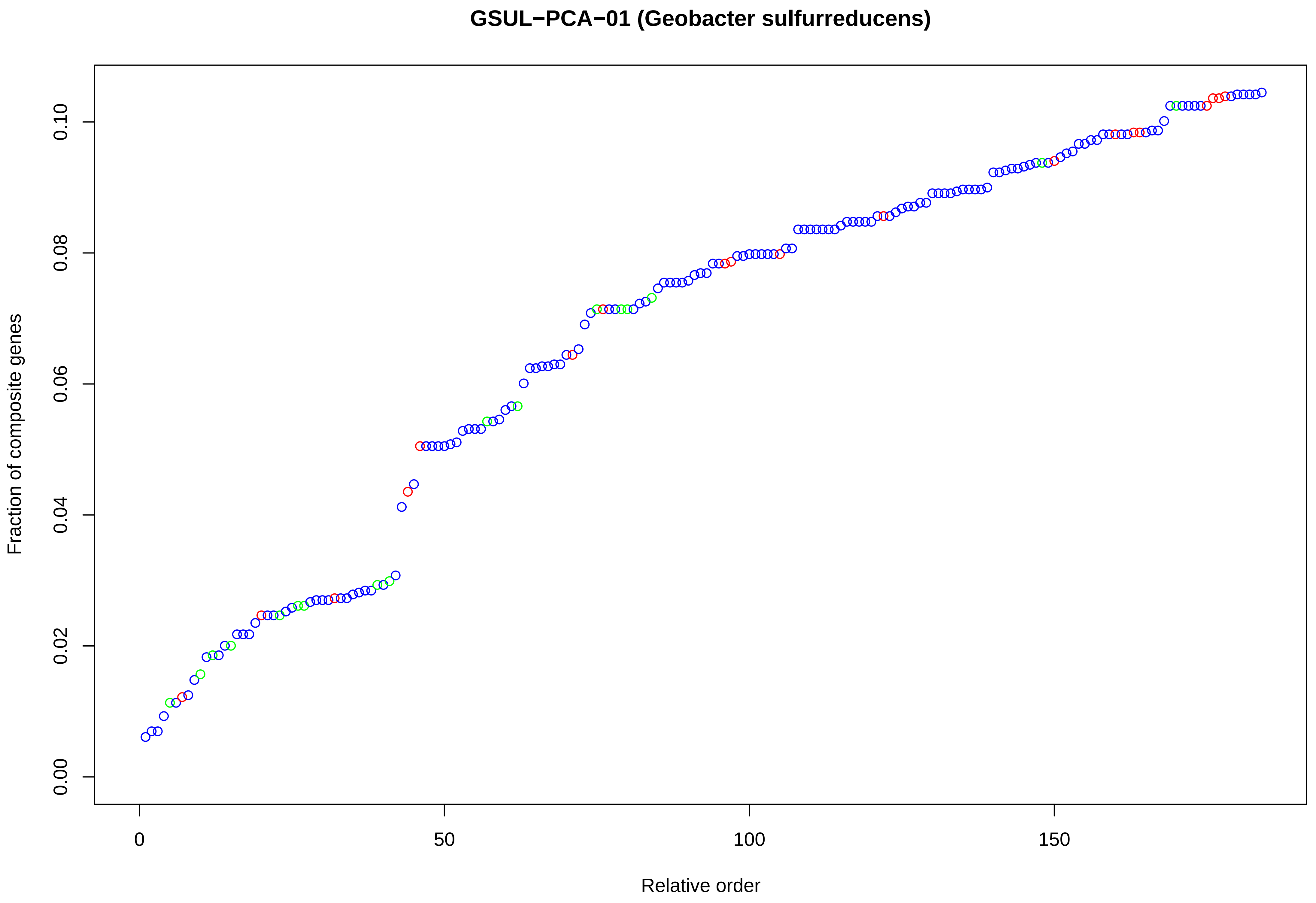

GSUL−PCA−01 (Geobacter sulfurreducens)
Fraction of composite genes
0.00
0.02
0.04
0.06
0.08
0.10
Relative order
0
50
100
150

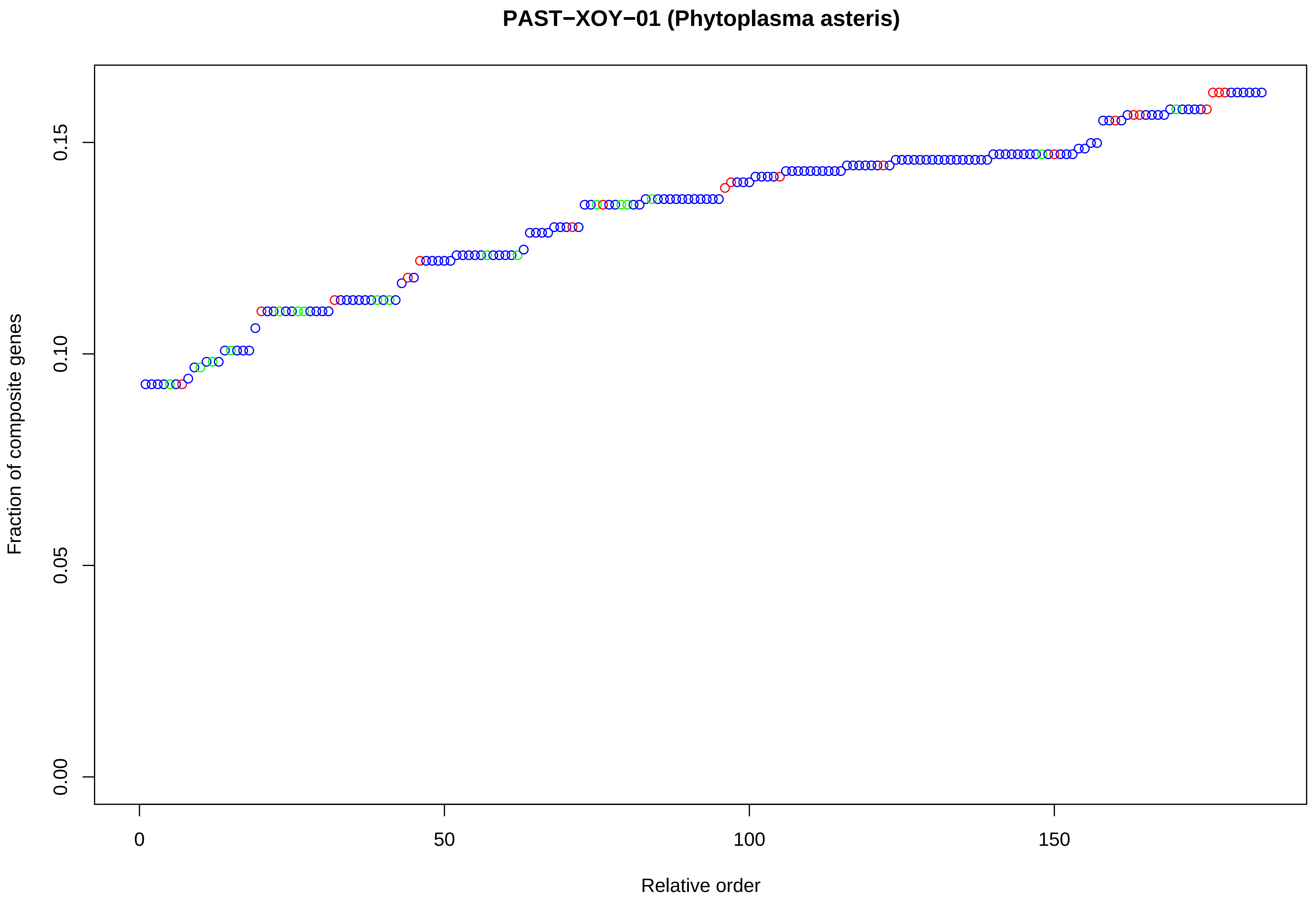
PAST−XOY−01 (Phytoplasma asteris)
Fraction of composite genes
0.00
0.05
0.10
0.15
0
50
100
150
Relative order

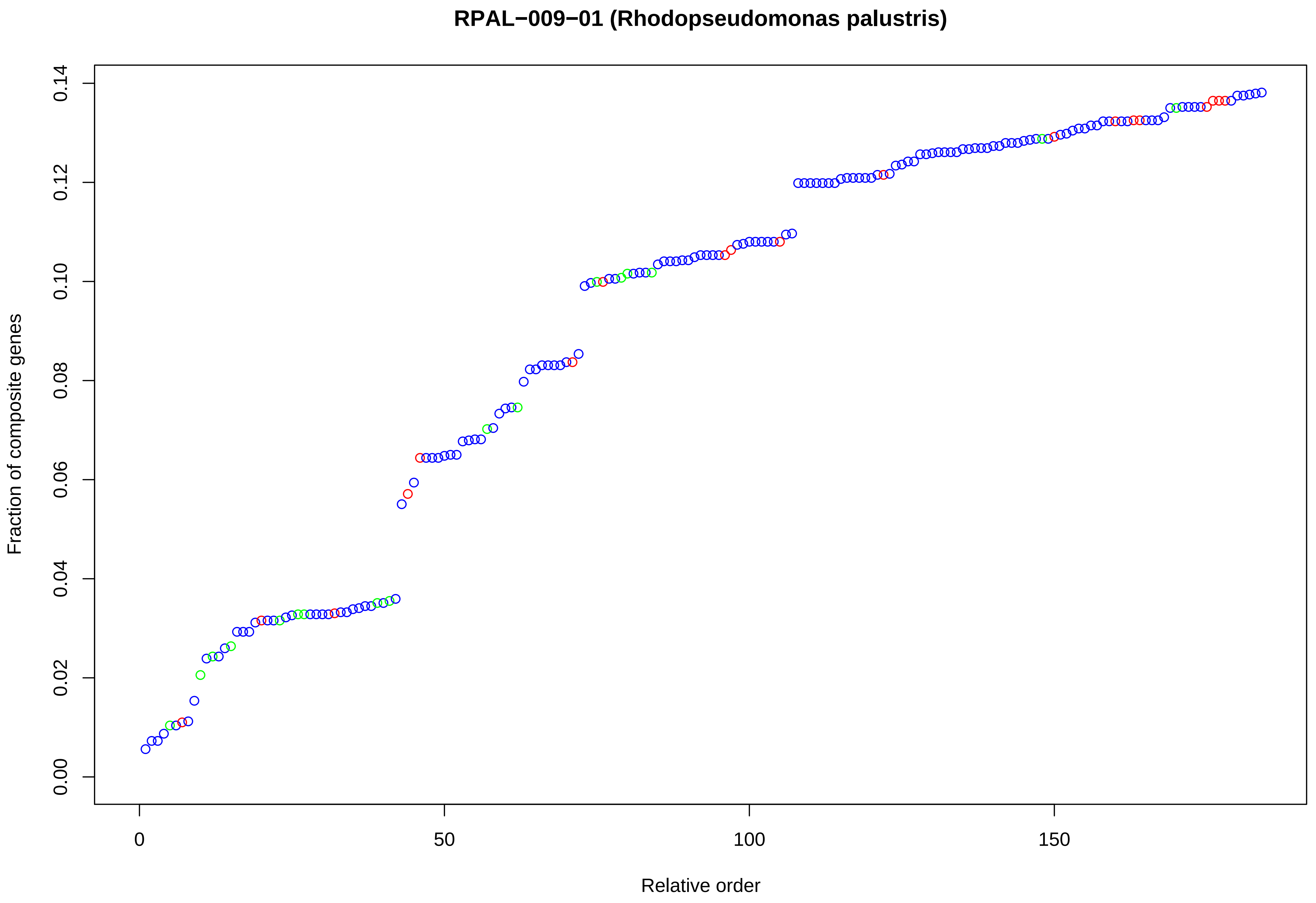

RPAL−009−01 (Rhodopseudomonas palustris)
Fraction of composite genes
0.00
0.02
0.04
0.06
0.08
0.10
0.12
0.14
0
50
100
150
Relative order

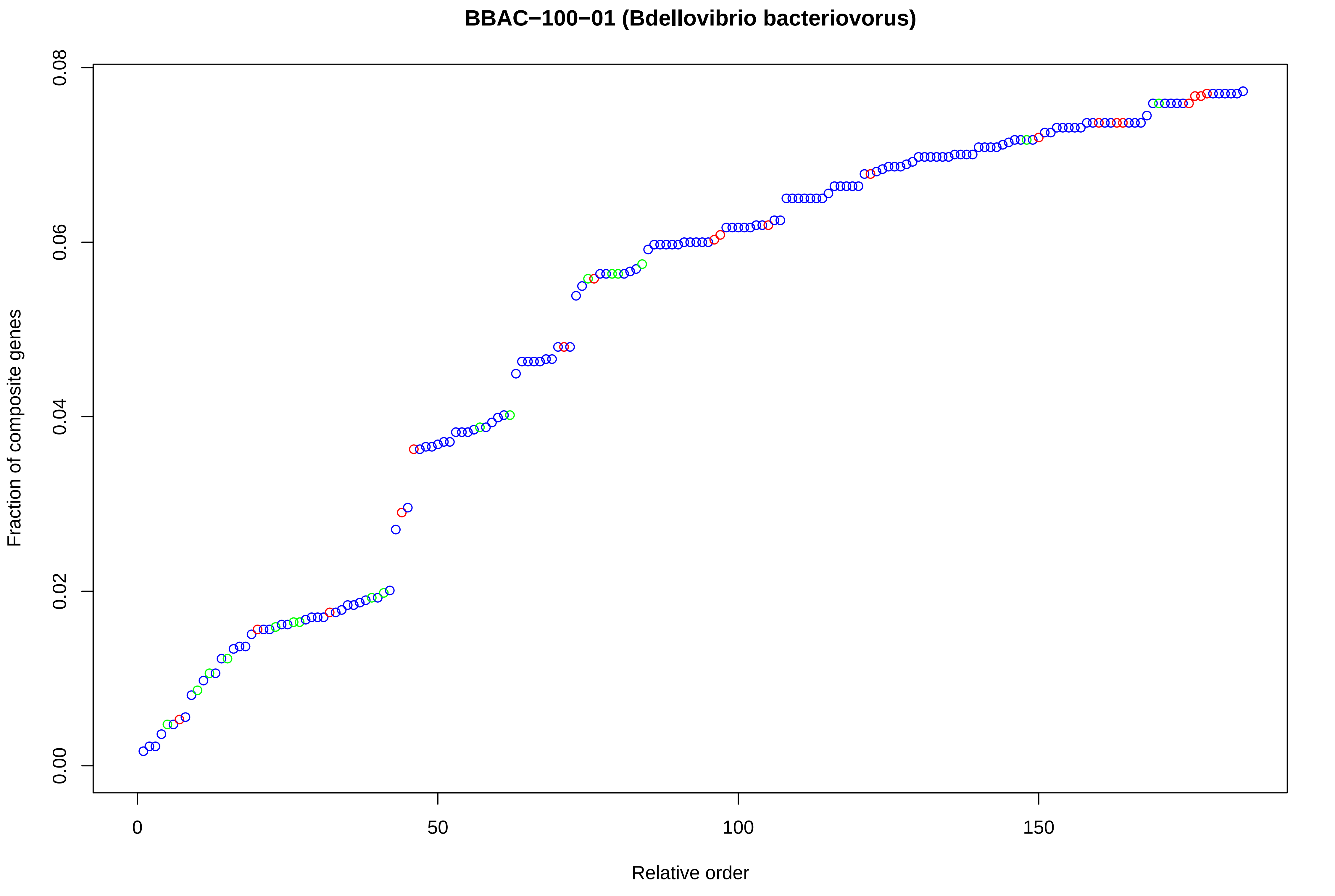

BBAC−100−01 (Bdellovibrio bacteriovorus)
Fraction of composite genes
0.00
0.02
0.04
0.06
0.08
0
50
100
150
Relative order

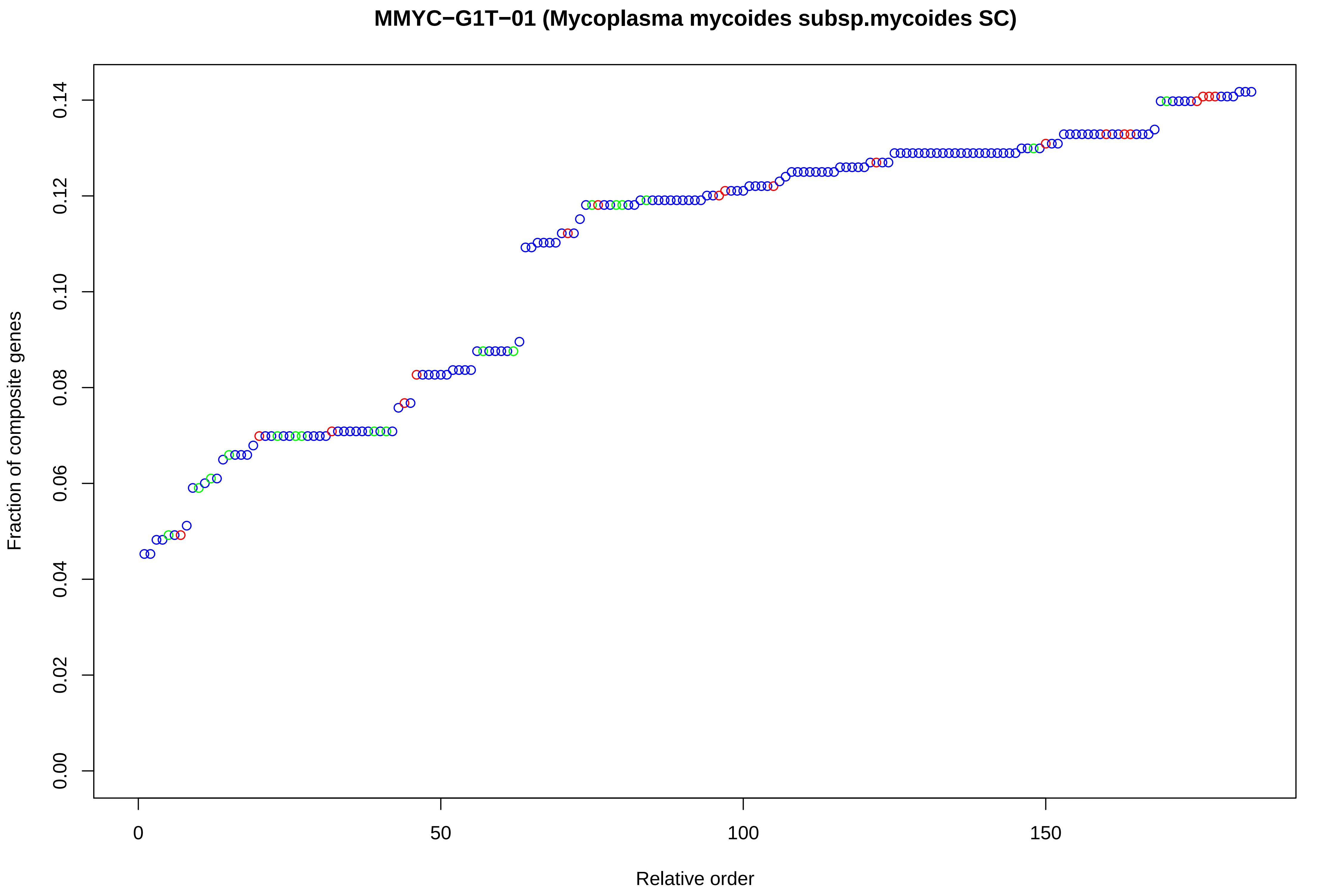
MMYC−G1T−01 (Mycoplasma mycoides subsp.mycoides SC)
Fraction of composite genes
0.00
0.02
0.04
0.06
0.08
0.10
0.12
0.14
Relative order
0
50
100
150

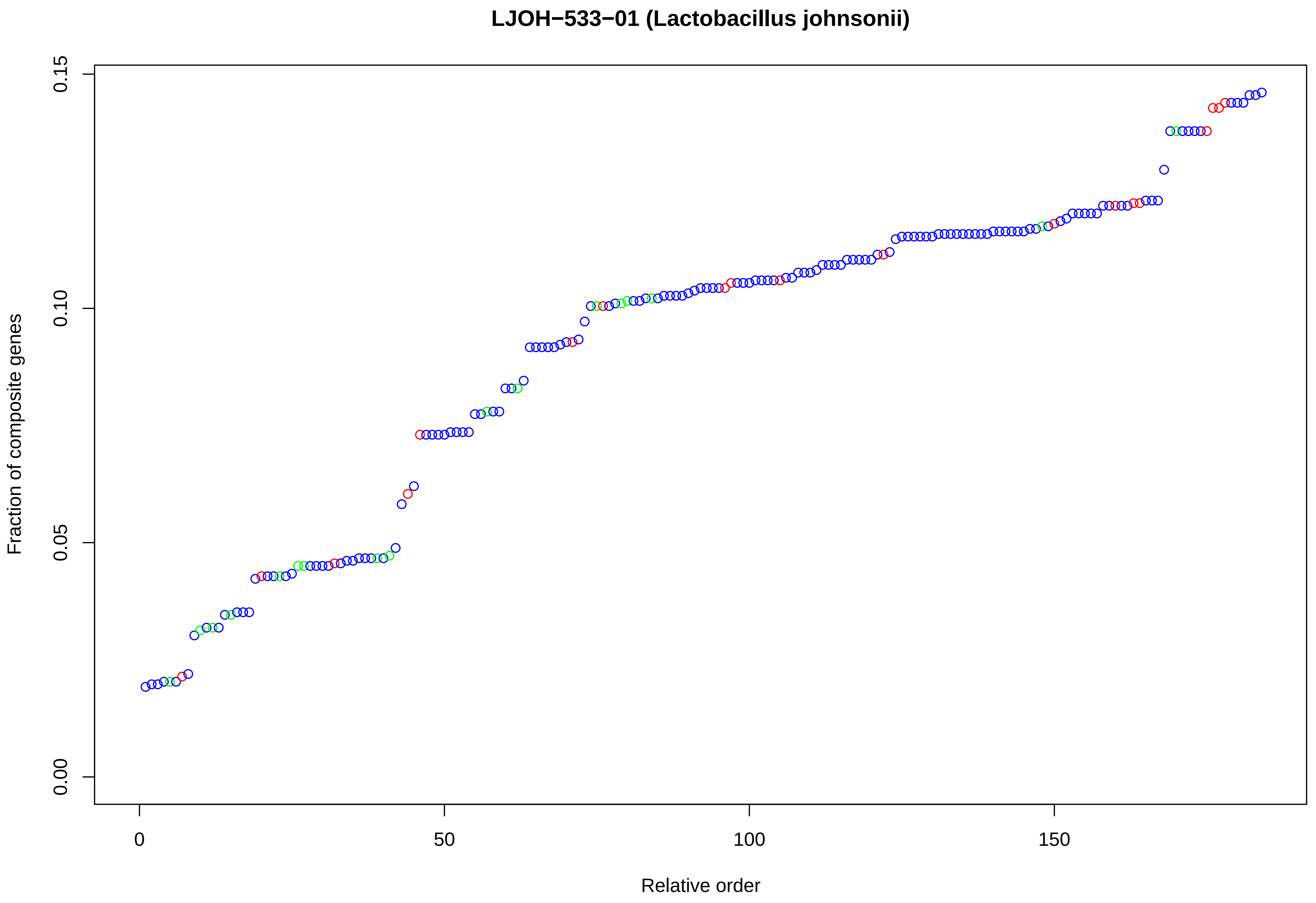
LJOH−533−01 (Lactobacillus johnsonii)
Fraction of composite genes
0.00
0.05
0.10
0.15
0
50
100
150
Relative order

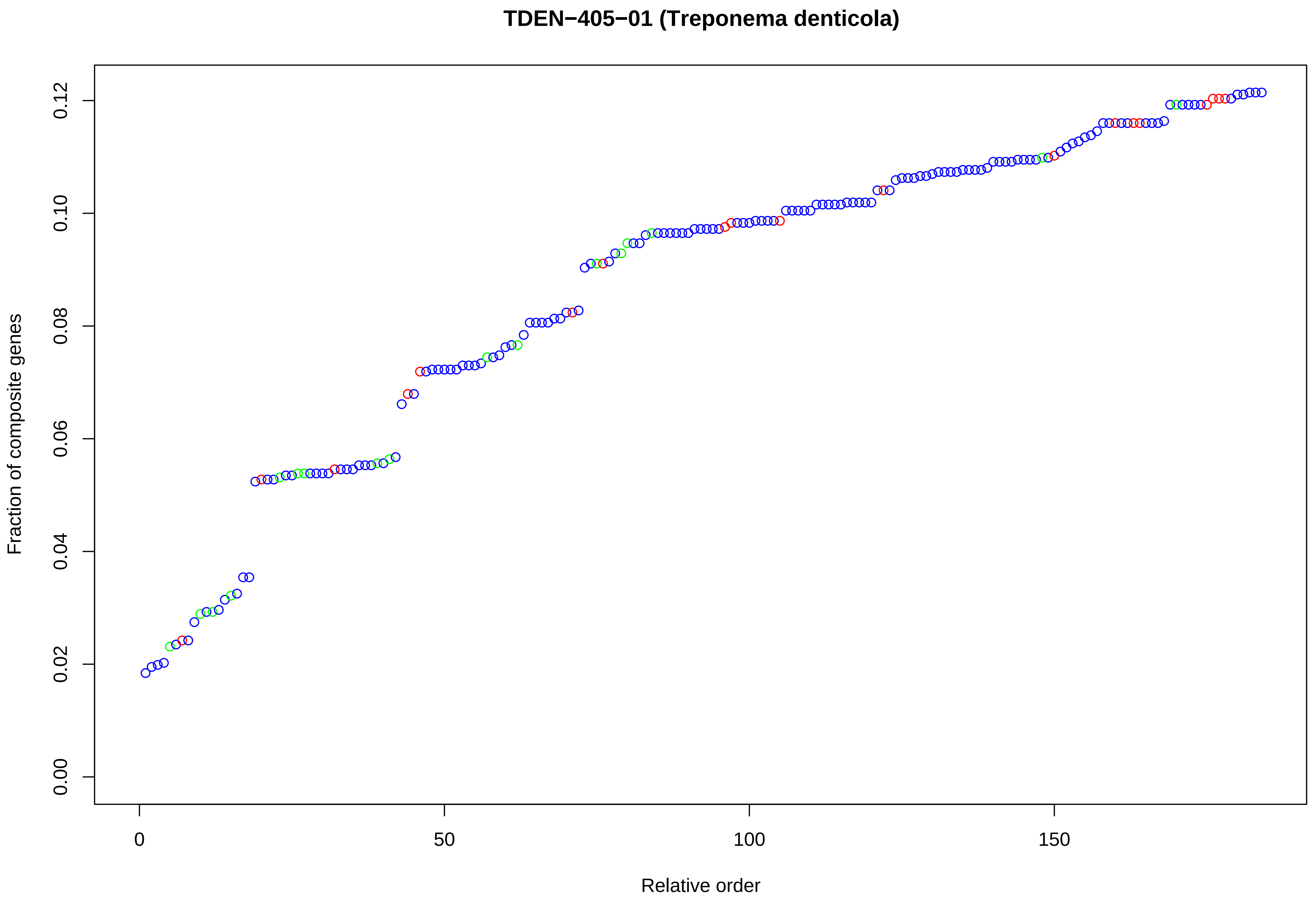
TDEN−405−01 (Treponema denticola)
Fraction of composite genes
0.00
0.02
0.04
0.06
0.08
0.10
0.12
Relative order
0
50
100
150

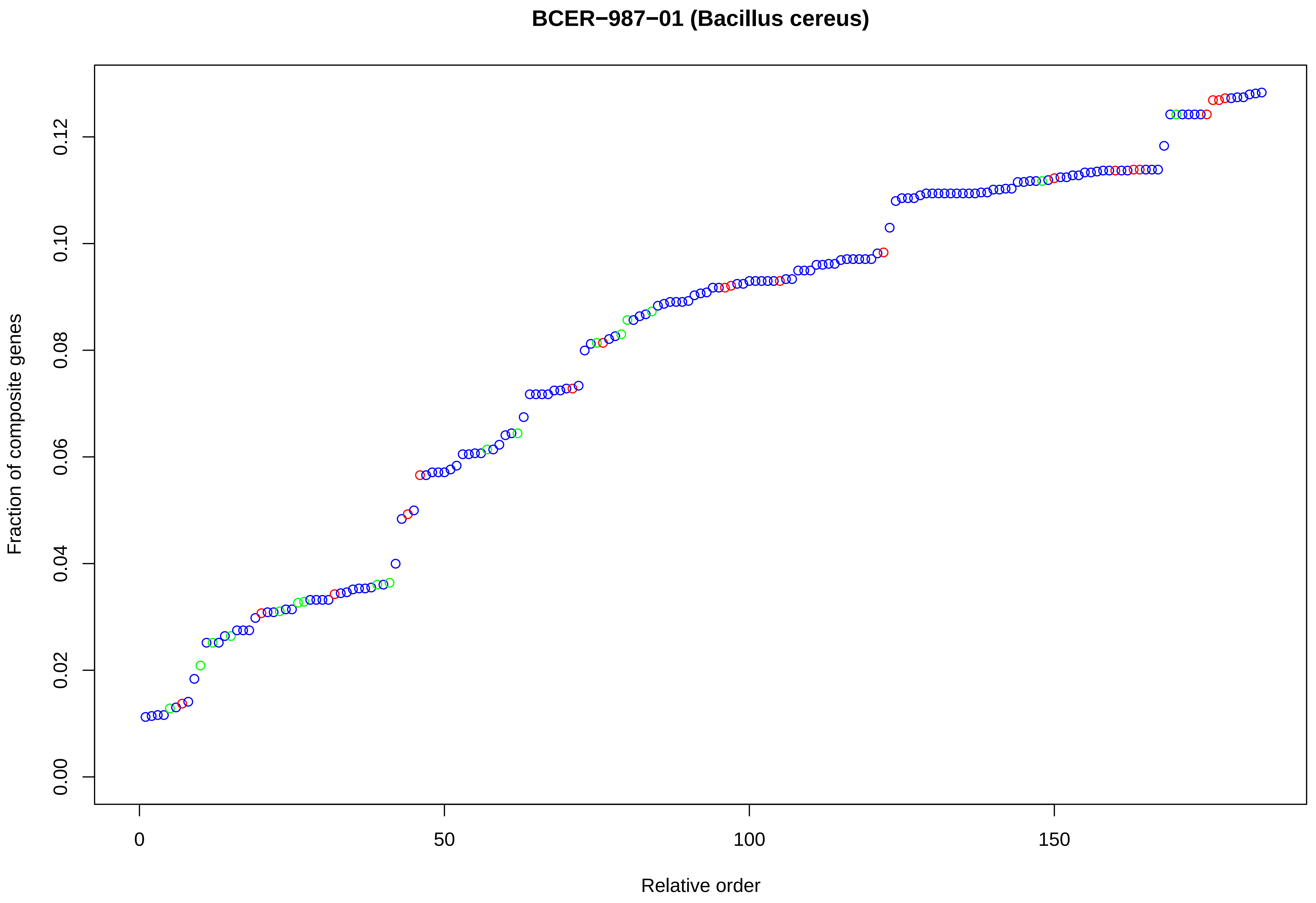

BCER−987−01 (Bacillus cereus)
Fraction of composite genes
0.00
0.02
0.04
0.06
0.08
0.10
0.12
0
50
100
150
Relative order

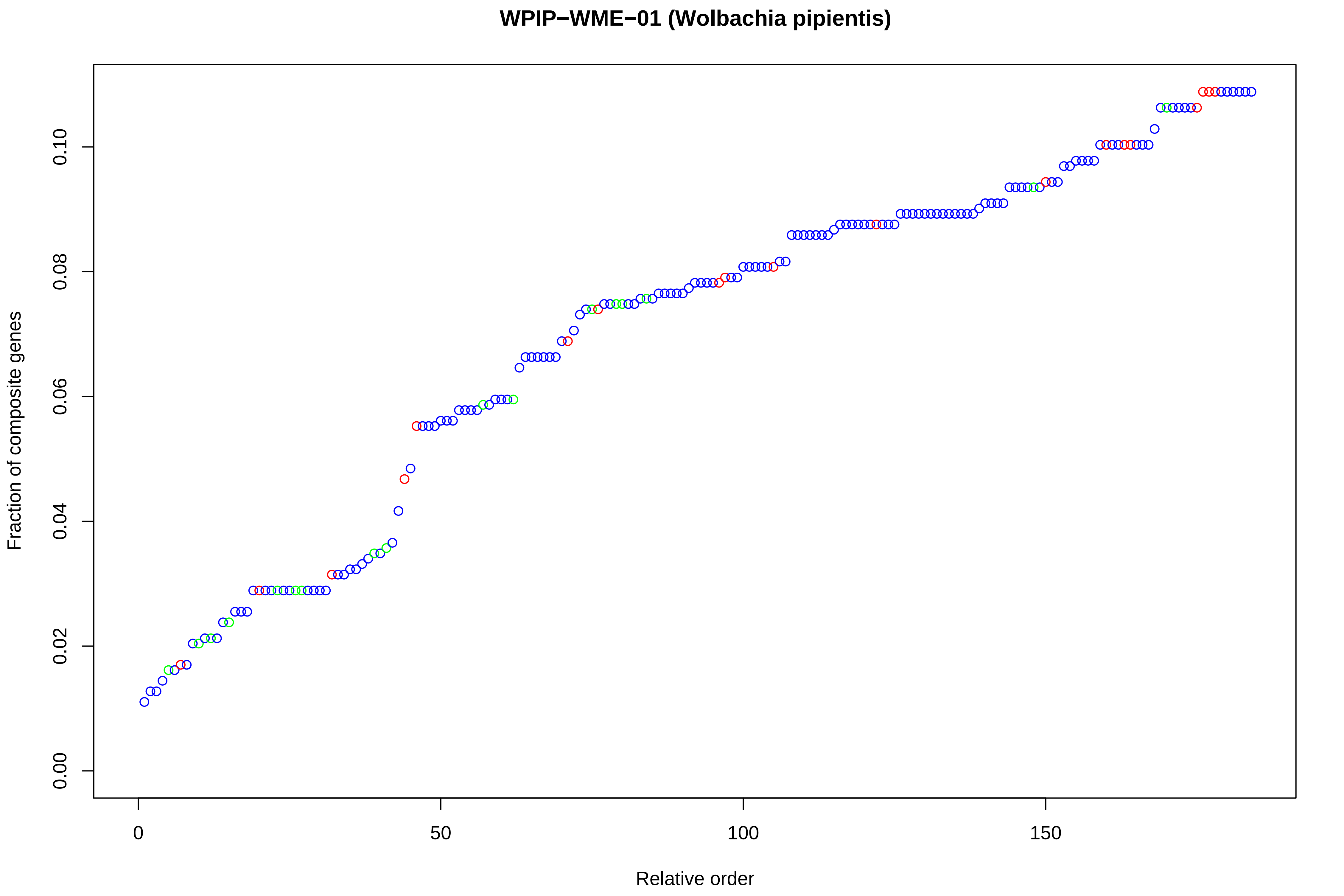
WPIP−WME−01 (Wolbachia pipientis)
Fraction of composite genes
Relative order
0.00
0.02
0.04
0.06
0.08
0.10
0
50
100
150

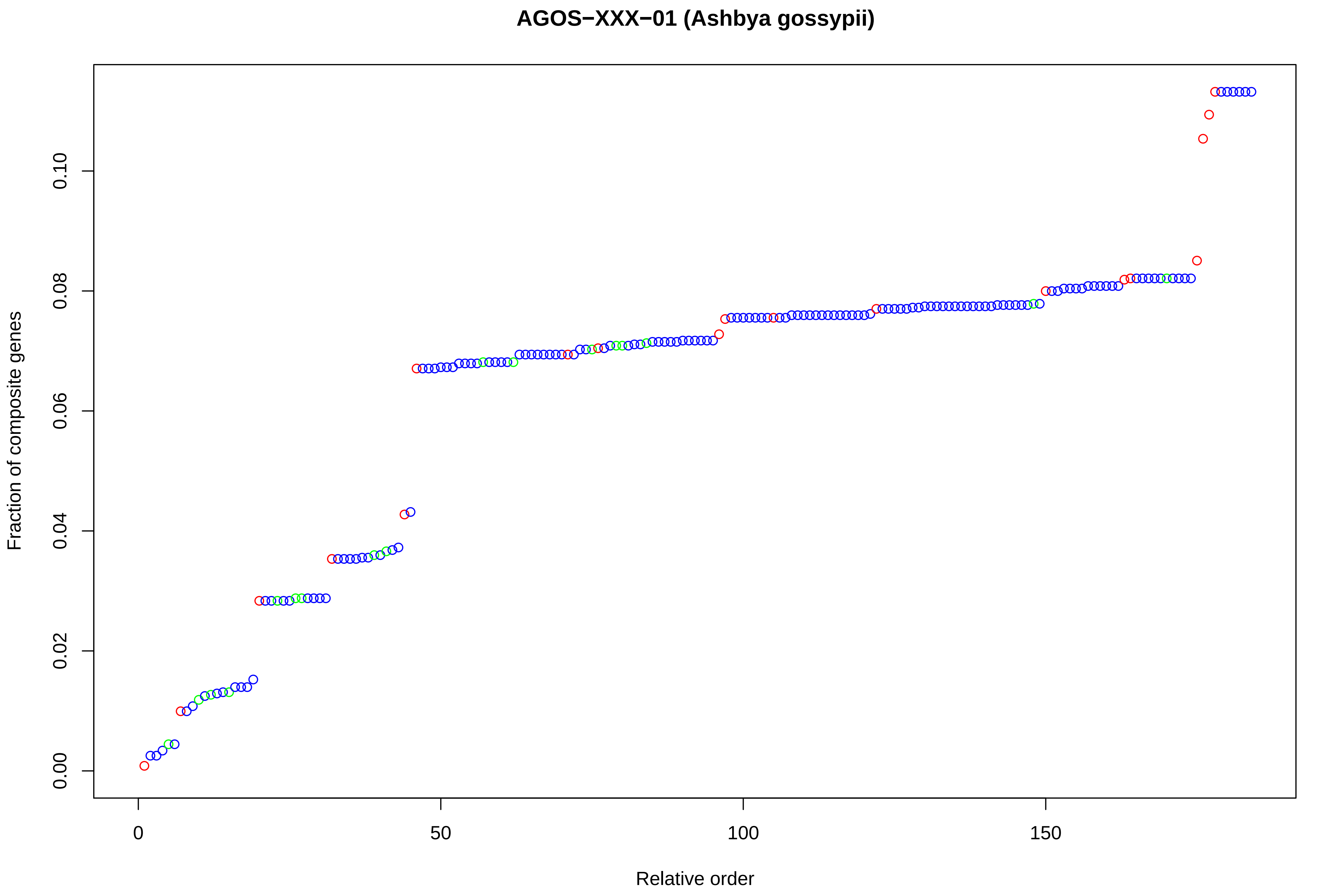
AGOS−XXX−01 (Ashbya gossypii)
Fraction of composite genes
0.00
0.02
0.04
0.06
0.08
0.10
Relative order
0
50
100
150

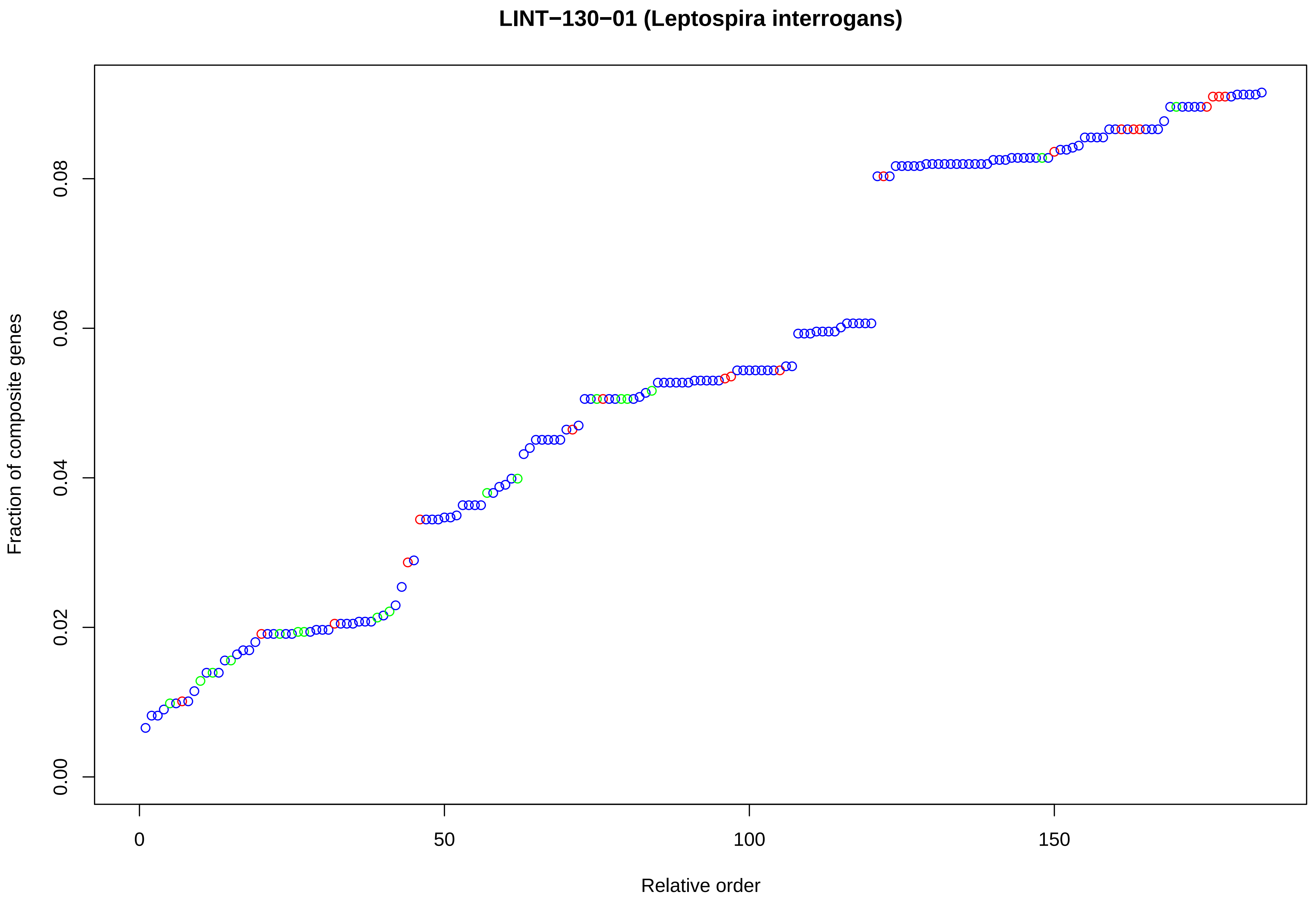
LINT−130−01 (Leptospira interrogans)
Fraction of composite genes
0.00
0.02
0.04
0.06
0.08
0
50
100
150
Relative order

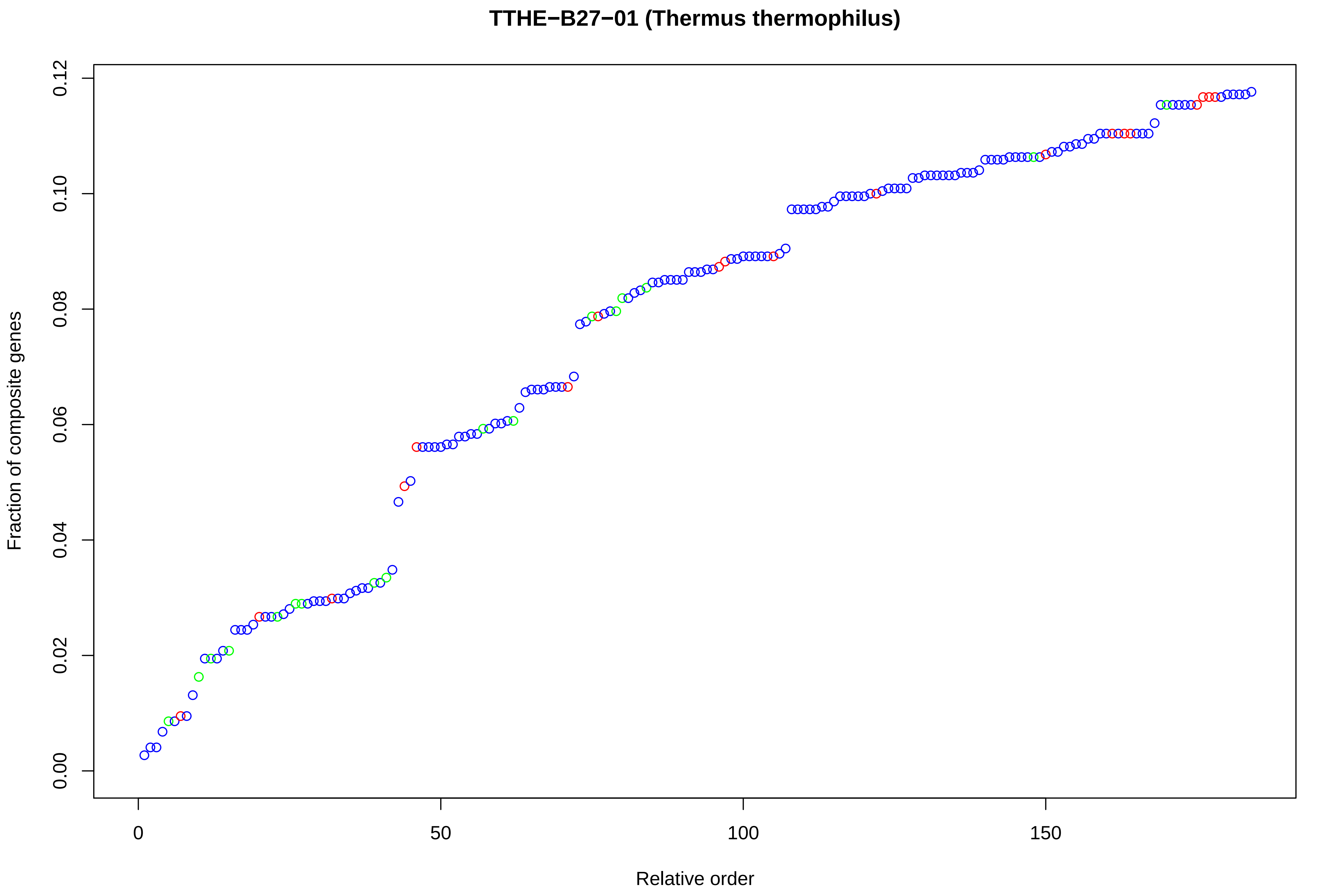
TTHE−B27−01 (Thermus thermophilus)
Fraction of composite genes
Relative order
0.00
0.02
0.04
0.06
0.08
0.10
0.12
0
50
100
150

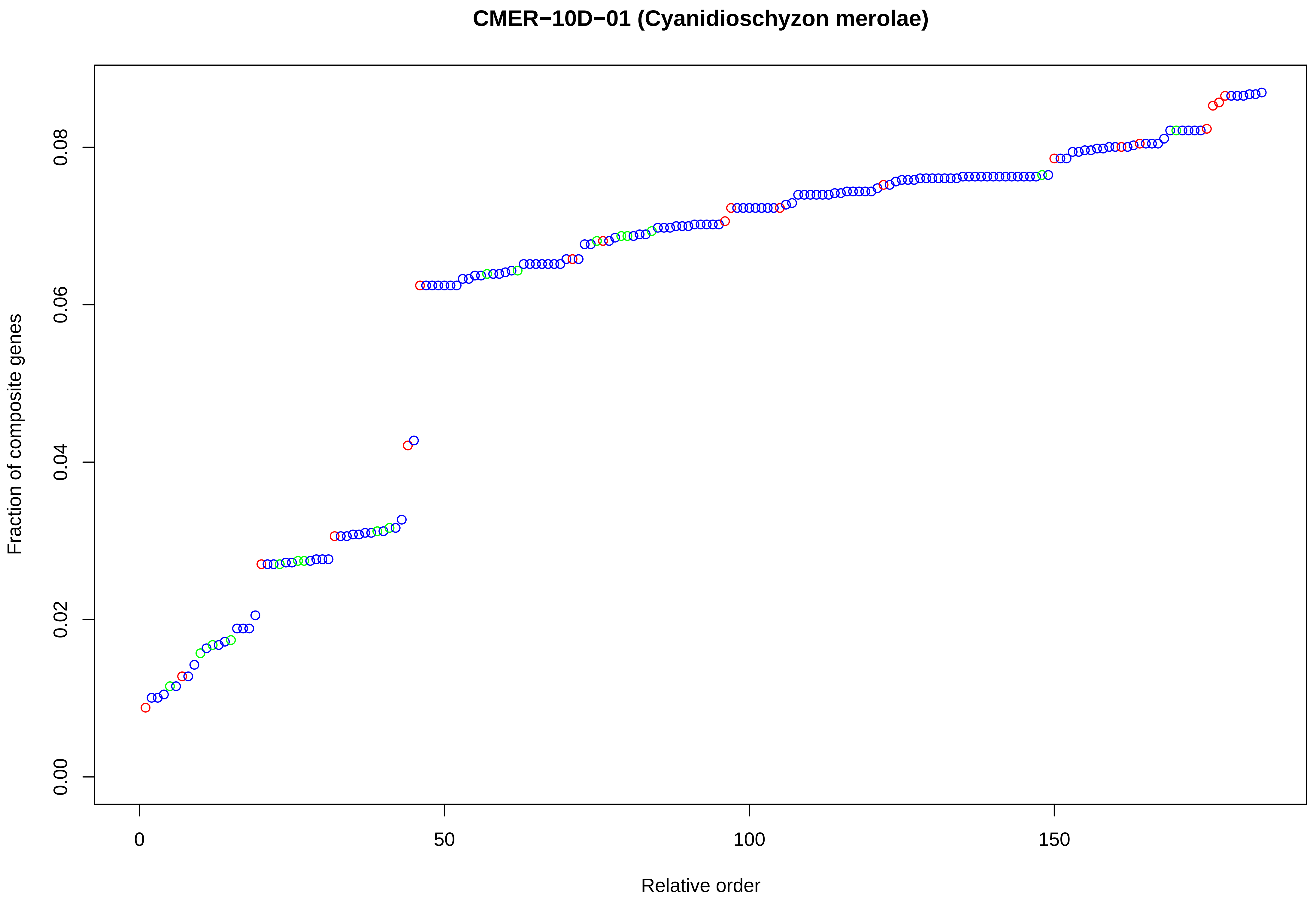
CMER−10D−01 (Cyanidioschyzon merolae)
Fraction of composite genes
0.00
0.02
0.04
0.06
0.08
0
50
100
150
Relative order

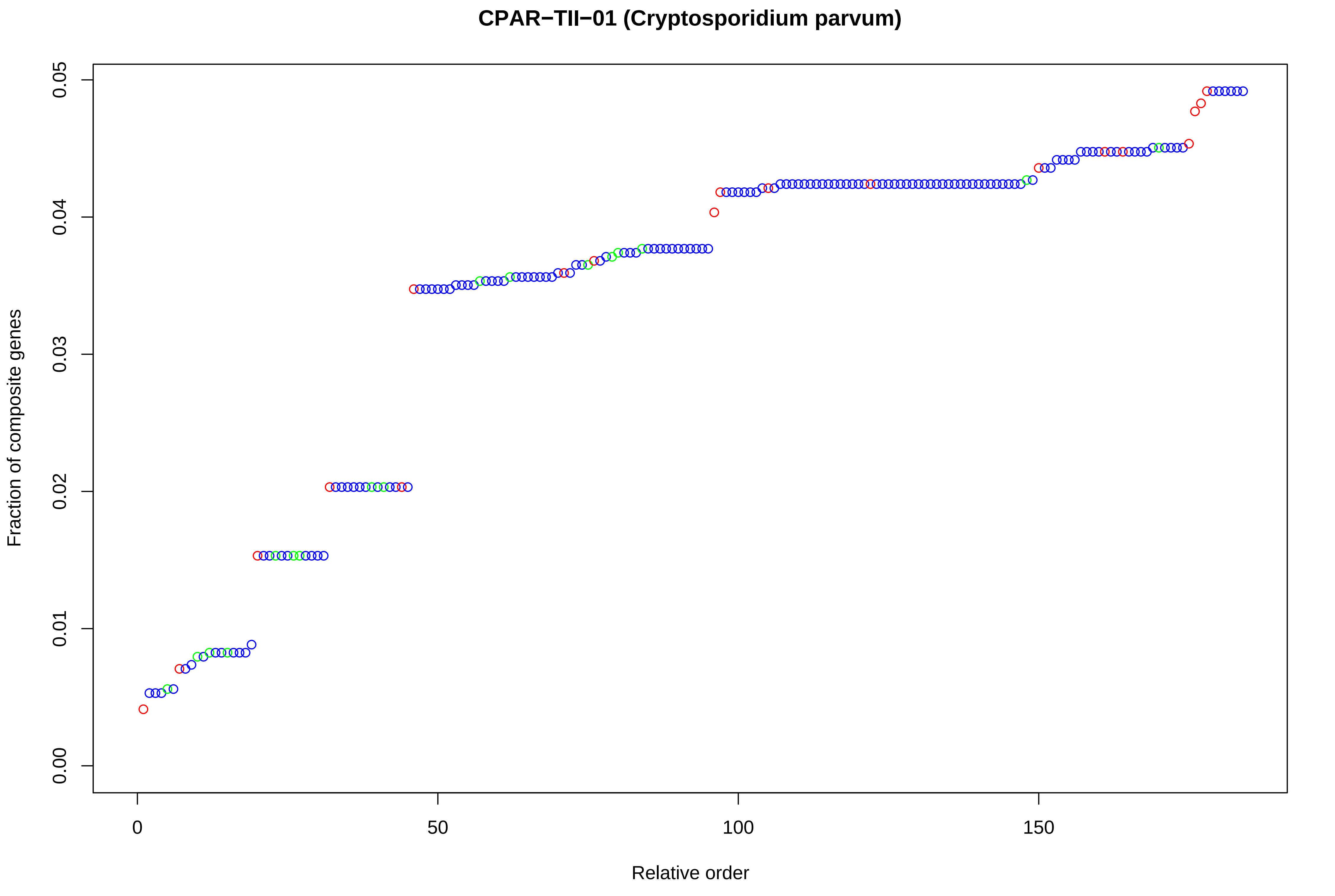

CPAR−TII−01 (Cryptosporidium parvum)
Fraction of composite genes
0.00
0.01
0.02
0.03
0.04
0.05
0
50
100
150
Relative order

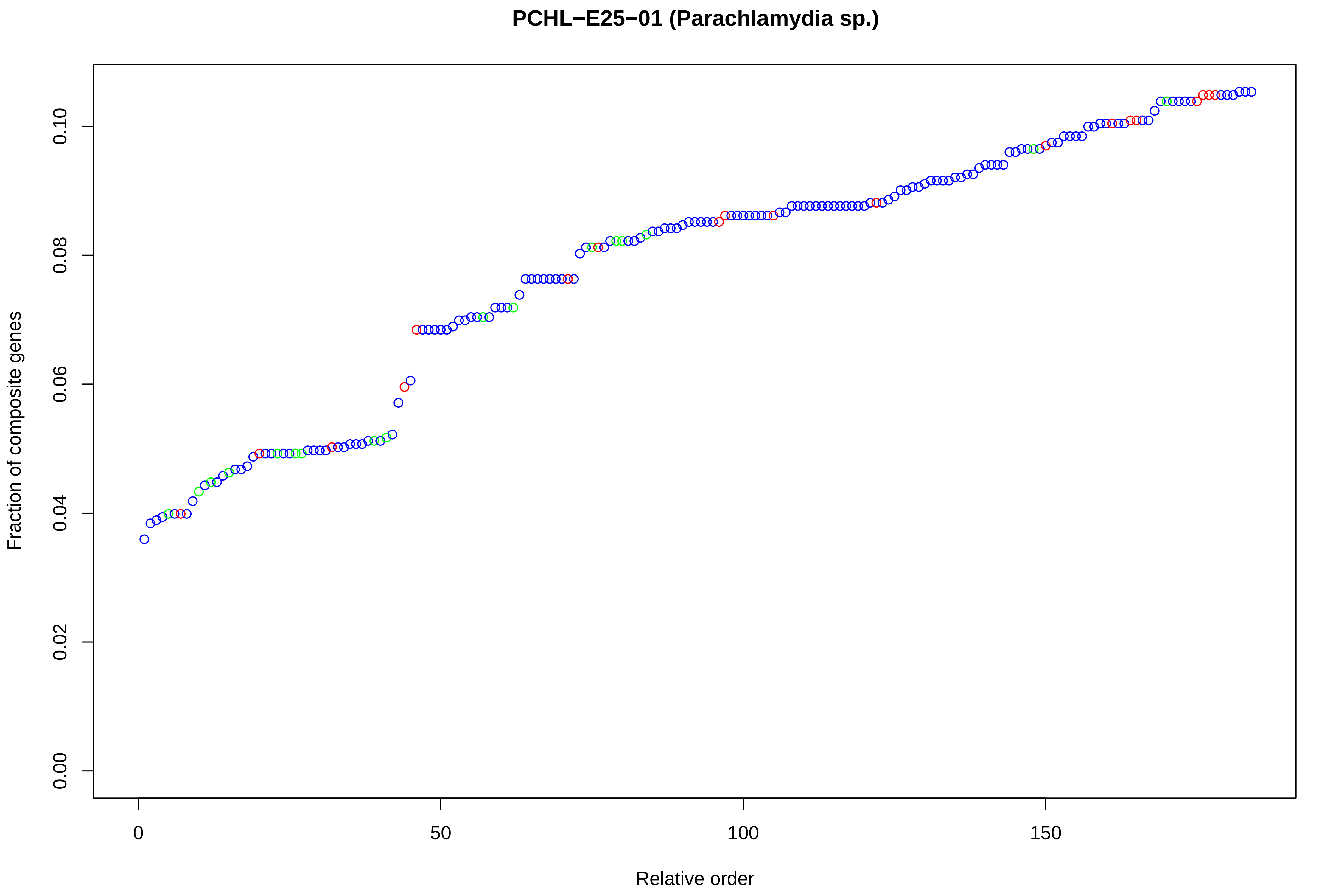
PCHL−E25−01 (Parachlamydia sp.)
Fraction of composite genes
0.00
0.02
0.04
0.06
0.08
0.10
Relative order
0
50
100
150

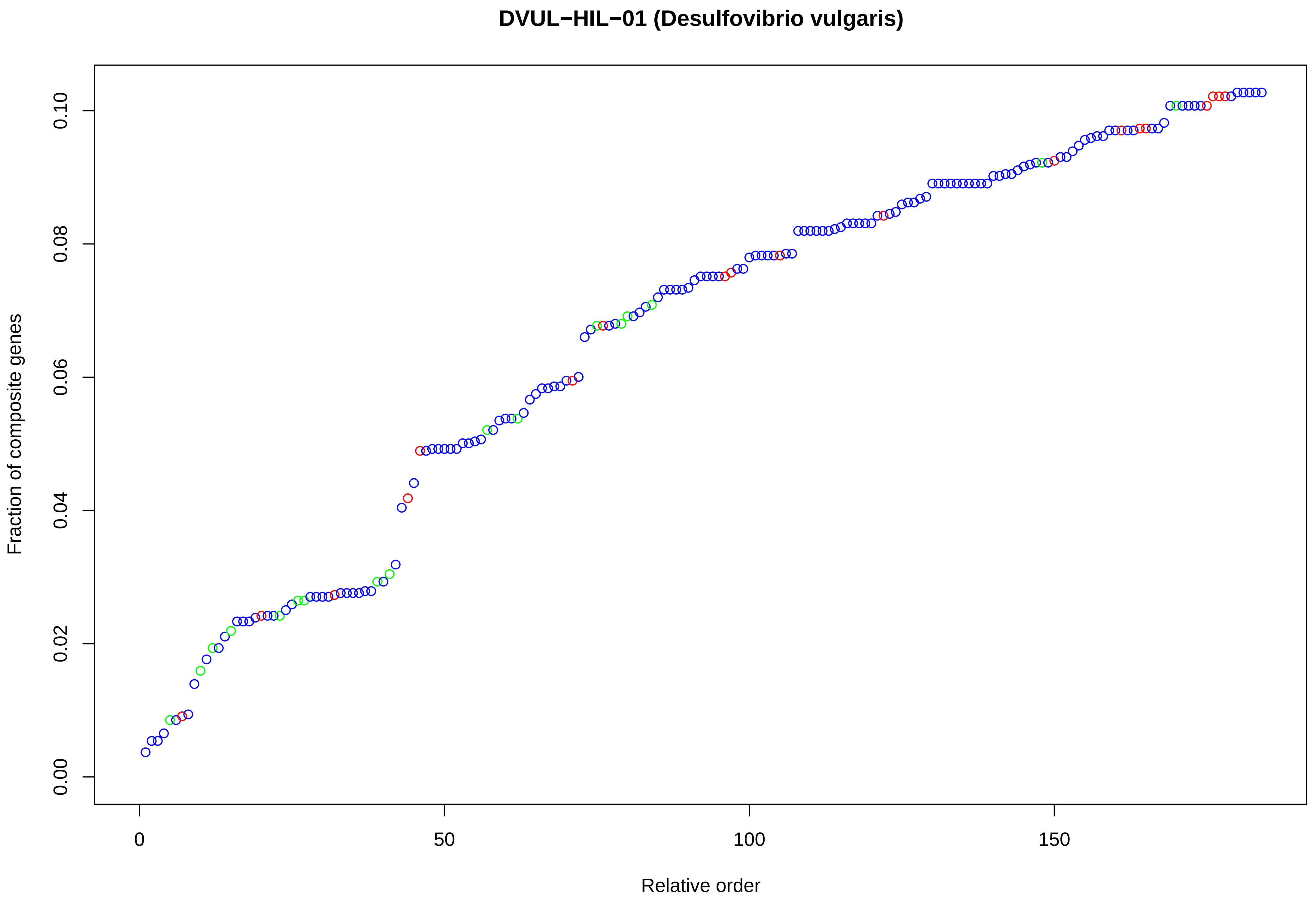
DVUL−HIL−01 (Desulfovibrio vulgaris)
Fraction of composite genes
0.00
0.02
0.04
0.06
0.08
0.10
0
50
100
150
Relative order

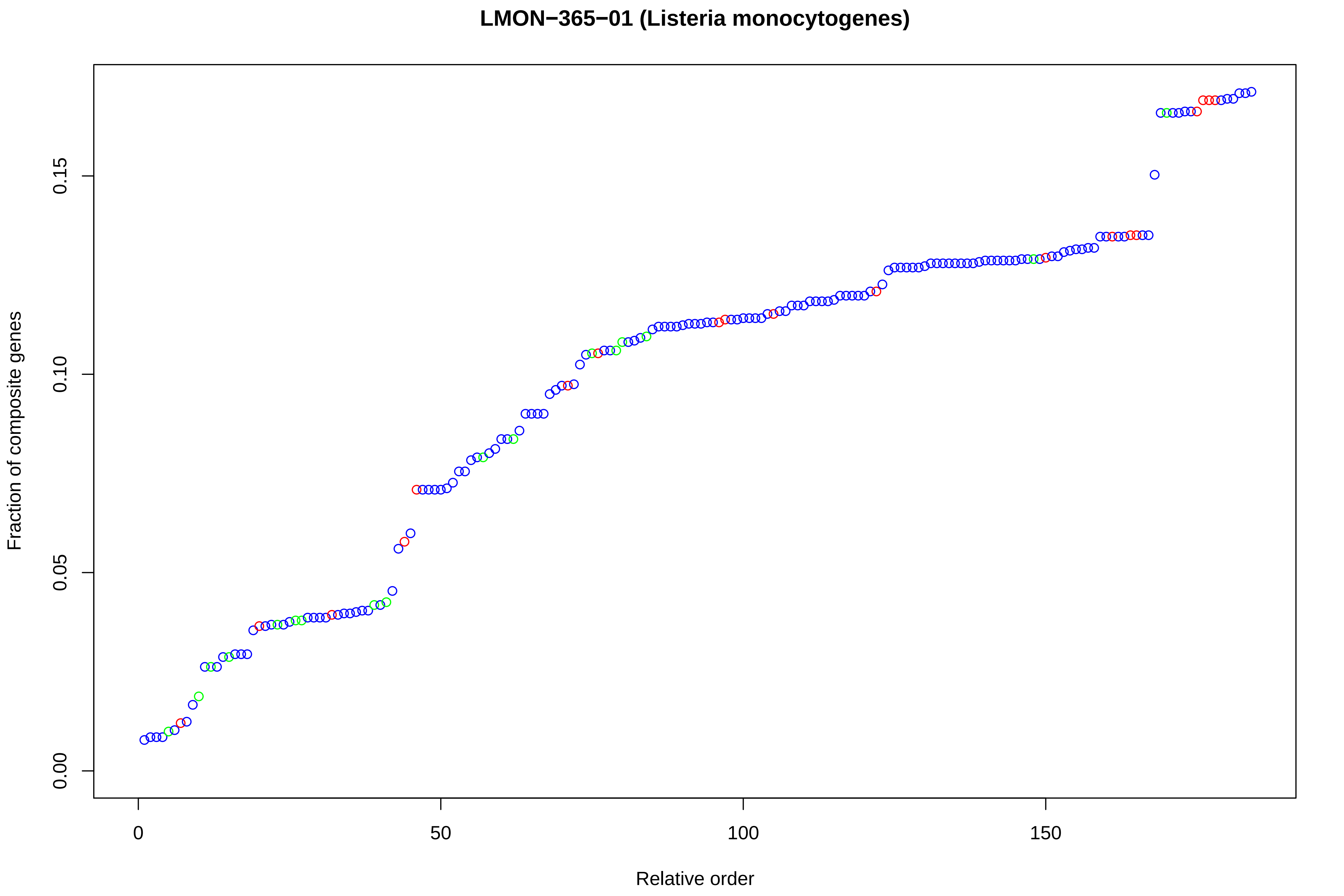

LMON−365−01 (Listeria monocytogenes)
Fraction of composite genes
0.00
0.05
0.10
0.15
0
50
100
150
Relative order

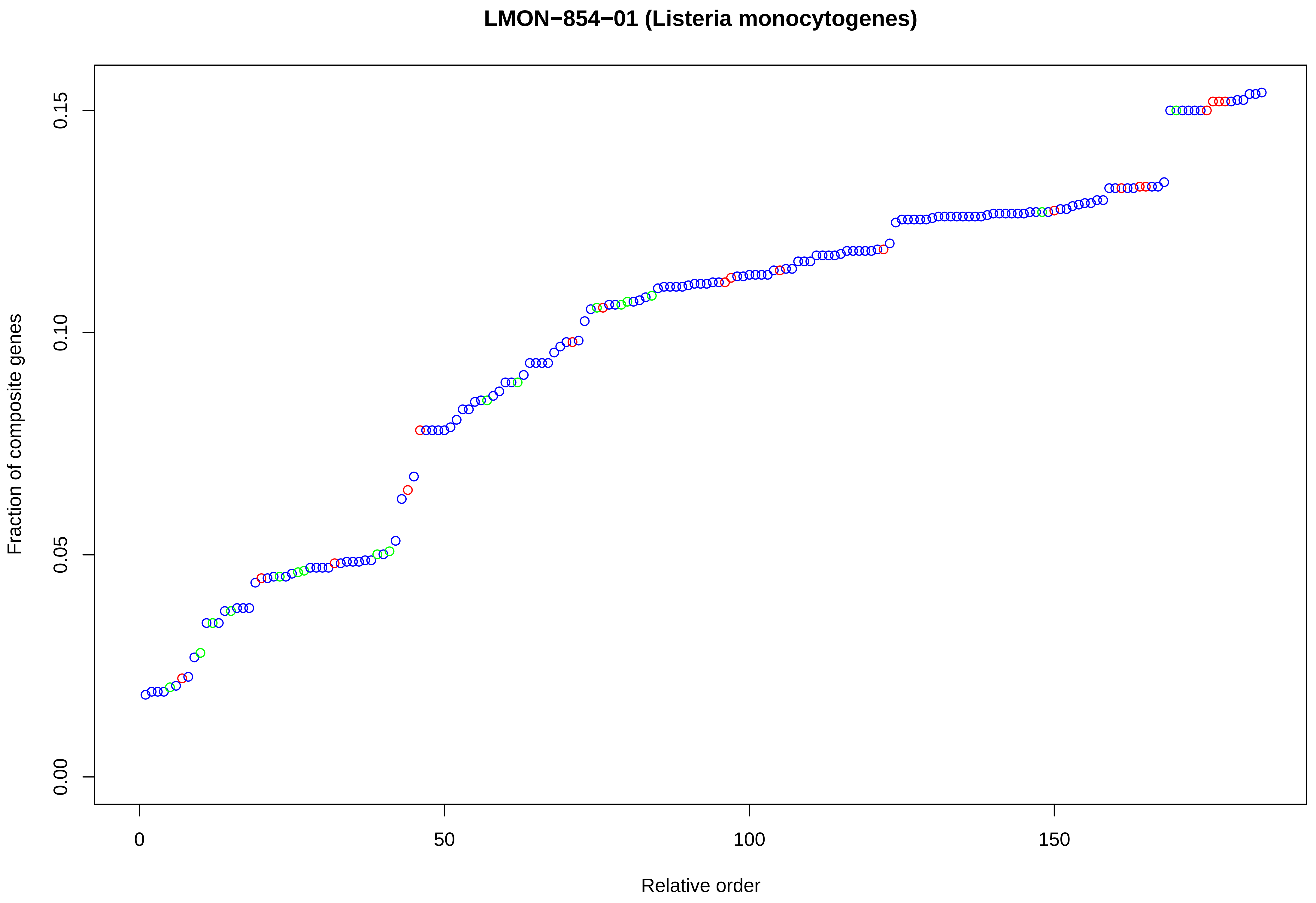
LMON−854−01 (Listeria monocytogenes)
Fraction of composite genes
0.00
0.05
0.10
0.15
Relative order
0
50
100
150

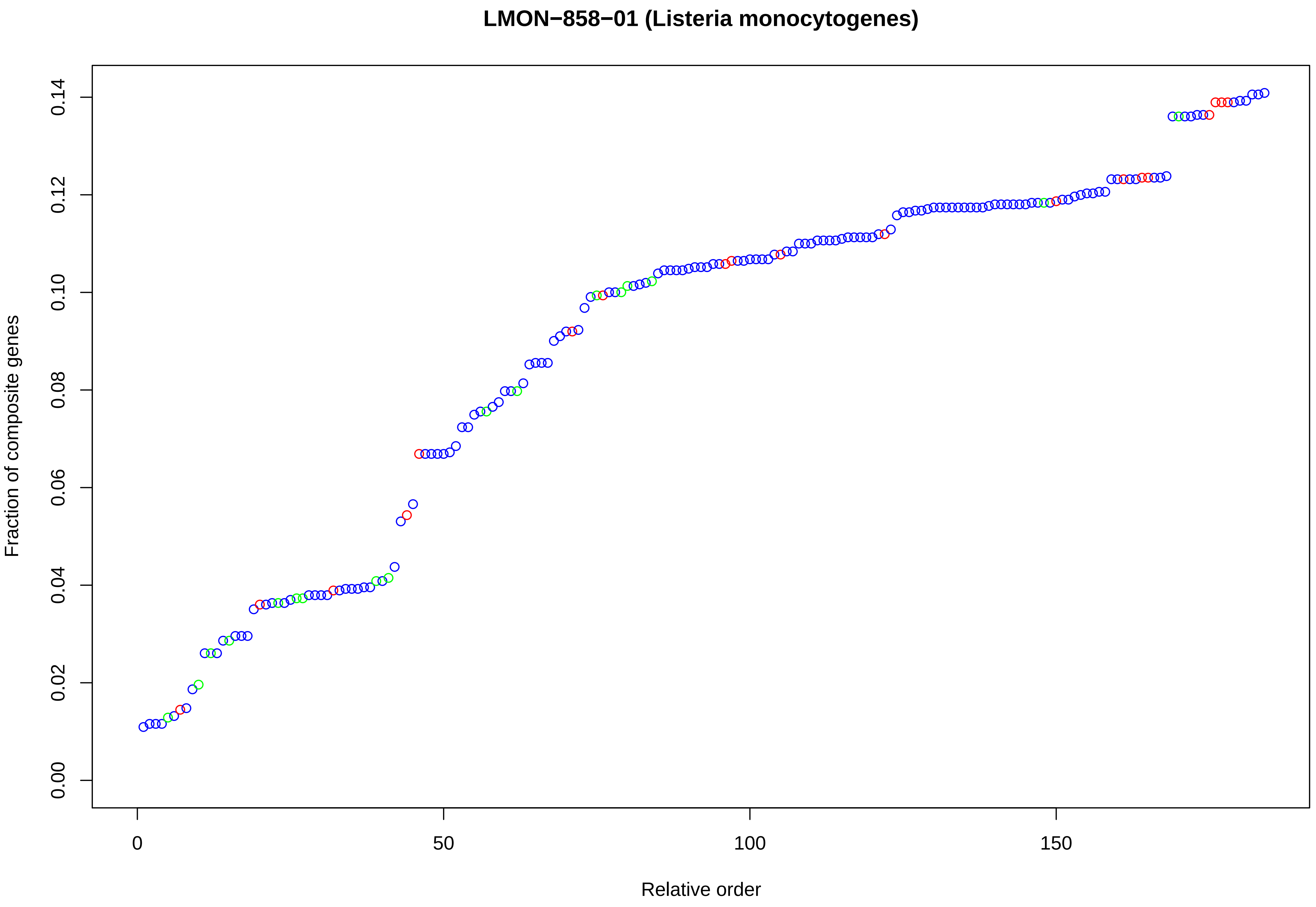
LMON−858−01 (Listeria monocytogenes)
Fraction of composite genes
0.00
0.02
0.04
0.06
0.08
0.10
0.12
0.14
Relative order
0
50
100
150

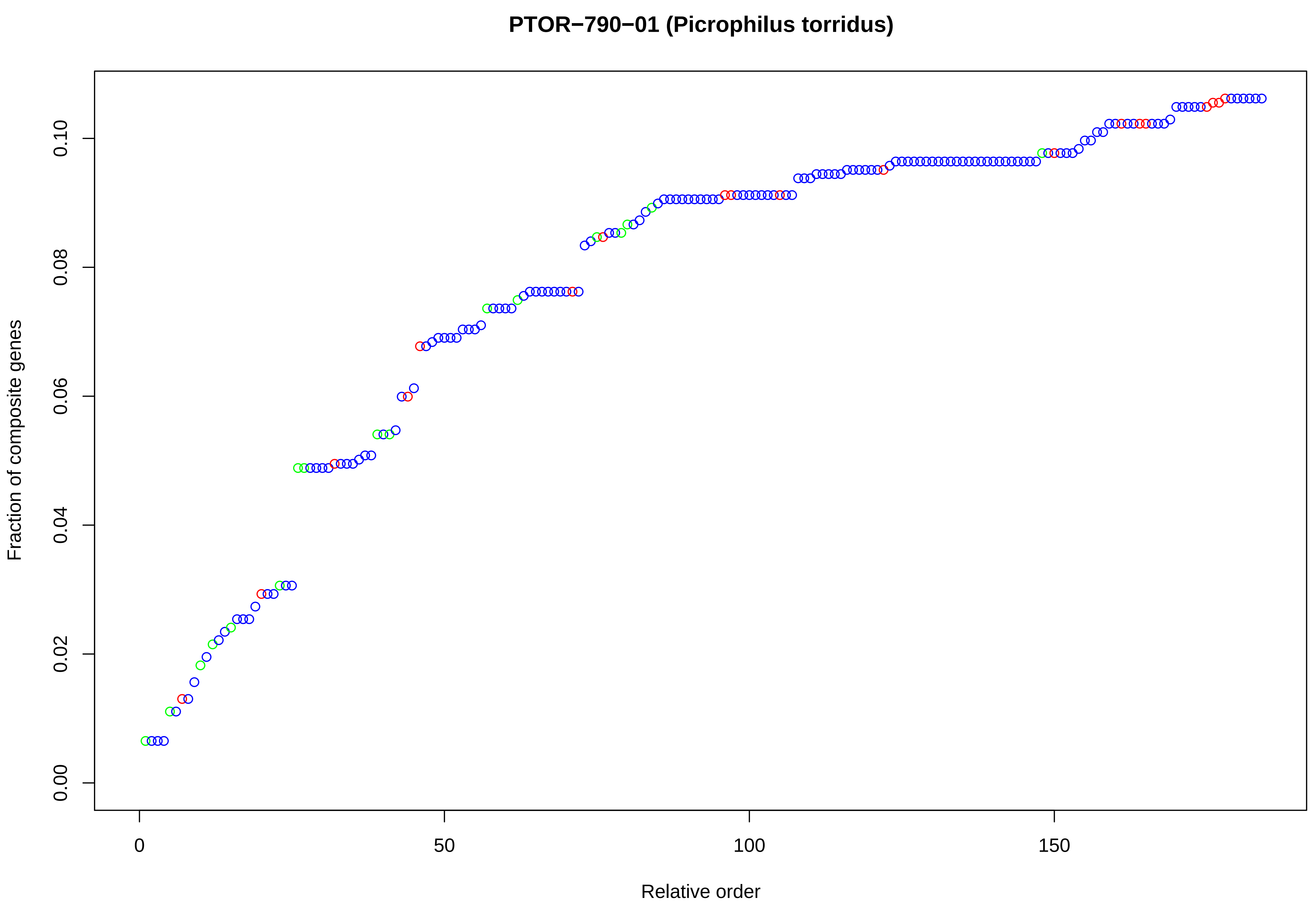

PTOR−790−01 (Picrophilus torridus)
Fraction of composite genes
Relative order
0.00
0.02
0.04
0.06
0.08
0.10
0
50
100
150

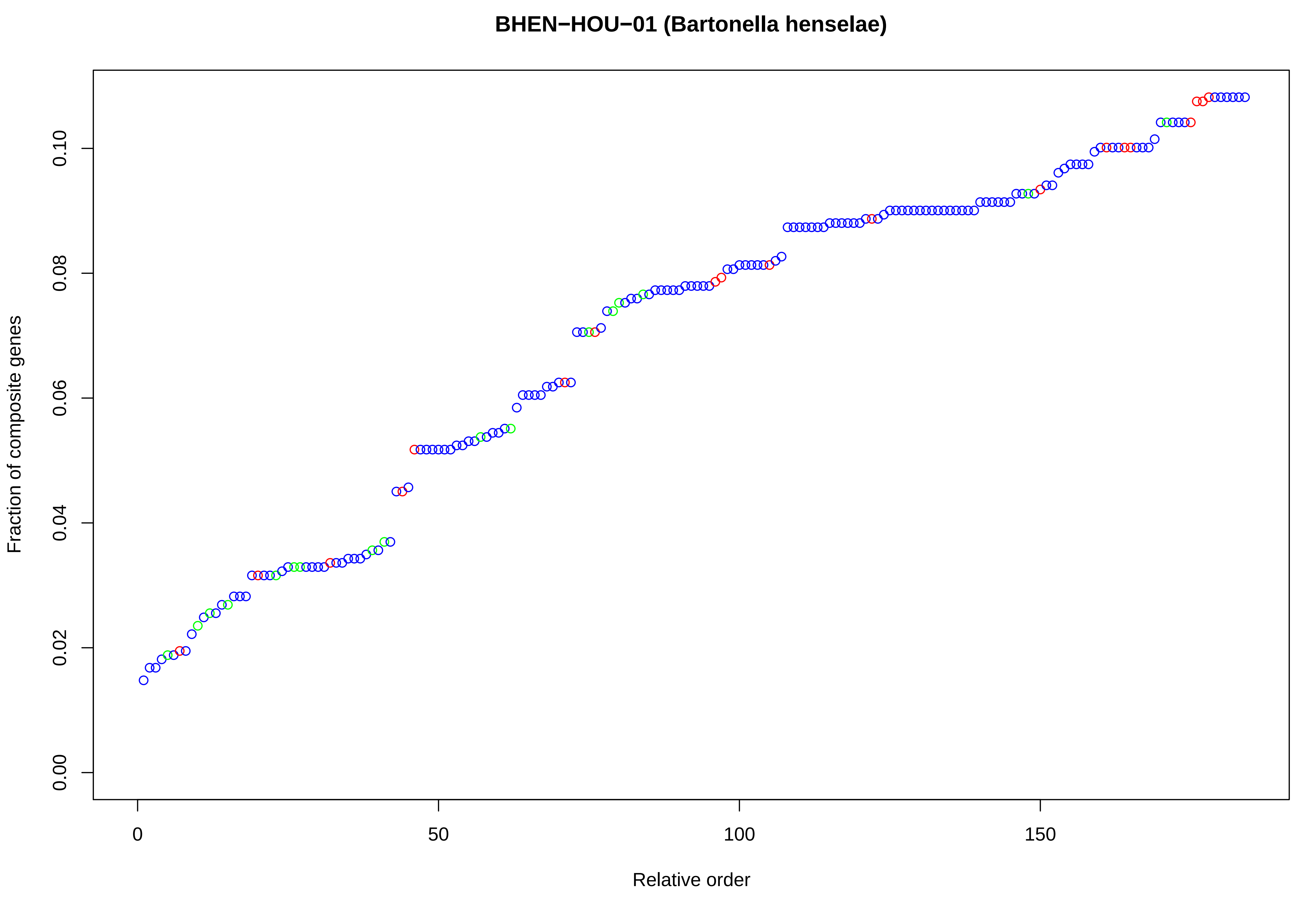

BHEN−HOU−01 (Bartonella henselae)
Fraction of composite genes
0.00
0.02
0.04
0.06
0.08
0.10
Relative order
0
50
100
150

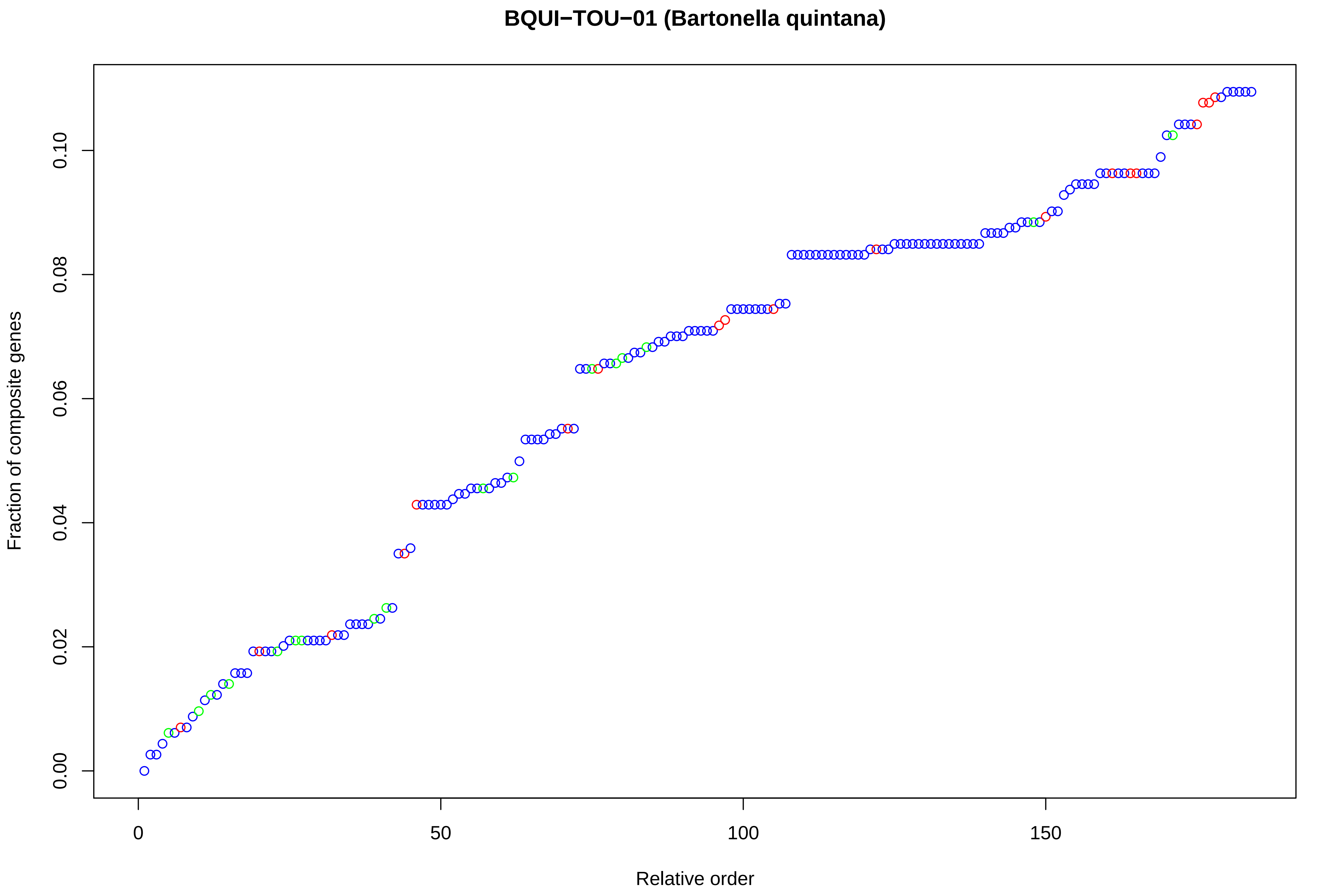
BQUI−TOU−01 (Bartonella quintana)
Fraction of composite genes
0.00
0.02
0.04
0.06
0.08
0.10
0
50
100
150
Relative order

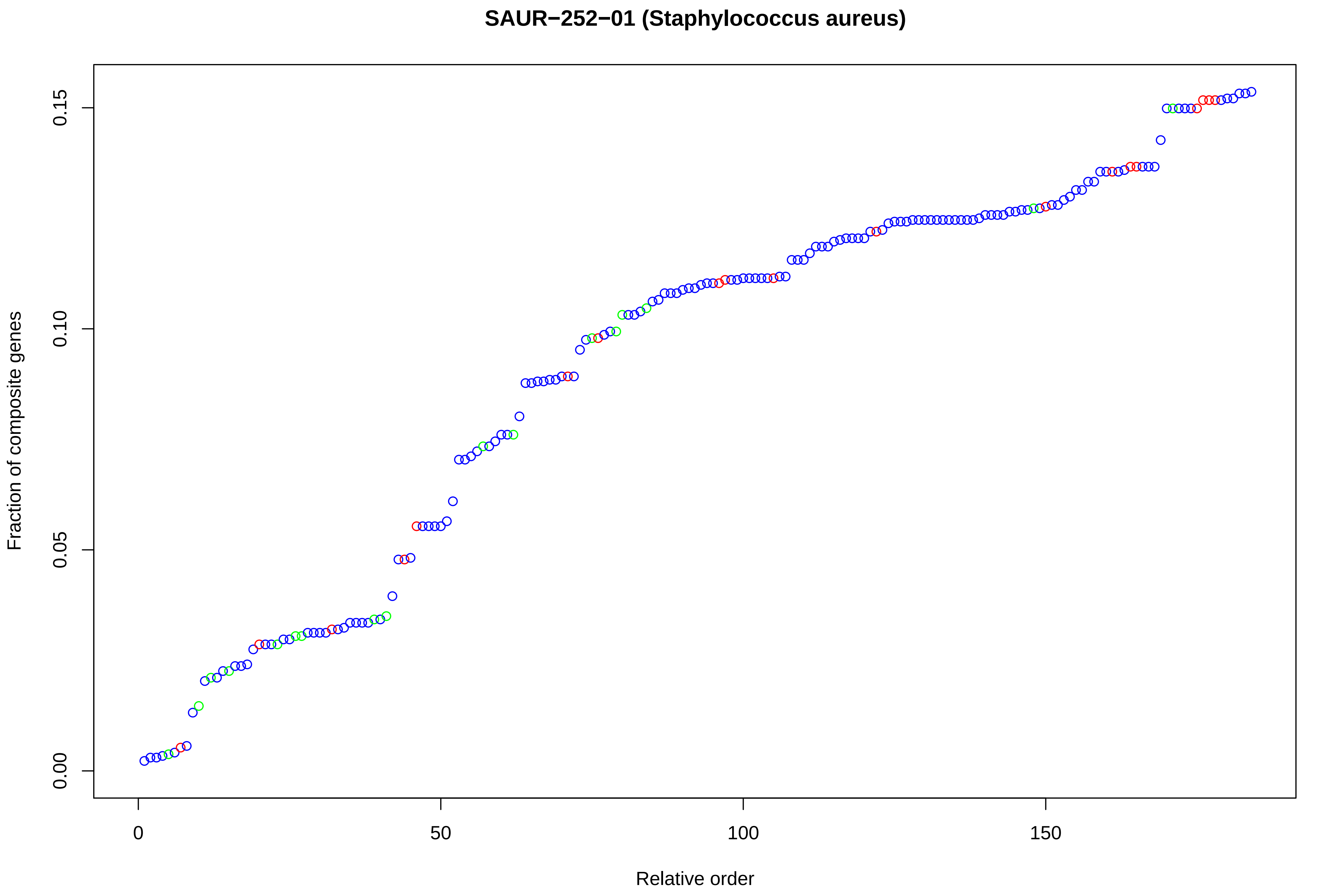

SAUR−252−01 (Staphylococcus aureus)
Fraction of composite genes
0.00
0.05
0.10
0.15
0
50
100
150
Relative order

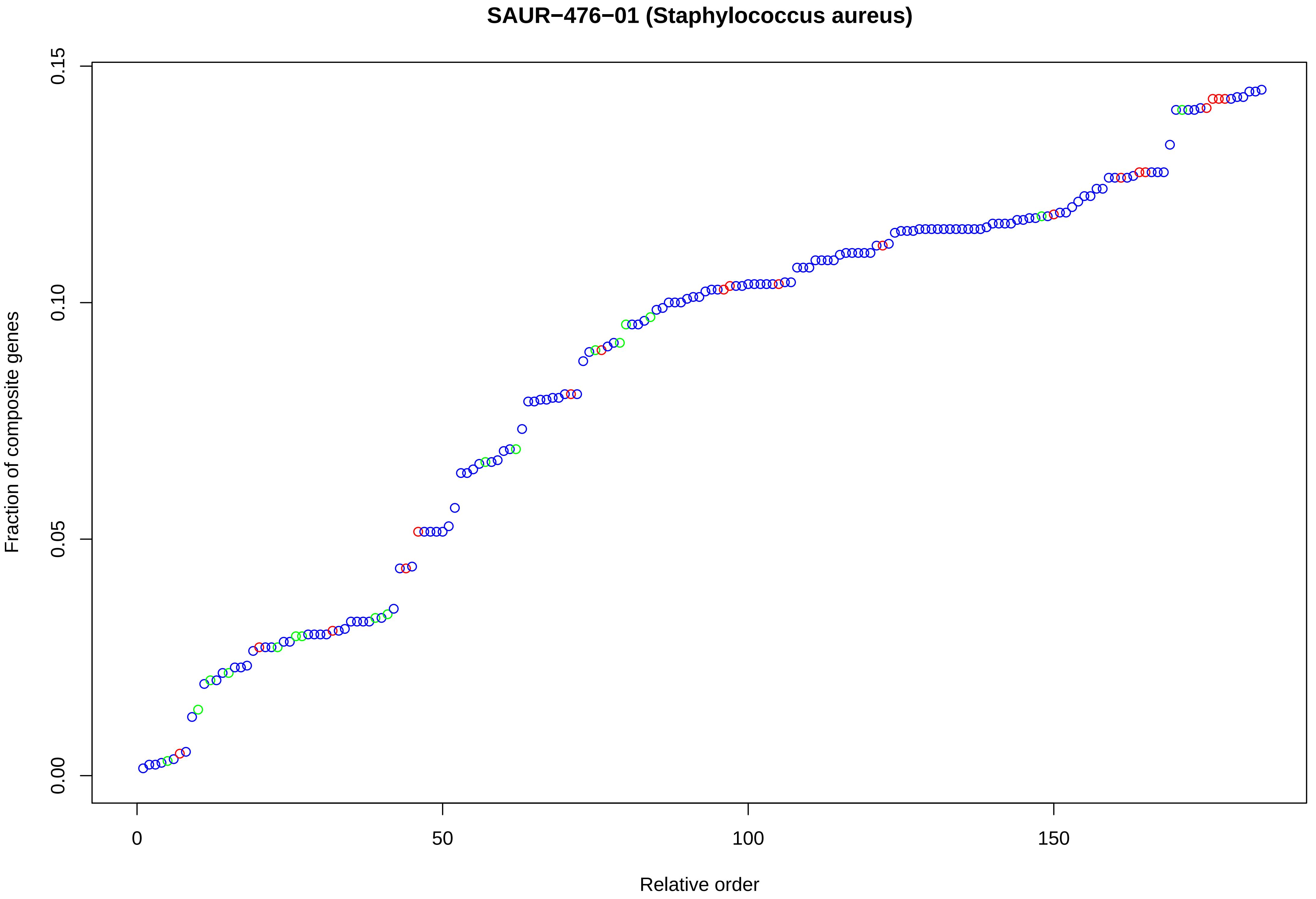
SAUR−476−01 (Staphylococcus aureus)
Fraction of composite genes
0.00
0.05
0.10
0.15
0
50
100
150
Relative order

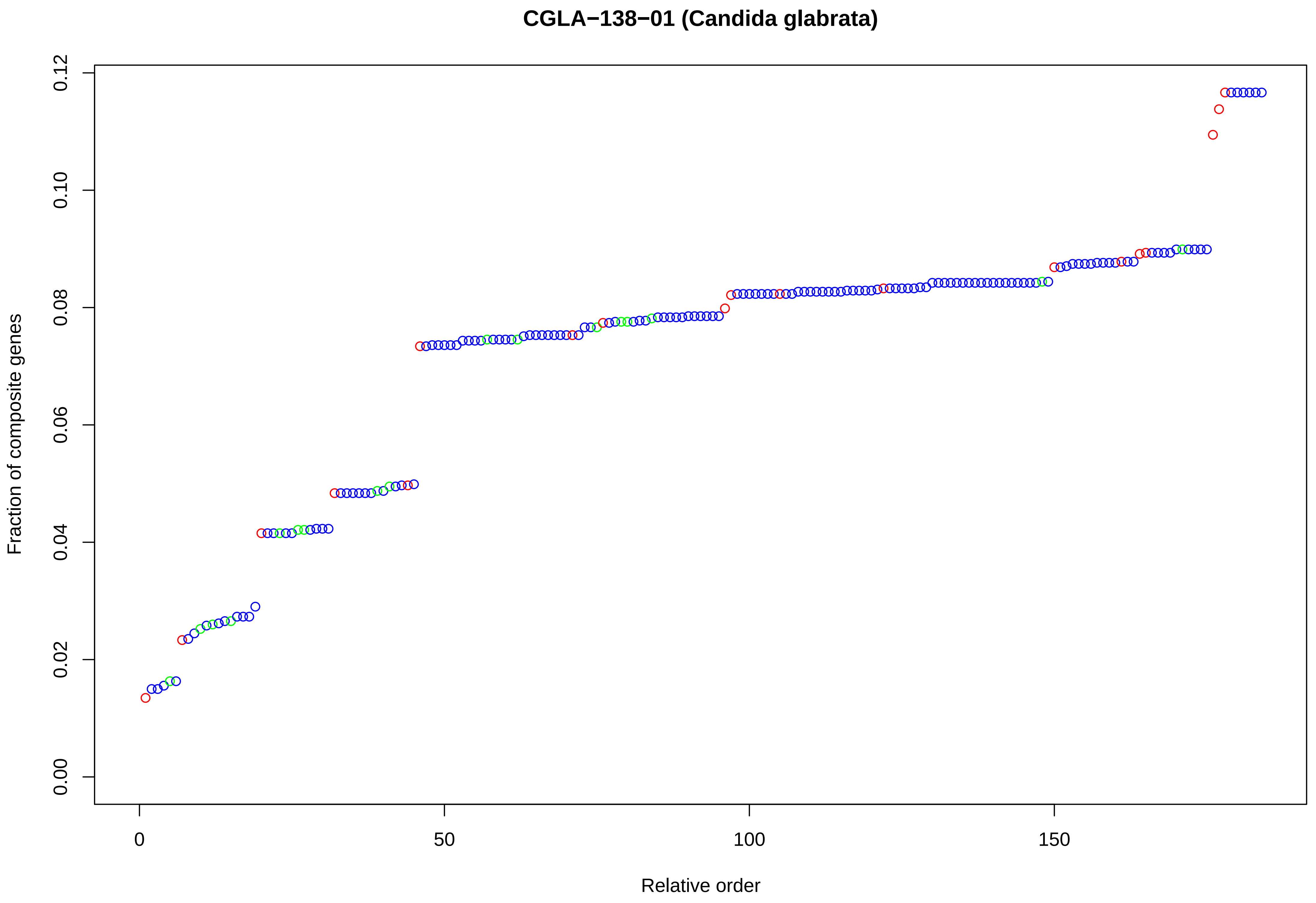

CGLA−138−01 (Candida glabrata)
Fraction of composite genes
0.00
0.02
0.04
0.06
0.08
0.10
0.12
Relative order
0
50
100
150

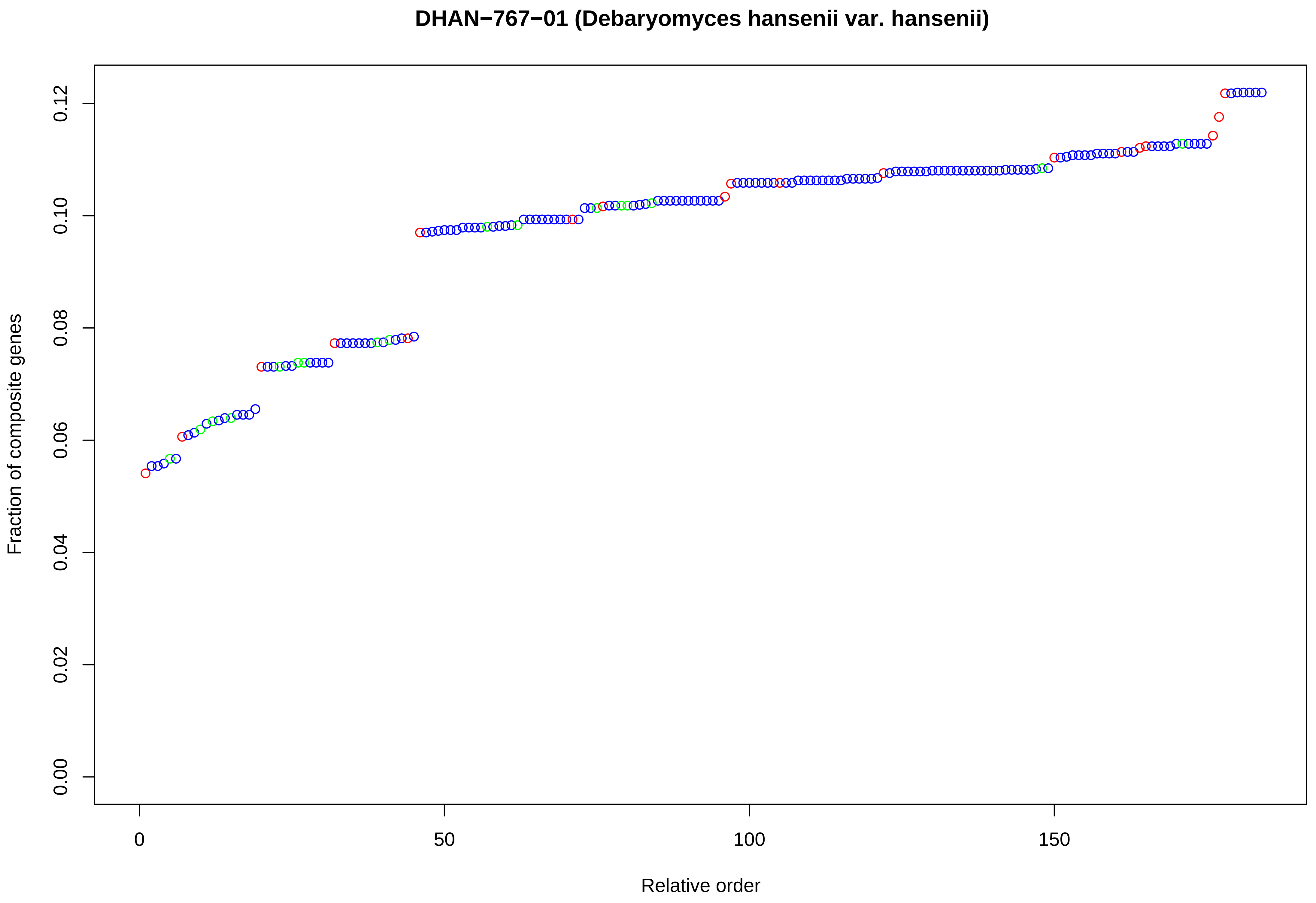

DHAN−767−01 (Debaryomyces hansenii var. hansenii)
Fraction of composite genes
0.00
0.02
0.04
0.06
0.08
0.10
0.12
0
50
100
150
Relative order

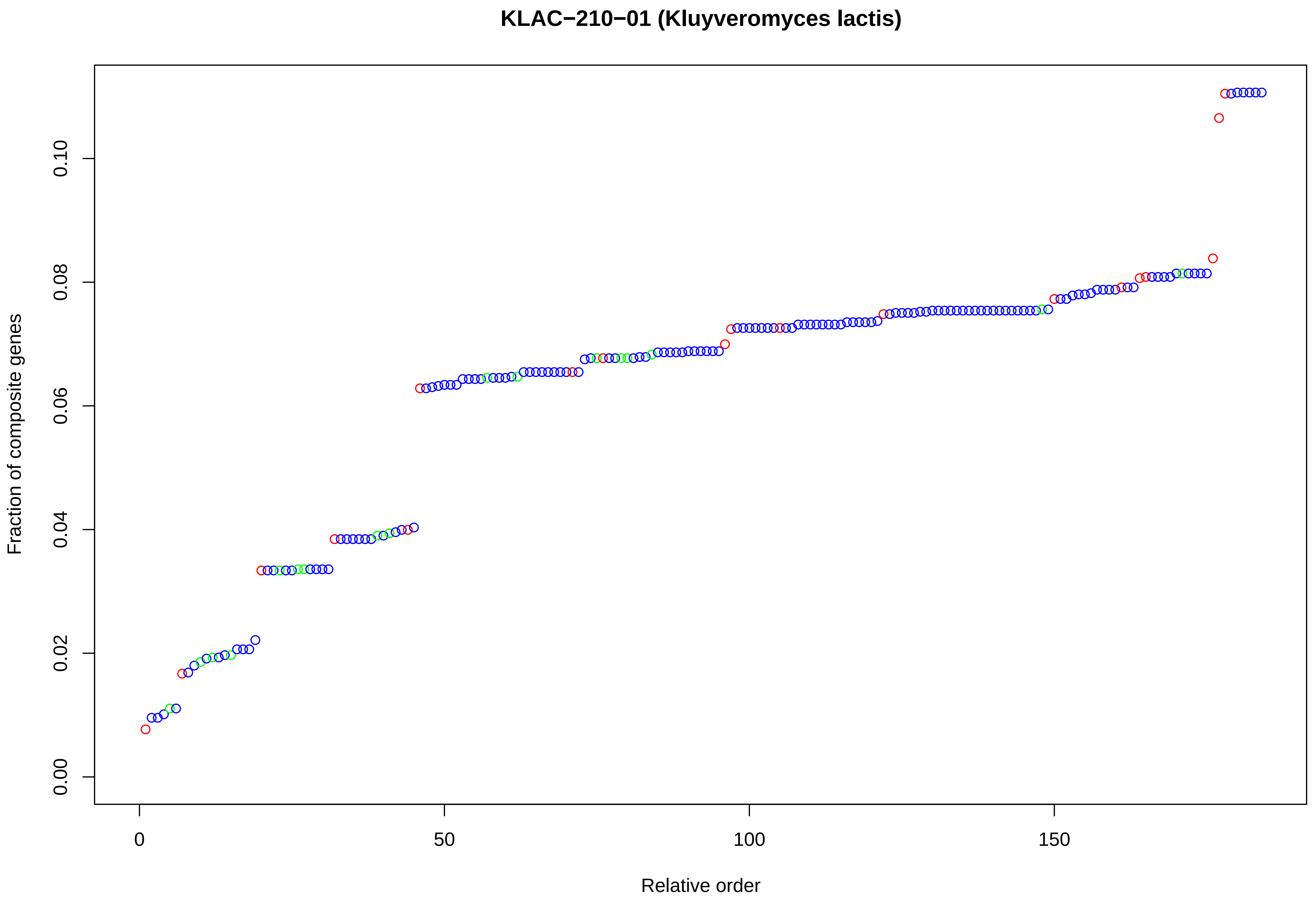
KLAC−210−01 (Kluyveromyces lactis)
Fraction of composite genes
0.00
0.02
0.04
0.06
0.08
0.10
0
50
100
150
Relative order

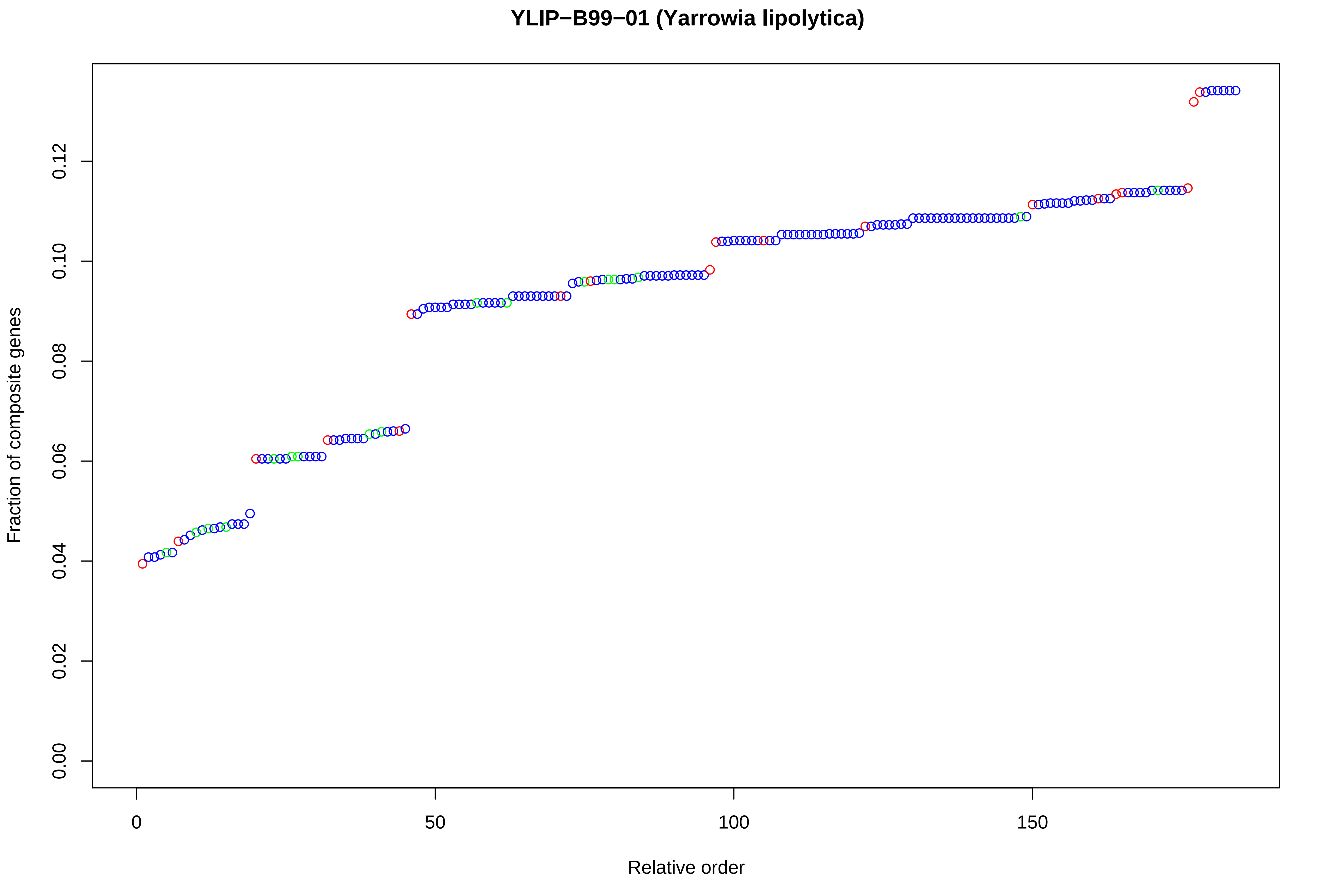

YLIP−B99−01 (Yarrowia lipolytica)
Fraction of composite genes
0.00
0.02
0.04
0.06
0.08
0.10
0.12
0
50
100
150
Relative order

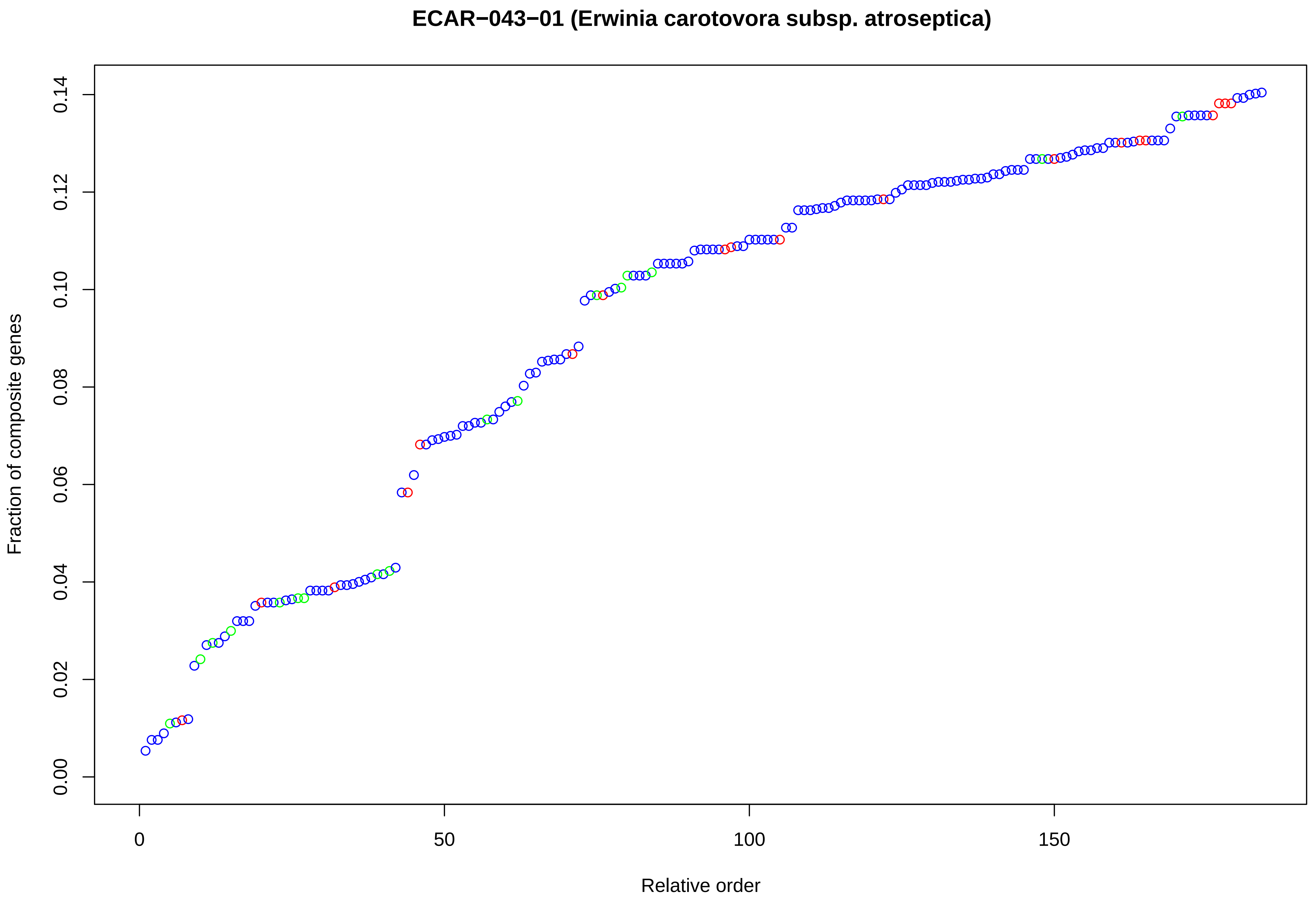
ECAR−043−01 (Erwinia carotovora subsp. atroseptica)
Fraction of composite genes
0.00
0.02
0.04
0.06
0.08
0.10
0.12
0.14
Relative order
0
50
100
150

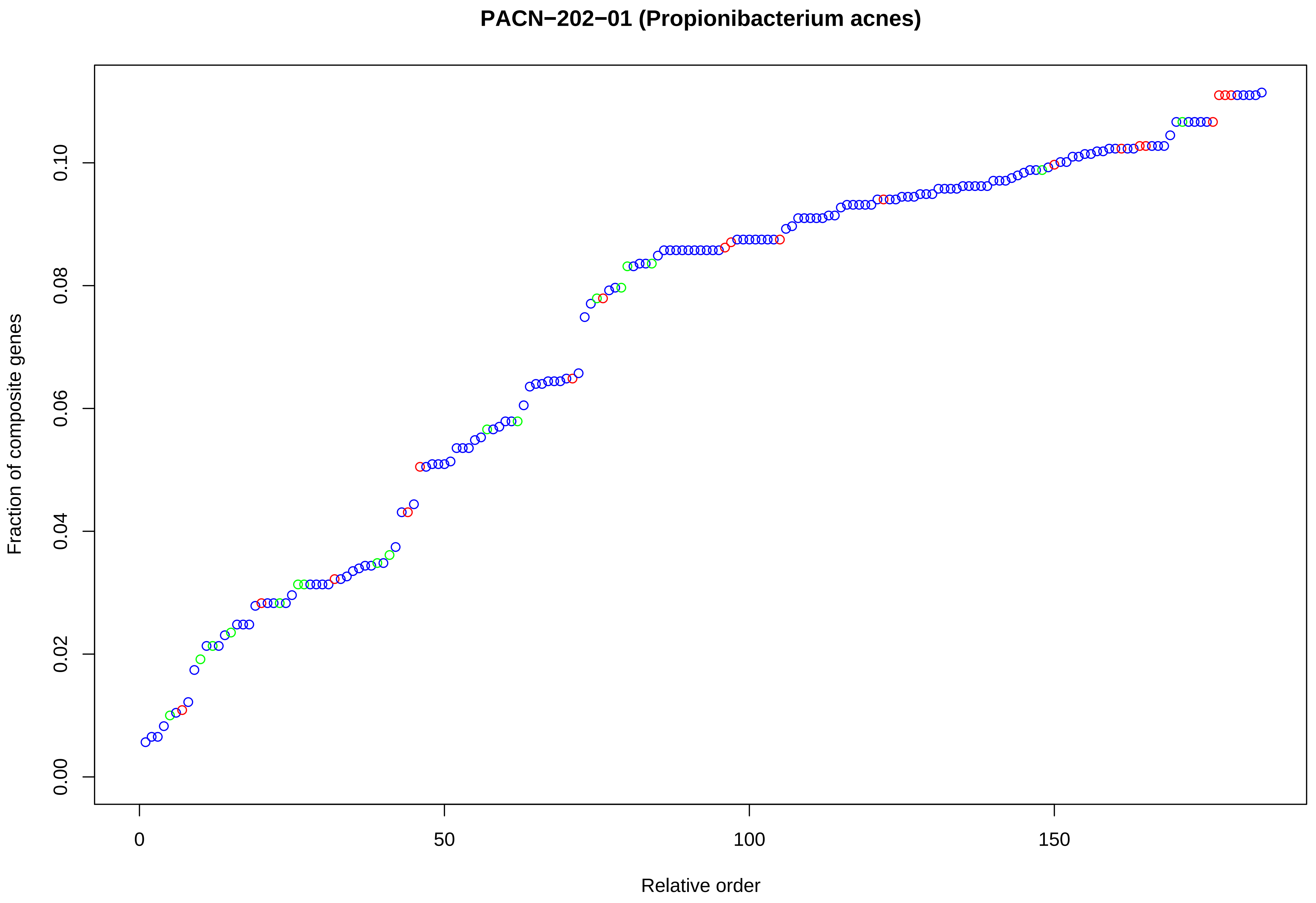
PACN−202−01 (Propionibacterium acnes)
Fraction of composite genes
0.00
0.02
0.04
0.06
0.08
0.10
Relative order
0
50
100
150

MMOB−63K−01 (Mycoplasma mobile)

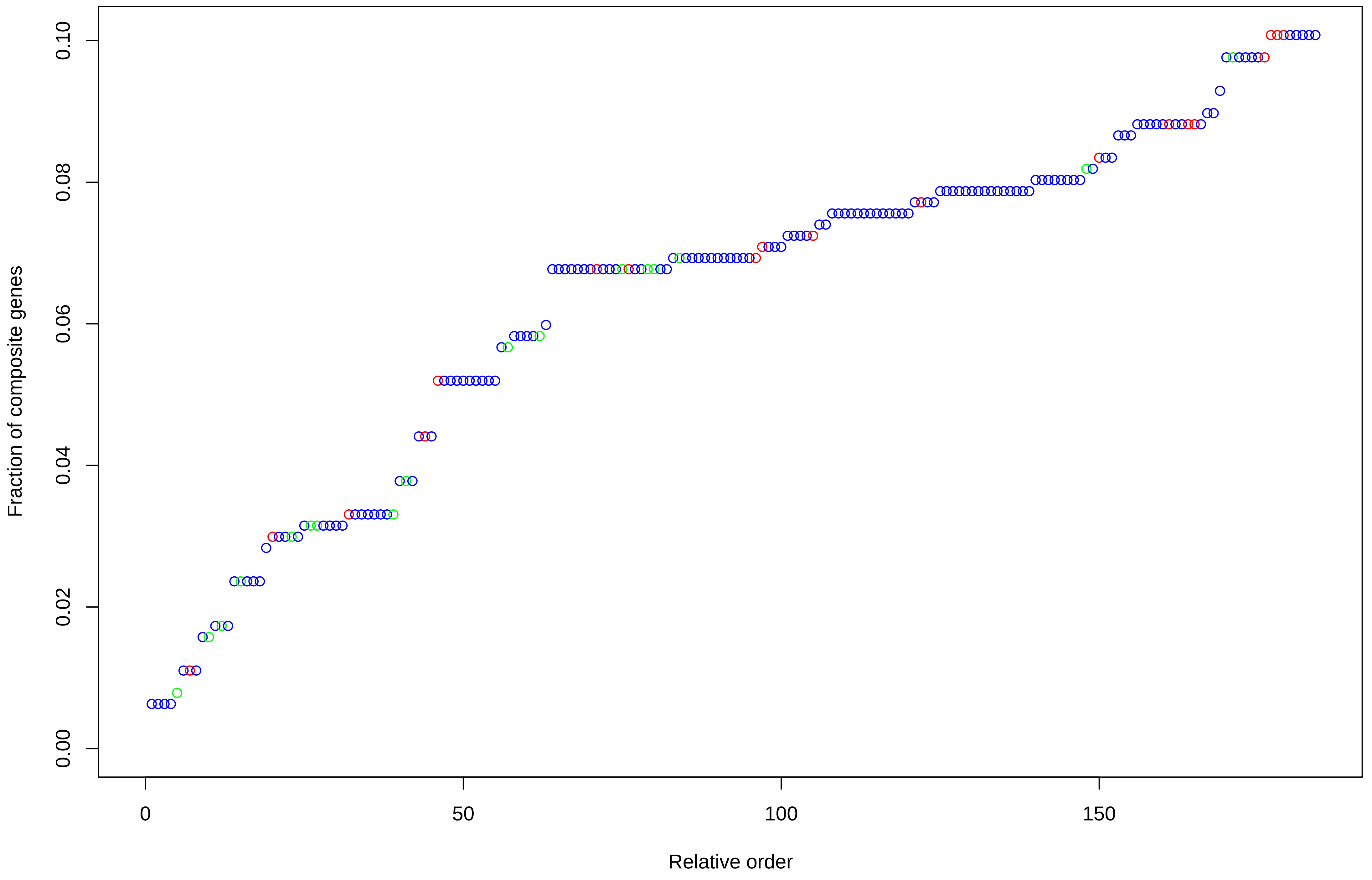

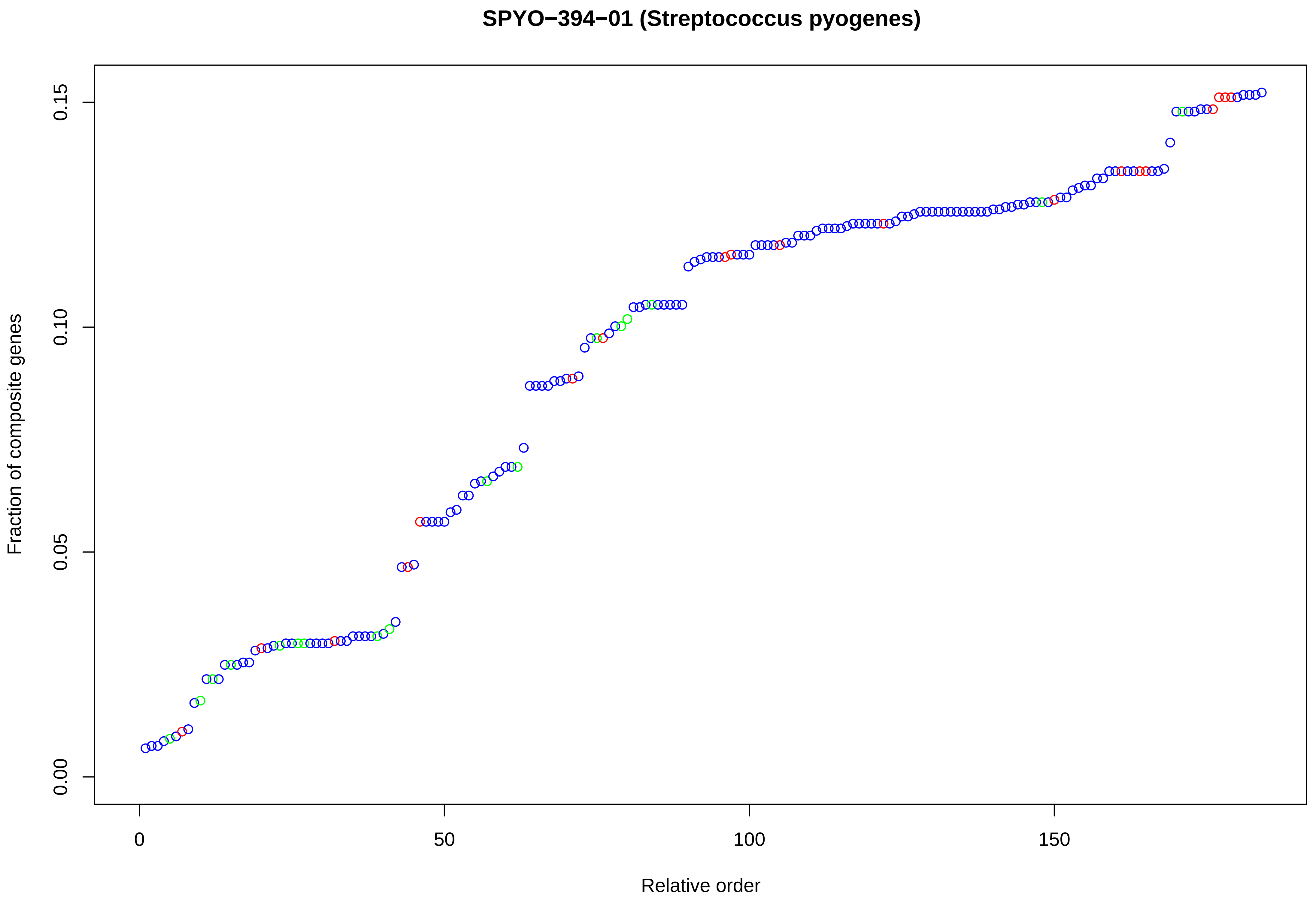
SPYO−394−01 (Streptococcus pyogenes)
Fraction of composite genes
Relative order
0.00
0.05
0.10
0.15
0
50
100
150

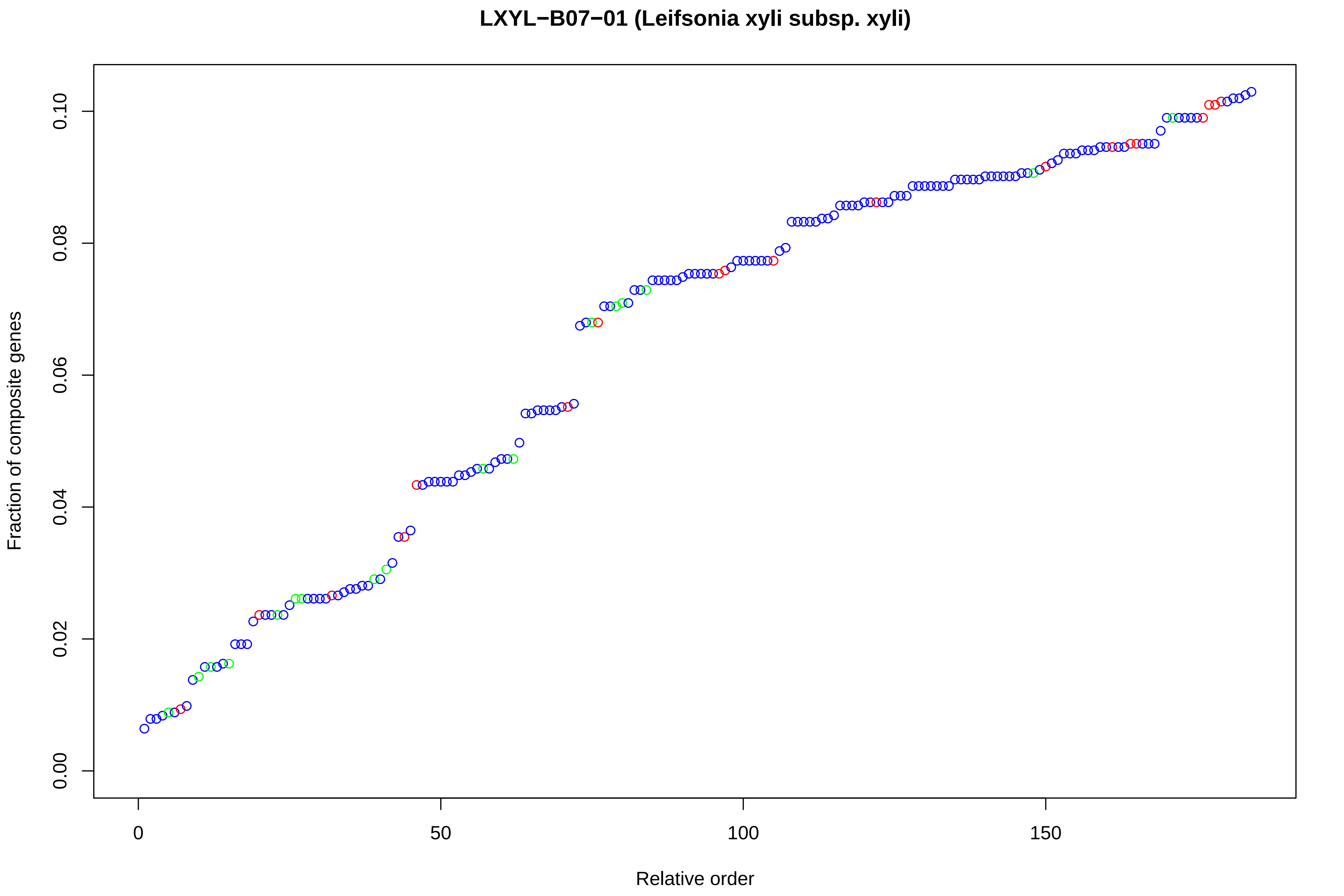
LXYL−B07−01 (Leifsonia xyli subsp. xyli)
Fraction of composite genes
0.00
0.02
0.04
0.06
0.08
0.10
0
50
100
150
Relative order

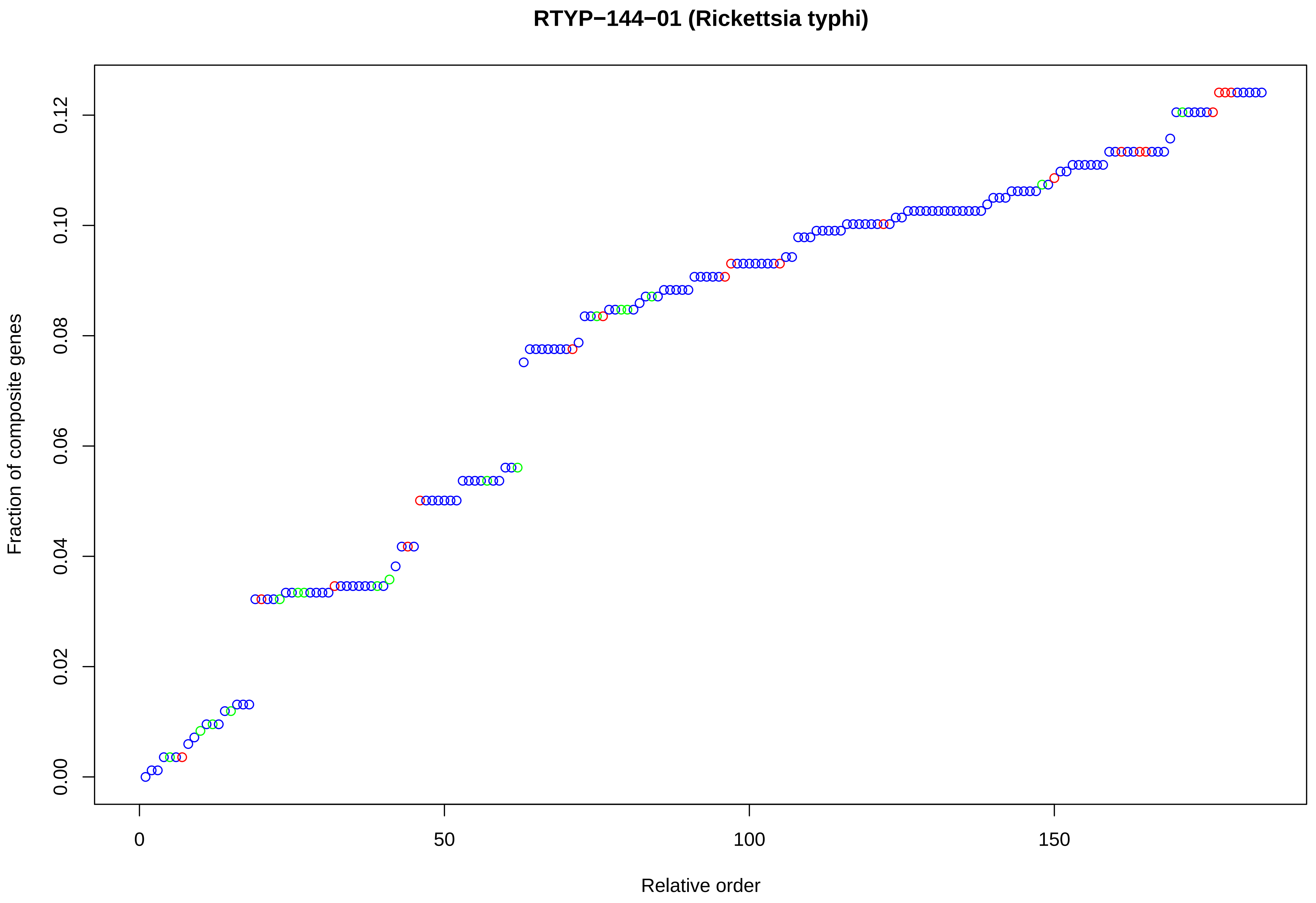
RTYP−144−01 (Rickettsia typhi)
Fraction of composite genes
0.00
0.02
0.04
0.06
0.08
0.10
0.12
0
50
100
150
Relative order
