# Supplementary material for: Denoising inferred functional association networks obtained by gene fusion analysis
Source: BMC Genomics. 2007 Dec 14;8:460. doi: 10.1186/1471-2164-8-460 (PMC2248599; doi:10.1186/1471-2164-8-460)
Supplement: Additional File 5 — Distribution of interactions across 184 genomes. [file 1471-2164-8-460-S5.pdf]

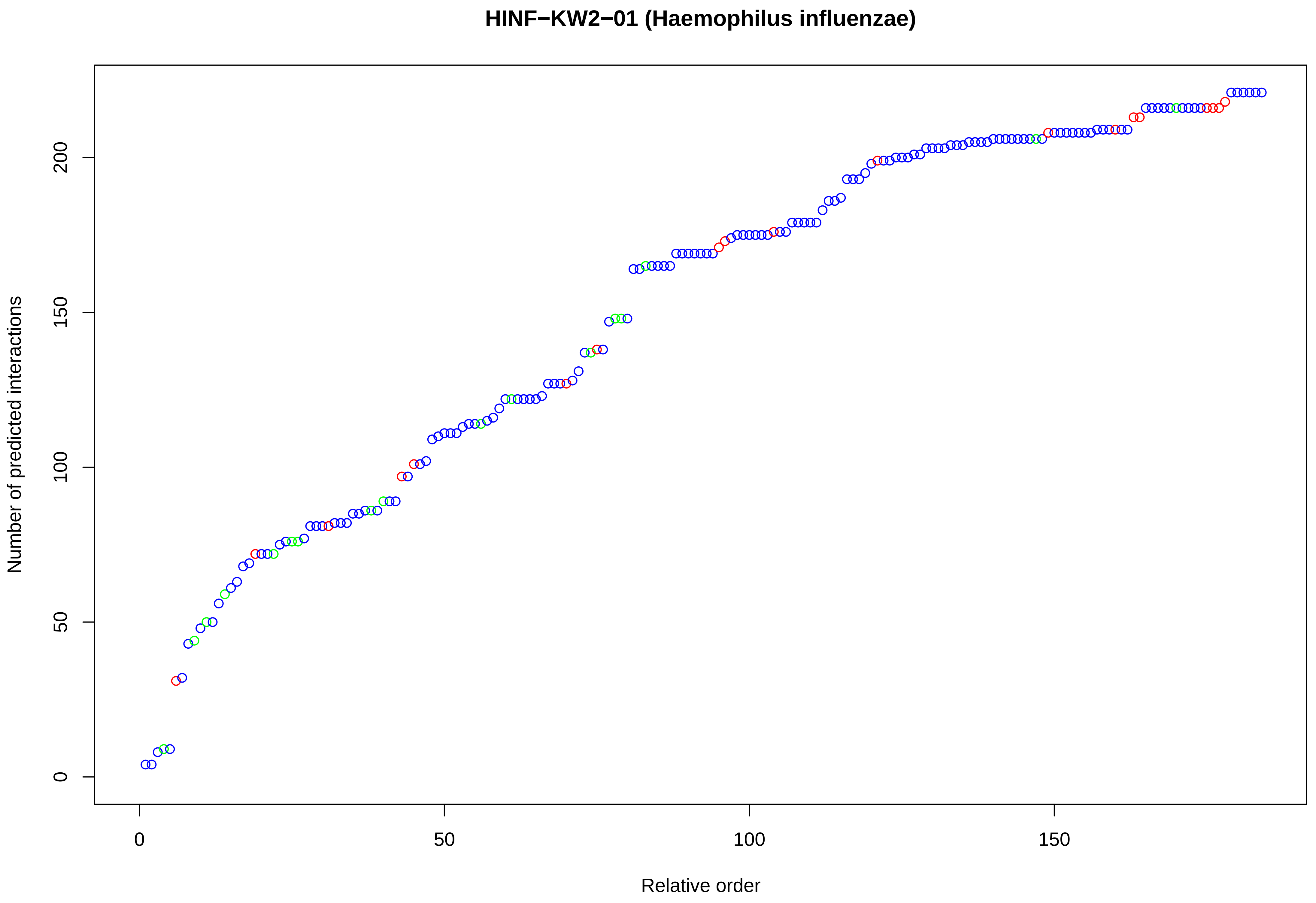

HINF−KW2−01 (Haemophilus influenzae)
Number of predicted interactions
0
50
100
150
200
0
50
100
150
Relative order

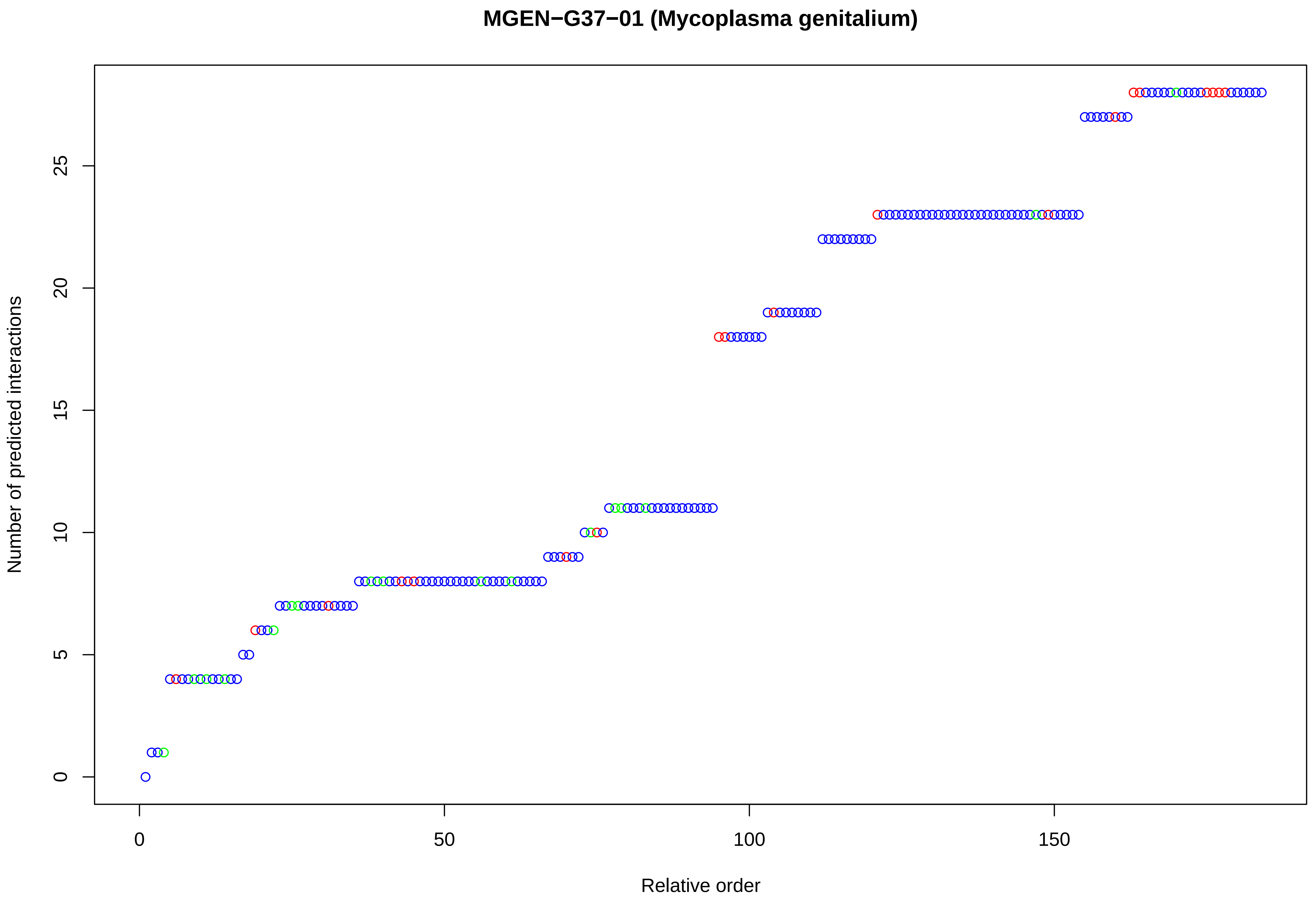
MGEN−G37−01 (Mycoplasma genitalium)
Number of predicted interactions
Relative order
0
5
10
15
20
25
0
50
100
150

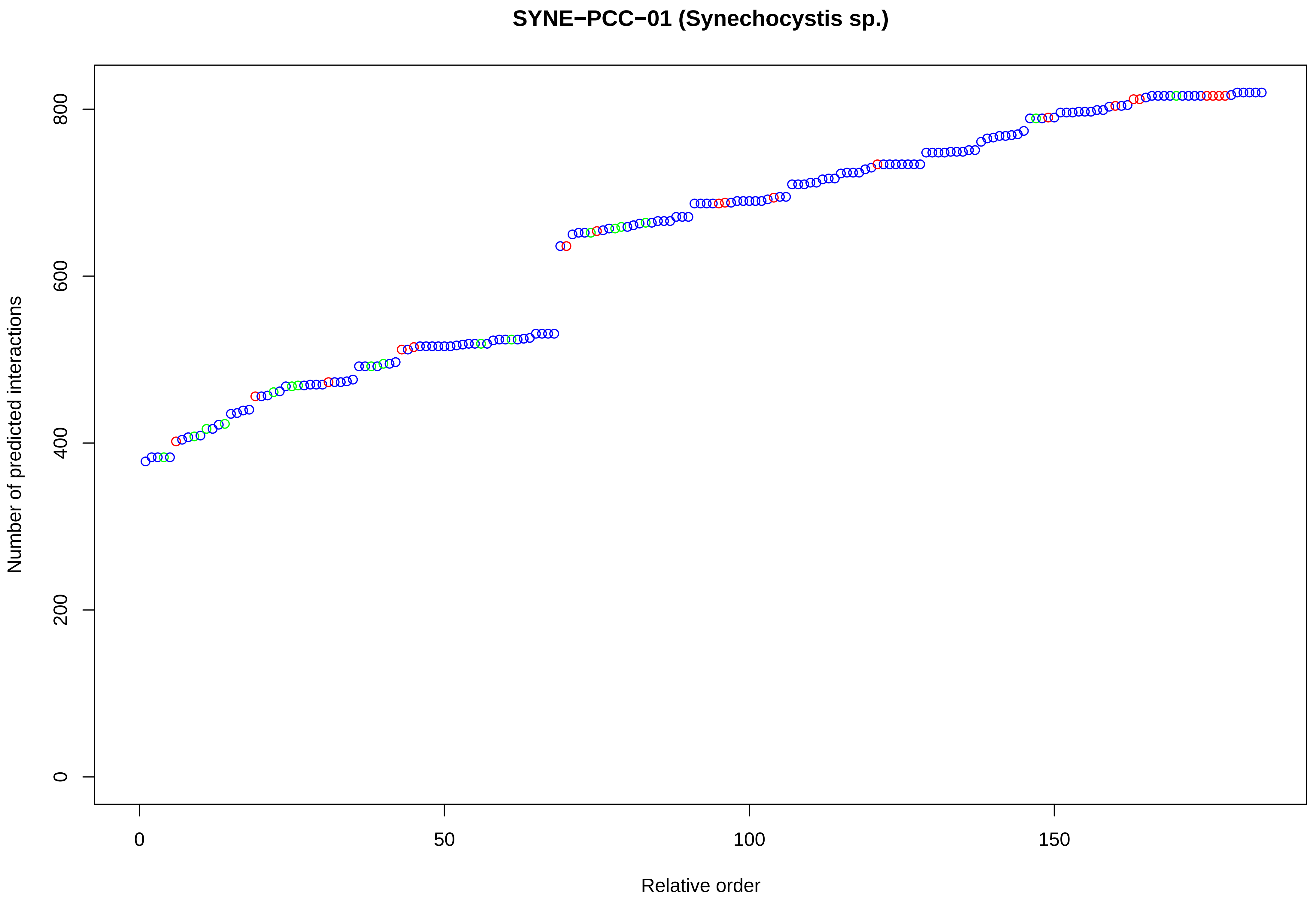
SYNE−PCC−01 (Synechocystis sp.)
Number of predicted interactions
Relative order
0
200
400
600
800
0
50
100
150

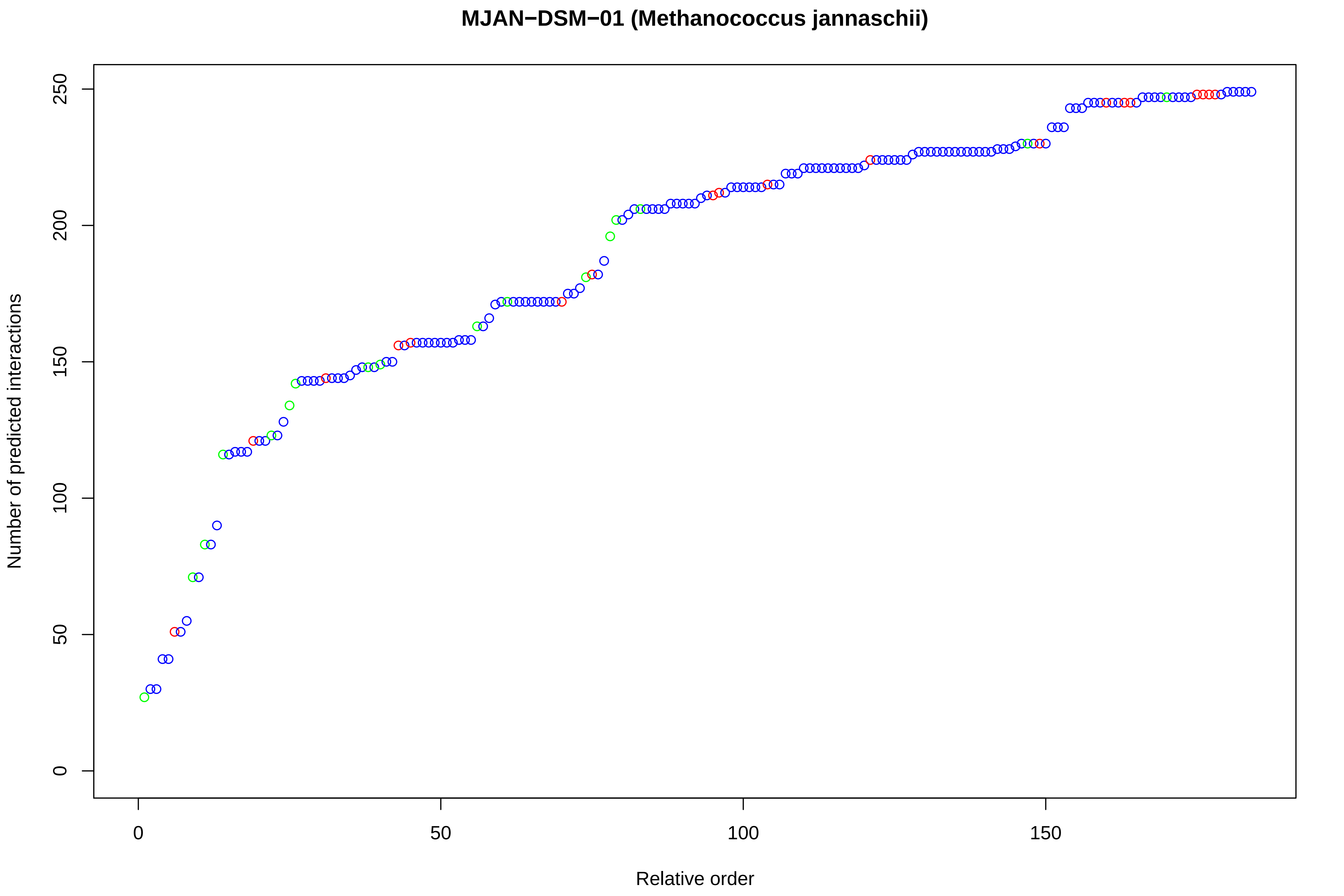
MJAN−DSM−01 (Methanococcus jannaschii)
Number of predicted interactions
Relative order
0
50
100
150
200
250
0
50
100
150

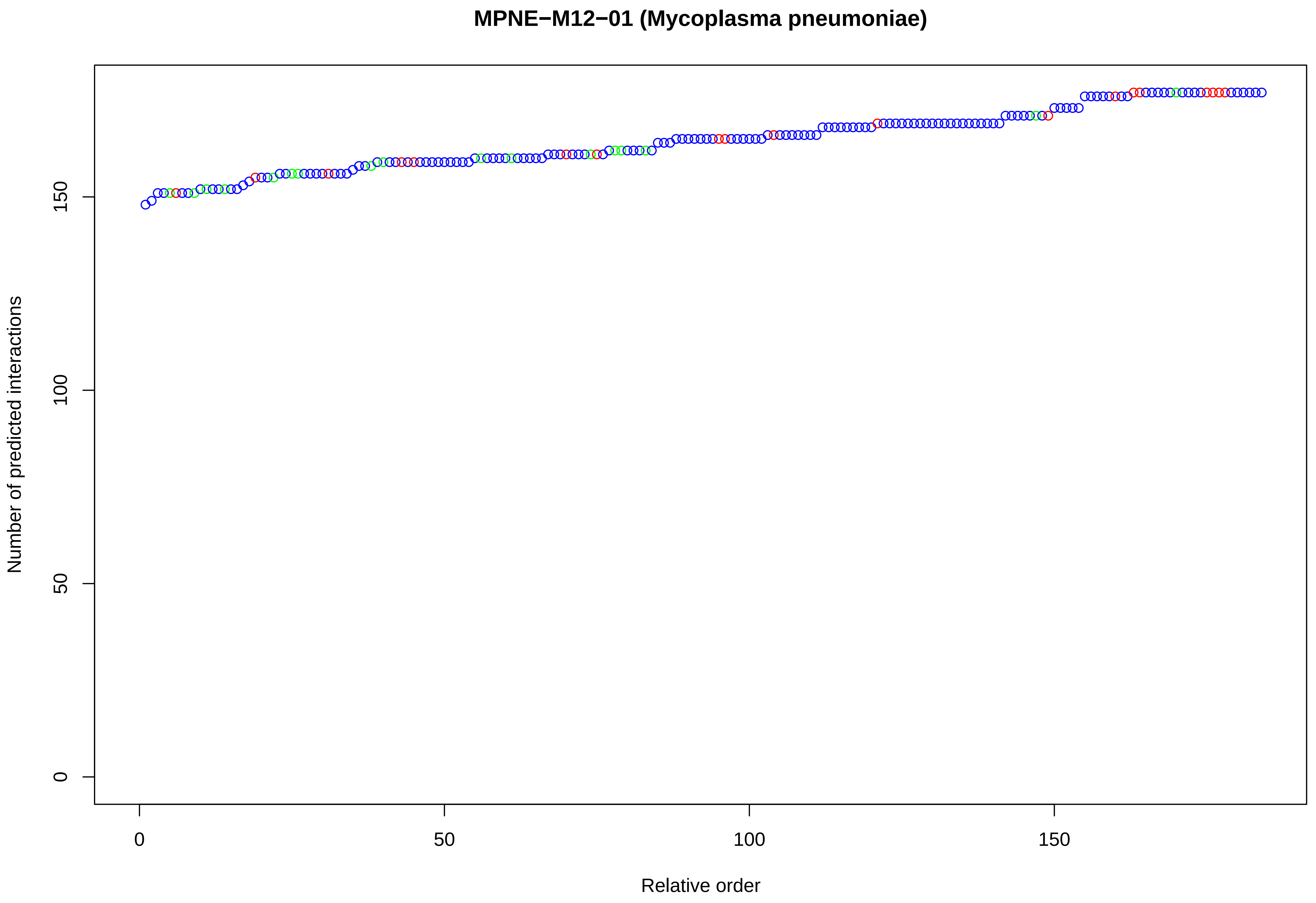
MPNE−M12−01 (Mycoplasma pneumoniae)
Number of predicted interactions
Relative order
0
50
100
150
0
50
100
150

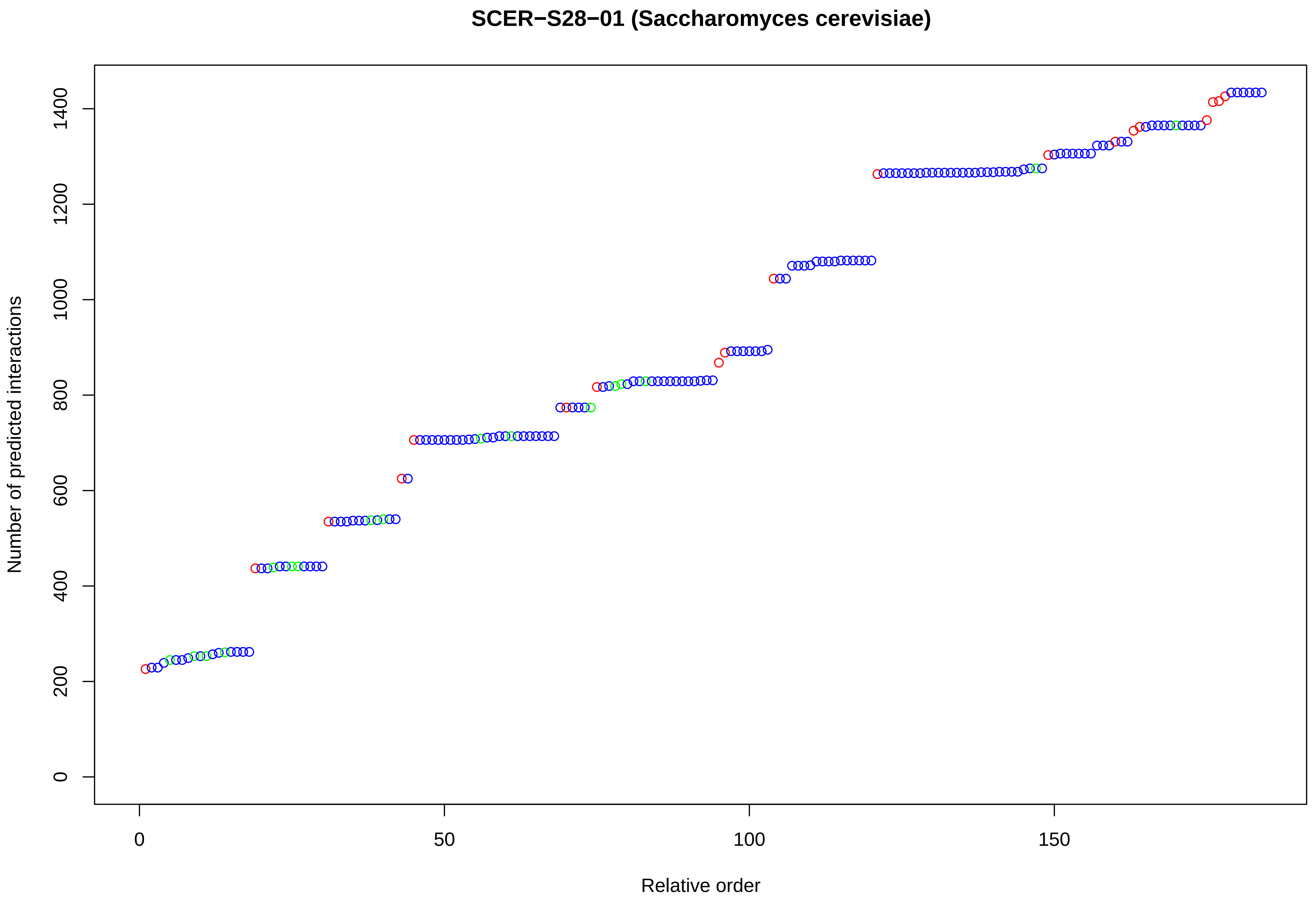

SCER−S28−01 (Saccharomyces cerevisiae)
Number of predicted interactions
Relative order
0
200
400
600
800
1000
1200
1400
0
50
100
150

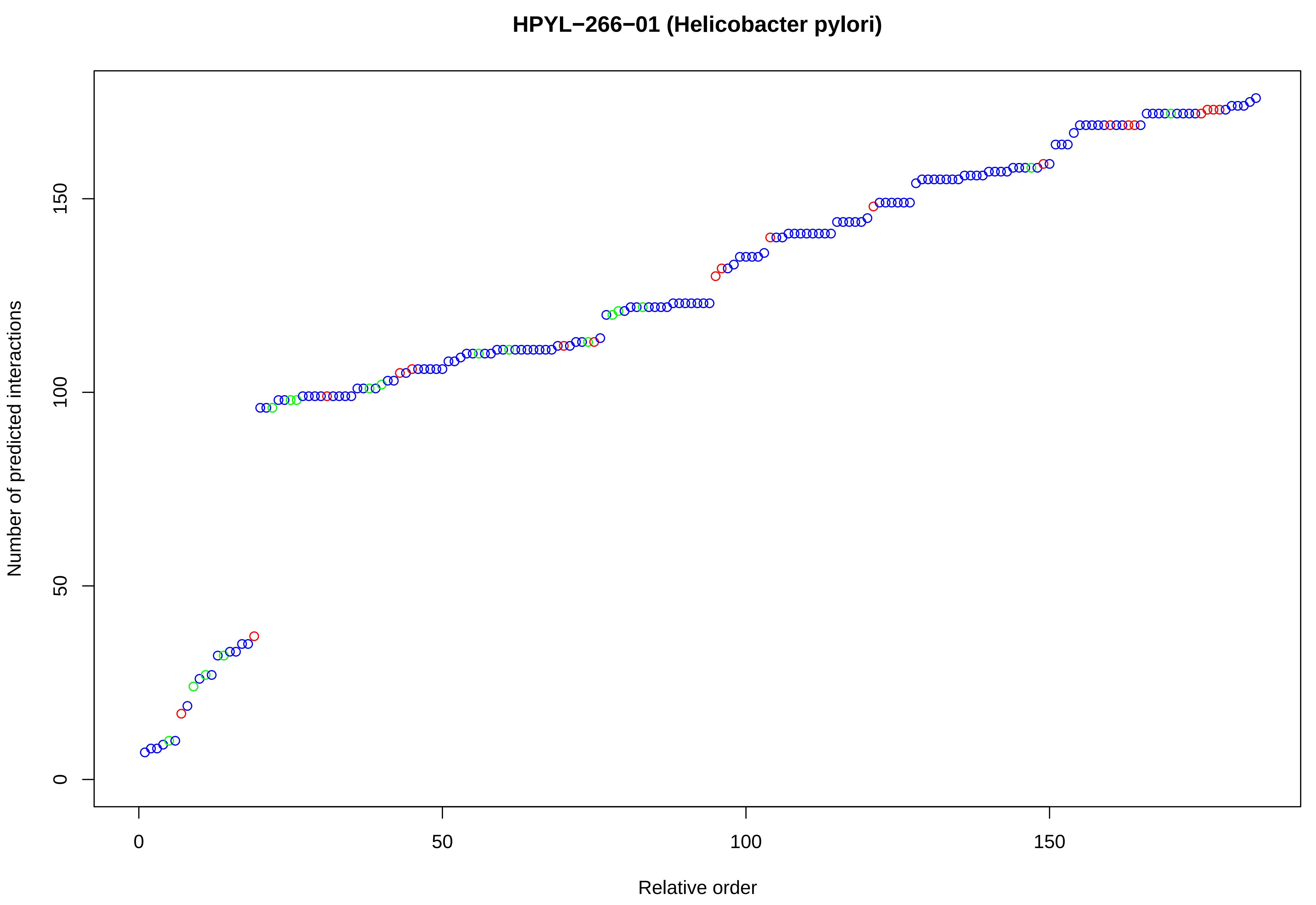

HPYL−266−01 (Helicobacter pylori)
Number of predicted interactions
0
50
100
150
Relative order
0
50
100
150

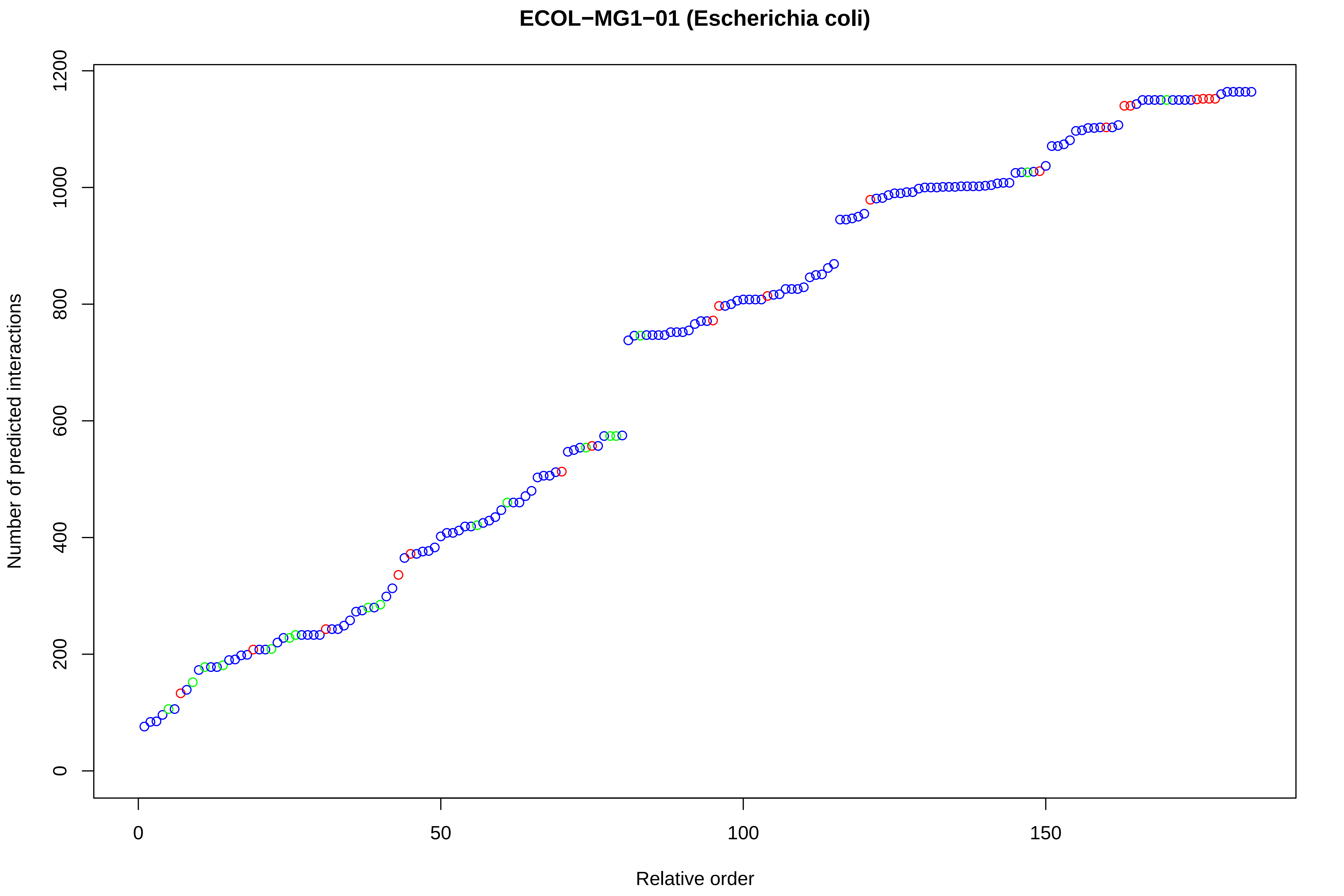
ECOL−MG1−01 (Escherichia coli)
Number of predicted interactions
0
200
400
600
800
1000
1200
Relative order
0
50
100
150

MTHE−DEL−01 (Methanobacterium thermoautotrophicum)

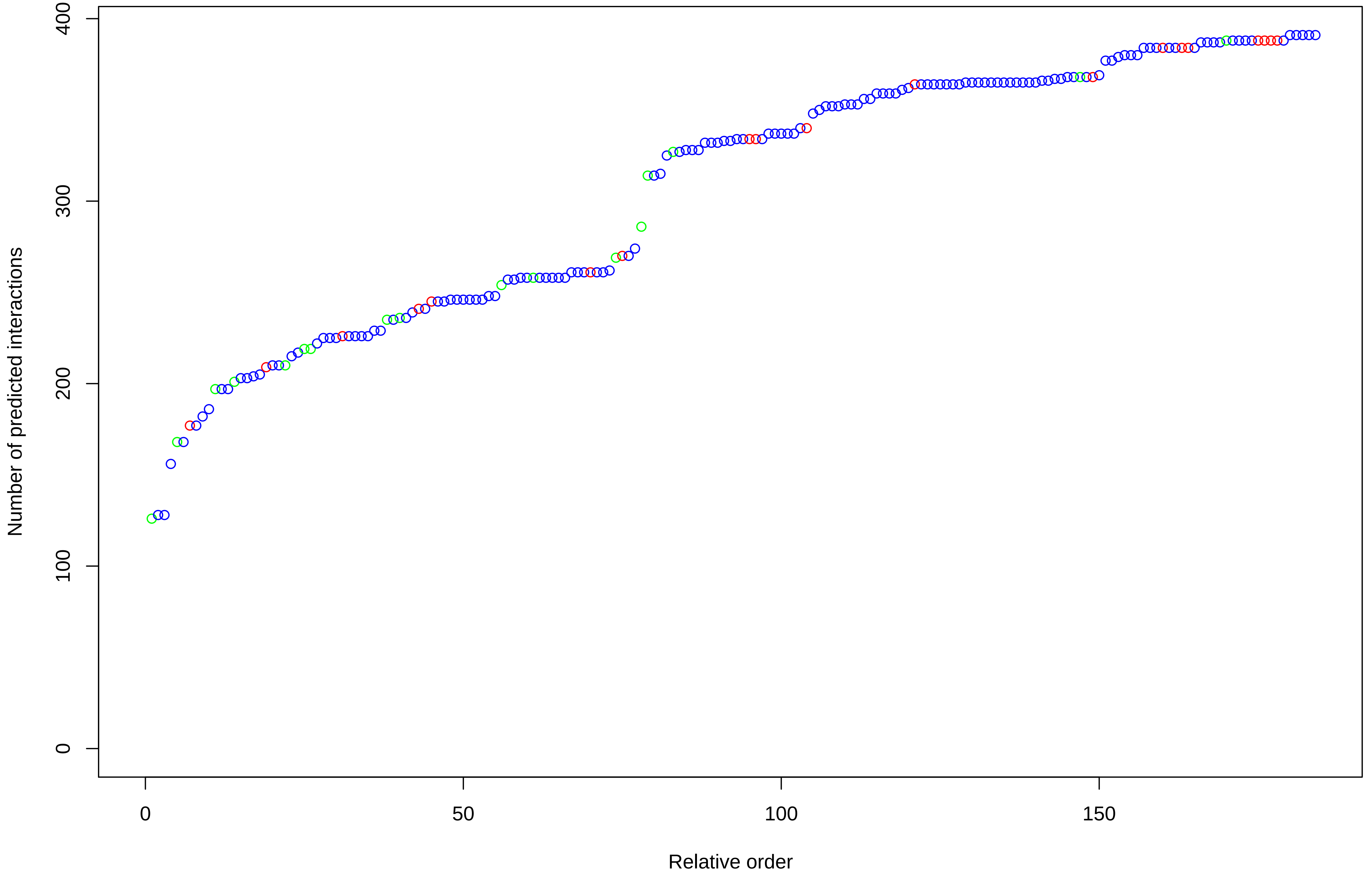

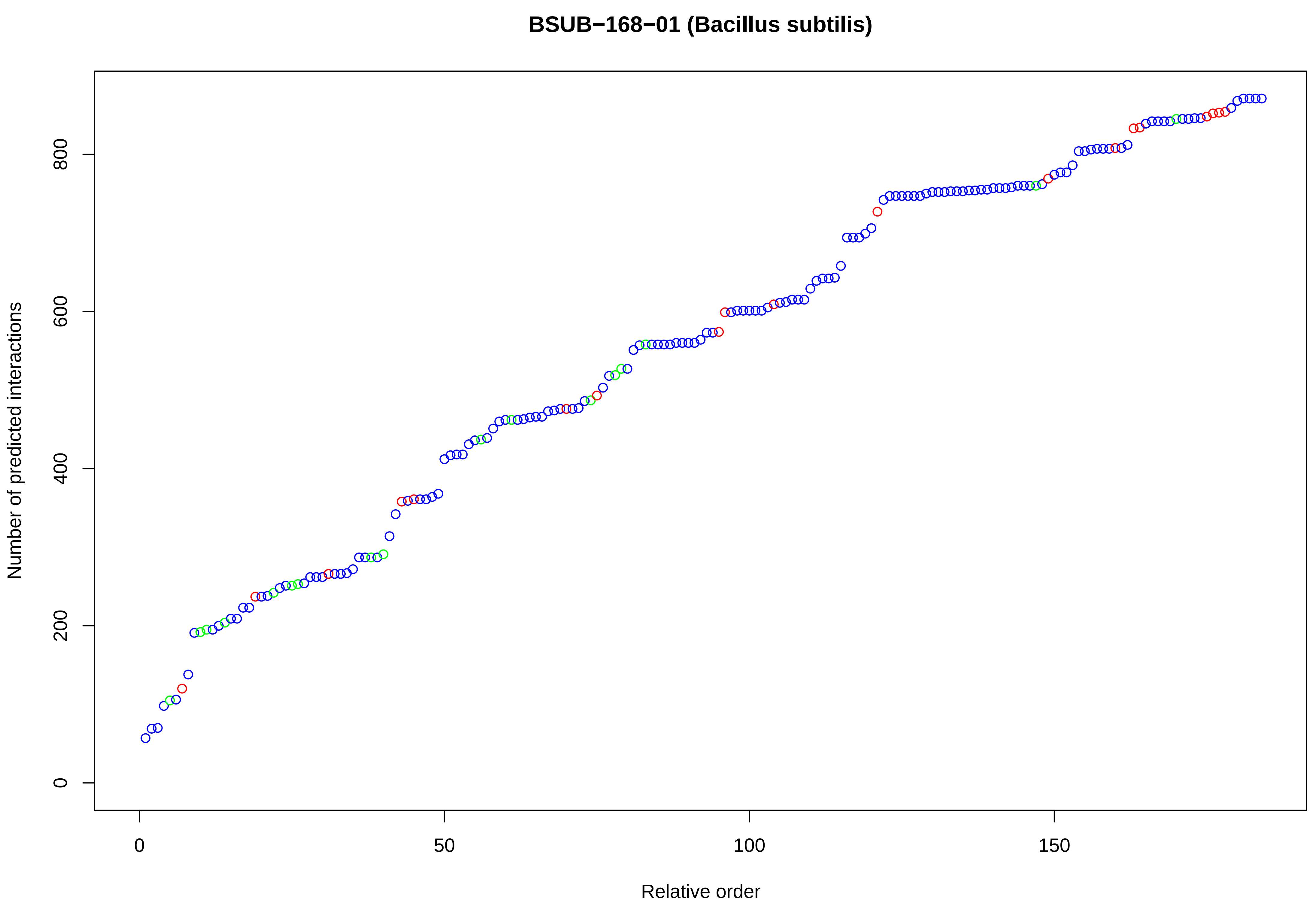
BSUB−168−01 (Bacillus subtilis)
Number of predicted interactions
0
200
400
600
800
0
50
100
150
Relative order

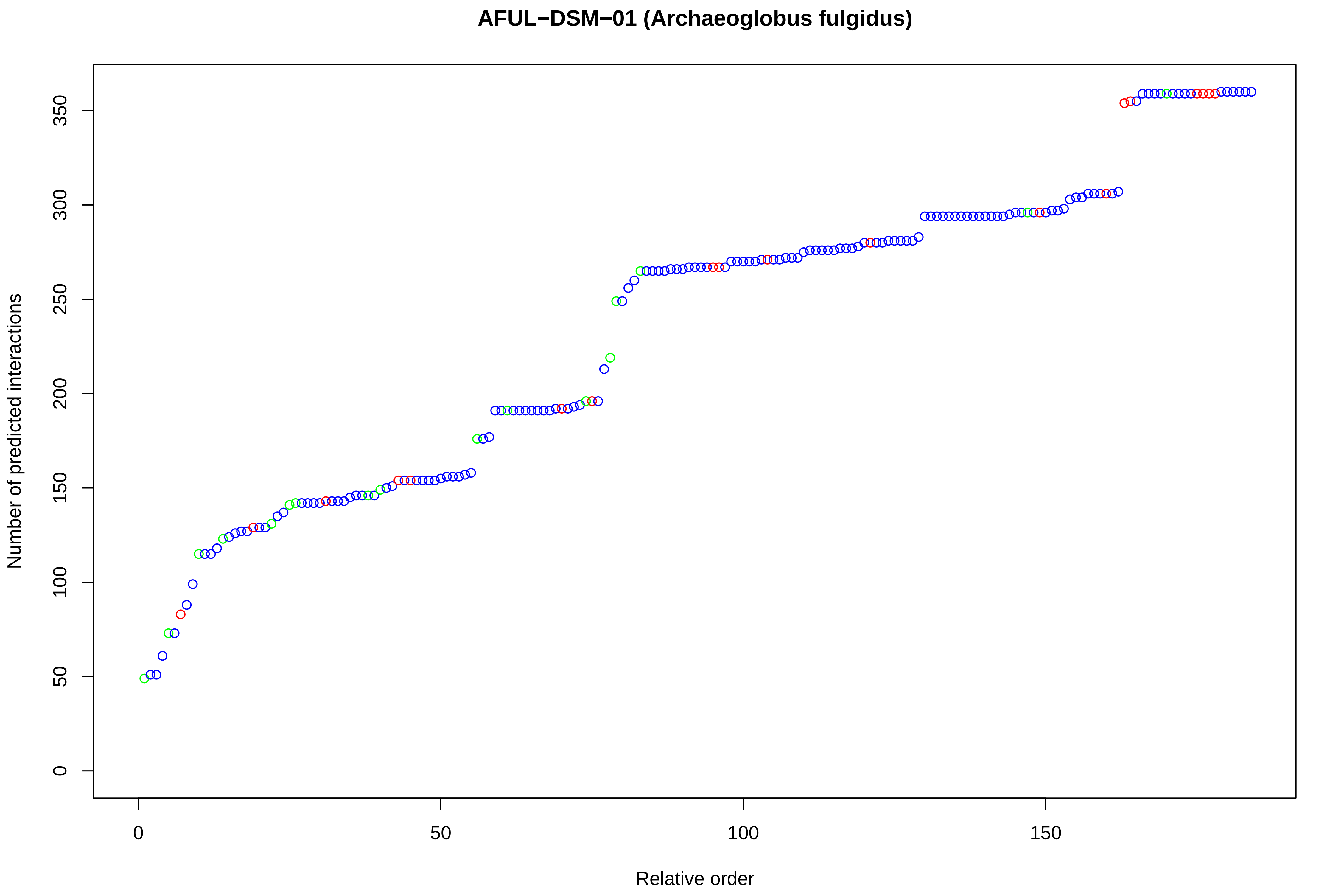
AFUL−DSM−01 (Archaeoglobus fulgidus)
Number of predicted interactions
Relative order
0
50
100
150
200
250
300
350
0
50
100
150

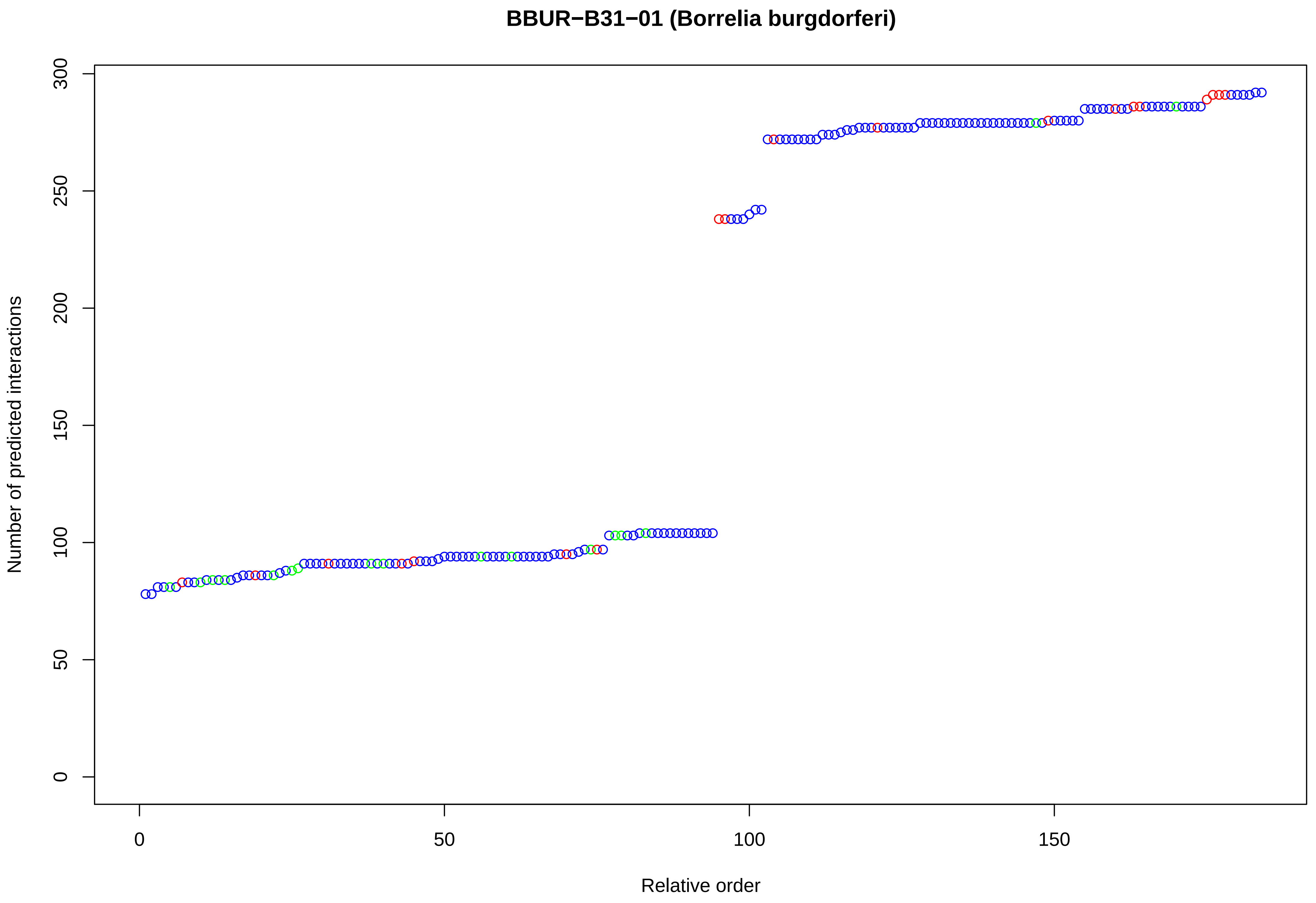
BBUR−B31−01 (Borrelia burgdorferi)
Number of predicted interactions
Relative order
0
50
100
150
200
250
300
0
50
100
150

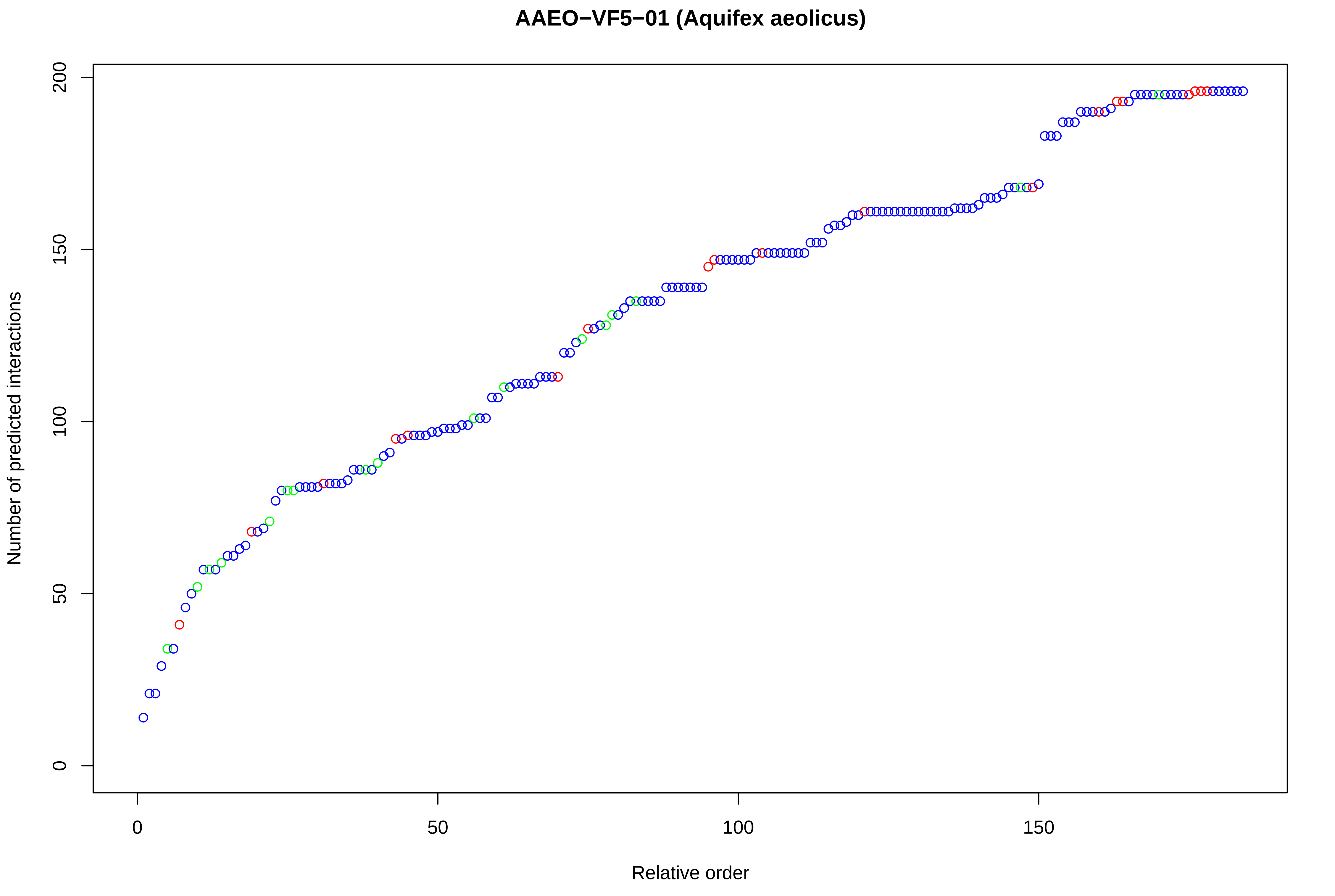
AAEO−VF5−01 (Aquifex aeolicus)
Number of predicted interactions
Relative order
0
50
100
150
200
0
50
100
150

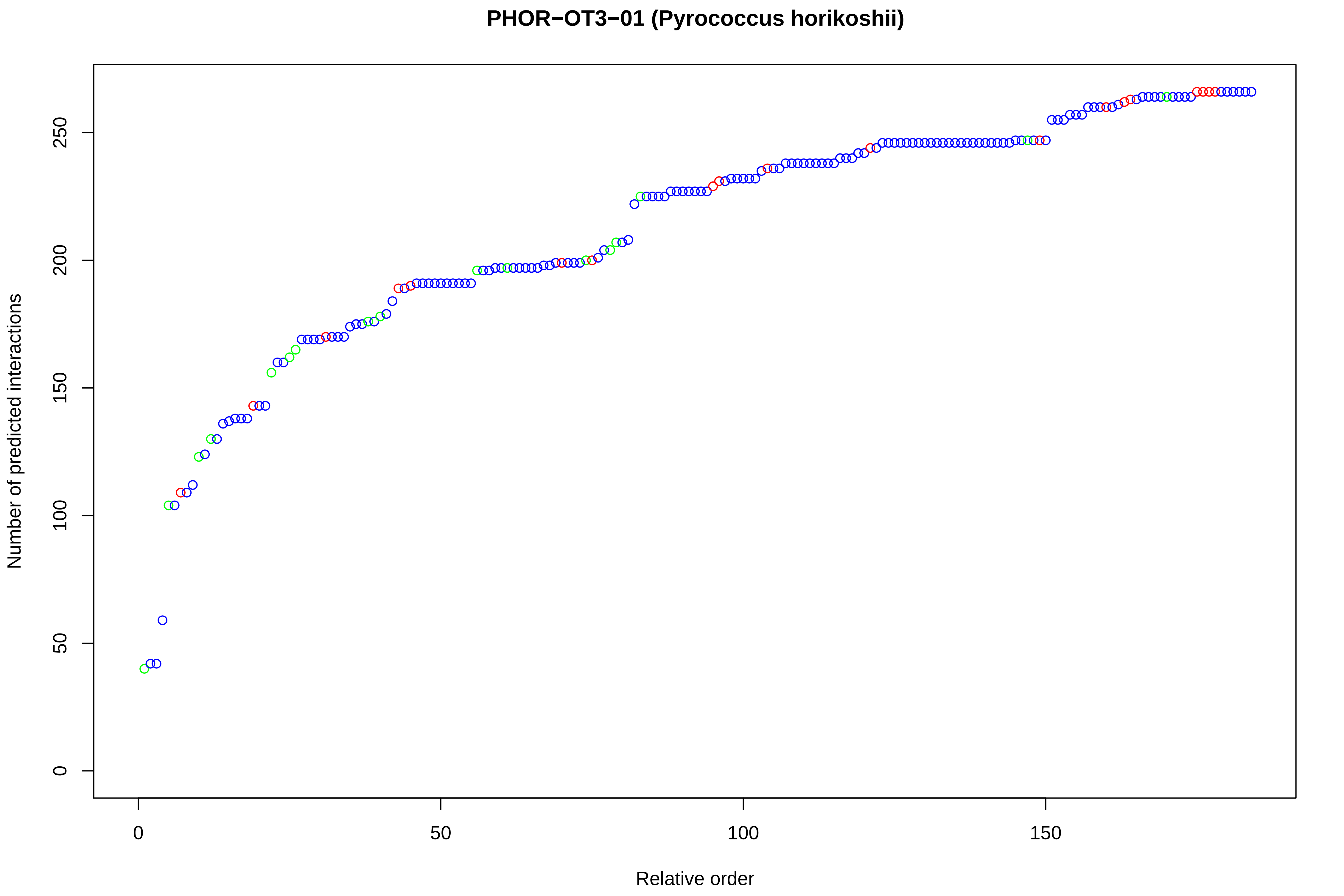
PHOR−OT3−01 (Pyrococcus horikoshii)
Number of predicted interactions
Relative order
0
50
100
150
200
250

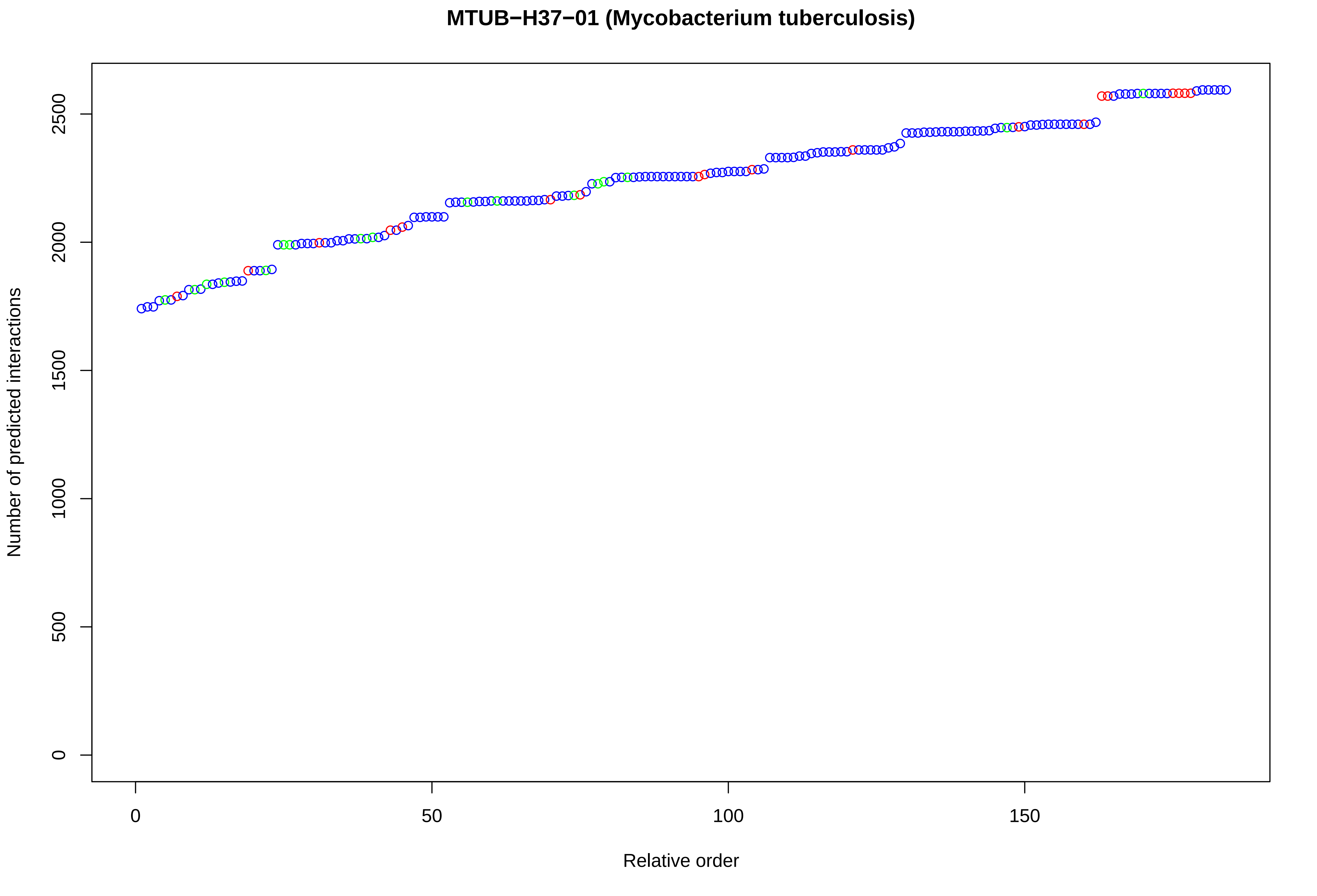
MTUB−H37−01 (Mycobacterium tuberculosis)
Number of predicted interactions
Relative order
0
500
1000
1500
2000
2500
0
50
100
150

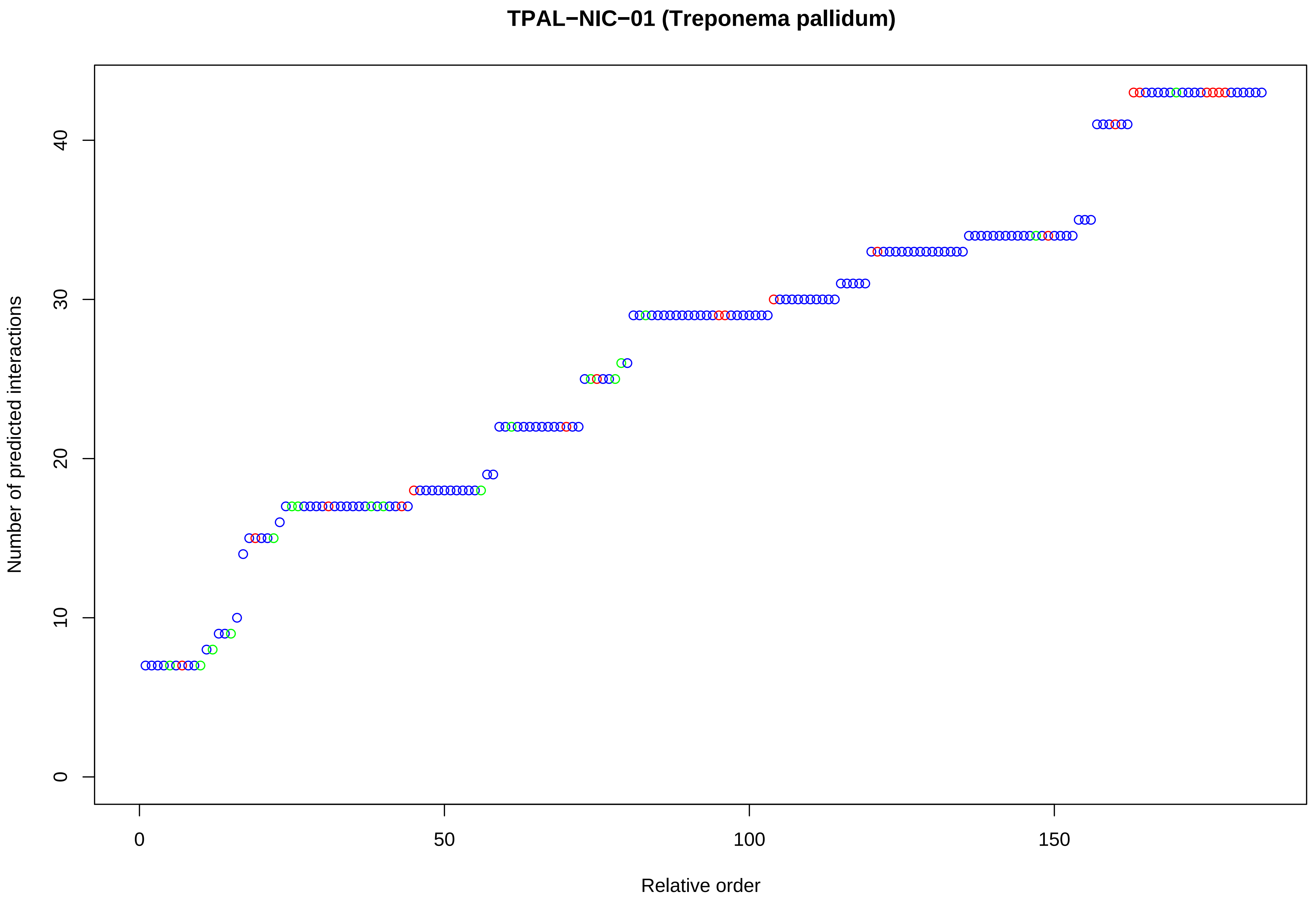

TPAL−NIC−01 (Treponema pallidum)
Number of predicted interactions
Relative order
0
10
20
30
40
0
50
100
150

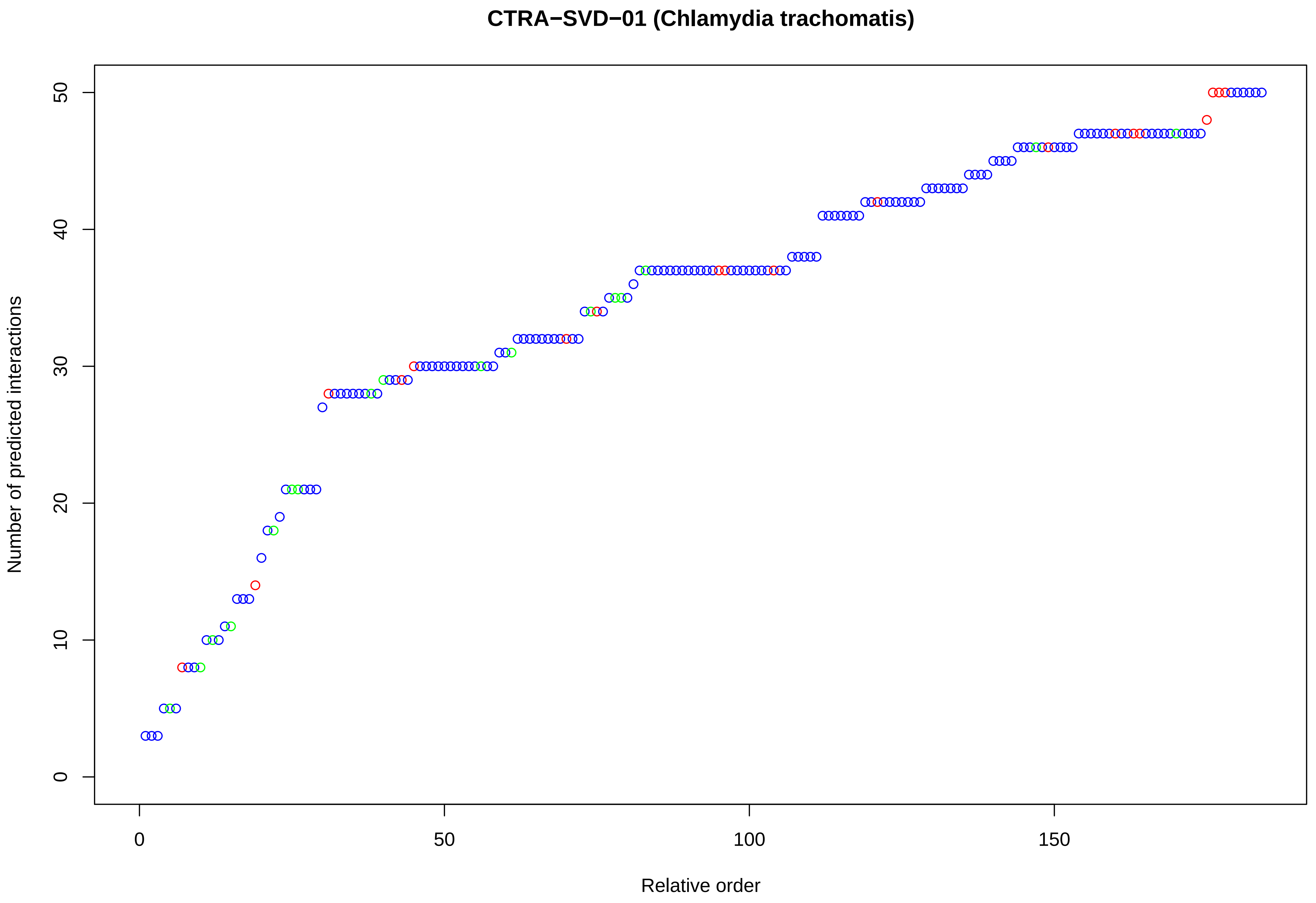

CTRA−SVD−01 (Chlamydia trachomatis)
Number of predicted interactions
Relative order
0
10
20
30
40
50
0
50
100
150

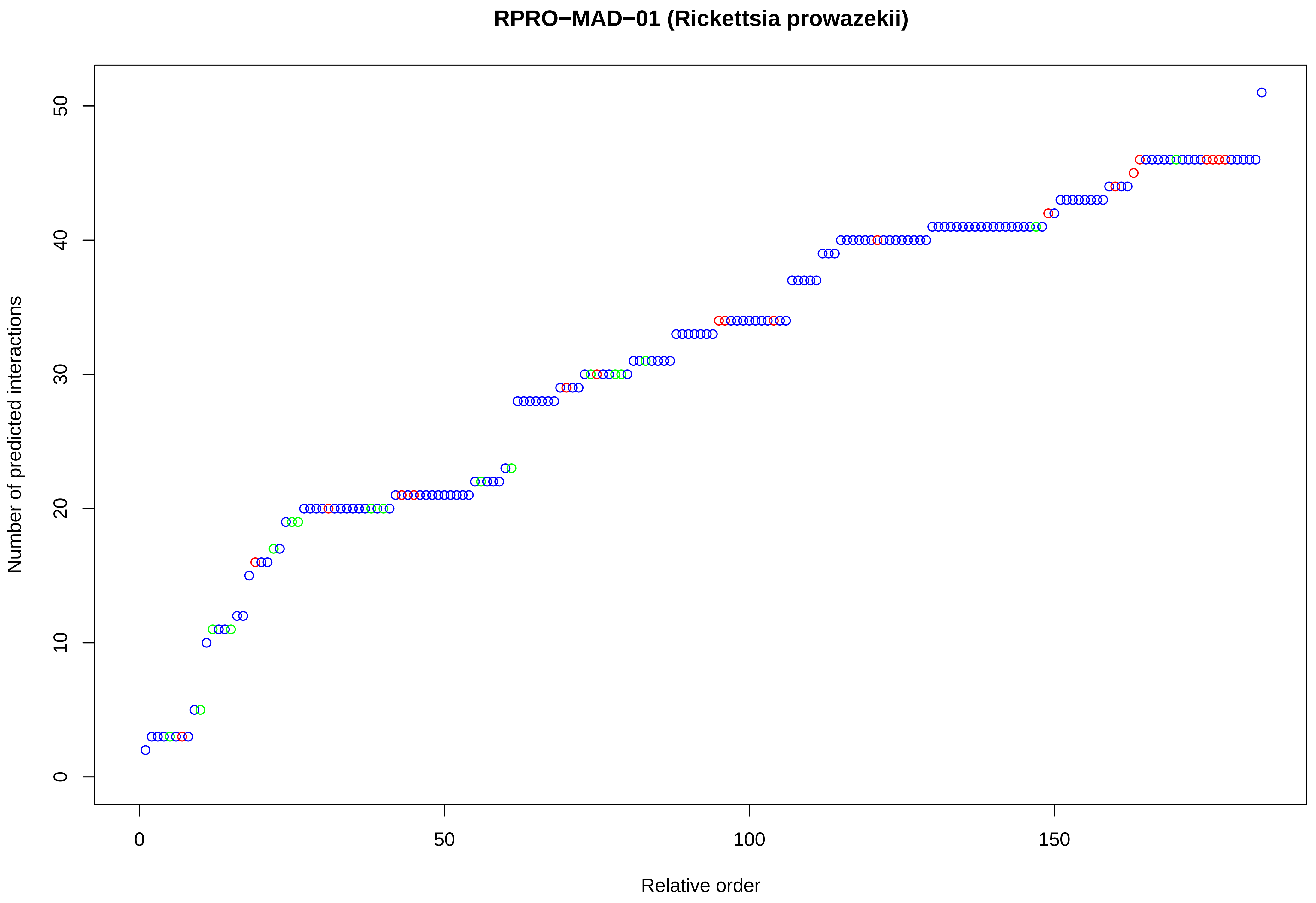
RPRO−MAD−01 (Rickettsia prowazekii)
Number of predicted interactions
Relative order
0
10
20
30
40
50
0
50
100
150

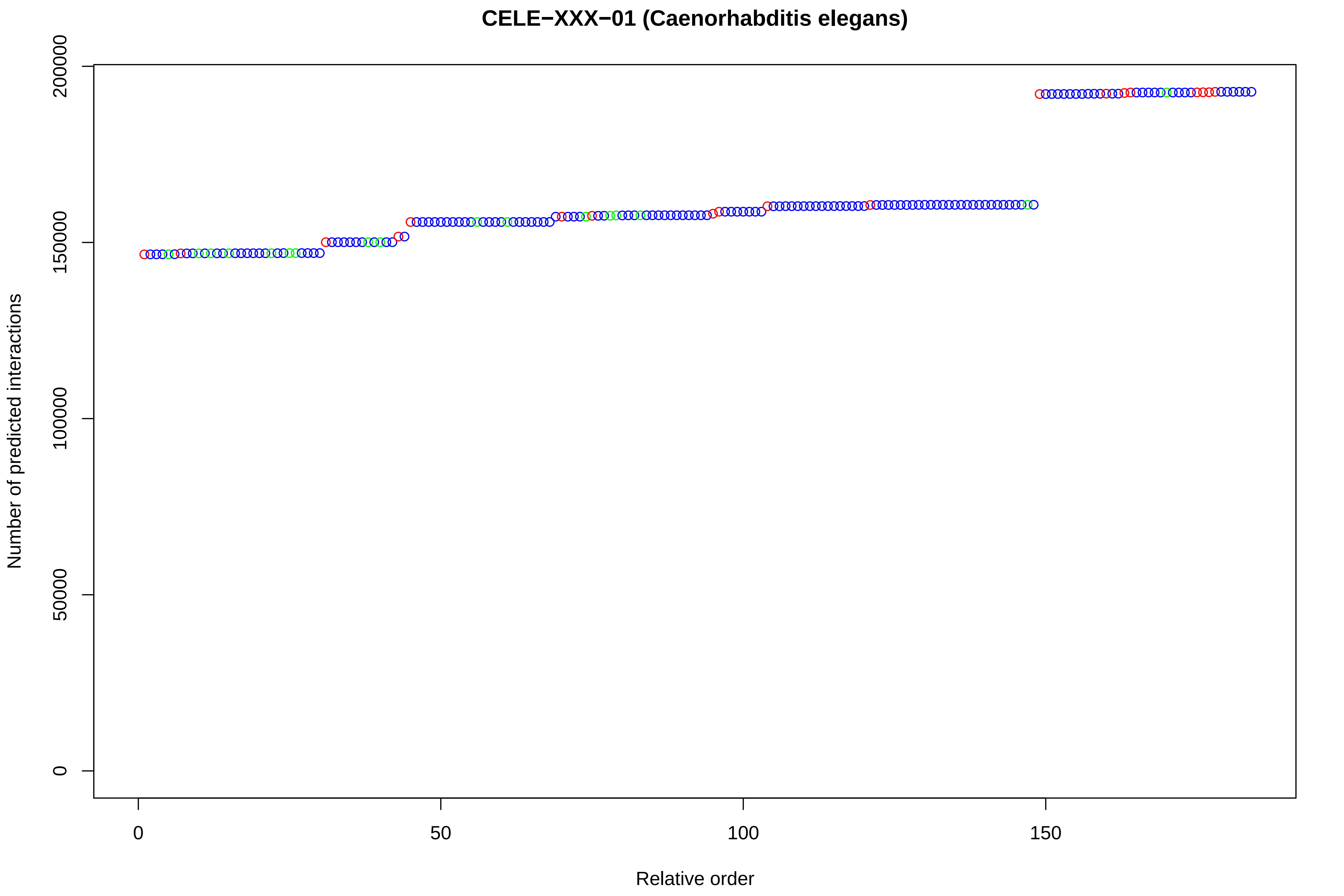
CELE−XXX−01 (Caenorhabditis elegans)
Number of predicted interactions
0
50000
100000
150000
200000
Relative order
0
50
100
150

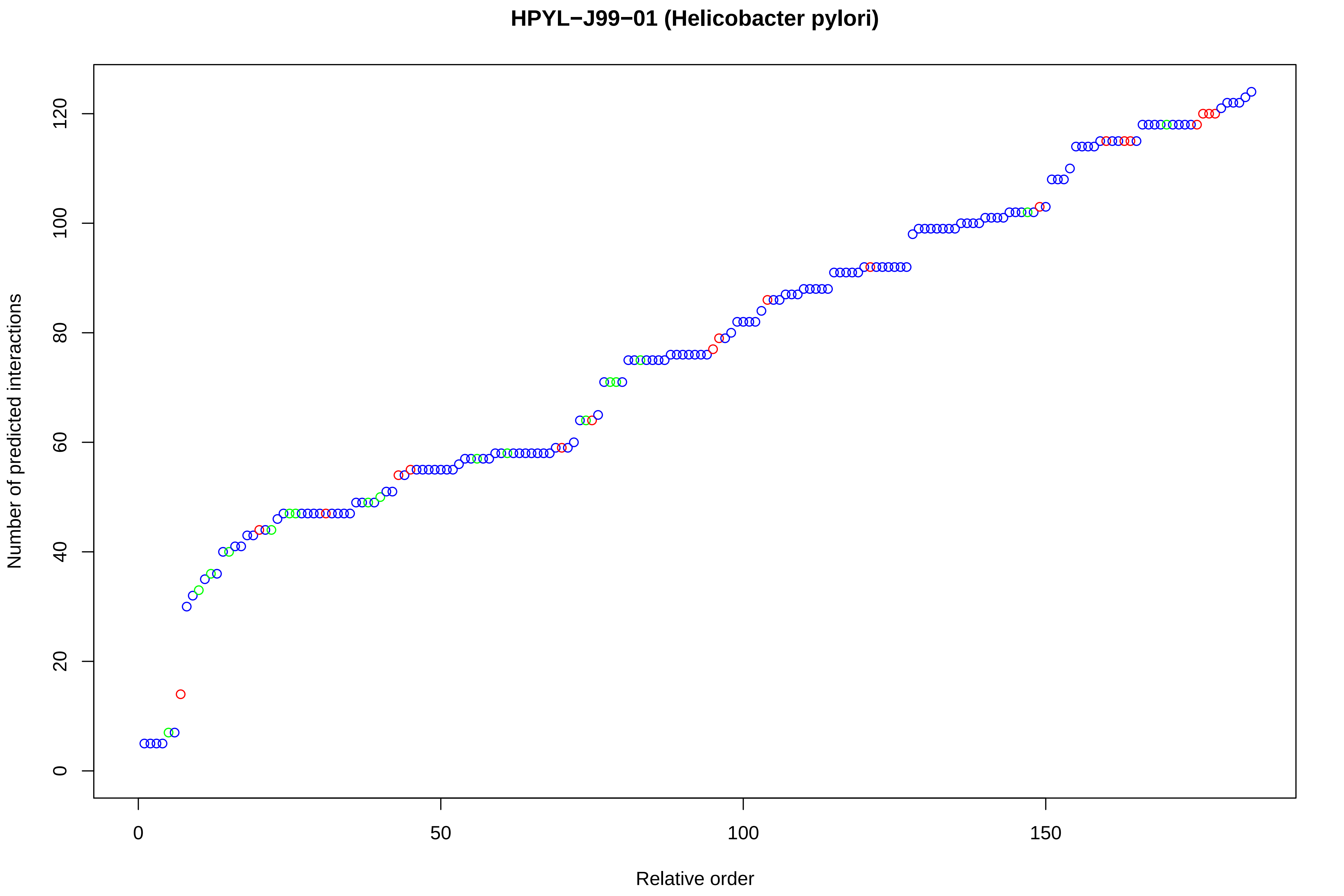

HPYL−J99−01 (Helicobacter pylori)
Number of predicted interactions
0
20
40
60
80
100
120
0
50
100
150
Relative order

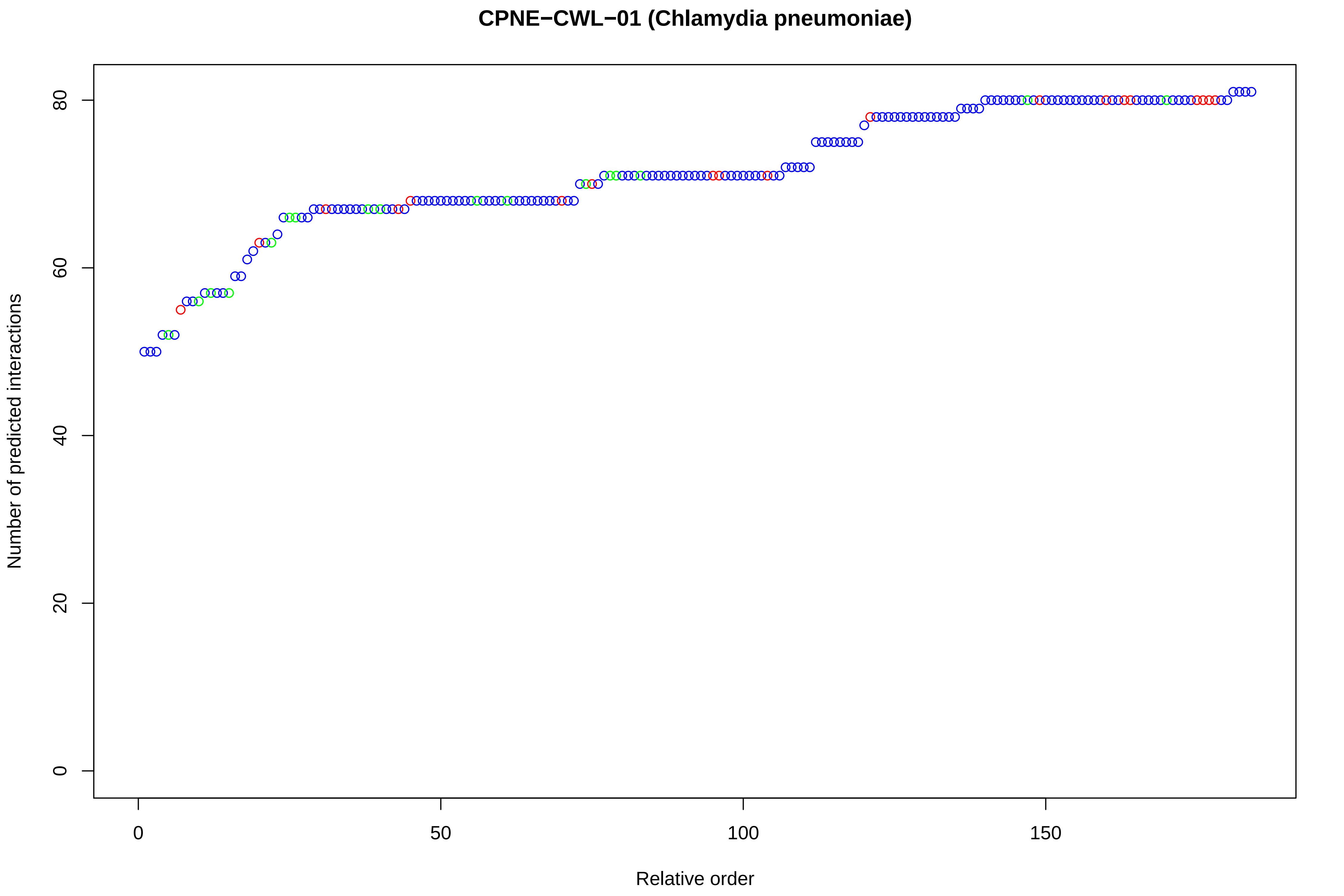

CPNE−CWL−01 (Chlamydia pneumoniae)
Number of predicted interactions
Relative order
0
20
40
60
80
0
50
100
150

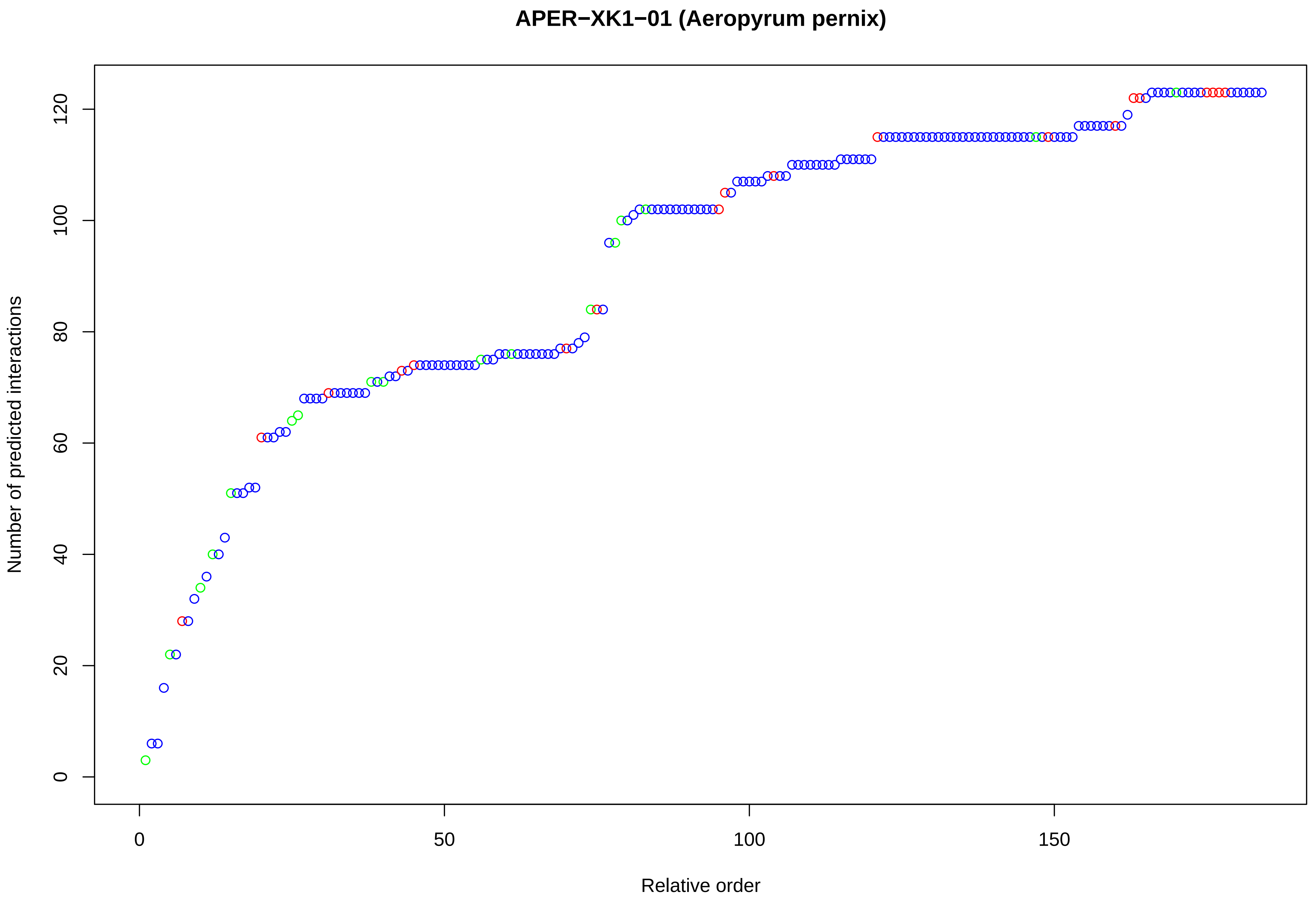
APER−XK1−01 (Aeropyrum pernix)
Number of predicted interactions
Relative order
0
20
40
60
80
100
120
0
50
100
150

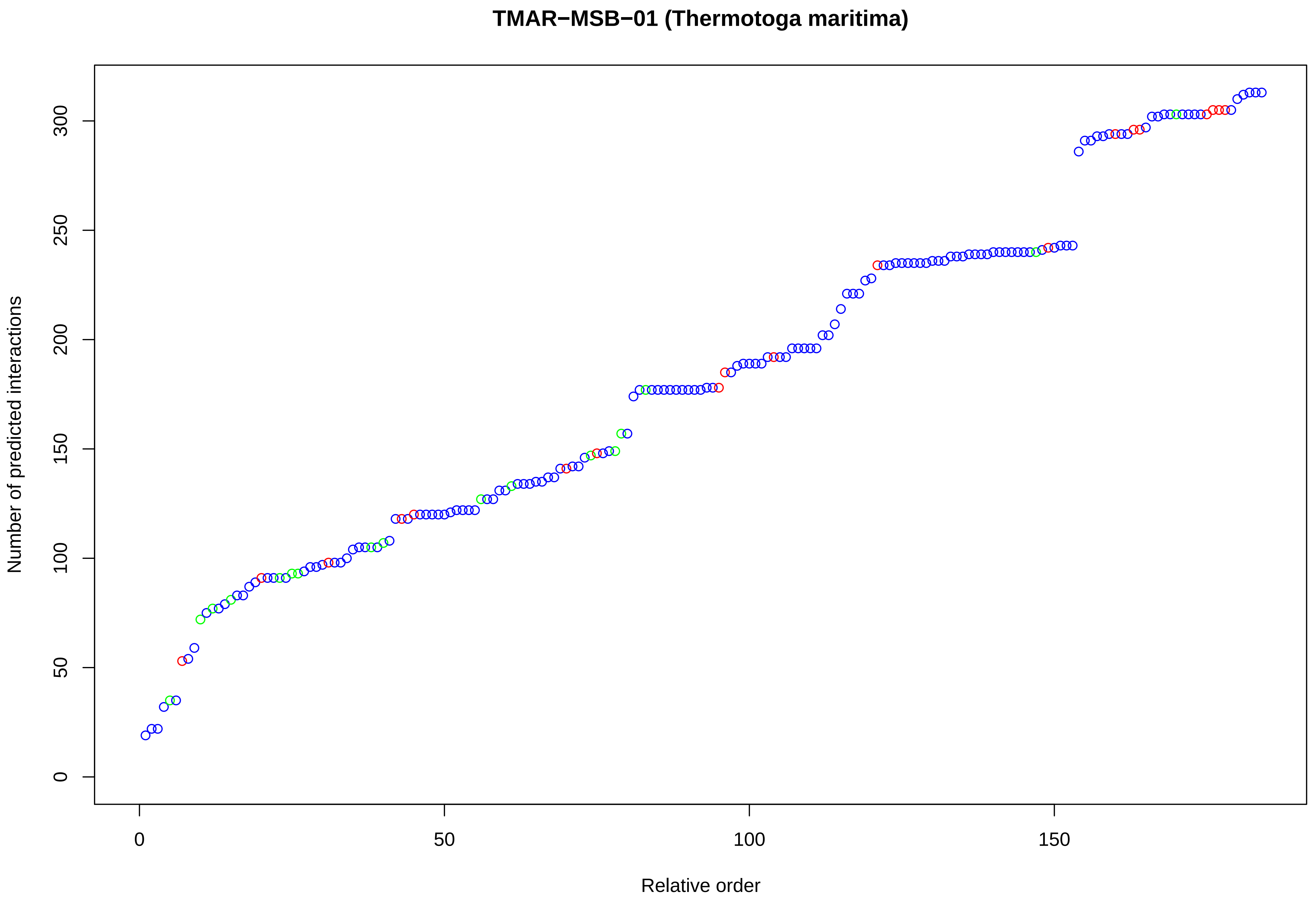

TMAR−MSB−01 (Thermotoga maritima)
Number of predicted interactions
Relative order
0
50
100
150
200
250
300

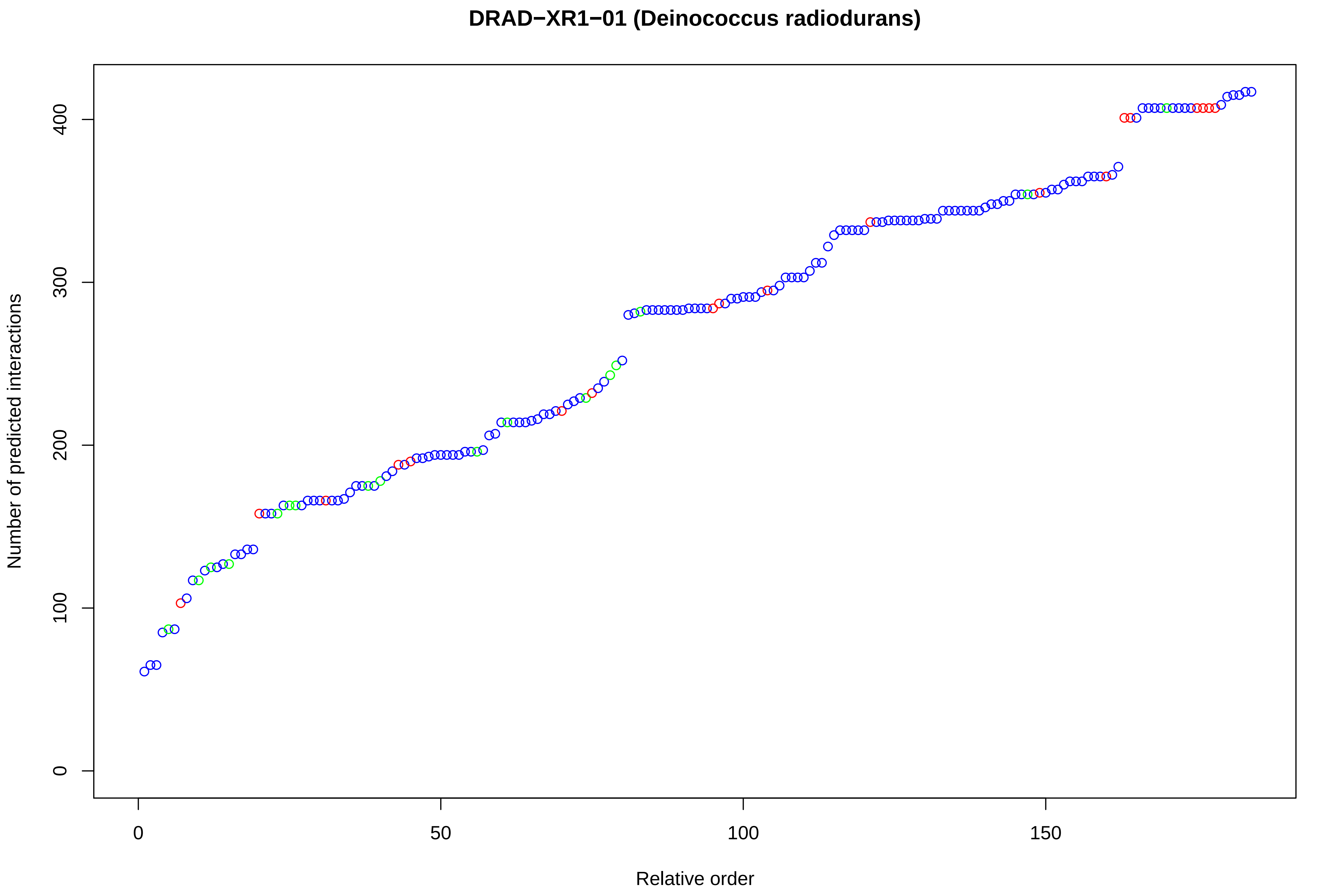
DRAD−XR1−01 (Deinococcus radiodurans)
Number of predicted interactions
Relative order
0
100
200
300
400
0
50
100
150

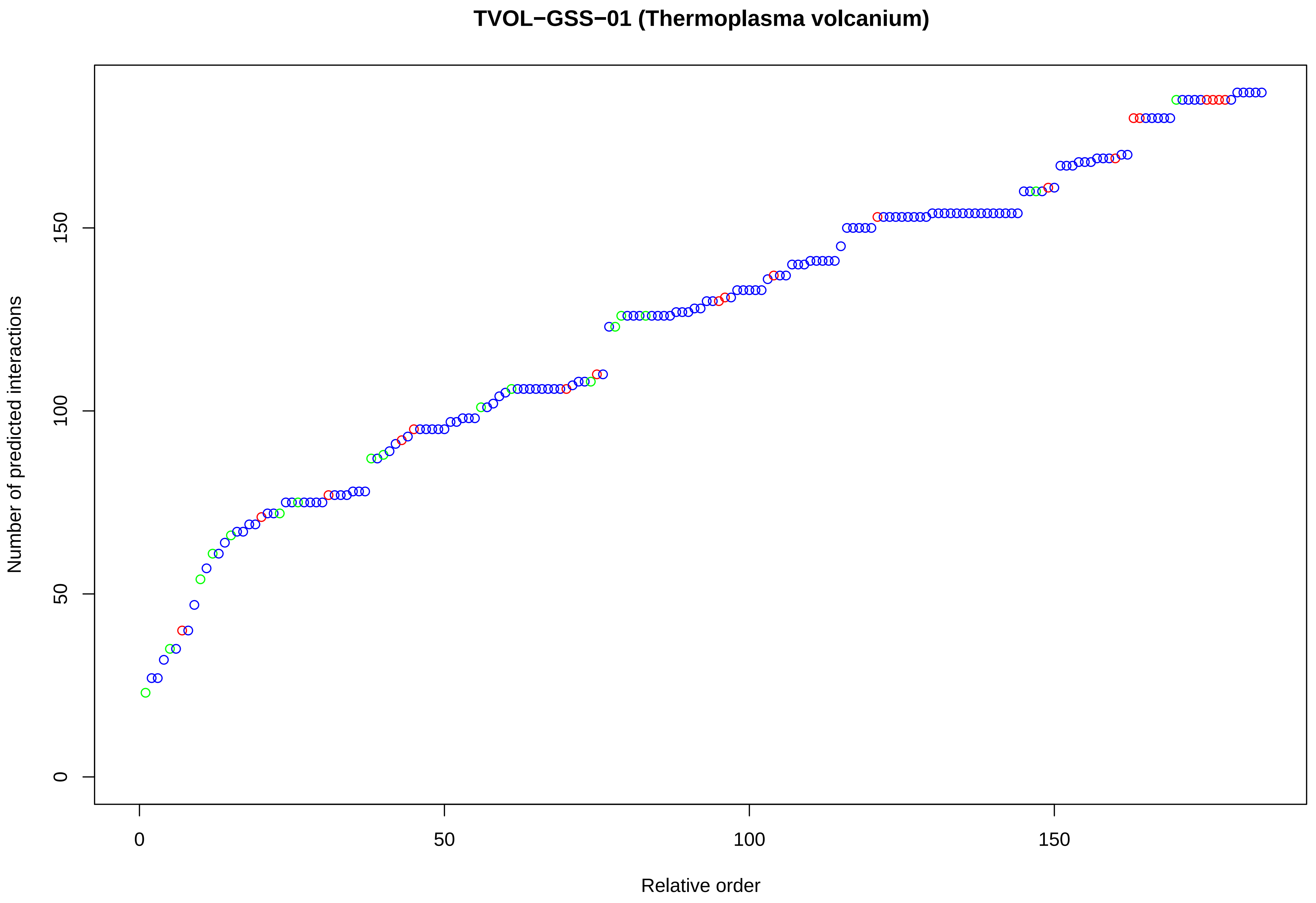
TVOL−GSS−01 (Thermoplasma volcanium)
Number of predicted interactions
Relative order
0
50
100
150
0
50
100
150

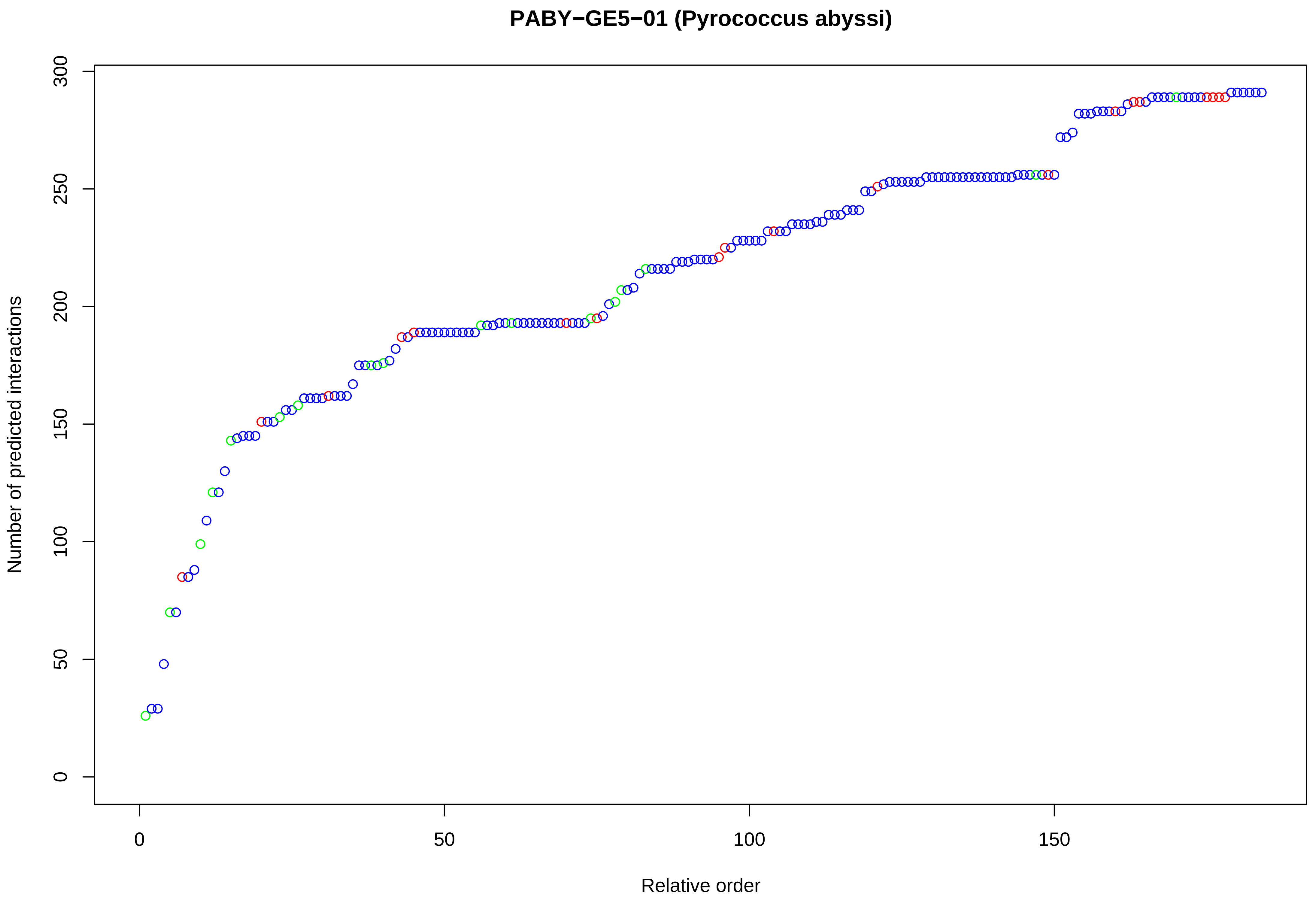

PABY−GE5−01 (Pyrococcus abyssi)
Number of predicted interactions
Relative order
0
50
100
150
200
250
300

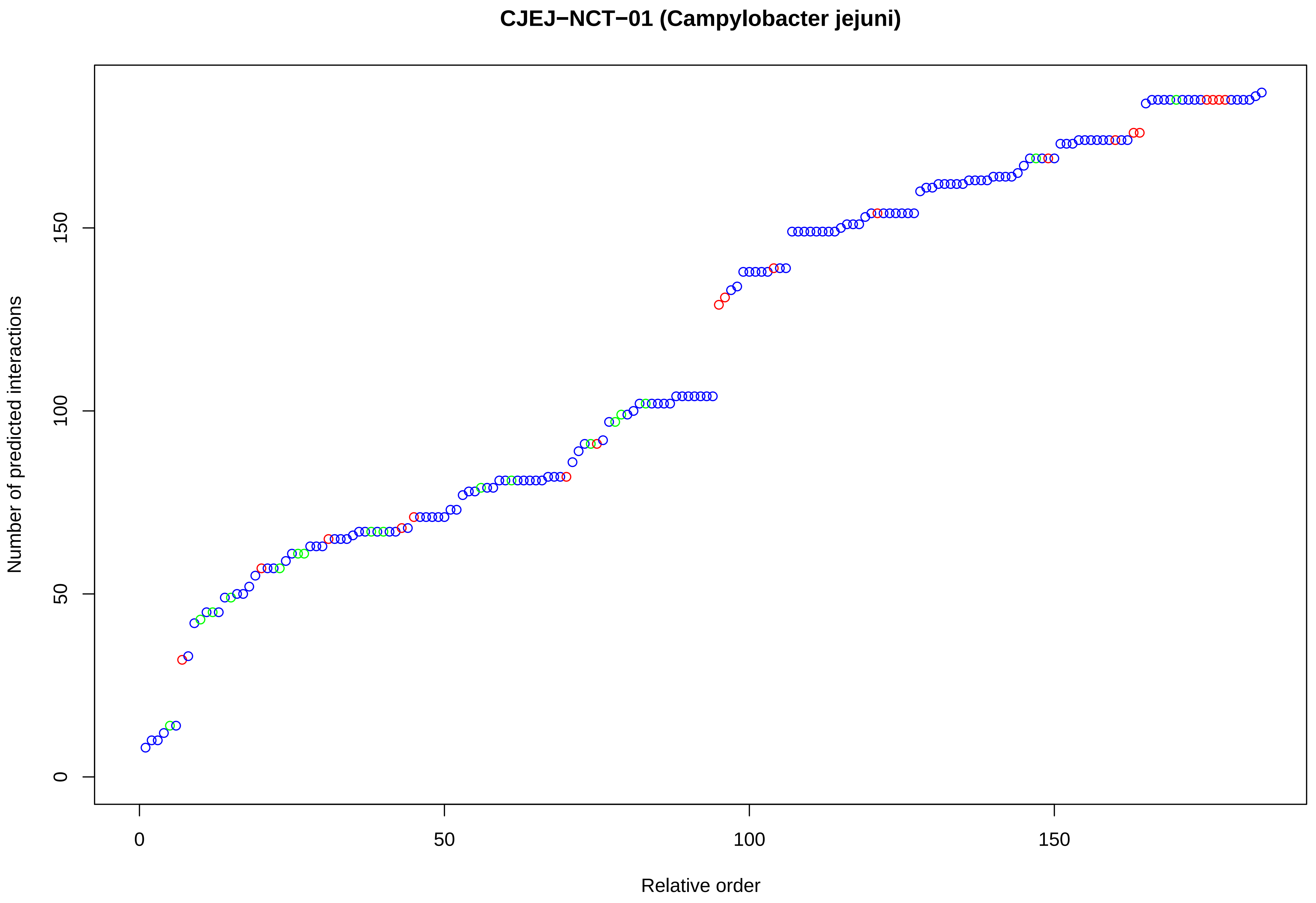
CJEJ−NCT−01 (Campylobacter jejuni)
Number of predicted interactions
0
50
100
150
Relative order
0
50
100
150

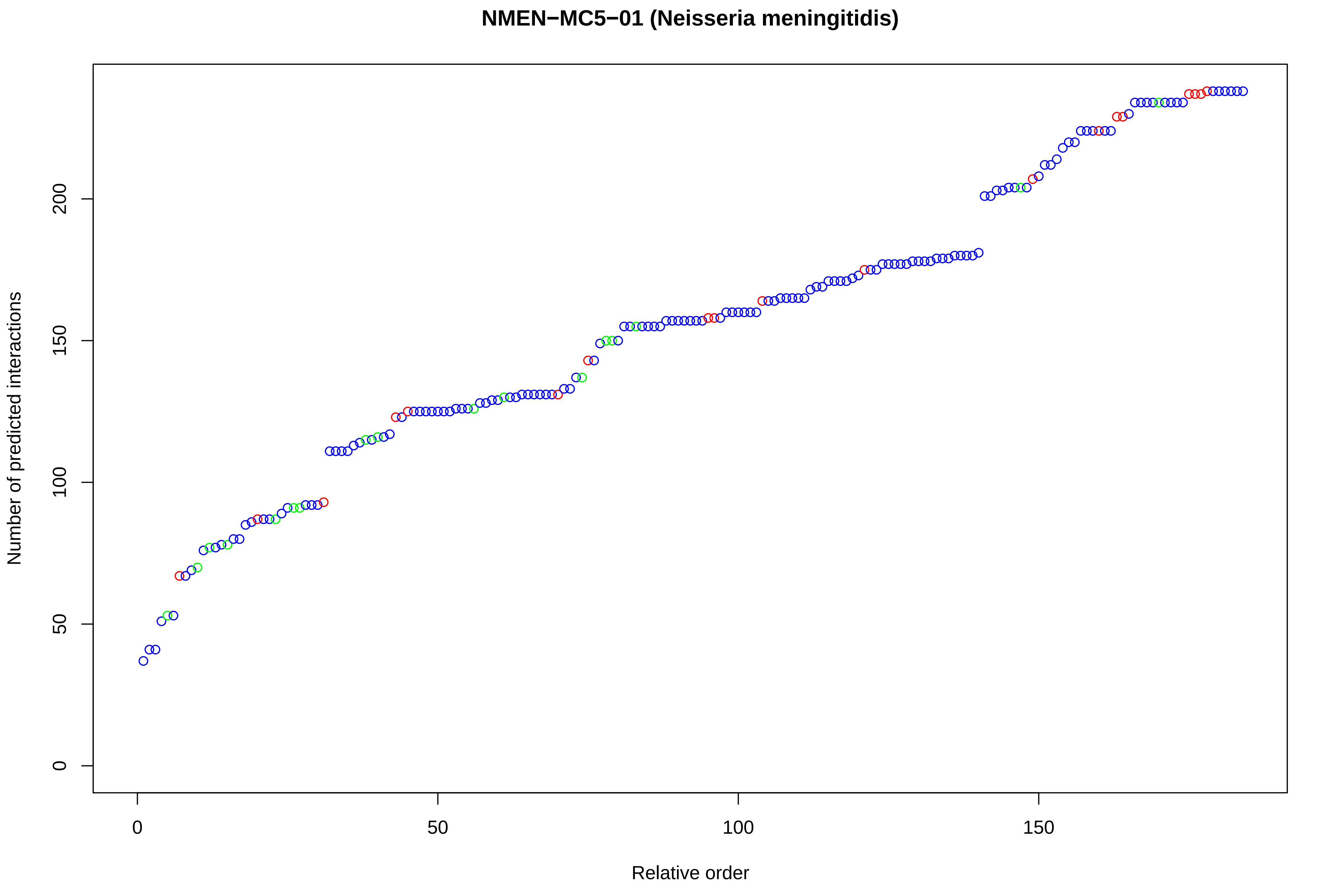

NMEN−MC5−01 (Neisseria meningitidis)
Number of predicted interactions
0
50
100
150
200
Relative order
0
50
100
150

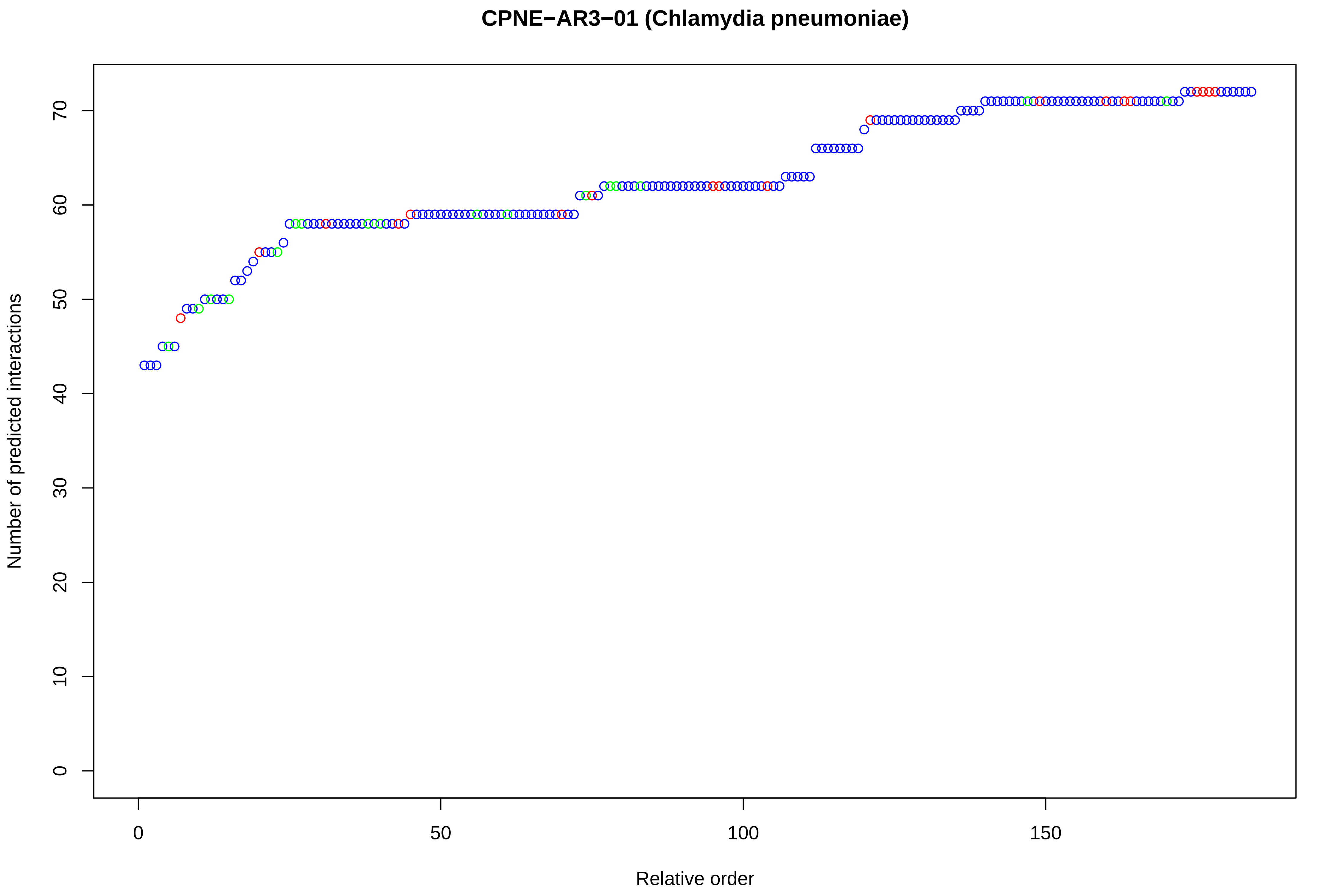

CPNE−AR3−01 (Chlamydia pneumoniae)
Number of predicted interactions
Relative order
0
10
20
30
40
50
60
70
0
50
100
150

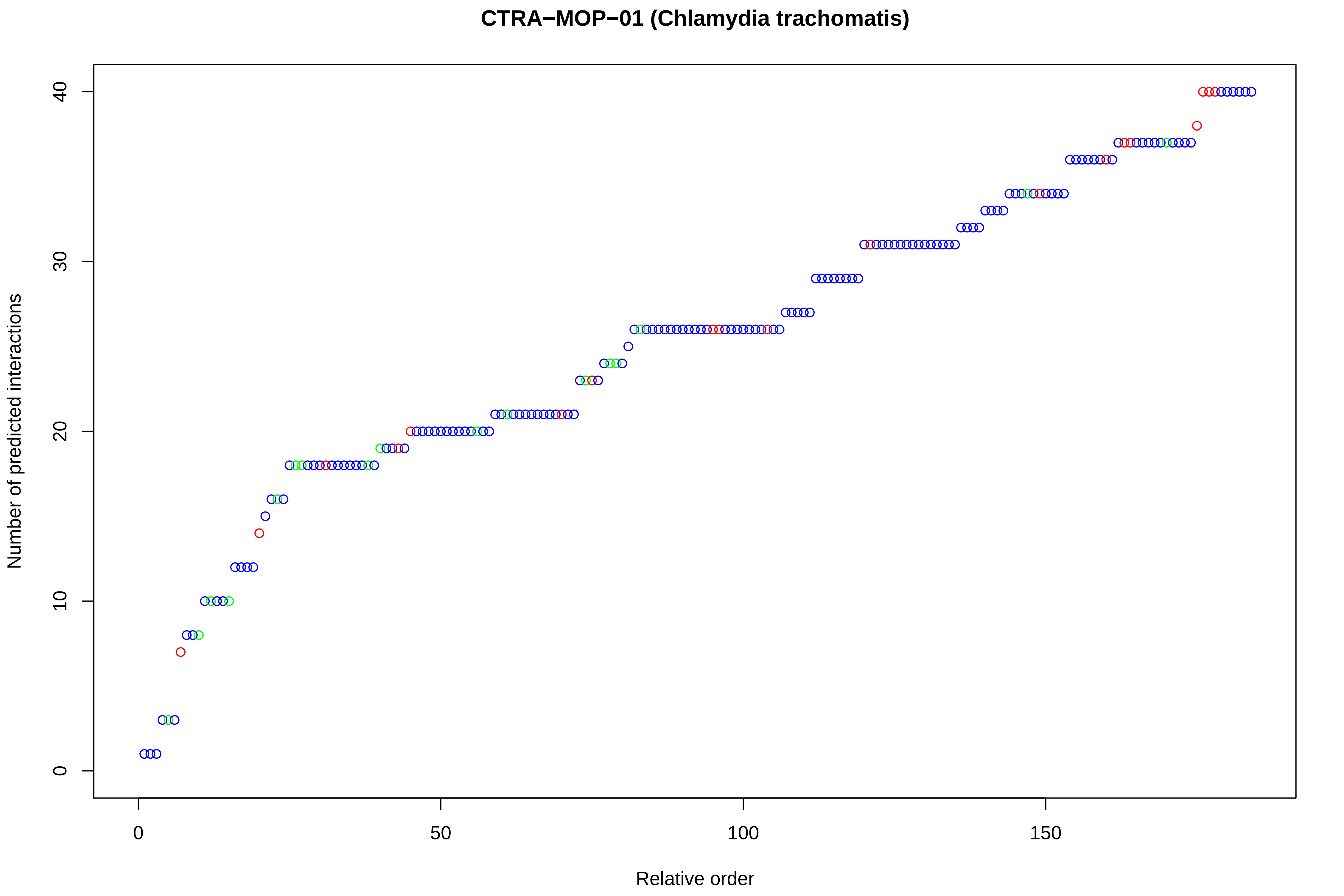

CTRA−MOP−01 (Chlamydia trachomatis)
Number of predicted interactions
Relative order
0
10
20
30
40
0
50
100
150

DMEL−XXX−02 (Drosophila melanogaster)

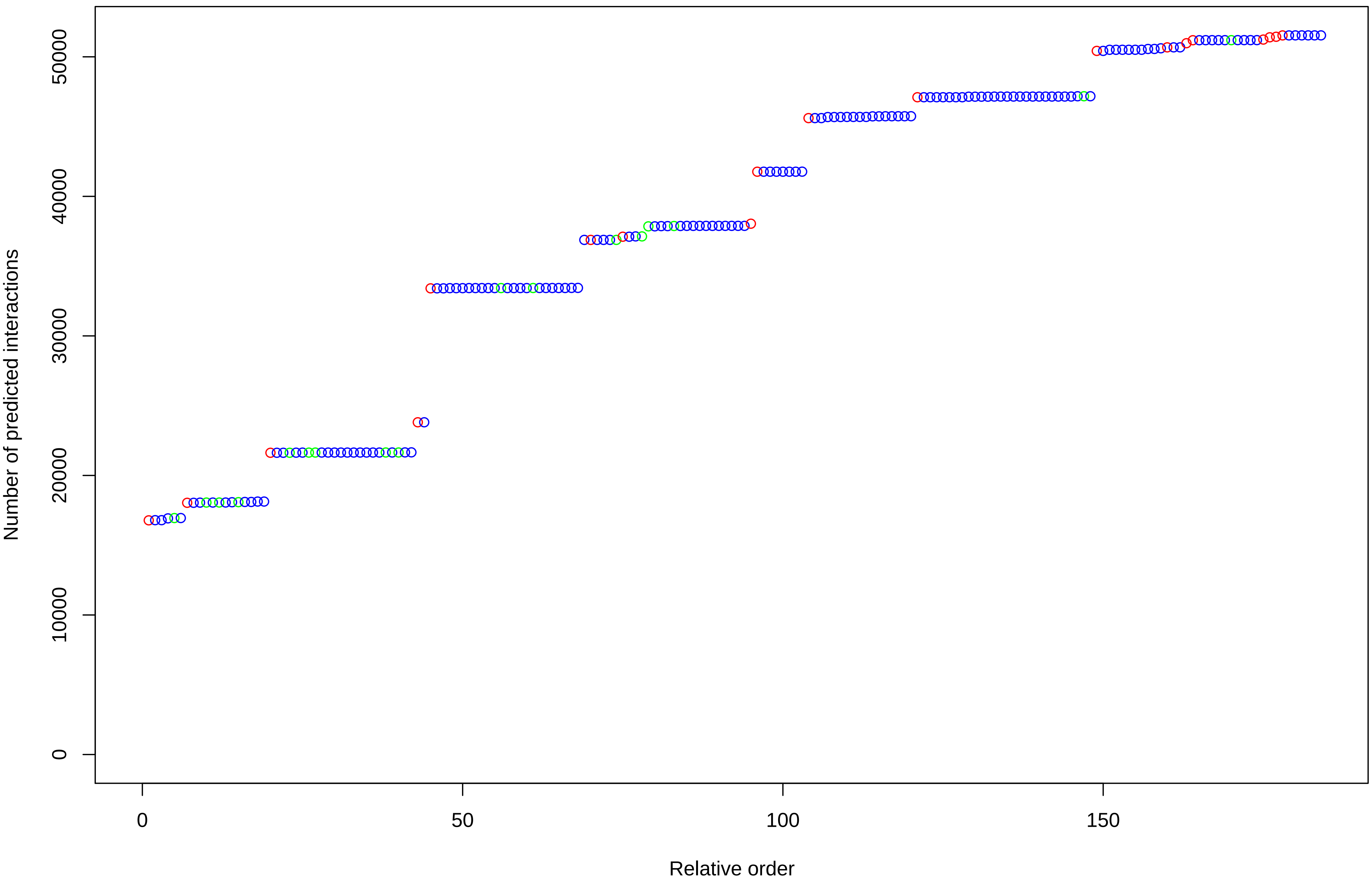

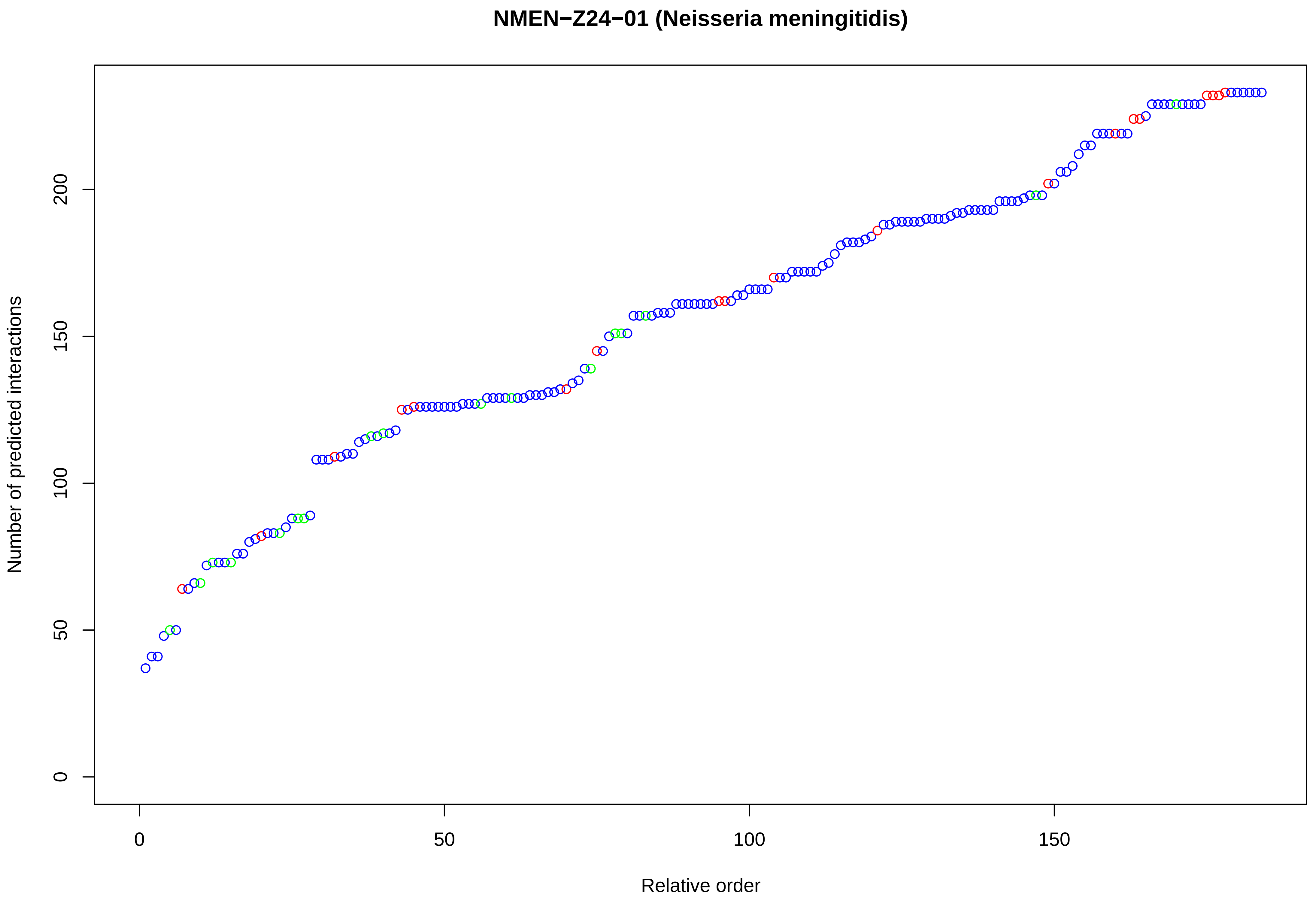
NMEN−Z24−01 (Neisseria meningitidis)
Number of predicted interactions
Relative order
0
50
100
150
200

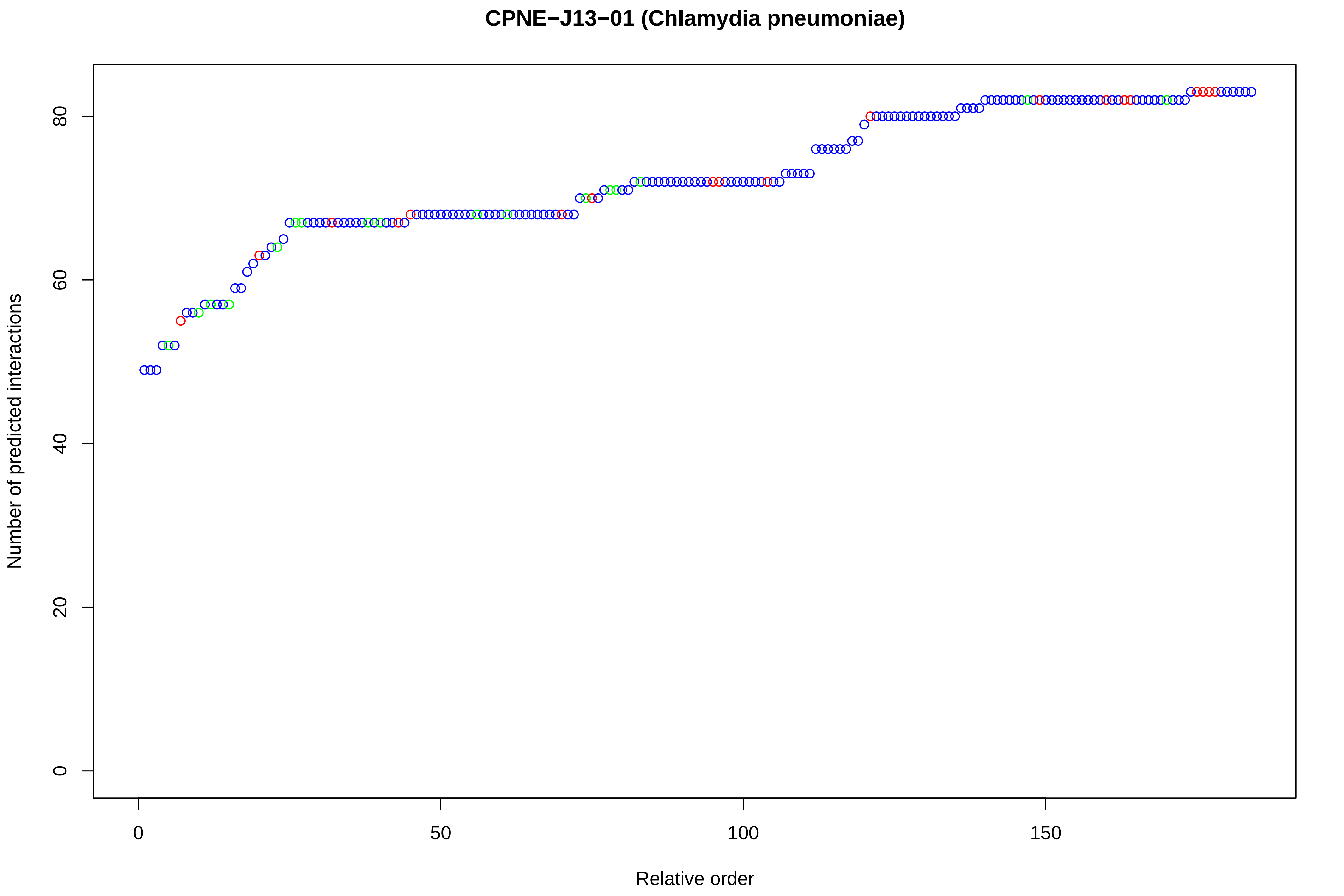
CPNE−J13−01 (Chlamydia pneumoniae)
Number of predicted interactions
Relative order
0
20
40
60
80
0
50
100
150

XFAS−9A5−01 (Xylella fastidiosa)

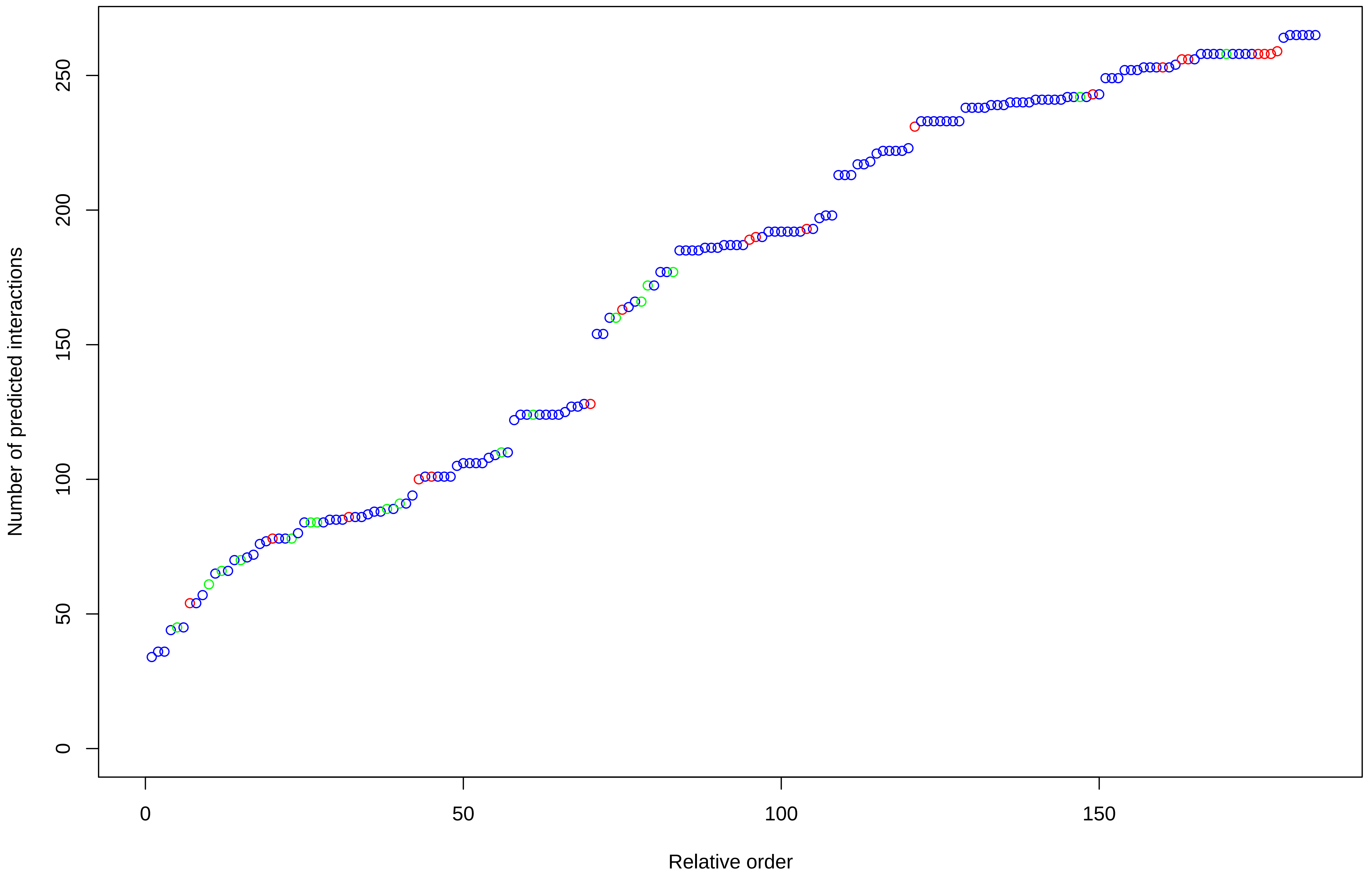

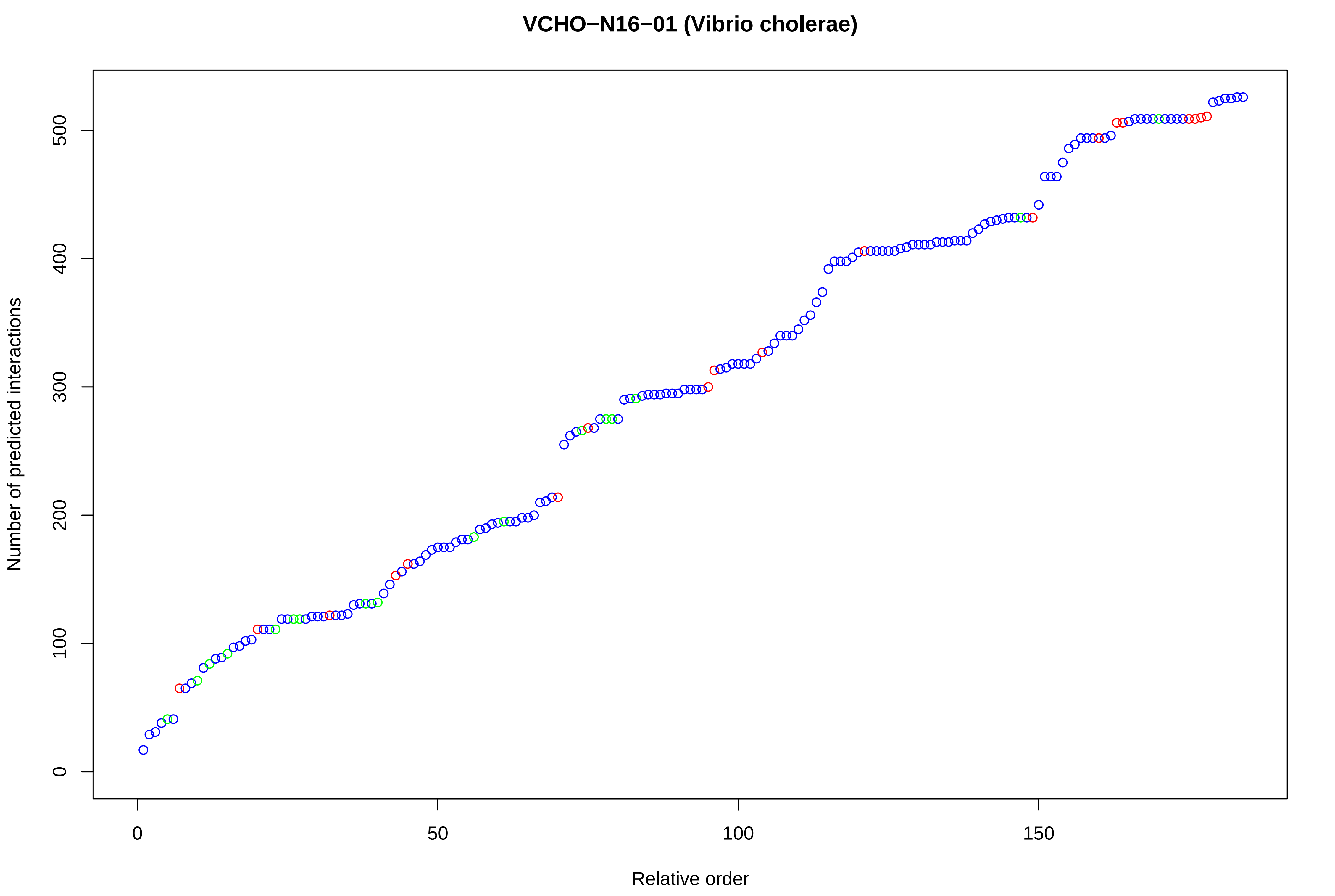
VCHO−N16−01 (Vibrio cholerae)
Number of predicted interactions
Relative order
0
100
200
300
400
500
0
50
100
150

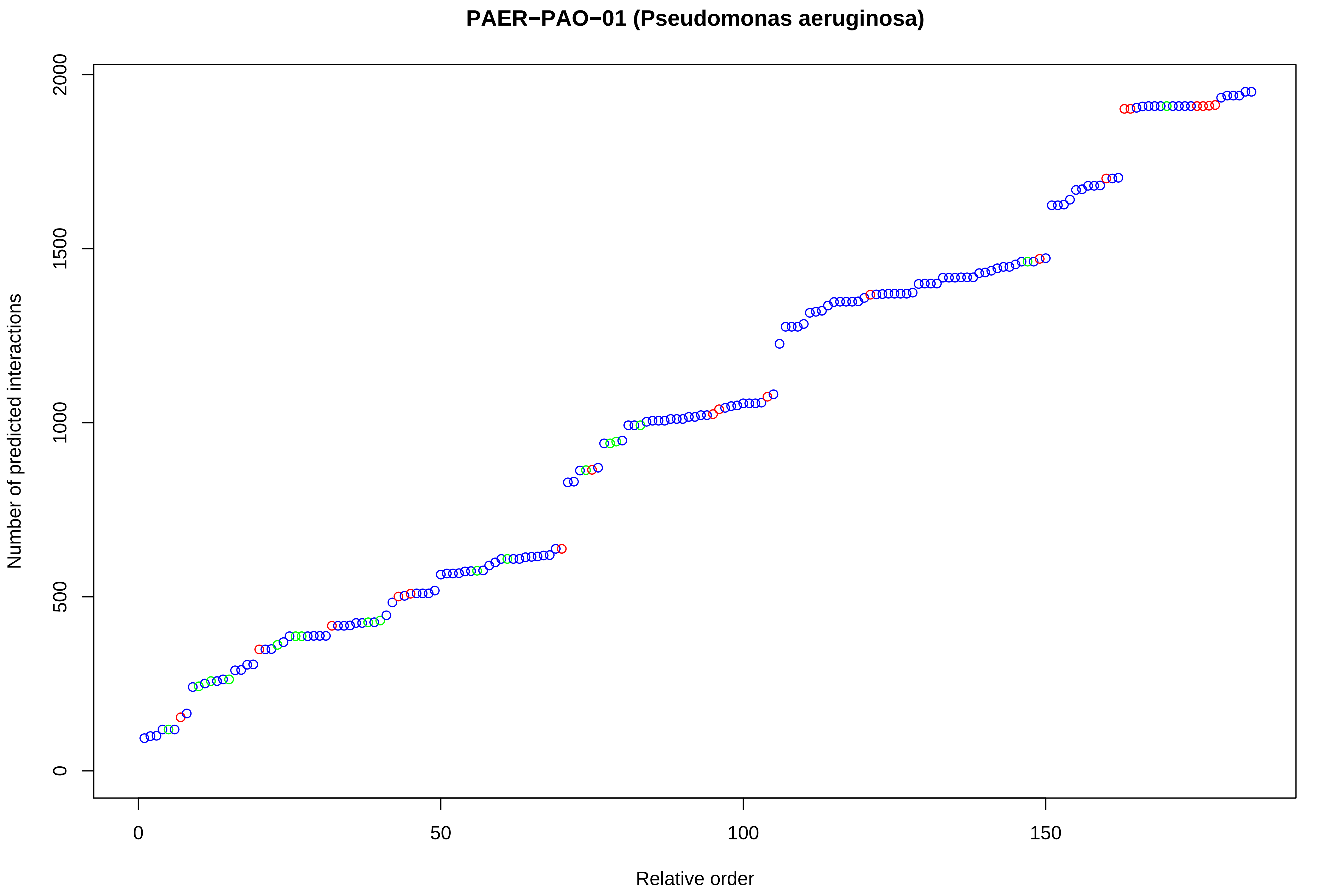

PAER−PAO−01 (Pseudomonas aeruginosa)
Number of predicted interactions
Relative order
0
500
1000
1500
2000
0
50
100
150

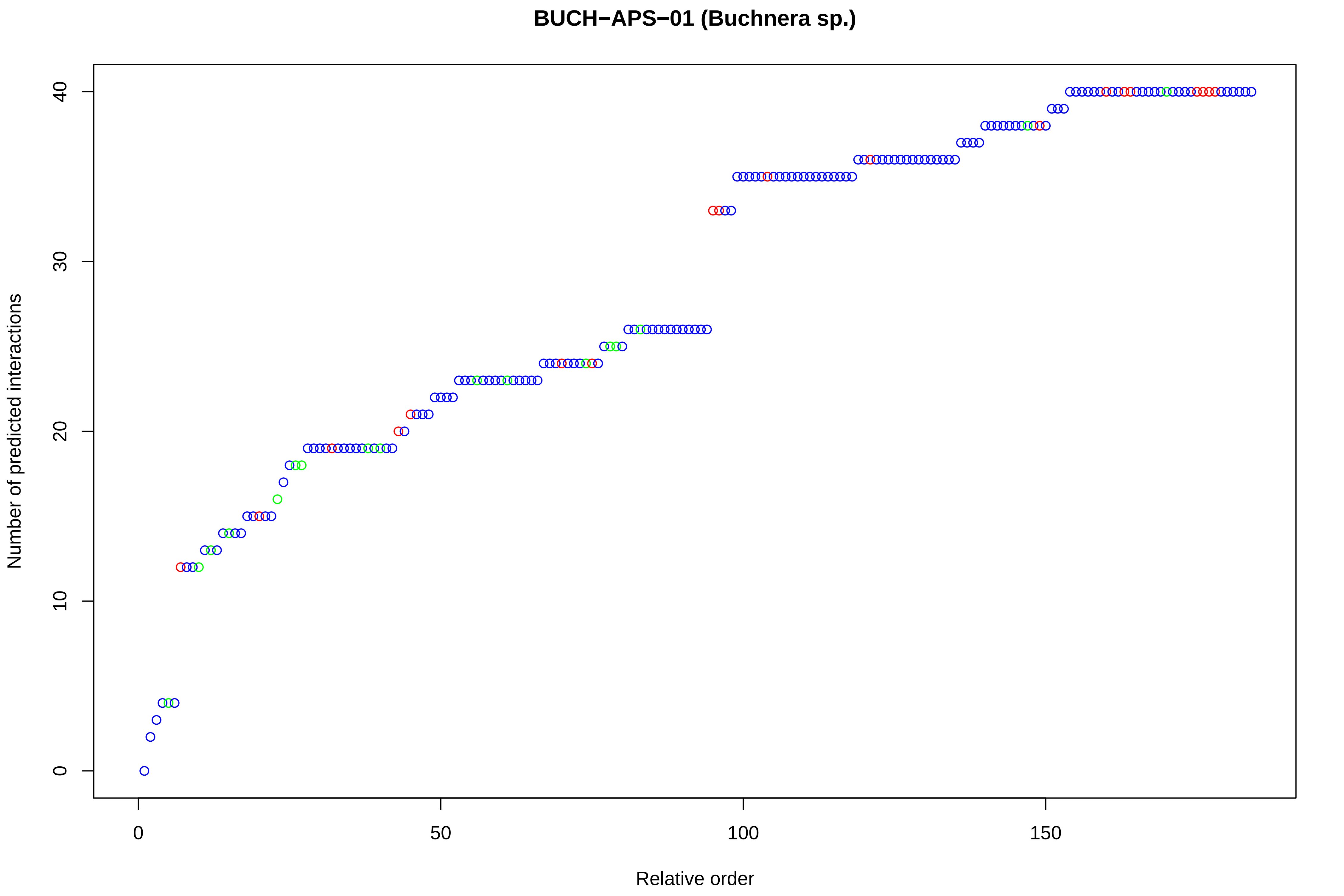
BUCH−APS−01 (Buchnera sp.)
Number of predicted interactions
Relative order
0
10
20
30
40
0
50
100
150

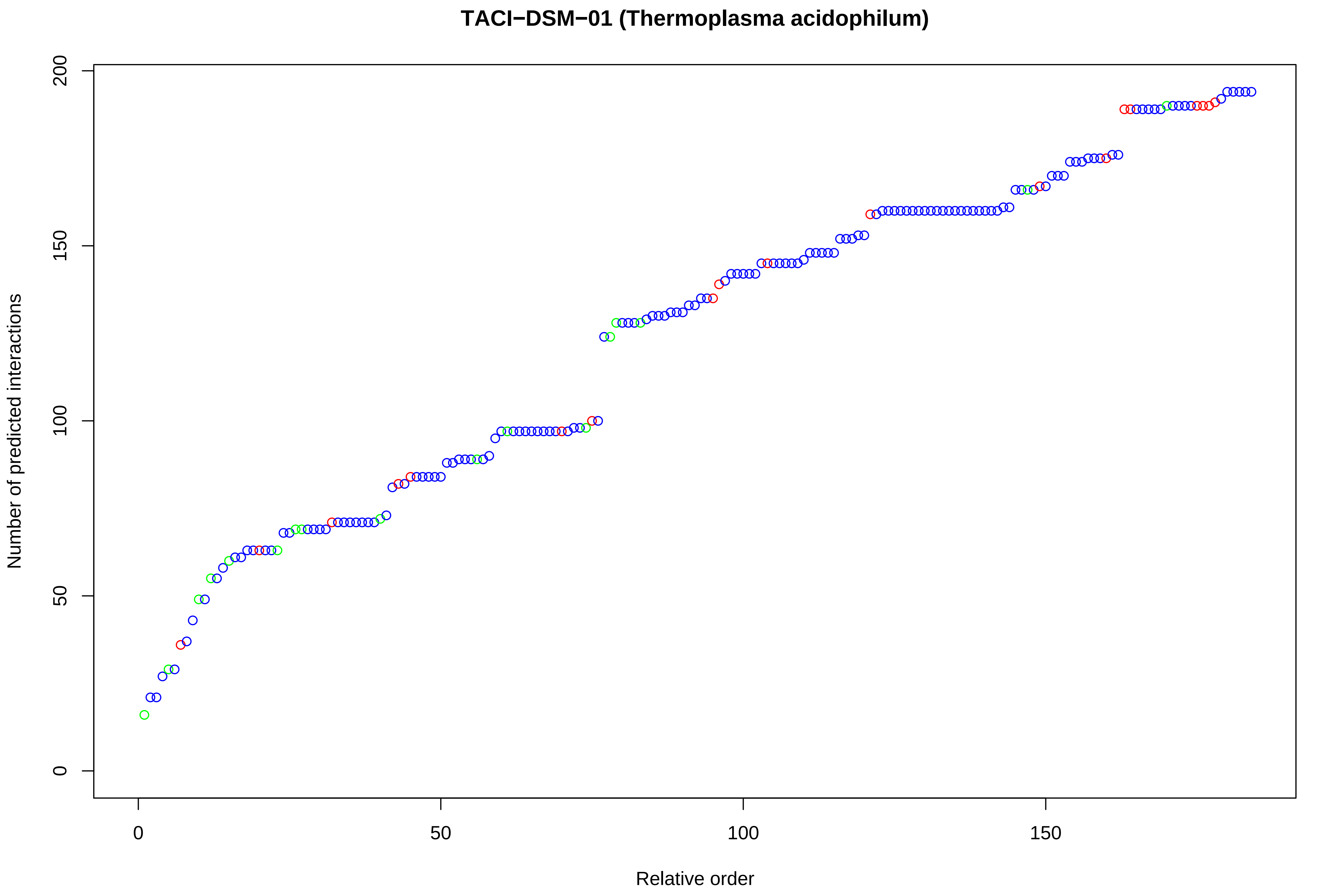

TACI−DSM−01 (Thermoplasma acidophilum)
Number of predicted interactions
Relative order
0
50
100
150
200

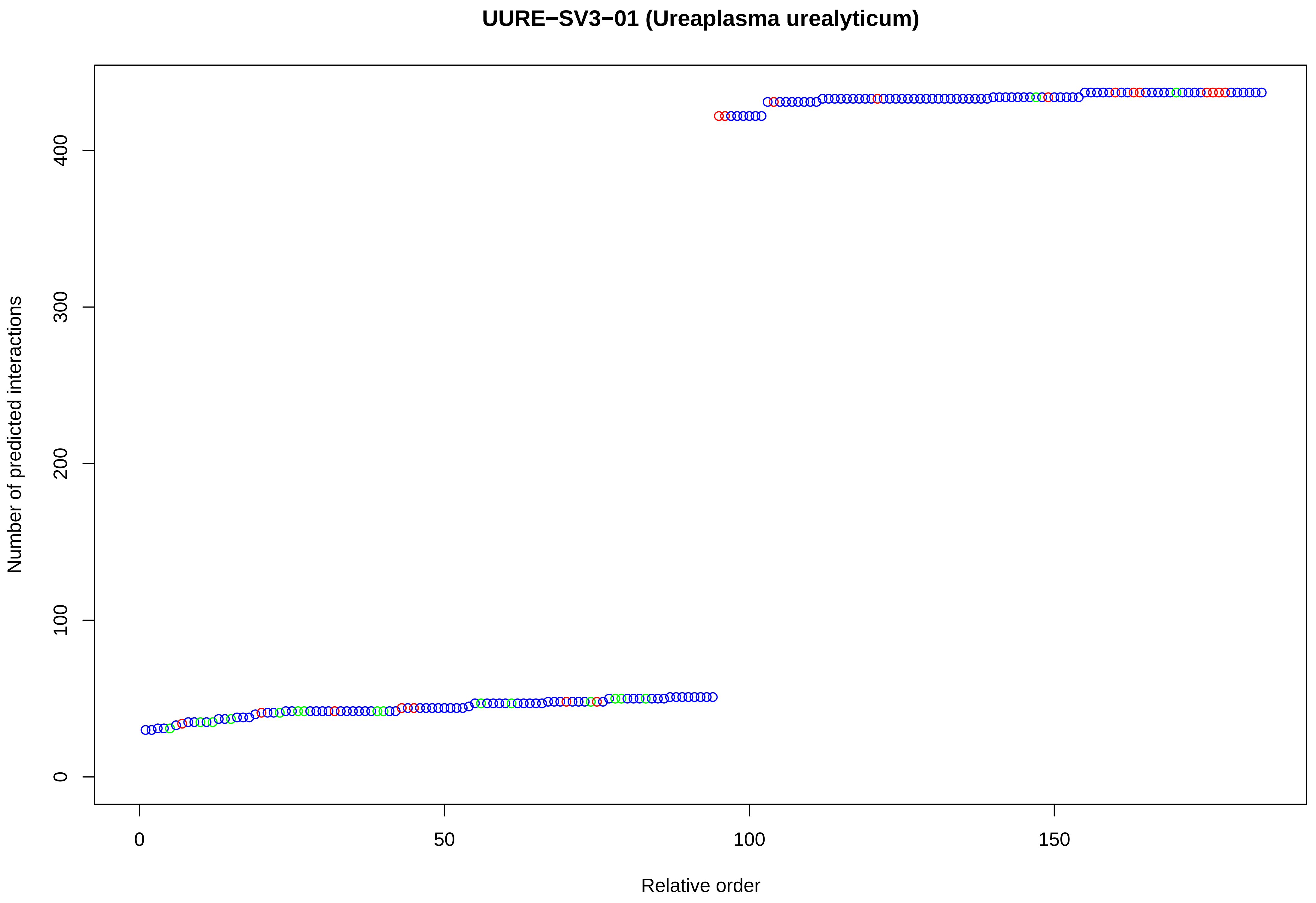
UURE−SV3−01 (Ureaplasma urealyticum)
Number of predicted interactions
0
100
200
300
400
Relative order
0
50
100
150

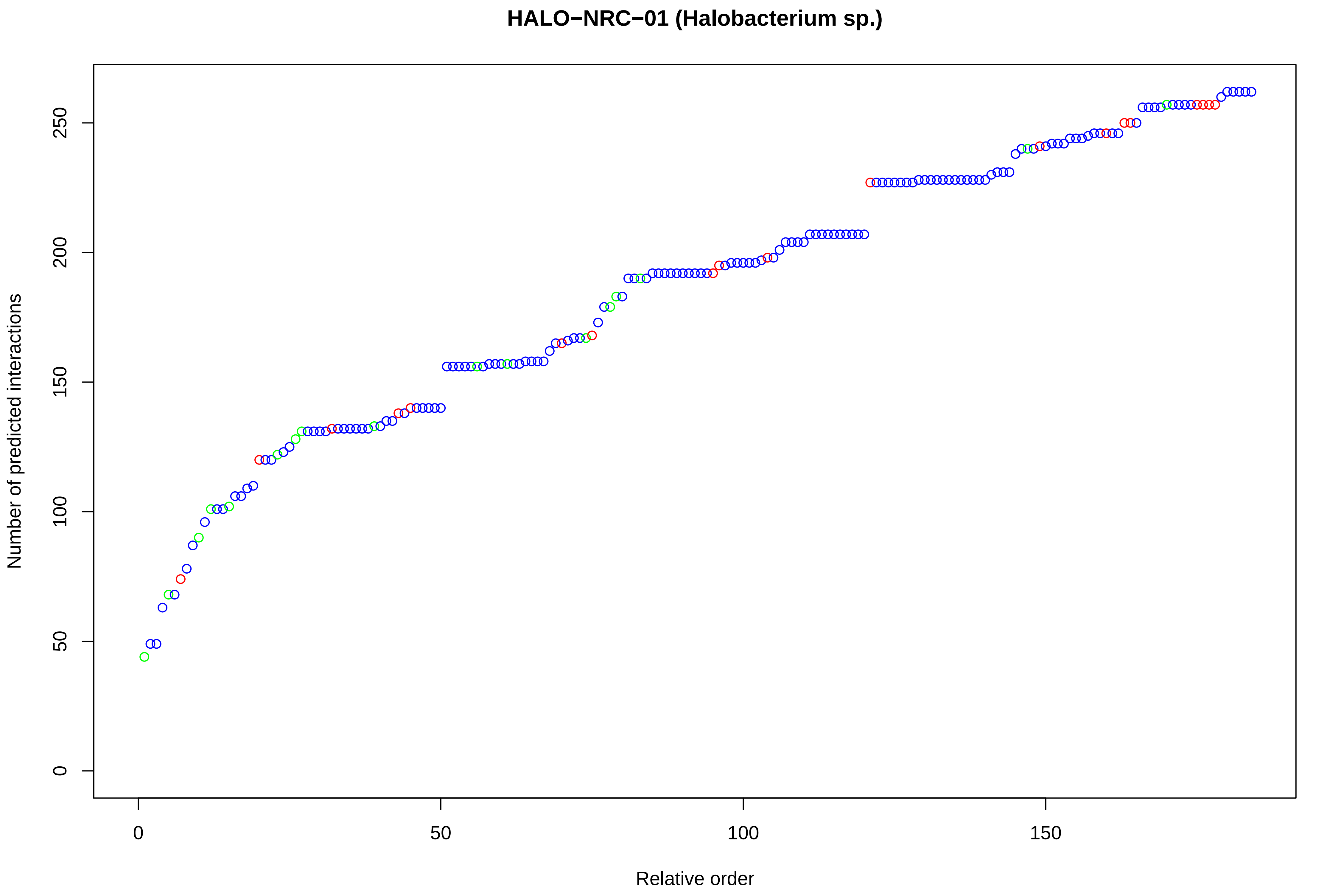

HALO−NRC−01 (Halobacterium sp.)
Number of predicted interactions
Relative order
0
50
100
150
200
250
0
50
100
150

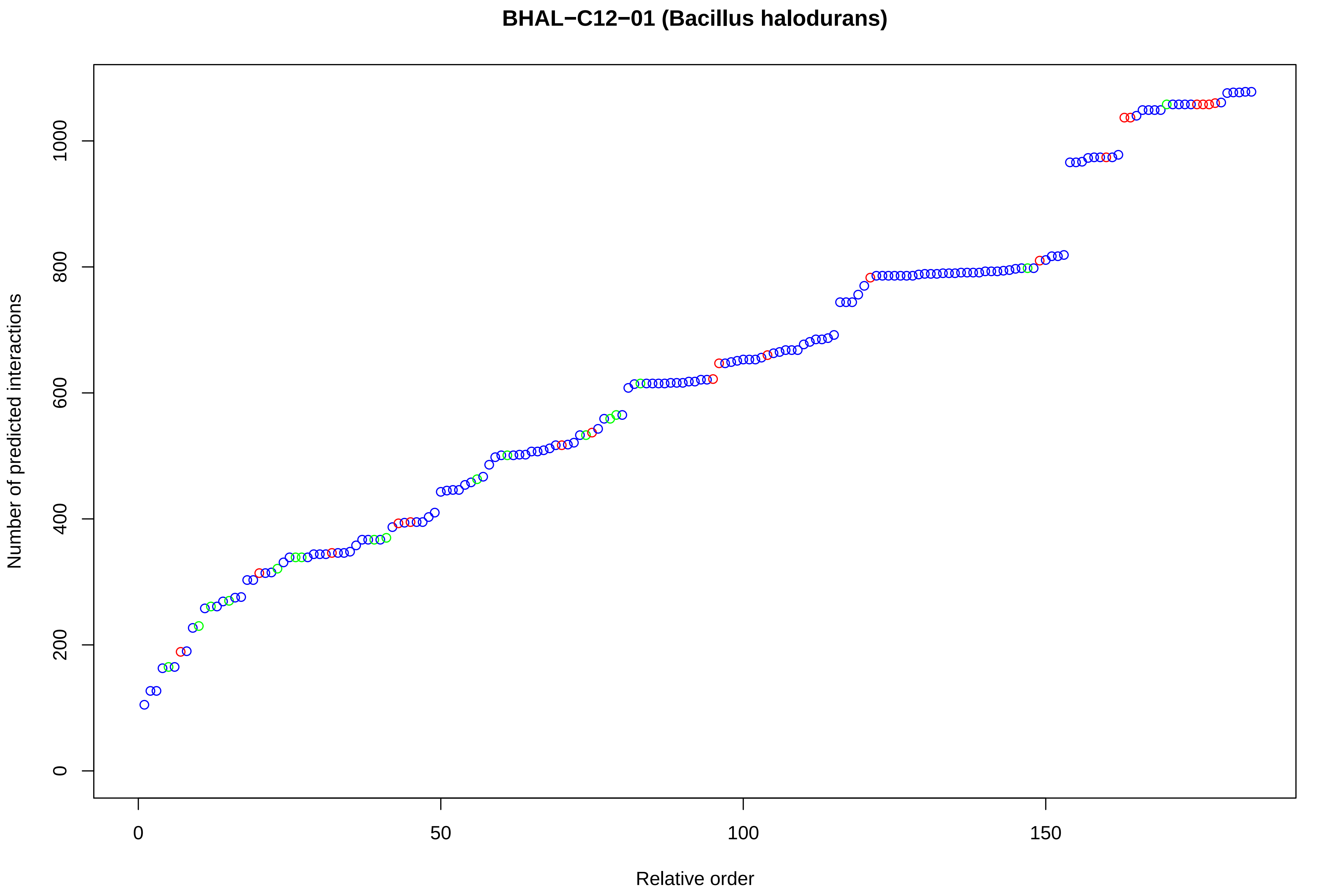

BHAL−C12−01 (Bacillus halodurans)
Number of predicted interactions
0
200
400
600
800
1000
0
50
100
150
Relative order

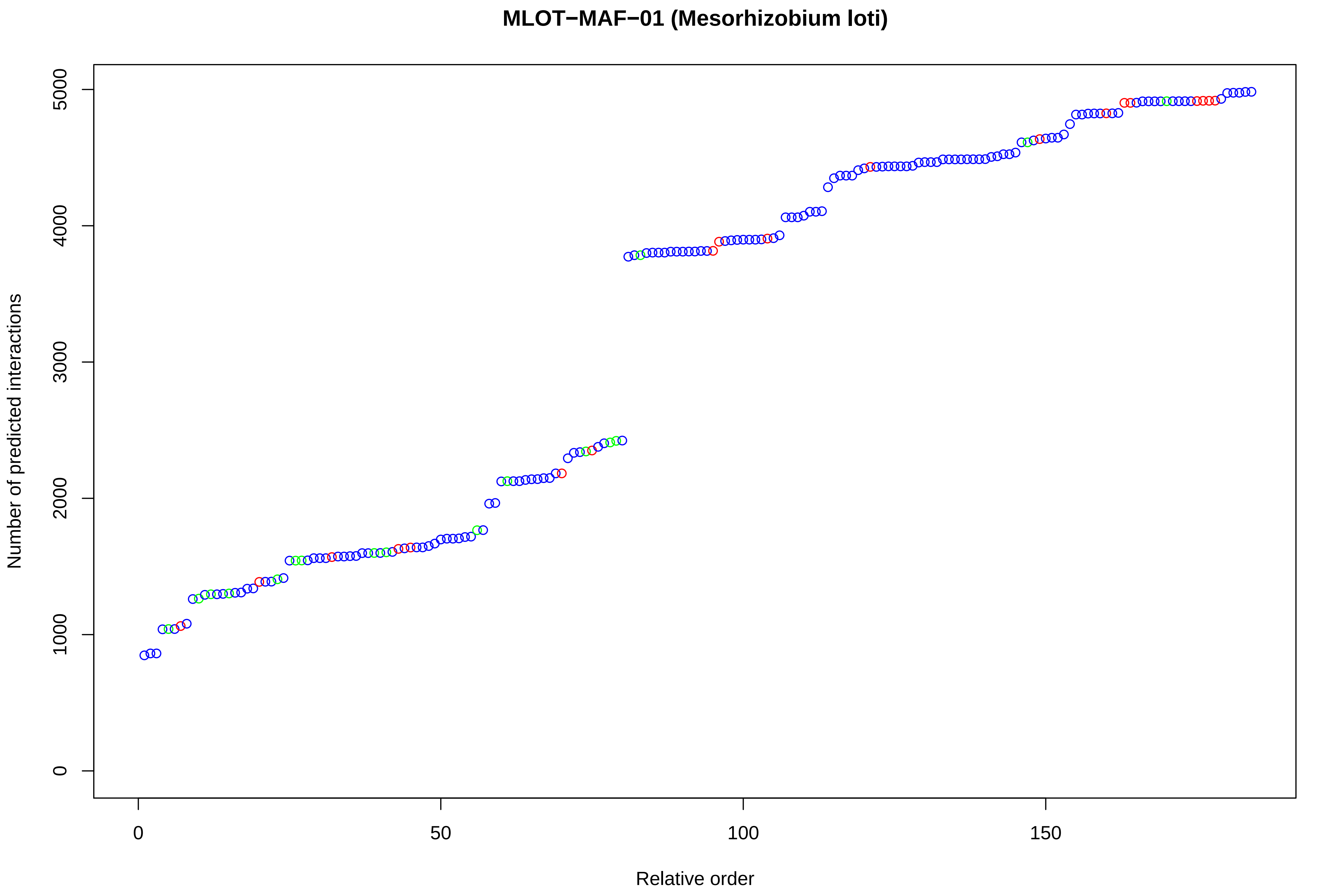

MLOT−MAF−01 (Mesorhizobium loti)
Number of predicted interactions
0
1000
2000
3000
4000
5000
Relative order
0
50
100
150

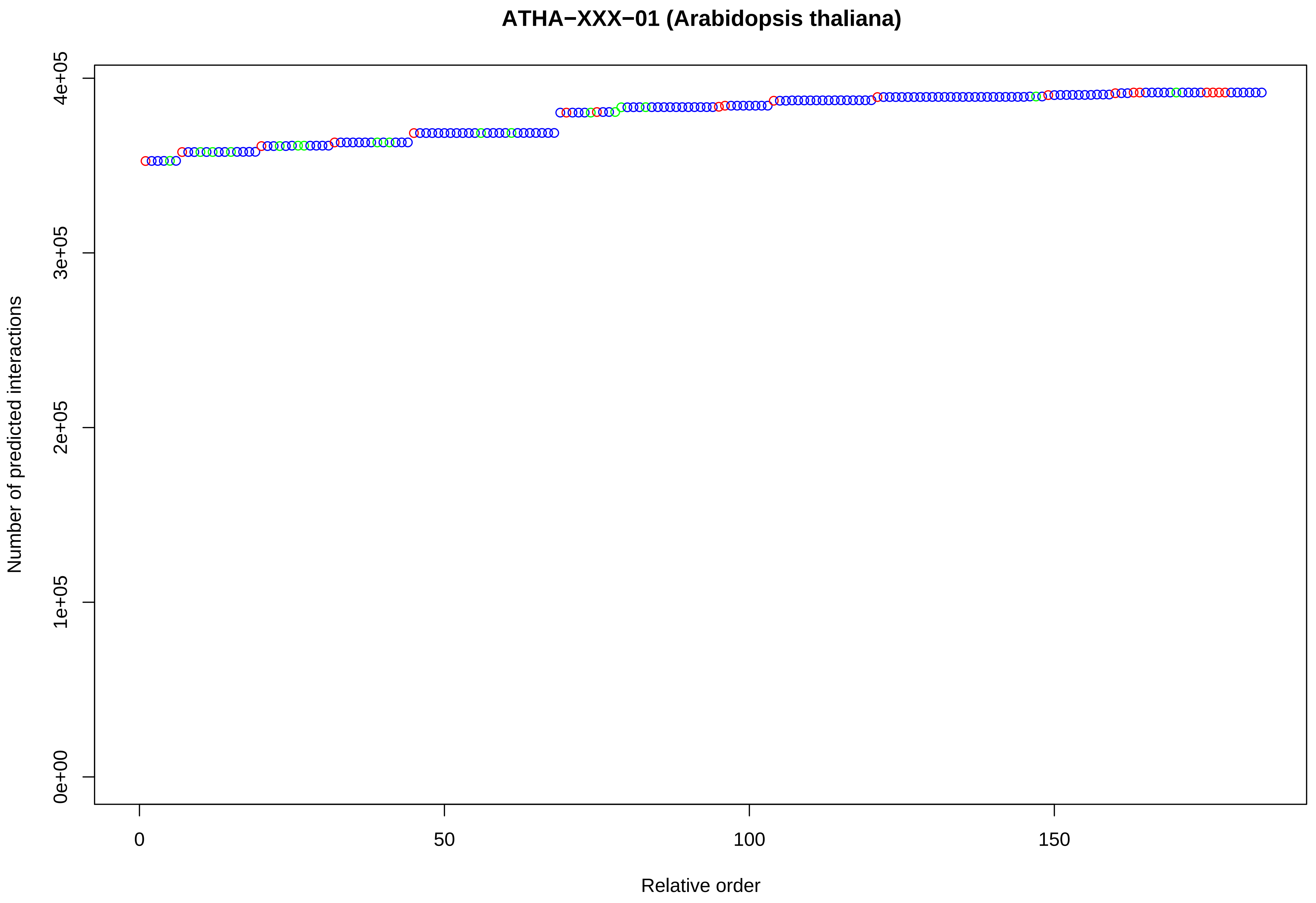

ATHA−XXX−01 (Arabidopsis thaliana)
Number of predicted interactions
0e+00
1e+05
2e+05
3e+05
4e+05
0
50
100
150
Relative order

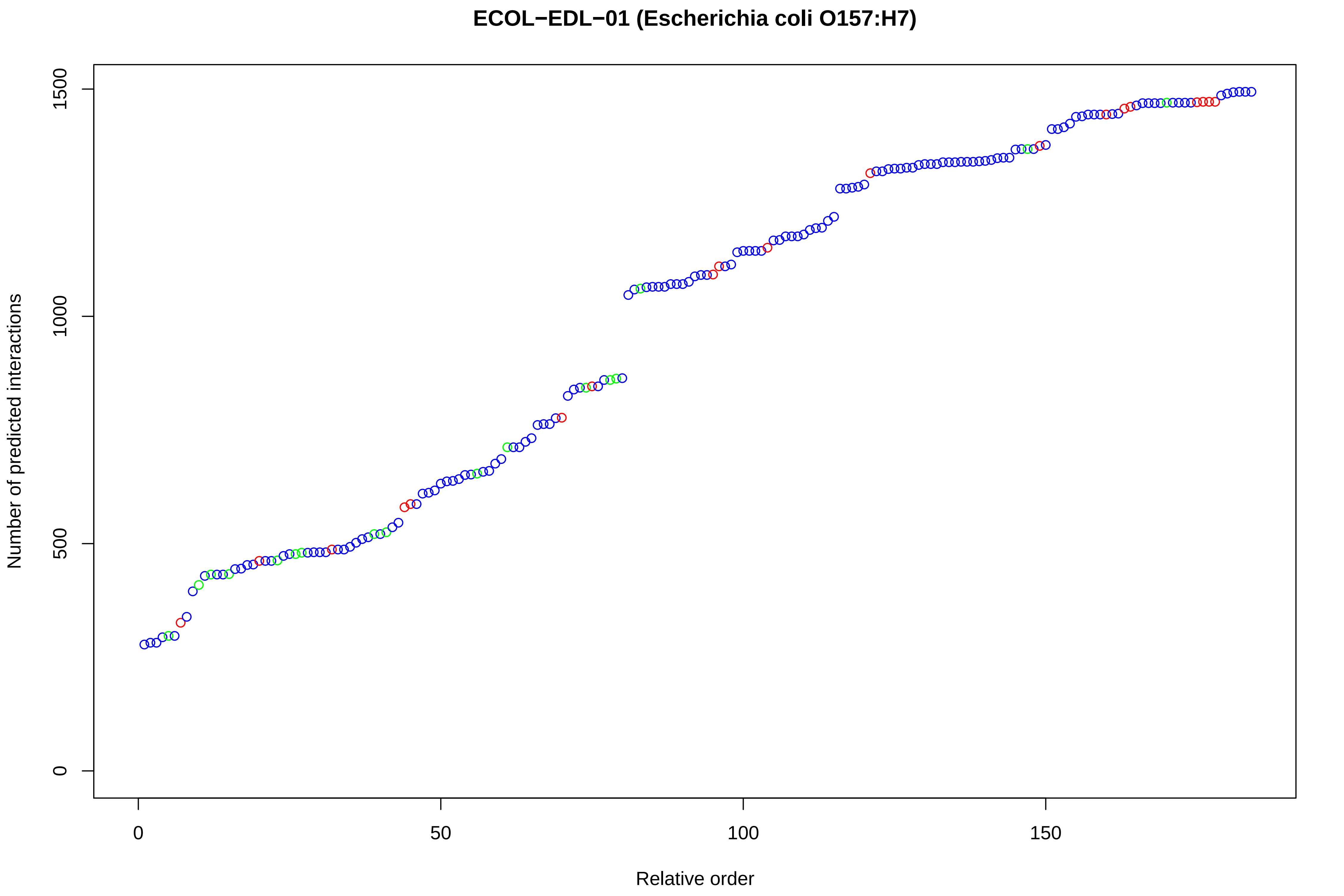
ECOL−EDL−01 (Escherichia coli O157:H7)
Number of predicted interactions
Relative order
0
500
1000
1500
0
50
100
150

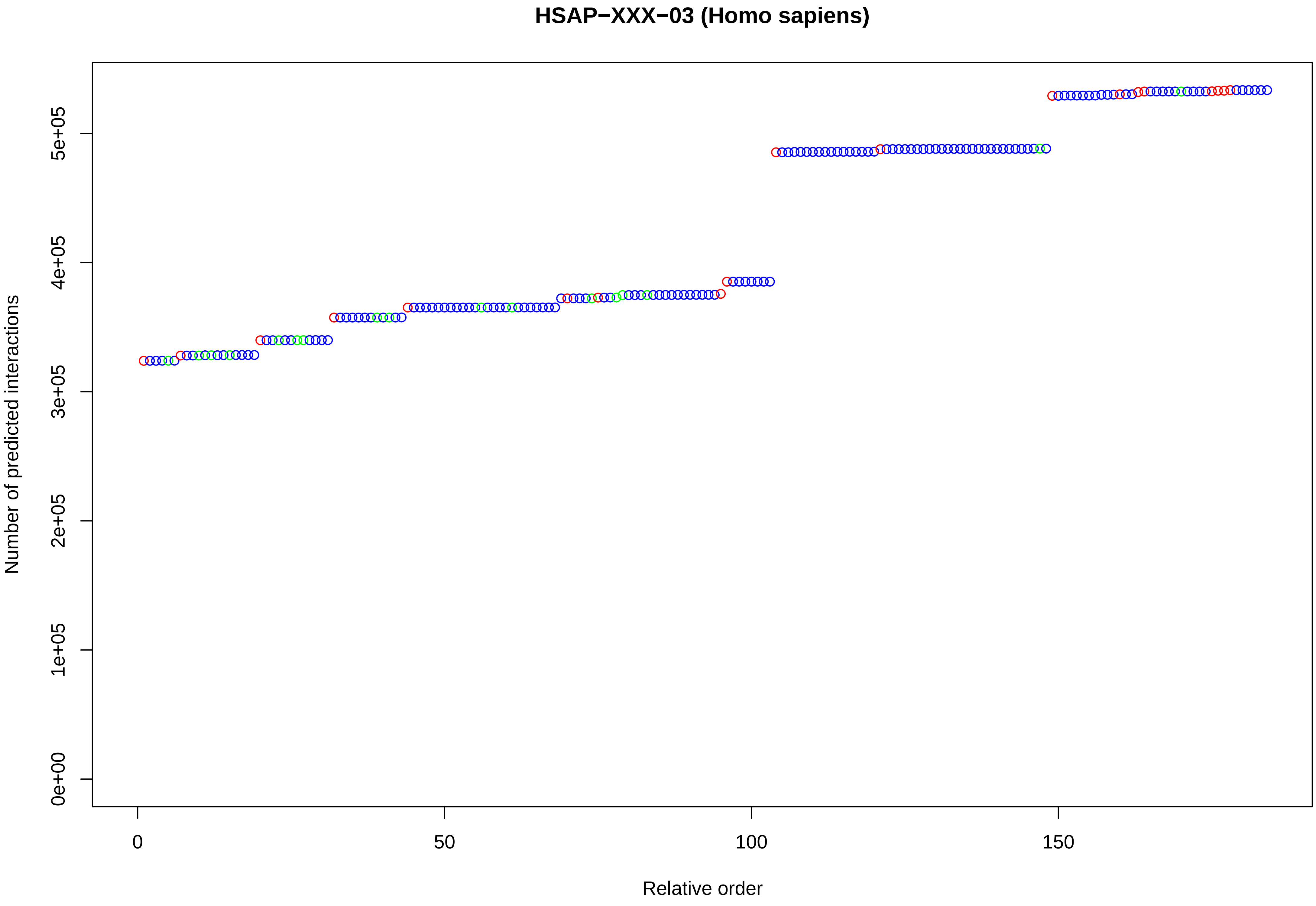

HSAP−XXX−03 (Homo sapiens)
Number of predicted interactions
0e+00
1e+05
2e+05
3e+05
4e+05
5e+05
0
50
100
150
Relative order

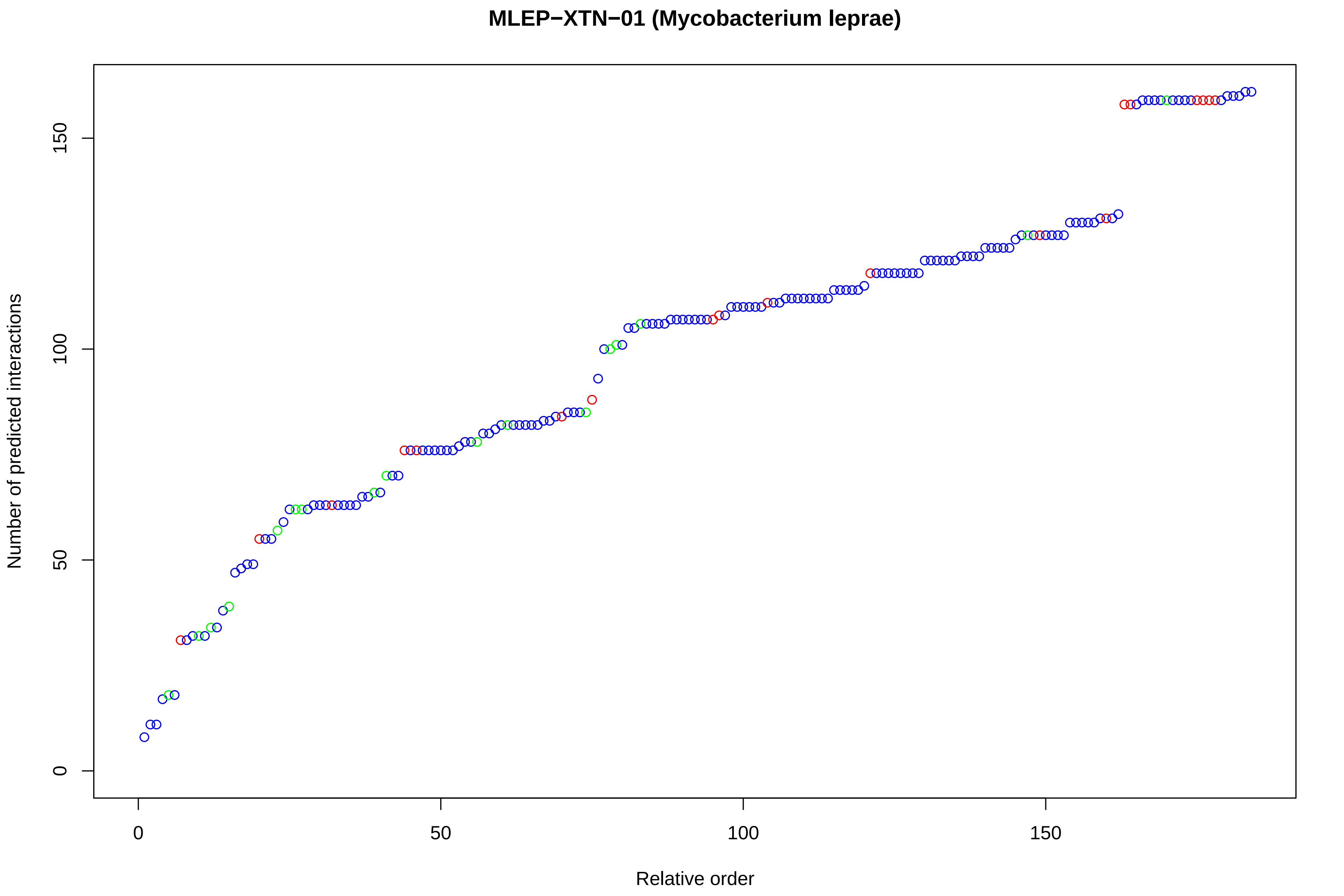
MLEP−XTN−01 (Mycobacterium leprae)
Number of predicted interactions
Relative order
0
50
100
150
0
50
100
150

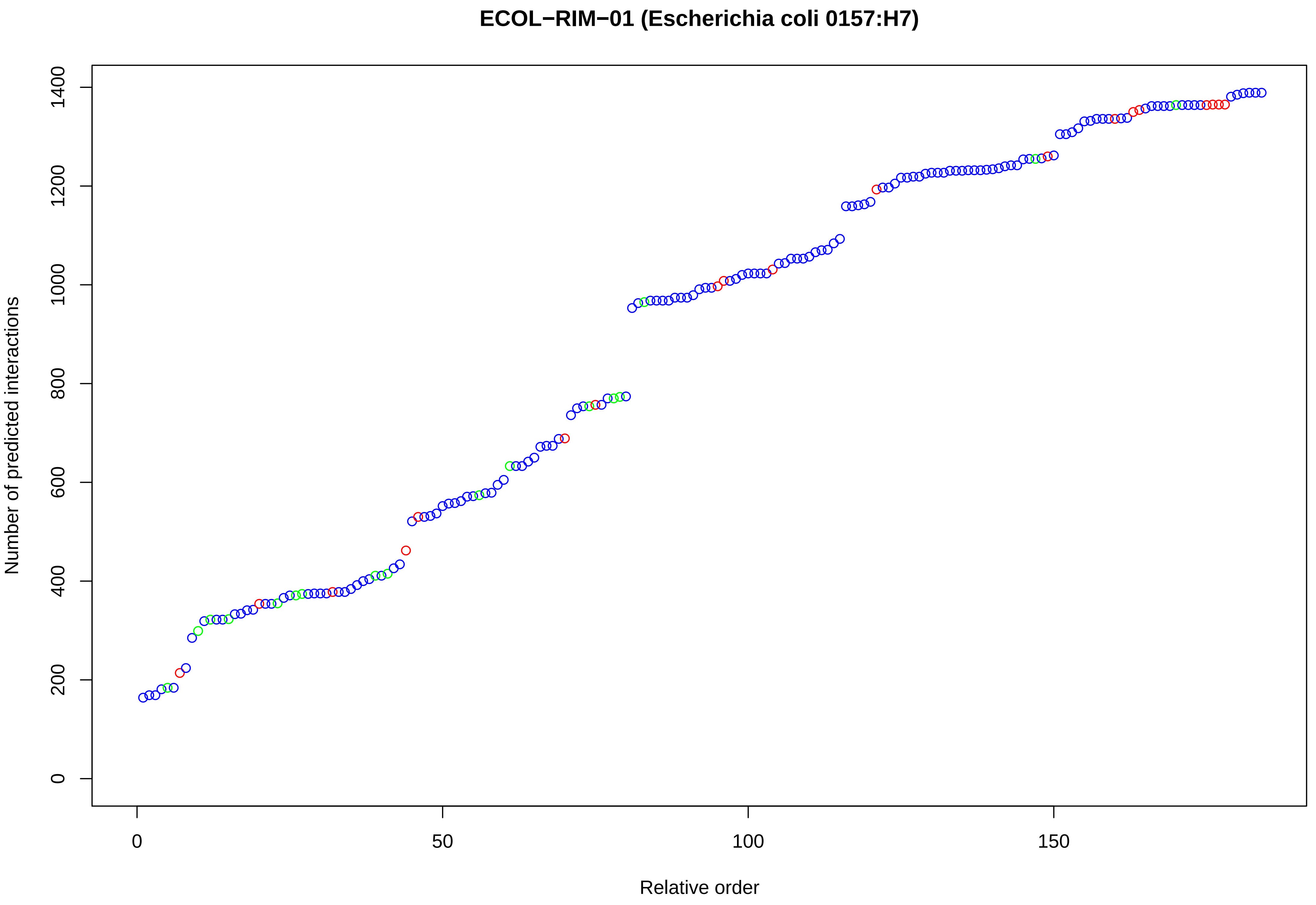
ECOL−RIM−01 (Escherichia coli 0157:H7)
Number of predicted interactions
0
200
400
600
800
1000
1200
1400
Relative order
0
50
100
150

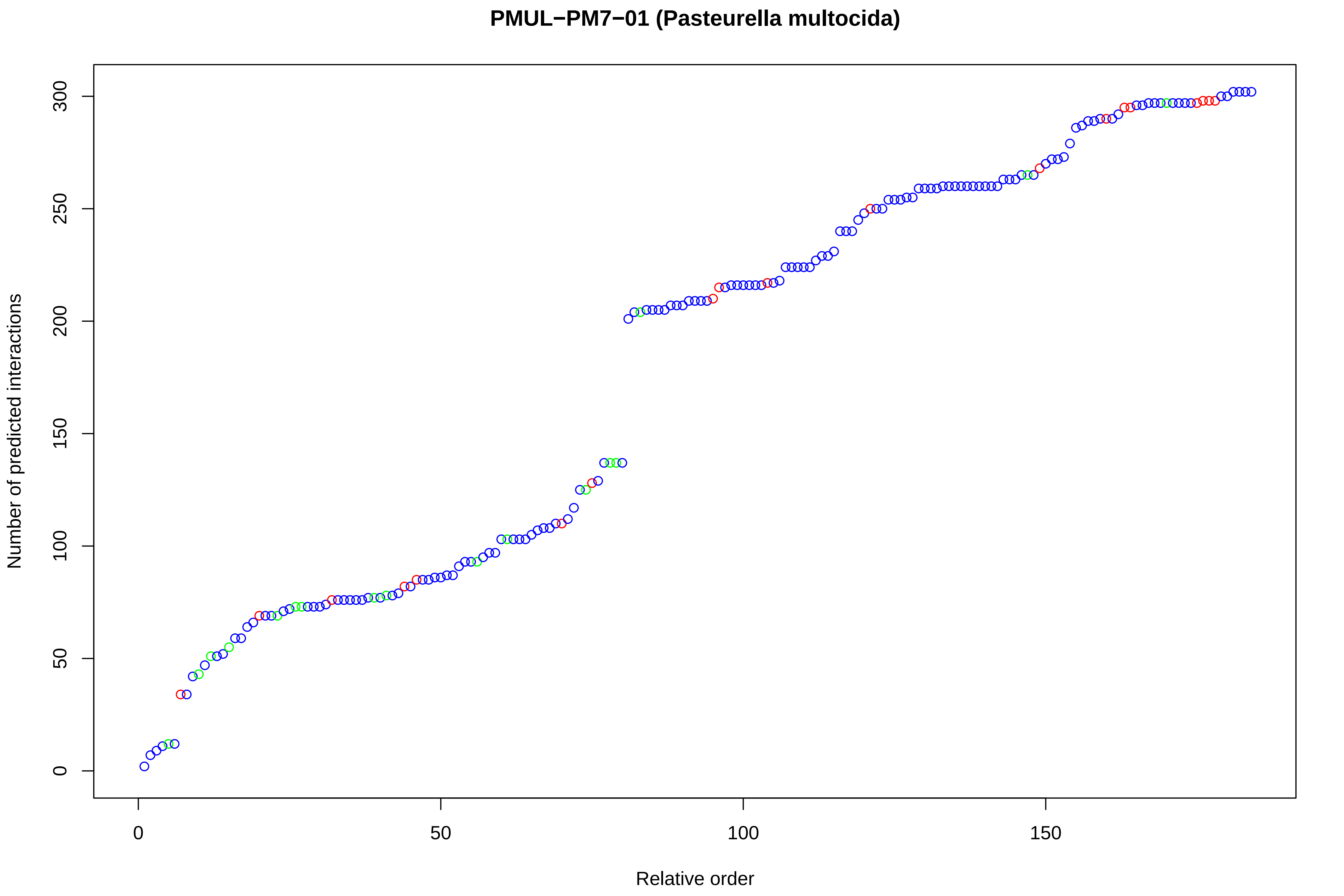

PMUL−PM7−01 (Pasteurella multocida)
Number of predicted interactions
0
50
100
150
200
250
300
Relative order
0
50
100
150

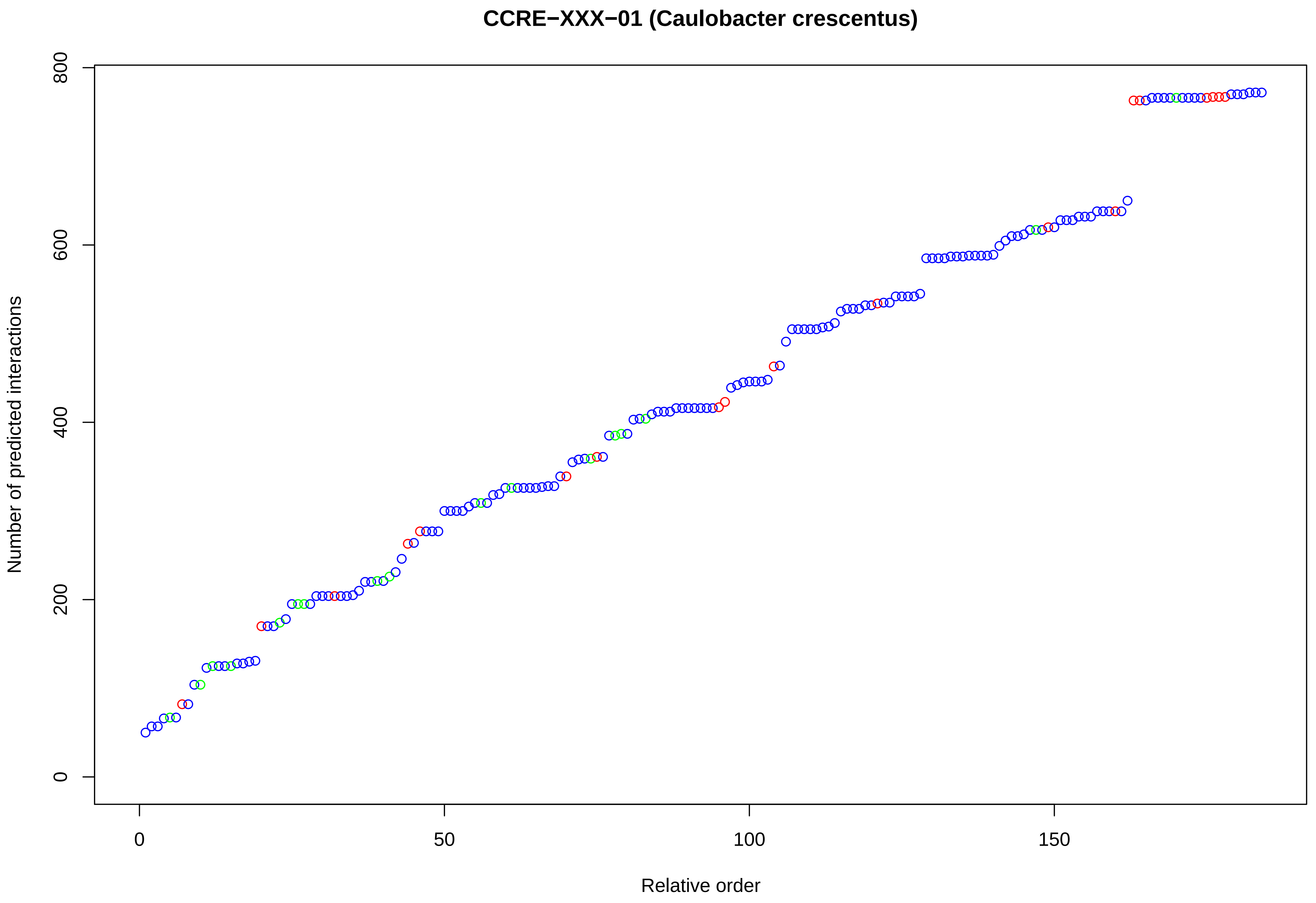
CCRE−XXX−01 (Caulobacter crescentus)
Number of predicted interactions
Relative order
0
200
400
600
800
0
50
100
150

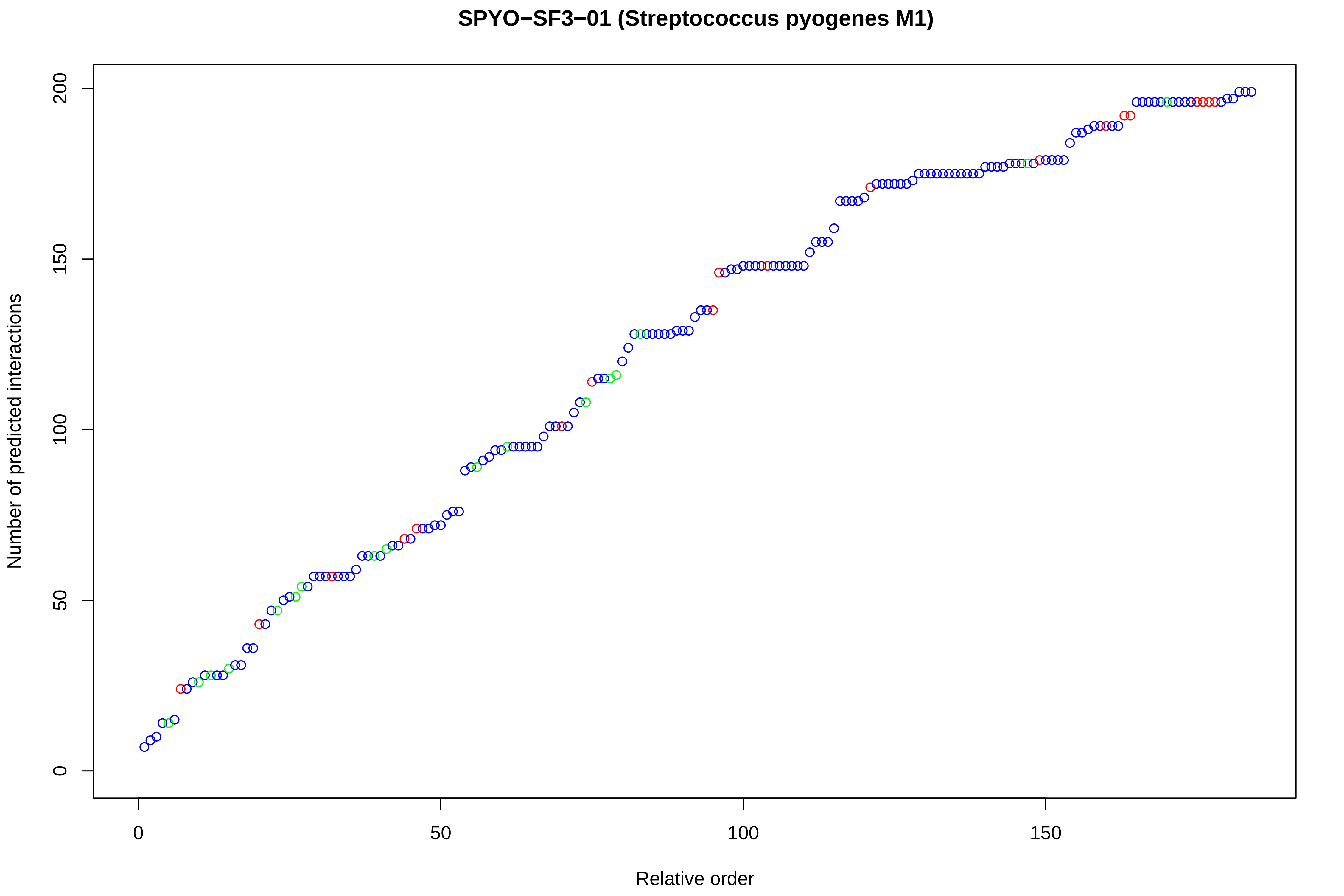

SPYO−SF3−01 (Streptococcus pyogenes M1)
Number of predicted interactions
Relative order
0
50
100
150
200

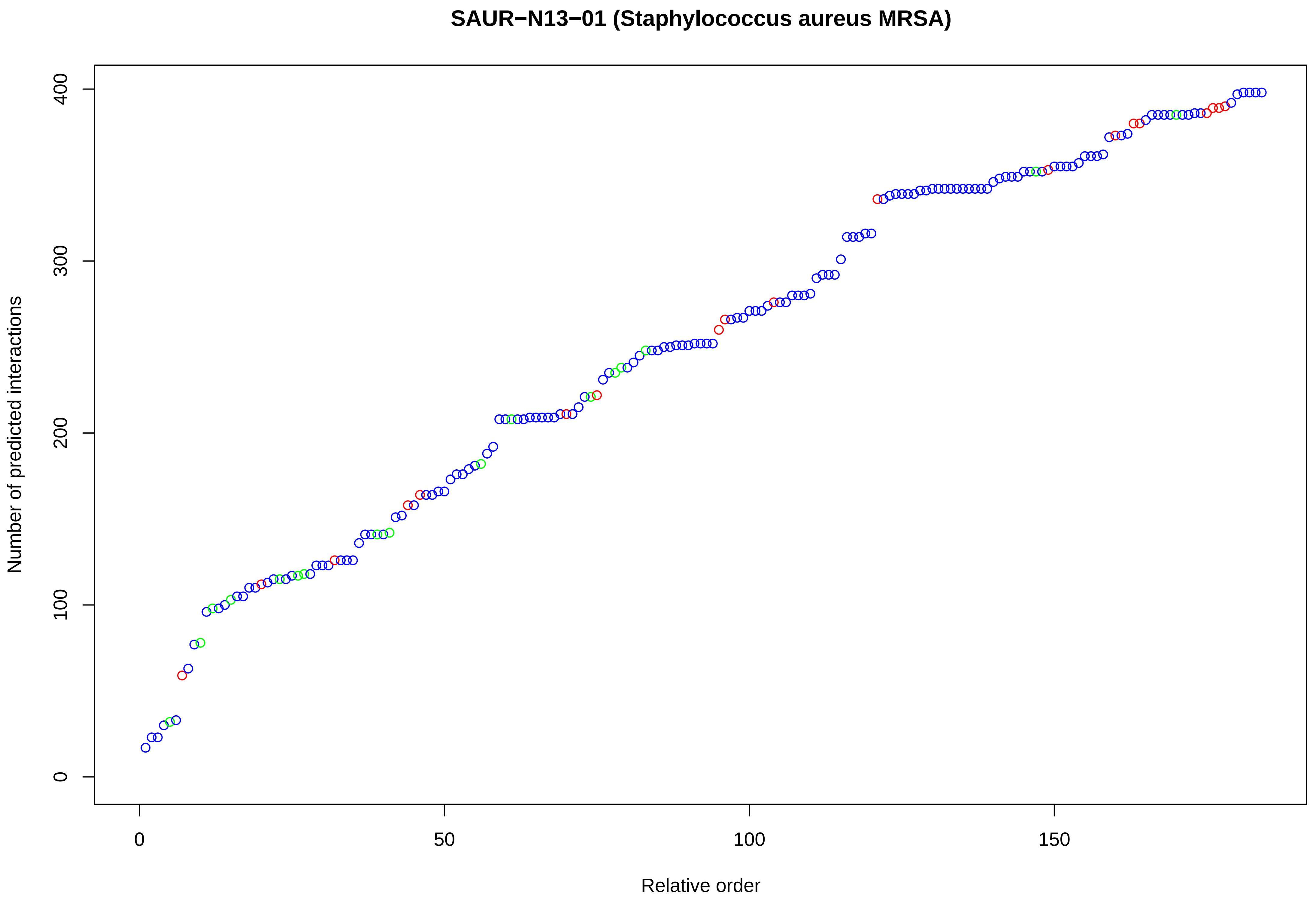
SAUR−N13−01 (Staphylococcus aureus MRSA)
Number of predicted interactions
Relative order
0
100
200
300
400
0
50
100
150

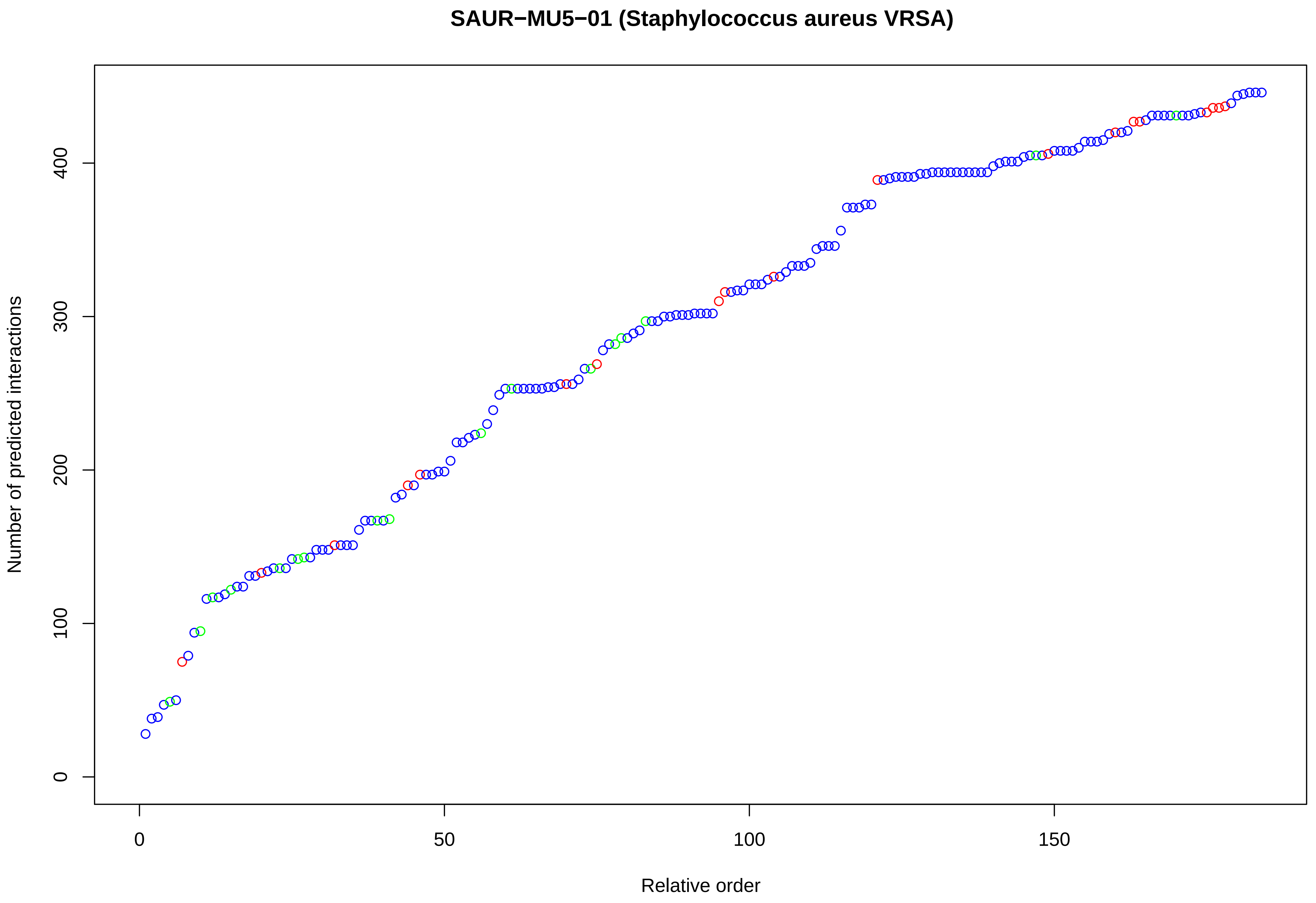

SAUR−MU5−01 (Staphylococcus aureus VRSA)
Number of predicted interactions
0
100
200
300
400
0
50
100
150
Relative order

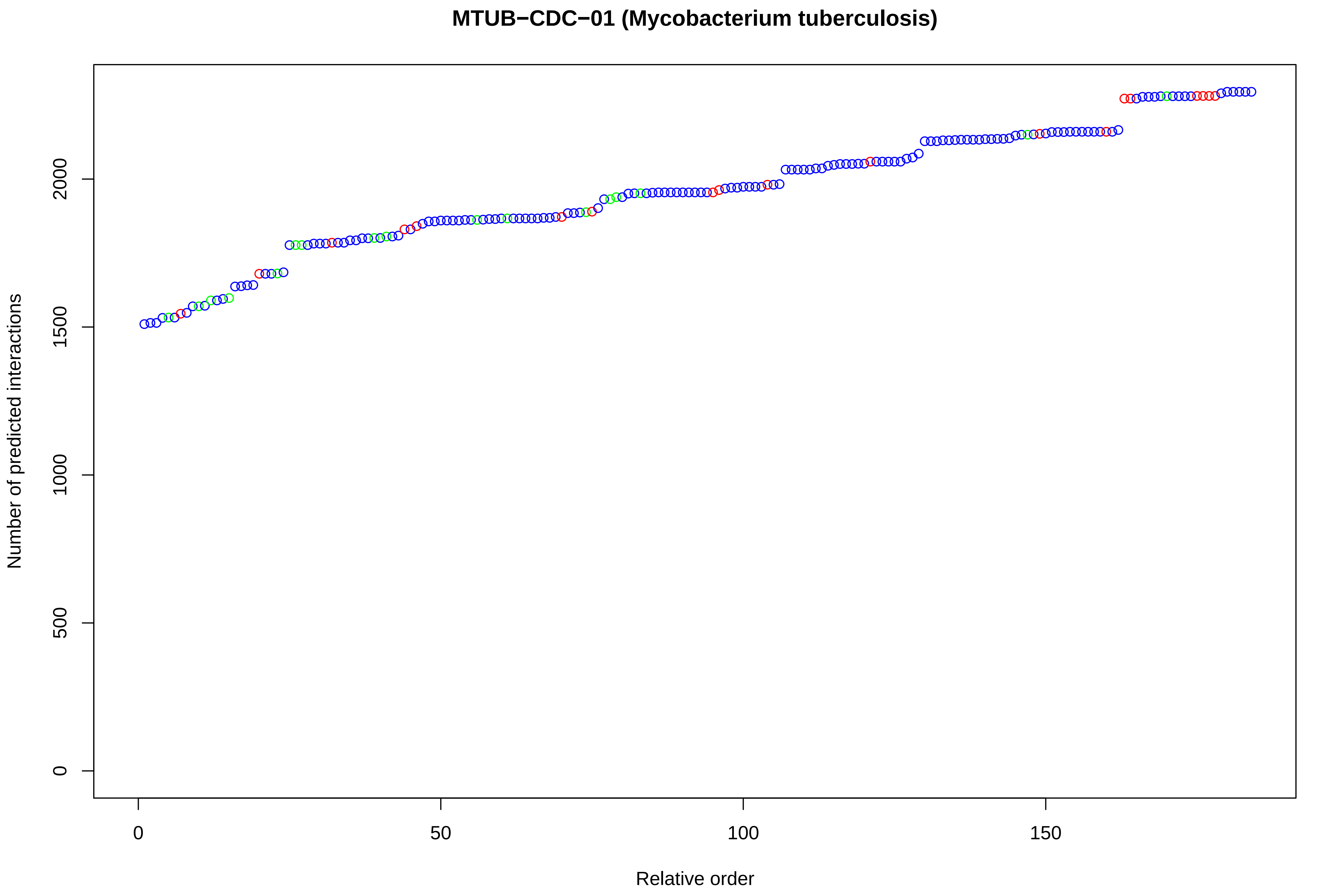
MTUB−CDC−01 (Mycobacterium tuberculosis)
Number of predicted interactions
0
500
1000
1500
2000
0
50
100
150
Relative order

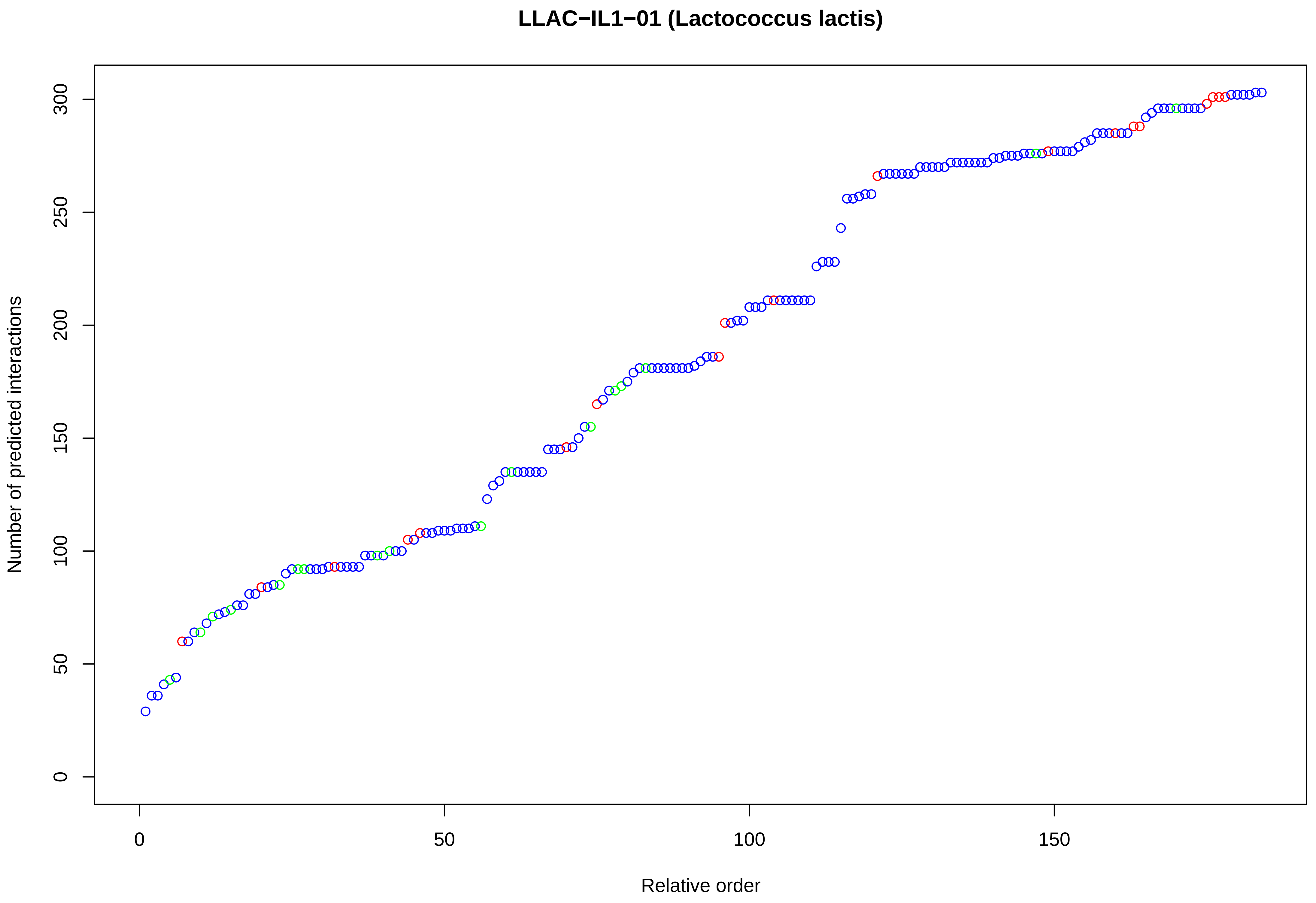

LLAC−IL1−01 (Lactococcus lactis)
Number of predicted interactions
0
50
100
150
200
250
300
Relative order
0
50
100
150

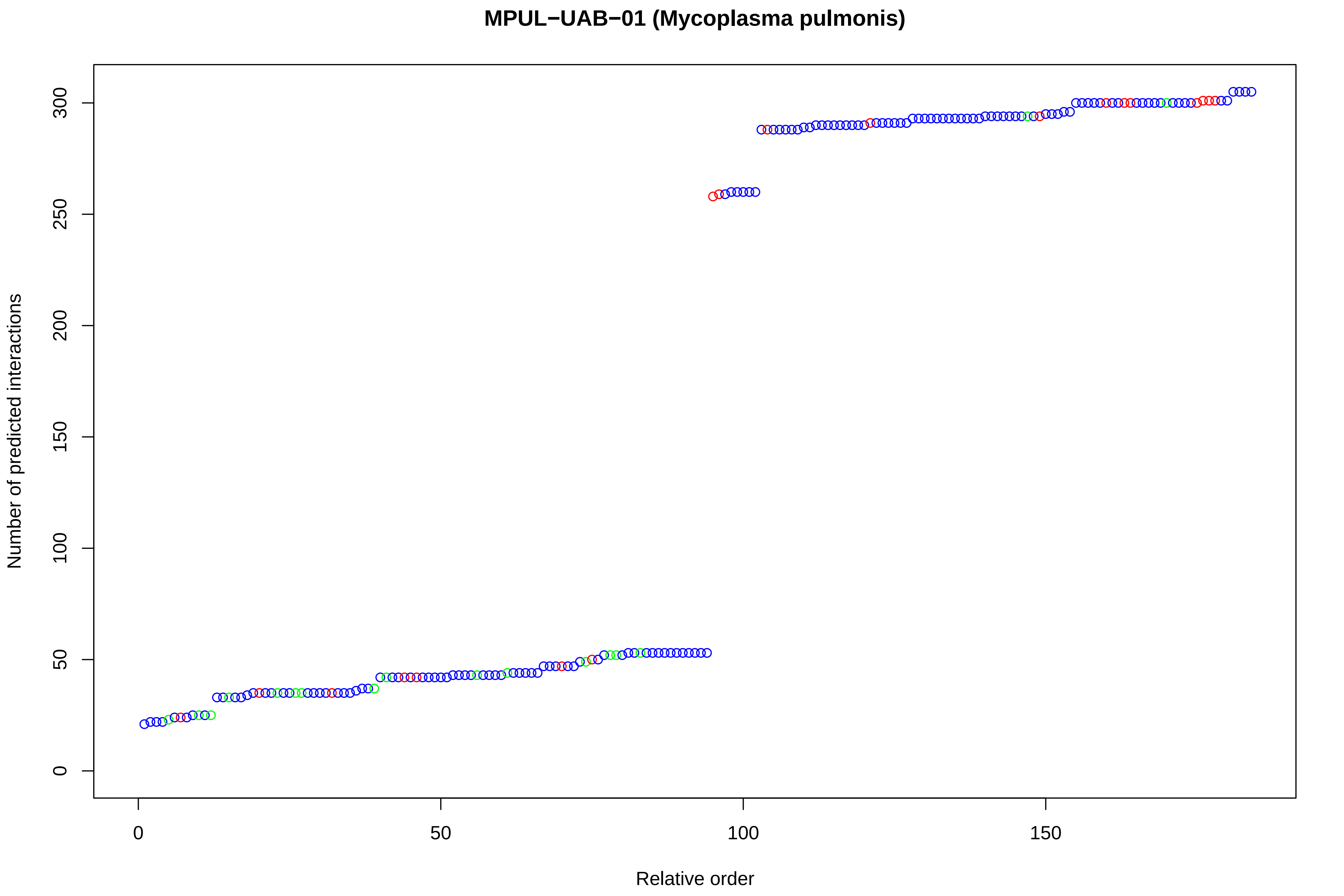

MPUL−UAB−01 (Mycoplasma pulmonis)
Number of predicted interactions
0
50
100
150
200
250
300
Relative order
0
50
100
150

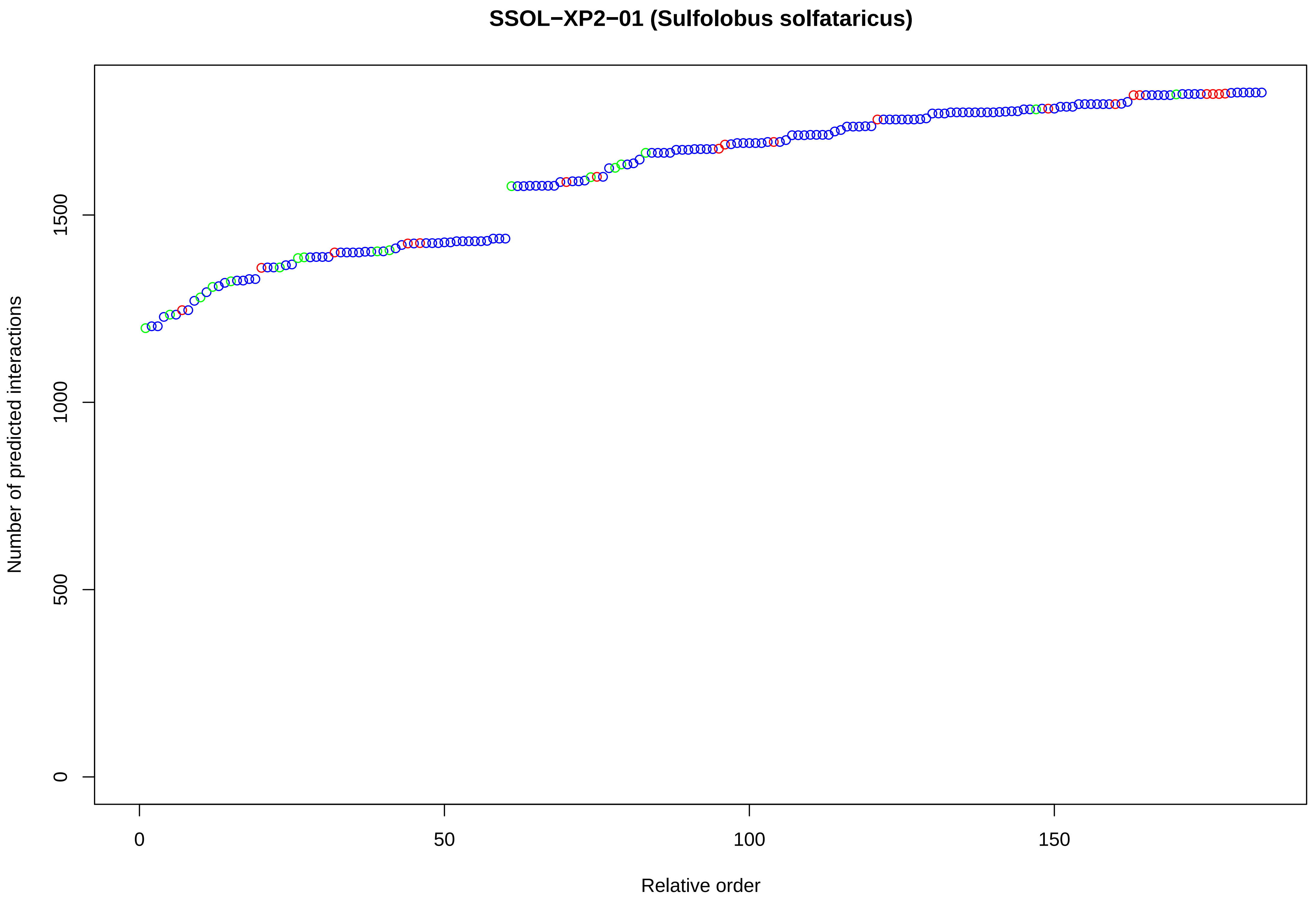
SSOL−XP2−01 (Sulfolobus solfataricus)
Number of predicted interactions
0
500
1000
1500
0
50
100
150
Relative order

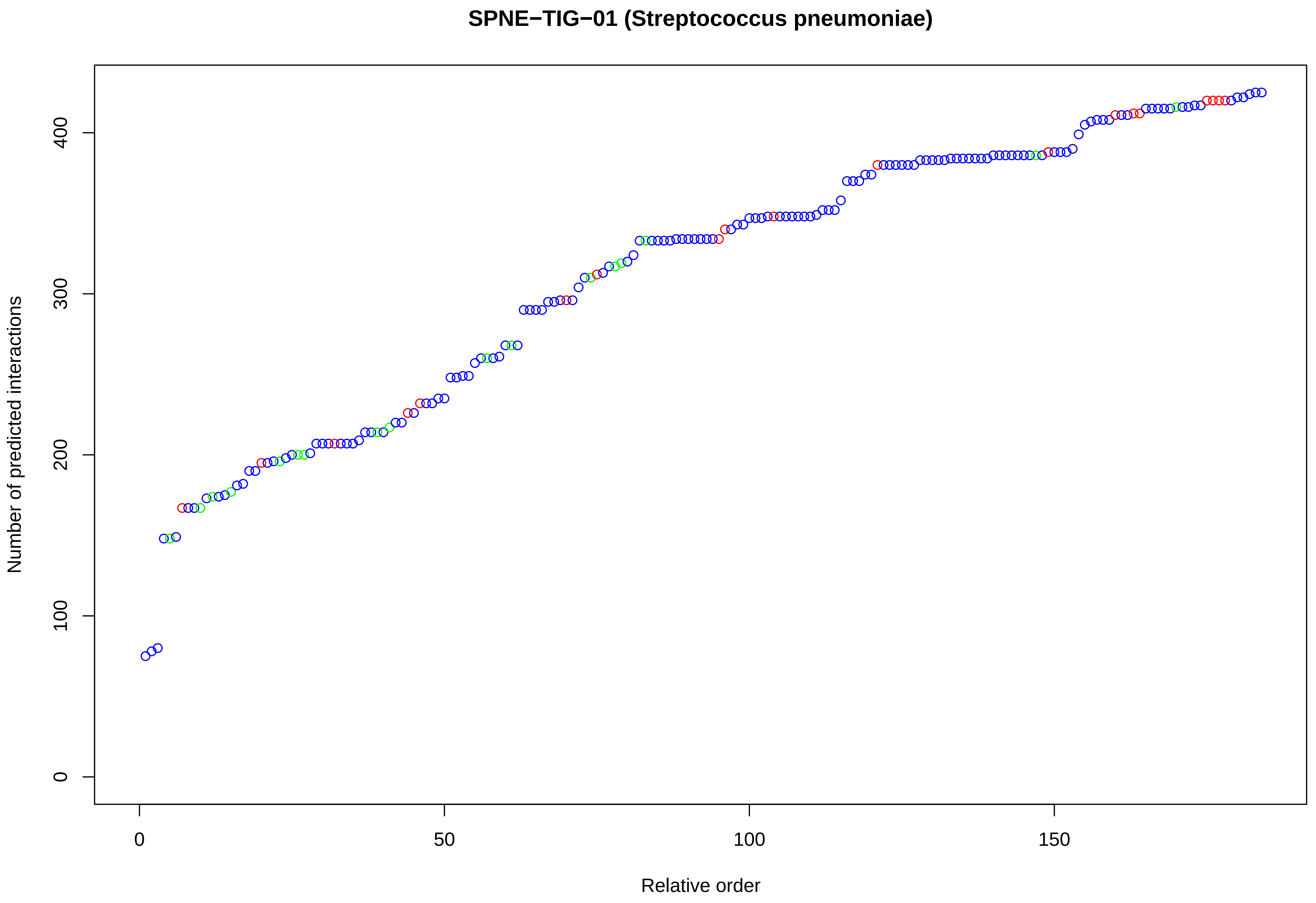
SPNE−TIG−01 (Streptococcus pneumoniae)
Number of predicted interactions
Relative order
0
100
200
300
400
0
50
100
150

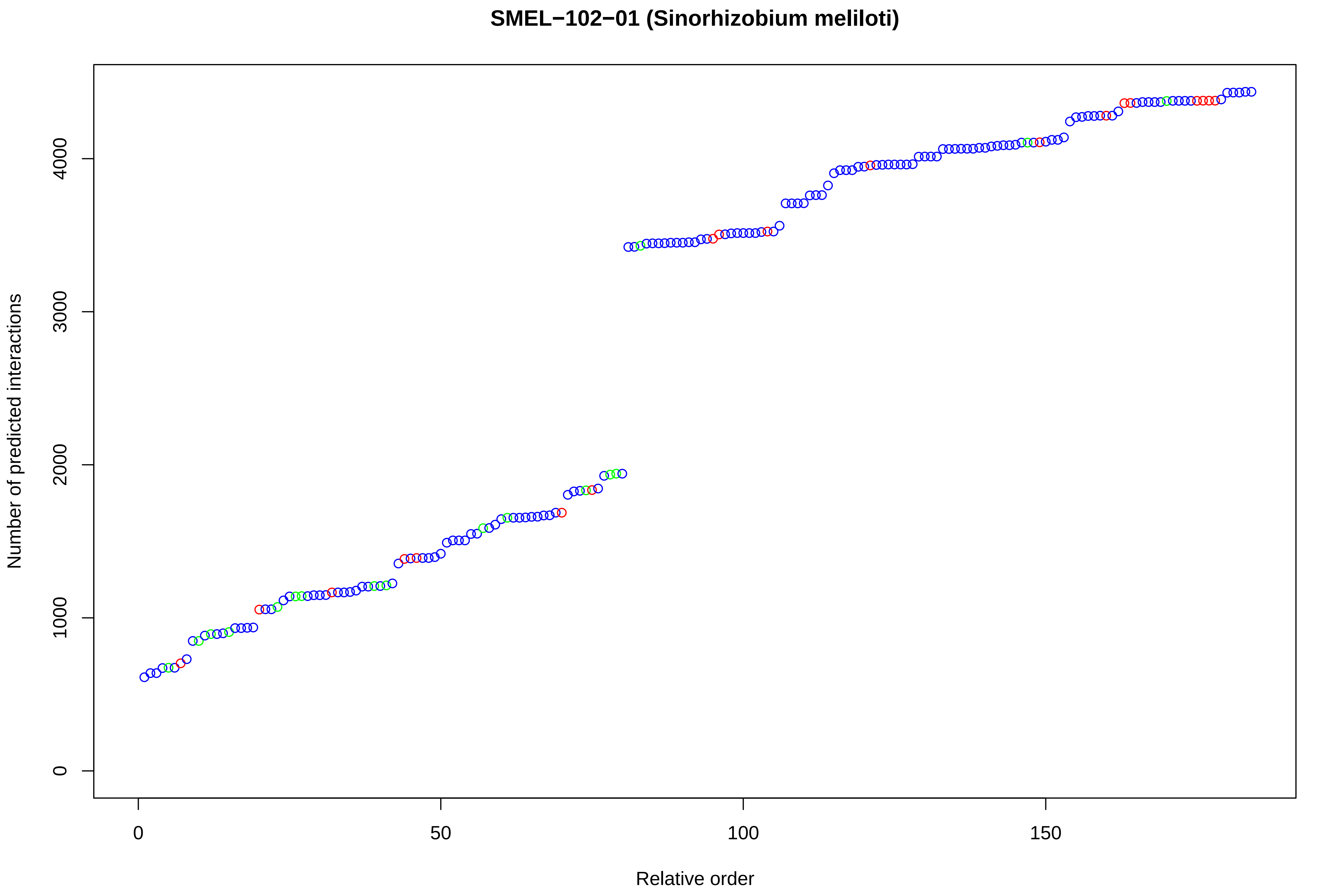

SMEL−102−01 (Sinorhizobium meliloti)
Number of predicted interactions
Relative order
0
1000
2000
3000
4000
0
50
100
150

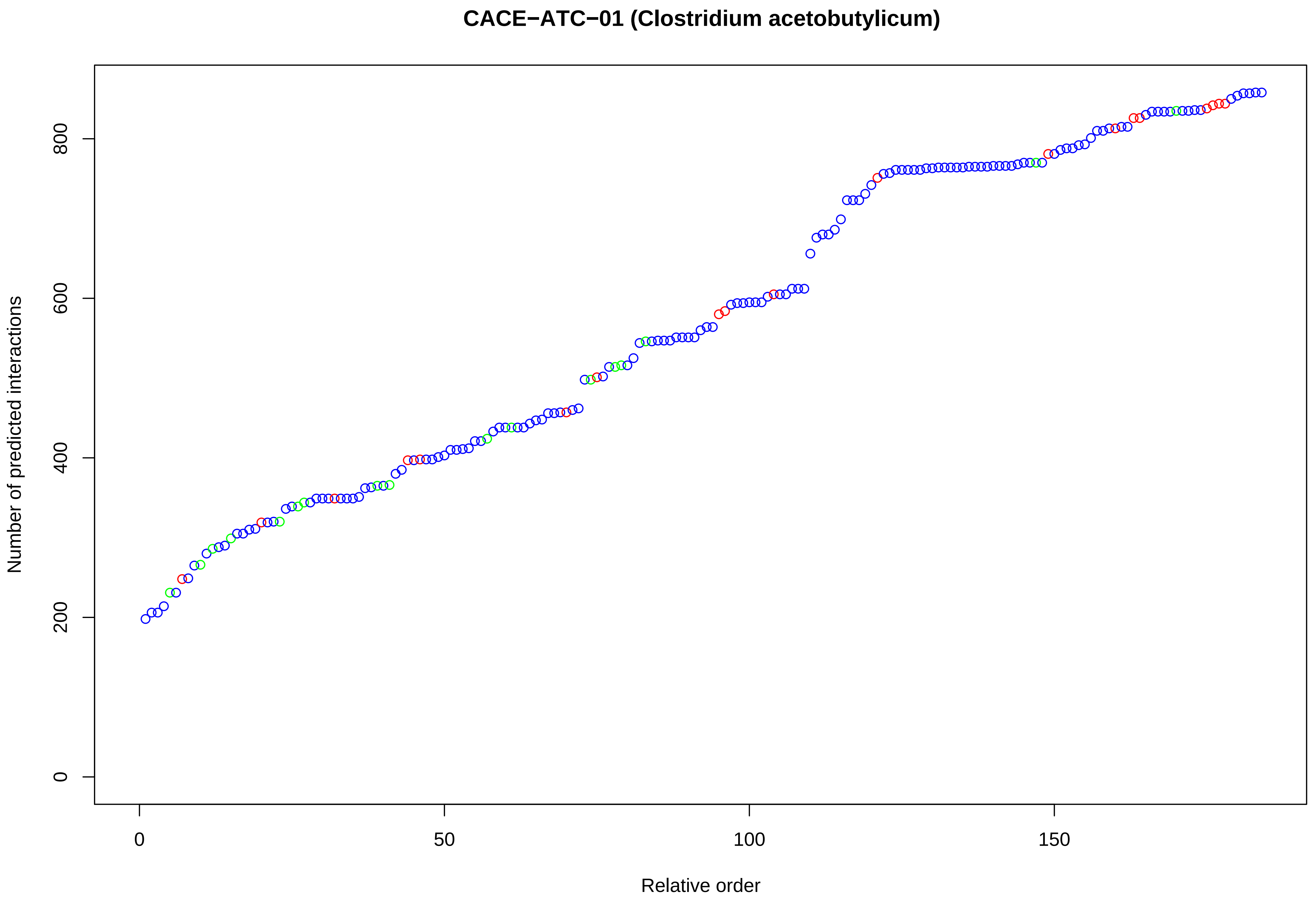
CACE−ATC−01 (Clostridium acetobutylicum)
Number of predicted interactions
0
200
400
600
800
0
50
100
150
Relative order

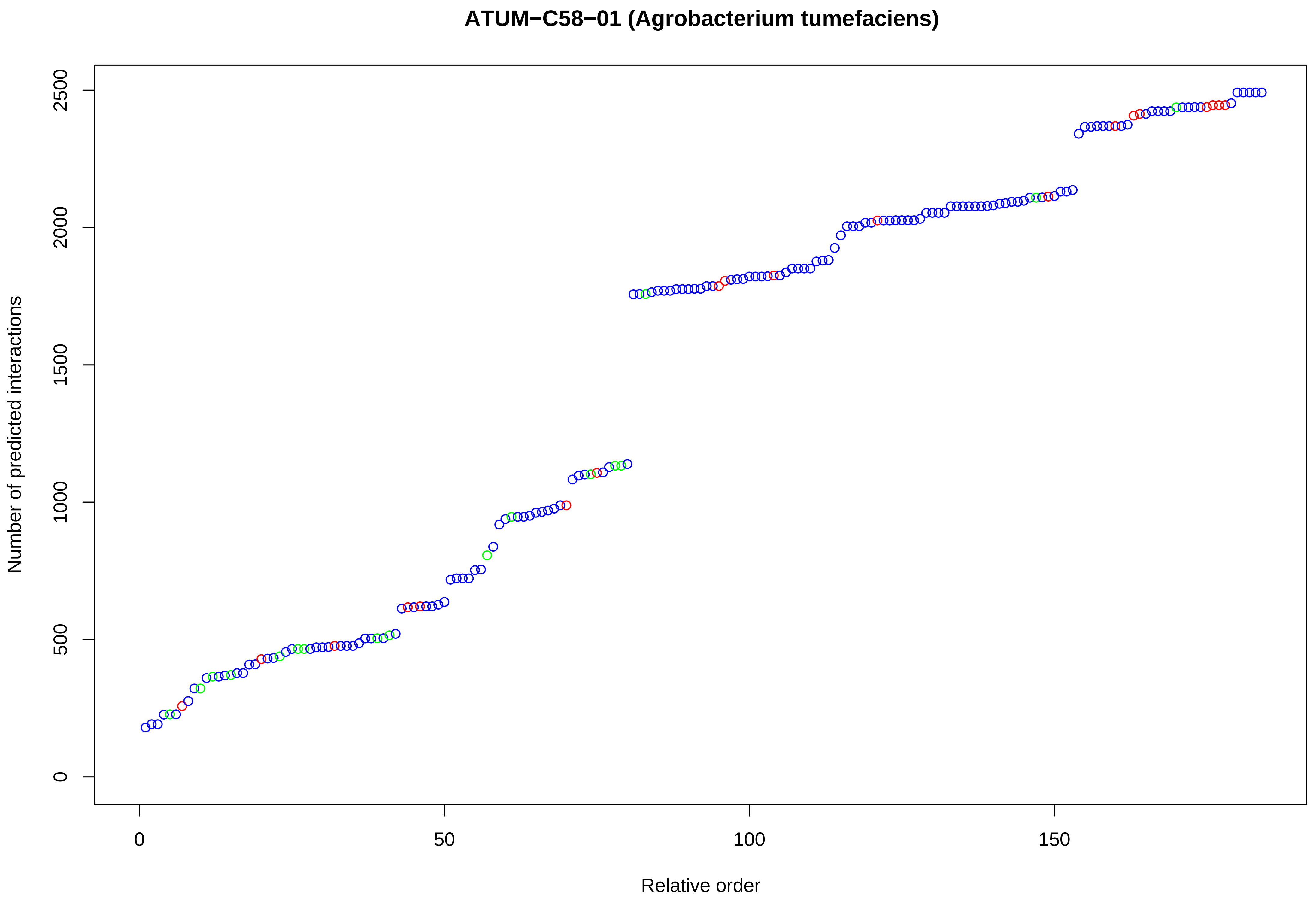
ATUM−C58−01 (Agrobacterium tumefaciens)
Number of predicted interactions
Relative order
0
500
1000
1500
2000
2500
0
50
100
150

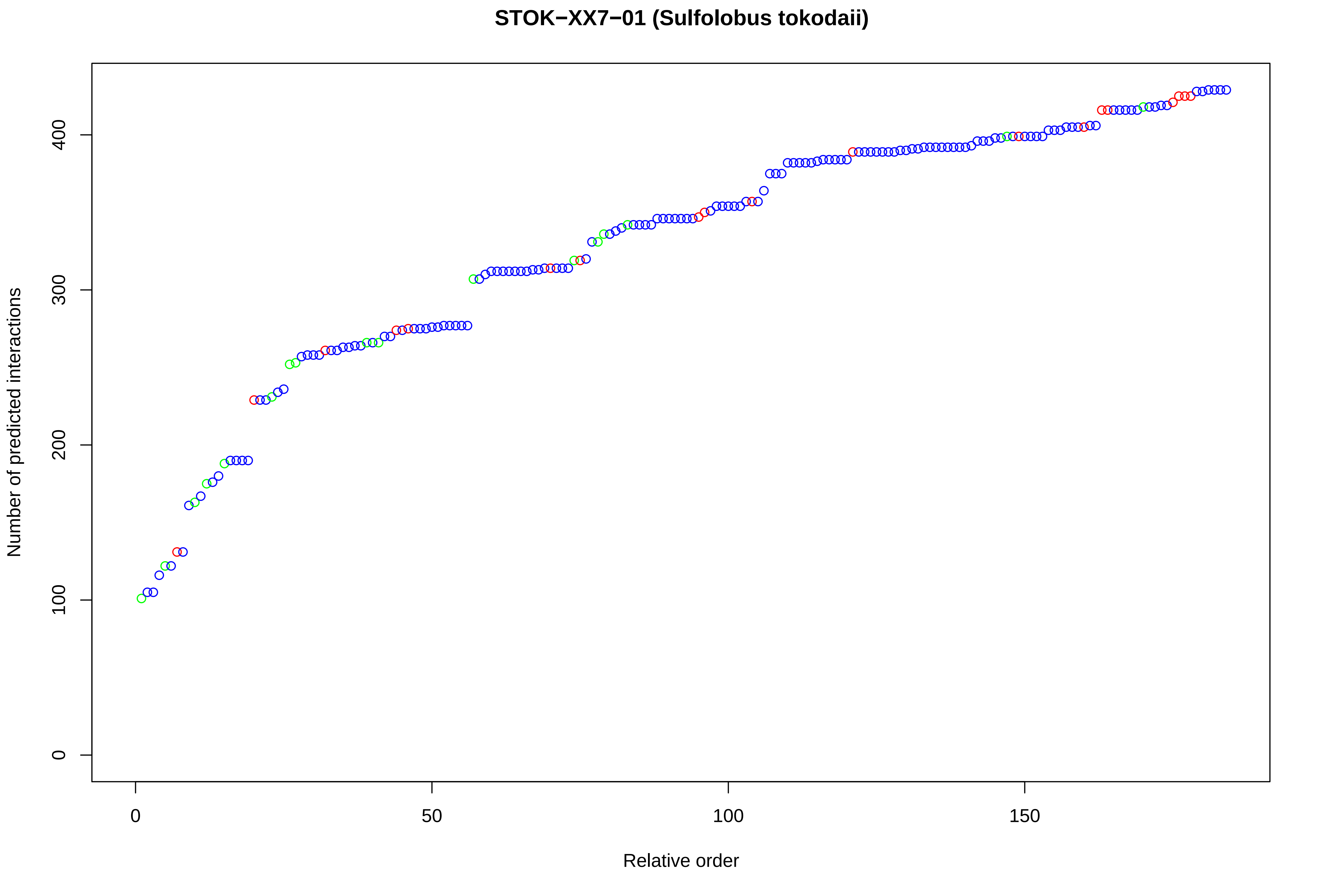

STOK−XX7−01 (Sulfolobus tokodaii)
Number of predicted interactions
0
100
200
300
400
Relative order
0
50
100
150

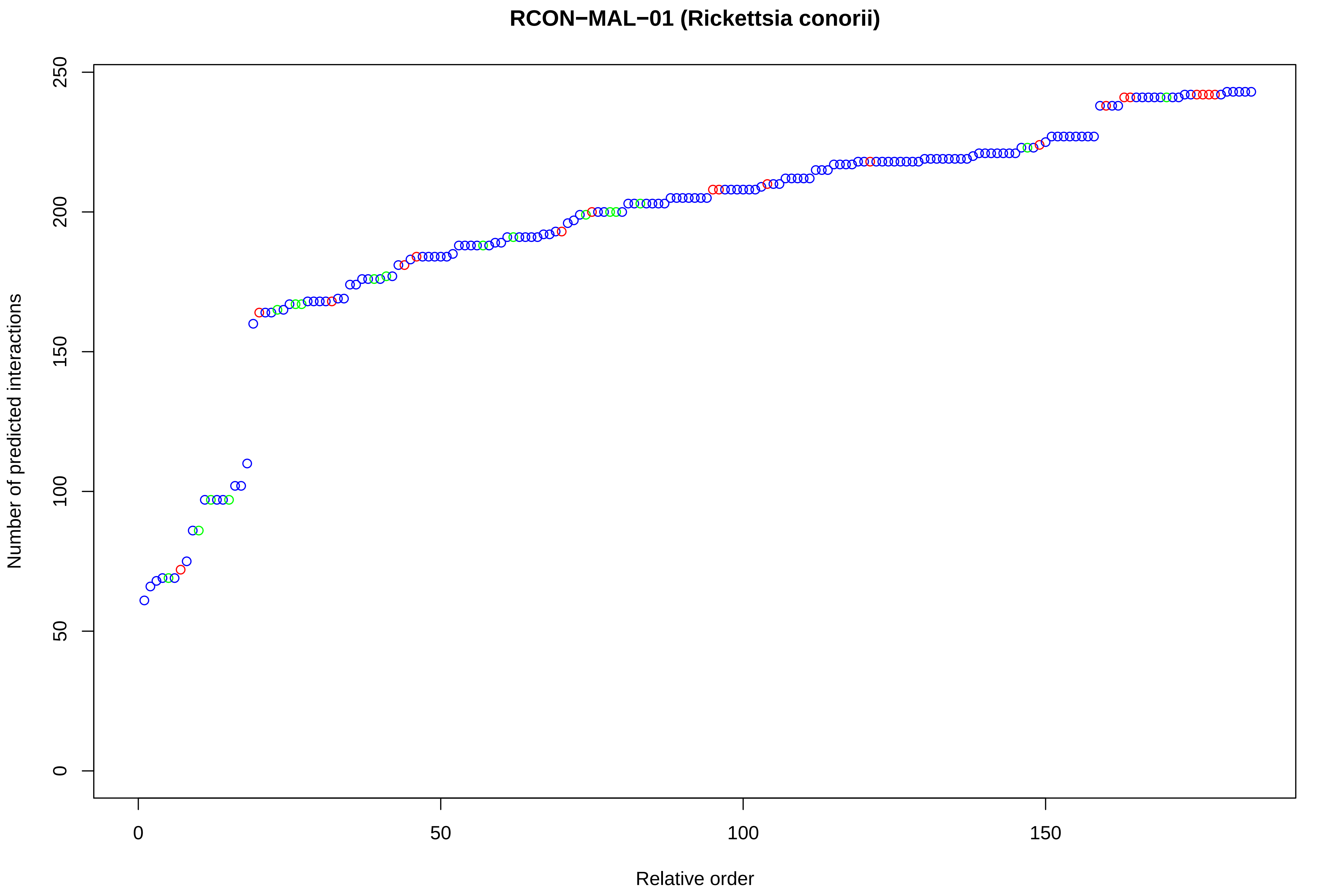

RCON−MAL−01 (Rickettsia conorii)
Number of predicted interactions
Relative order
0
50
100
150
200
250
0
50
100
150

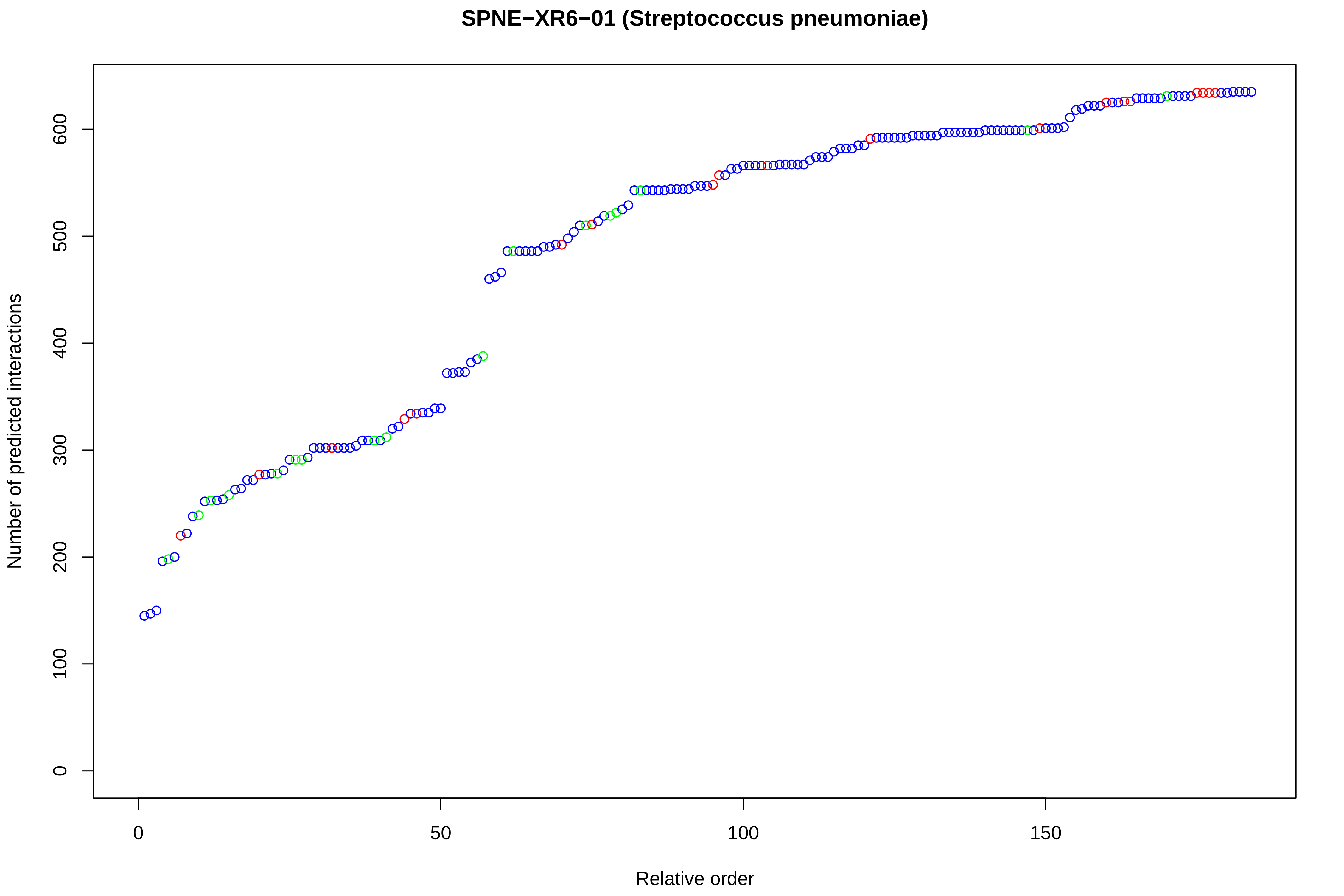
SPNE−XR6−01 (Streptococcus pneumoniae)
Number of predicted interactions
Relative order
0
100
200
300
400
500
600
0
50
100
150

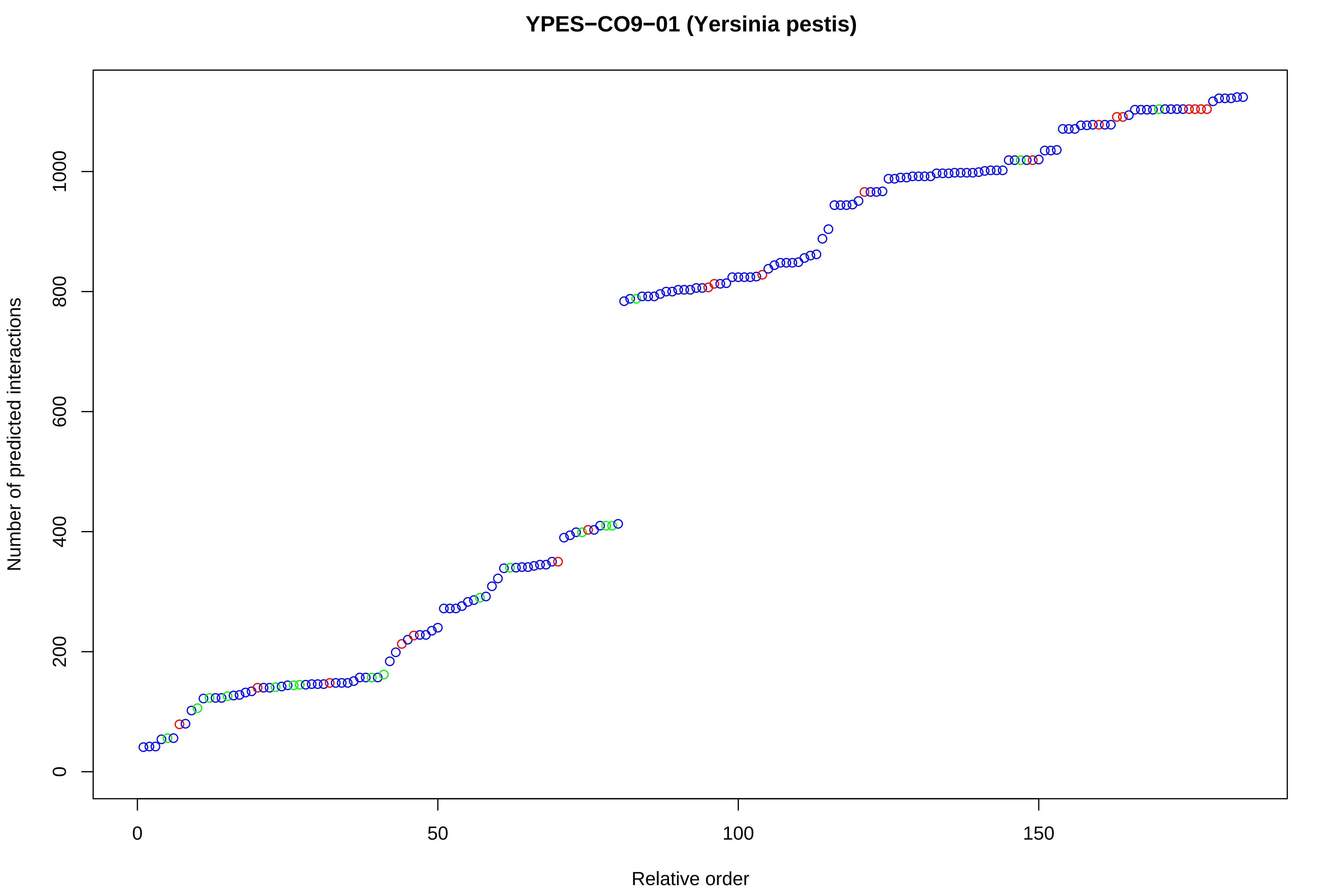
YPES−CO9−01 (Yersinia pestis)
Number of predicted interactions
Relative order
0
200
400
600
800
1000
0
50
100
150

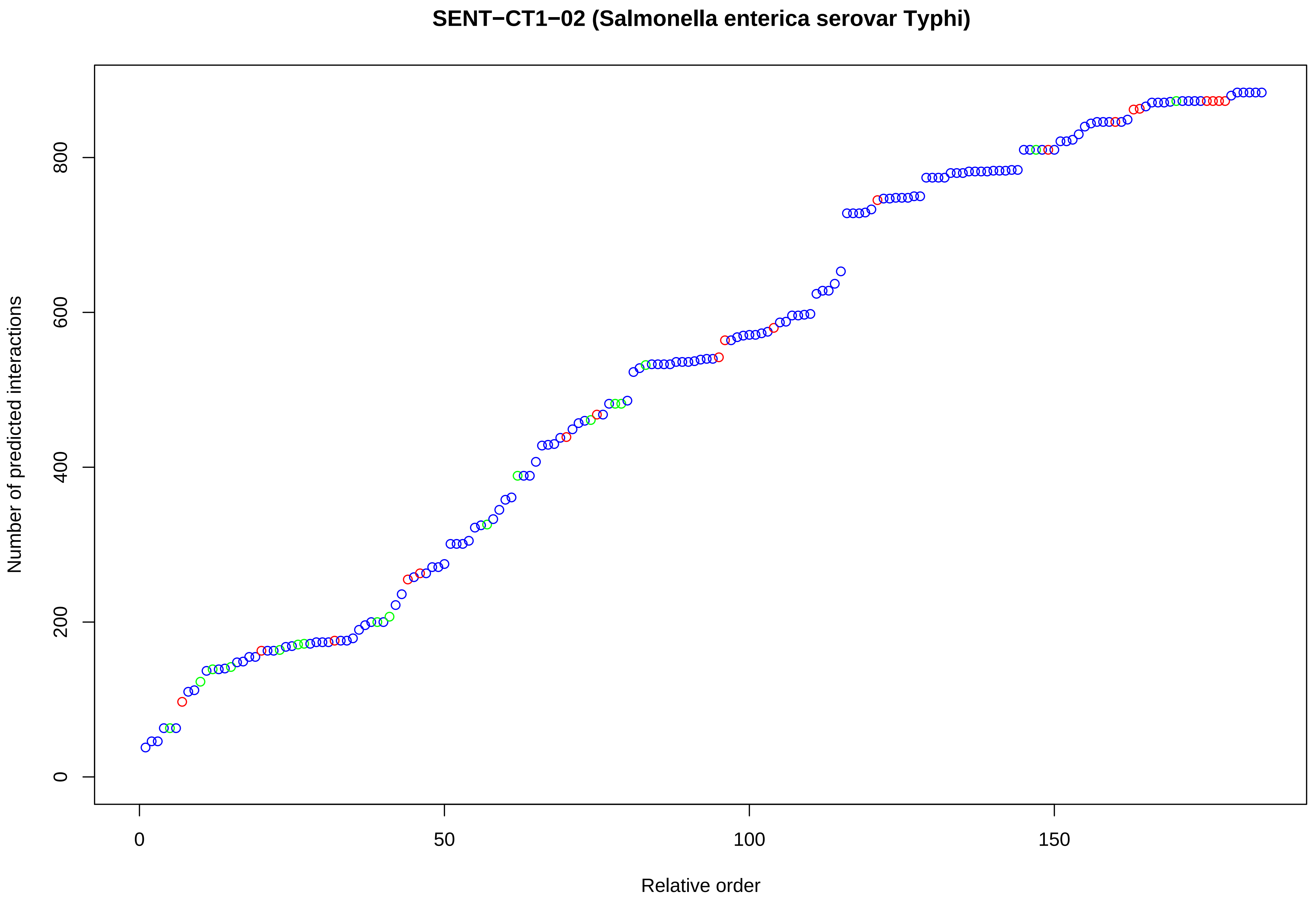

SENT−CT1−02 (Salmonella enterica serovar Typhi)
Number of predicted interactions
0
200
400
600
800
0
50
100
150
Relative order

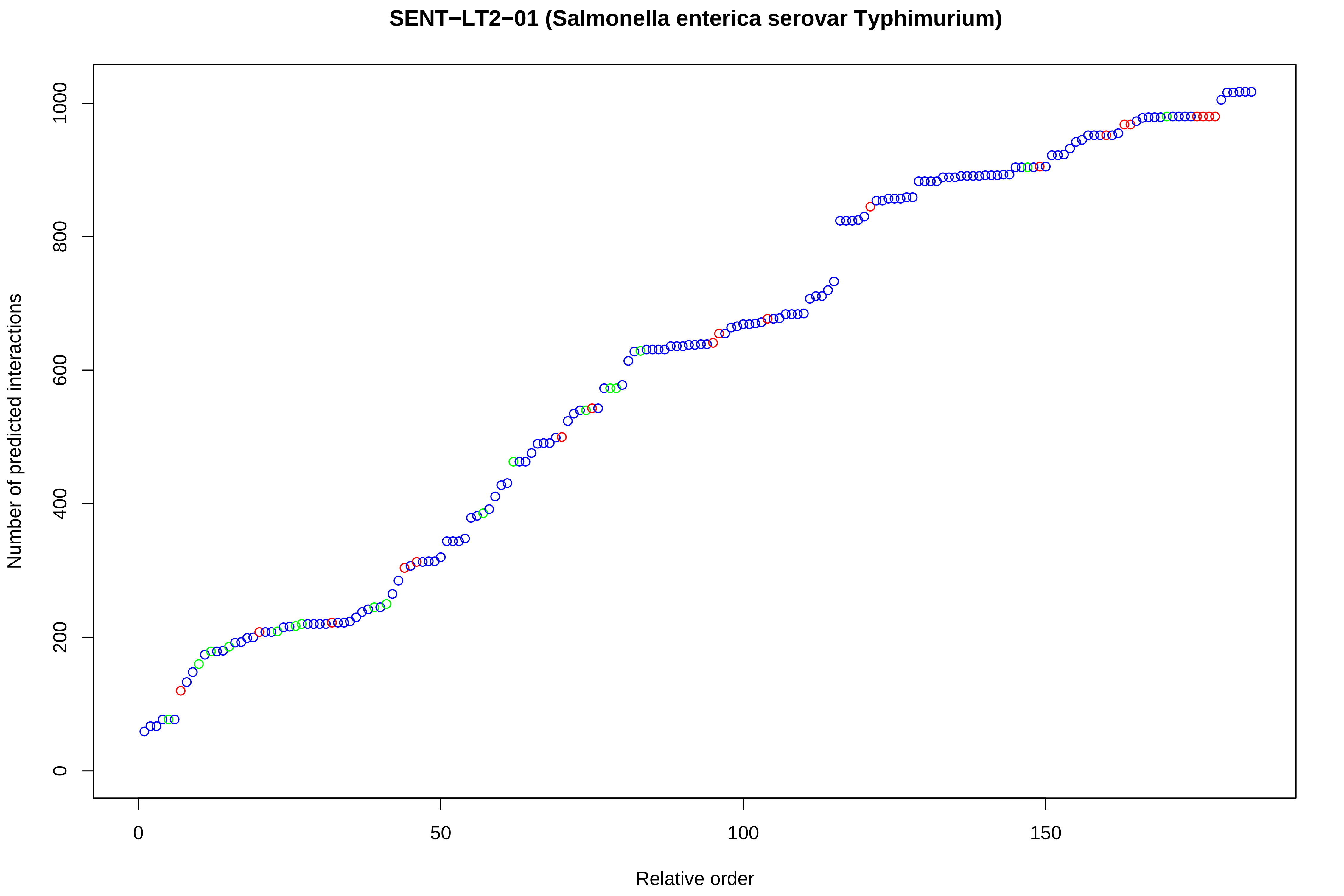
SENT−LT2−01 (Salmonella enterica serovar Typhimurium)
Number of predicted interactions
0
200
400
600
800
1000
0
50
100
150
Relative order

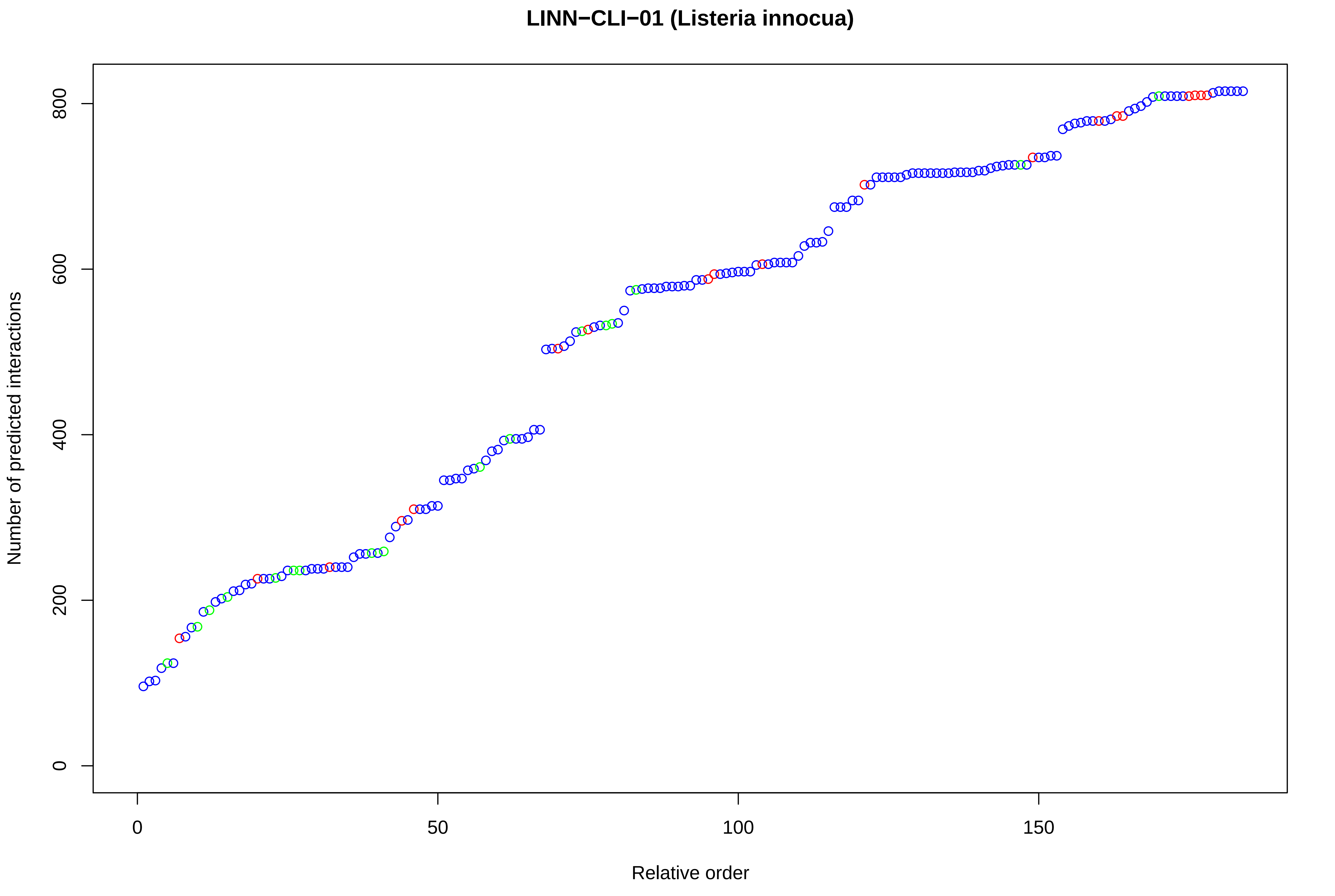

LINN−CLI−01 (Listeria innocua)
Number of predicted interactions
Relative order
0
200
400
600
800
0
50
100
150

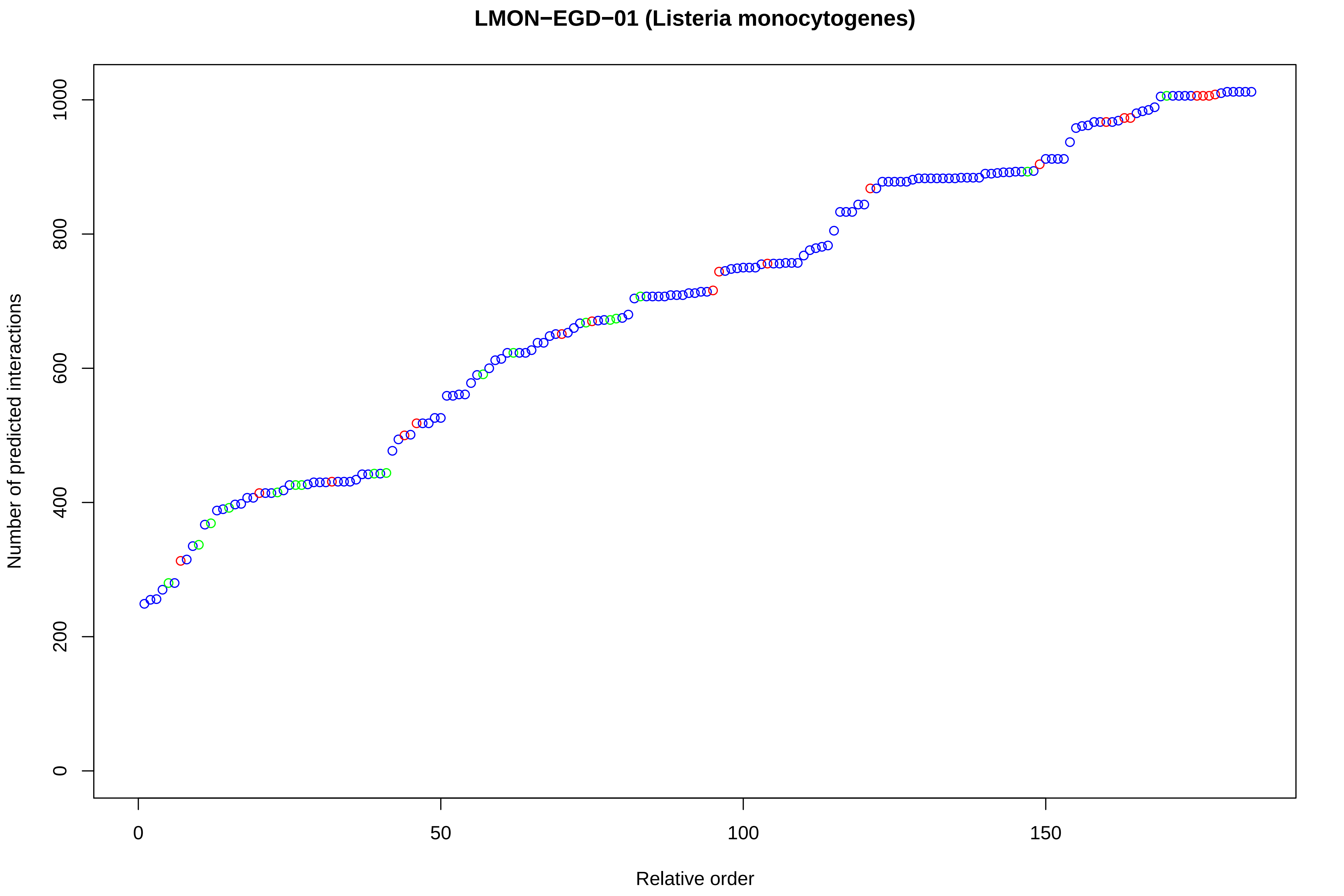
LMON−EGD−01 (Listeria monocytogenes)
Number of predicted interactions
0
200
400
600
800
1000
0
50
100
150
Relative order

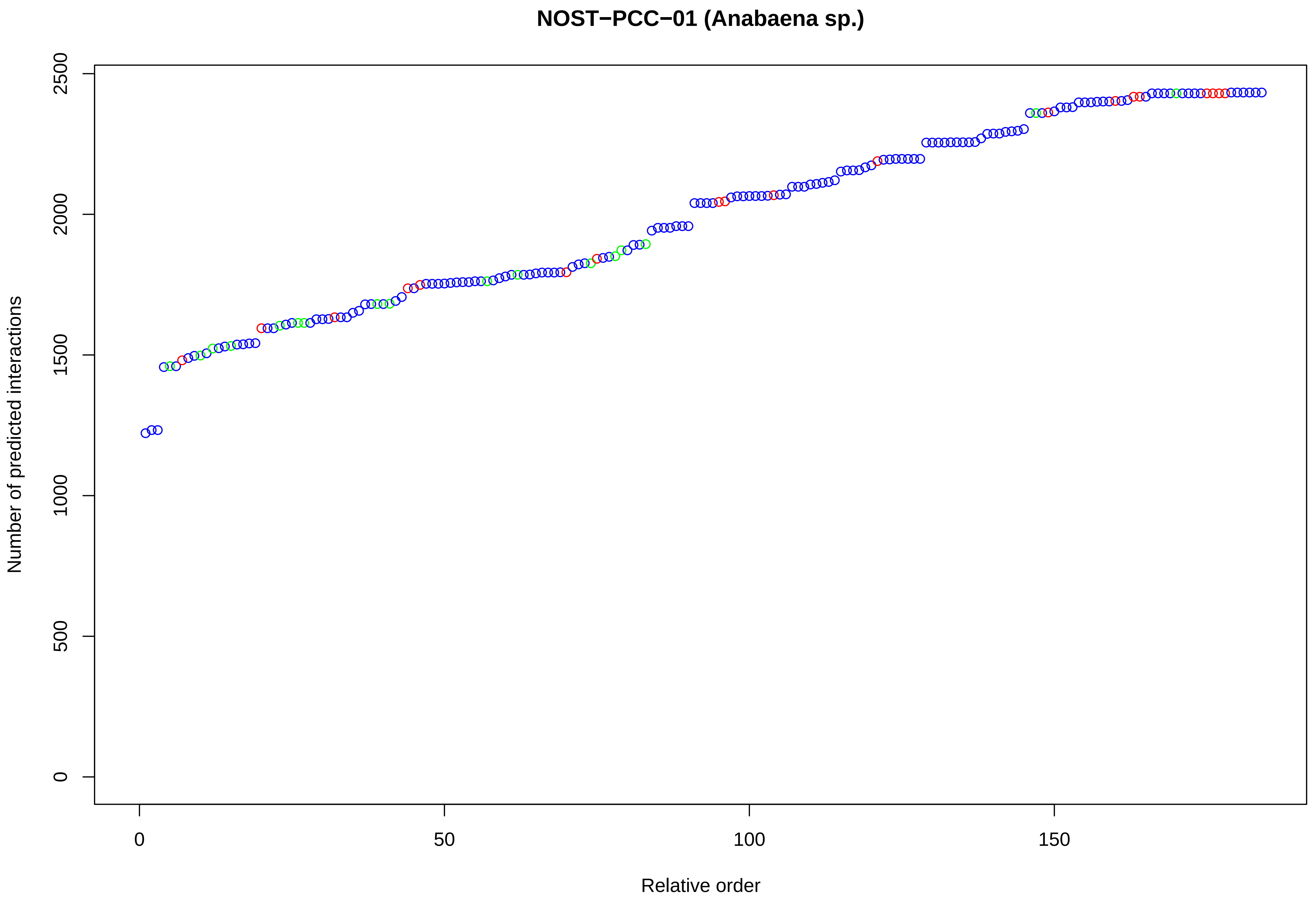
NOST−PCC−01 (Anabaena sp.)
Number of predicted interactions
0
500
1000
1500
2000
2500
0
50
100
150
Relative order

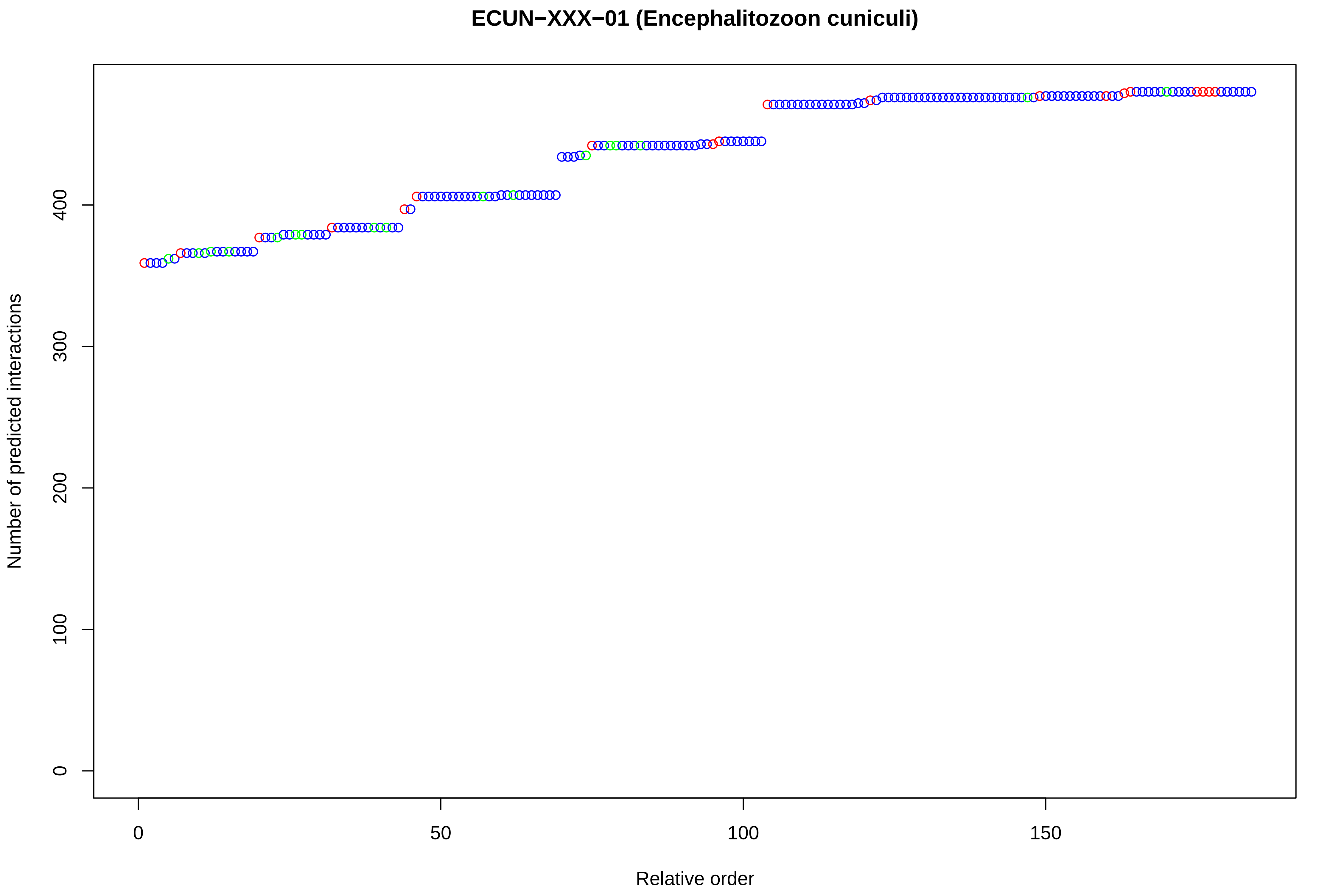

ECUN−XXX−01 (Encephalitozoon cuniculi)
Number of predicted interactions
0
100
200
300
400
0
50
100
150
Relative order

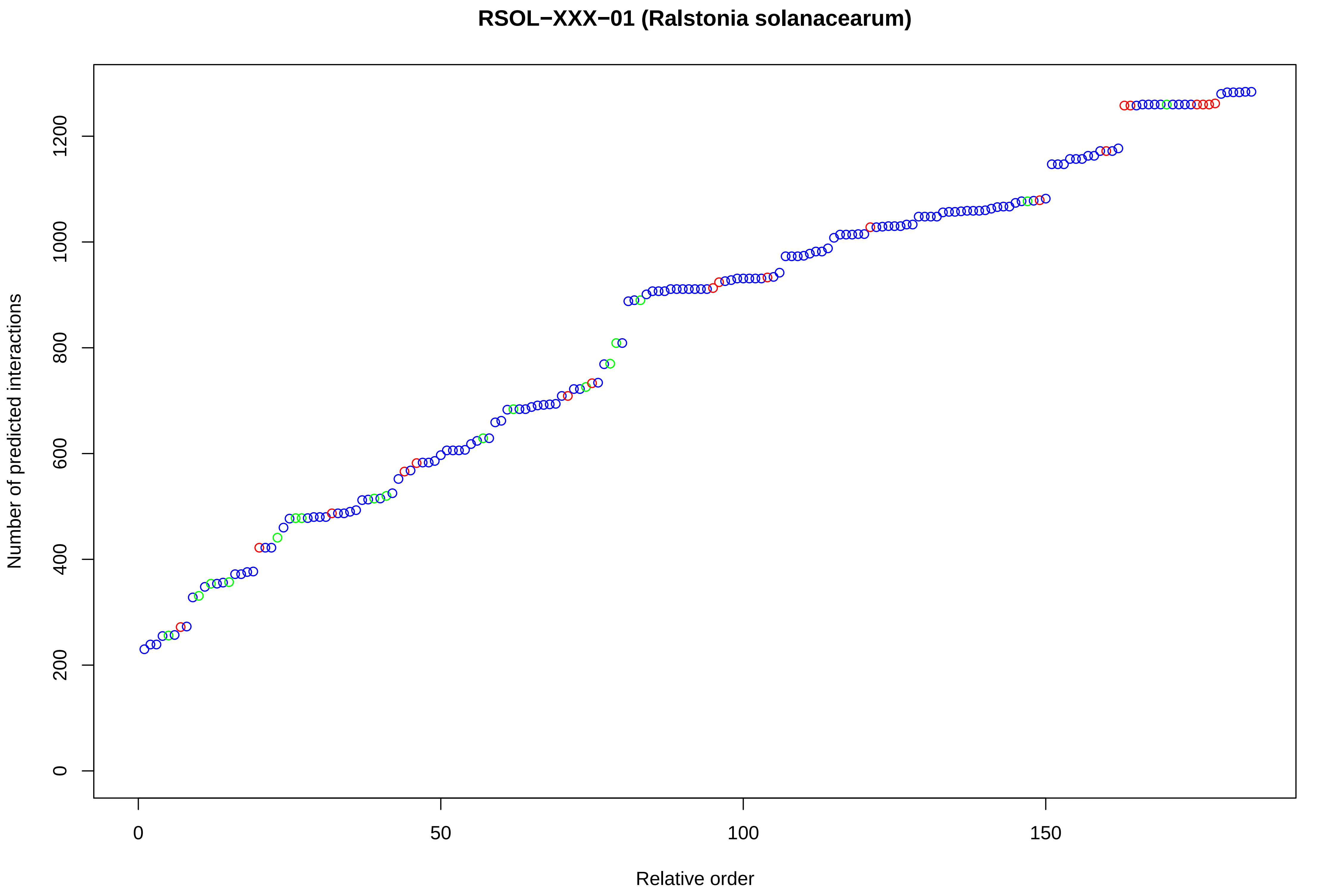
RSOL−XXX−01 (Ralstonia solanacearum)
Number of predicted interactions
0
200
400
600
800
1000
1200
Relative order
0
50
100
150

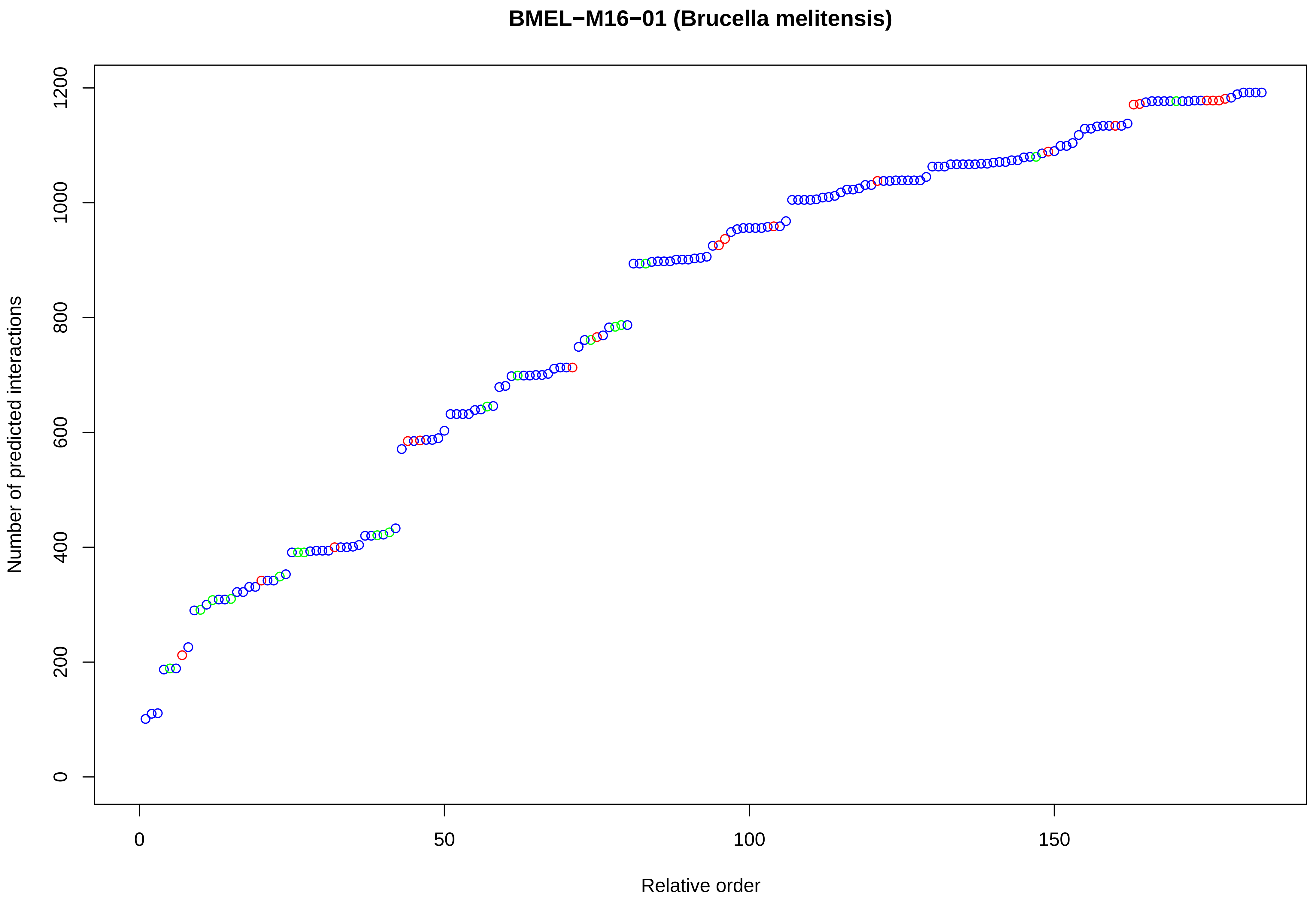

BMEL−M16−01 (Brucella melitensis)
Number of predicted interactions
Relative order
0
200
400
600
800
1000
1200
0
50
100
150

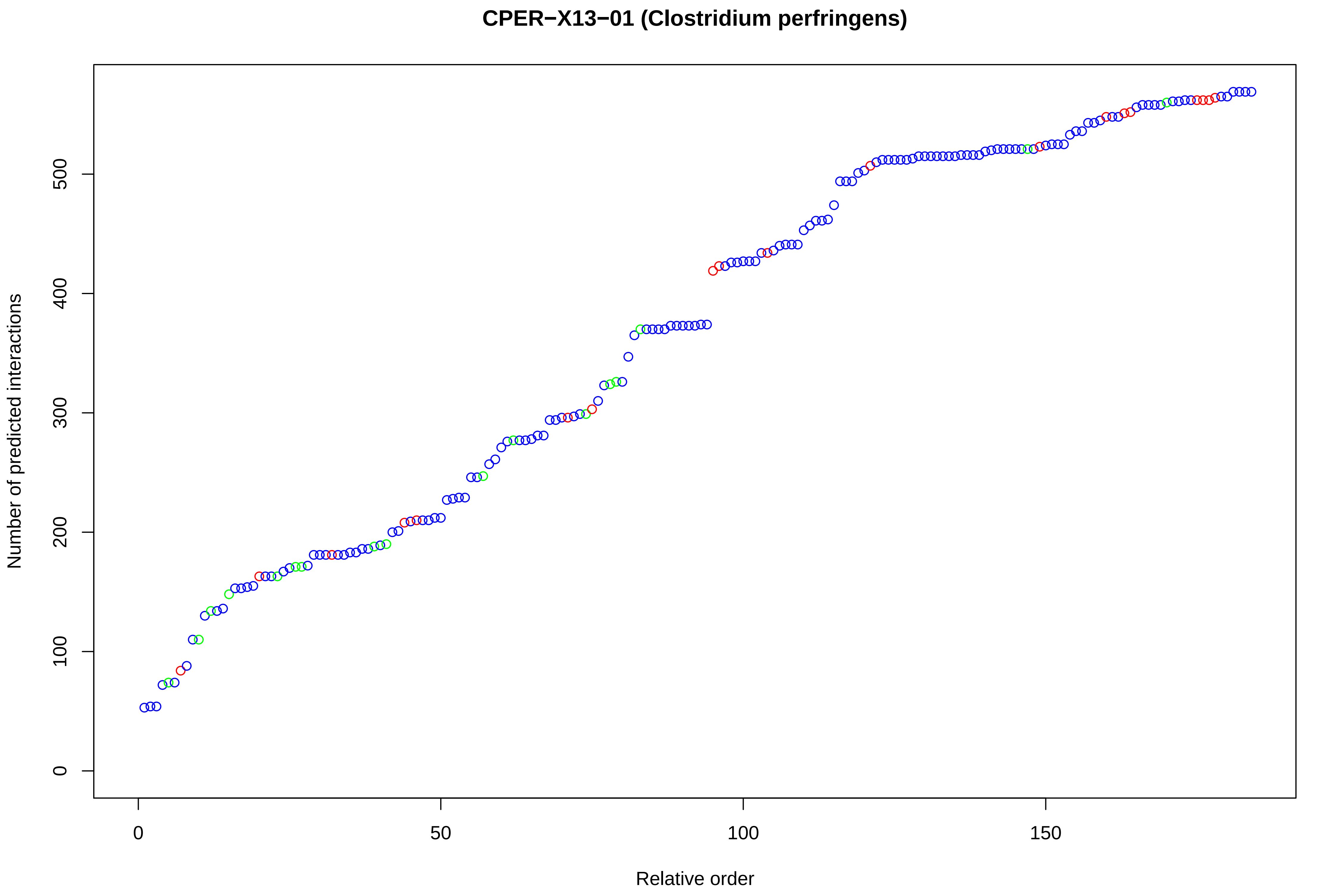
CPER−X13−01 (Clostridium perfringens)
Number of predicted interactions
0
100
200
300
400
500
0
50
100
150
Relative order

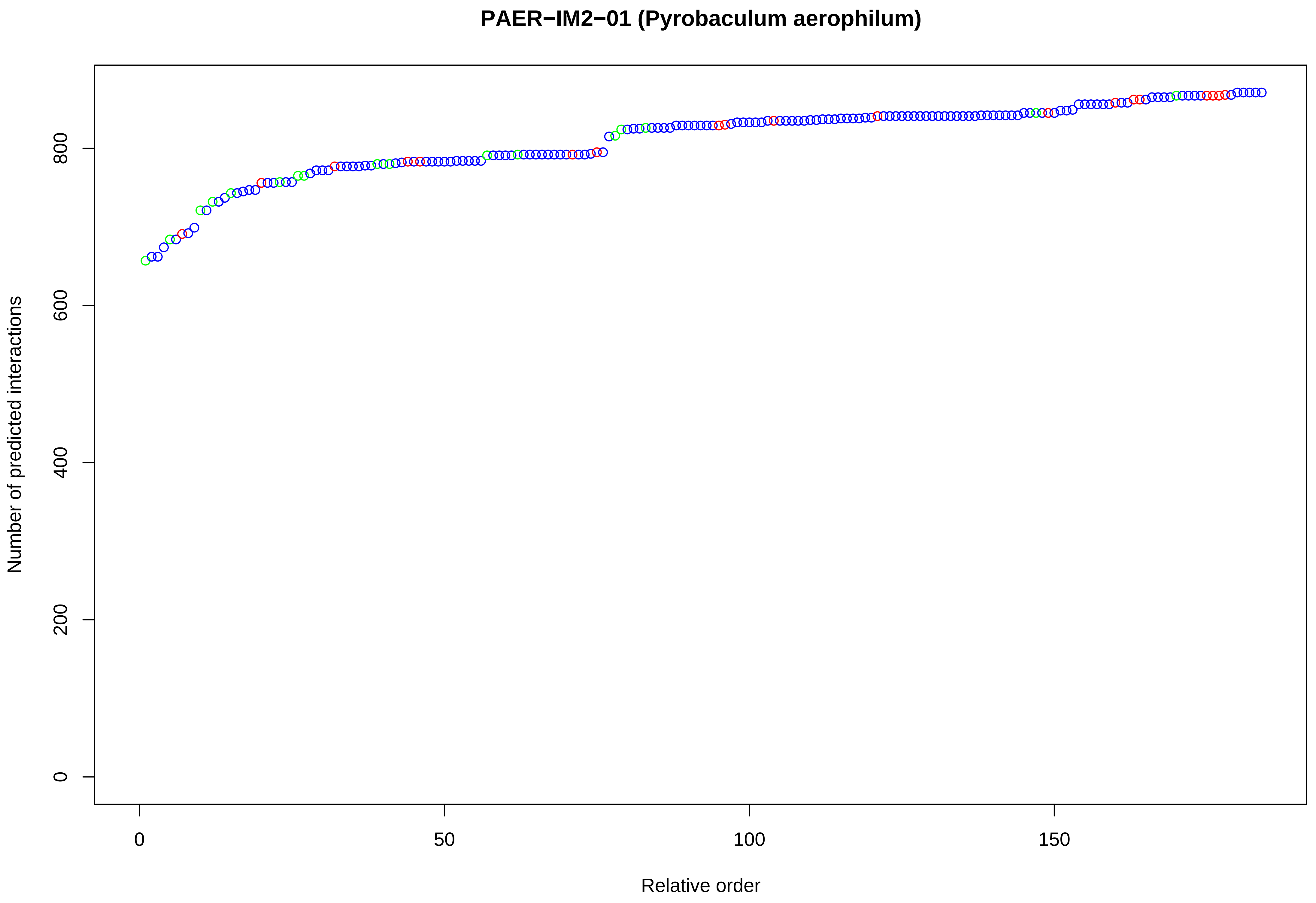
PAER−IM2−01 (Pyrobaculum aerophilum)
Number of predicted interactions
0
200
400
600
800
0
50
100
150
Relative order

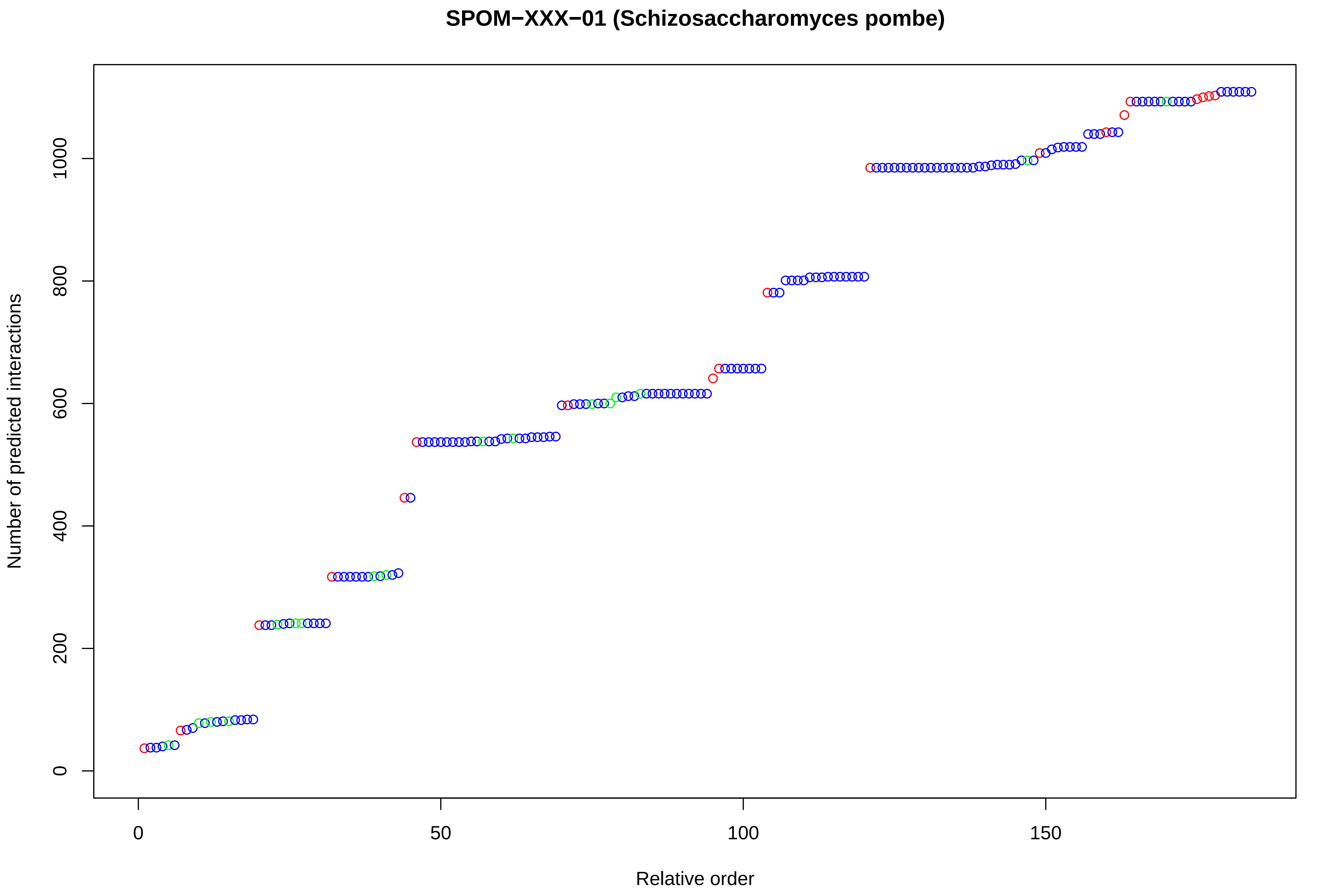

SPOM−XXX−01 (Schizosaccharomyces pombe)
Number of predicted interactions
0
200
400
600
800
1000
0
50
100
150
Relative order

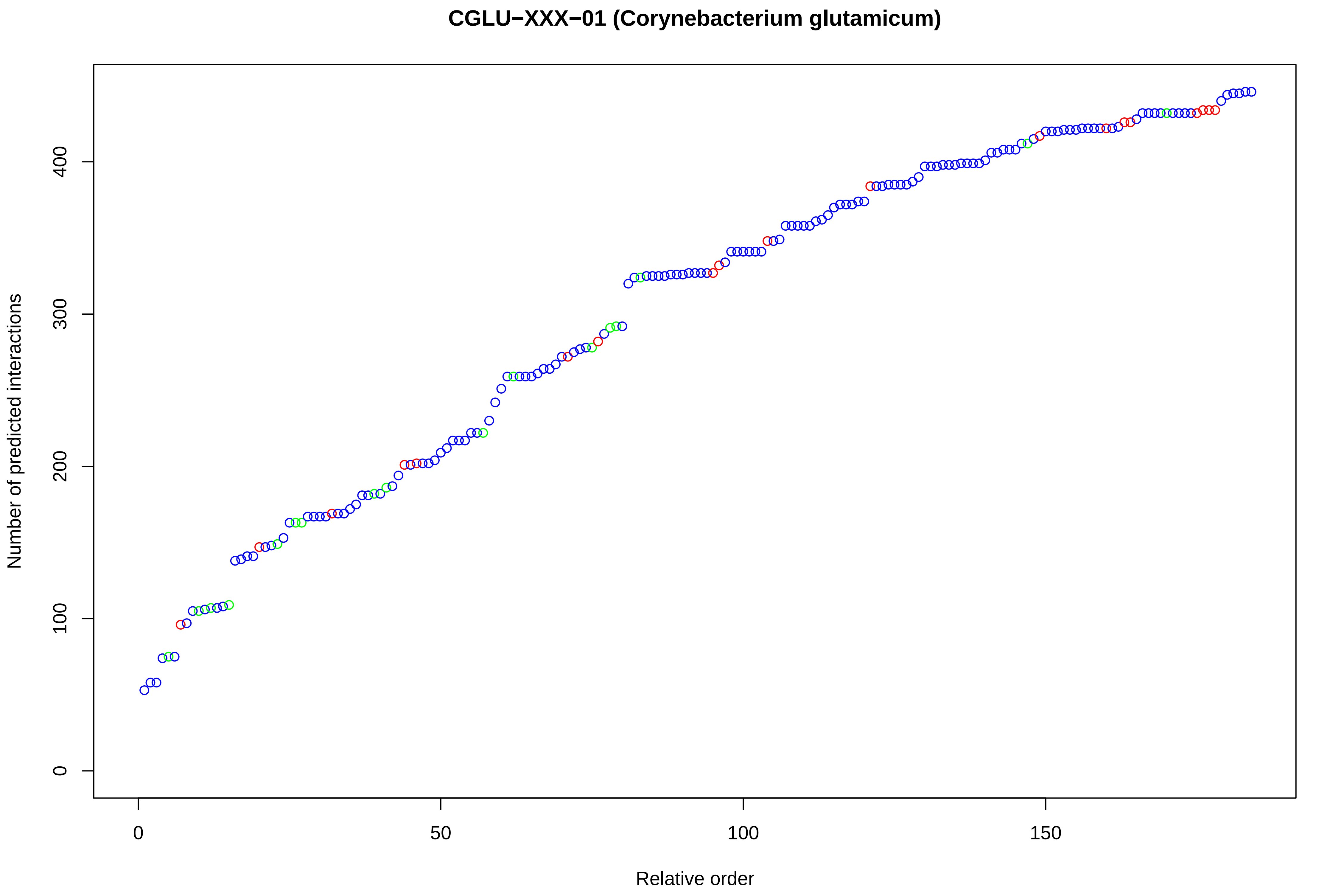

CGLU−XXX−01 (Corynebacterium glutamicum)
Number of predicted interactions
0
100
200
300
400
Relative order
0
50
100
150

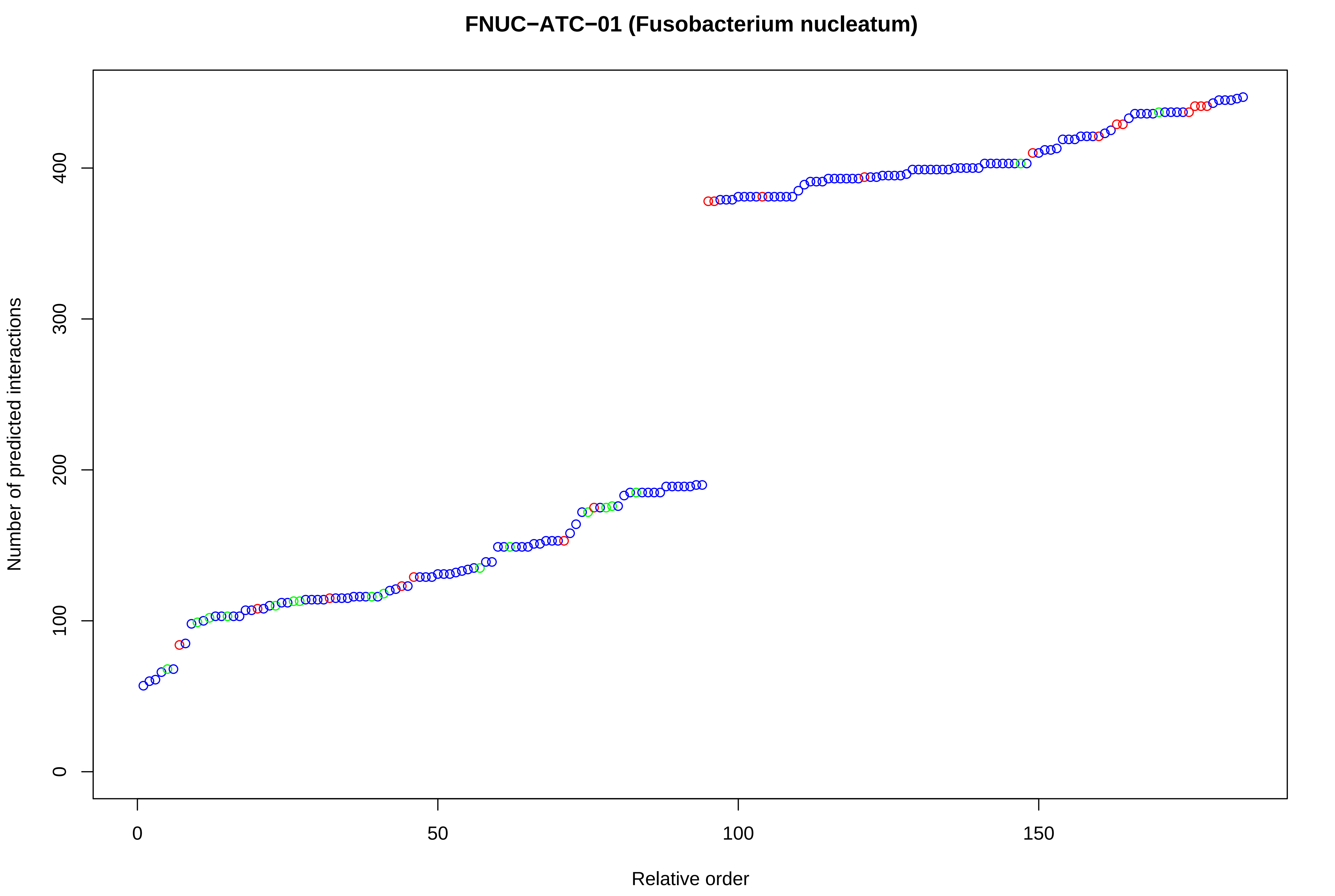

FNUC−ATC−01 (Fusobacterium nucleatum)
Number of predicted interactions
0
100
200
300
400
Relative order
0
50
100
150

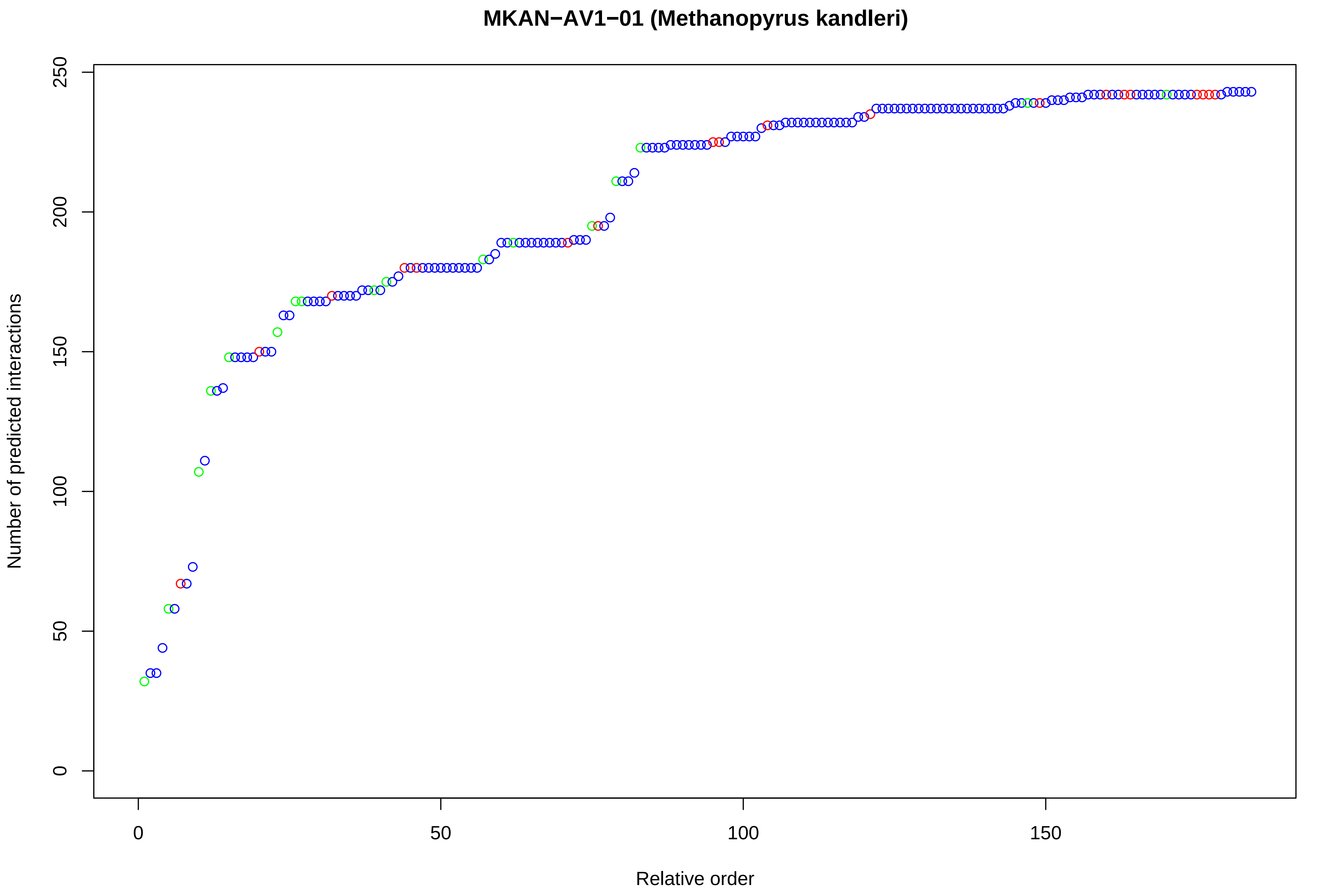

MKAN−AV1−01 (Methanopyrus kandleri)
Number of predicted interactions
Relative order
0
50
100
150
200
250
0
50
100
150

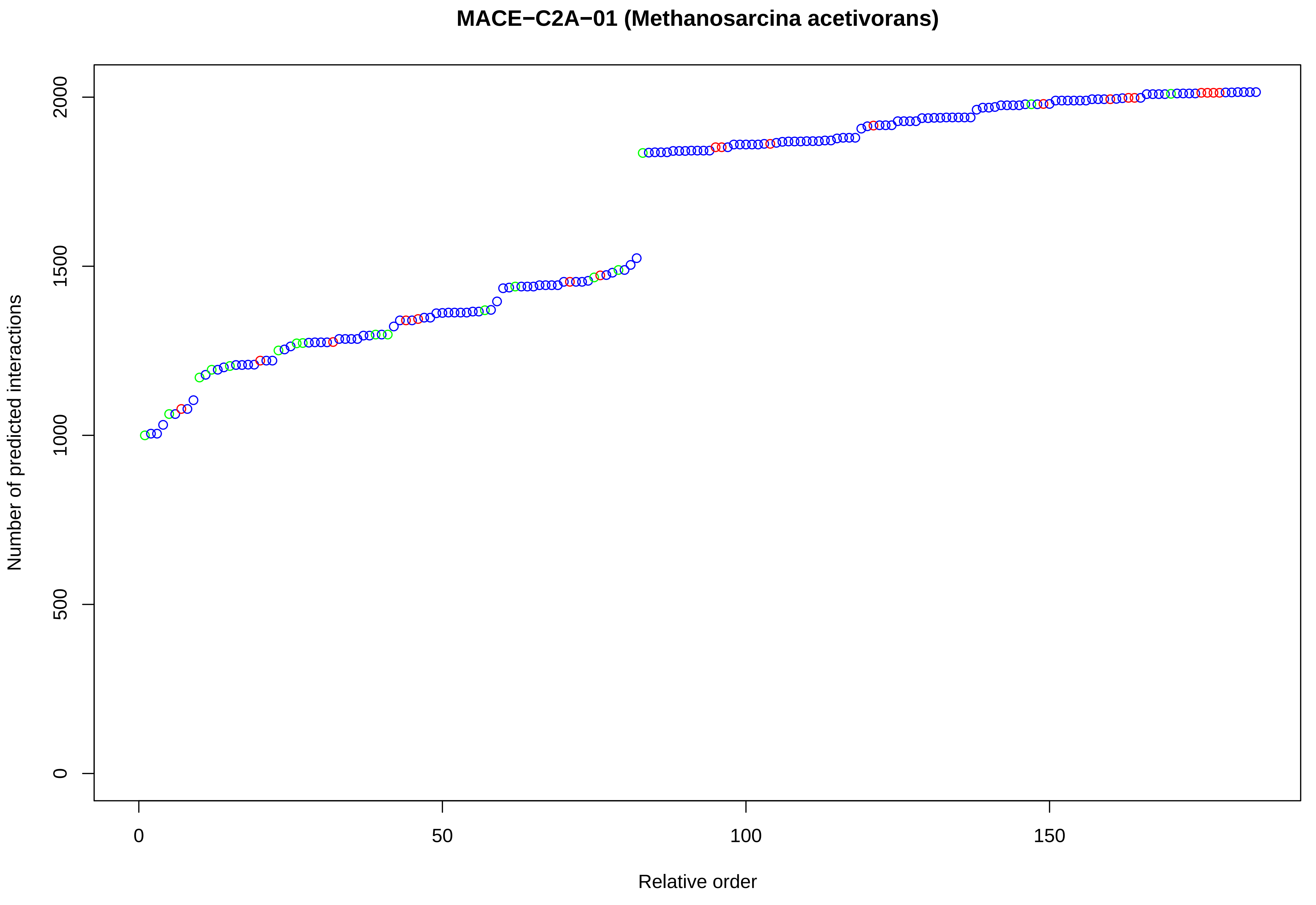
MACE−C2A−01 (Methanosarcina acetivorans)
Number of predicted interactions
Relative order
0
500
1000
1500
2000
0
50
100
150

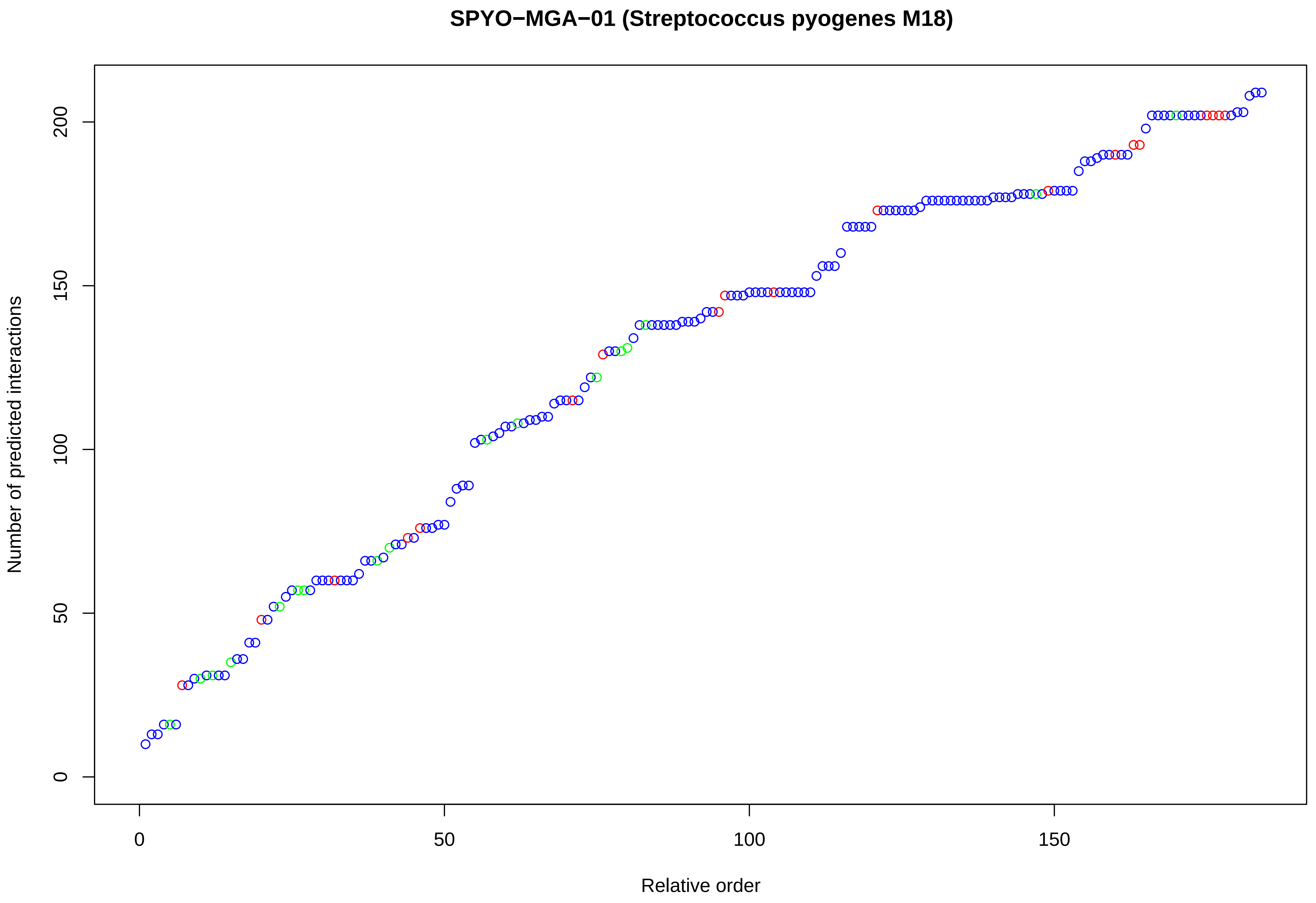

SPYO−MGA−01 (Streptococcus pyogenes M18)
Number of predicted interactions
Relative order
0
50
100
150
200

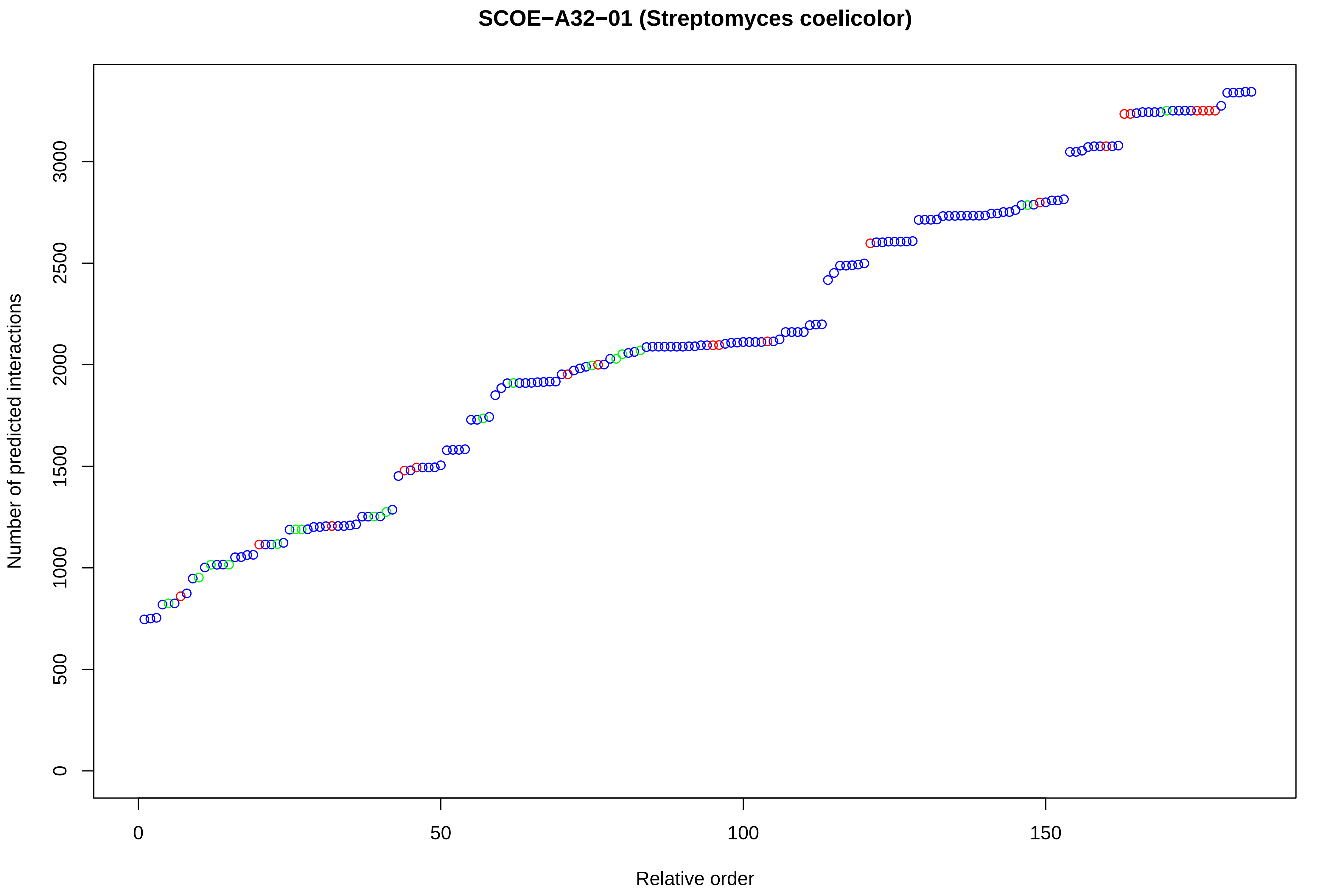
SCOE−A32−01 (Streptomyces coelicolor)
Number of predicted interactions
0
500
1000
1500
2000
2500
3000
0
50
100
150
Relative order

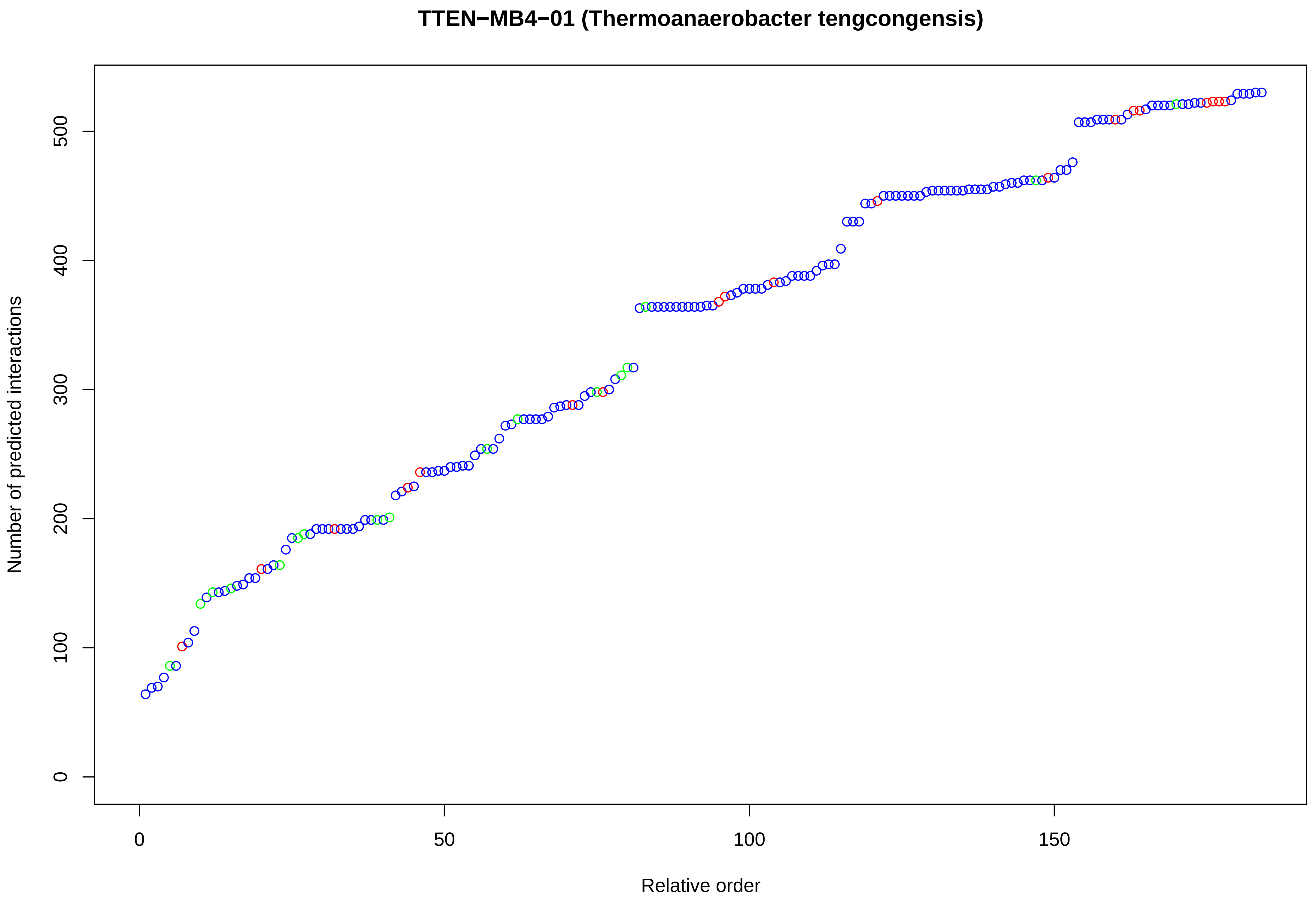
TTEN−MB4−01 (Thermoanaerobacter tengcongensis)
Number of predicted interactions
0
100
200
300
400
500
0
50
100
150
Relative order

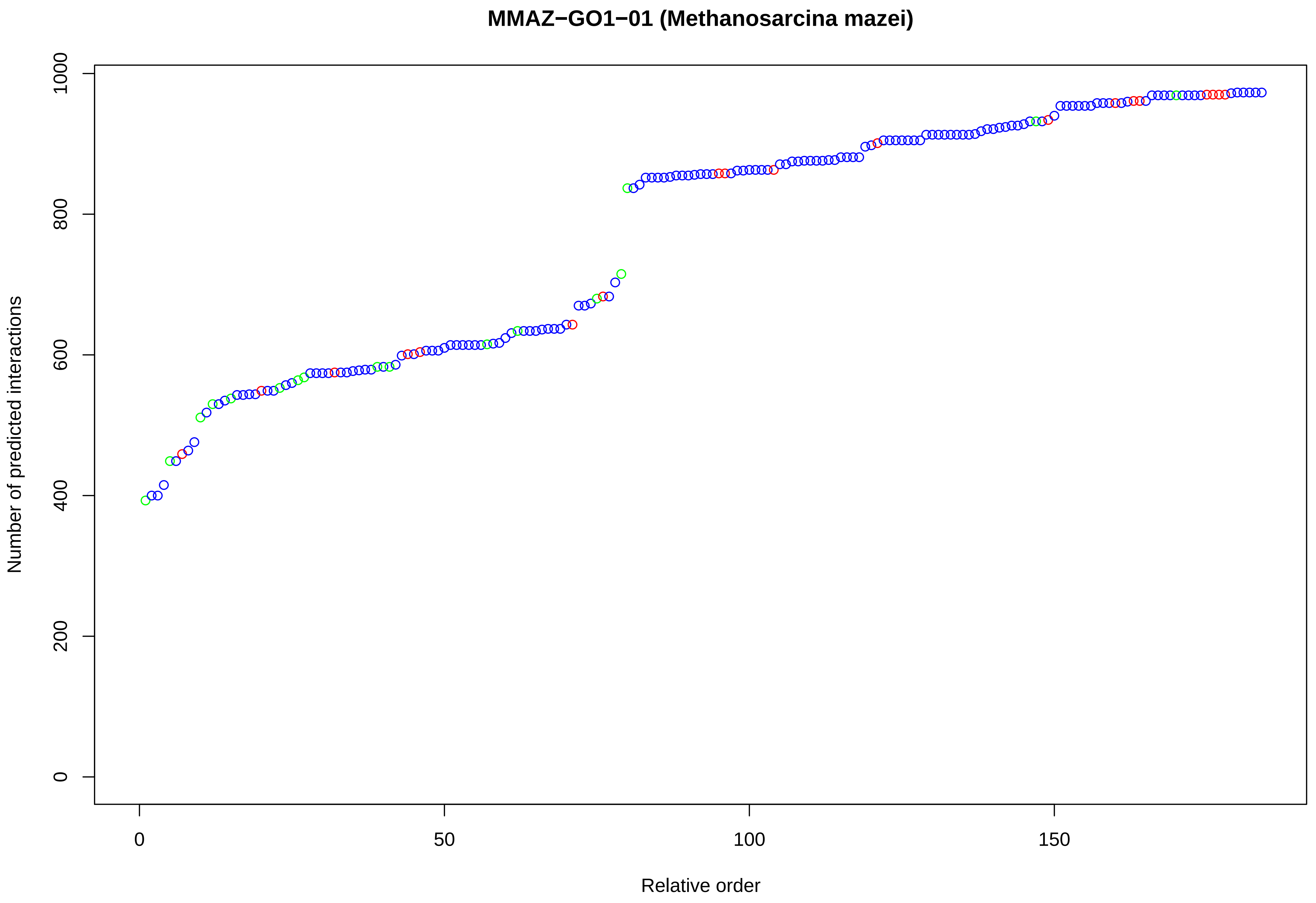
MMAZ−GO1−01 (Methanosarcina mazei)
Number of predicted interactions
Relative order
0
200
400
600
800
1000
0
50
100
150

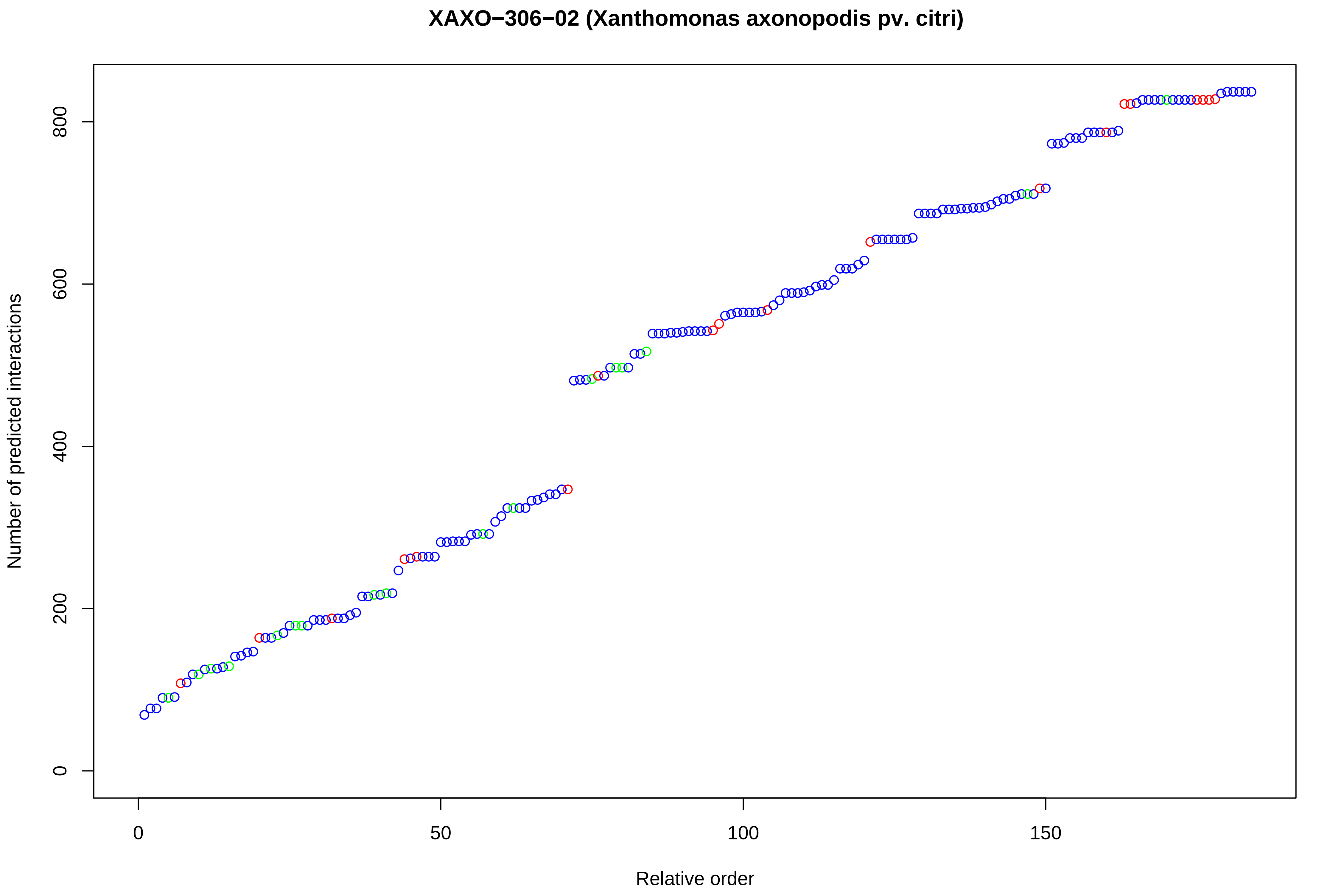

XAXO−306−02 (Xanthomonas axonopodis pv. citri)
Number of predicted interactions
Relative order
0
200
400
600
800
0
50
100
150

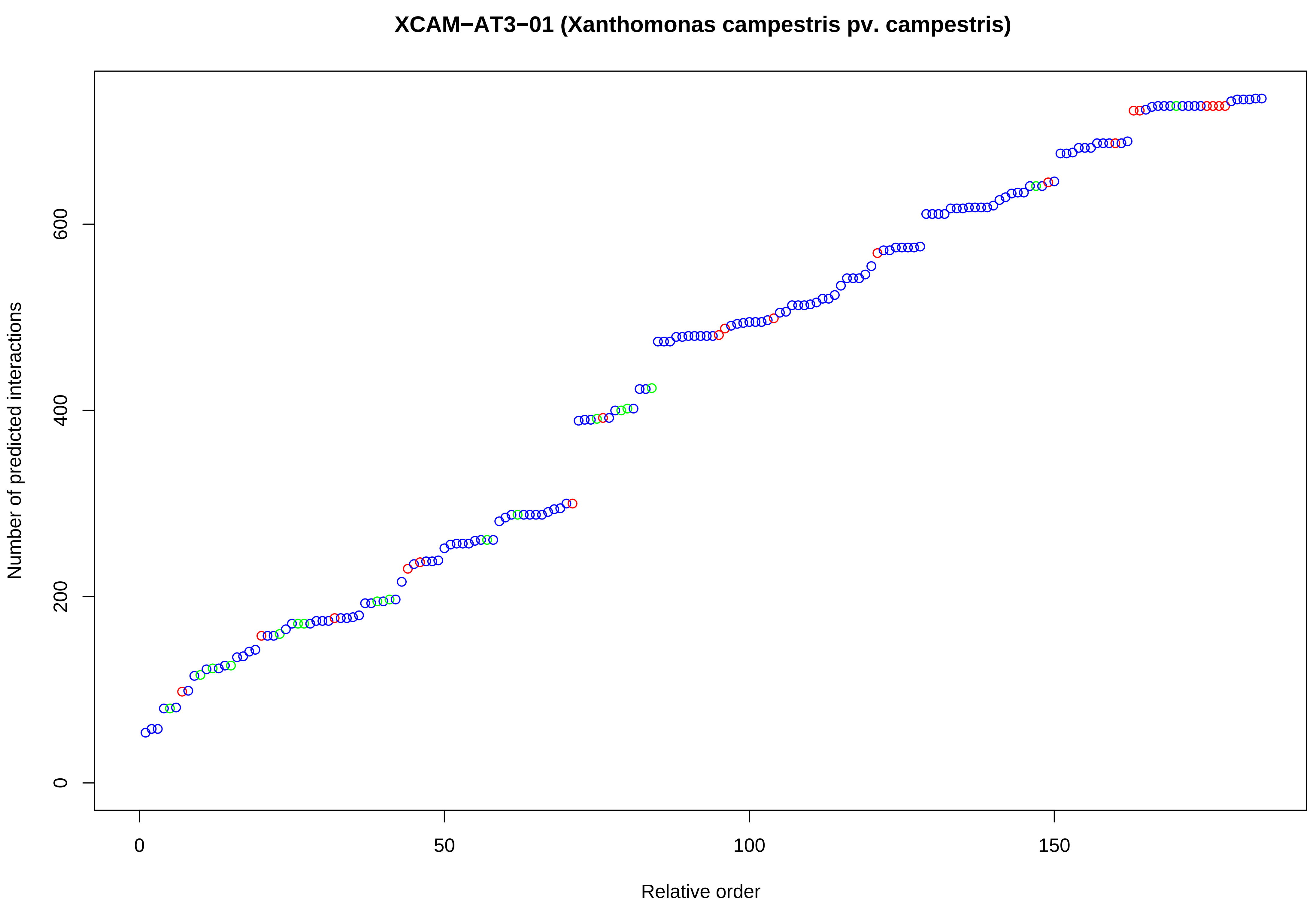
XCAM−AT3−01 (Xanthomonas campestris pv. campestris)
Number of predicted interactions
0
200
400
600
0
50
100
150
Relative order

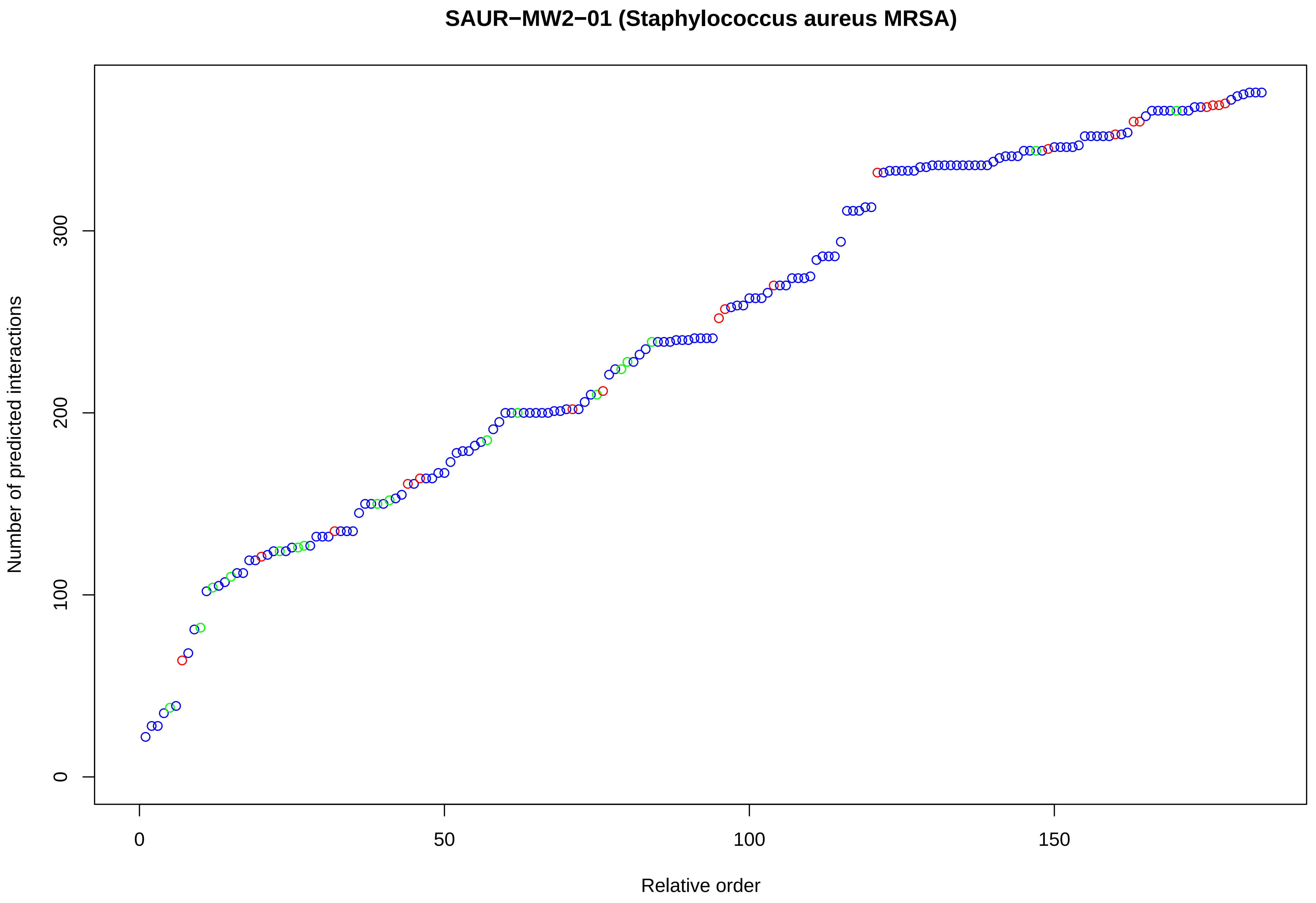

SAUR−MW2−01 (Staphylococcus aureus MRSA)
Number of predicted interactions
0
100
200
300
0
50
100
150
Relative order

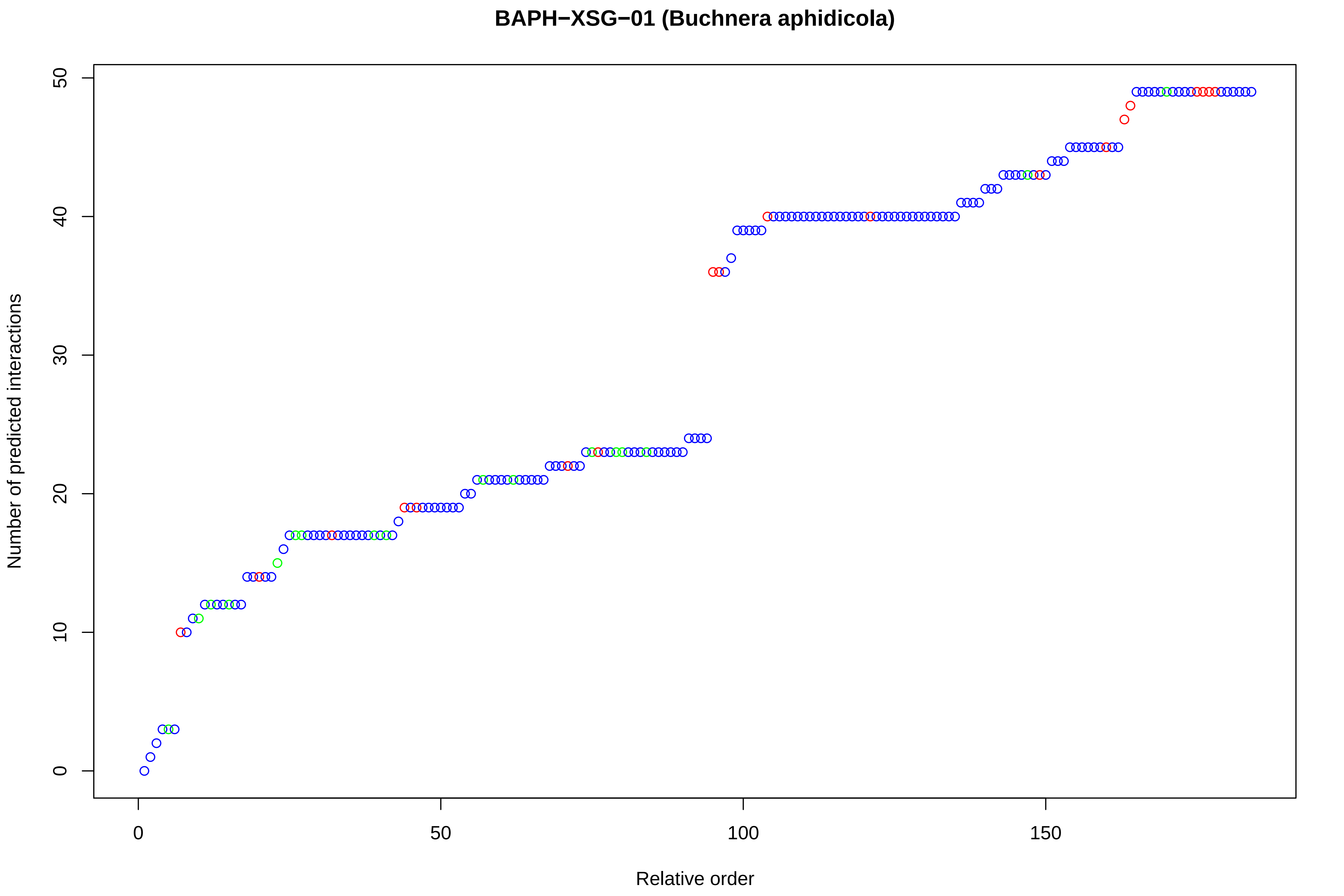

BAPH−XSG−01 (Buchnera aphidicola)
Number of predicted interactions
0
10
20
30
40
50
0
50
100
150
Relative order

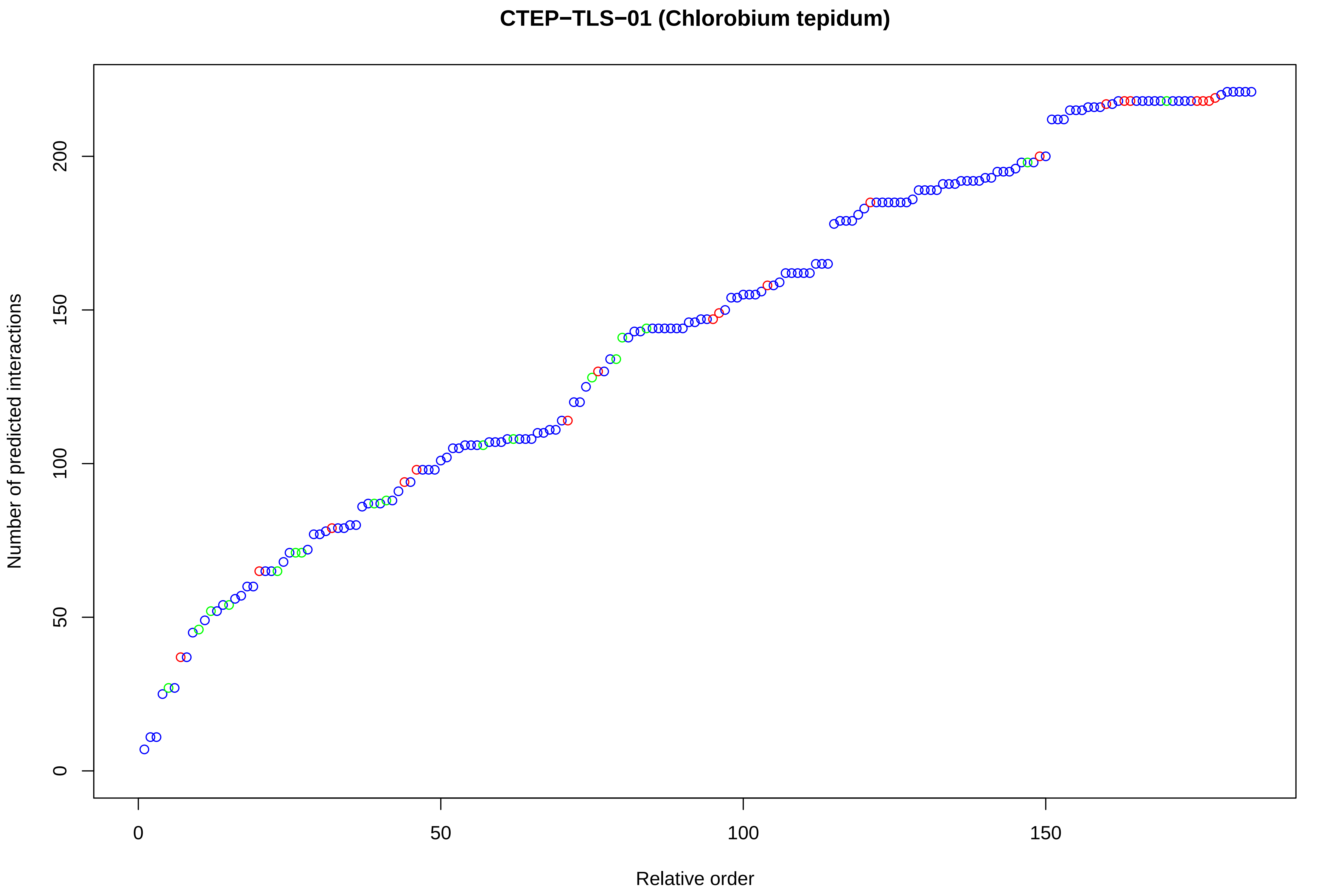
CTEP−TLS−01 (Chlorobium tepidum)
Number of predicted interactions
Relative order
0
50
100
150
200

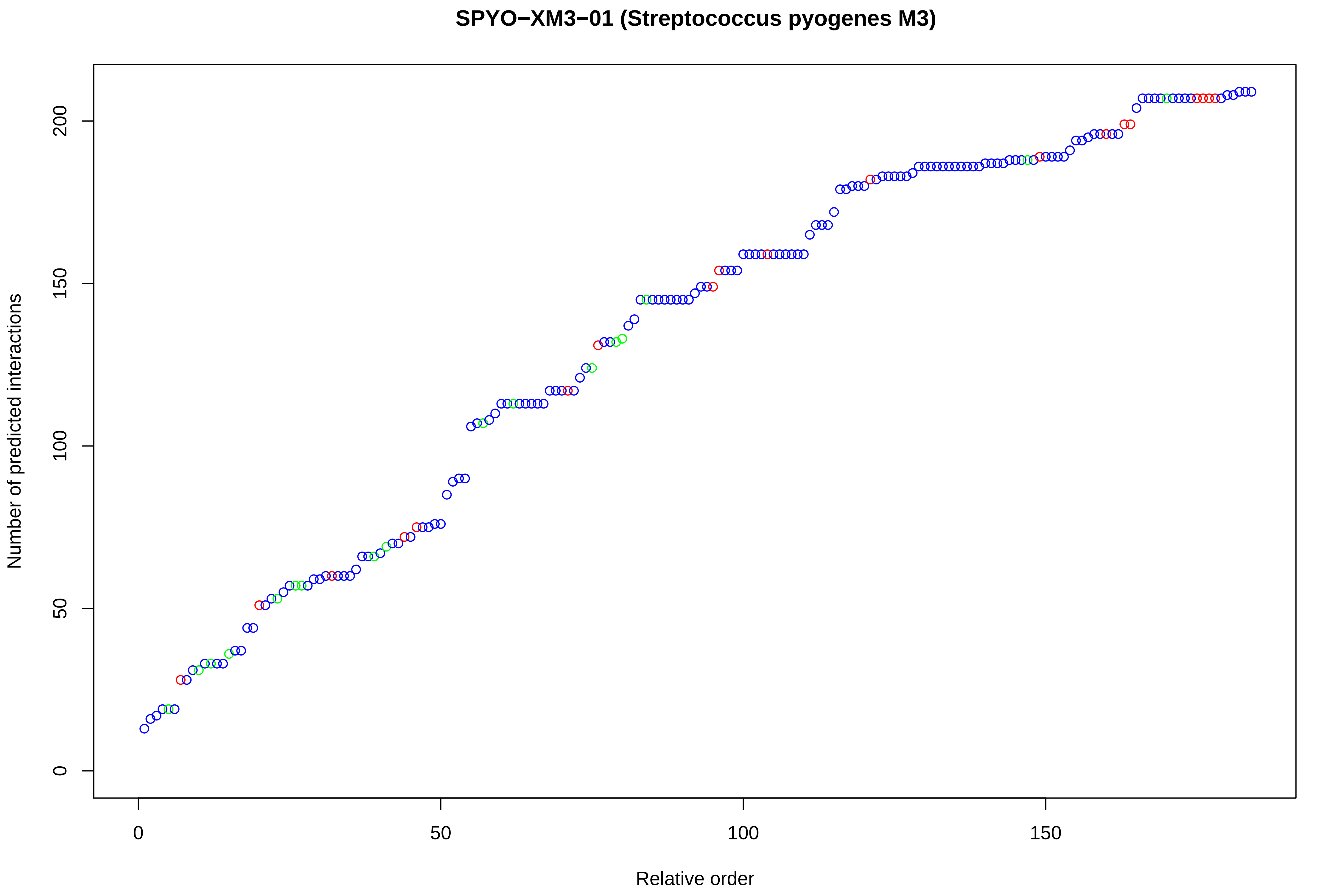

SPYO−XM3−01 (Streptococcus pyogenes M3)
Number of predicted interactions
Relative order
0
50
100
150
200

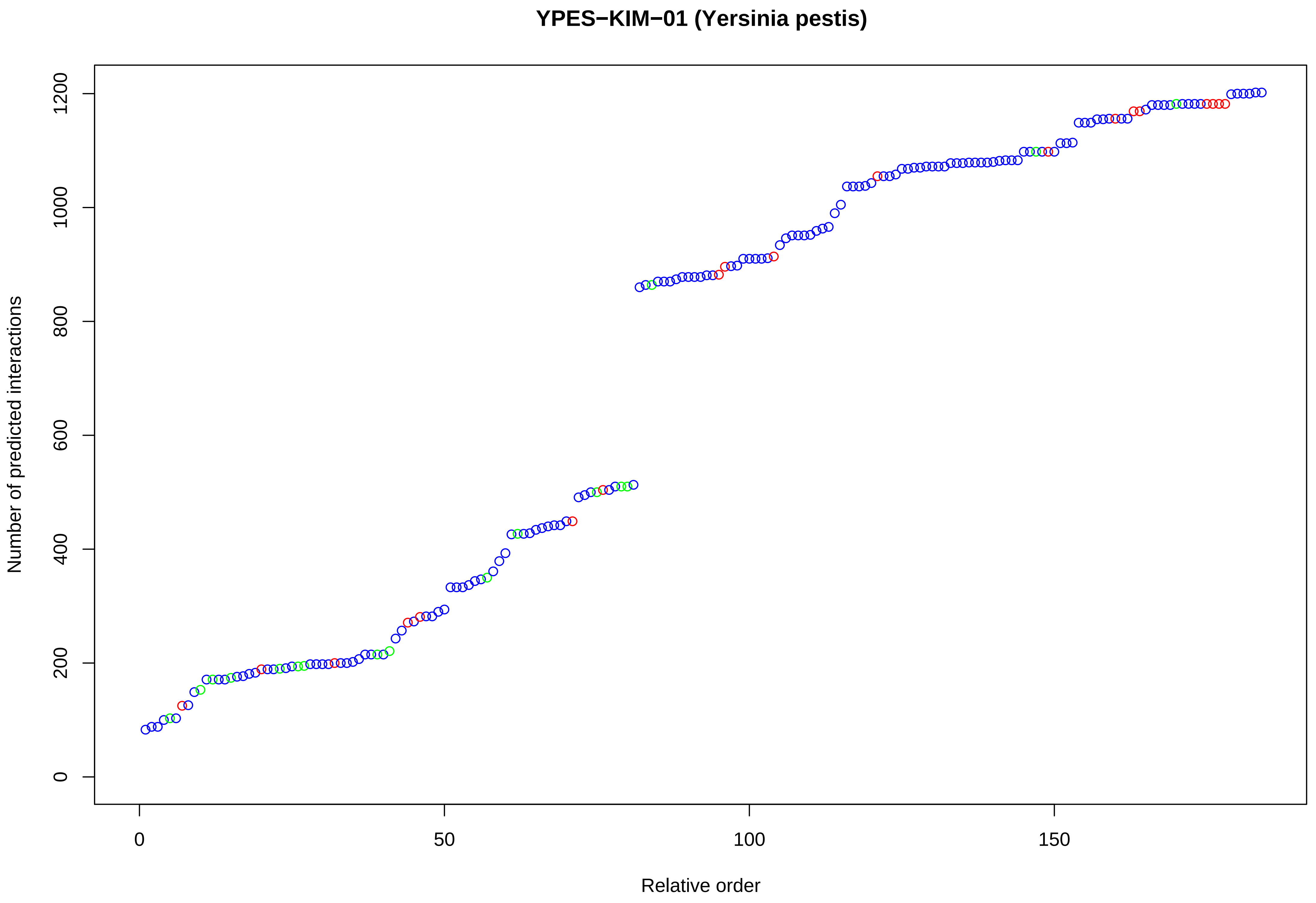
YPES−KIM−01 (Yersinia pestis)
Number of predicted interactions
Relative order
0
200
400
600
800
1000
1200
0
50
100
150

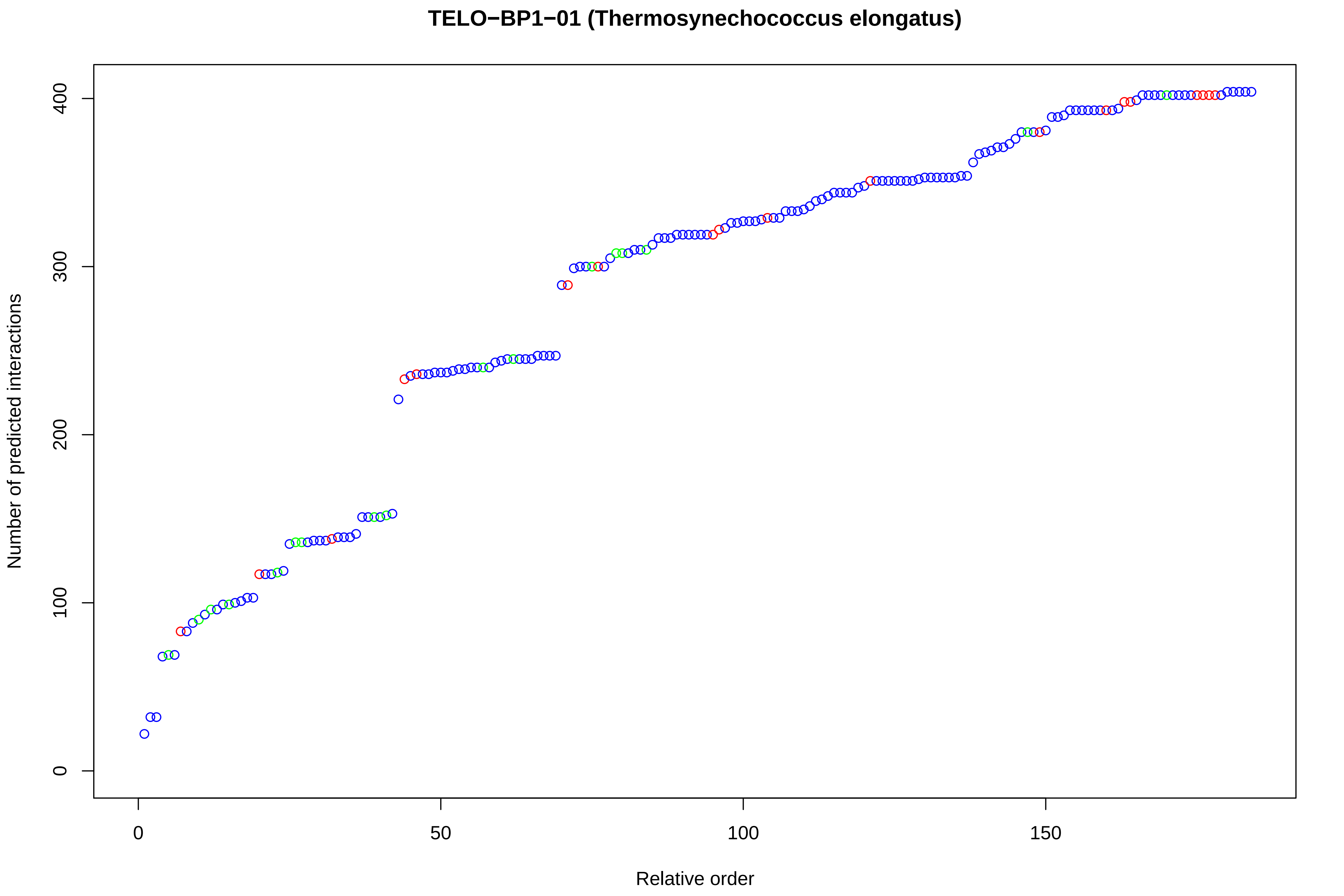
TELO−BP1−01 (Thermosynechococcus elongatus)
Number of predicted interactions
0
100
200
300
400
0
50
100
150
Relative order

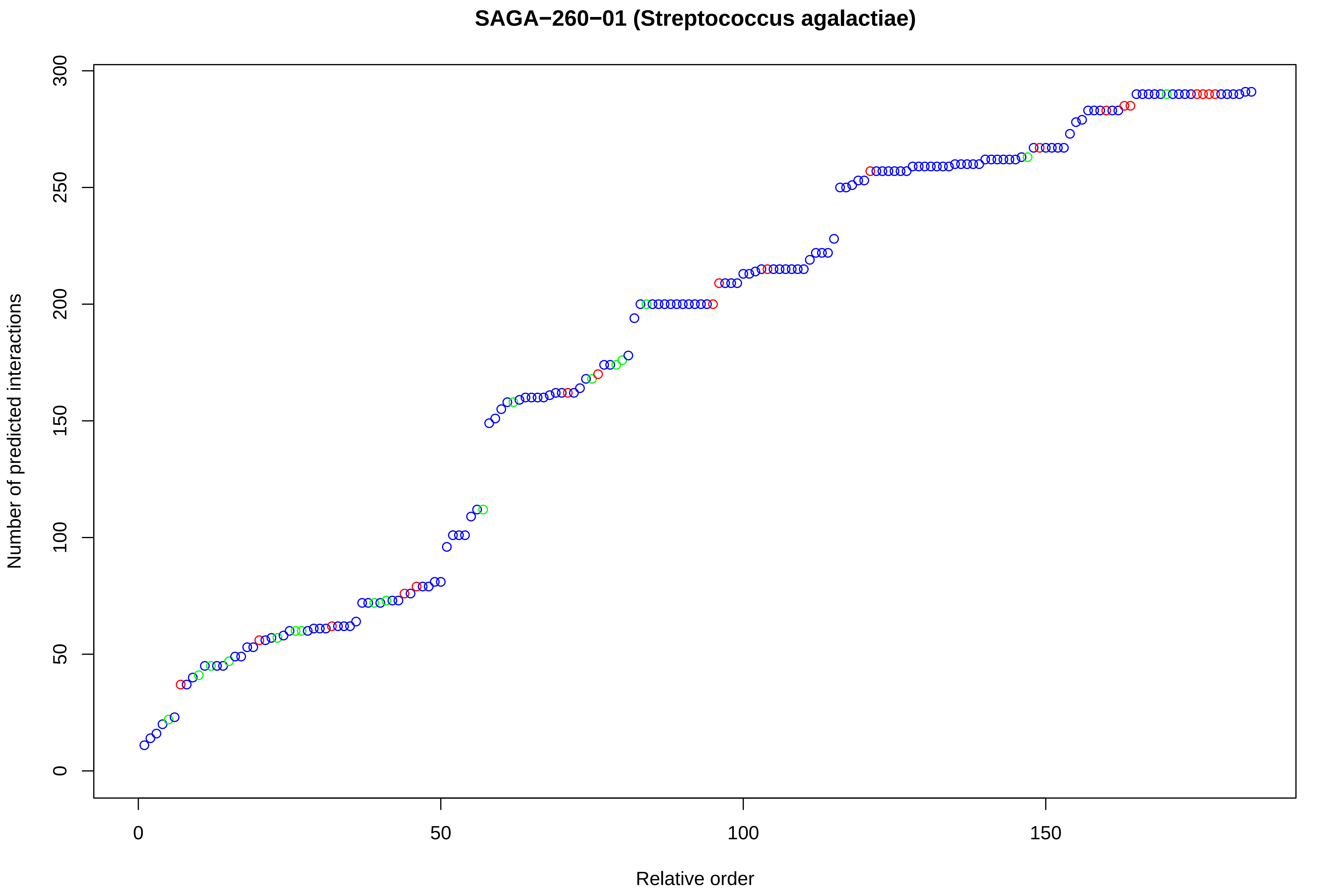

SAGA−260−01 (Streptococcus agalactiae)
Number of predicted interactions
Relative order
0
50
100
150
200
250
300
0
50
100
150

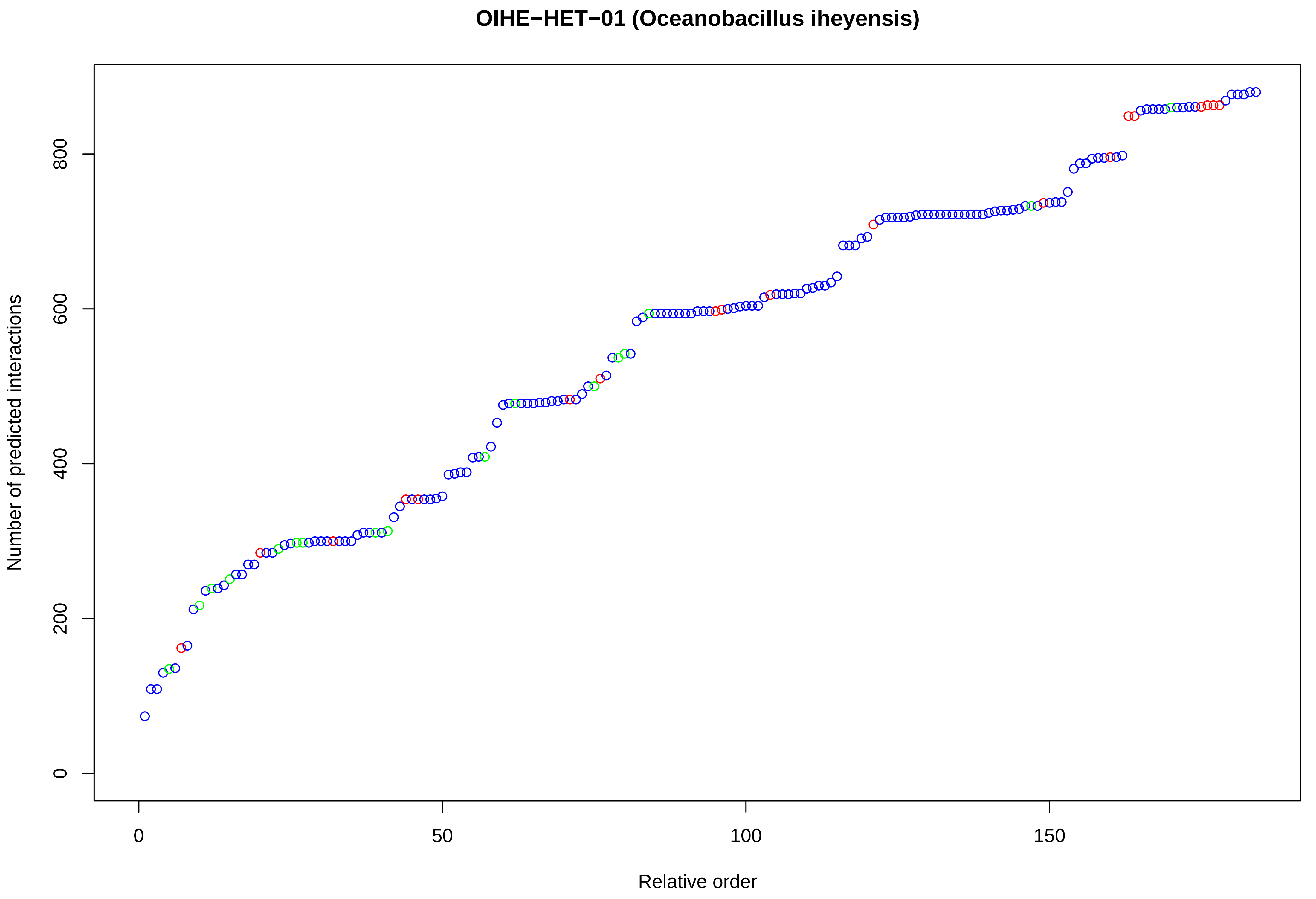
OIHE−HET−01 (Oceanobacillus iheyensis)
Number of predicted interactions
0
200
400
600
800
0
50
100
150
Relative order

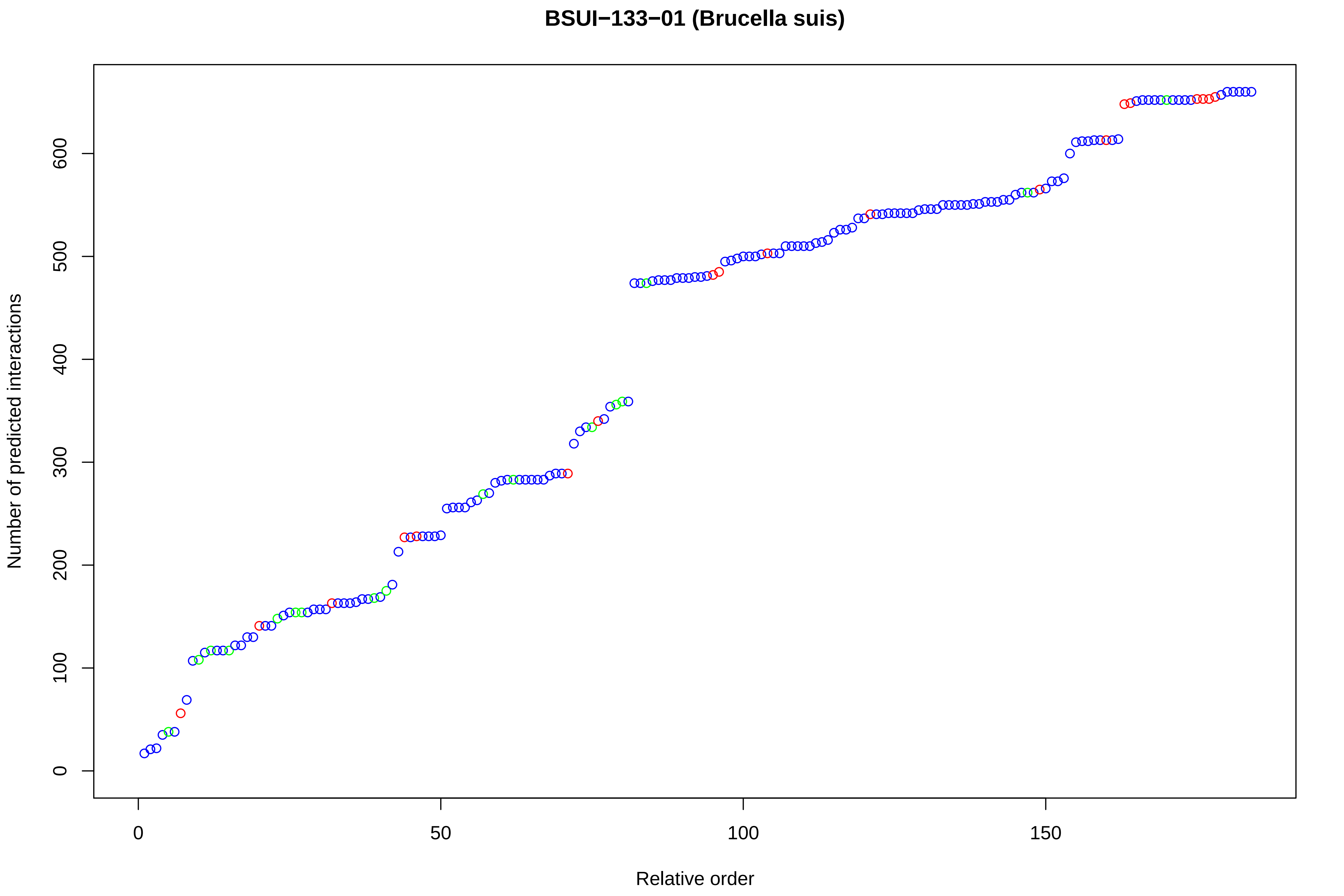

BSUI−133−01 (Brucella suis)
Number of predicted interactions
0
100
200
300
400
500
600
Relative order
0
50
100
150

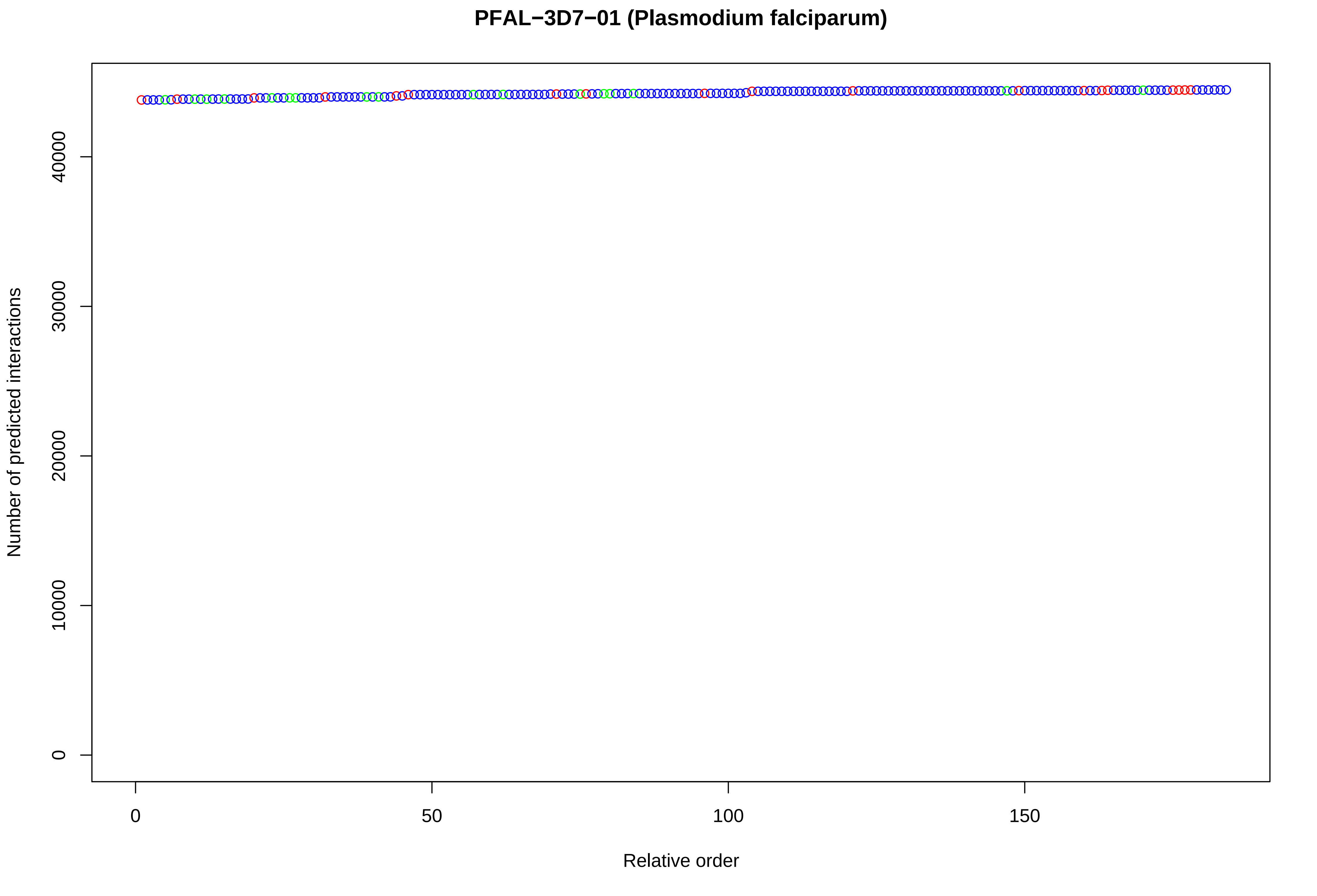
PFAL−3D7−01 (Plasmodium falciparum)
Number of predicted interactions
0
10000
20000
30000
40000
Relative order
0
50
100
150

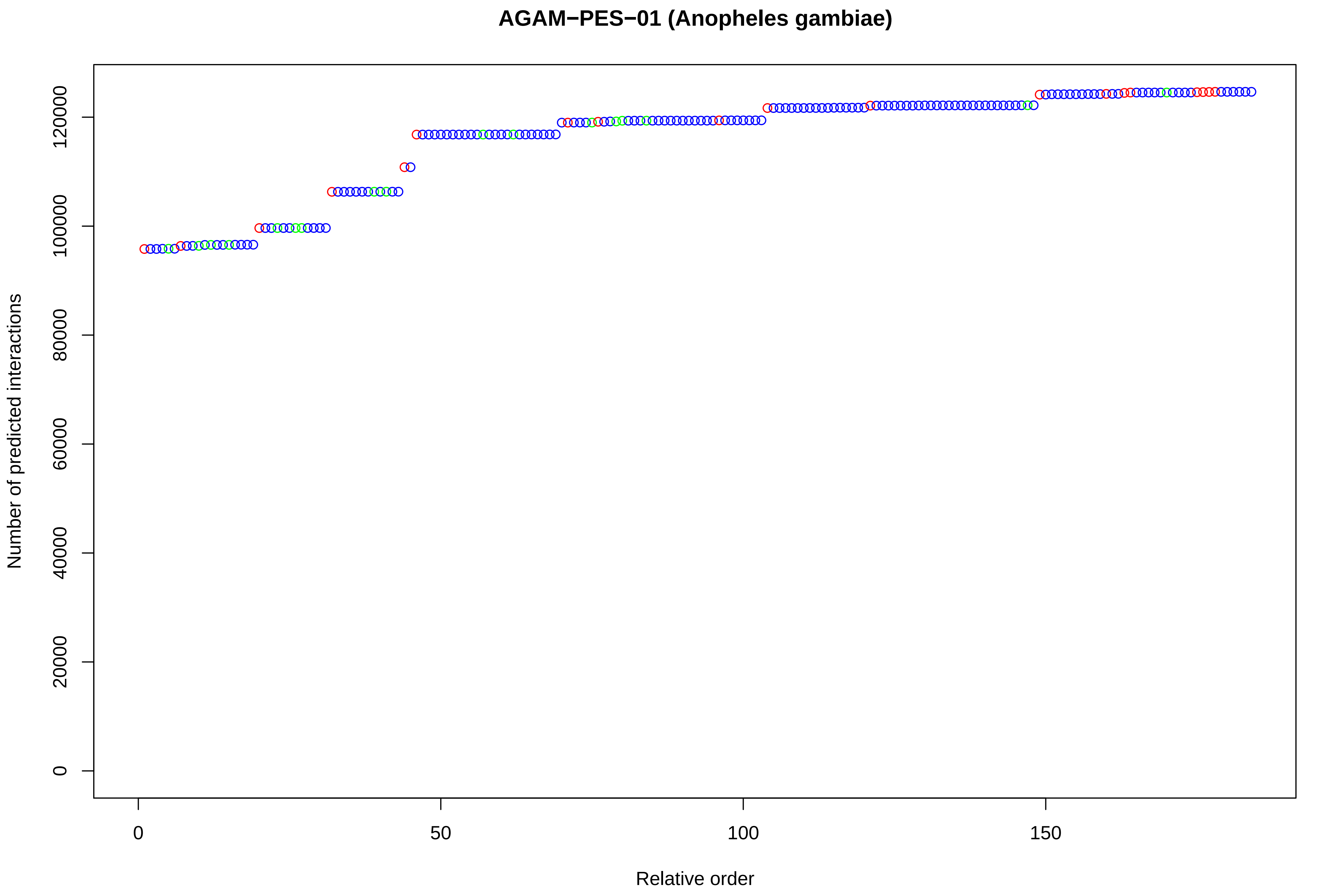

AGAM−PES−01 (Anopheles gambiae)
Number of predicted interactions
0
20000
40000
60000
80000
100000
120000
Relative order
0
50
100
150

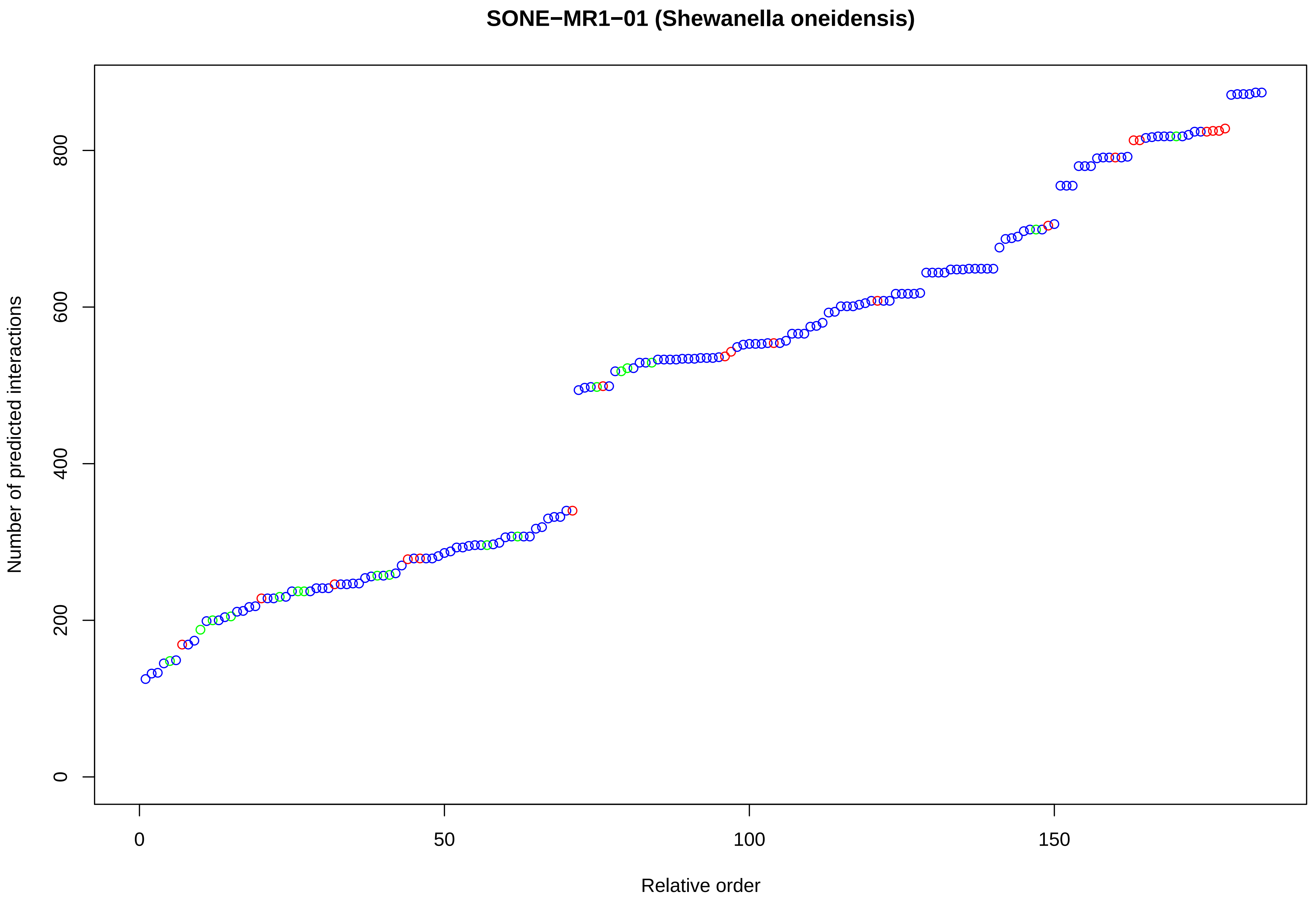
SONE−MR1−01 (Shewanella oneidensis)
Number of predicted interactions
Relative order
0
200
400
600
800
0
50
100
150

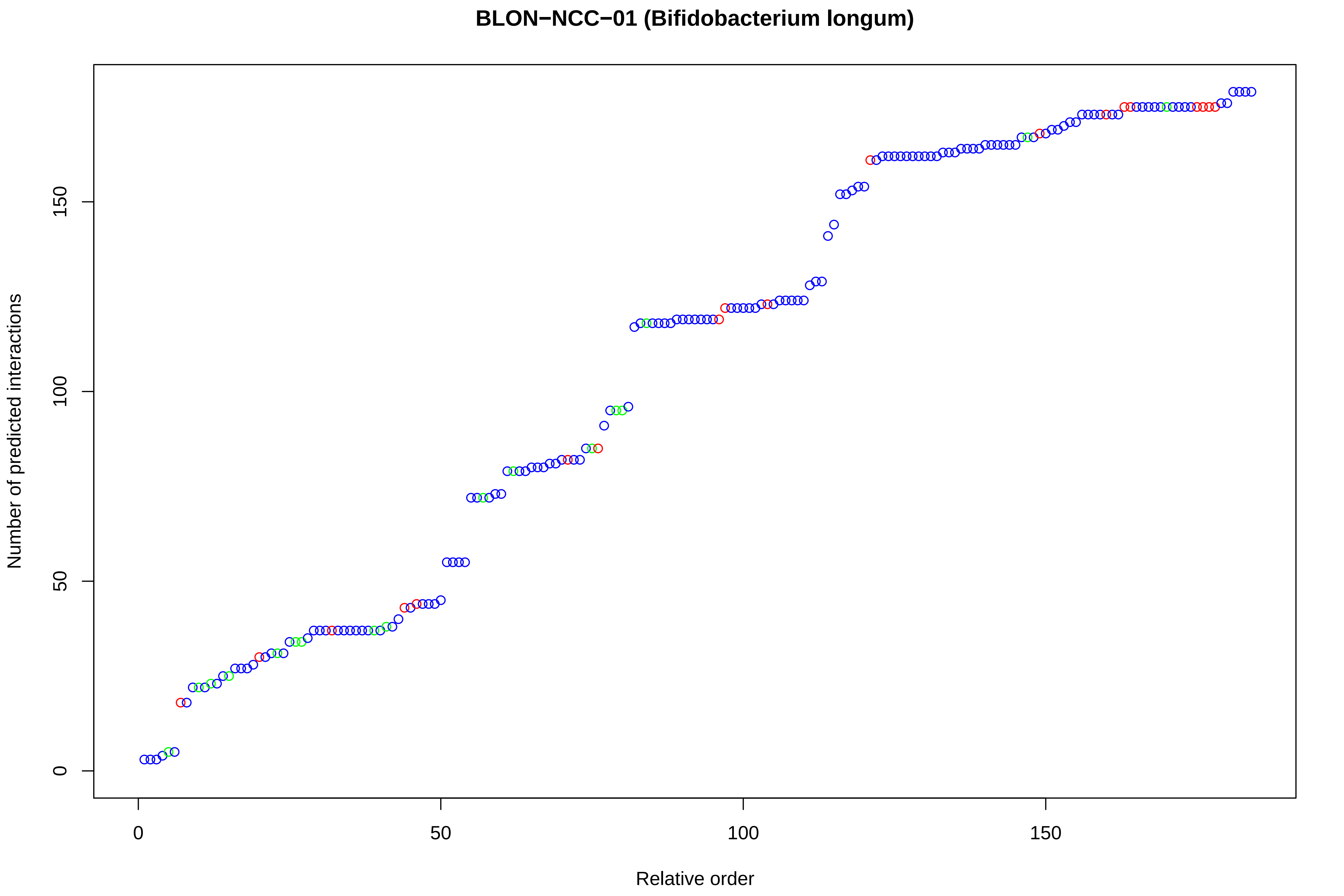

BLON−NCC−01 (Bifidobacterium longum)
Number of predicted interactions
Relative order
0
50
100
150
0
50
100
150

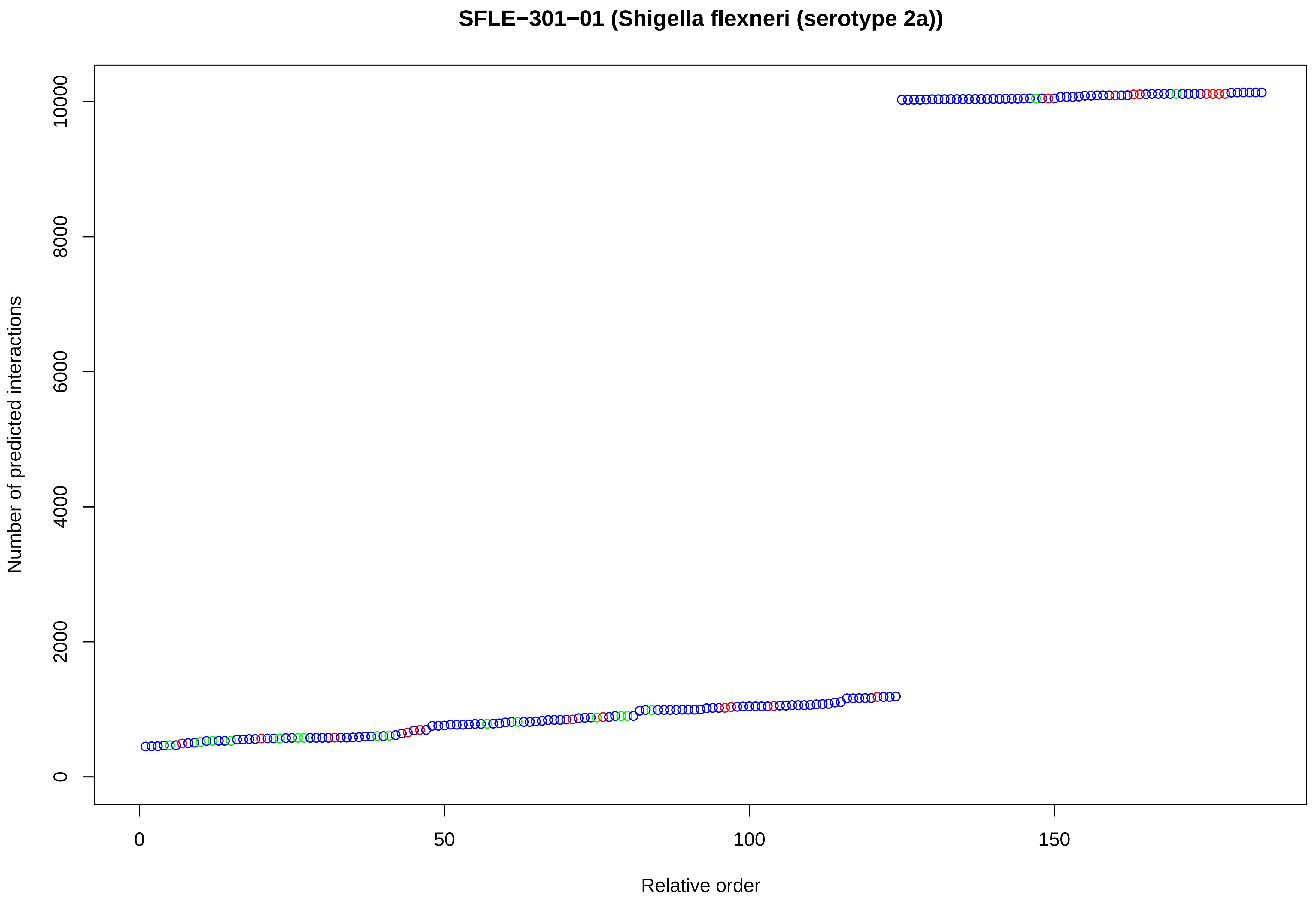

SFLE−301−01 (Shigella flexneri (serotype 2a))
Number of predicted interactions
Relative order
0
2000
4000
6000
8000
10000
0
50
100
150

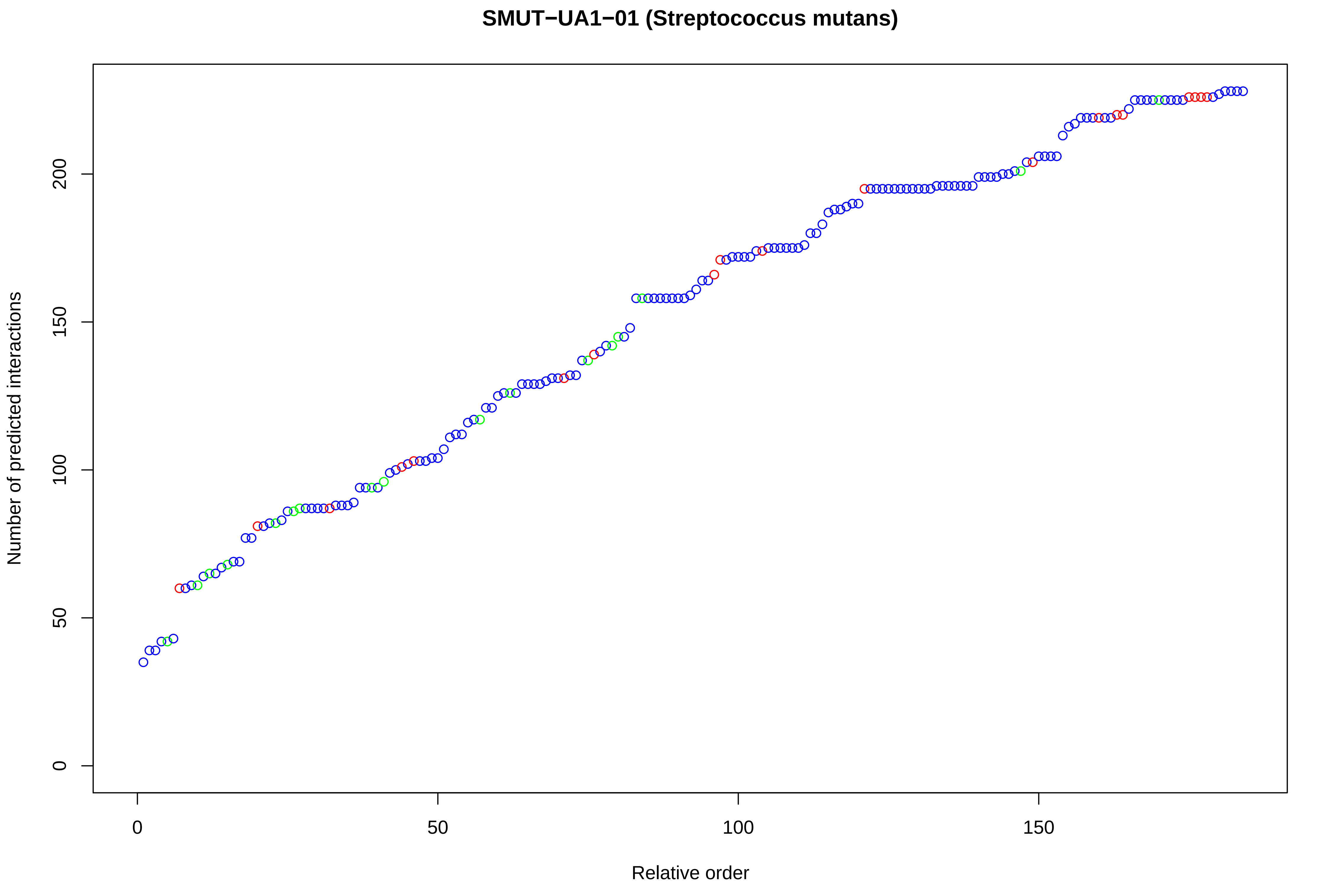

SMUT−UA1−01 (Streptococcus mutans)
Number of predicted interactions
Relative order
0
50
100
150
200
0
50
100
150

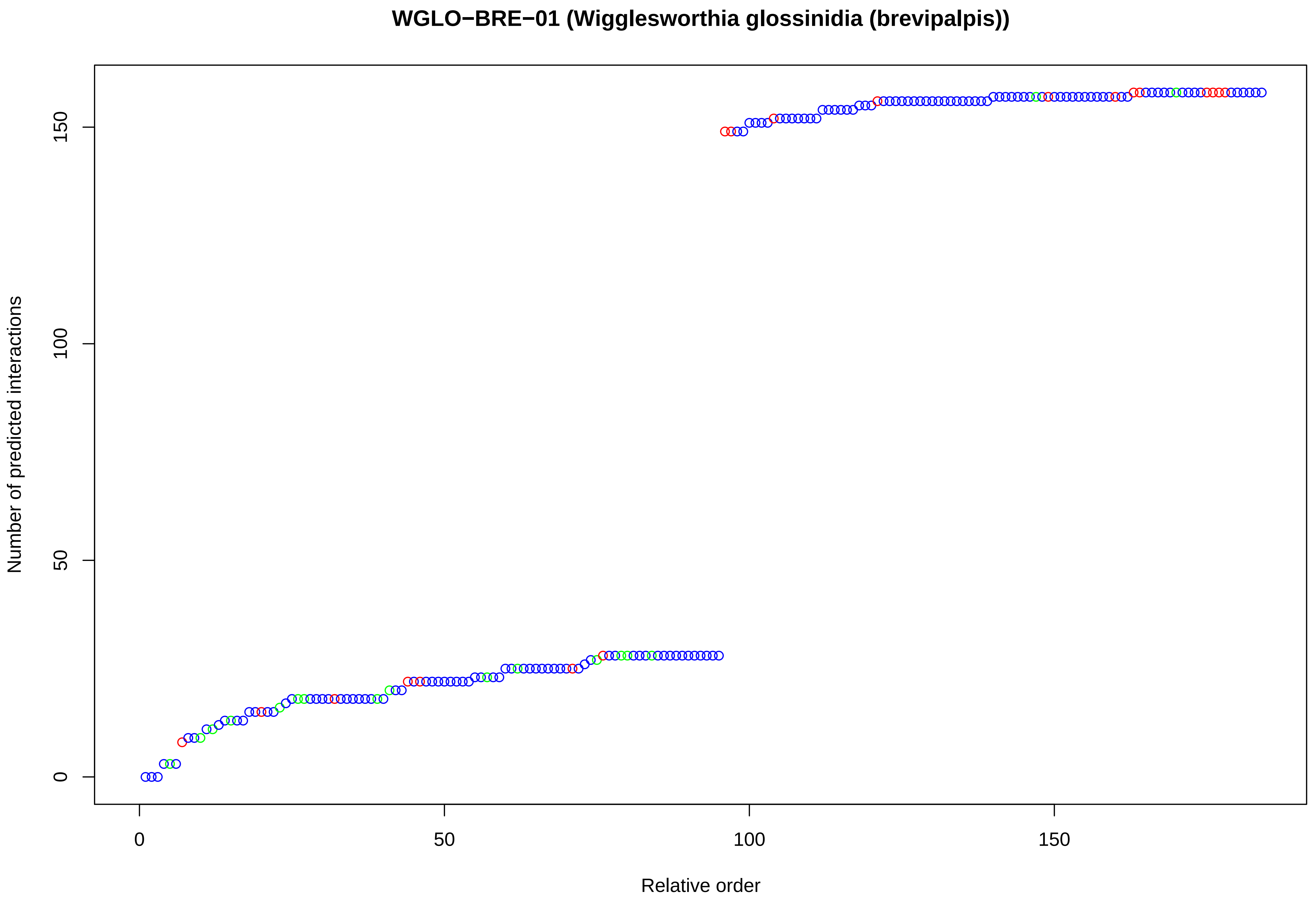

WGLO−BRE−01 (Wigglesworthia glossinidia (brevipalpis))
Number of predicted interactions
0
50
100
150
Relative order
0
50
100
150

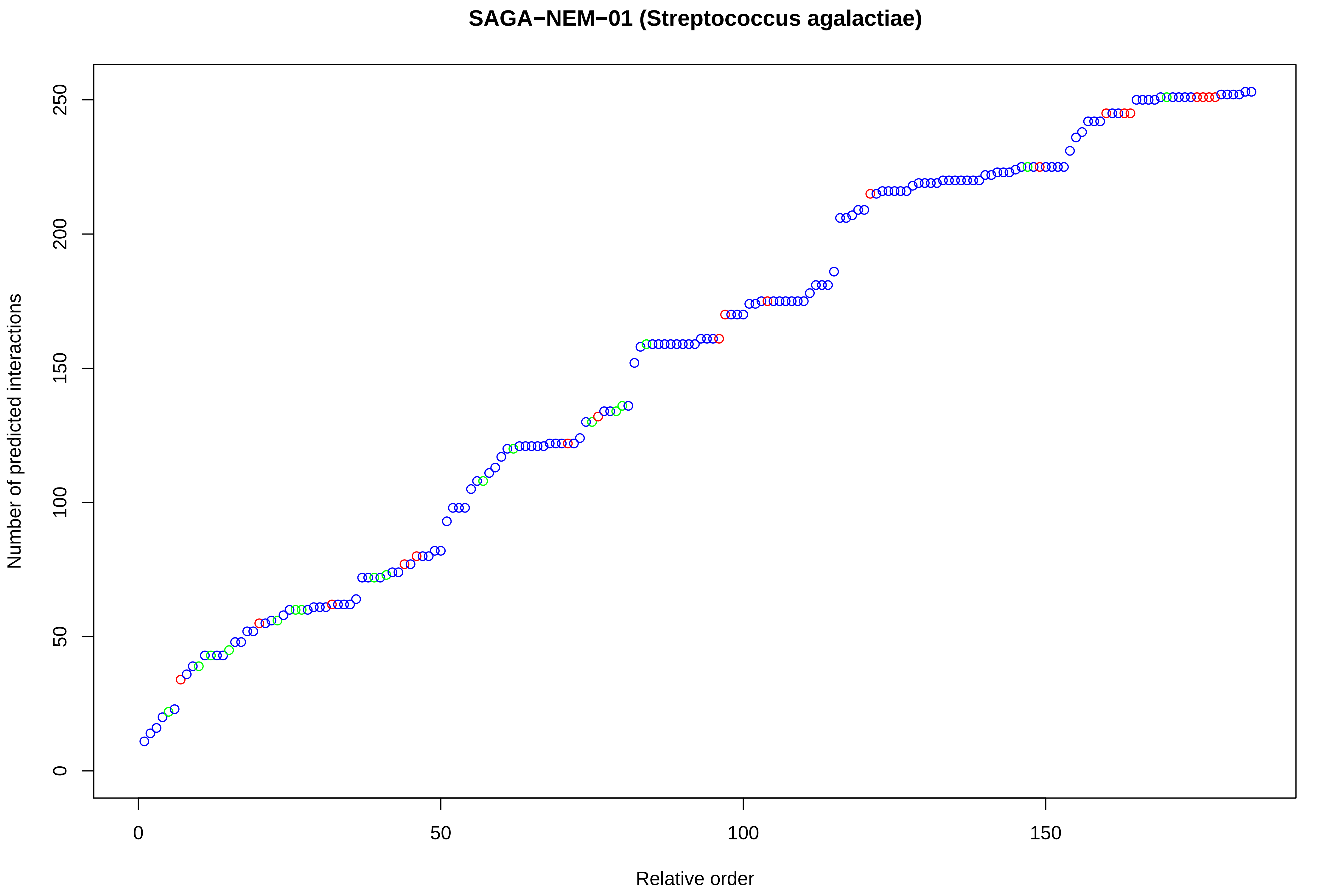

SAGA−NEM−01 (Streptococcus agalactiae)
Number of predicted interactions
Relative order
0
50
100
150
200
250
0
50
100
150

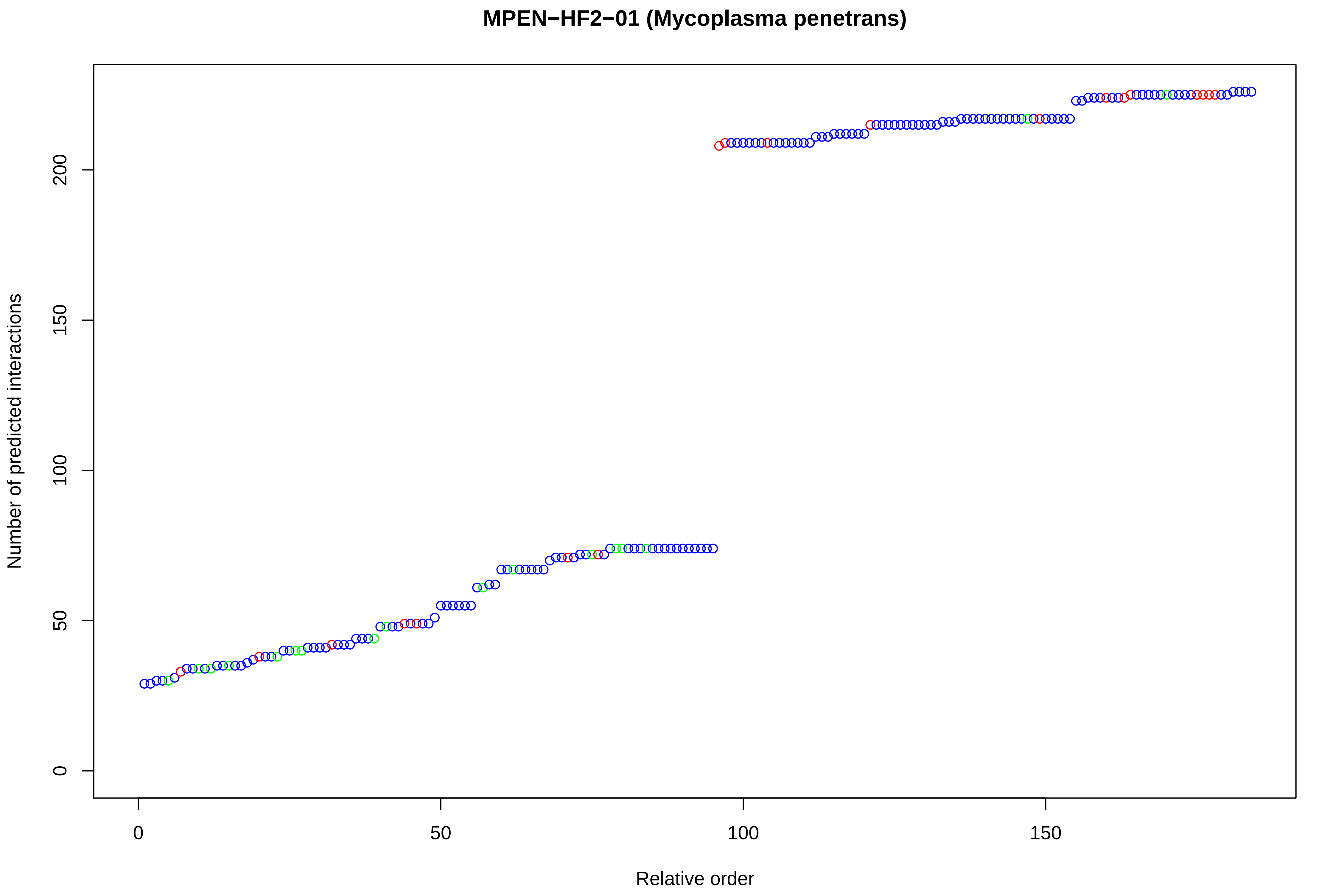
MPEN−HF2−01 (Mycoplasma penetrans)
Number of predicted interactions
0
50
100
150
200
0
50
100
150
Relative order

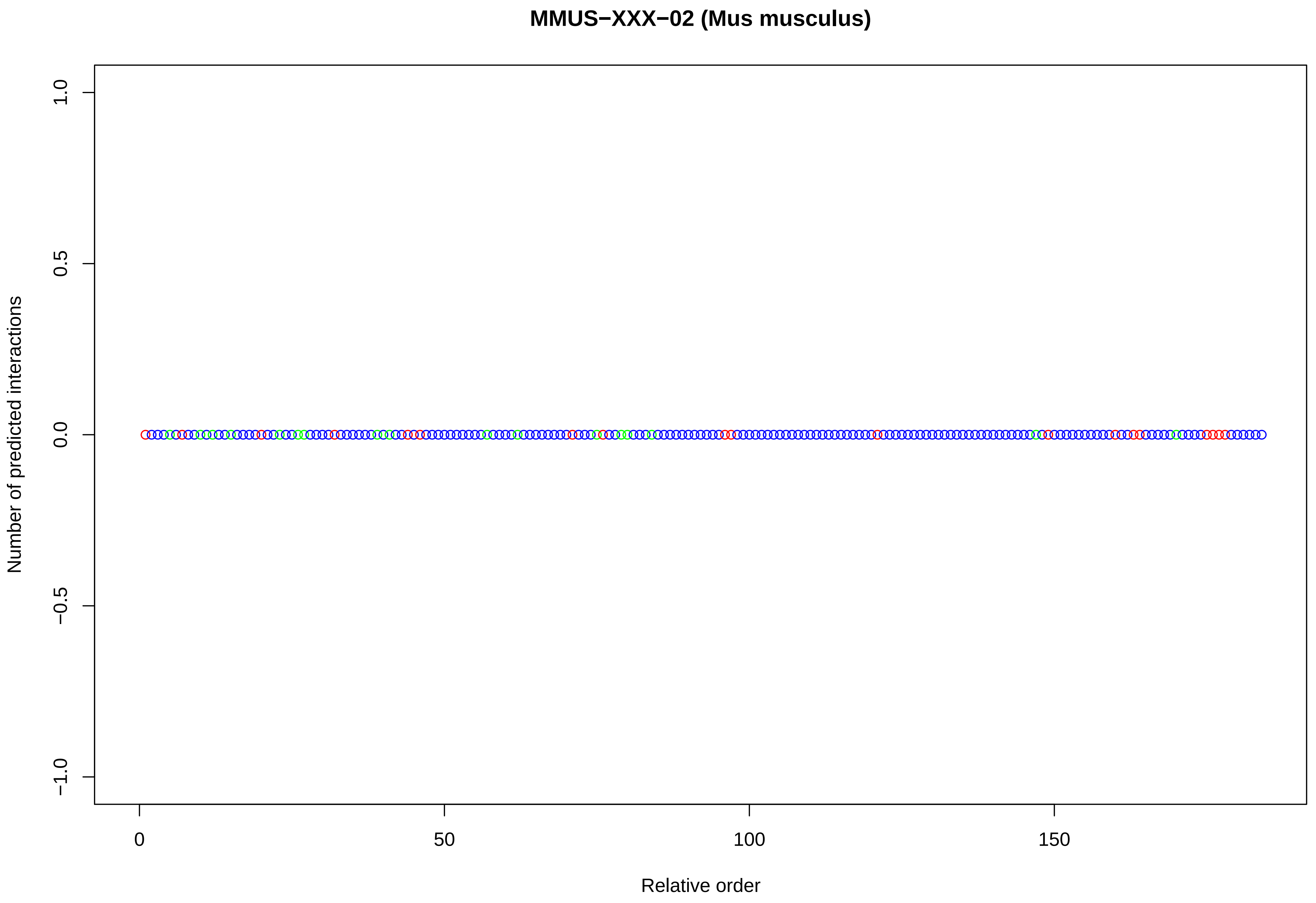
MMUS−XXX−02 (Mus musculus)
Number of predicted interactions
Relative order
1.0
0.5
0.0
−0.5
−1.0
0
50
100
150

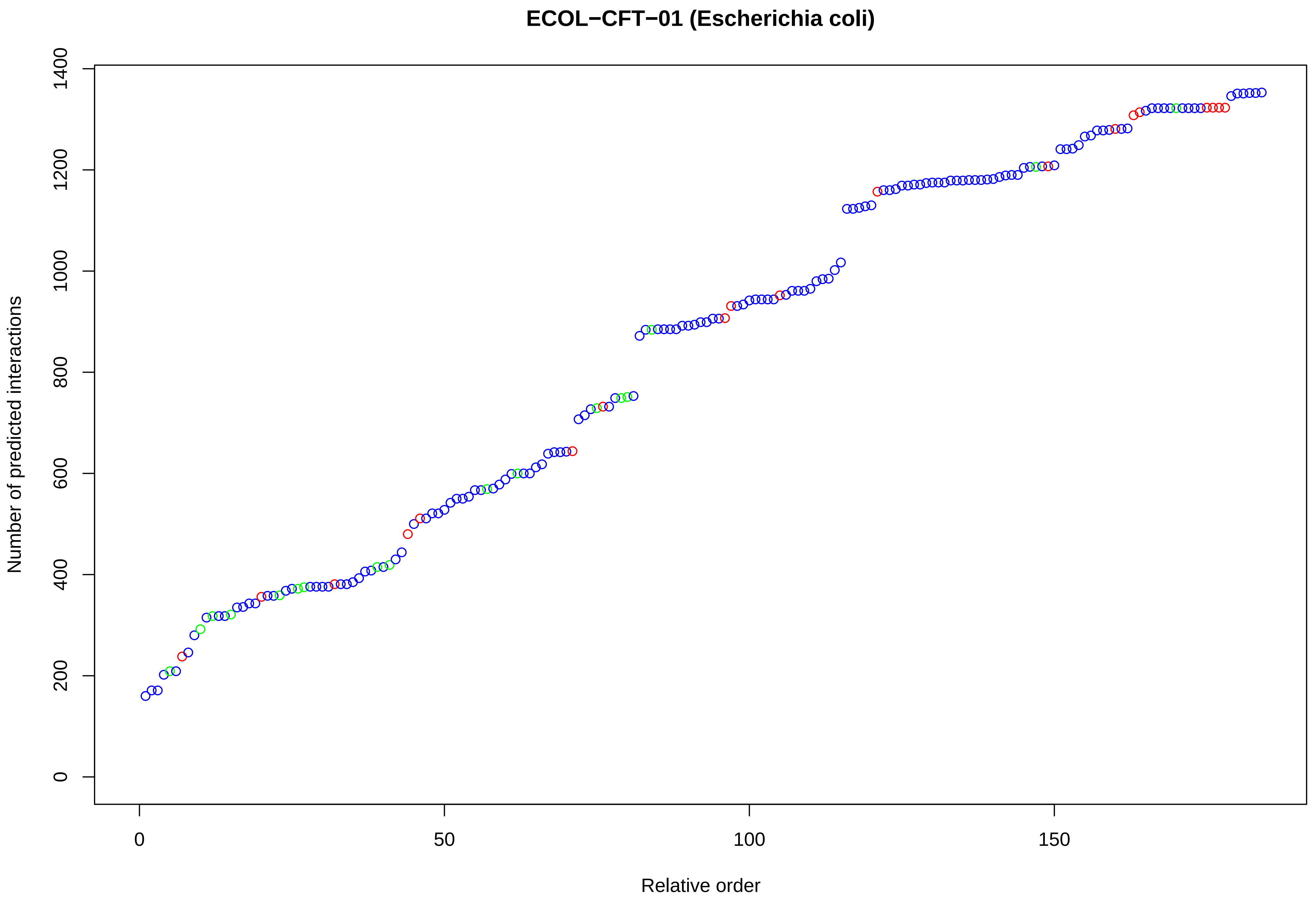
ECOL−CFT−01 (Escherichia coli)
Number of predicted interactions
0
200
400
600
800
1000
1200
1400
0
50
100
150
Relative order

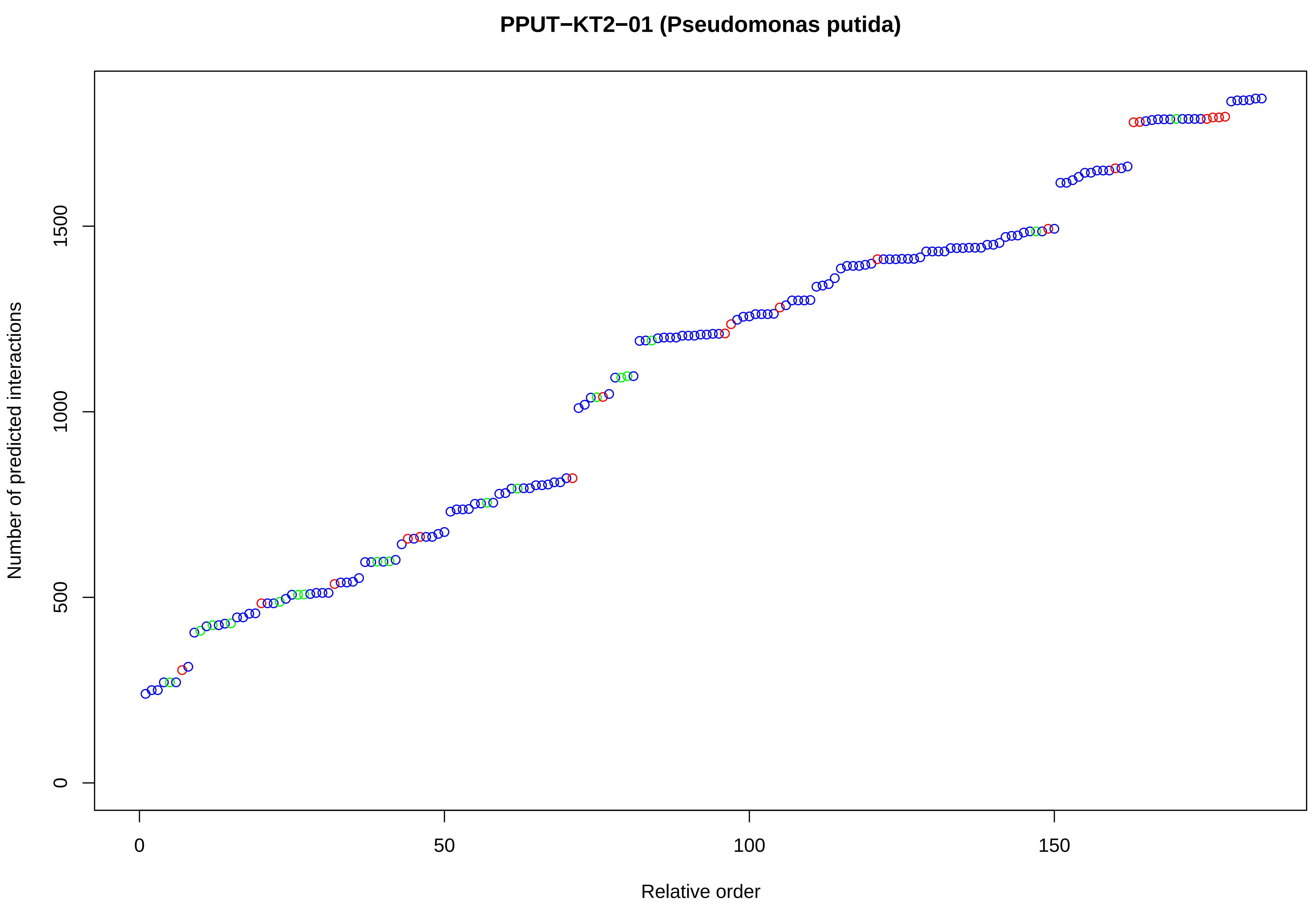
PPUT−KT2−01 (Pseudomonas putida)
Number of predicted interactions
0
500
1000
1500
Relative order
0
50
100
150

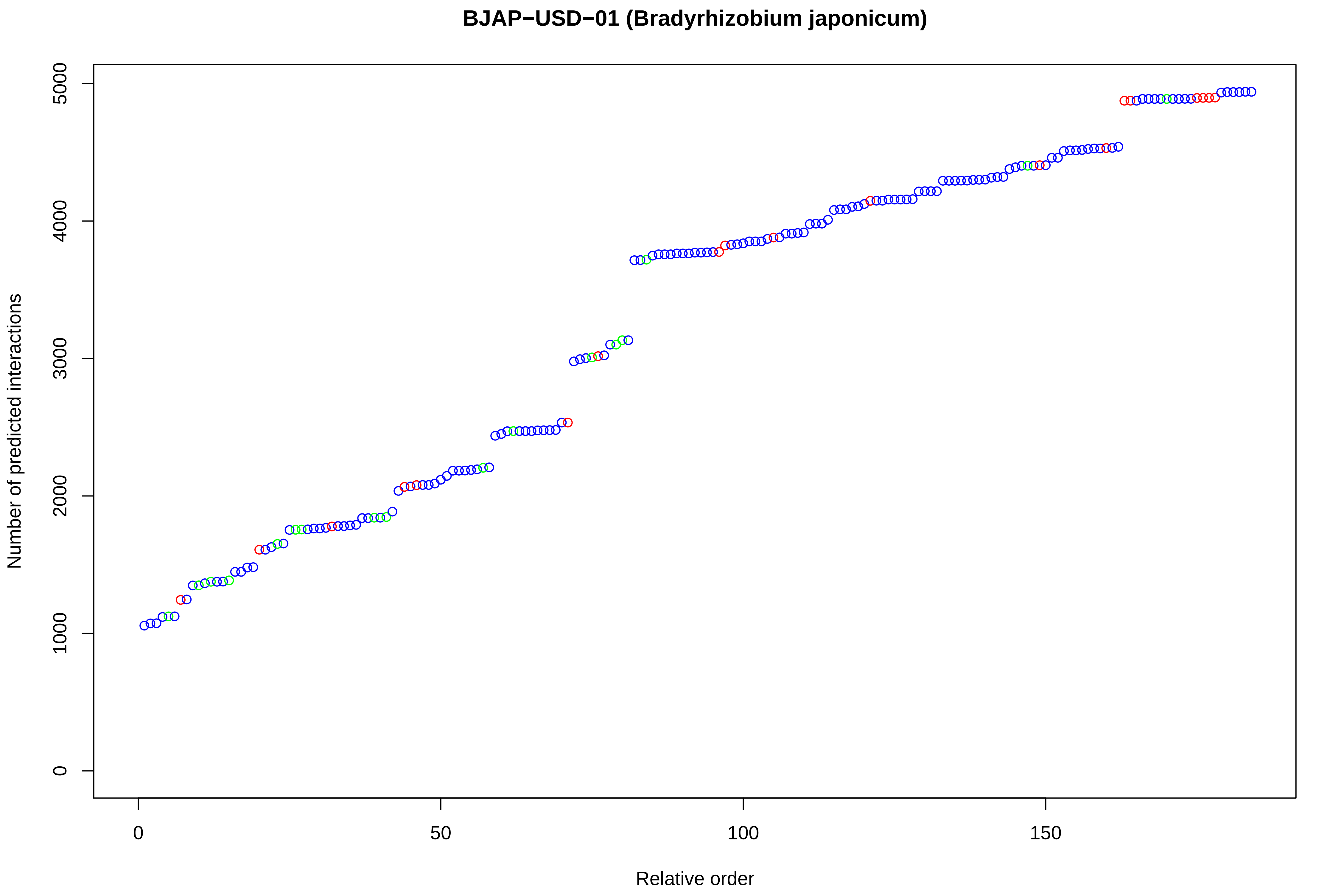

BJAP−USD−01 (Bradyrhizobium japonicum)
Number of predicted interactions
Relative order
0
1000
2000
3000
4000
5000
0
50
100
150

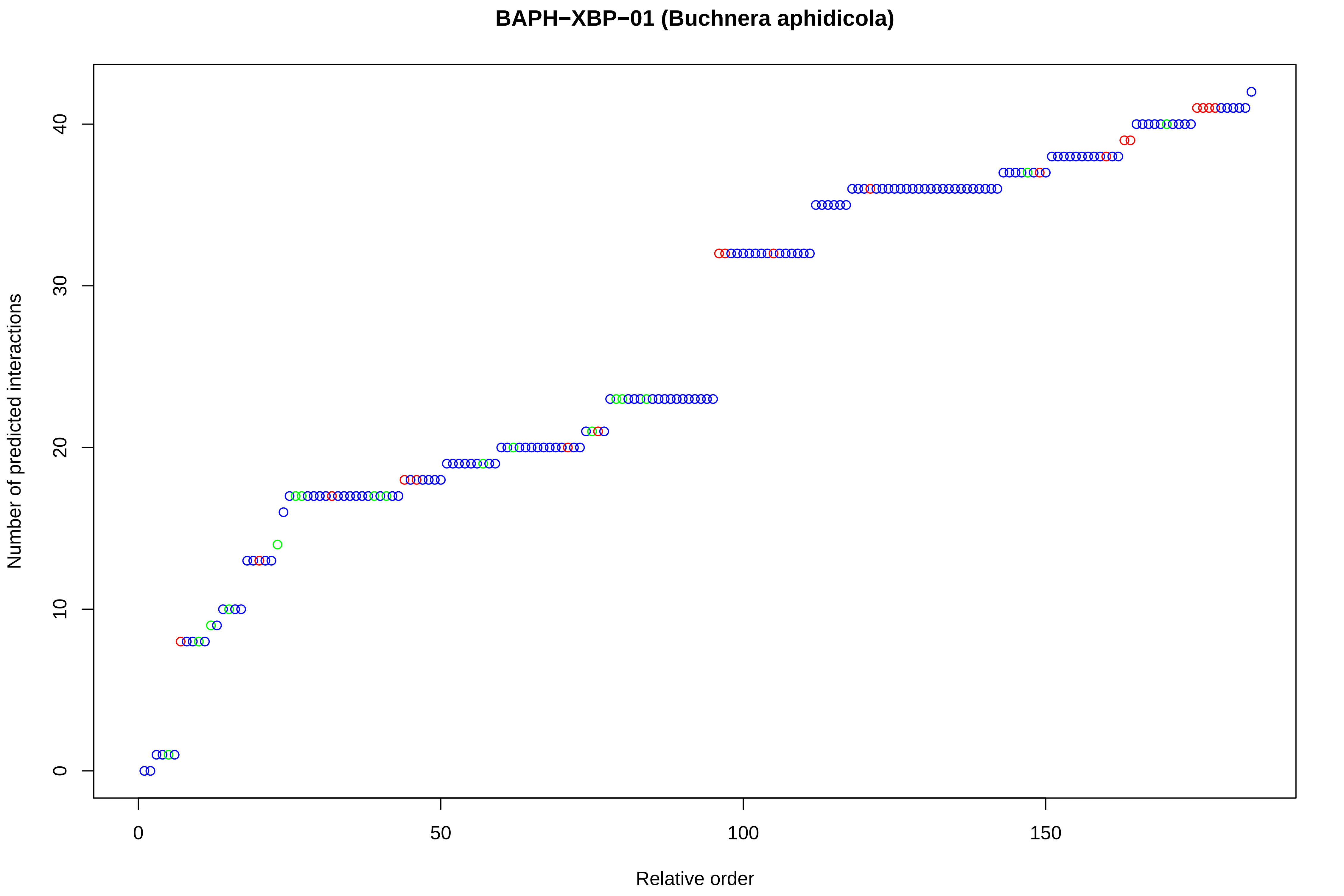

BAPH−XBP−01 (Buchnera aphidicola)
Number of predicted interactions
Relative order
0
10
20
30
40
0
50
100
150

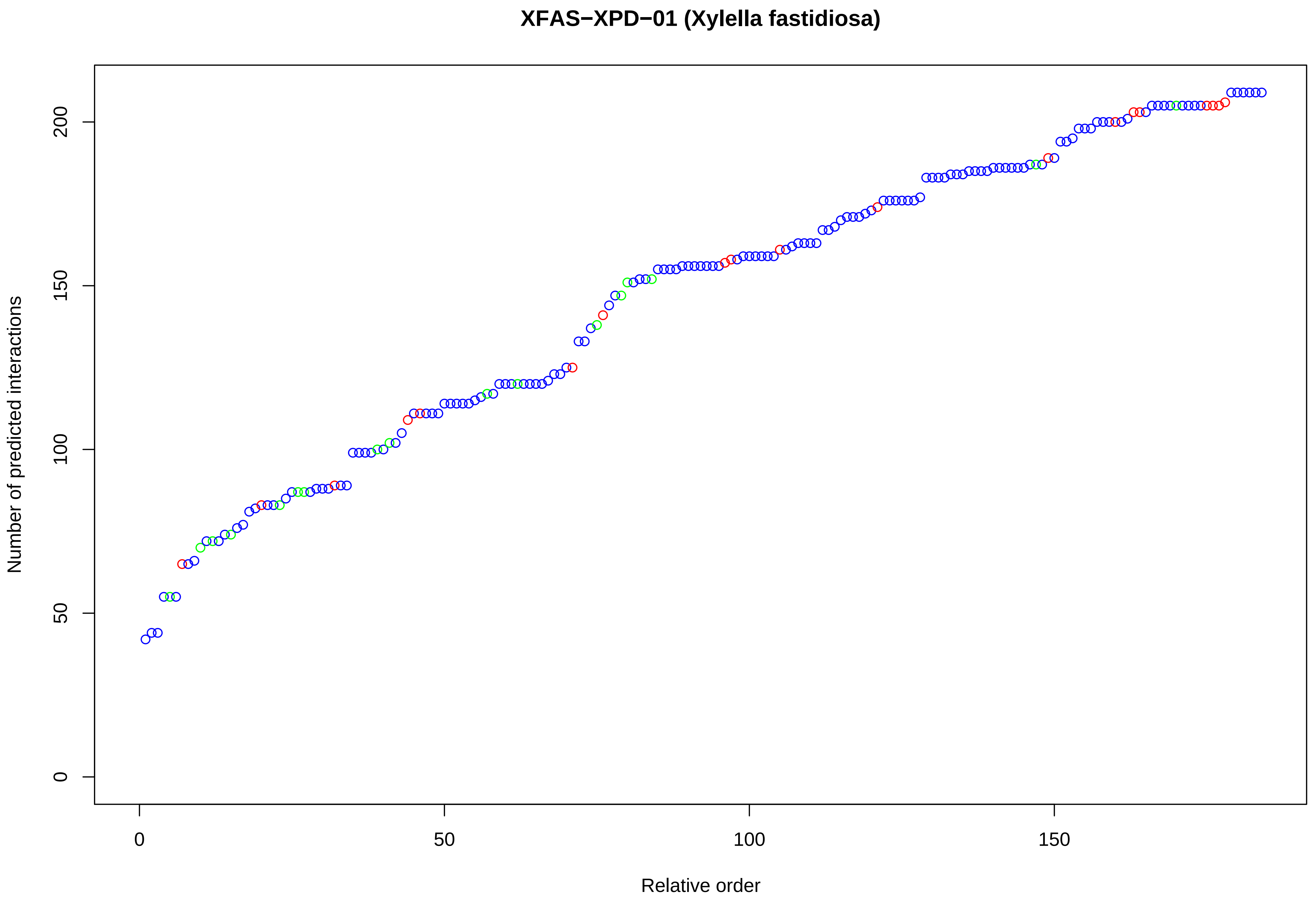
XFAS−XPD−01 (Xylella fastidiosa)
Number of predicted interactions
0
50
100
150
200
Relative order
0
50
100
150

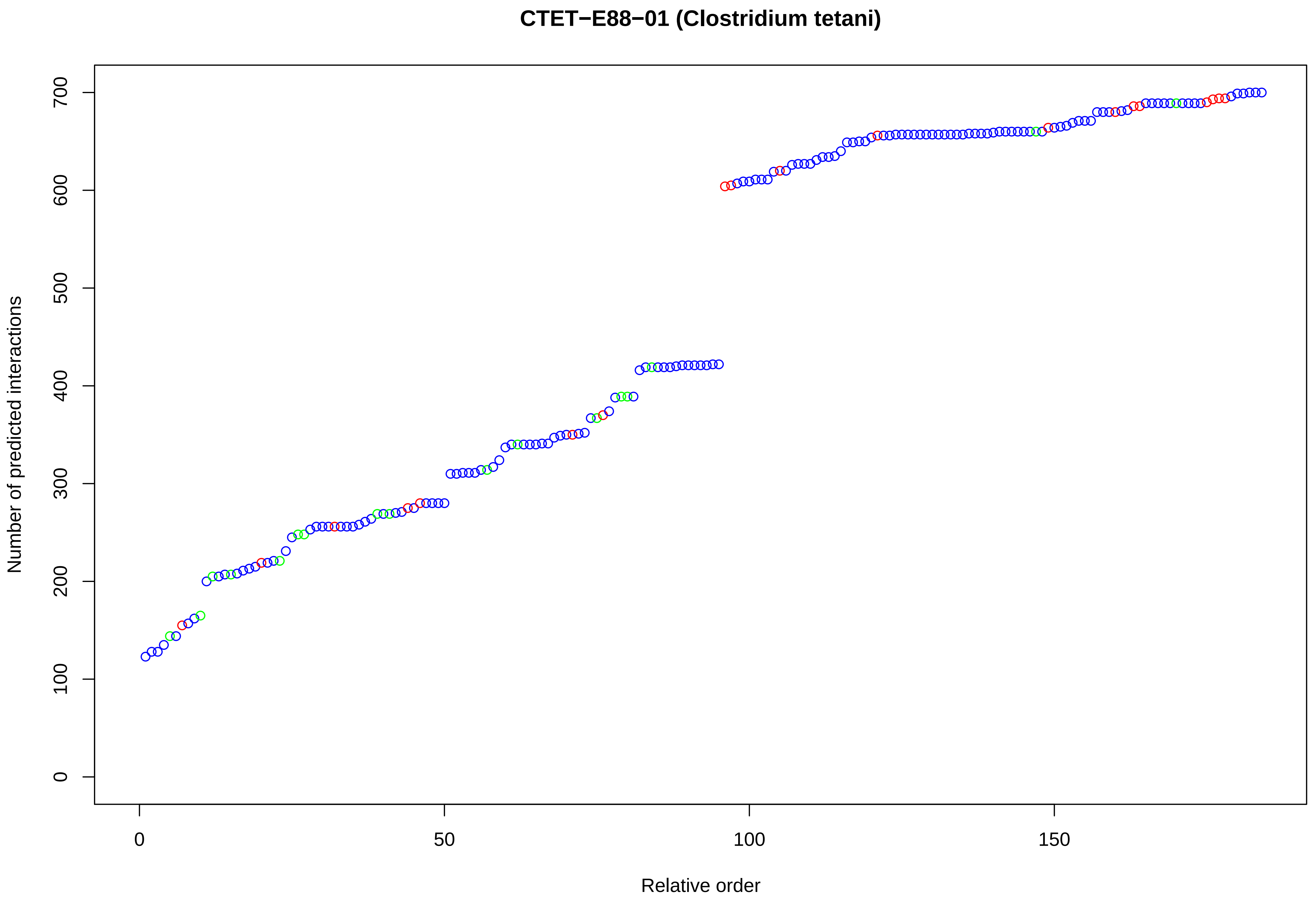
CTET−E88−01 (Clostridium tetani)
Number of predicted interactions
Relative order
0
100
200
300
400
500
600
700
0
50
100
150

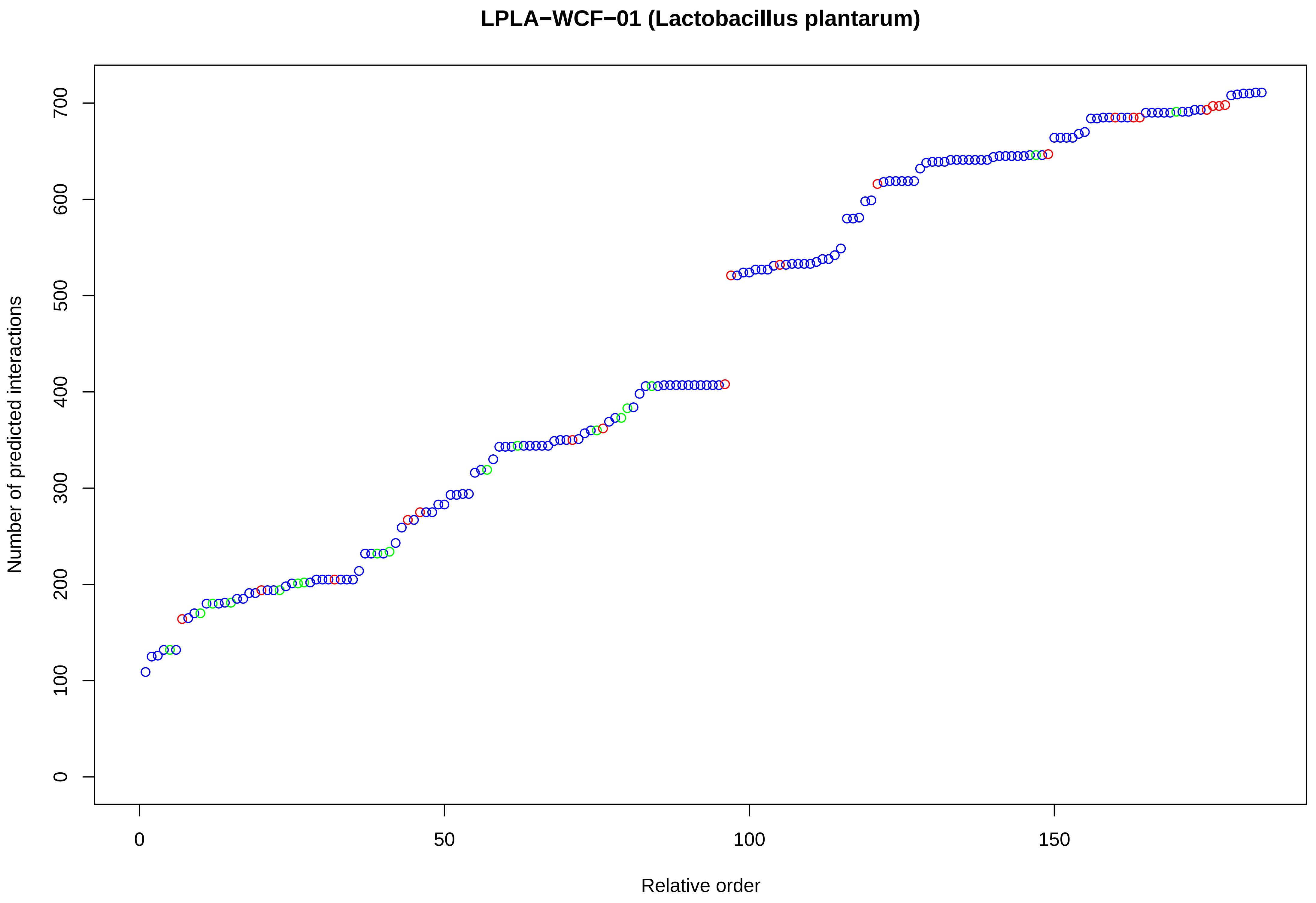

LPLA−WCF−01 (Lactobacillus plantarum)
Number of predicted interactions
0
100
200
300
400
500
600
700
Relative order
0
50
100
150

TWHI−TW0−01 (Tropheryma whipplei)

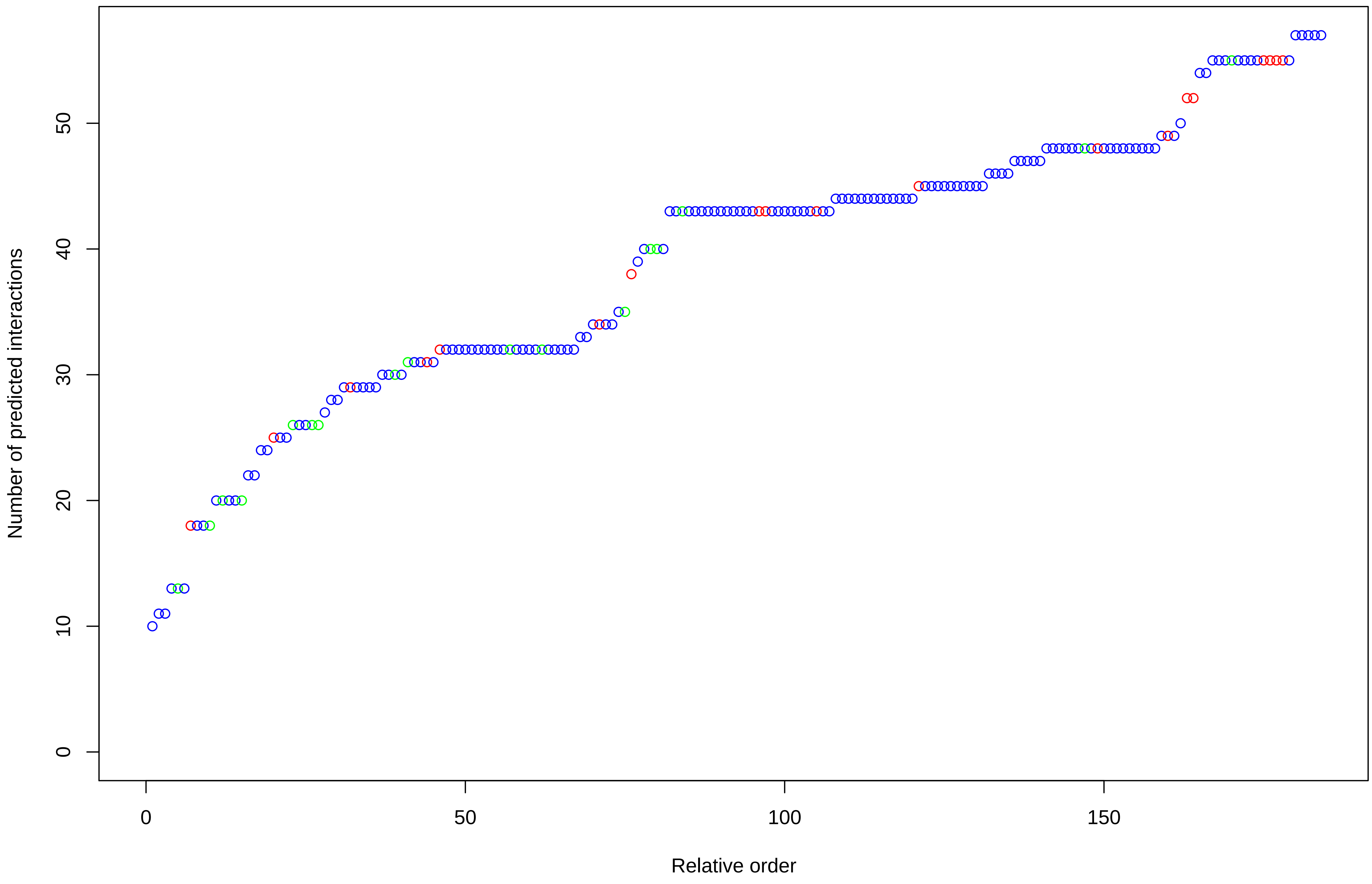

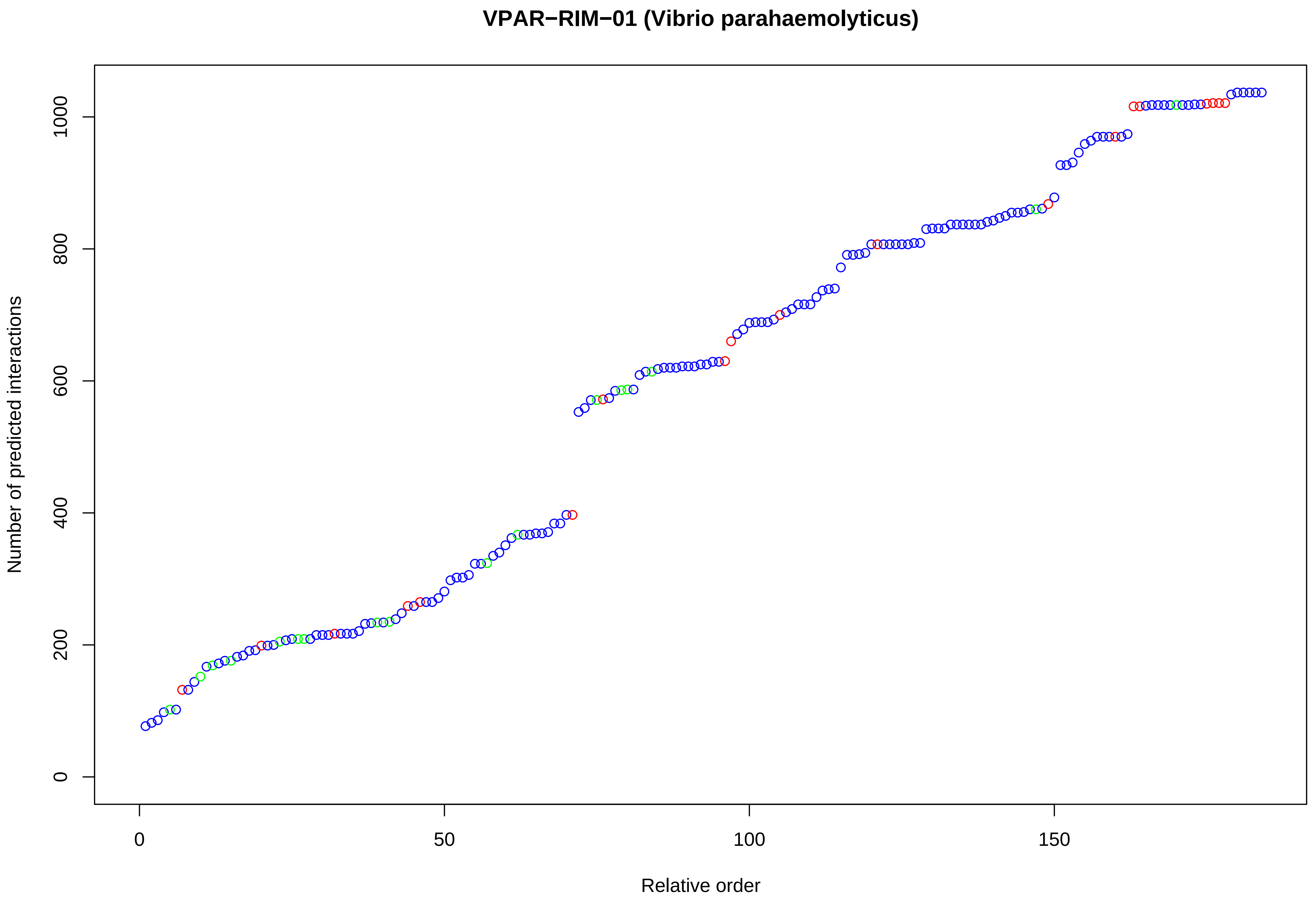

VPAR−RIM−01 (Vibrio parahaemolyticus)
Number of predicted interactions
Relative order
0
200
400
600
800
1000
0
50
100
150

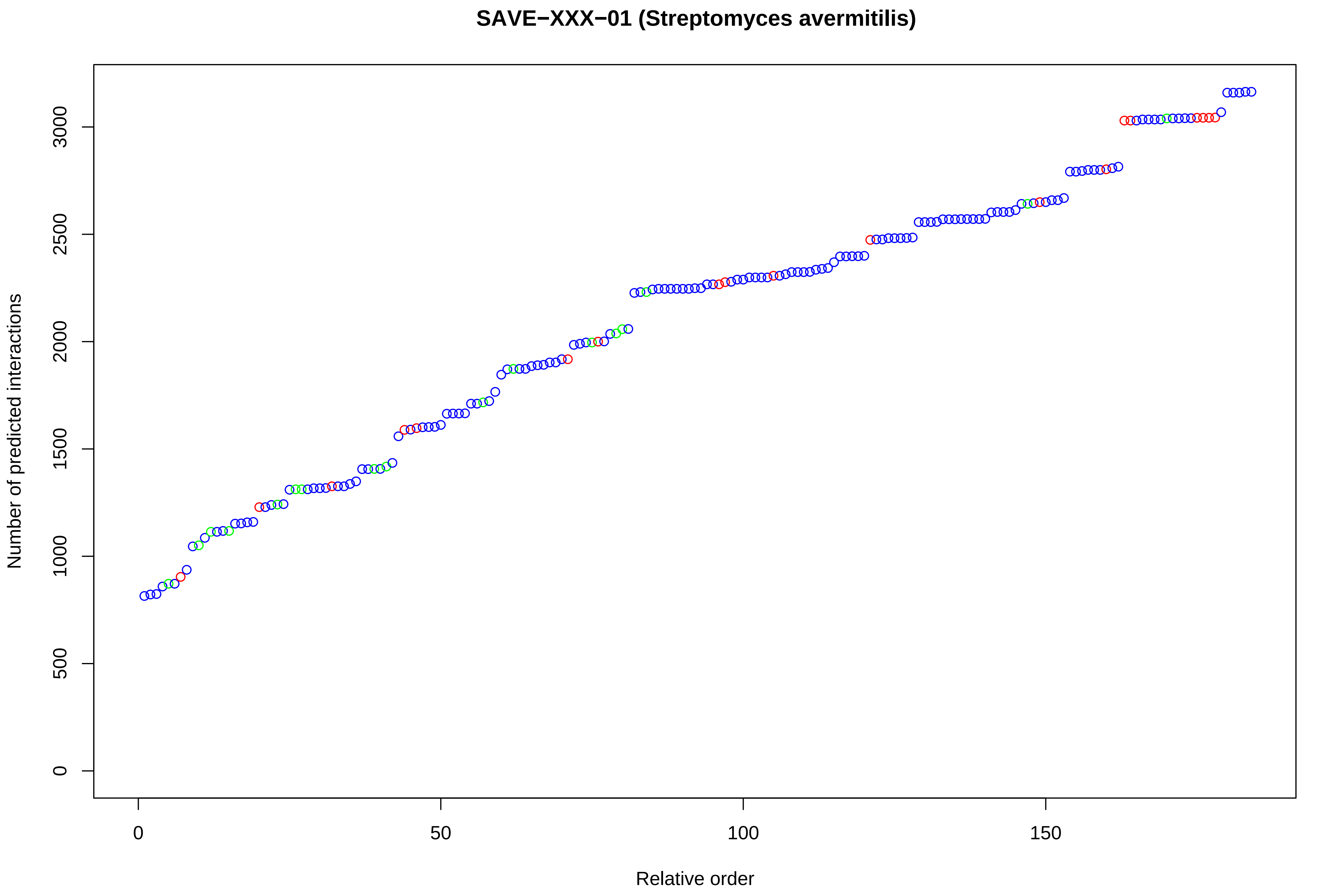
SAVE−XXX−01 (Streptomyces avermitilis)
Number of predicted interactions
0
500
1000
1500
2000
2500
3000
Relative order
0
50
100
150

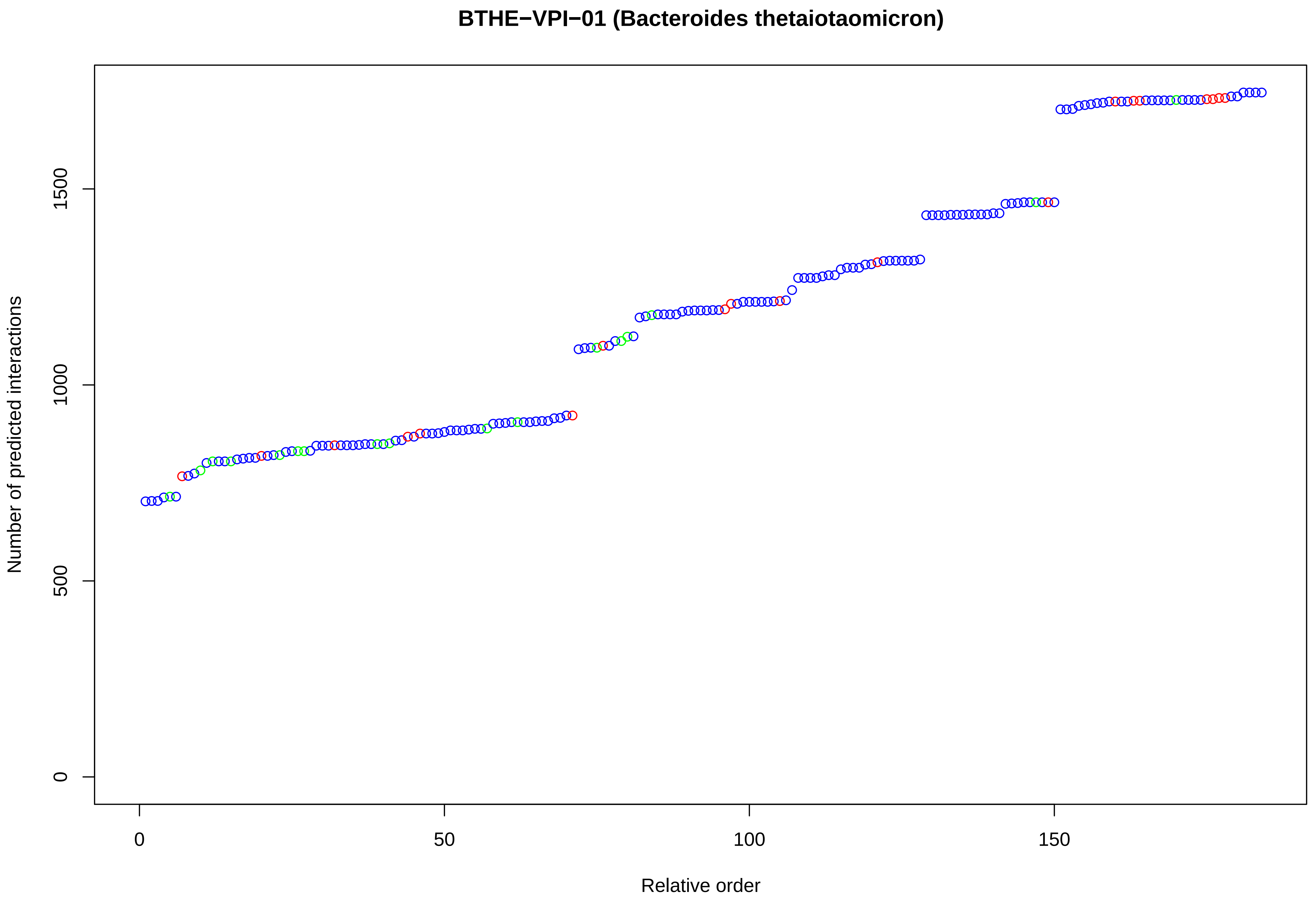
BTHE−VPI−01 (Bacteroides thetaiotaomicron)
Number of predicted interactions
Relative order
0
500
1000
1500
0
50
100
150

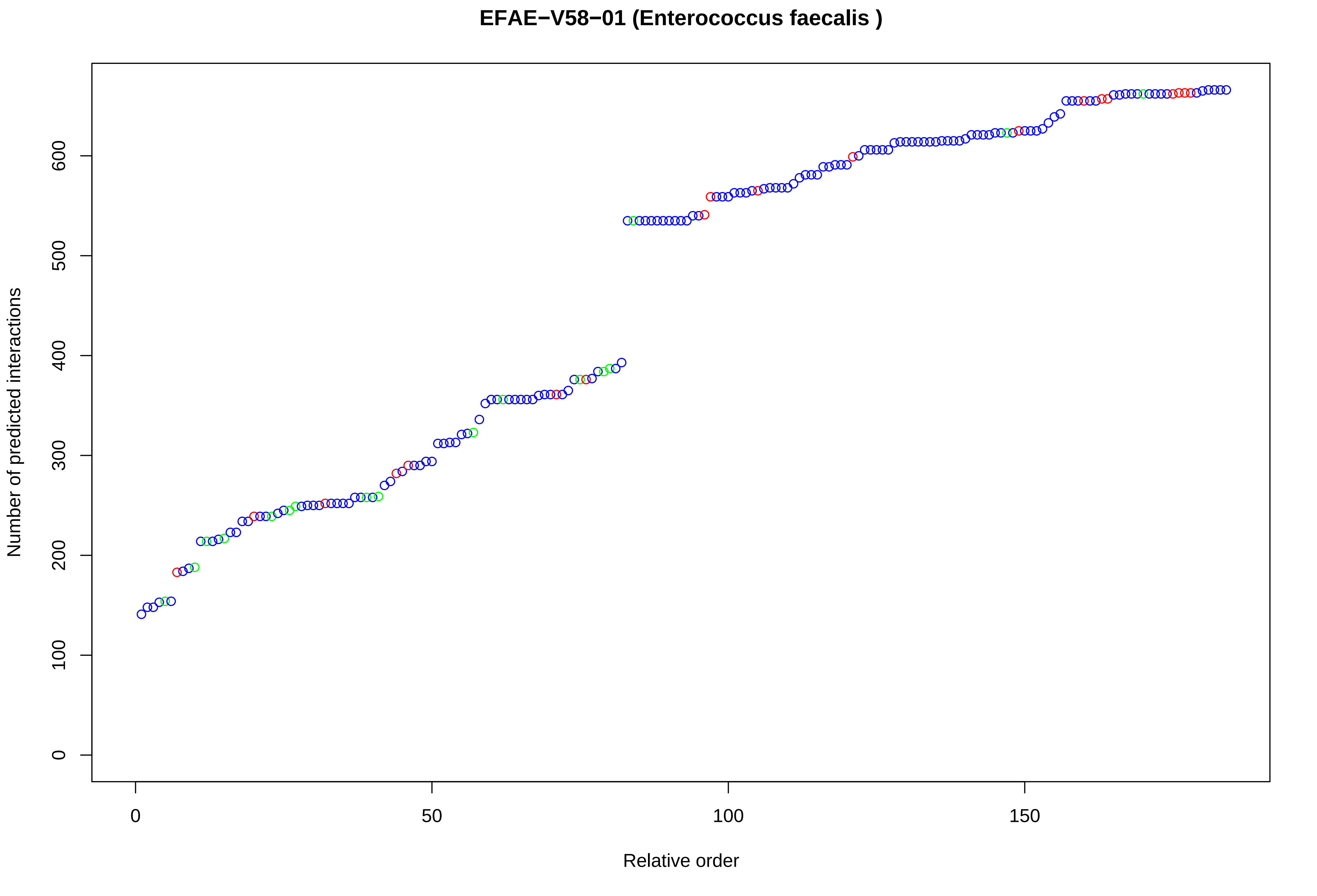

EFAE−V58−01 (Enterococcus faecalis)
Number of predicted interactions
Relative order
0
100
200
300
400
500
600
0
50
100
150

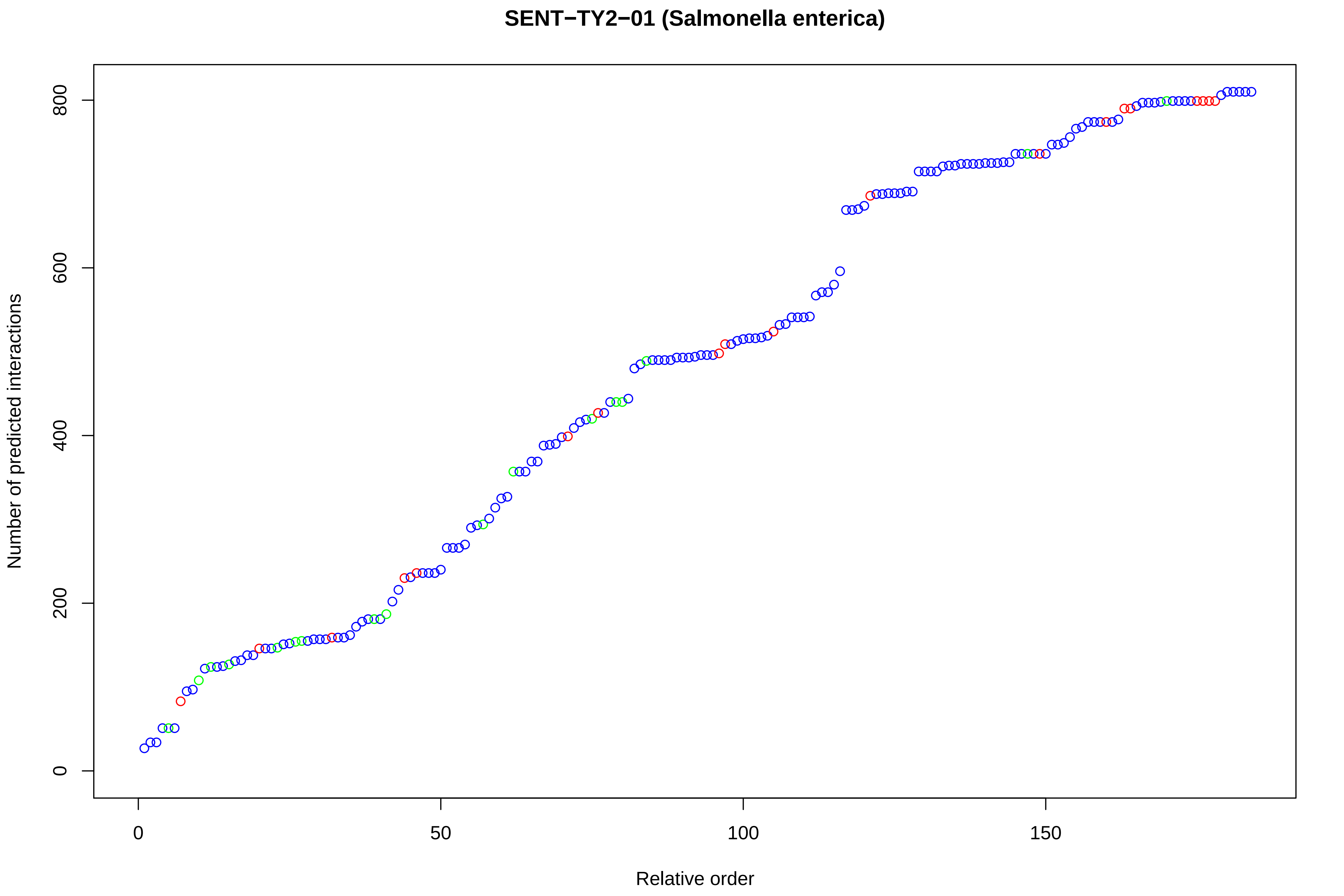

SENT−TY2−01 (Salmonella enterica)
Number of predicted interactions
Relative order
0
200
400
600
800
0
50
100
150

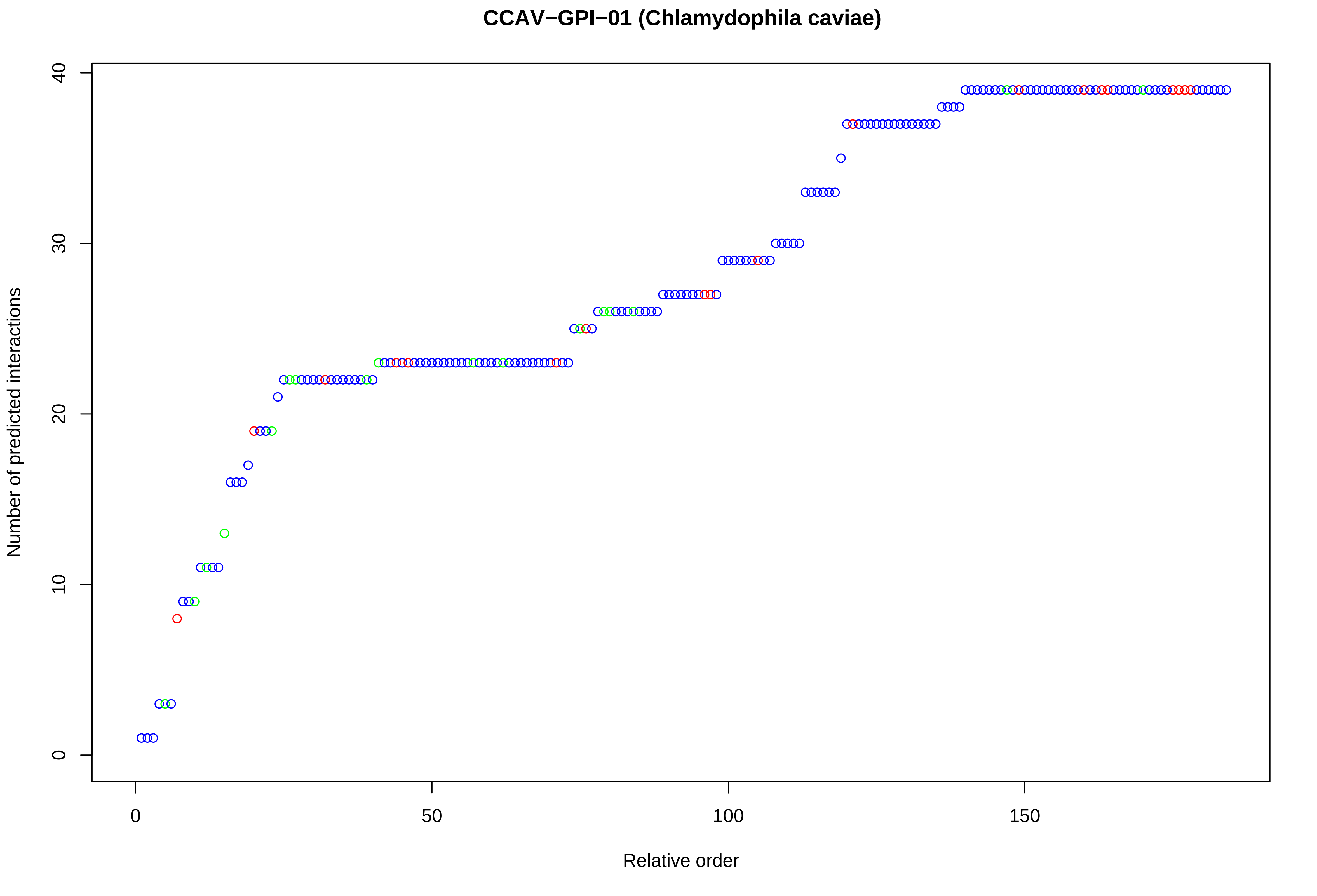
CCAV−GPI−01 (Chlamydophila caviae)
Number of predicted interactions
Relative order
0
10
20
30
40
0
50
100
150

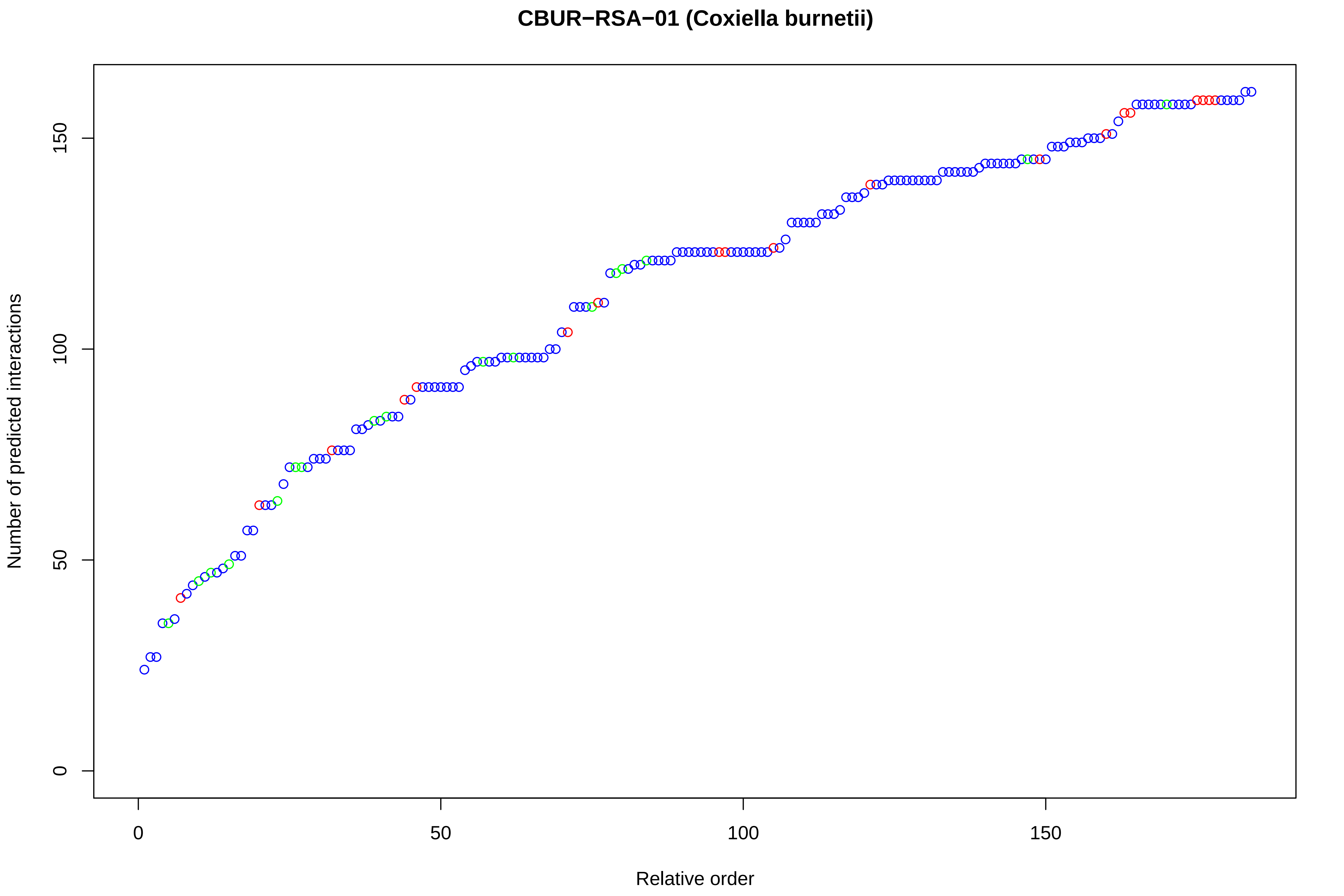
CBUR−RSA−01 (Coxiella burnetii)
Number of predicted interactions
0
50
100
150
Relative order
0
50
100
150

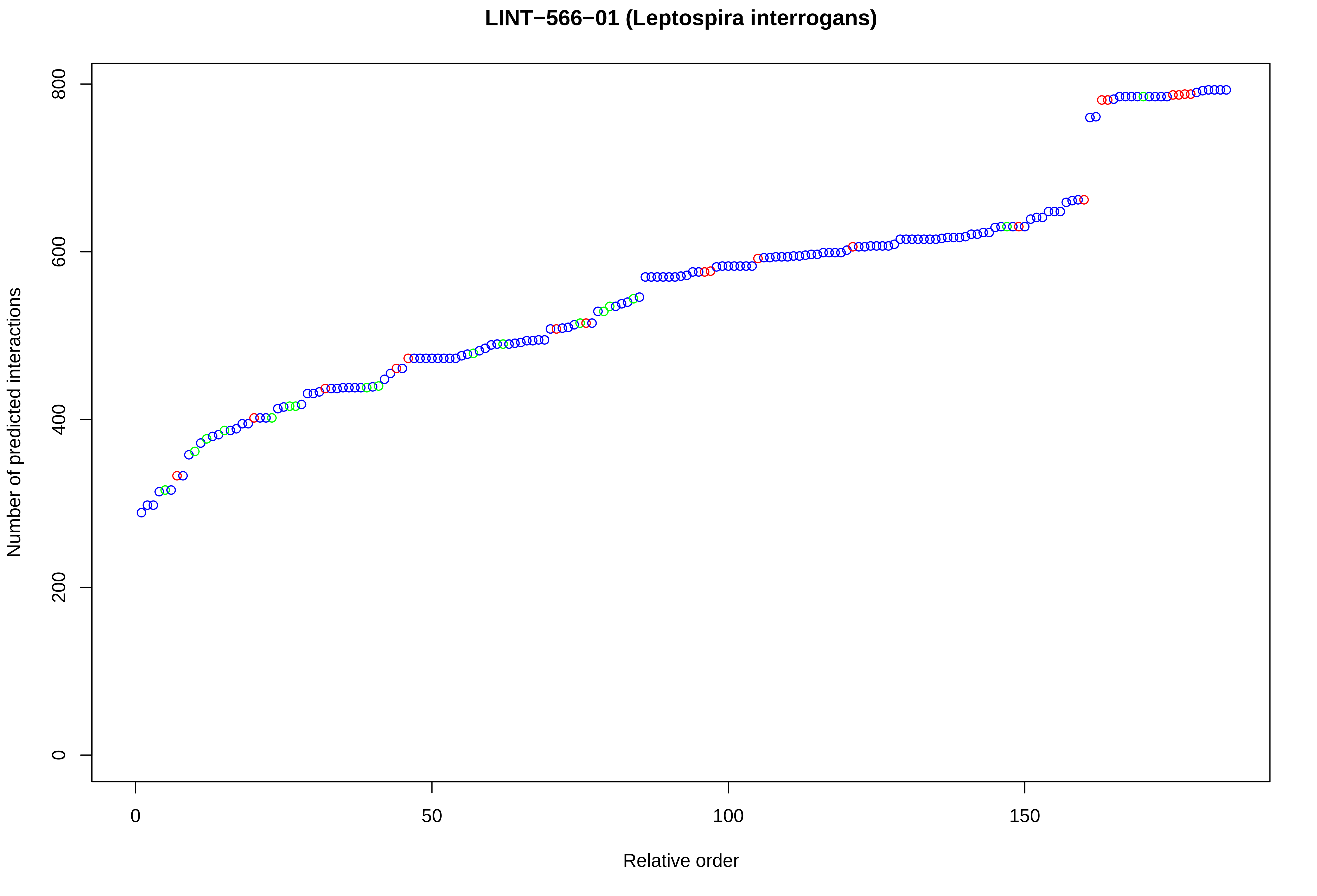

LINT−566−01 (Leptospira interrogans)
Number of predicted interactions
Relative order
0
200
400
600
800
0
50
100
150

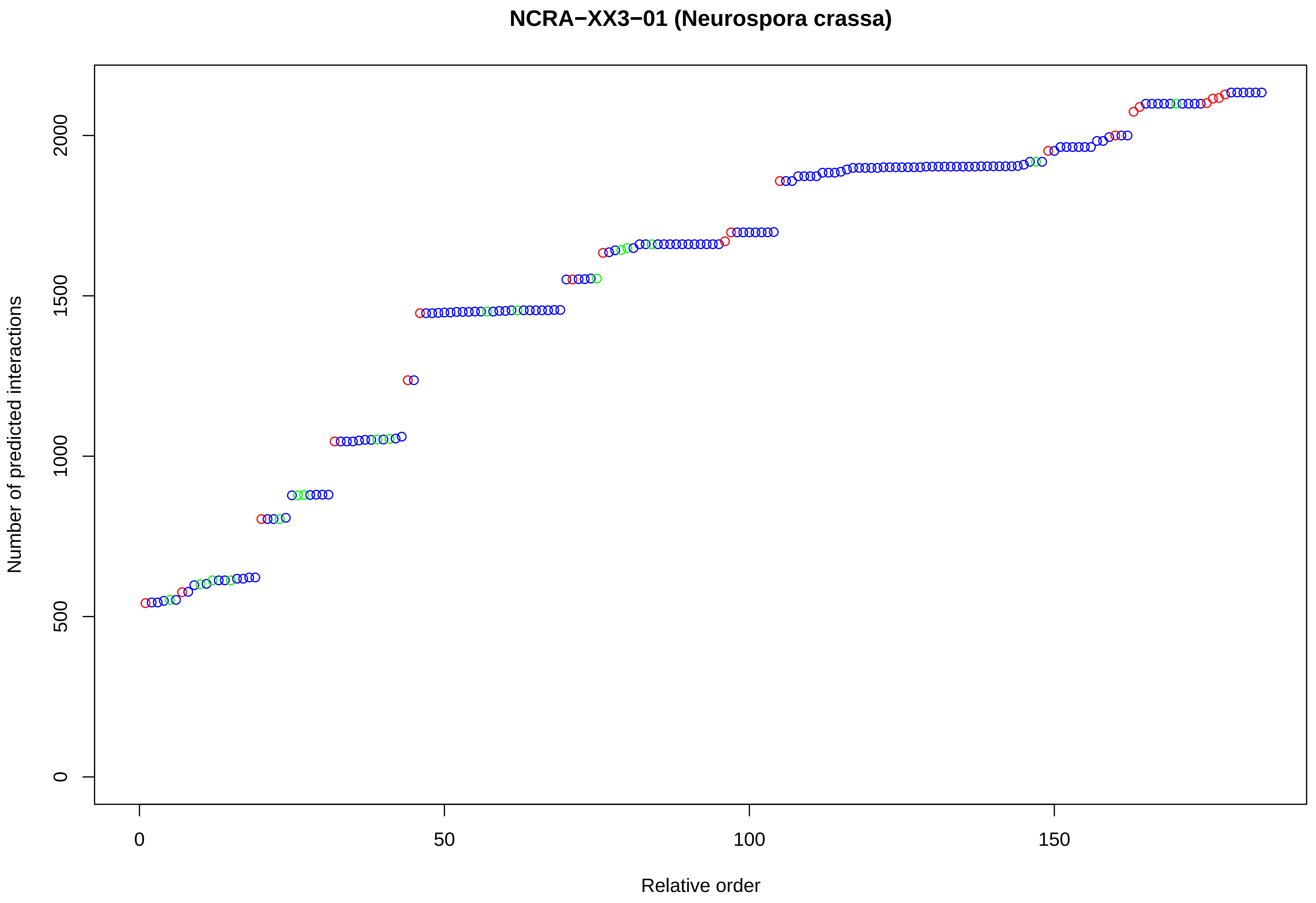

NCRA−XX3−01 (Neurospora crassa)
Number of predicted interactions
Relative order
0
500
1000
1500
2000
0
50
100
150

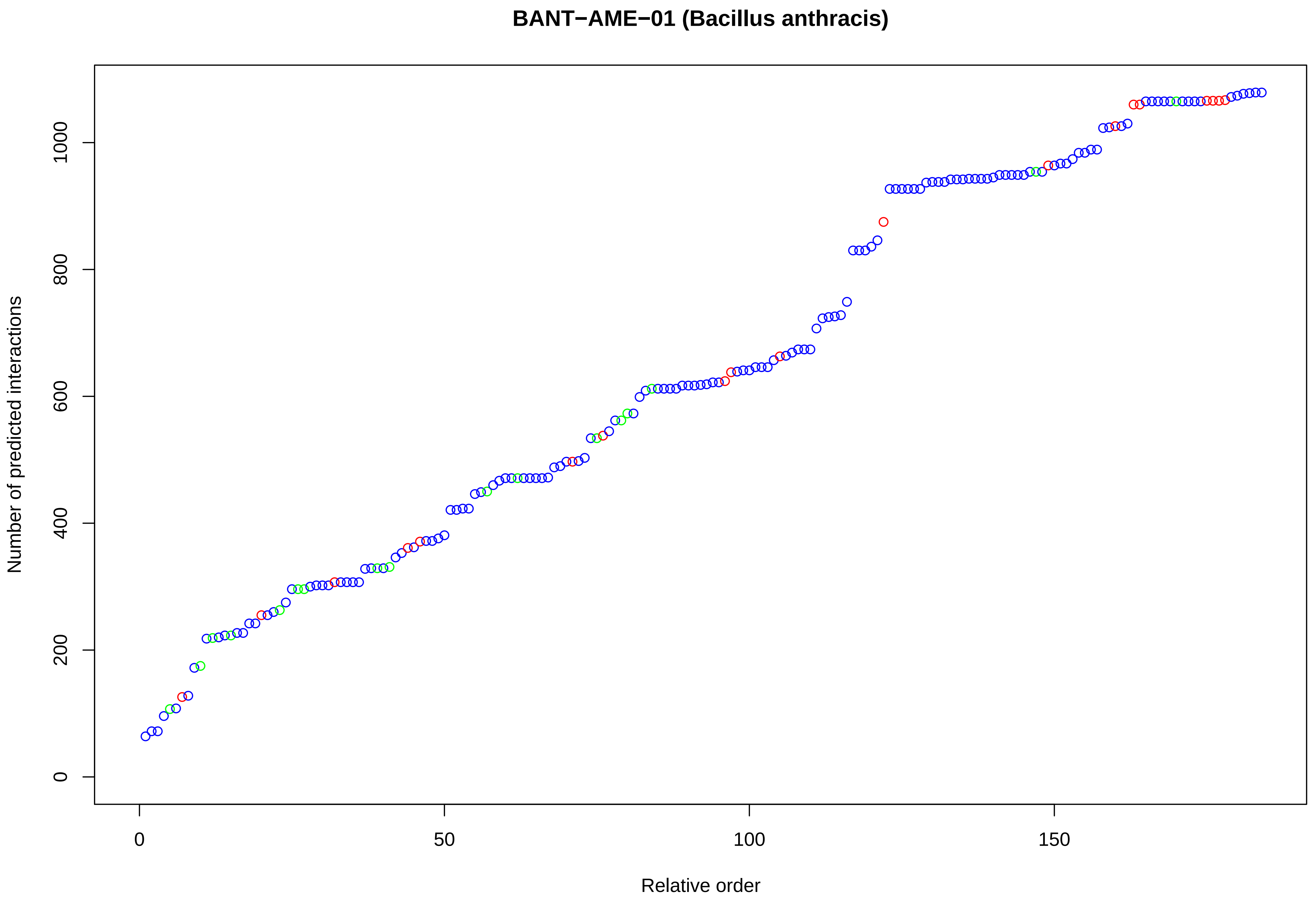
BANT−AME−01 (Bacillus anthracis)
Number of predicted interactions
0
200
400
600
800
1000
Relative order
0
50
100
150

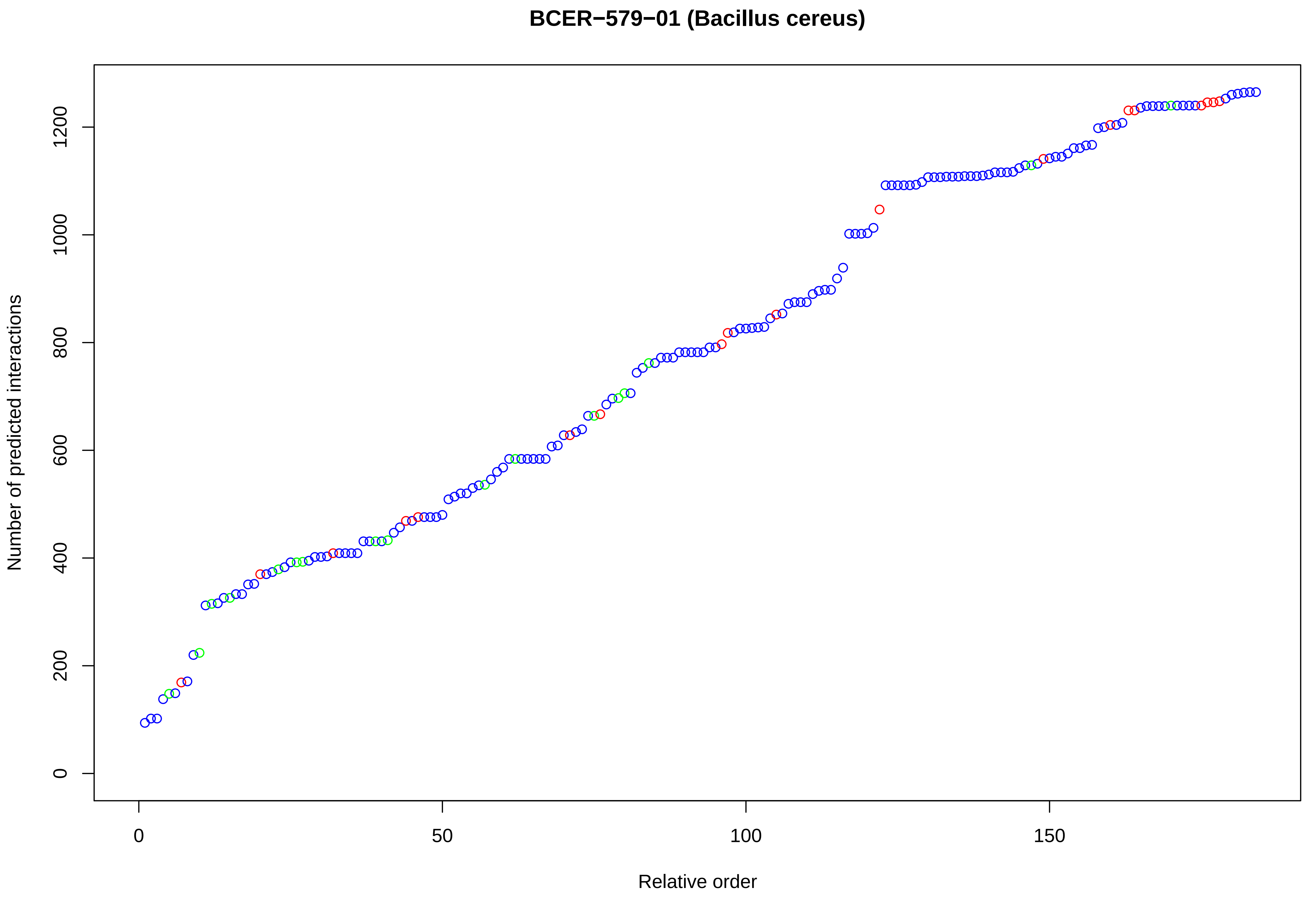
BCER−579−01 (Bacillus cereus)
Number of predicted interactions
Relative order
0
200
400
600
800
1000
1200
0
50
100
150

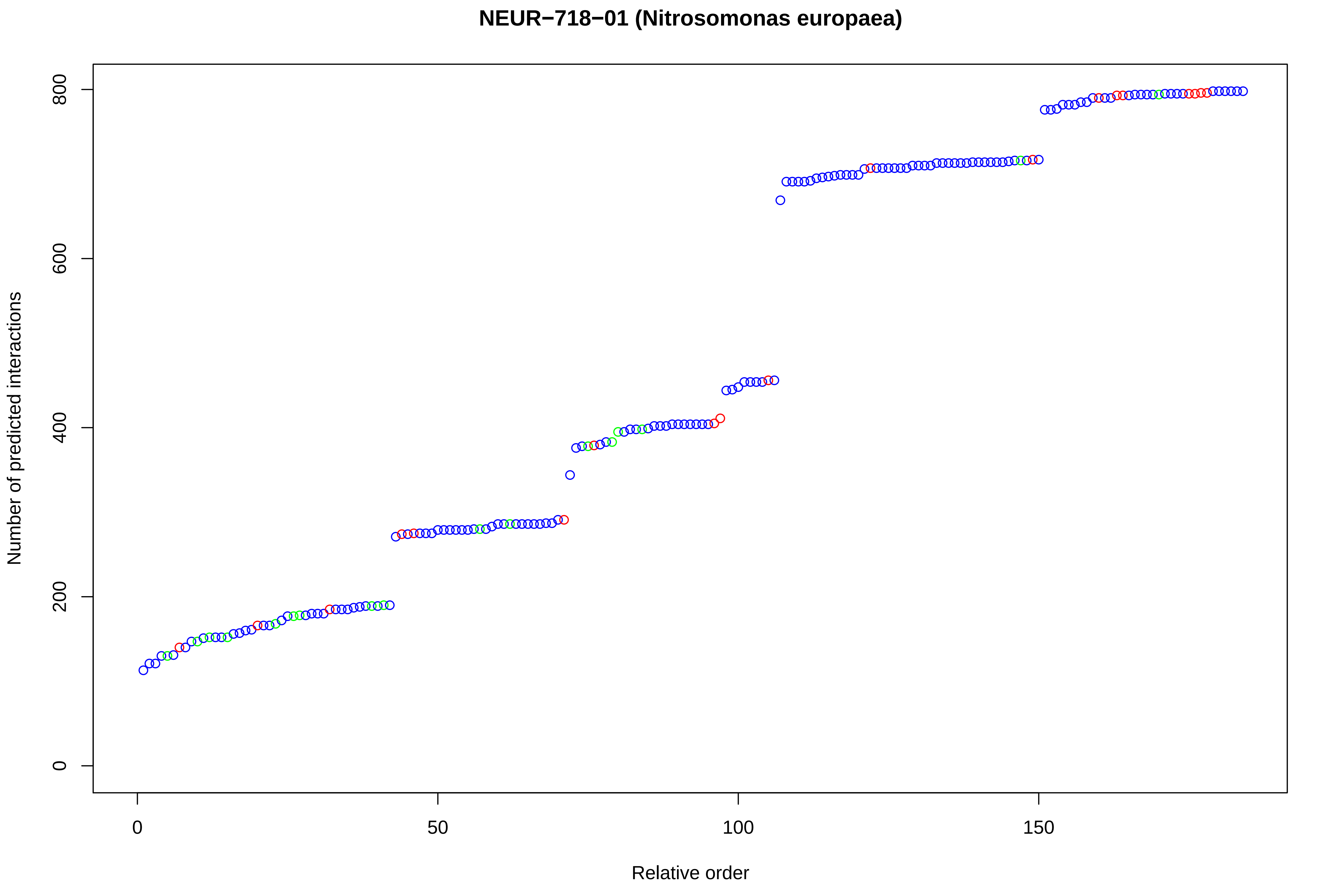

NEUR−718−01 (Nitrosomonas europaea)
Number of predicted interactions
Relative order
0
200
400
600
800
0
50
100
150

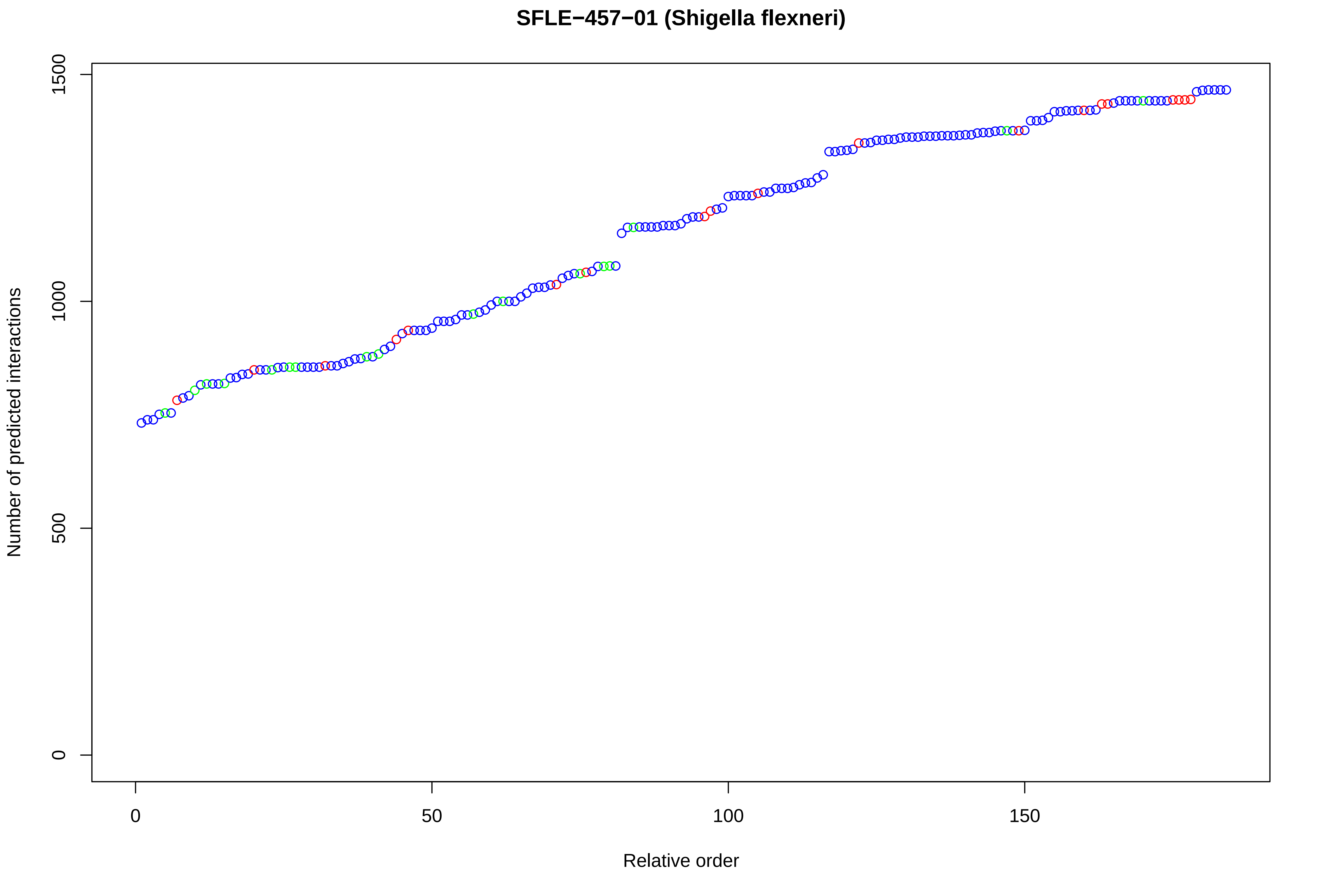

SFLE−457−01 (Shigella flexneri)
Number of predicted interactions
0
500
1000
1500
Relative order
0
50
100
150

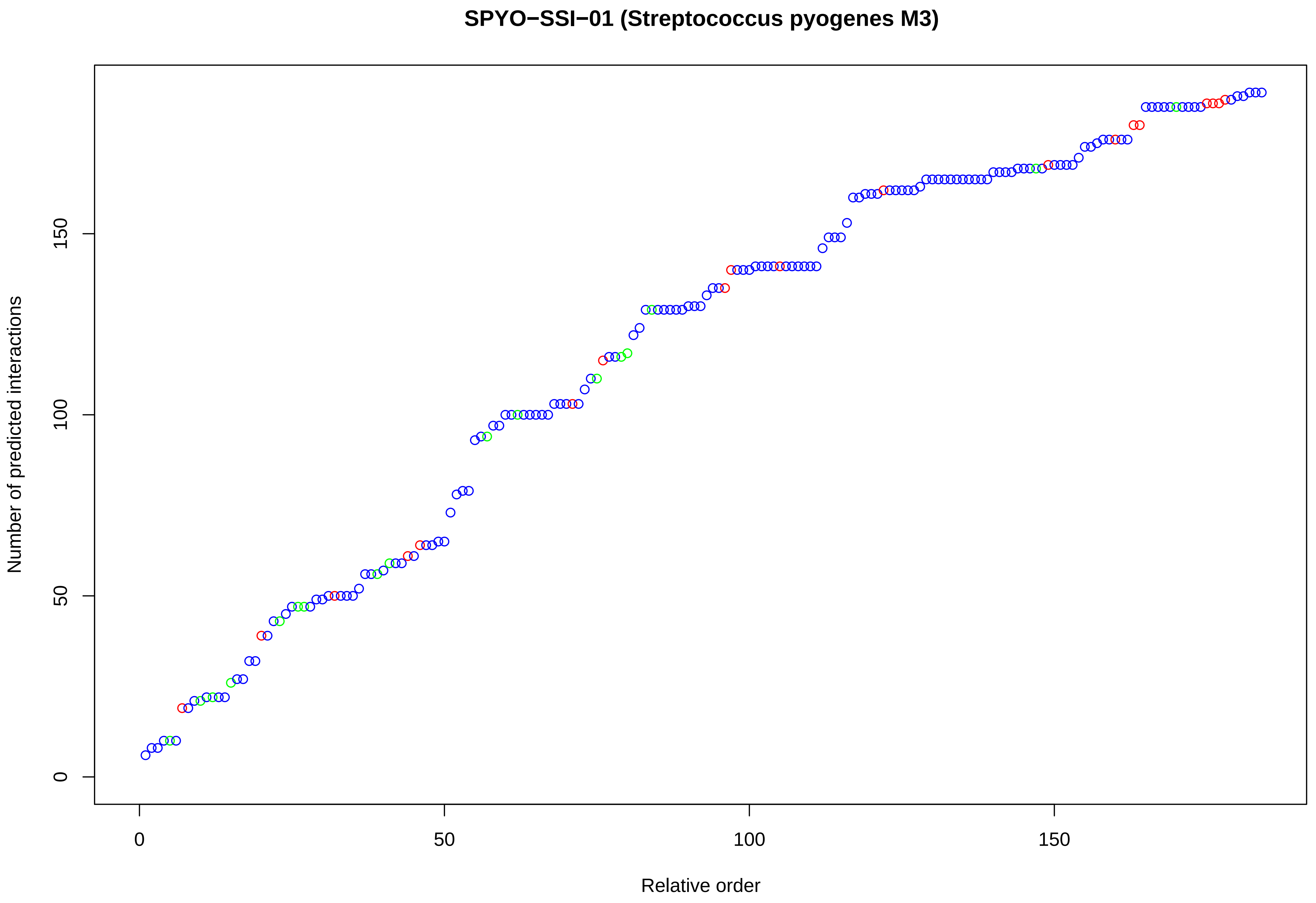

SPYO−SSI−01 (Streptococcus pyogenes M3)
Number of predicted interactions
Relative order
0
50
100
150
0
50
100
150

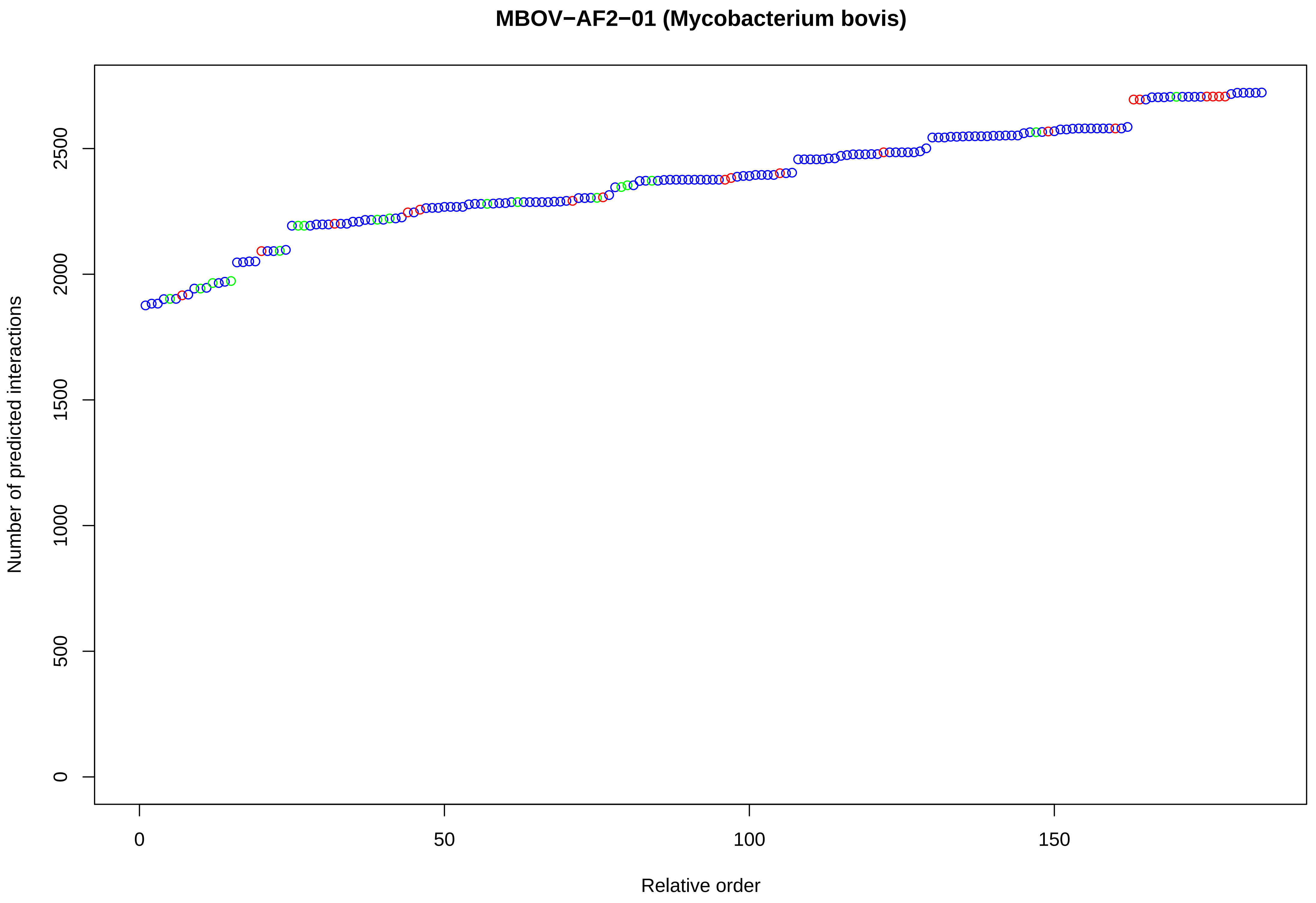

MBOV−AF2−01 (Mycobacterium bovis)
Number of predicted interactions
Relative order
0
500
1000
1500
2000
2500
0
50
100
150

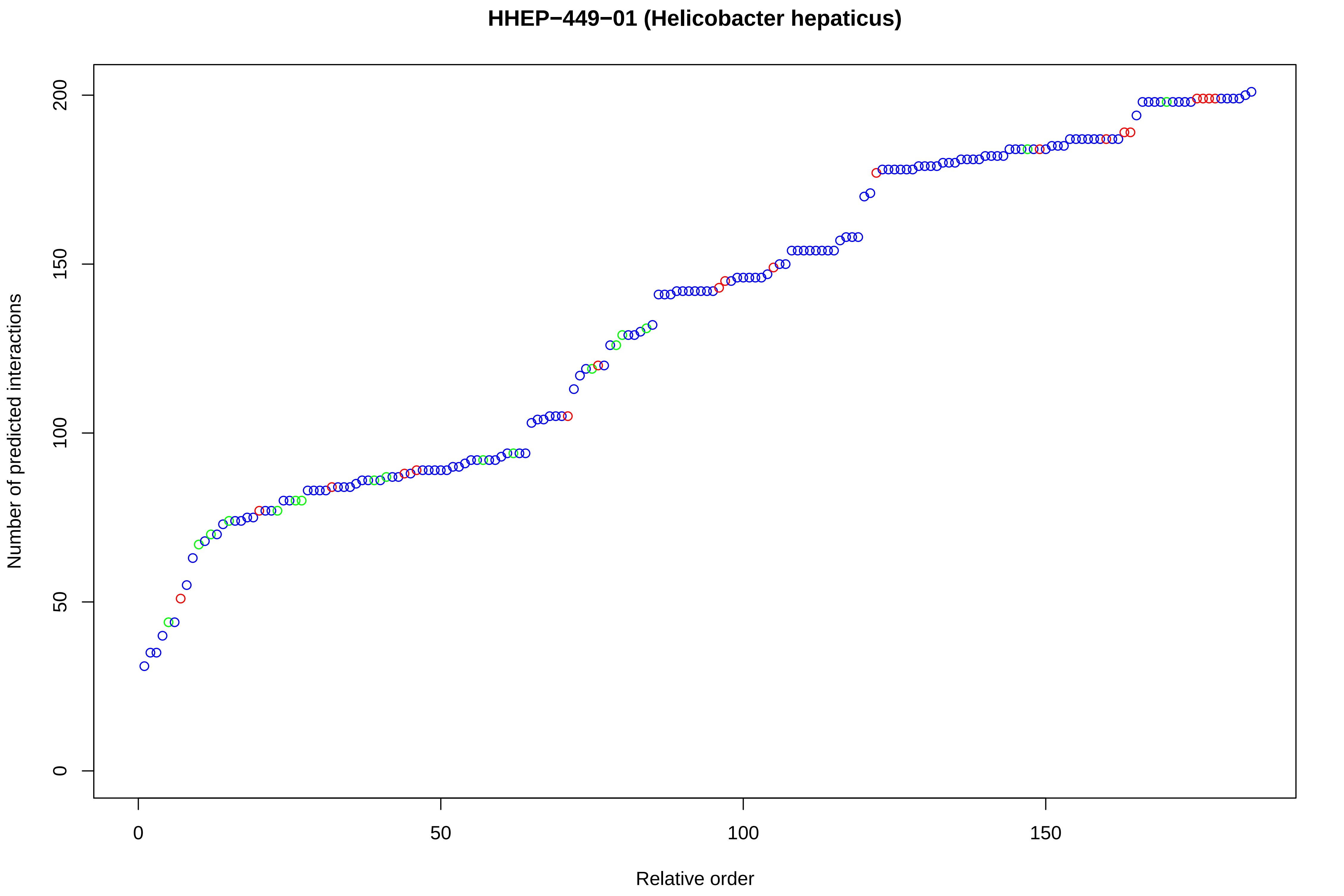
HHEP−449−01 (Helicobacter hepaticus)
Number of predicted interactions
Relative order
0
50
100
150
200
0
50
100
150

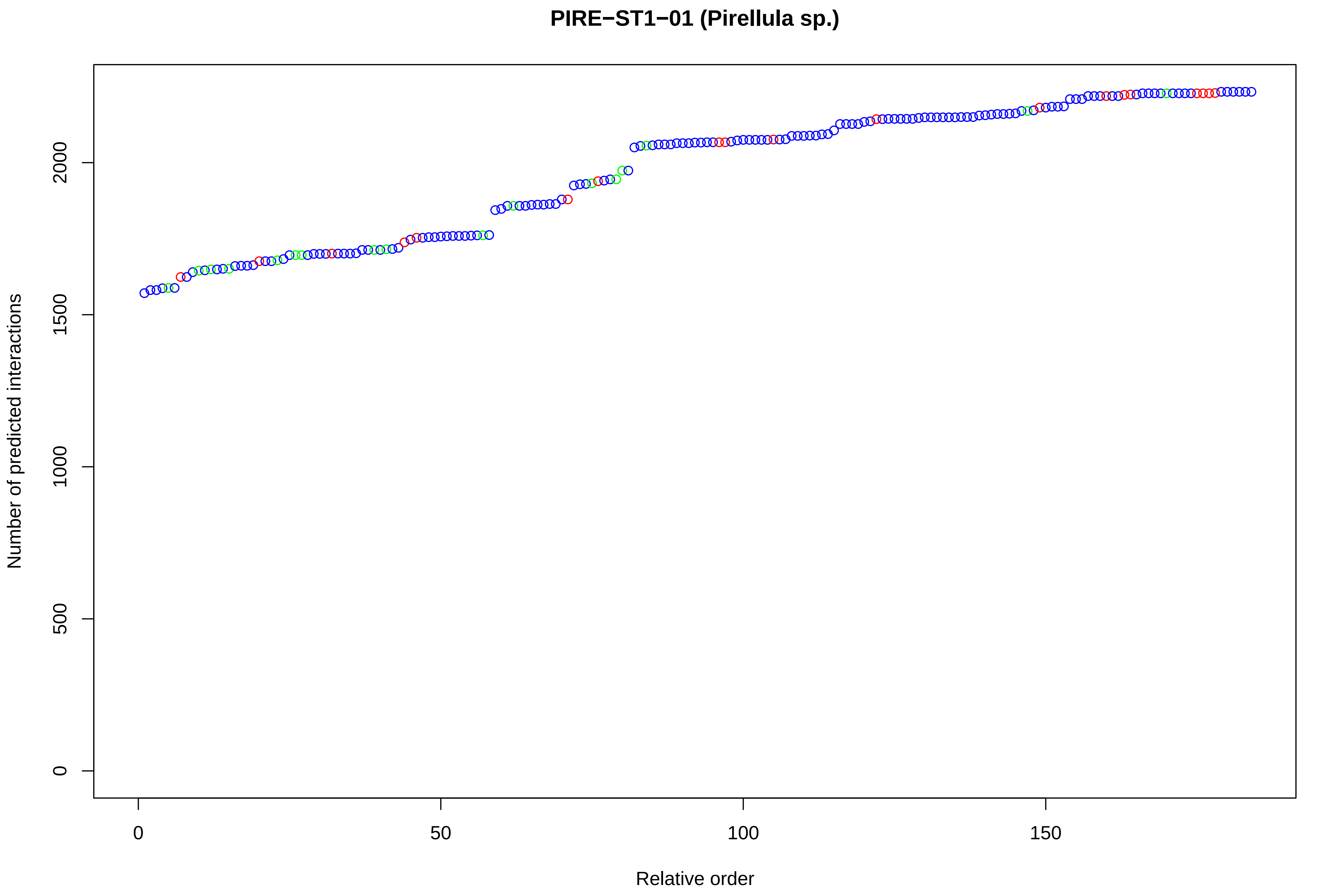

PIRE−ST1−01 (Pirellula sp.)
Number of predicted interactions
0
500
1000
1500
2000
0
50
100
150
Relative order

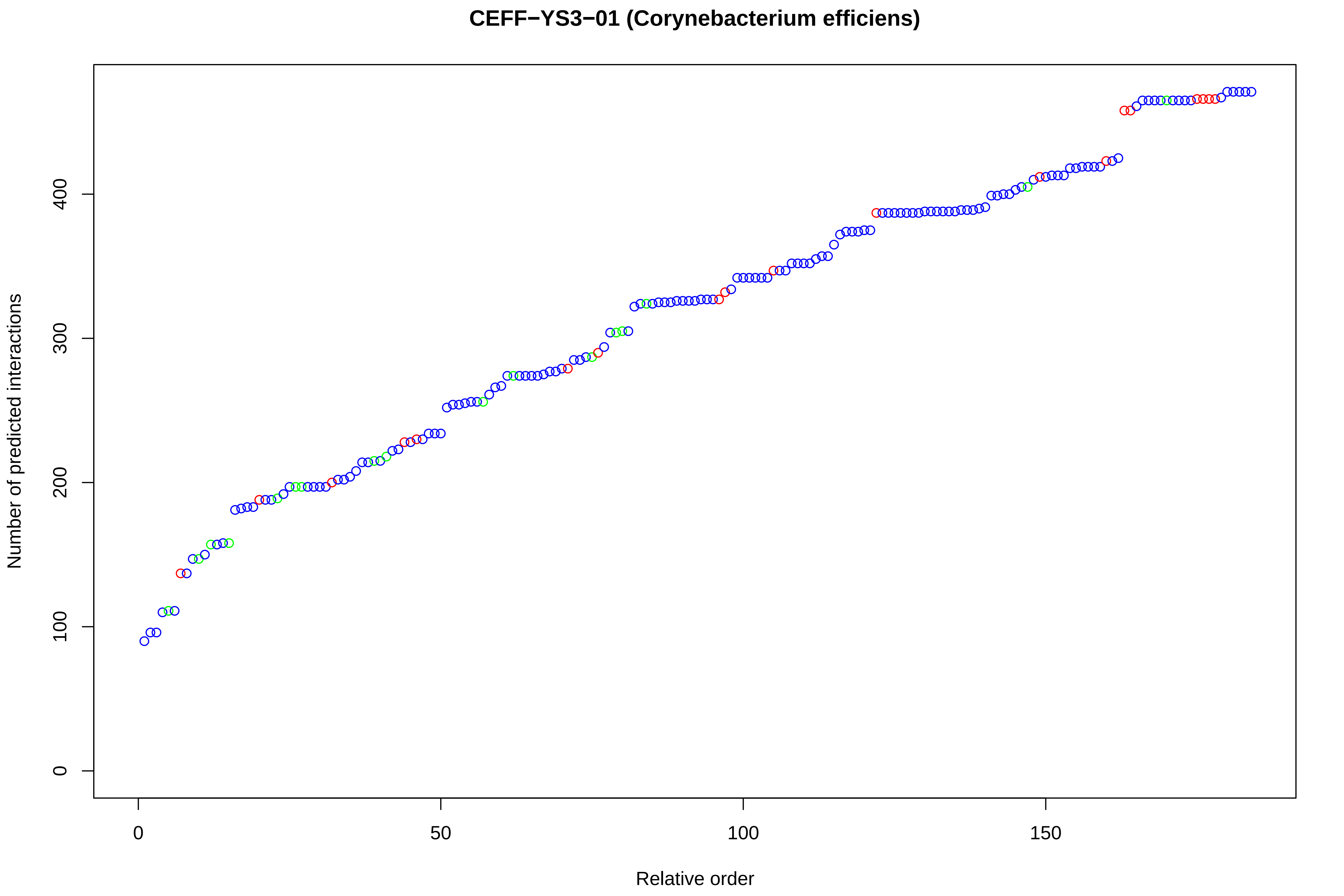
CEFF−YS3−01 (Corynebacterium efficiens)
Number of predicted interactions
0
100
200
300
400
0
50
100
150
Relative order

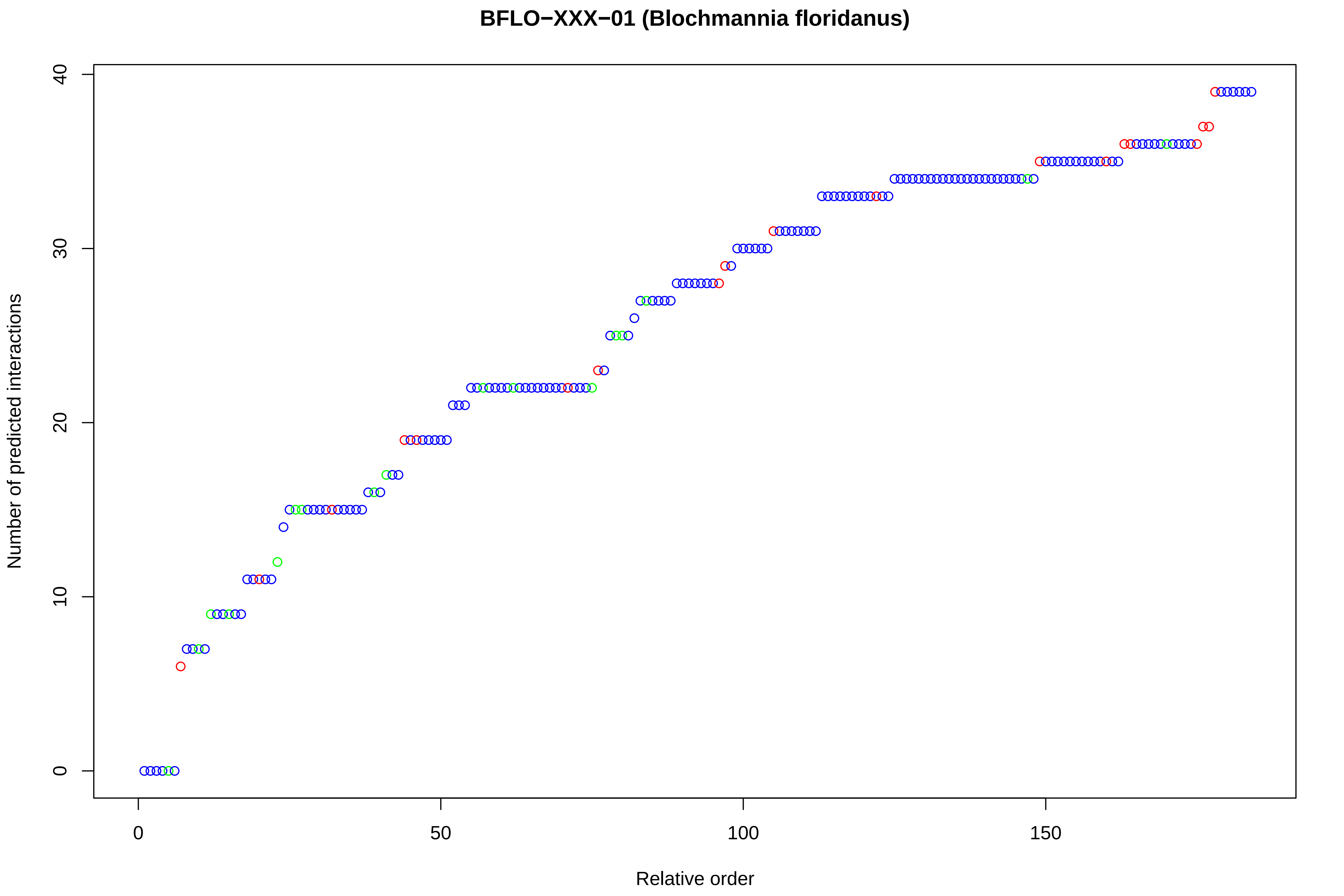
BFLO−XXX−01 (Blochmannia floridanus)
Number of predicted interactions
Relative order
0
10
20
30
40
0
50
100
150

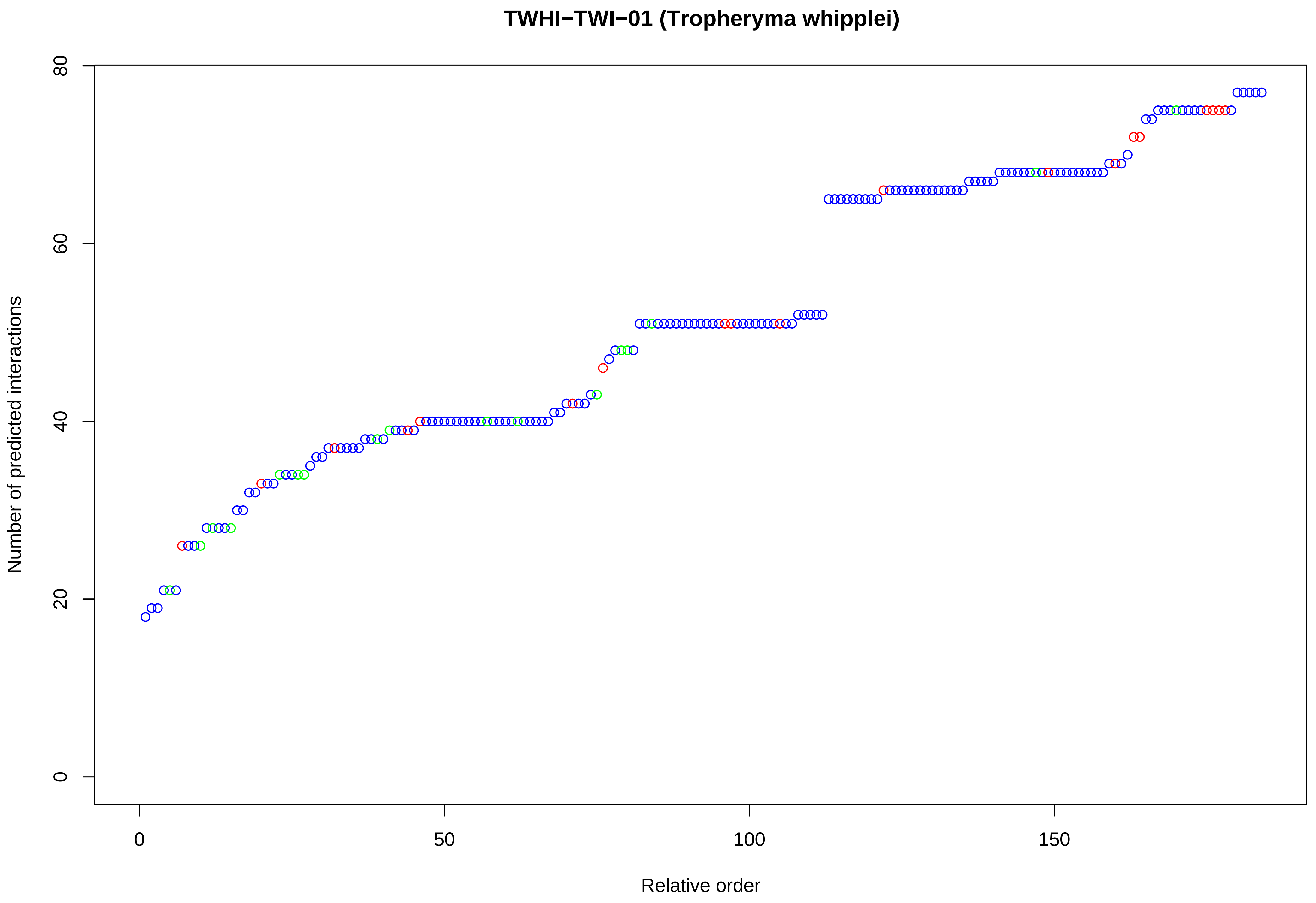

TWHI−TWI−01 (Tropheryma whipplei)
Number of predicted interactions
Relative order
0
20
40
60
80
0
50
100
150

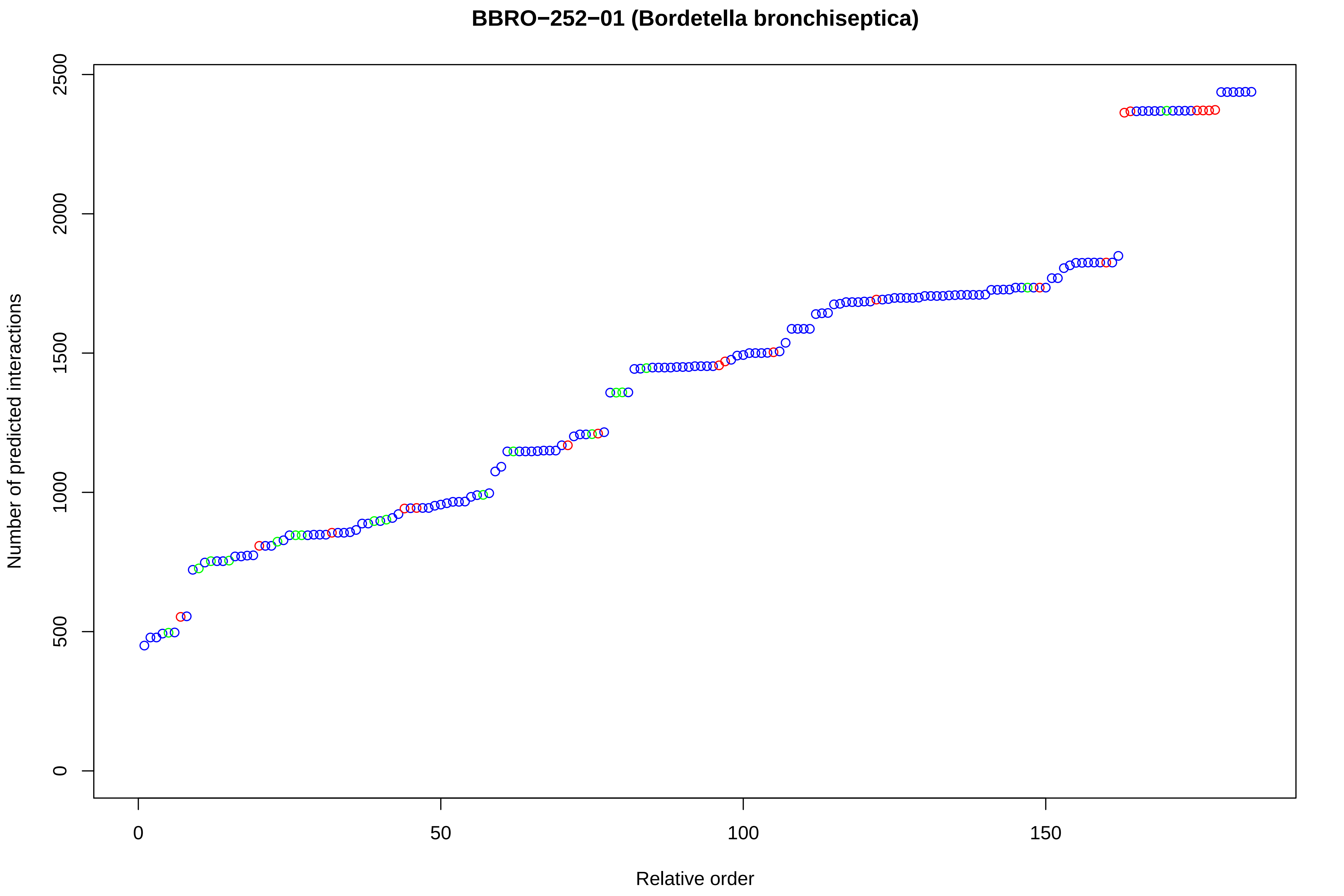
BBRO−252−01 (Bordetella bronchiseptica)
Number of predicted interactions
0
500
1000
1500
2000
2500
0
50
100
150
Relative order

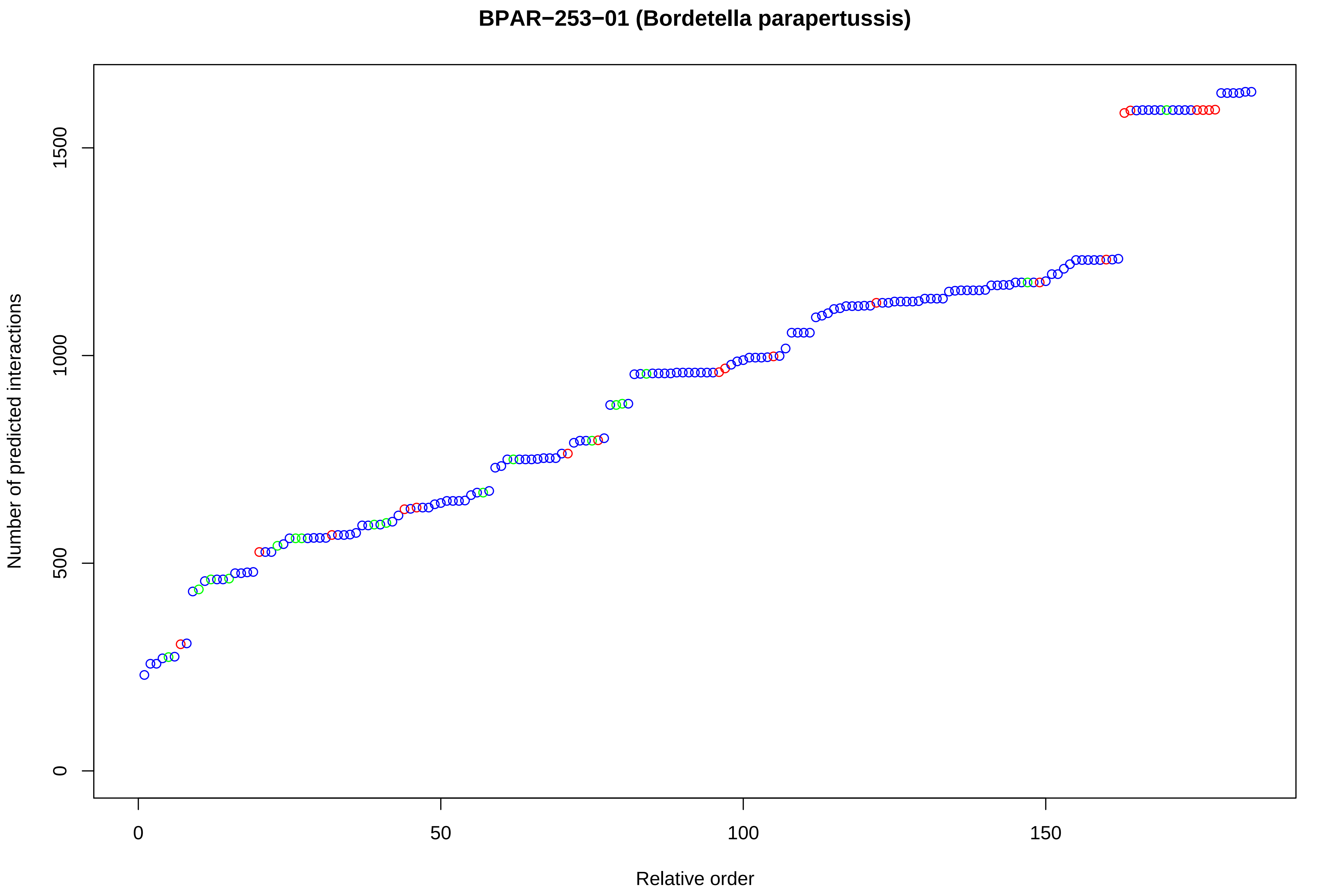
BPAR−253−01 (Bordetella parapertussis)
Number of predicted interactions
0
500
1000
1500
Relative order
0
50
100
150

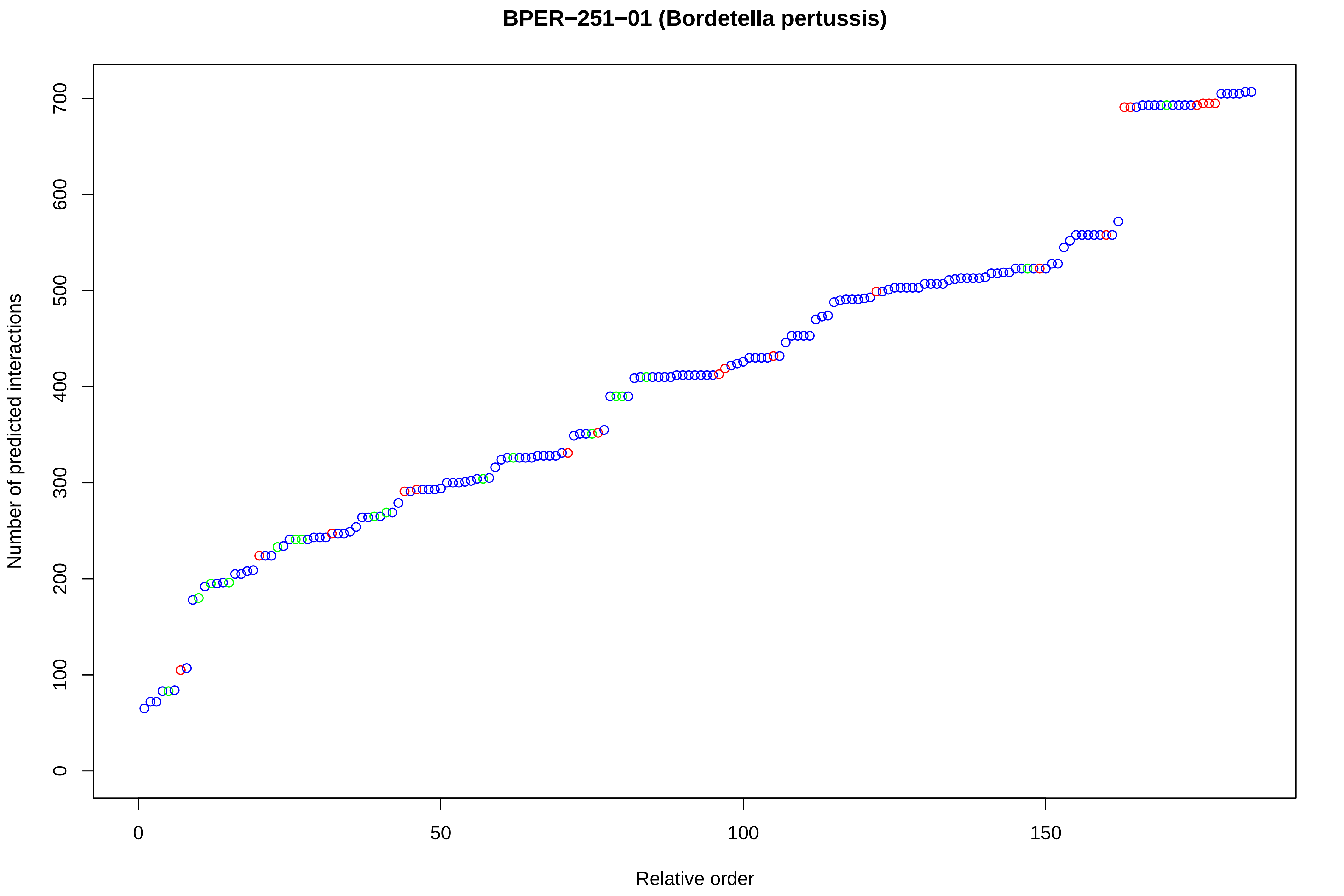
BPER−251−01 (Bordetella pertussis)
Number of predicted interactions
Relative order
0
100
200
300
400
500
600
700
0
50
100
150

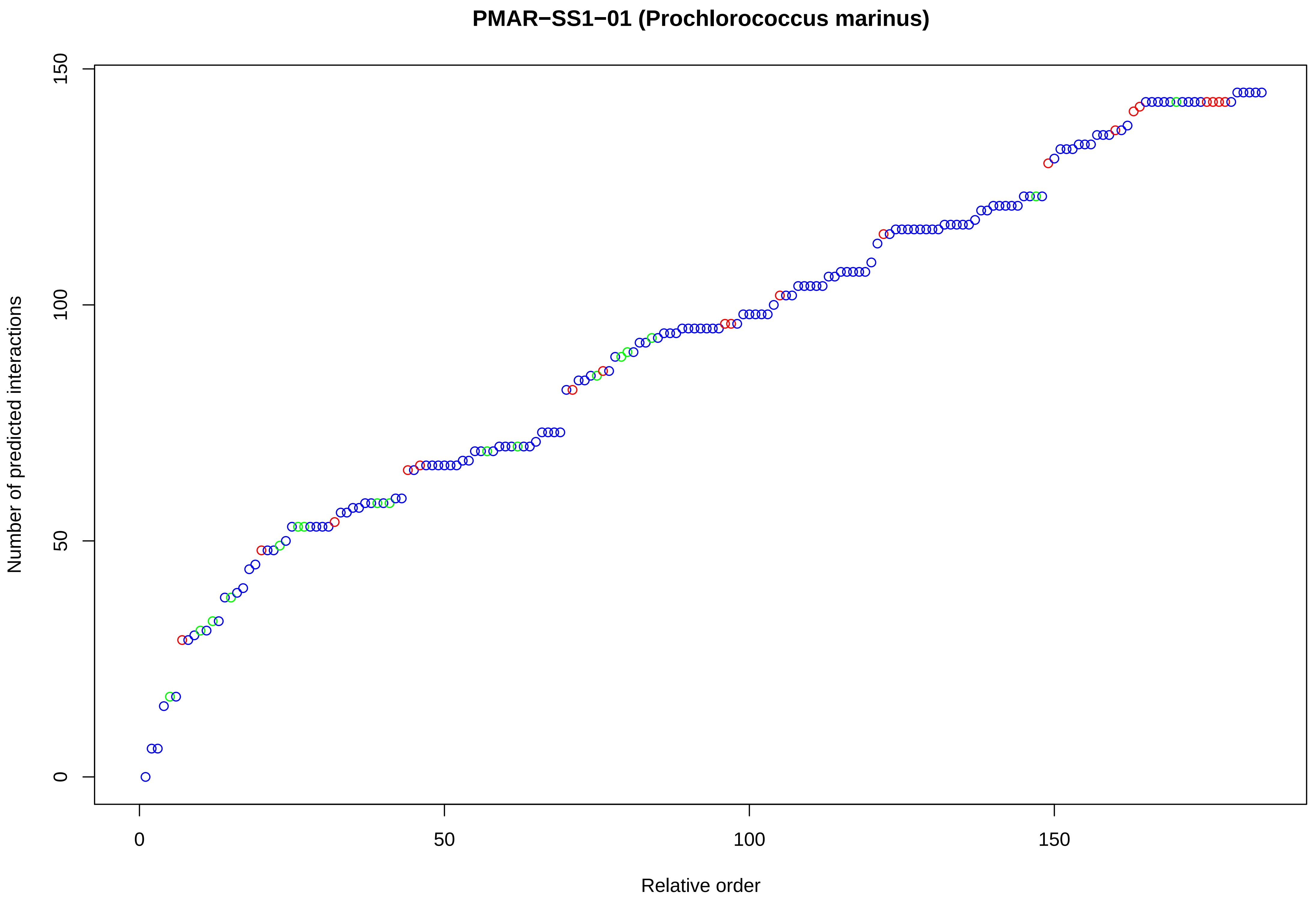

PMAR−SS1−01 (Prochlorococcus marinus)
Number of predicted interactions
Relative order
0
50
100
150
0
50
100
150

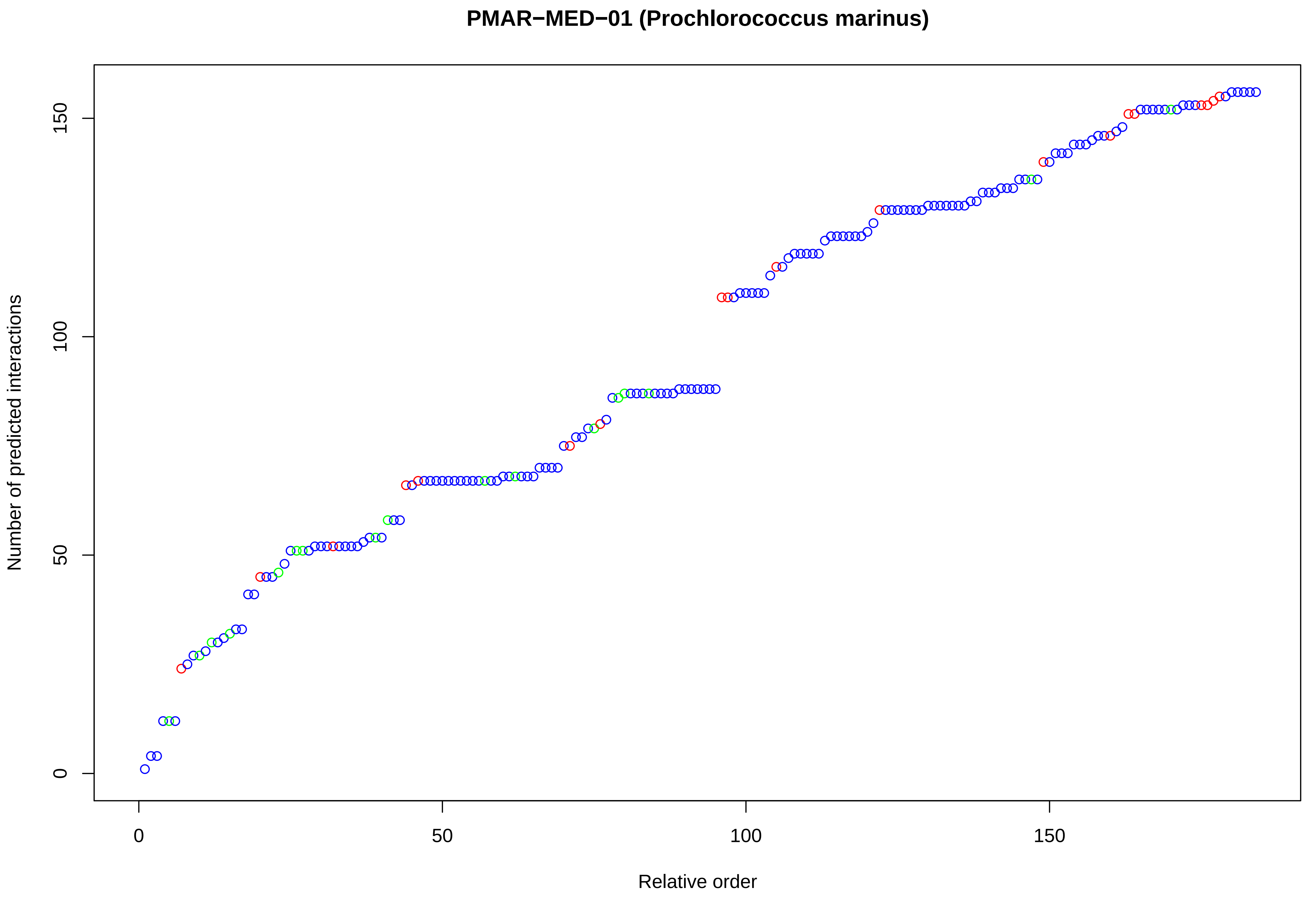
PMAR−MED−01 (Prochlorococcus marinus)
Number of predicted interactions
Relative order
0
50
100
150
0
50
100
150

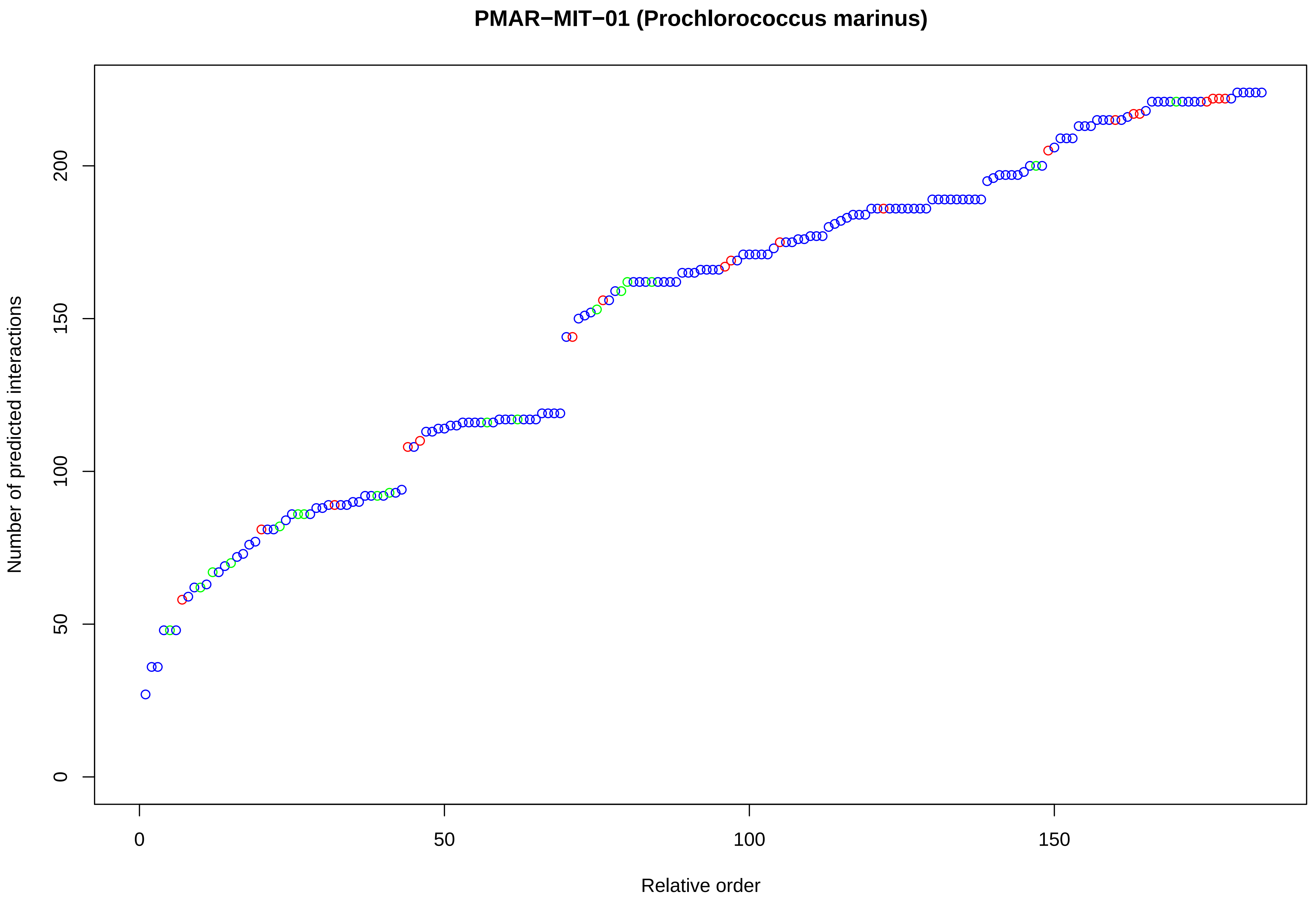

PMAR−MIT−01 (Prochlorococcus marinus)
Number of predicted interactions
Relative order
0
50
100
150
200
0
50
100
150

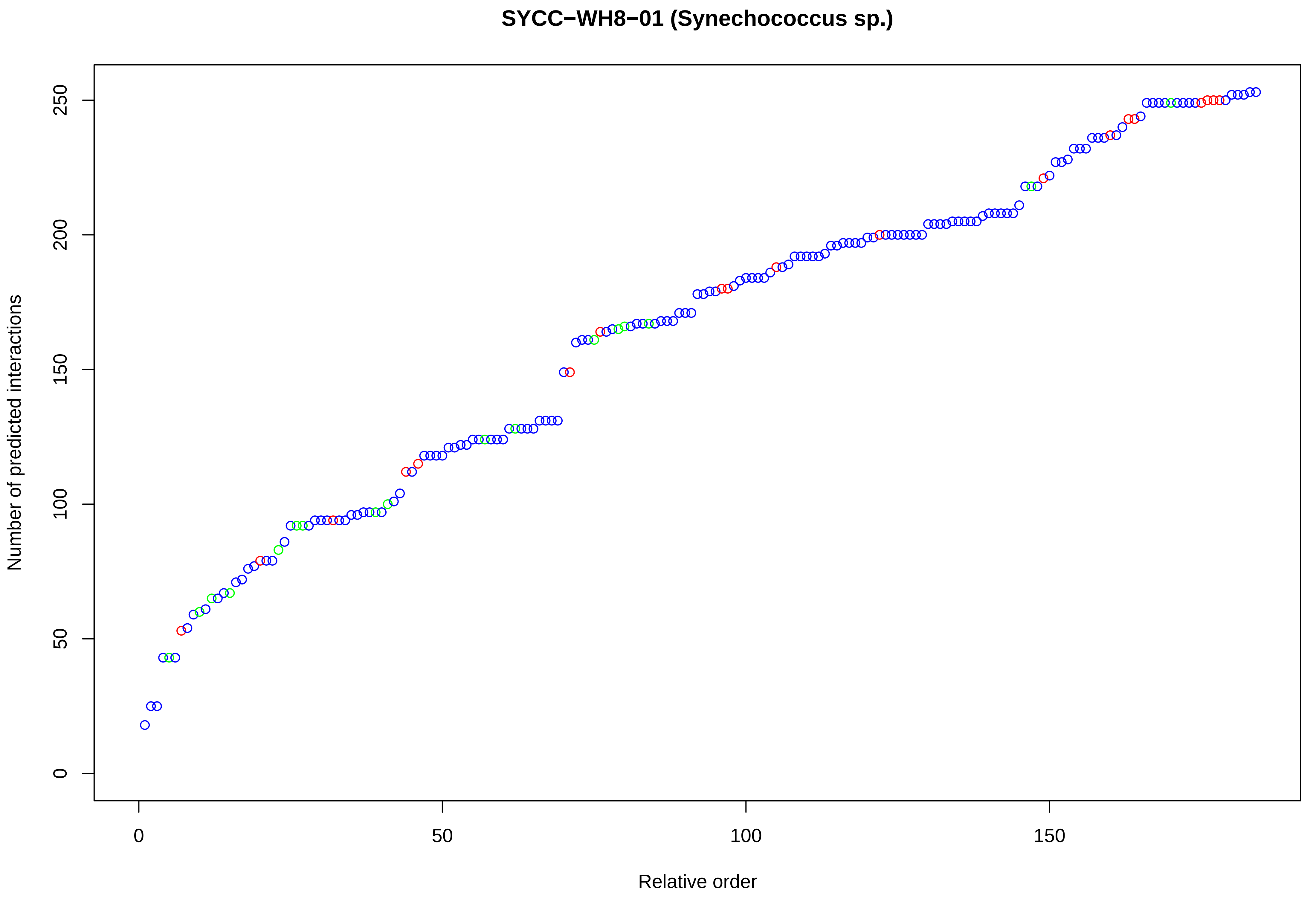
SYCC−WH8−01 (Synechococcus sp.)
Number of predicted interactions
Relative order
0
50
100
150
200
250

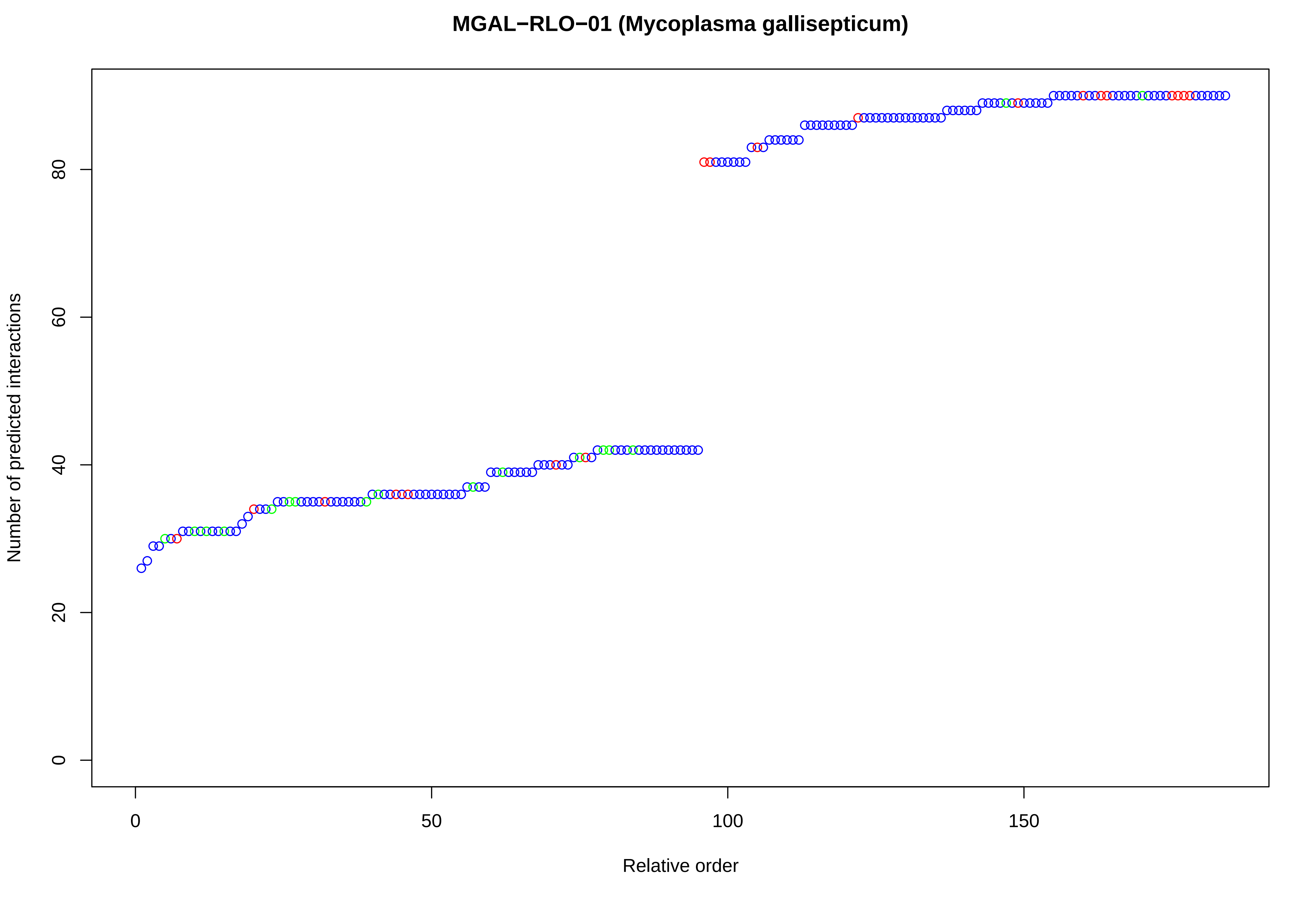
MGAL−RLO−01 (Mycoplasma gallisepticum)
Number of predicted interactions
Relative order
0
20
40
60
80
0
50
100
150

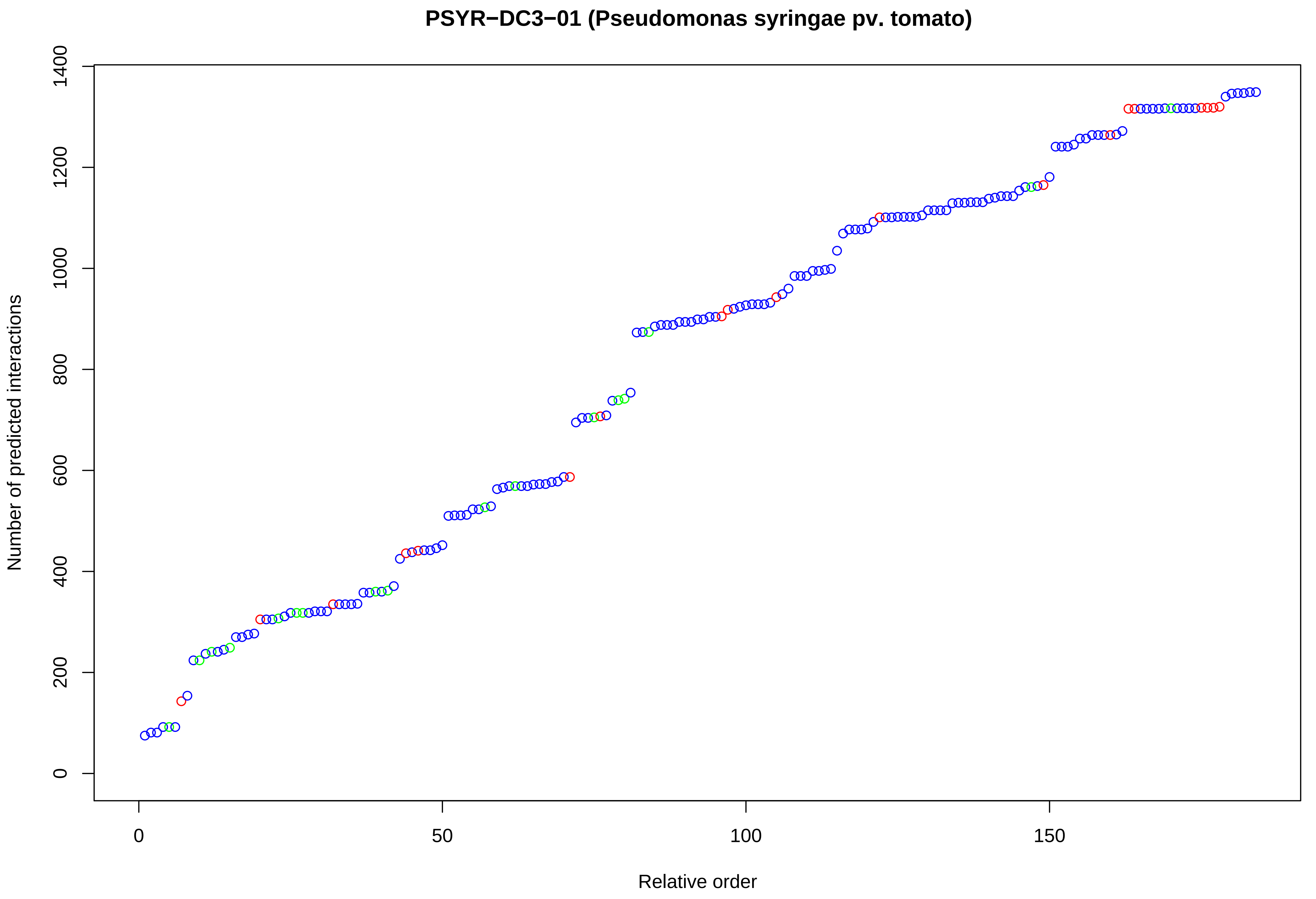
PSYR−DC3−01 (Pseudomonas syringae pv. tomato)
Number of predicted interactions
0
200
400
600
800
1000
1200
1400
0
50
100
150
Relative order

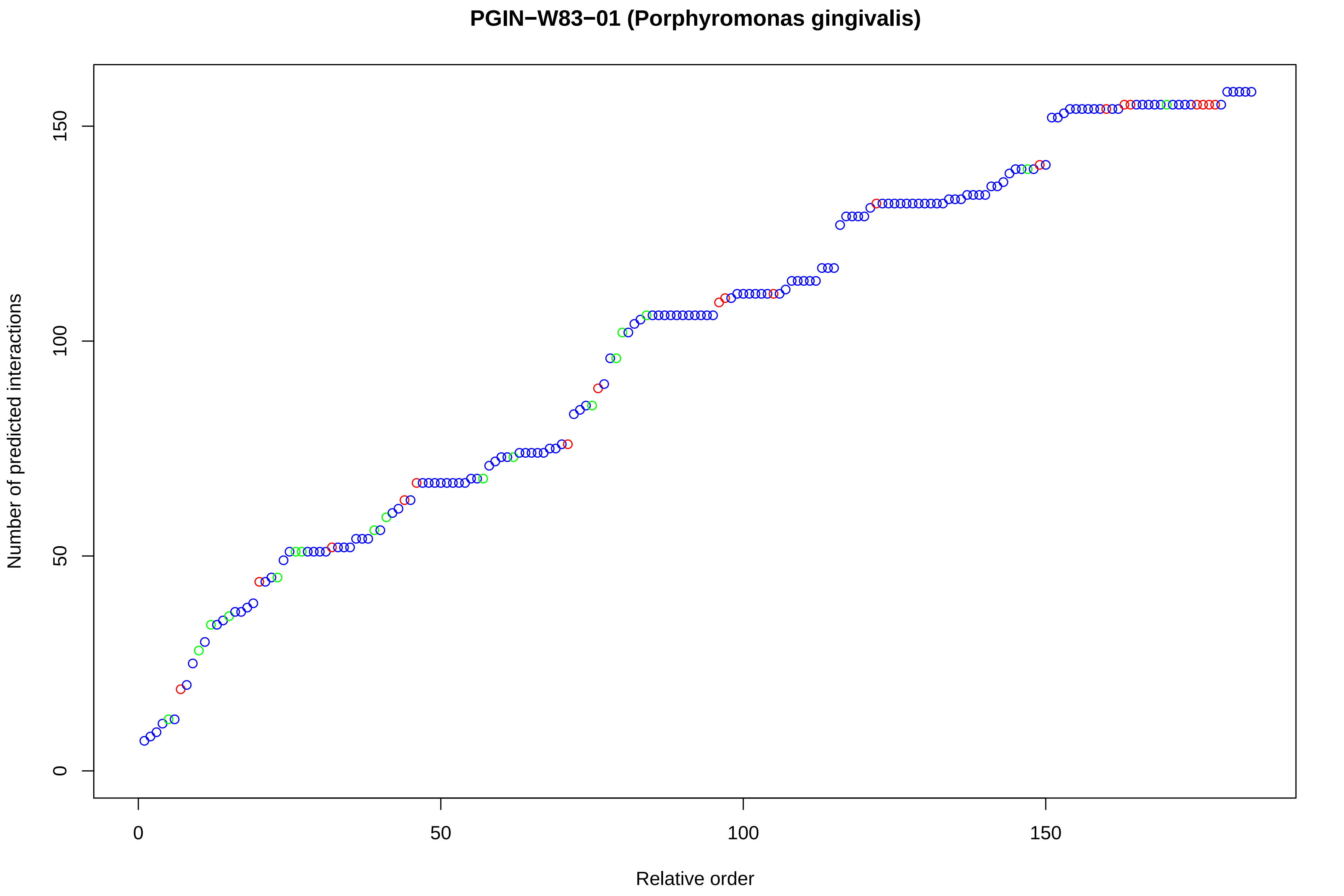
PGIN−W83−01 (Porphyromonas gingivalis)
Number of predicted interactions
0
50
100
150
Relative order
0
50
100
150

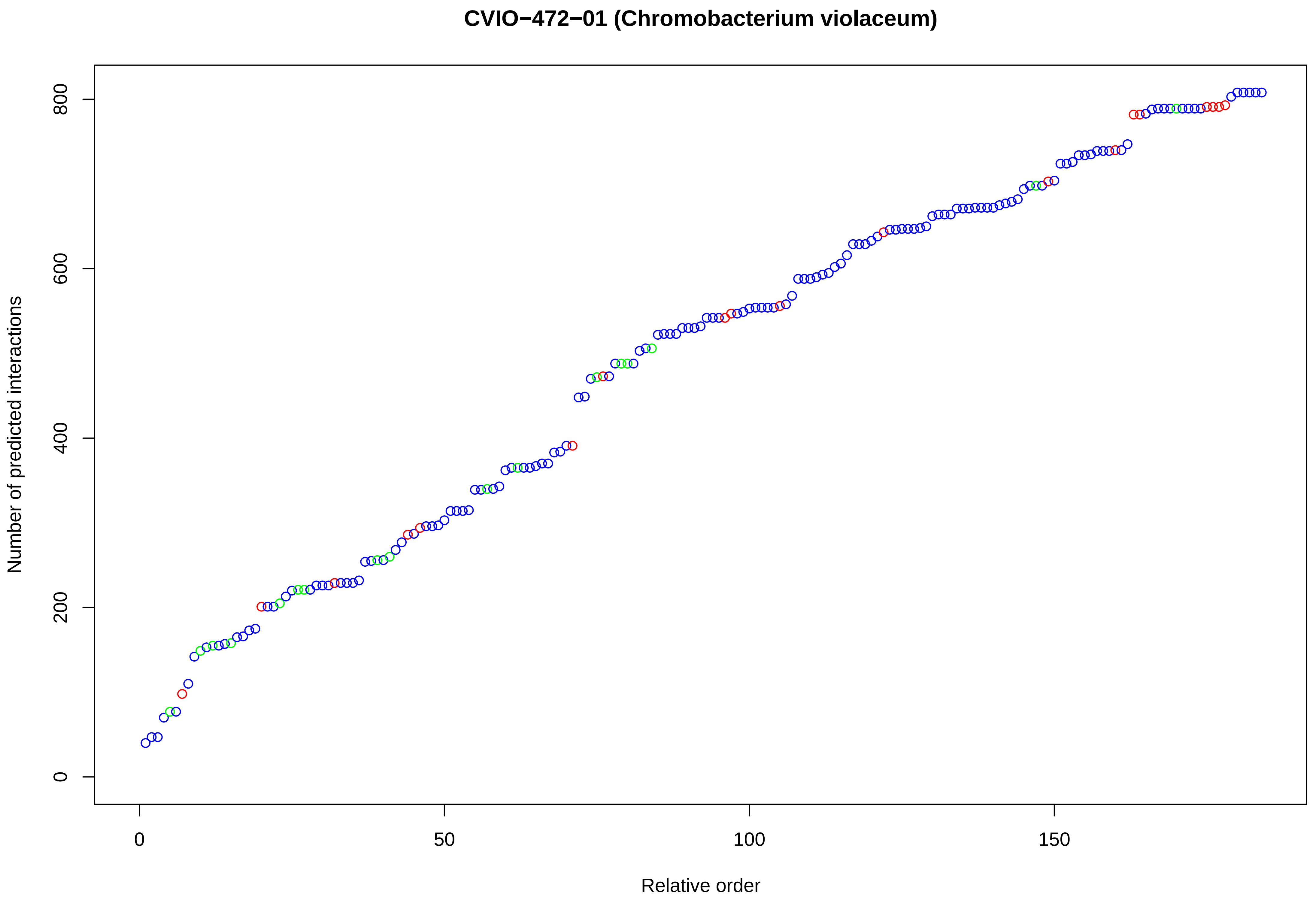
CVIO−472−01 (Chromobacterium violaceum)
Number of predicted interactions
Relative order
0
200
400
600
800
0
50
100
150

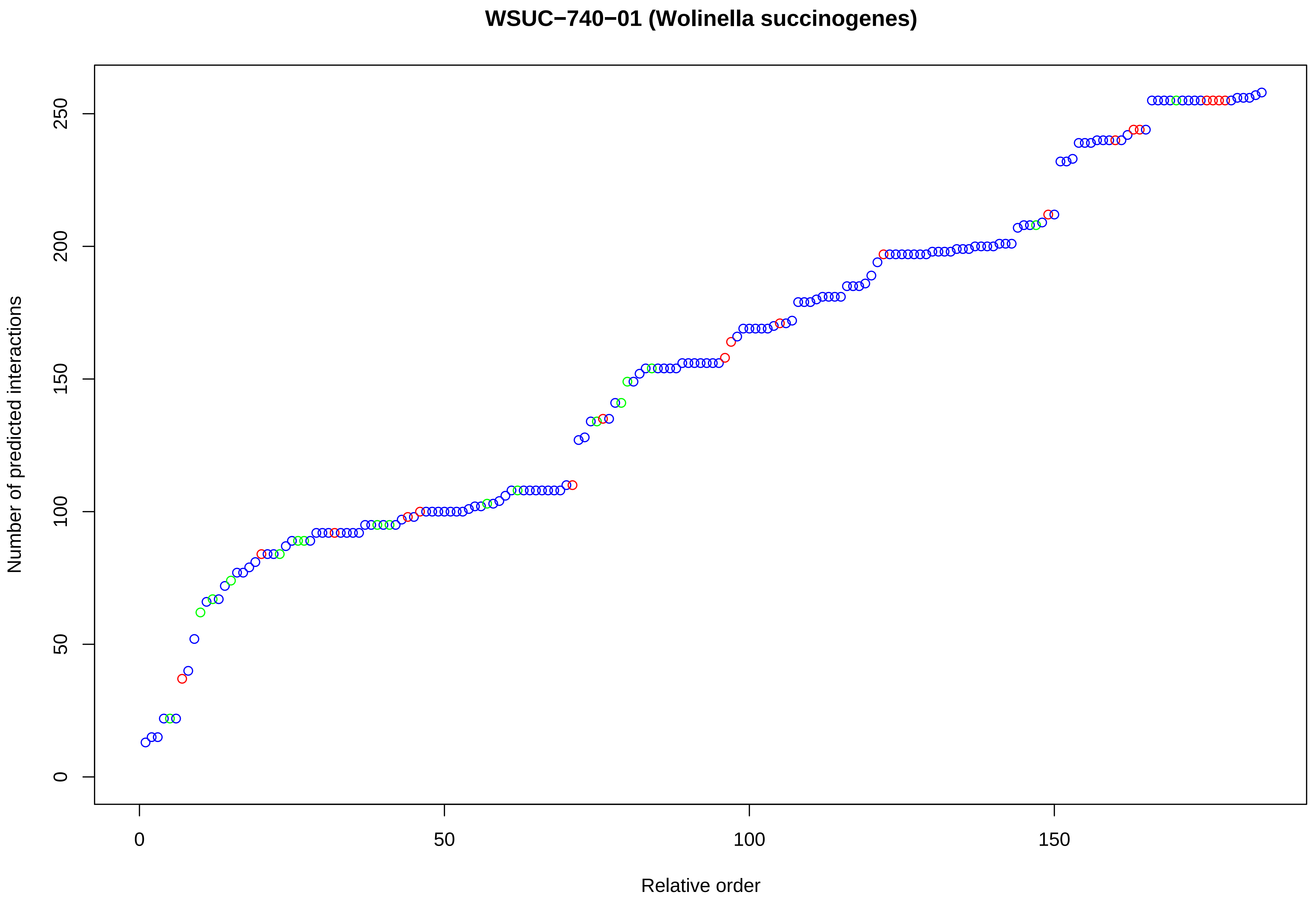

WSUC−740−01 (Wolinella succinogenes)
Number of predicted interactions
0
50
100
150
200
250
Relative order
0
50
100
150

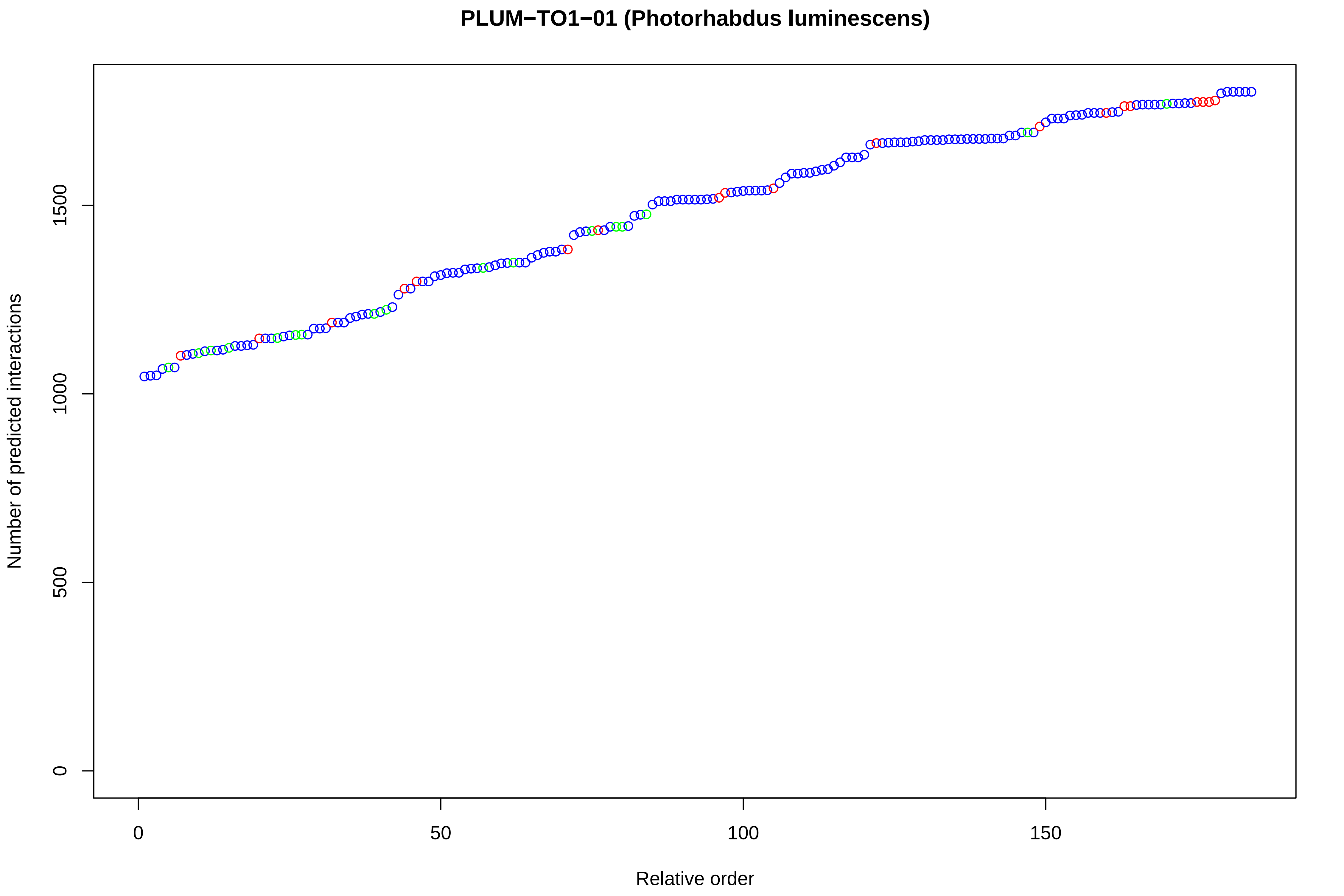

PLUM−TO1−01 (Photorhabdus luminescens)
Number of predicted interactions
0
500
1000
1500
Relative order
0
50
100
150

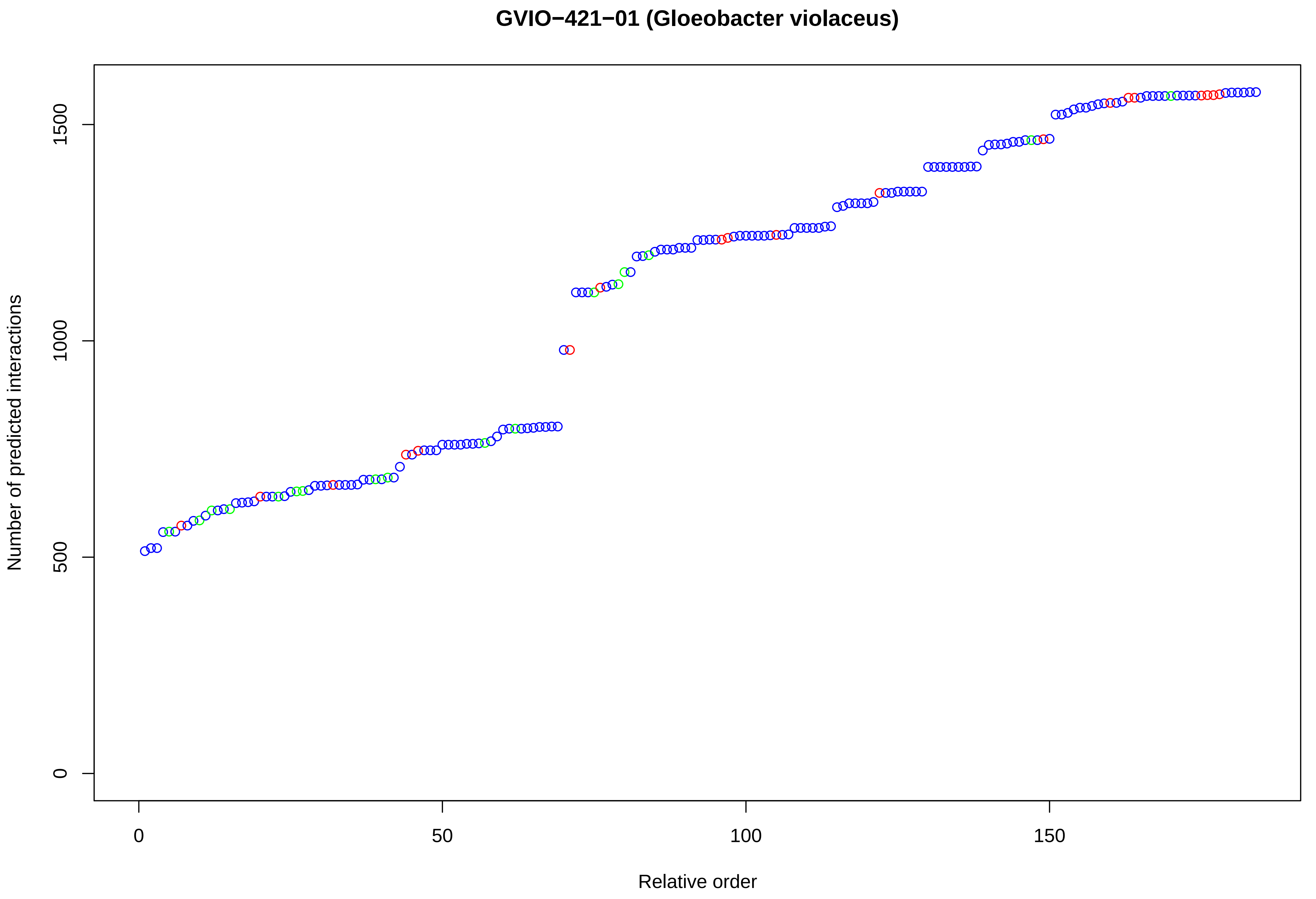
GVIO−421−01 (Gloeobacter violaceus)
Number of predicted interactions
0
500
1000
1500
0
50
100
150
Relative order

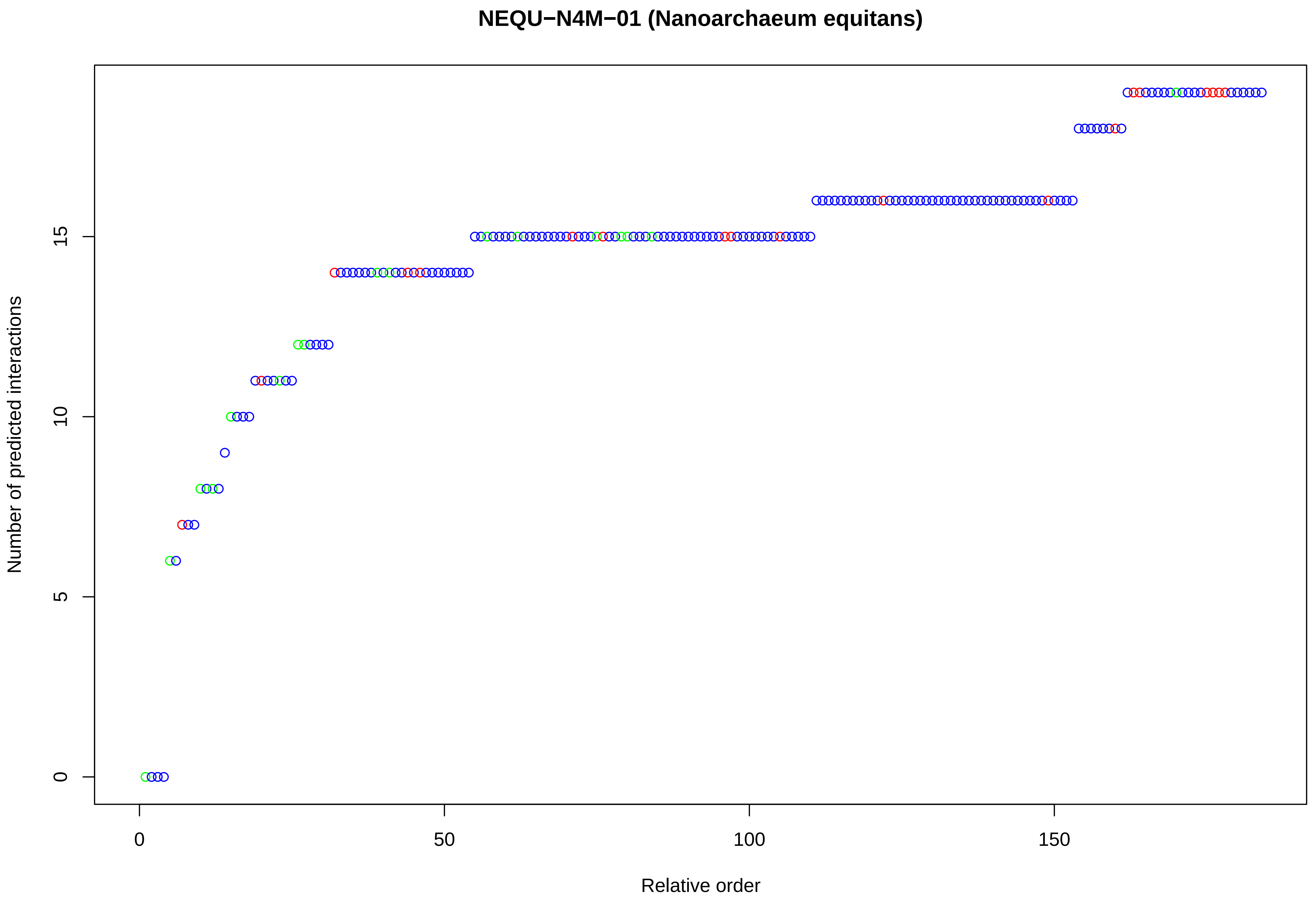
NEQU−N4M−01 (Nanoarchaeum equitans)
Number of predicted interactions
Relative order
0
5
10
15
0
50
100
150

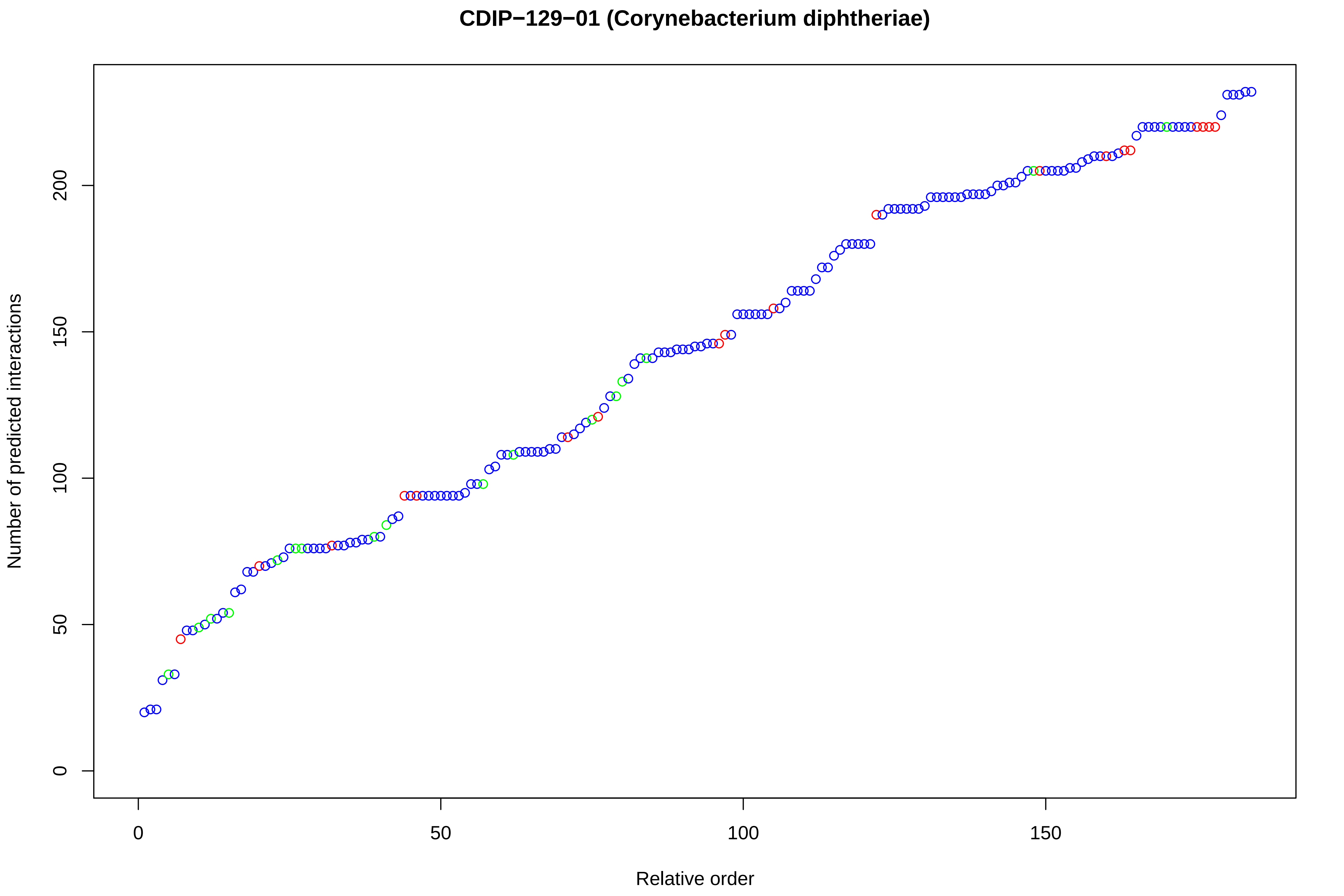
CDIP−129−01 (Corynebacterium diphtheriae)
Number of predicted interactions
0
50
100
150
200
0
50
100
150
Relative order

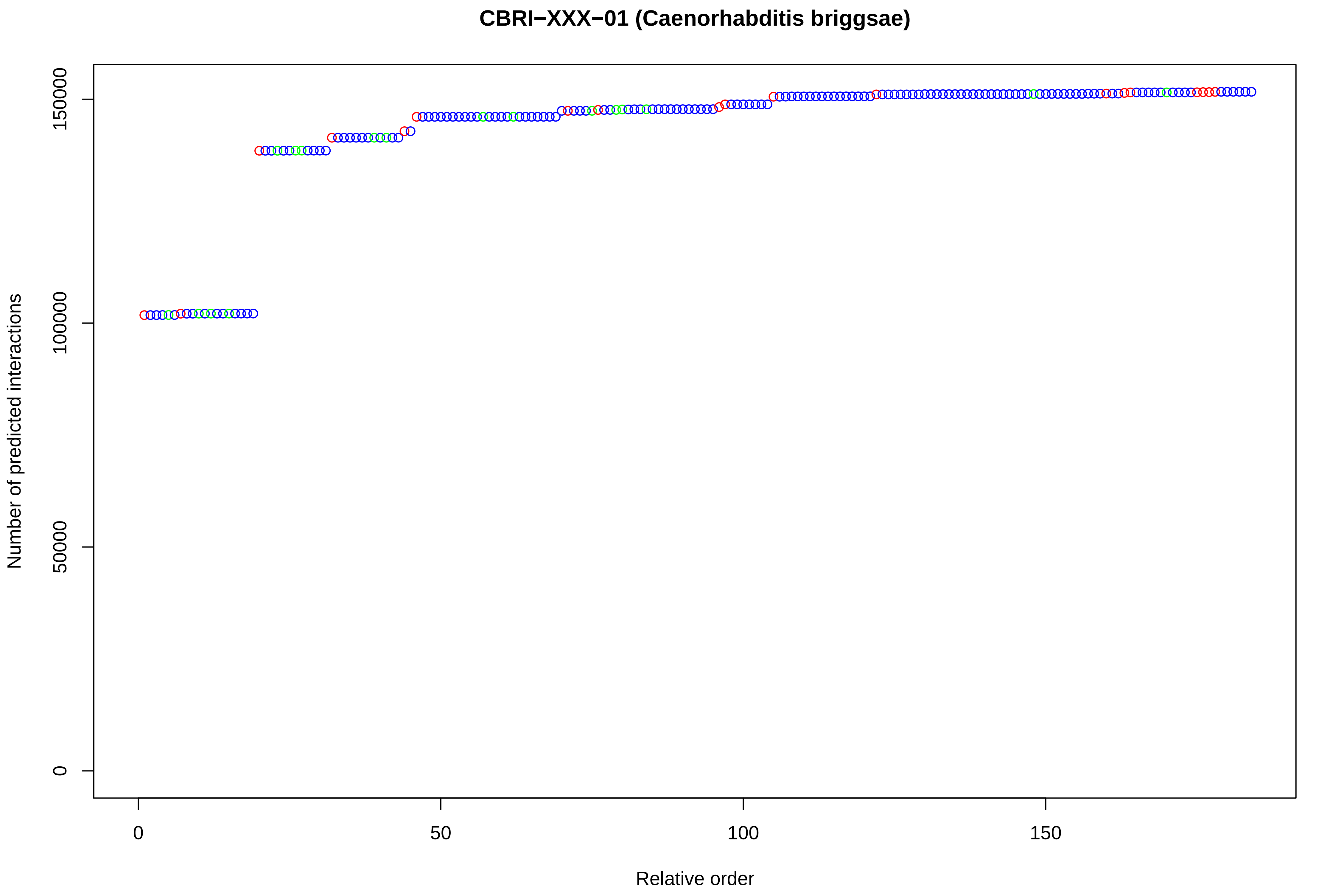

CBRI−XXX−01 (Caenorhabditis briggsae)
Number of predicted interactions
0
50000
100000
150000
Relative order
0
50
100
150

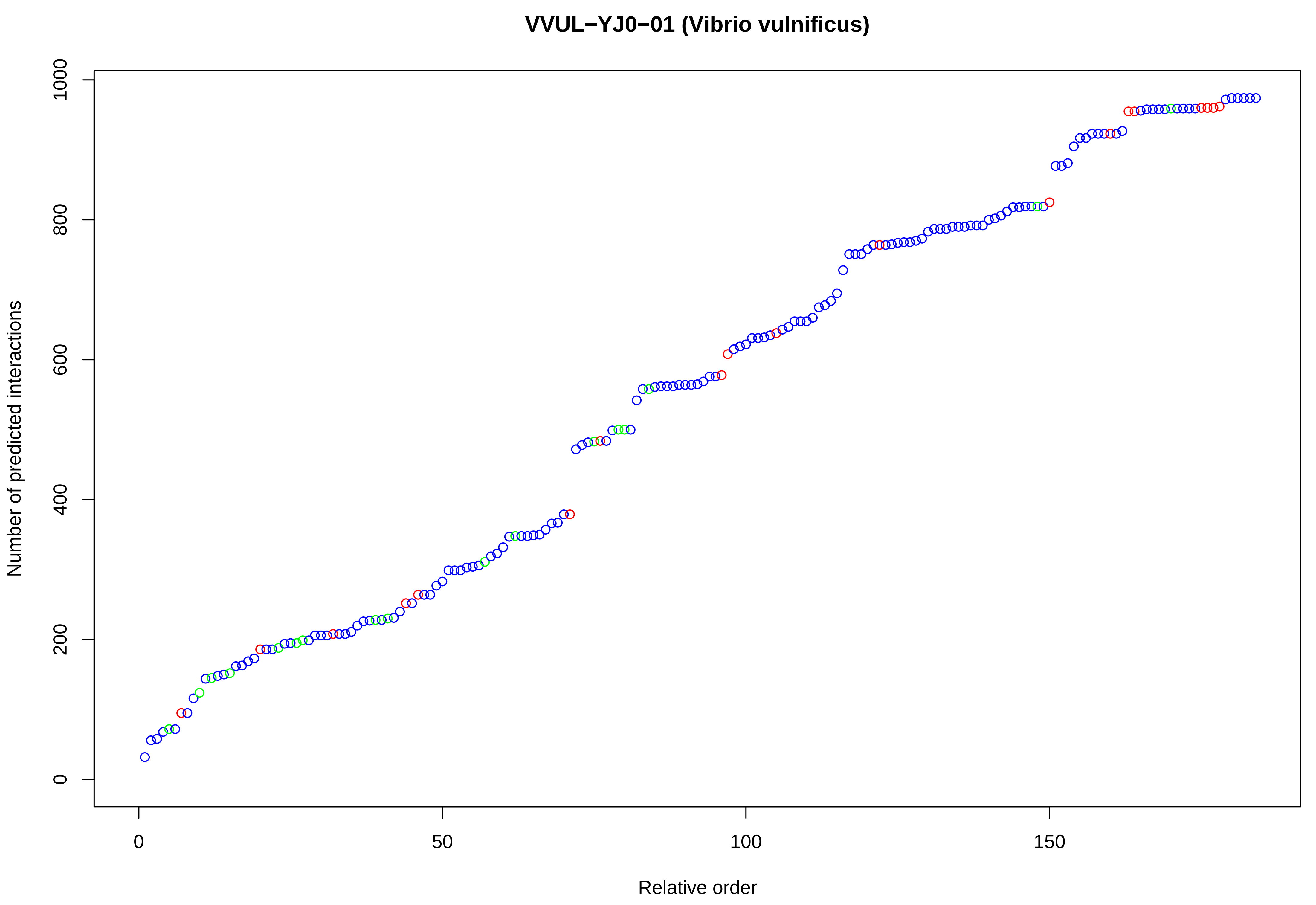
VVUL−YJ0−01 (Vibrio vulnificus)
Number of predicted interactions
Relative order
0
200
400
600
800
1000
0
50
100
150

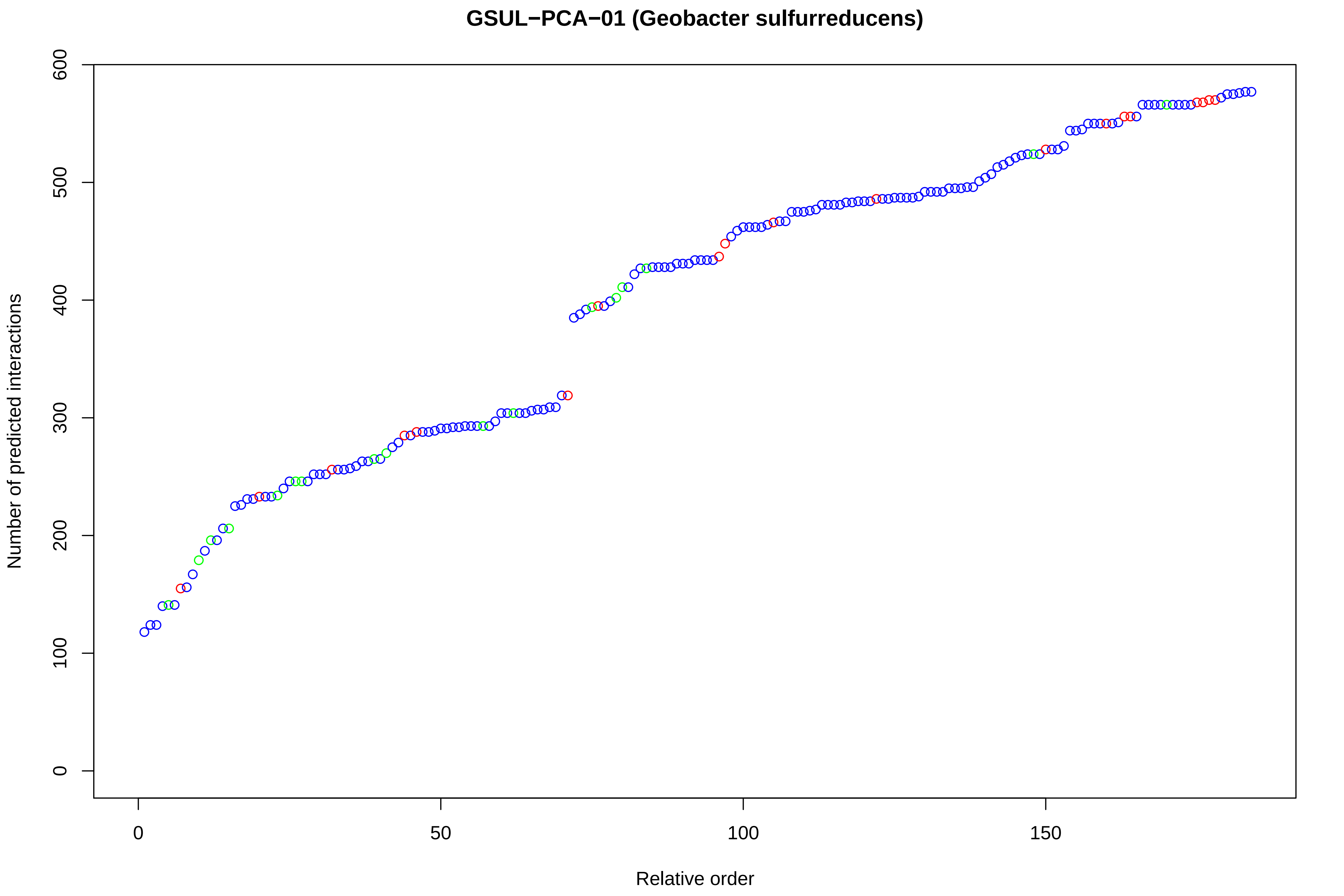
GSUL−PCA−01 (Geobacter sulfurreducens)
Number of predicted interactions
Relative order
0
100
200
300
400
500
600
0
50
100
150

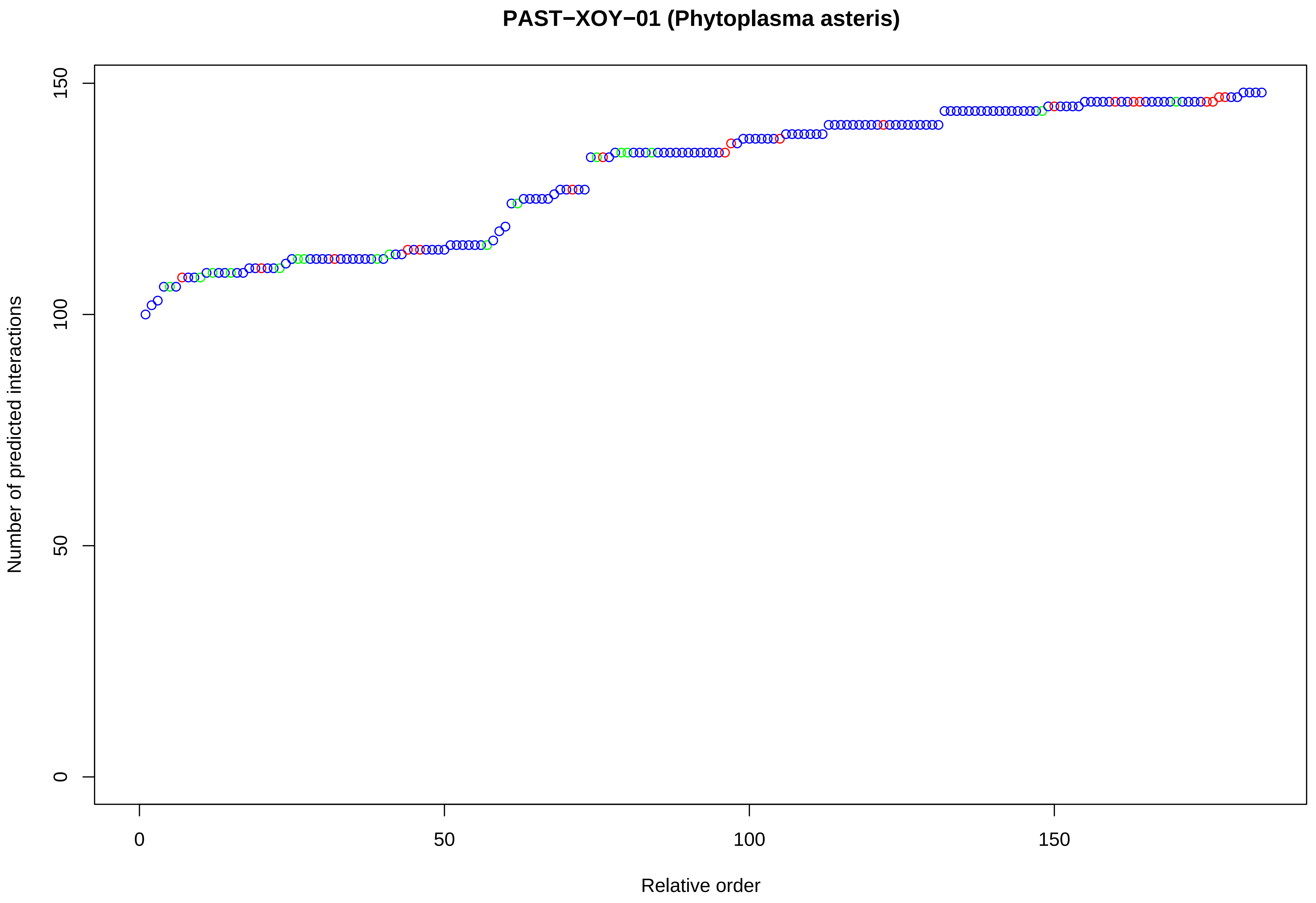
PAST−XOY−01 (Phytoplasma asteris)
Number of predicted interactions
Relative order
0
50
100
150

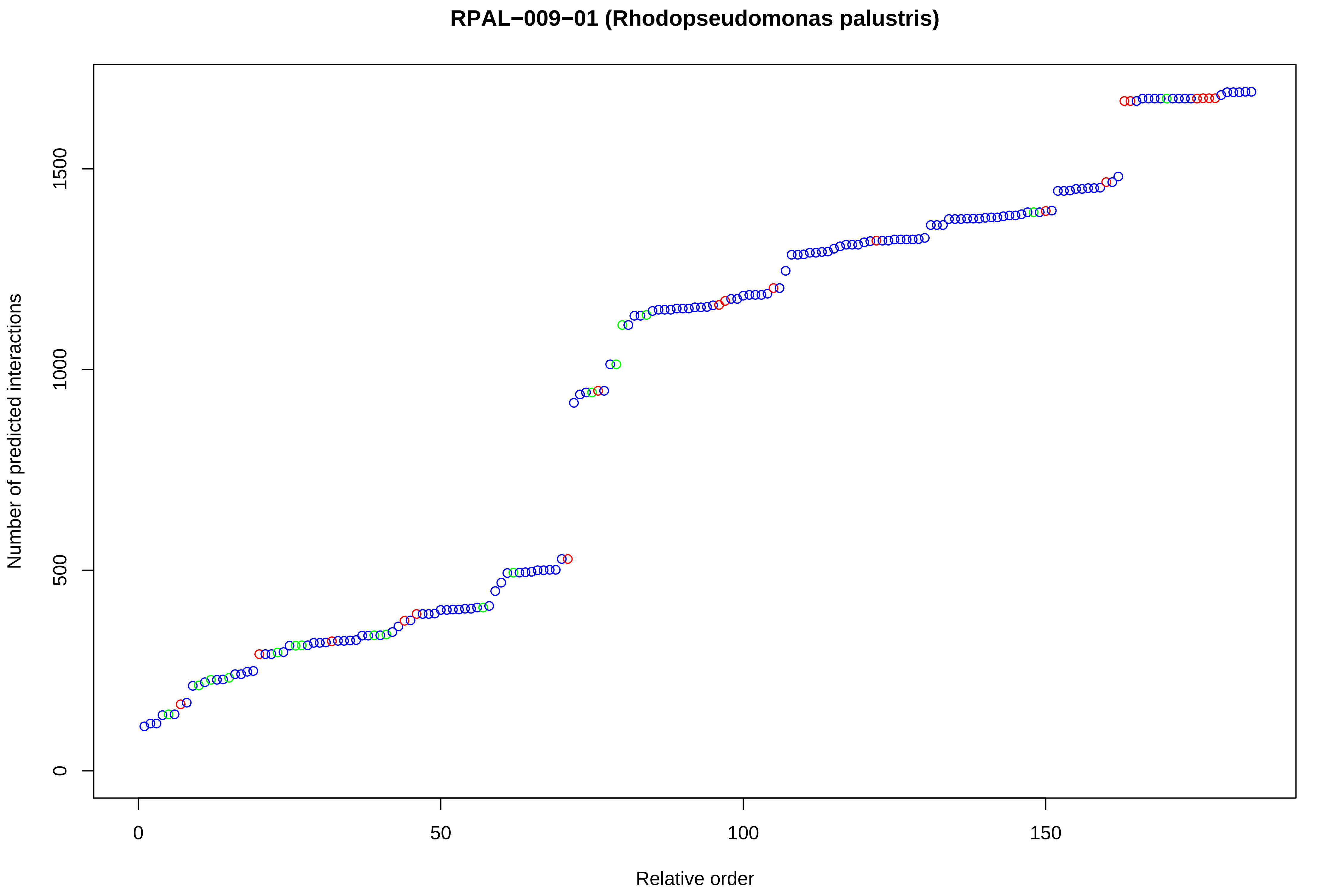
RPAL−009−01 (Rhodopseudomonas palustris)
Number of predicted interactions
0
500
1000
1500
Relative order
0
50
100
150

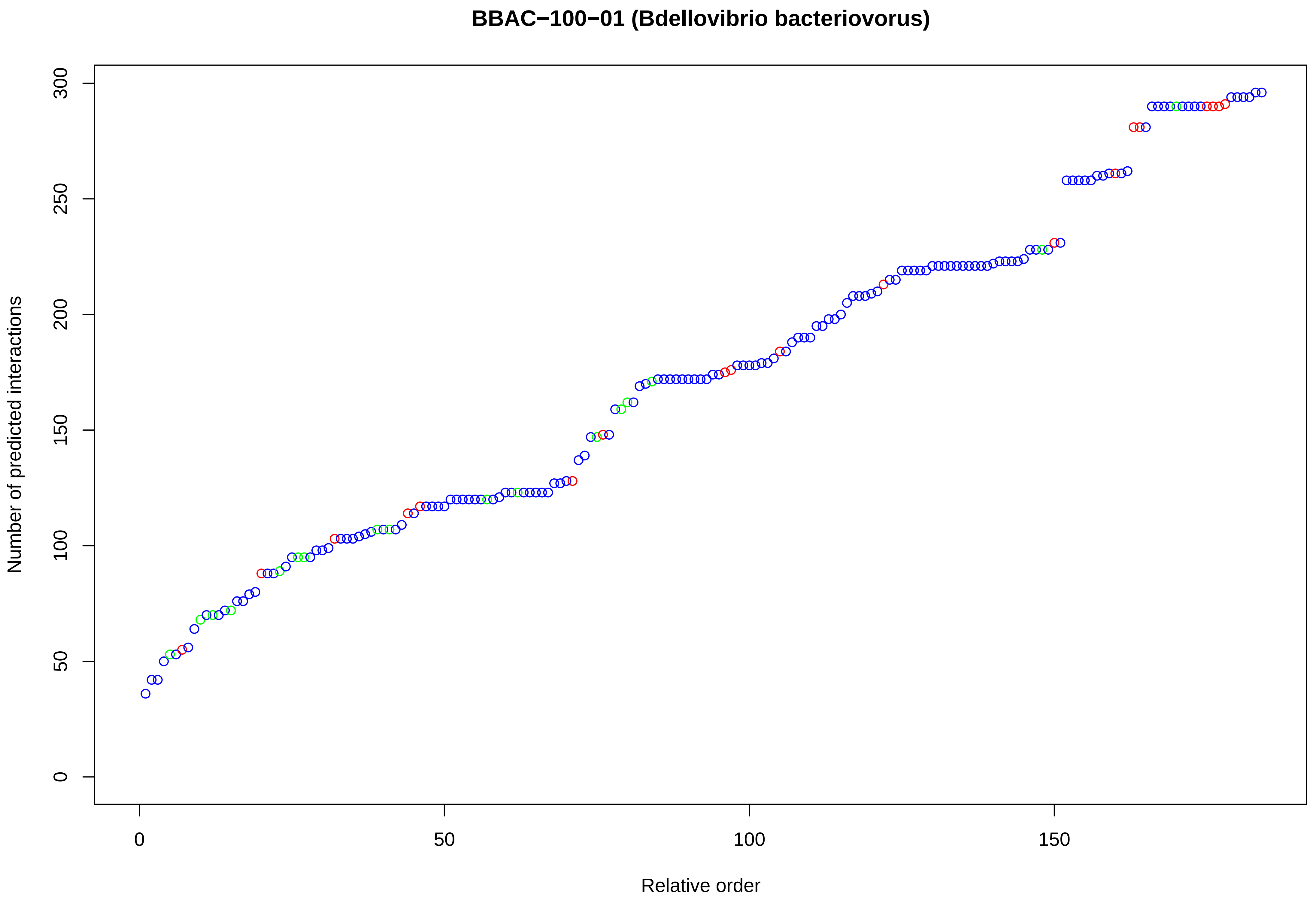
BBAC−100−01 (Bdellovibrio bacteriovorus)
Number of predicted interactions
Relative order
0
50
100
150
200
250
300
0
50
100
150

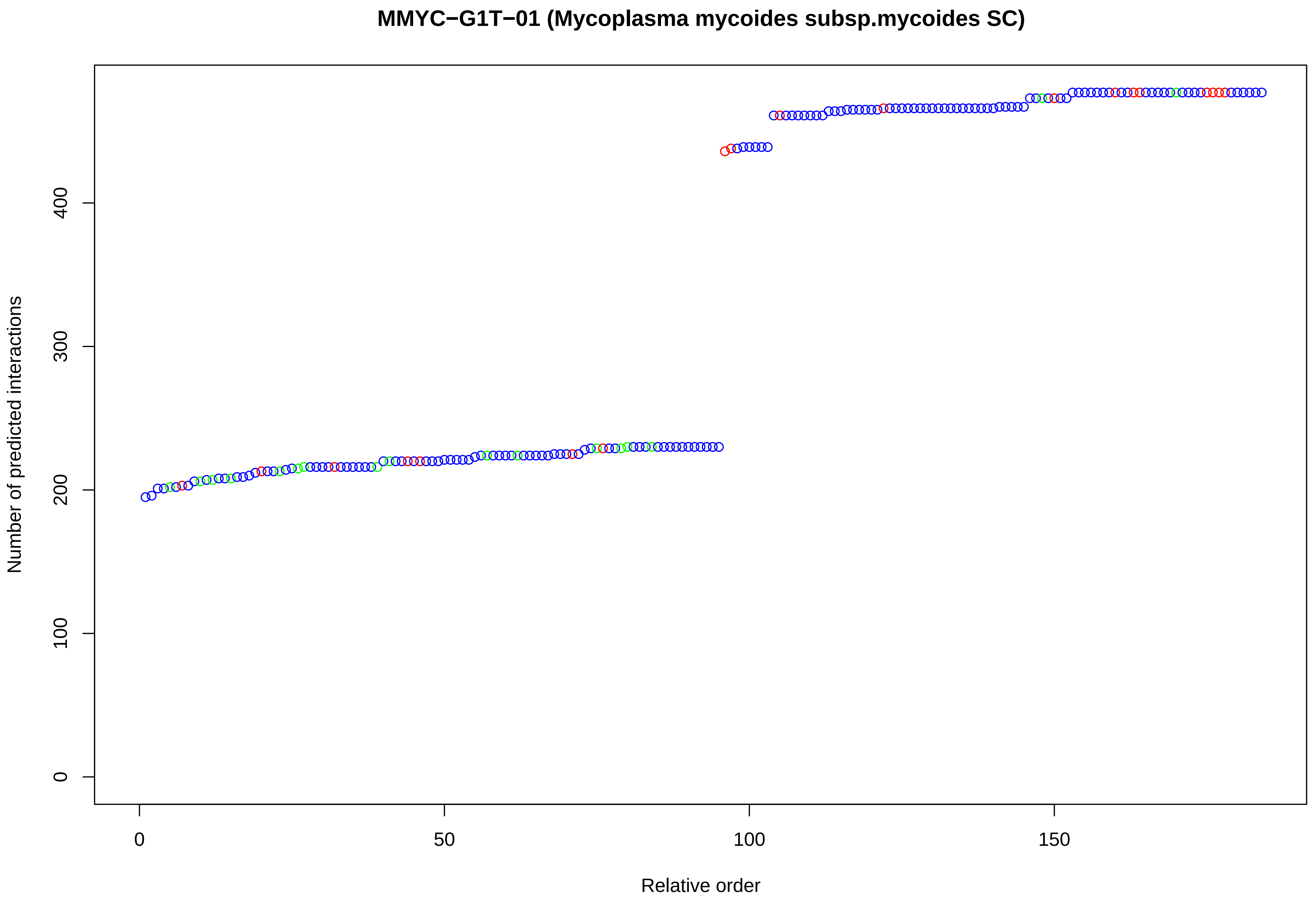

MMYC−G1T−01 (Mycoplasma mycoides subsp.mycoides SC)
Number of predicted interactions
0
100
200
300
400
0
50
100
150
Relative order

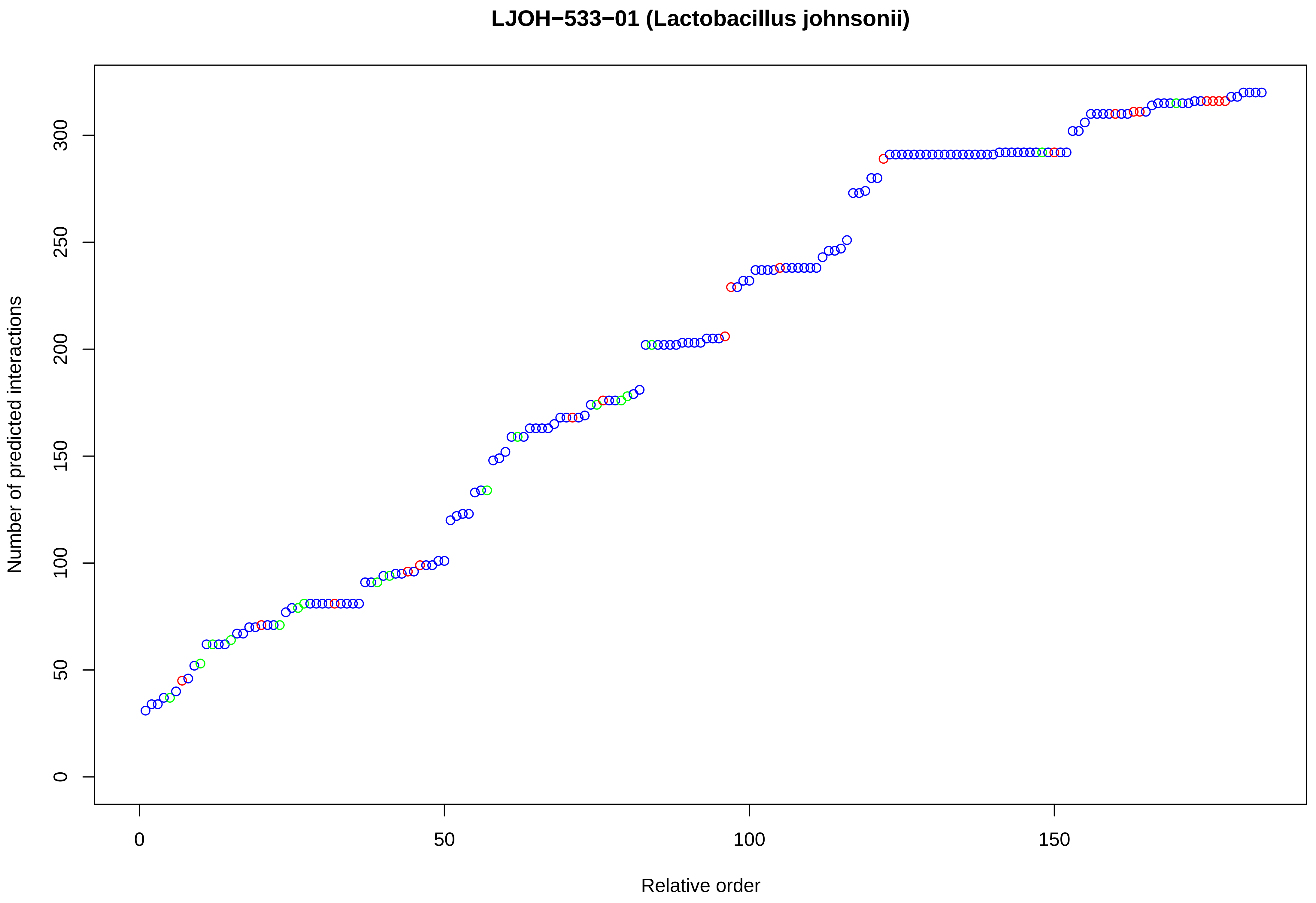

LJOH−533−01 (Lactobacillus johnsonii)
Number of predicted interactions
0
50
100
150
200
250
300
Relative order
0
50
100
150

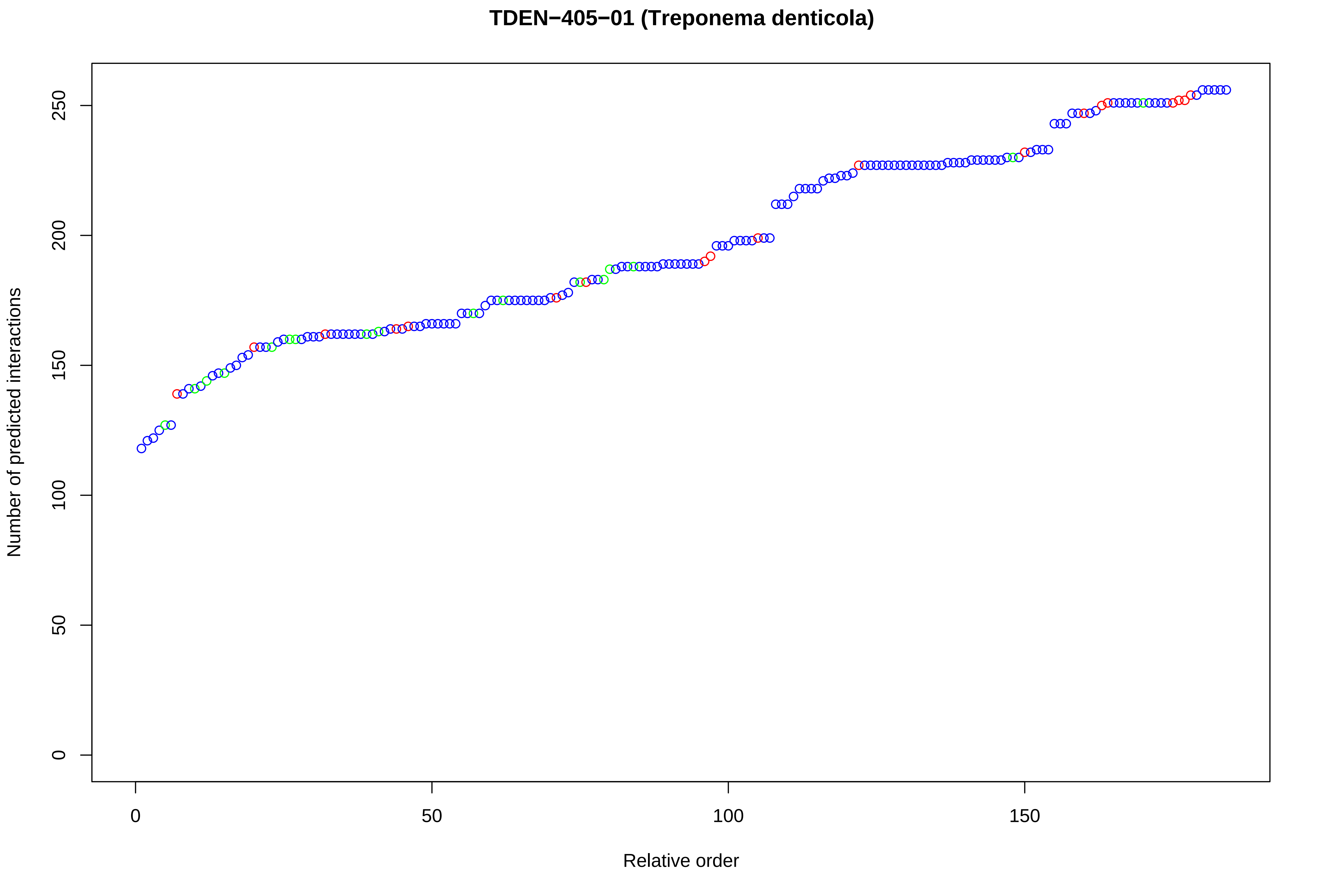
TDEN−405−01 (Treponema denticola)
Number of predicted interactions
Relative order
0
50
100
150
200
250
0
50
100
150

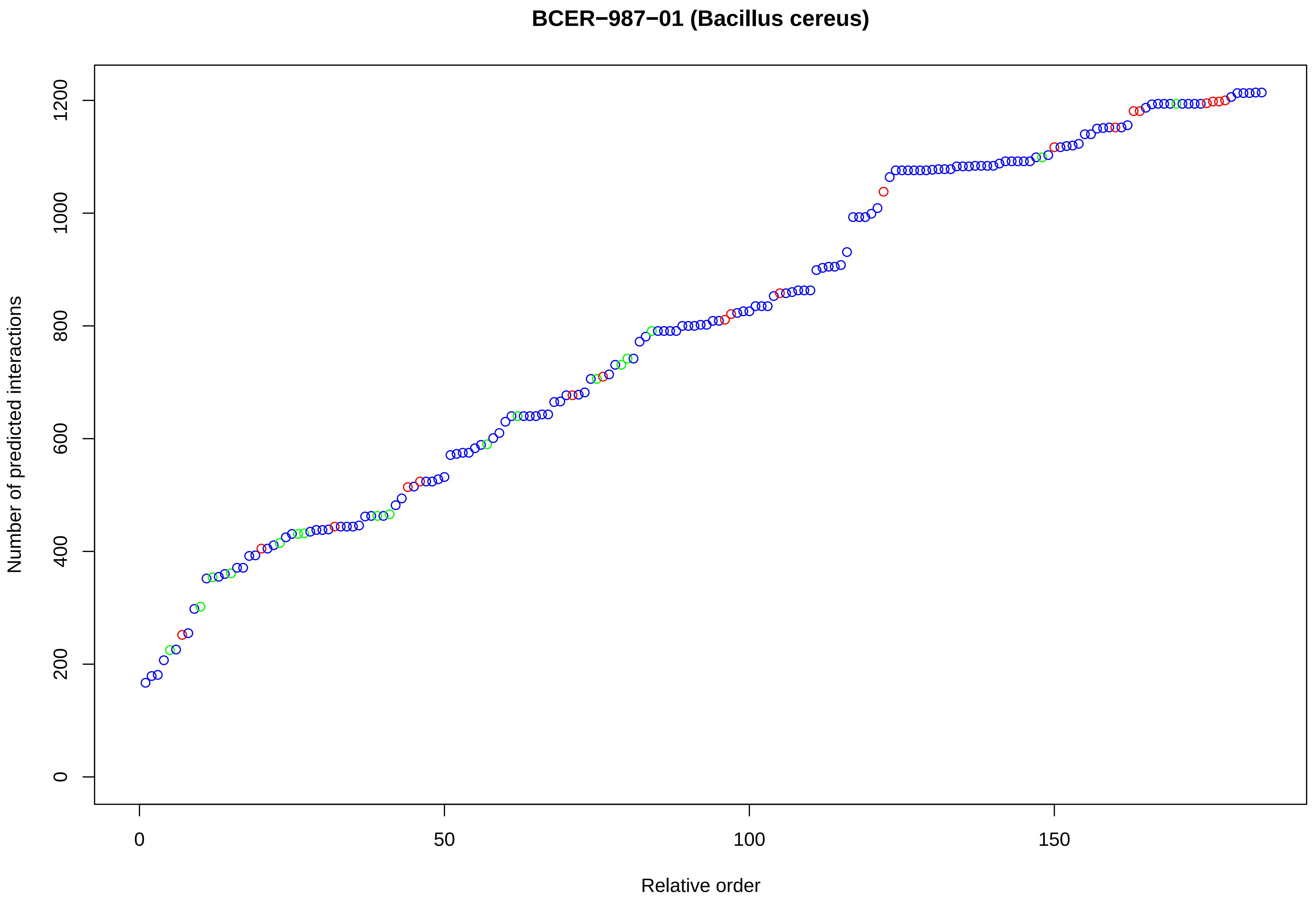

BCER−987−01 (Bacillus cereus)
Number of predicted interactions
Relative order
0
200
400
600
800
1000
1200
0
50
100
150

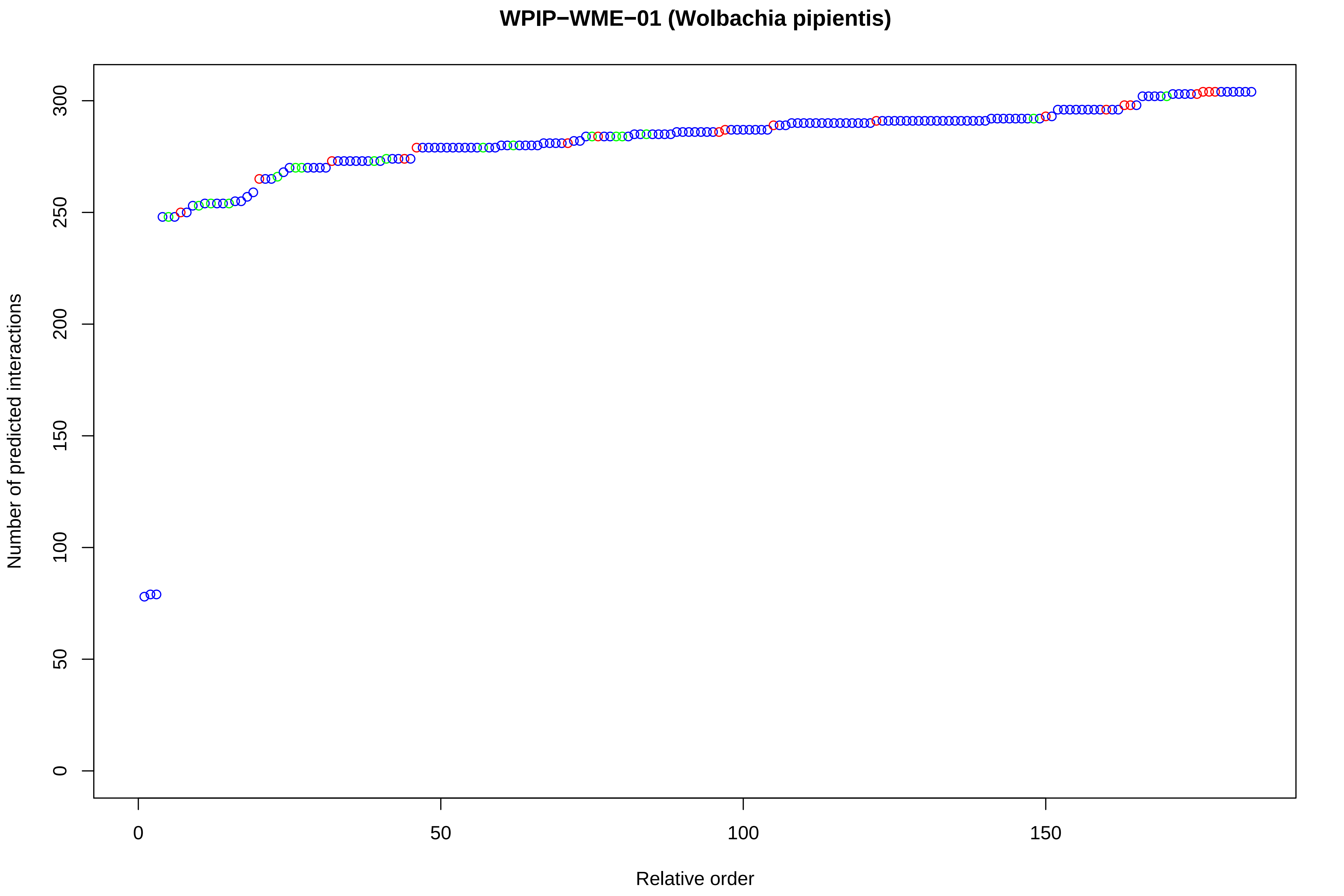

WPIP−WME−01 (Wolbachia pipientis)
Number of predicted interactions
Relative order
0
50
100
150
200
250
300
0
50
100
150

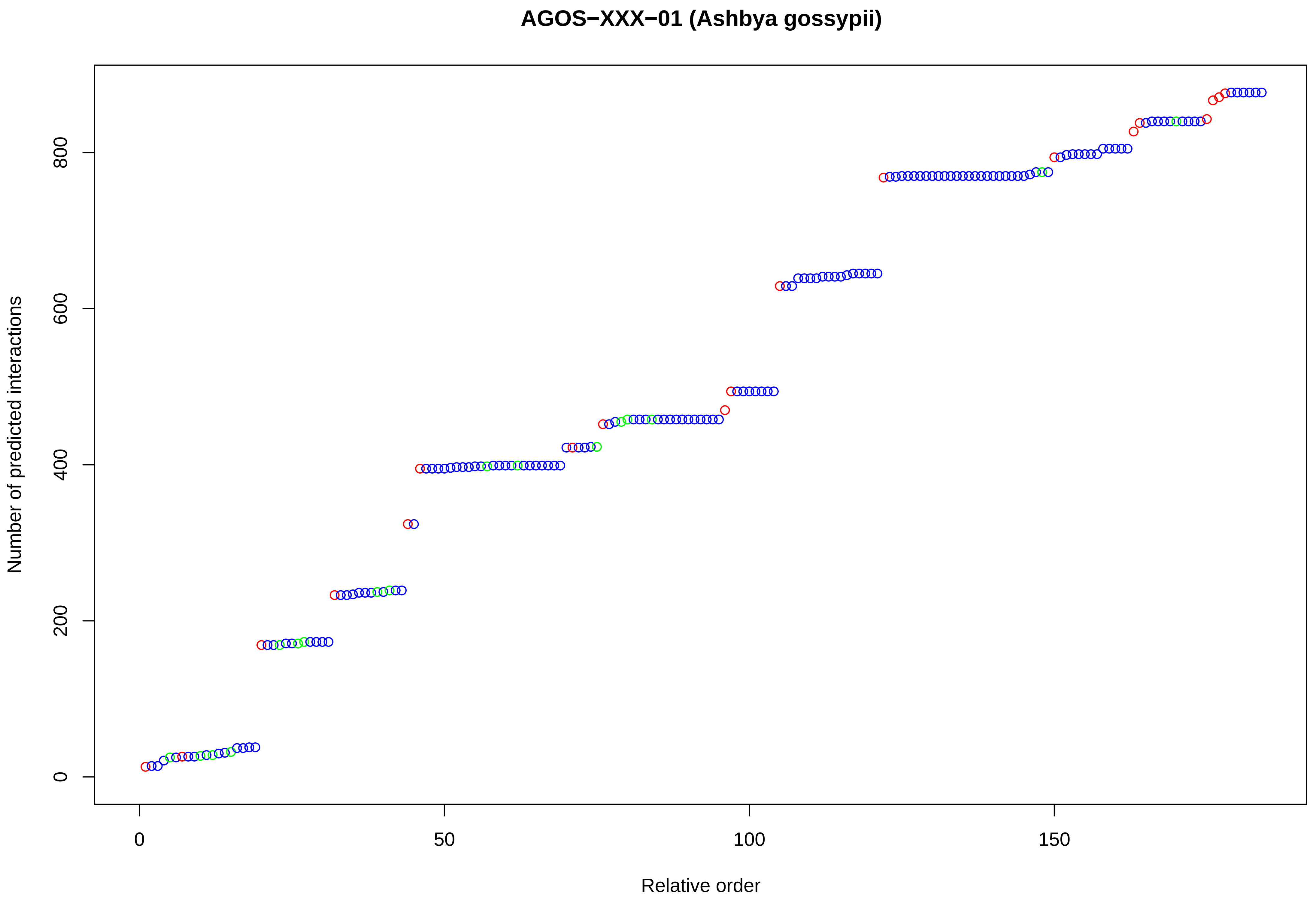
AGOS−XXX−01 (Ashbya gossypii)
Number of predicted interactions
0
200
400
600
800
0
50
100
150
Relative order

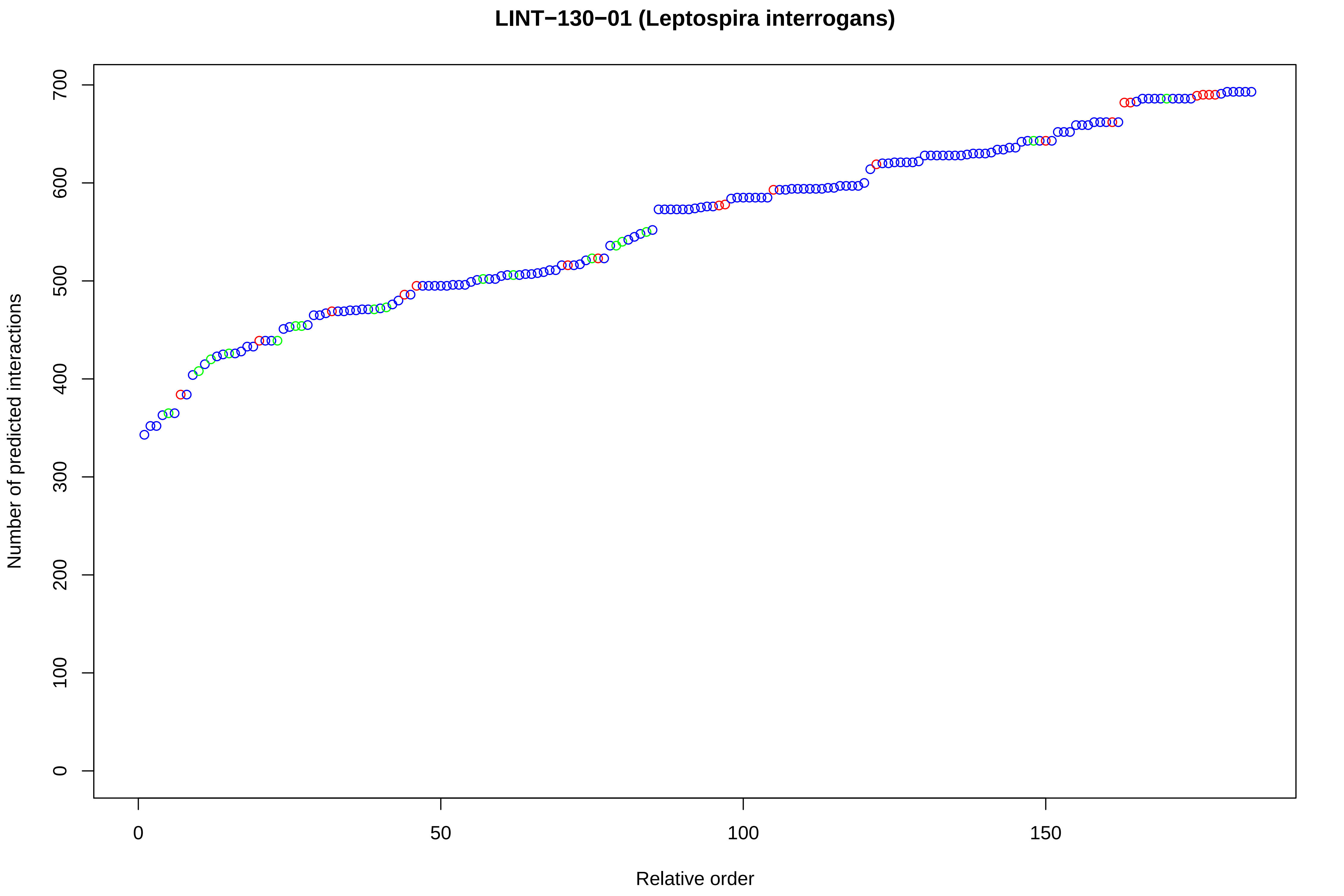

LINT−130−01 (Leptospira interrogans)
Number of predicted interactions
0
100
200
300
400
500
600
700
Relative order
0
50
100
150

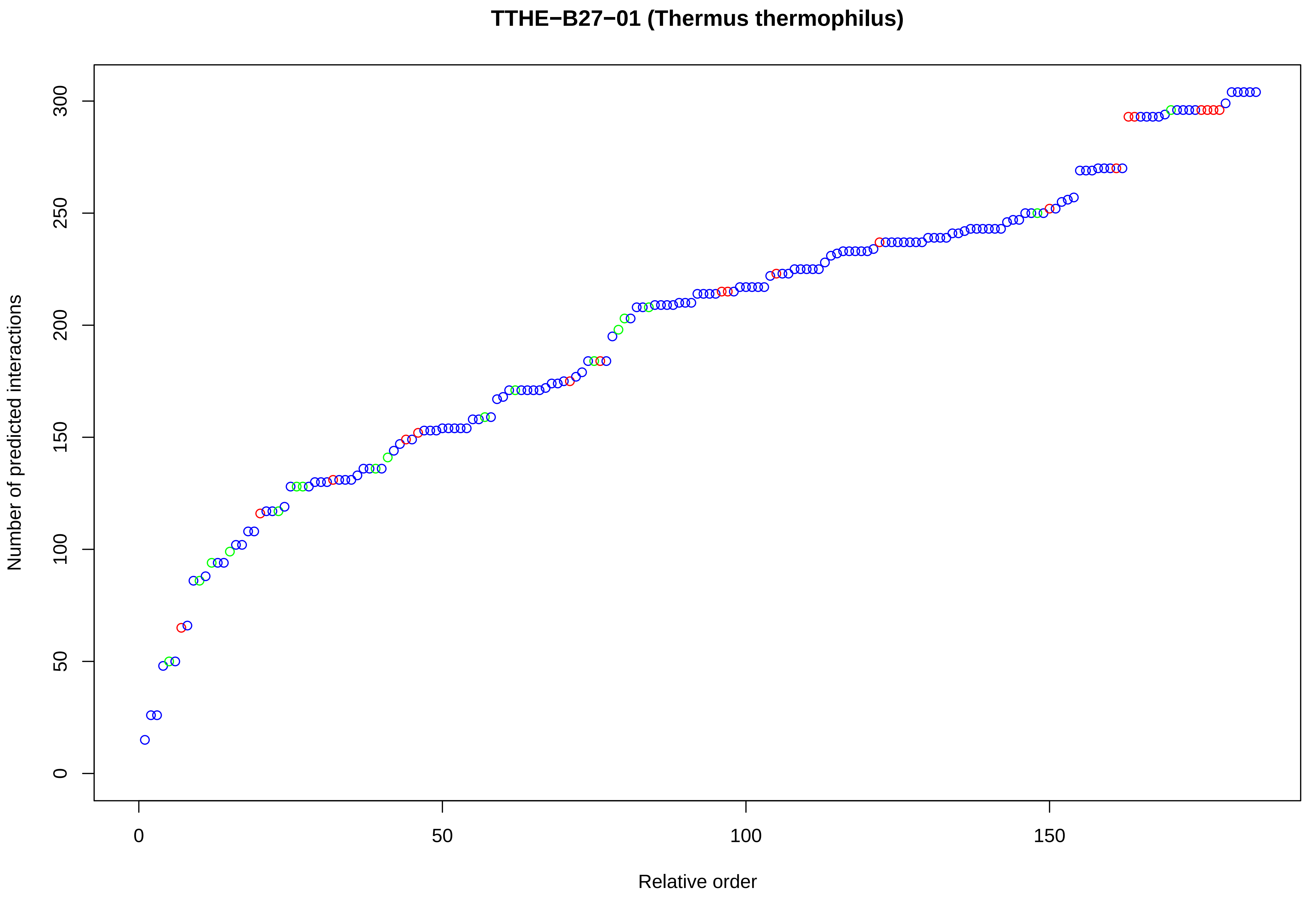

TTHE−B27−01 (Thermus thermophilus)
Number of predicted interactions
0
50
100
150
200
250
300
Relative order
0
50
100
150

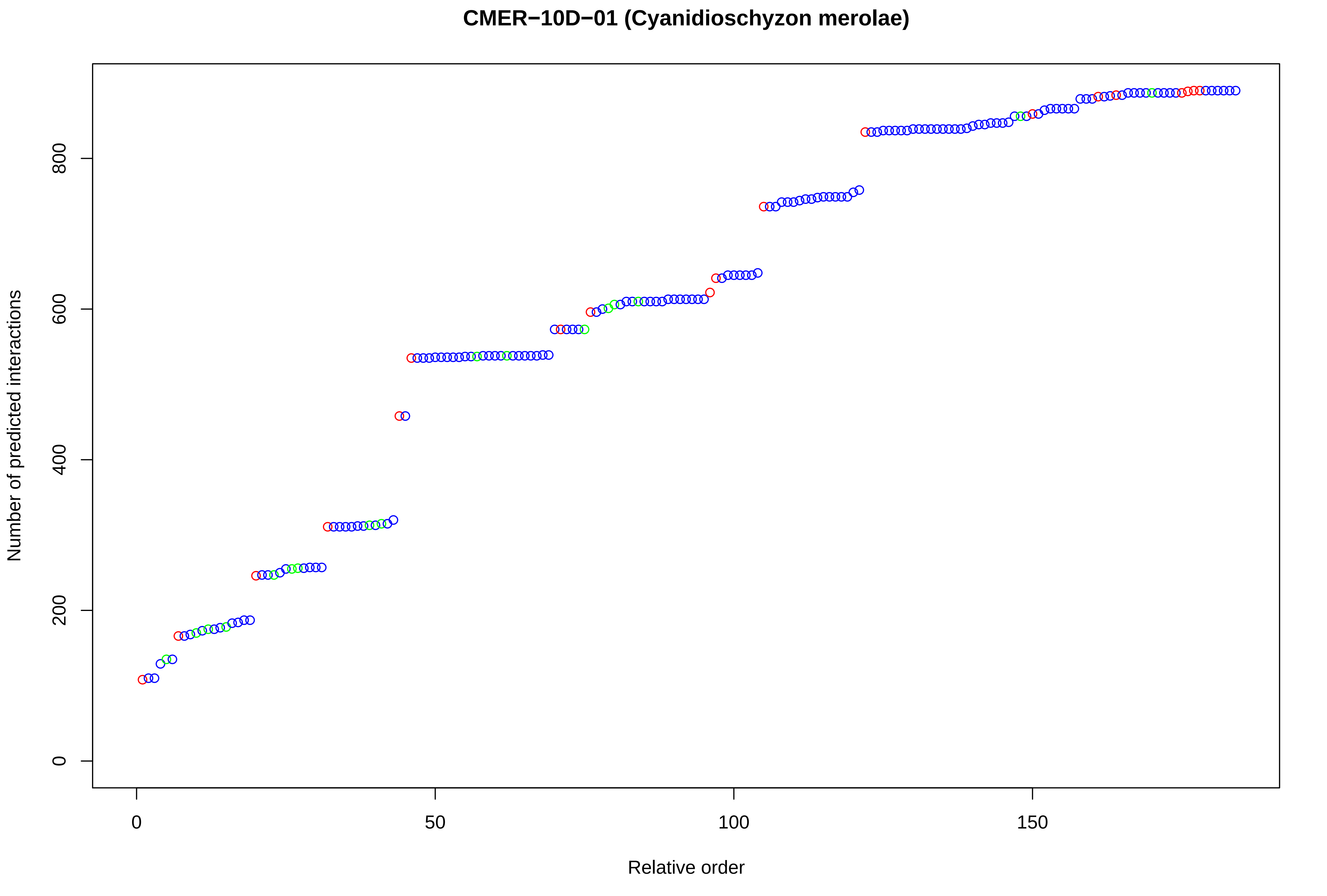
CMER−10D−01 (Cyanidioschyzon merolae)
Number of predicted interactions
0
200
400
600
800
0
50
100
150
Relative order

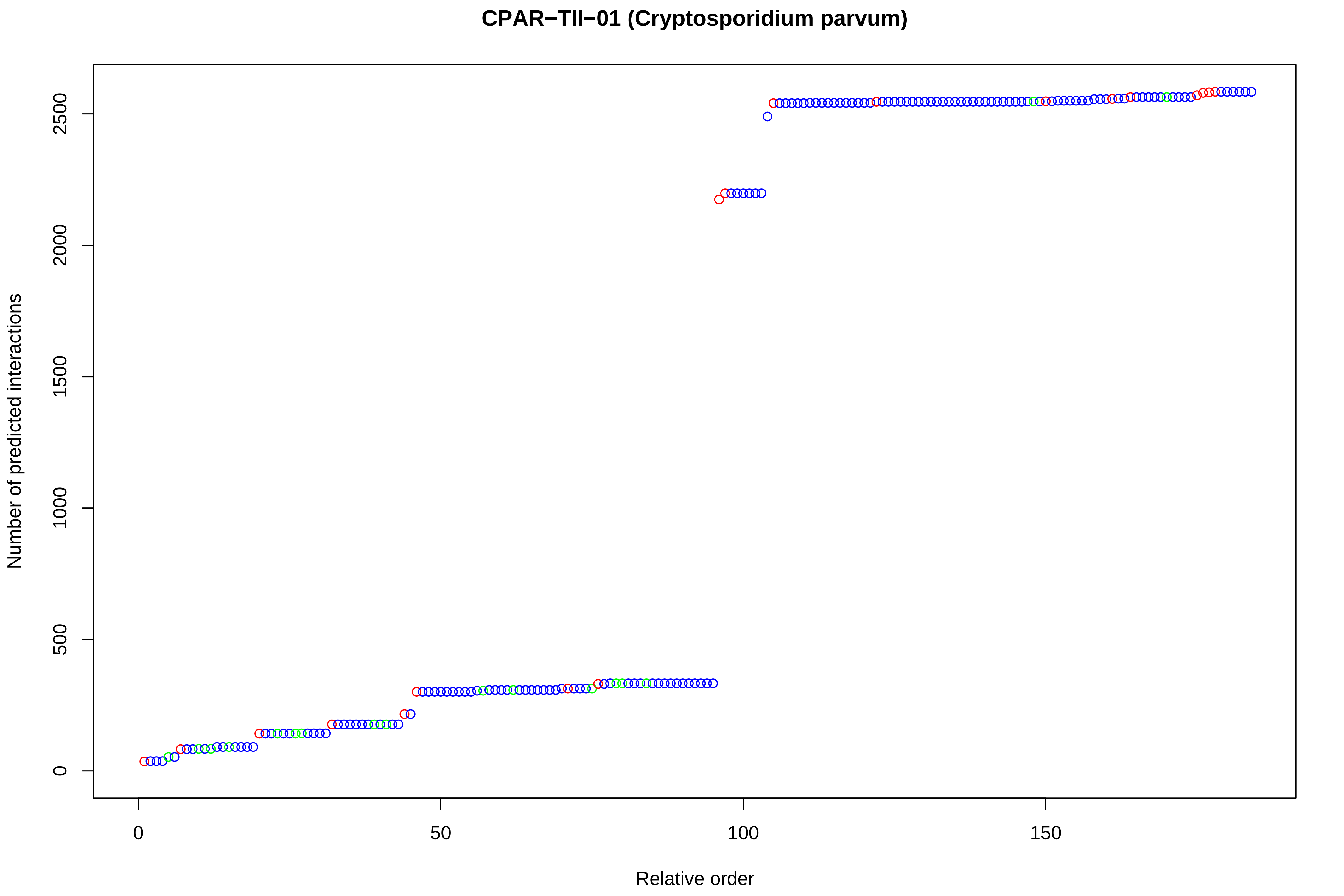

CPAR−TII−01 (Cryptosporidium parvum)
Number of predicted interactions
0
500
1000
1500
2000
2500
0
50
100
150
Relative order

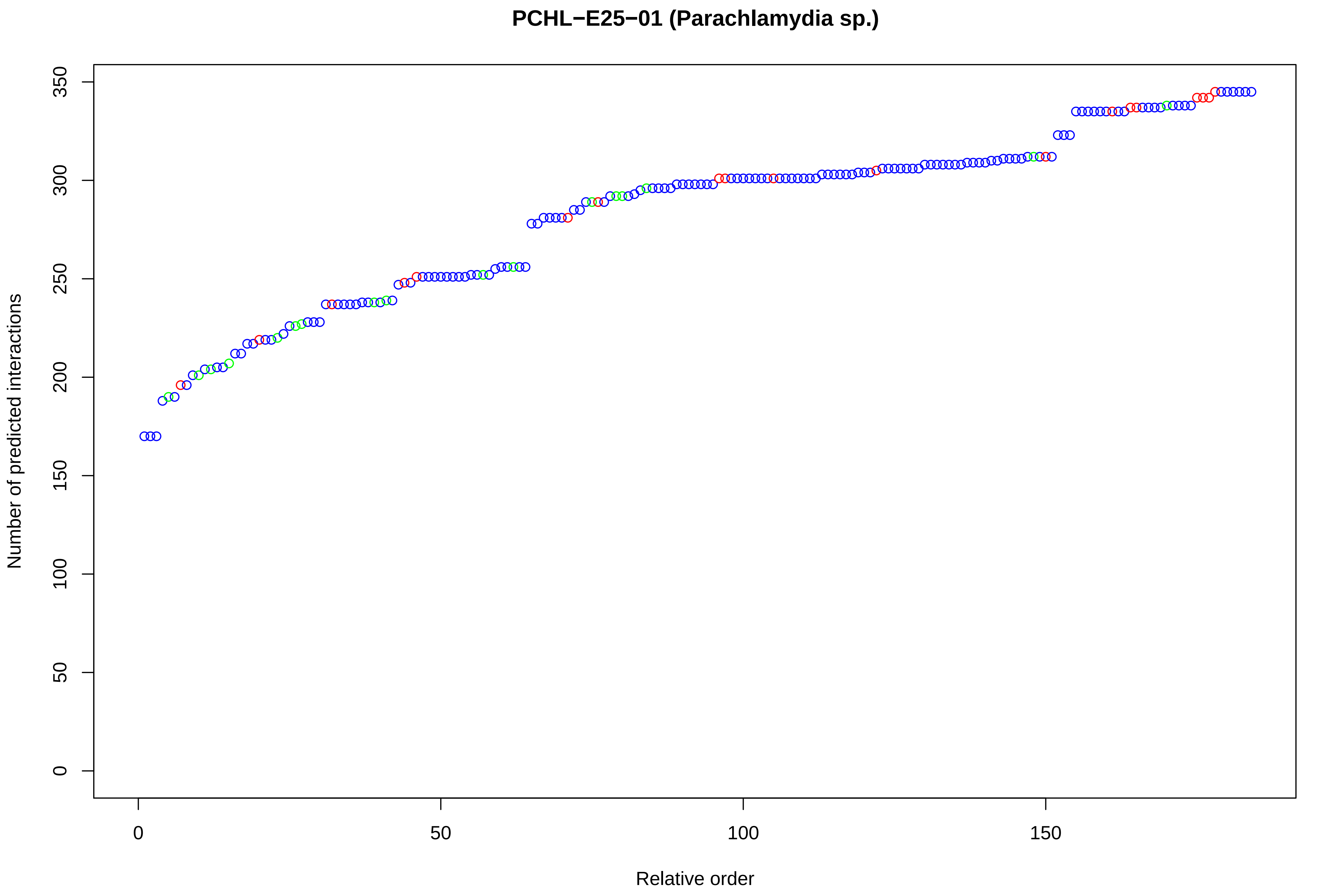
PCHL−E25−01 (Parachlamydia sp.)
Number of predicted interactions
Relative order
0
50
100
150
200
250
300
350

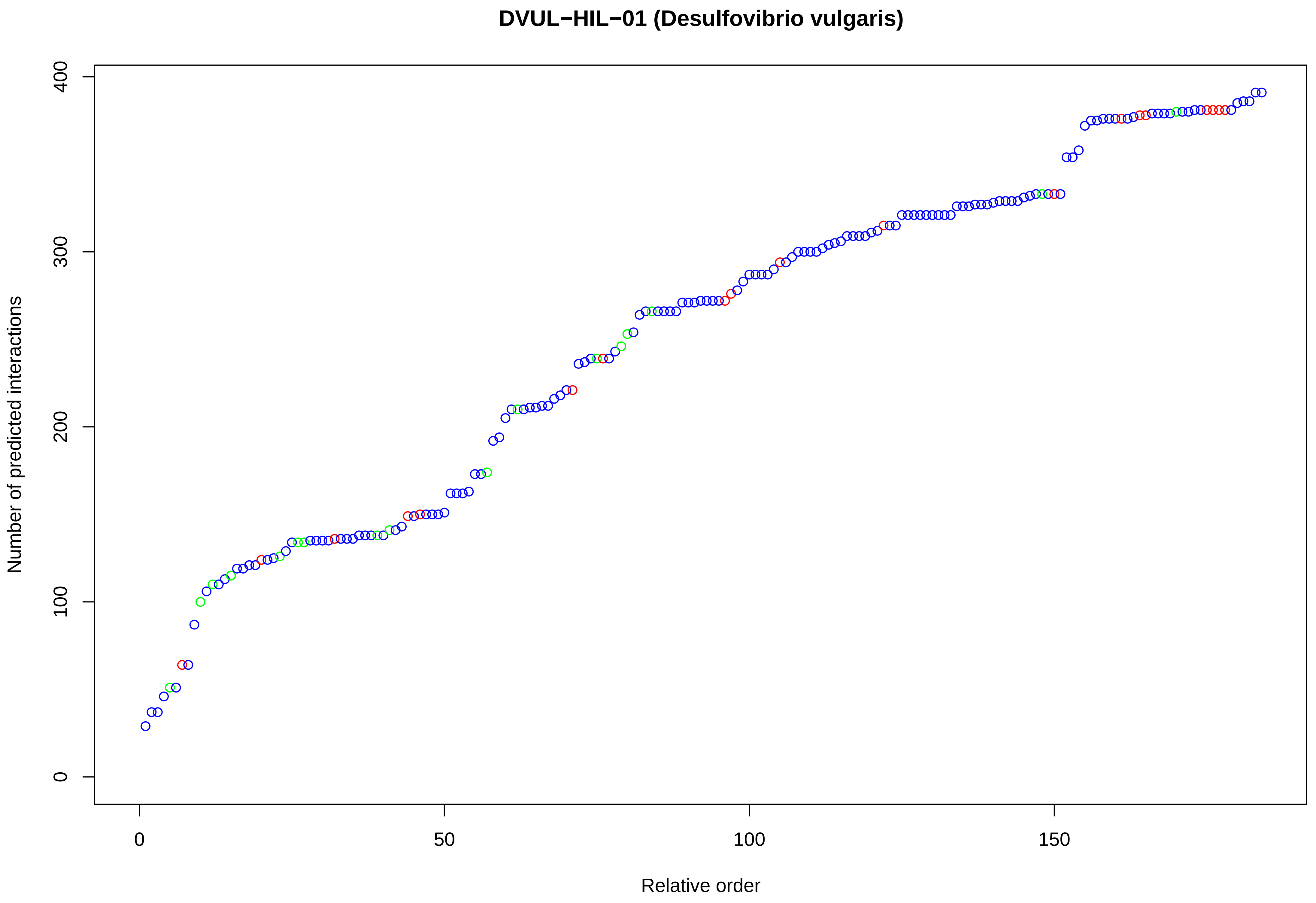

DVUL−HIL−01 (Desulfovibrio vulgaris)
Number of predicted interactions
Relative order
0
100
200
300
400
0
50
100
150

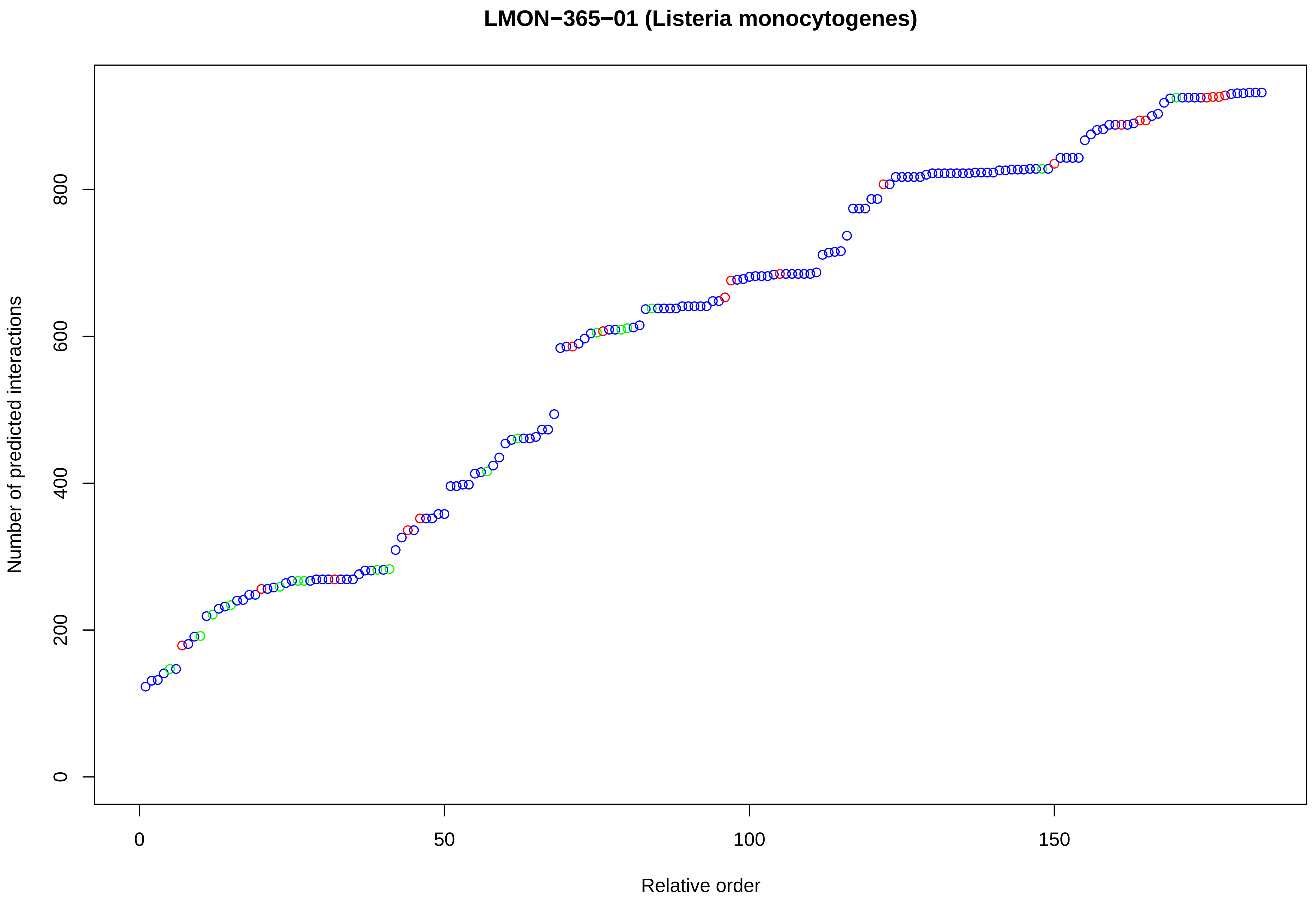
LMON−365−01 (Listeria monocytogenes)
Number of predicted interactions
Relative order
0
200
400
600
800
0
50
100
150

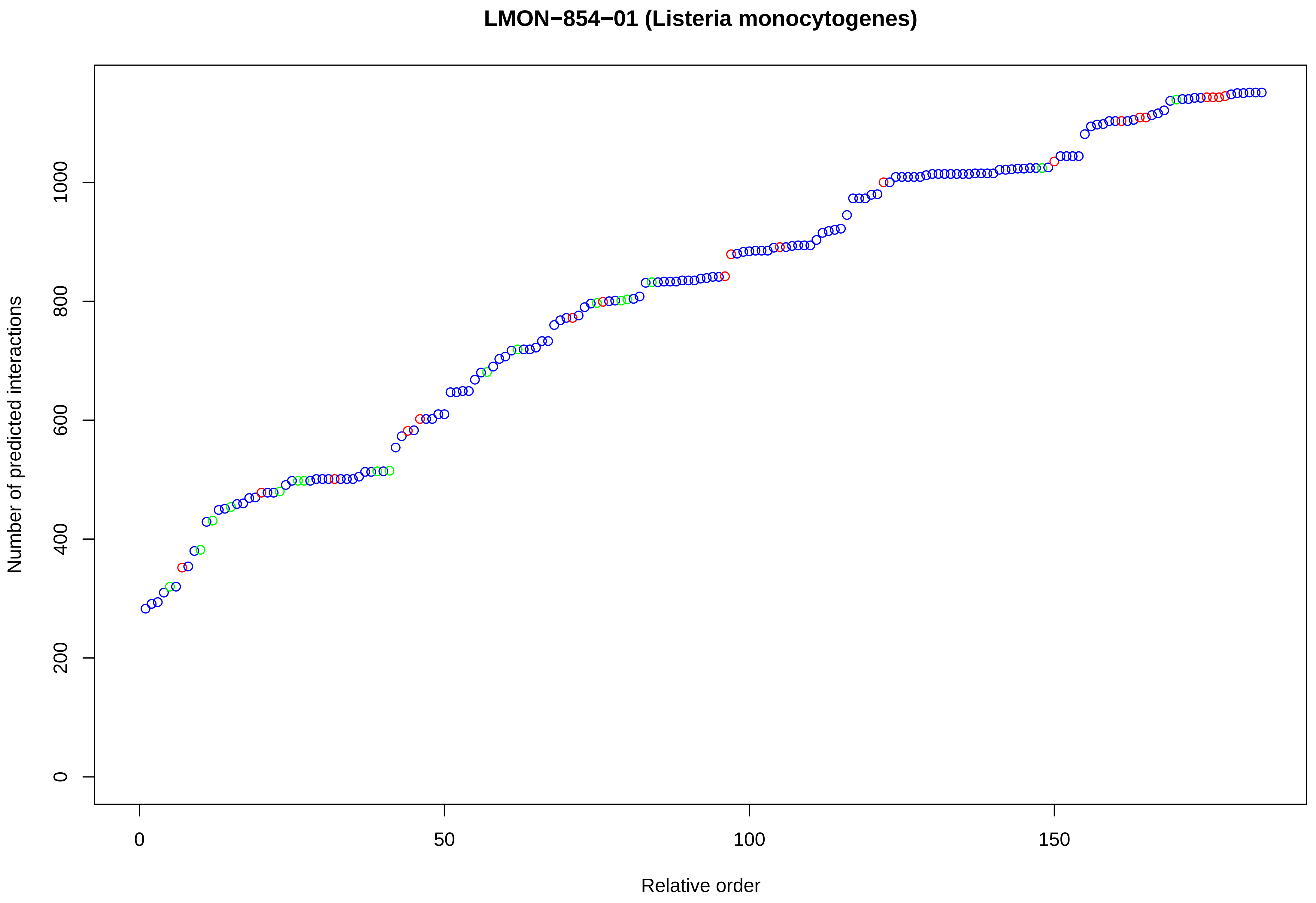

LMON−854−01 (Listeria monocytogenes)
Number of predicted interactions
0
200
400
600
800
1000
Relative order
0
50
100
150

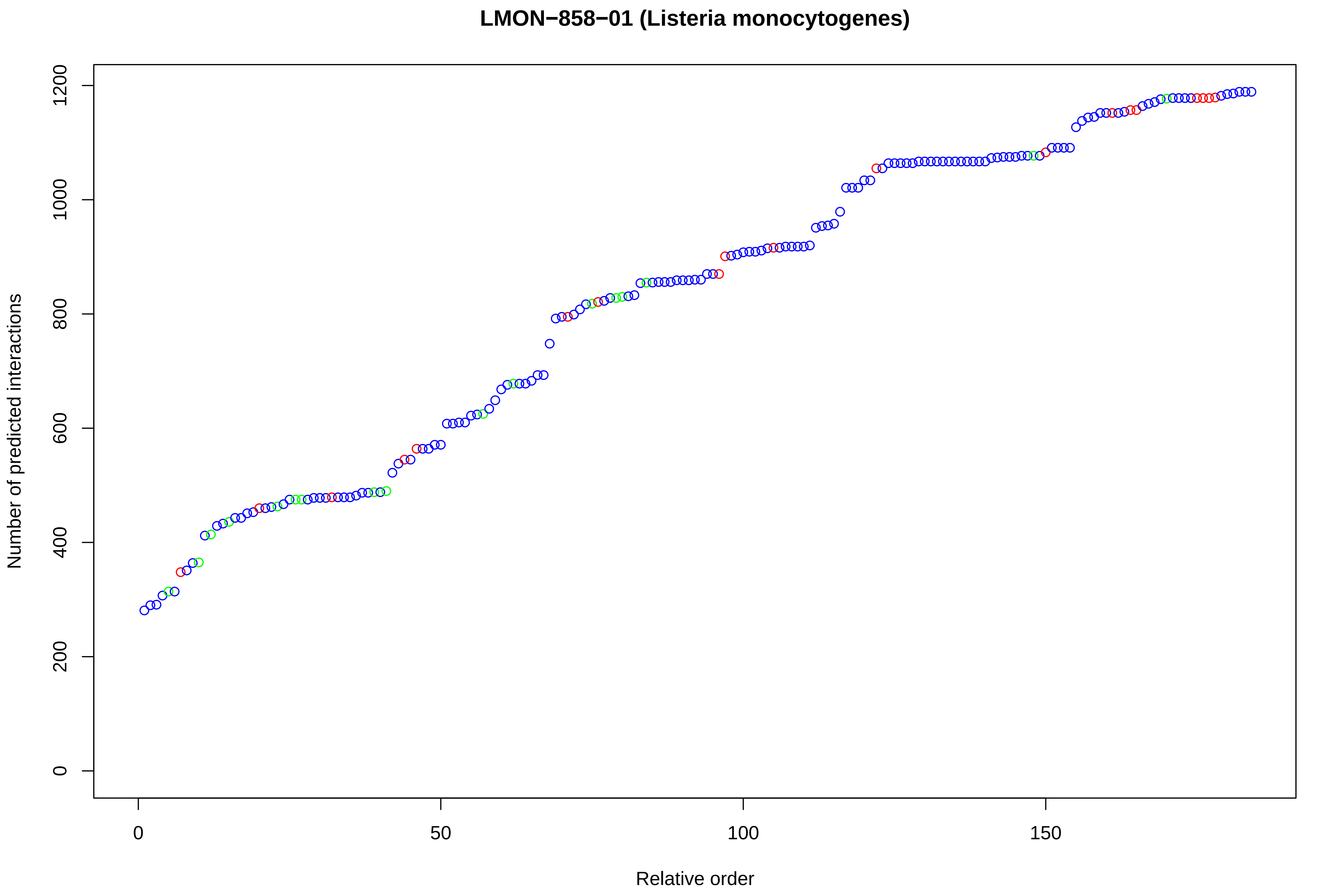

LMON−858−01 (Listeria monocytogenes)
Number of predicted interactions
0
200
400
600
800
1000
1200
Relative order
0
50
100
150

PTOR−790−01 (Picrophilus torridus)

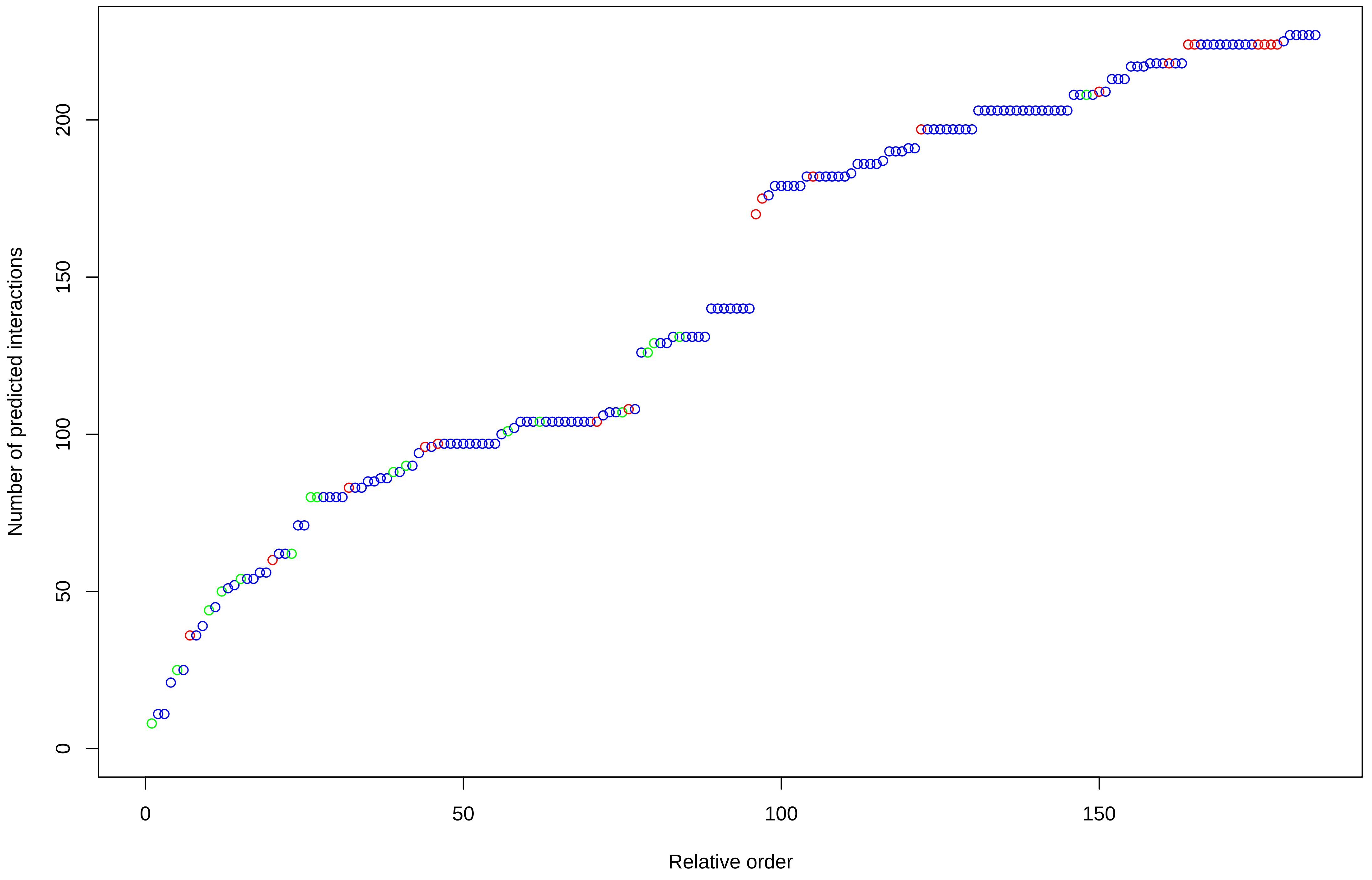

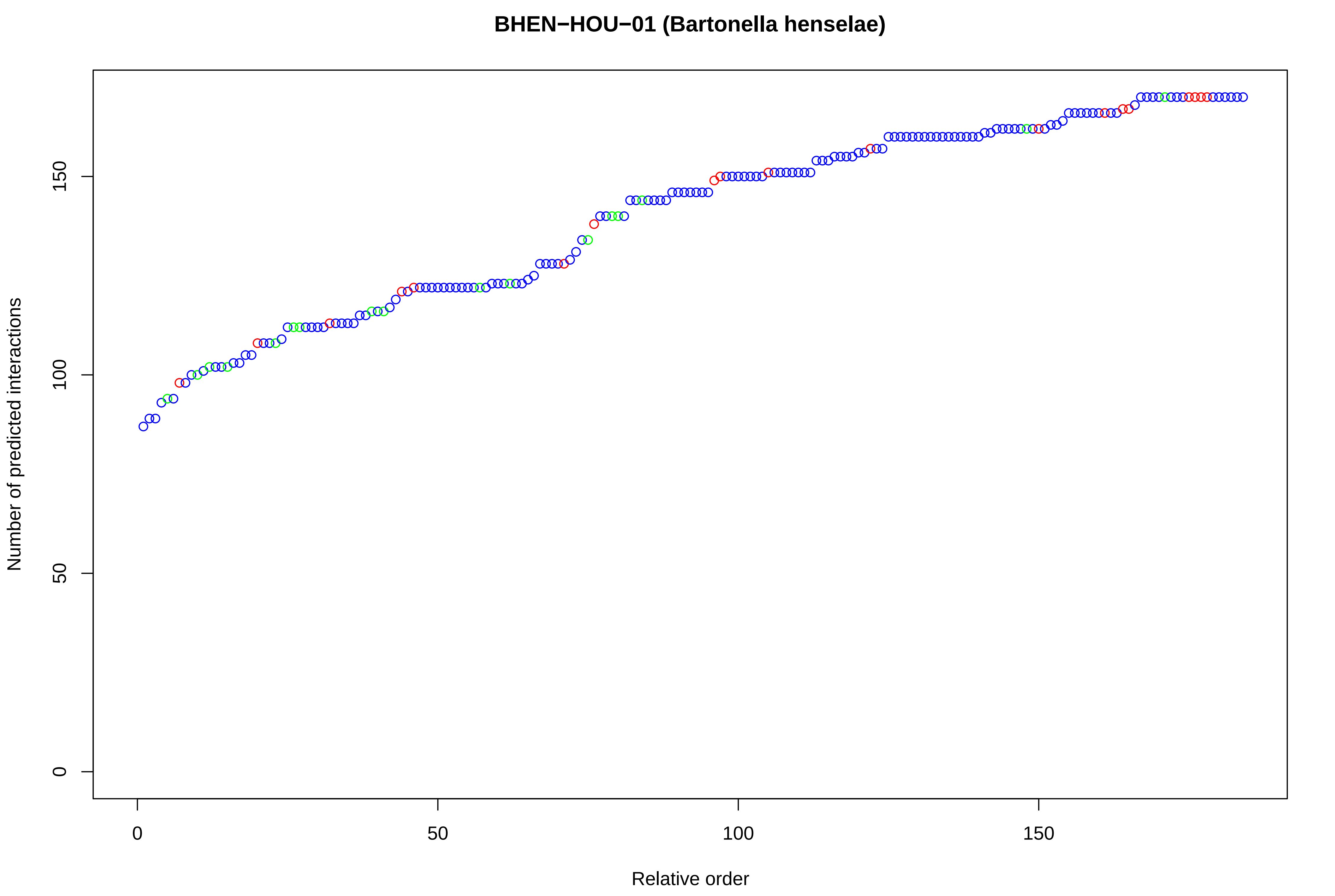
BHEN−HOU−01 (Bartonella henselae)
Number of predicted interactions
Relative order
0
50
100
150
0
50
100
150

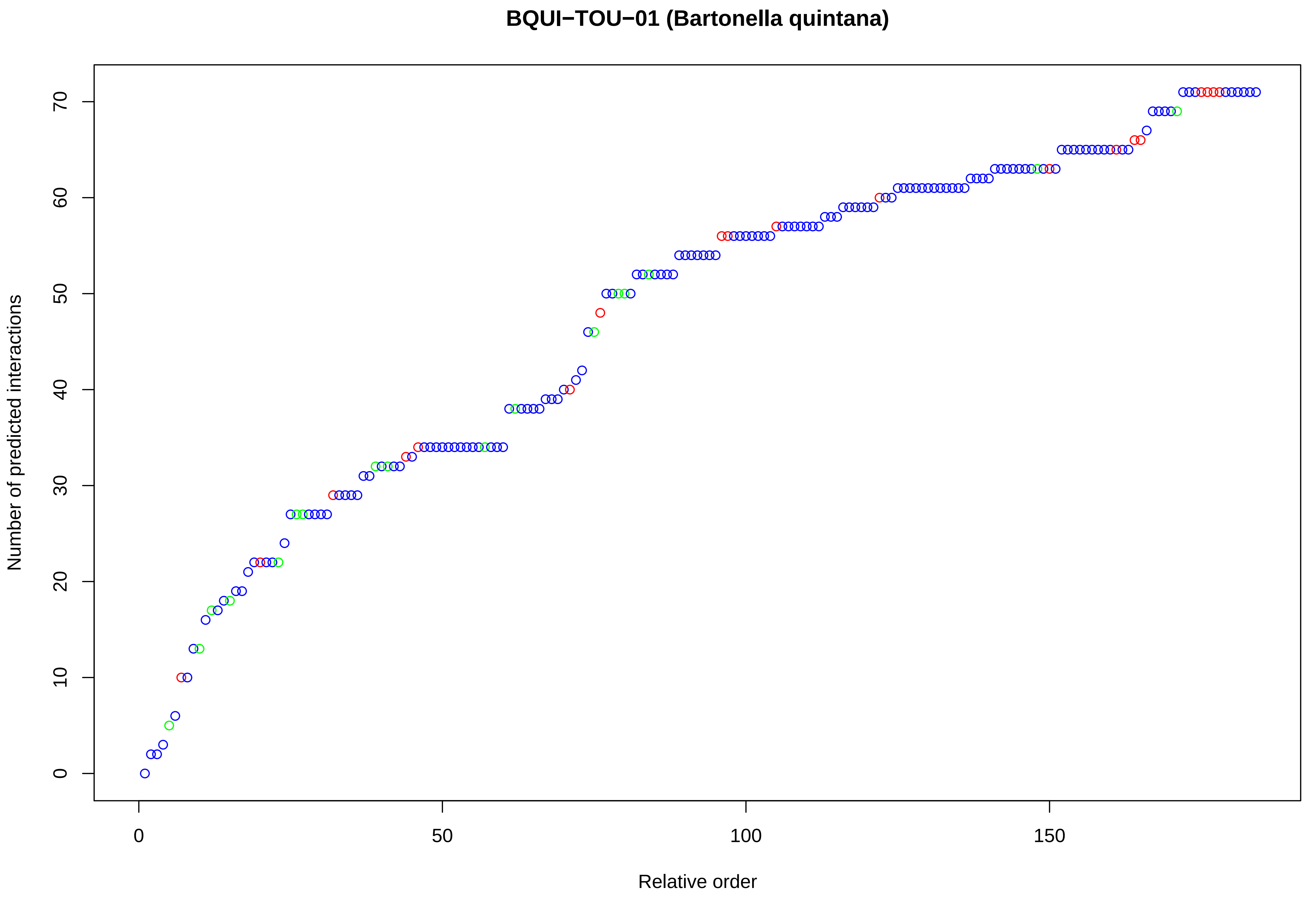
BQUI−TOU−01 (Bartonella quintana)
Number of predicted interactions
Relative order
0
10
20
30
40
50
60
70
0
50
100
150

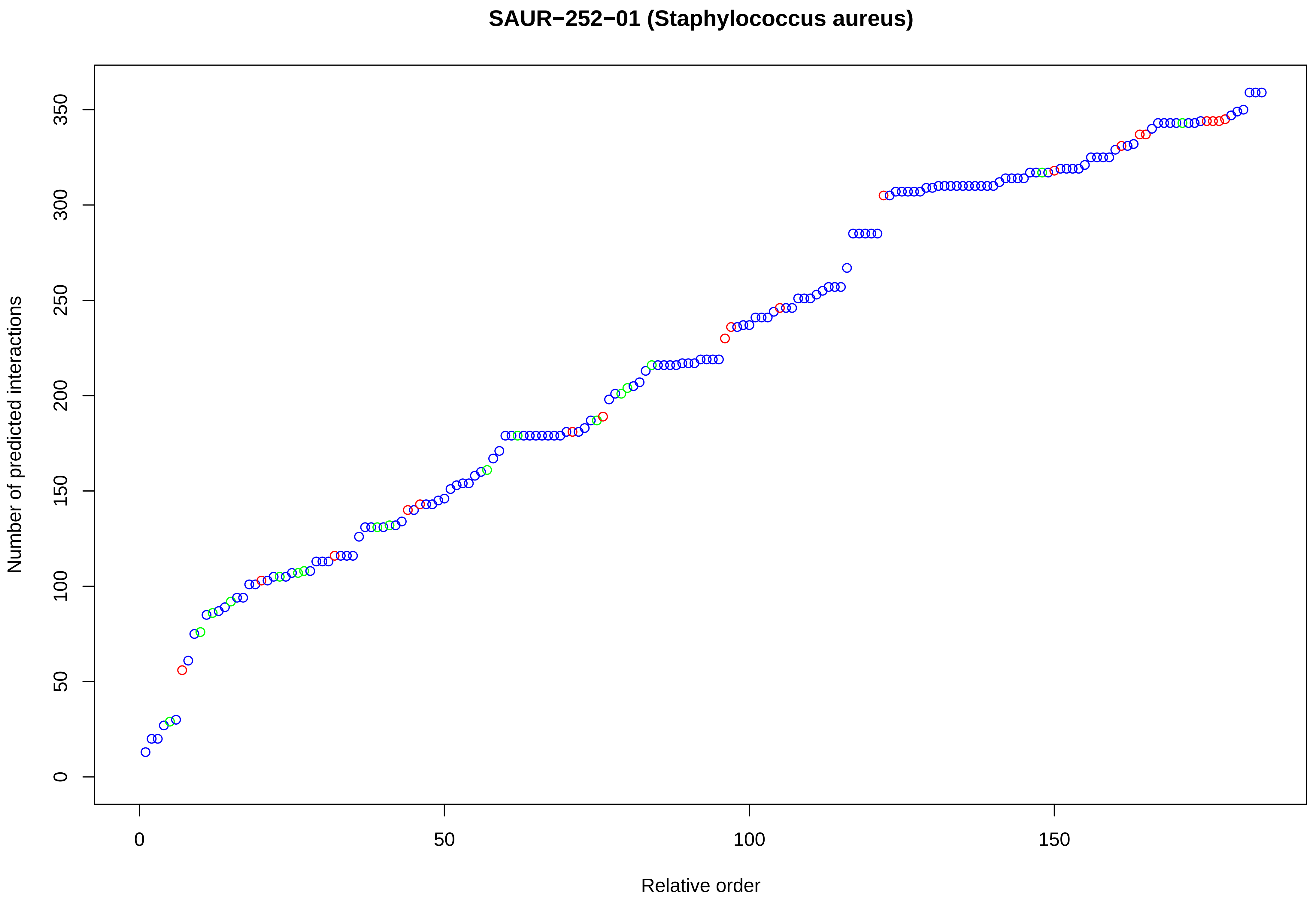
SAUR−252−01 (Staphylococcus aureus)
Number of predicted interactions
0
50
100
150
200
250
300
350
0
50
100
150
Relative order

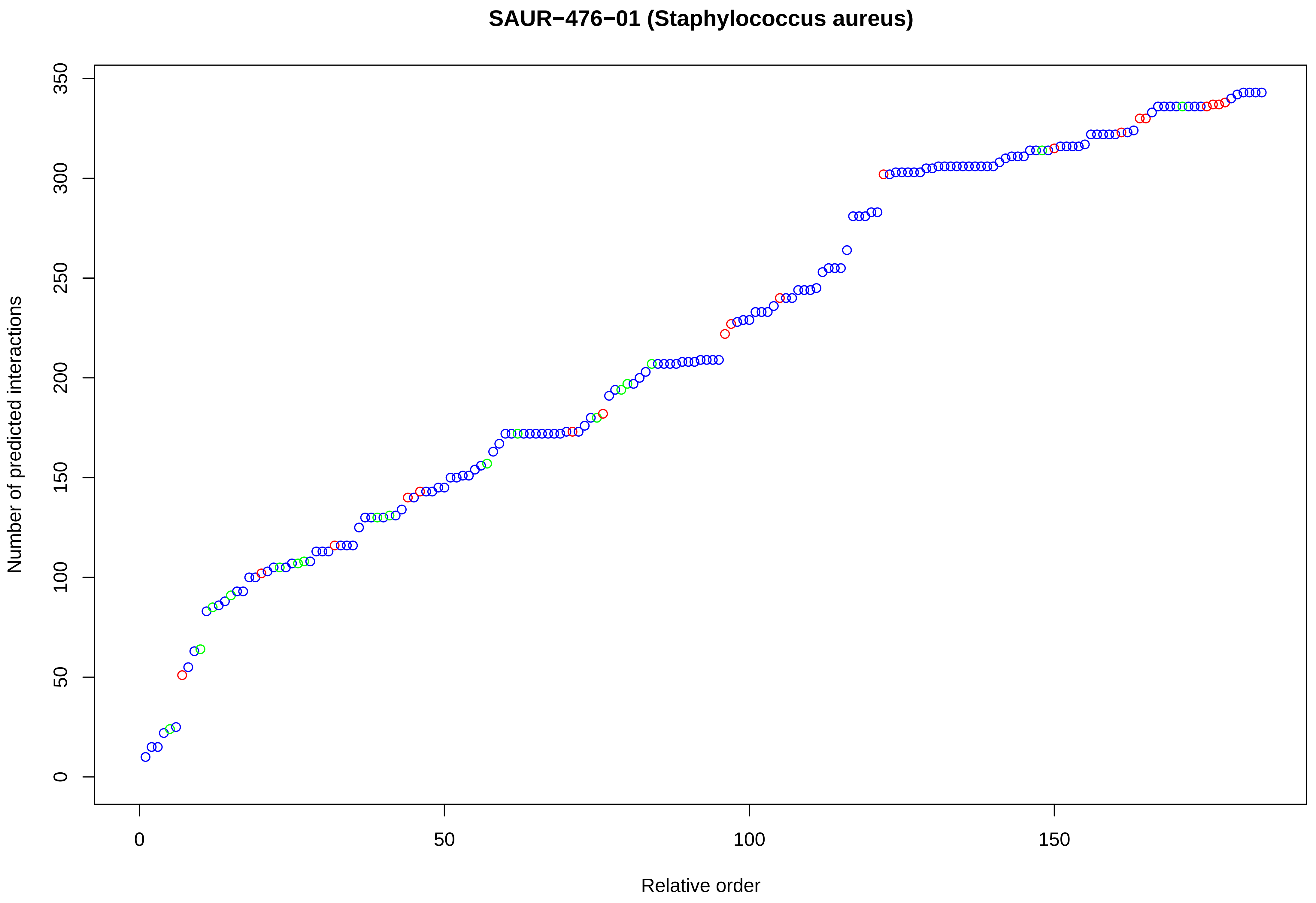

SAUR−476−01 (Staphylococcus aureus)
Number of predicted interactions
Relative order
0
50
100
150
200
250
300
350
0
50
100
150

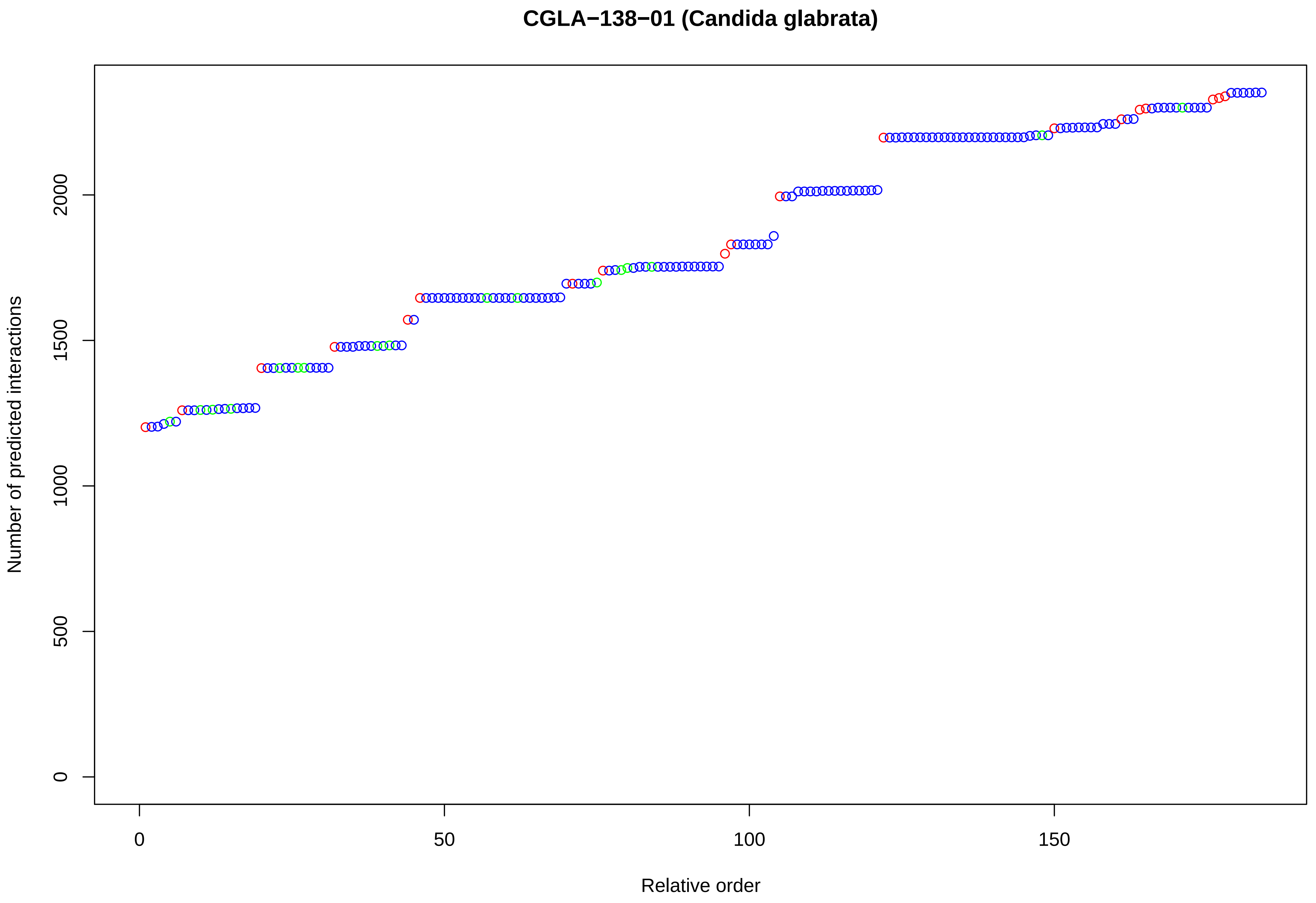

CGLA−138−01 (Candida glabrata)
Number of predicted interactions
Relative order
0
500
1000
1500
2000
0
50
100
150

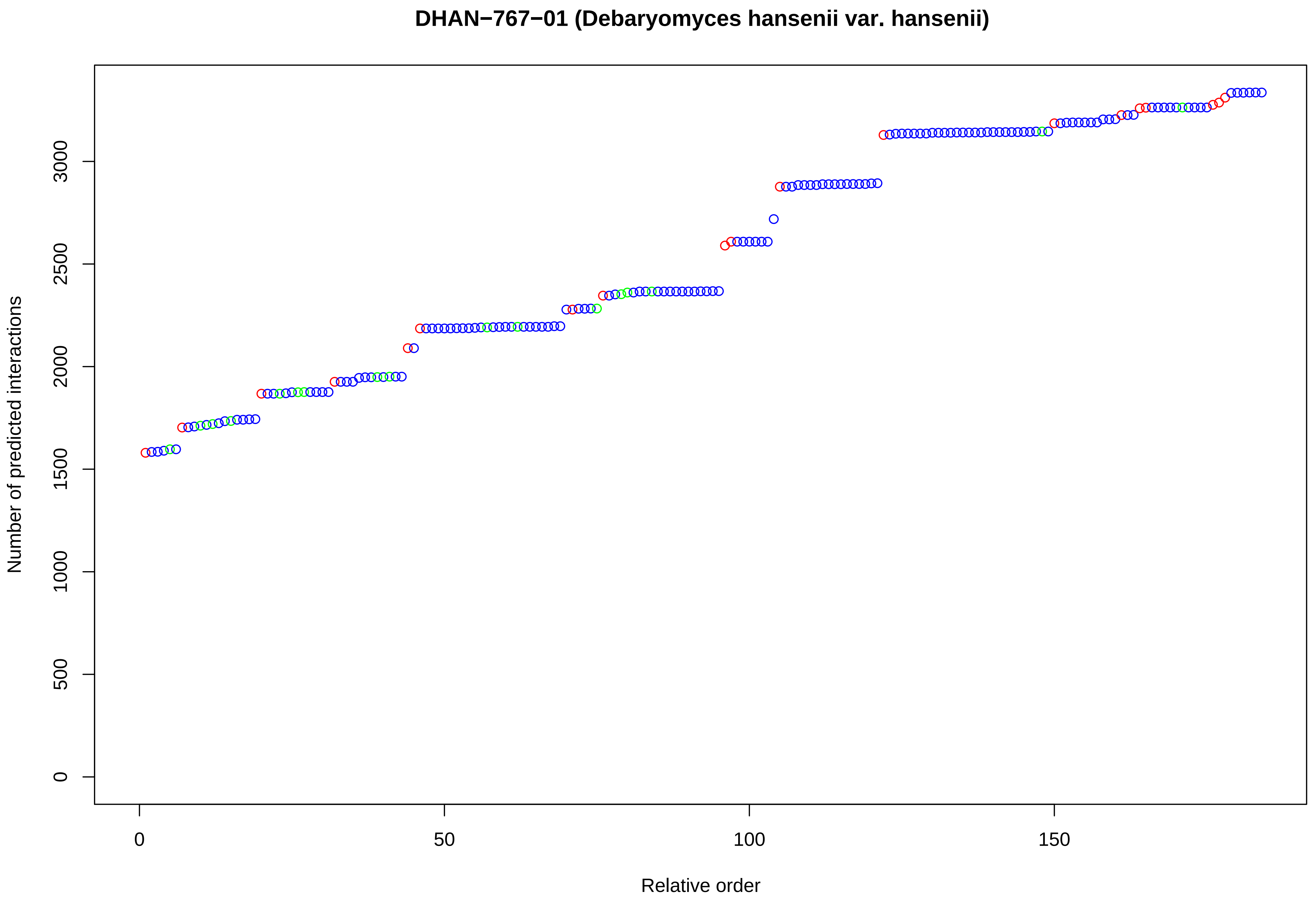

DHAN−767−01 (Debaryomyces hansenii var. hansenii)
Number of predicted interactions
0
500
1000
1500
2000
2500
3000
0
50
100
150
Relative order

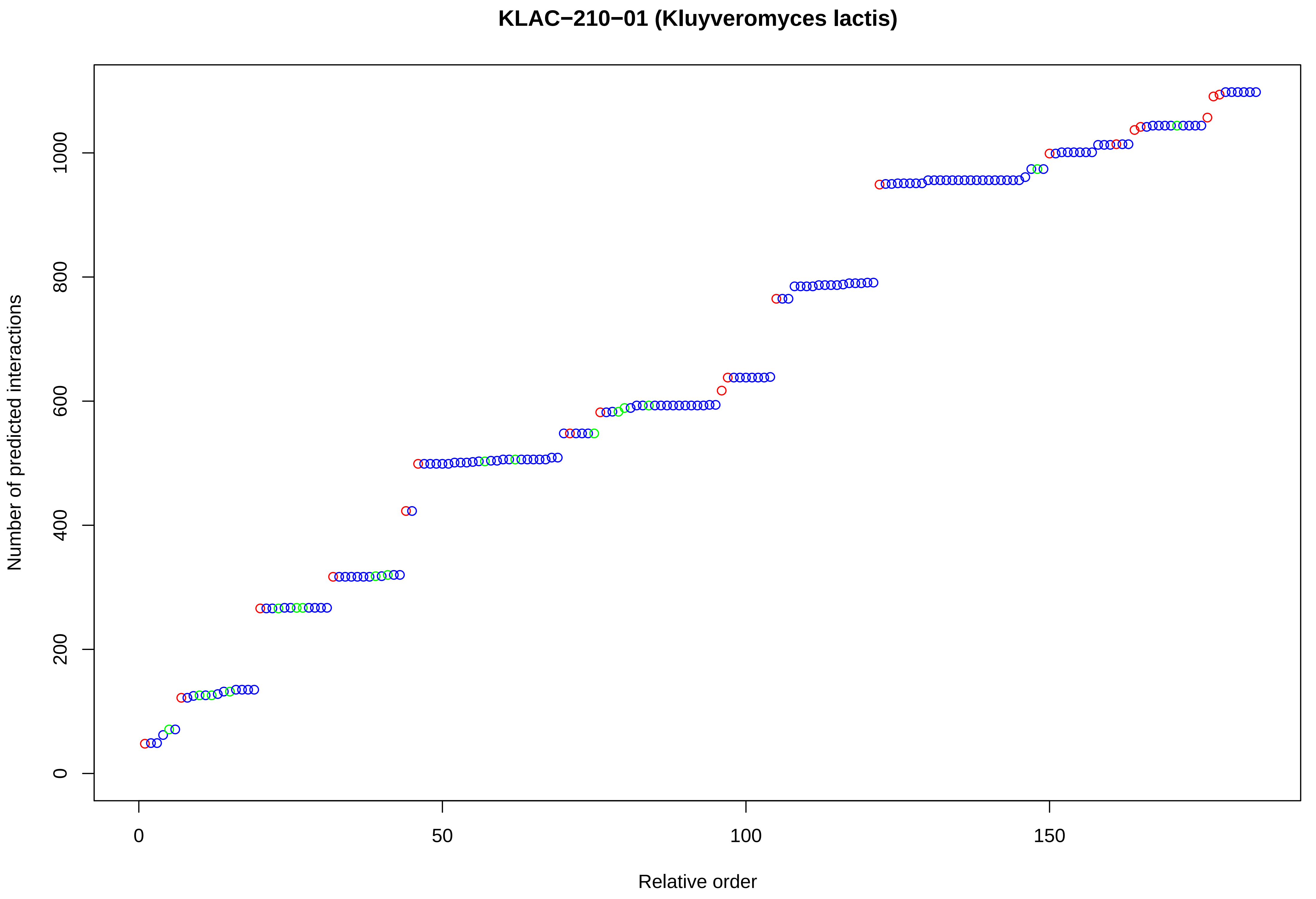

KLAC−210−01 (Kluyveromyces lactis)
Number of predicted interactions
0
200
400
600
800
1000
Relative order
0
50
100
150

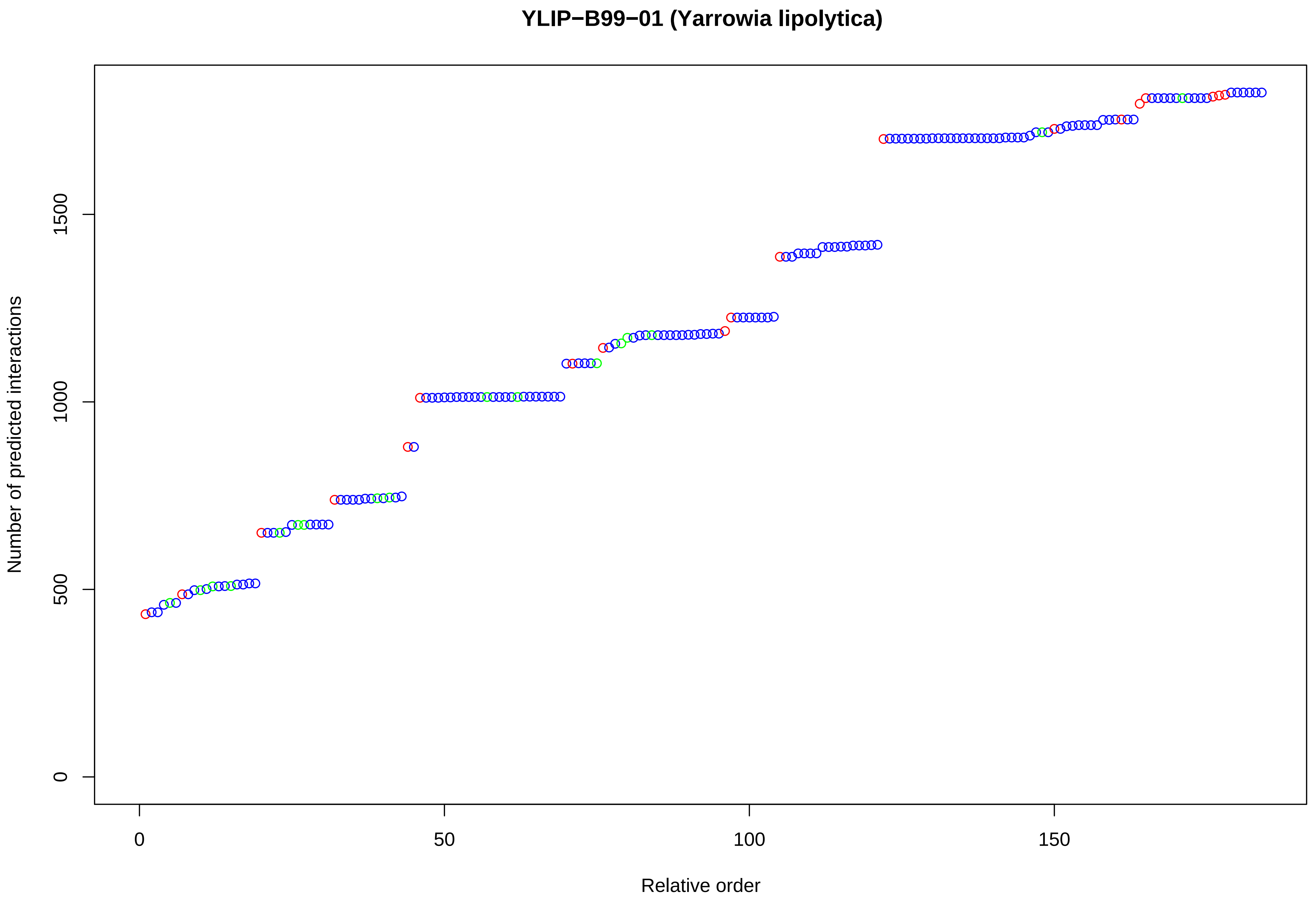
YLIP−B99−01 (Yarrowia lipolytica)
Number of predicted interactions
0
500
1000
1500
0
50
100
150
Relative order

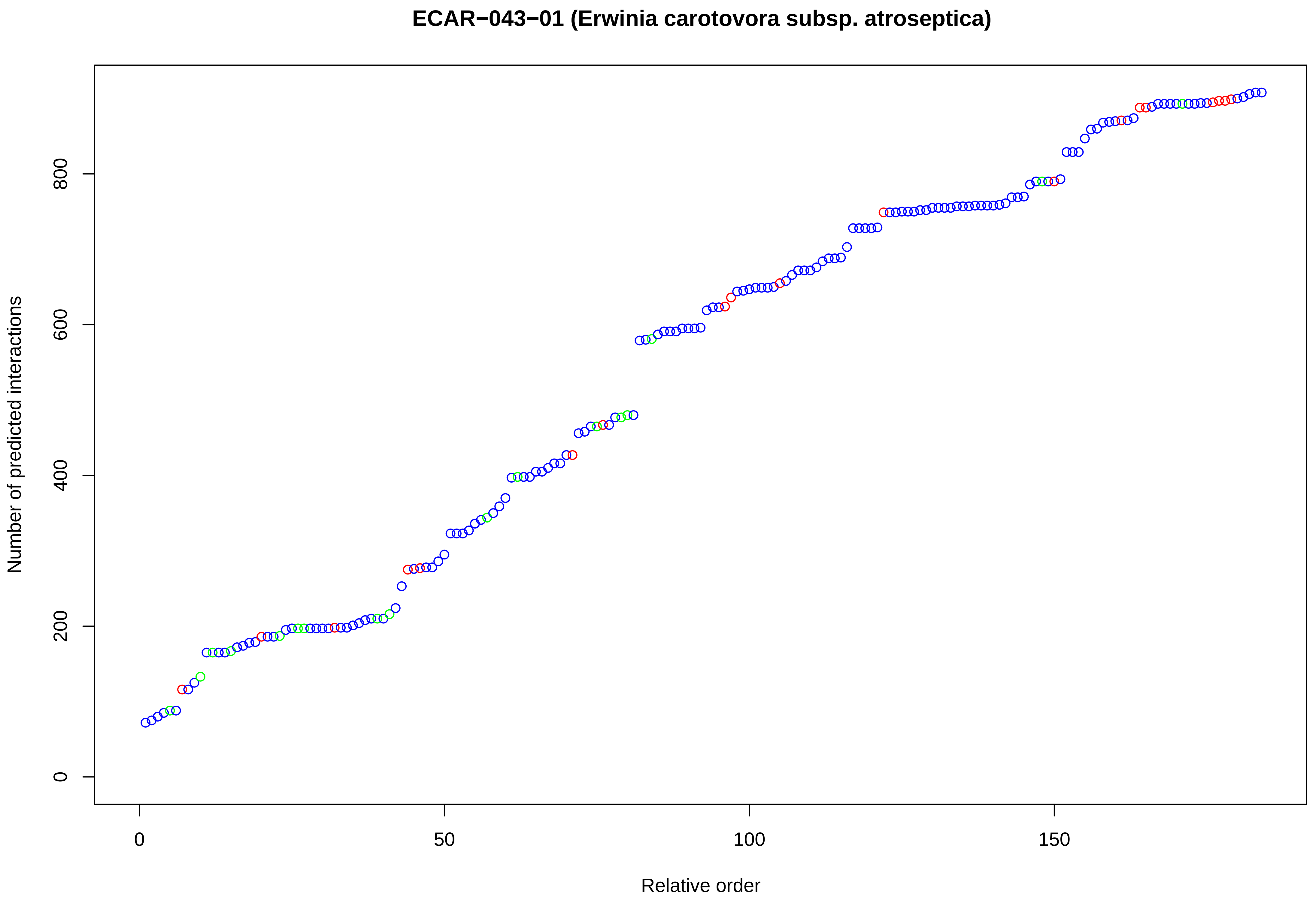
ECAR−043−01 (Erwinia carotovora subsp. atroseptica)
Number of predicted interactions
0
200
400
600
800
0
50
100
150
Relative order

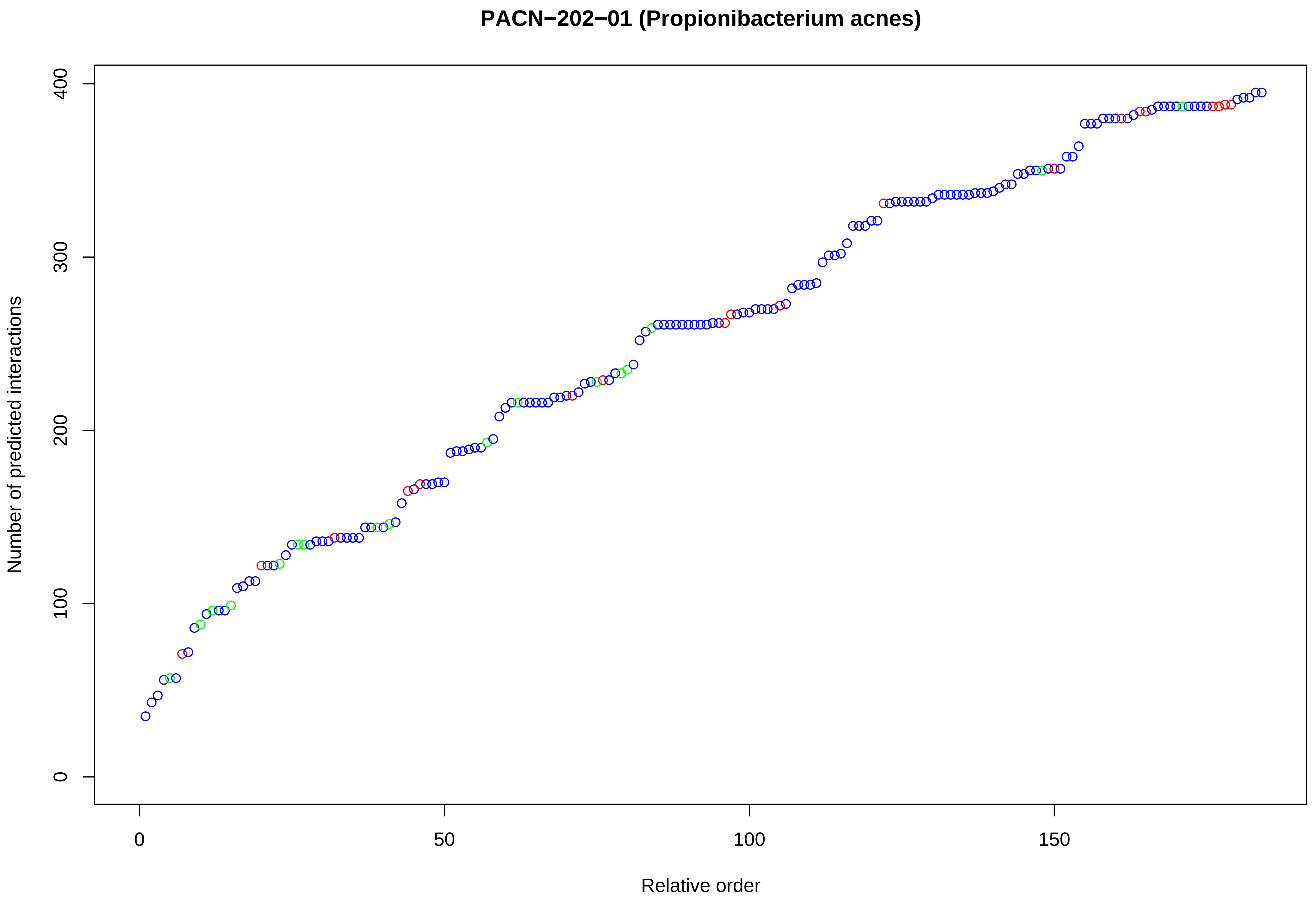

PACN−202−01 (Propionibacterium acnes)
Number of predicted interactions
0
100
200
300
400
Relative order
0
50
100
150

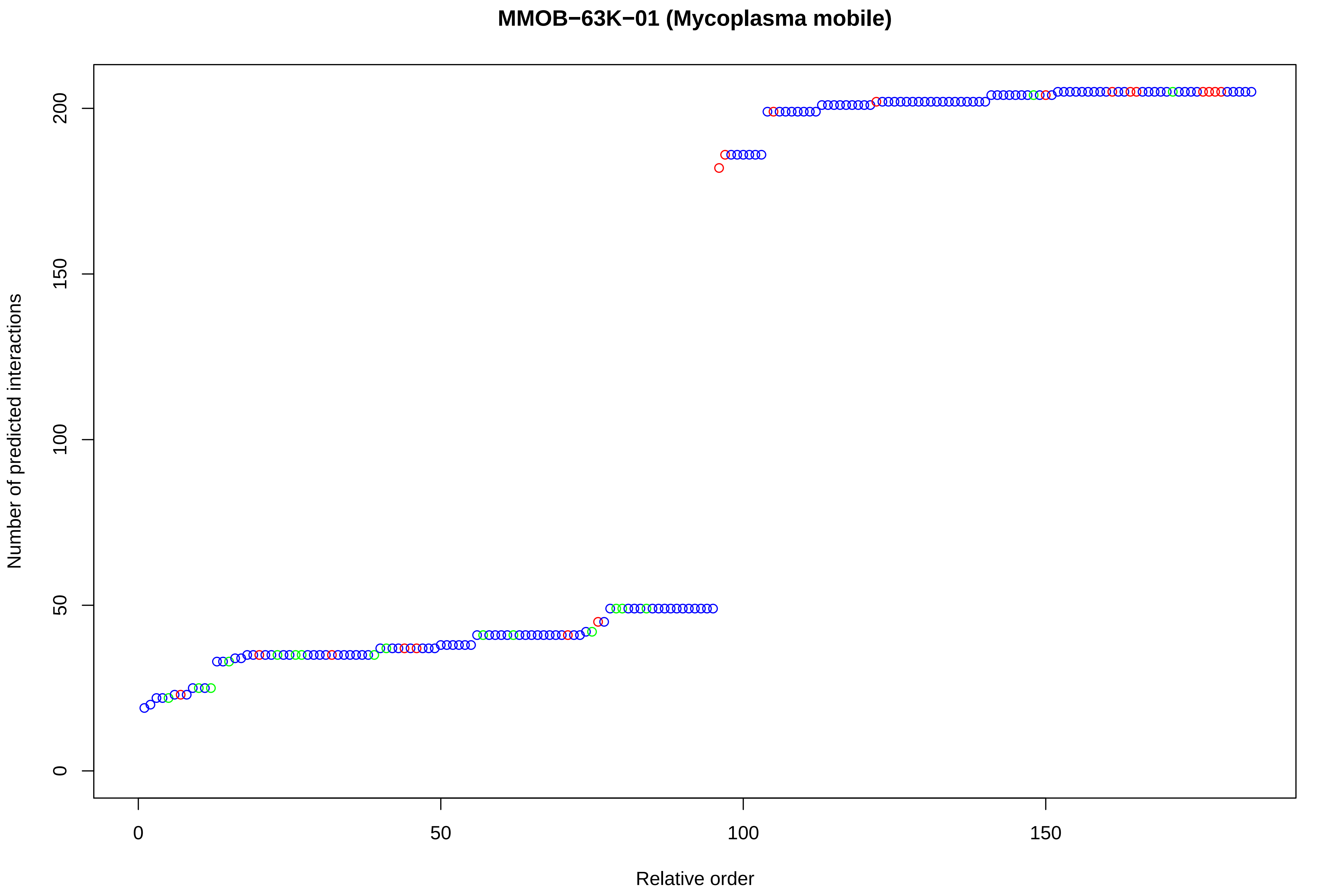

MMOB−63K−01 (Mycoplasma mobile)
Number of predicted interactions
Relative order
0
50
100
150
200
0
50
100
150

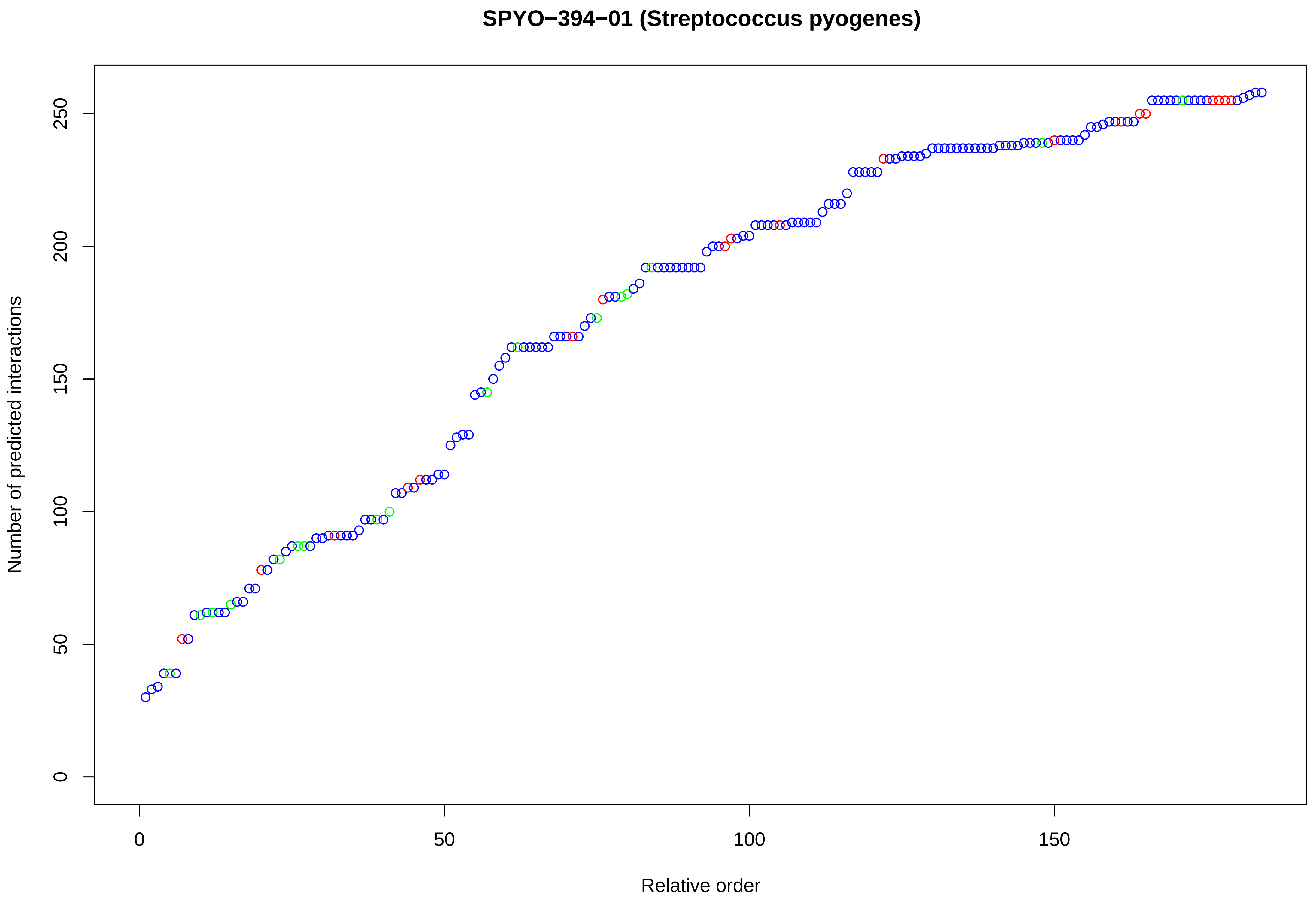
SPYO−394−01 (Streptococcus pyogenes)
Number of predicted interactions
Relative order
0
50
100
150
200
250
0
50
100
150

LXYL−B07−01 (Leifsonia xyli subsp. xyli)

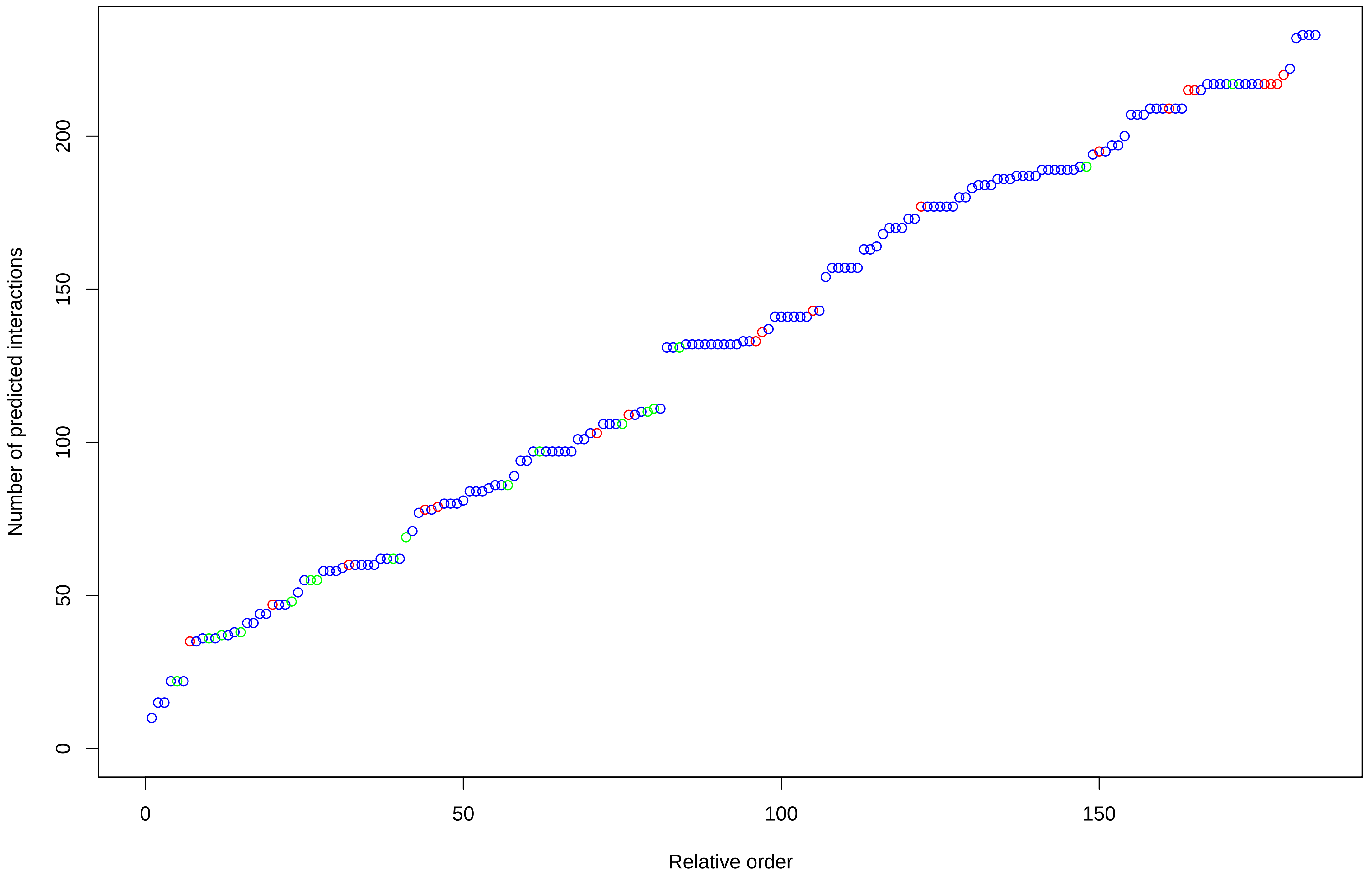

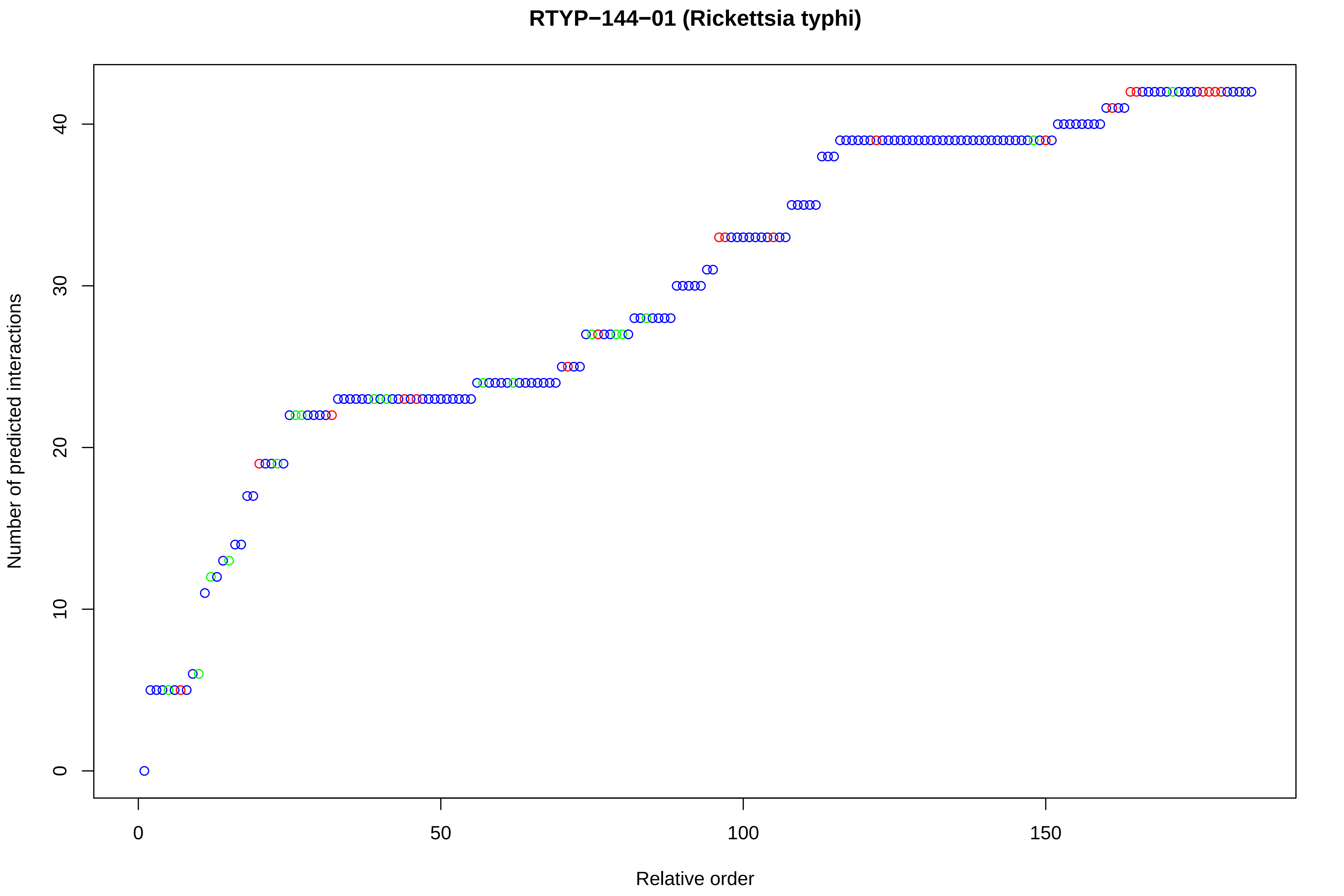

RTYP−144−01 (Rickettsia typhi)
Number of predicted interactions
Relative order
0
10
20
30
40
0
50
100
150
