# Supplementary material for: Denoising inferred functional association networks obtained by gene fusion analysis
Source: BMC Genomics. 2007 Dec 14;8:460. doi: 10.1186/1471-2164-8-460 (PMC2248599; doi:10.1186/1471-2164-8-460)
Supplement: Additional File 6 — Analysis of predicted interactions from Chlamydia trachomatis. Examples of previously characterised interactions in C. trachomatis that are detected by gene fusion analysis. Cases in bold are presumed as known; cases in purple are artifacts from genomic paralogy. [file 1471-2164-8-460-S6.doc]

| **#** | **gene-id** | **annotation** | **status** | **gene-id** | **annotation** |
| --- | --- | --- | --- | --- | --- |
| 1 | CT003 | glutamyl-tRNA (Gln) amidotransferase, subunit A EC 3.5.1.4 | predicted | CT762 | UDP-N-acetylmuramate-alanine ligase EC 6.3.2.8 D-alanine-D-alanine ligase A EC 6.3.2.4 |
| 2 | CT017 |  | unknown | CT127 | polysaccharide hydrolase EC 3.2.1.14 |
| 3 | CT106 | Ribosomal large subunit pseudouridine synthase C EC 4.2.1.70 | predicted | CT730 | Diaminohydroxyphosphoribosylaminopyrimidine deaminase EC 3.5.4.26 5-amino-6-(5-phosphoribosylamino)uracil reductase EC 1.1.1.193 |
| 4 | CT161 |  | unknown | CT174 |  |
| **5** | **CT170** | **Tryptophan synthase beta chain EC 4.2.1.20** | **known** | **CT171** | **Tryptophan synthase alpha chain EC 4.2.1.20** |
| **6** | **CT245** | **Pyruvate dehydrogenase E1 component alpha subunit EC 1.2.4.1** | **known** | **CT246** | **Pyruvate dehydrogenase E1 component beta subunit EC 1.2.4.1** |
| 7 | CT261 | DNA polymerase III epsilon chain EC 2.7.7.7 | predicted | CT545 | DNA polymerase III alpha chain EC 2.7.7.7 |
| 8 | CT261 | DNA polymerase III epsilon chain EC 2.7.7.7 | predicted | CT608 | S1 ATP-dependent helicase PcrA EC 3.6.1.- |
| 9 | CT261 | DNA polymerase III epsilon chain EC 2.7.7.7 | predicted | CT791 | Excinuclease ABC subunit C EC -.-.-.- |
| 10 | CT269 | UDP-N-acetylmuramoylalanylglutamyl DAP ligase EC 6.3.2.13 | predicted | CT613 | Dihydropteroate synthase FolP EC 2.5.1.15 2-amino-4-hydroxy-6-hydroxymethyldihydropteridine pyrophosphokinase FolK EC 2.7.6.3 |
| 11 | CT287 | tRNA (5-methylaminomethyl-2-thiouridylate)-methyltransferase EC 2.1.1.61 | predicted | CT093 | Riboflavin kinase EC 2.7.1.26 |
| ***12*** | ***CT327*** | ***Phosphoribosylanthranilate isomerase EC 5.3.1.24*** | ***known, paralog*** | ***CT170*** | ***Tryptophan synthase beta chain EC 4.2.1.20*** |
| **13** | **CT328** | **Triosephosphate isomerase EC 5.3.1.1** | **known** | **CT693** | **Phosphoglycerate kinase EC 2.7.2.3** |
| 14 | CT366 | 3-phosphoshikimate 1-carboxyvinyltransferase EC 2.5.1.19 | predicted | CT369 | 3-dehydroquinate synthase EC 4.6.1.3 |
| 15 | CT366 | 3-phosphoshikimate 1-carboxyvinyltransferase EC 2.5.1.19 | predicted | CT370 | 3-dehydroquinate dehydratase EC 4.2.1.10 Shikimate 5-dehydrogenase EC 1.1.1.25 |
| 16 | CT378 | Glucose-6-phosphate isomerase EC 5.3.1.9 | predicted | CT313 | Transaldolase EC 2.2.1.2 |
| 17 | CT379 |  | unknown | CT452 | Cytidylate kinase EC 2.7.4.14 |
| 18 | CT396 | DnaK protein | predicted | CT145 | Serine/threonine protein kinase EC 2.7.1.- |
| 19 | CT398 |  | unknown | CT108 |  |
| 20 | CT404 | N-methyltransferase EC -.-.-.- | predicted | CT106 | Ribosomal large subunit pseudouridine synthase C EC 4.2.1.70 |
| 21 | CT434 |  | unknown | CT027 | tRNA (guanine N-1) methyltransferase EC 2.1.1.31 |
| 22 | CT434 |  | unknown | CT462 | CMP-KDO synthetase homolog EC -.-.-.- |
| 23 | CT445 | Glutamyl-tRNA Synthetase EC 6.1.1.17 | predicted | CT004 | glutamyl-tRNA (Gln) amidotransferase, subunit B EC 3.5.1.4 |
| 24 | CT452 | Cytidylate kinase EC 2.7.4.14 | predicted | CT698 | Thiophene and furan oxidation protein ThdF |
| 25 | CT452 | Cytidylate kinase EC 2.7.4.14 | unknown | CT703 | D2 GTP-binding |
| 26 | CT480 | Dipeptide binding protein DppA | predicted | CT139 | Oligopeptide binding protein OppA |
| 27 | CT486 | Aminoacid-binding periplasmic protein precursor | predicted | CT129 | Amino acid transporter permease |
| 28 | CT489 | Glucose-1-phosphate adenylyltransferase EC 2.7.7.27 | predicted | CT815 | S1 Phosphohexomutase EC 5.4.2.- |
| *29* | *CT536* | *S1 DNA polymerase III epsilon chain homolog EC 2.7.7.7* | ***paralog*** | *CT545* | *DNA polymerase III alpha chain EC 2.7.7.7* |
| *30* | *CT536* | *S1 DNA polymerase III epsilon chain homolog EC 2.7.7.7* | ***paralog*** | *CT608* | *S1 ATP-dependent helicase PcrA EC 3.6.1.-* |
| *31* | *CT536* | *S1 DNA polymerase III epsilon chain homolog EC 2.7.7.7* | ***paralog*** | *CT791* | *Excinuclease ABC subunit C EC -.-.-.-* |
| 32 | CT564 | protein secretion system YscT homolog | predicted | CT091 | YopS Translocation Protein U |
| 33 | CT658 | Ribosomal large subunit pseudouridine synthase D EC 4.2.1.70 | predicted | CT730 | Diaminohydroxyphosphoribosylaminopyrimidine deaminase EC 3.5.4.26 5-amino-6-(5-phosphoribosylamino)uracil reductase EC 1.1.1.193 |
| 34 | CT687 | S1 Amino-acid aminotransferase class V EC 2.6.1.- | unknown | CT217 |  |
| 35 | CT701 | preprotein translocase subunit SecA | unknown | CT141 |  |
| 36 | CT777 | 8-amino-7-oxononanoate synthase EC 2.3.1.47 | predicted | CT238 | Malonyl CoA-acyl carrier protein transacylase EC 2.3.1.39 |
| **37** | **CT821** | **Succinyl-CoA synthetase beta chain EC 6.2.1.5** | **known** | **CT822** | **Succinyl-CoA synthetase alpha chain EC 6.2.1.5** |
| 38 | CT829 | S1 methyltransferase homolog EC 2.1.1.- | predicted | CT024 | HemK modification methylase EC 2.1.1.72 |
| 39 | CT829 | S1 methyltransferase homolog EC 2.1.1.- | predicted | CT208 | 3-deoxy-d-manno-octulosonic-acid transferase EC 2.-.-.- |
| 40 | CT840 |  | unknown | CT844 | S1 Cyclic amidine deaminase EC 3.5.4.- |

Notes: S1: Similar to; D2: Domain
