# Supplementary material for: Denoising inferred functional association networks obtained by gene fusion analysis
Source: BMC Genomics. 2007 Dec 14;8:460. doi: 10.1186/1471-2164-8-460 (PMC2248599; doi:10.1186/1471-2164-8-460)
Supplement: Additional File 7 — Examples of predicted interactions from Arabidopsis thaliana. The species of origin is also given for the composite proteins. [file 1471-2164-8-460-S7.doc]

| **Description of interaction** | **Component 1 in *A. thaliana*** | **Component 2 in *A. thaliana*** | **Composite**  **Protein/s** |
| --- | --- | --- | --- |
| Components of the NADH-ubiquinone oxidoreductase complex | NDUV2_ARATH (NADH-ubiquinone oxidoreductase 24 kDa subunit) 255aa | Q9FNN5_ARATH (NADH dehydrogenase) 486aa | Q9ZBV8_STRCO (Putative respiratory chain oxidoreductase) *Streptomyces coelicolor* (Bacteria) 646aa |
| The two proteins catalyze different steps in the L-arginine biosynthesis pathway | ARGC_ARATH (Probable N-acetyl-gamma-glutamyl-phosphate reductase) 401aa | Q3E9P2_ARATH (acetylglutamate kinase / glutamate 5-kinase) 613aa | ARG56_SCHPO (Protein arg11) *Schizosaccharomyces pombe* (Eukaryota) 885aa |
| Both proteins catalyze different reactions from the glycerophospholipid metabolism pathway | Q7XJN4_ARATH (Glycerol-3-phosphate dehydrogenase) 433aa | LPAT1_ARATH (1-acyl-sn-glycerol-3-phosphate acyltransferase 1) 356aa | Q8F736_LEPIN (Glycerol-3-phosphate dehydrogenase) *Leptospira interrogans*  (Bacteria) 669aa |
| Both proteins are likely to be components of molybdopterin synthase | Q9S7A3_ARATH (Molybdopterin synthase small subunit) 96aa | O22827_ARATH (Putative molybdopterin synthase large subunit) 198aa | Q7TWQ6_MYCBO (Probable MoaD-MoaE fusion protein MoaX) *Mycobacterium bovis* (Bacteria) 221aa |
| Possibly, both proteins are involved in spliceosome activity | O80897_ARATH (Putative spliceosome associated protein) 277aa | Q9LUK6_ARATH (Similarity to RNA-binding protein) 501aa | Q19335_CAEEL (Hypothetical protein) *Caenorhabditis elegans* (Eukaryota) 222aa |
| Possibly both proteins participate in maintenance and assembly of ribosome subunits | Q1KS85_ARATH (Hypothetical protein) 495aa | Q6NME0_ARATH (Hypothetical protein) 311aa | NOC3_SCHPO (Nucleolar complex-associated protein 3) *Schizosaccharomyces pombe* (Eukaryota) 747aa |
